# Abstracts

**DOI:** 10.1002/jia2.25148

**Published:** 2018-07-26

## Oral Abstracts

## TUAA0101

### Two‐component self‐assembling nanoparticle vaccines that present multiple HIV‐1 envelope trimers

**P. Brouwer^1^; D. Ellis^2^; A. Antanasijevic^3^; A. Yasmeen^4^; Z. Berndsen^3^; T. Bijl^1^; J. Burger^1^; B. Nickerson^2^; C. Cottrell^3^; J. Allen^5^; I. Bontjer^1^; M. Crispin^5^; D. Baker^2^; A. Ward^3^; J. Moore^4^; P.‐J. Klasse^4^; N. King^2^ and R. Sanders^1^**

^1^Academic Medical Center, Amsterdam, Netherlands, ^2^University of Washington, Seattle, United States, ^3^The Scripps Research Institute, San Diego, United States, ^4^Weill Medical College of Cornell University, New York, United States, ^5^University of Southampton, Biological Sciences, Southampton, United Kingdom

**Background:** The development of soluble native‐like HIV‐1 envelope trimers (SOSIP trimers) has enabled the induction of neutralizing antibodies against neutralization‐resistant (Tier‐2) primary HIV‐1 strains in several animal models. However, these neutralizing antibody responses are relatively weak, short‐lived, and narrow in specificity. Displaying antigens in a multivalent fashion on nanoparticles or virus‐like particles is a well‐established strategy to increase their immunogenicity. Here, we present the design and characterization of two‐component protein nanoparticles displaying twenty SOSIP trimers.

**Methods:** Using computational protein structure prediction the trimeric component of a two‐component self‐assembling protein nanoparticle (I53‐50NP) was redesigned to allow SOSIP trimer fusion. The resulting fusion proteins (SOSIP‐I53‐50A) were expressed in 293F cells and affinity purified with the trimer‐specific antibody PGT145 to obtain native‐like trimers. To obtain SOSIP‐presenting I53‐50NPs, fusion proteins where mixed in vitro with the other component of the two‐component nanoparticle, I53‐50B

**Results:** The nanoparticles self‐assemble with high efficiency into stable, monodisperse and well‐ordered icosahedral particles as observed by, size‐exclusion chromatography, negative‐stain electron microscopy, cryo‐EM and dynamic light scattering. The protruding SOSIP trimers maintain their antigenic integrity as observed by surface plasmon resonance and, in contrast to their soluble counterparts, induce strong activation of cognate B cells in vitro. Rabbits immunized with these nanoparticles induced significantly higher levels of neutralizing antibodies than the corresponding soluble SOSIP trimers (˜50‐fold higher median titer after one immunization) or SOSIP trimers presented on other nanoparticle platforms.

**Conclusions:** The design of the SOSIP‐I53‐50NP allows for selection of native‐like trimers prior to nanoparticle assembly. This may provide a considerable advantage over particles that are assembled intracellularly (i.e. ferritin particles and VLPs) which may naturally present a significant population of non‐native trimers. Two‐component I53‐50 self‐assembling nanoparticles represent a versatile platform for vaccine strategies aimed at increasing and broadening neutralizing antibody responses against viral envelope proteins.

## TUAA0102

### A CD4‐mimetic compound enhances vaccine efficacy against stringent immunodeficiency virus challenge

**N. Madani^1^; A. Princiotto^2^; L. Mach^3^; J. Richard^4^; B. Hora^5^; C. Zhao^6^; T. Bradley^7^; B. Melillo^8^; A. Finzi^9^; B. Haynes^10^; A. Smith^8^; S. Santra^11^; J. Moss^12^; M. Baum^12^ and J. Sodroski^2^**

^1^Harvard Medical School/Dana‐Farber Cancer Institut, Cancer Immunology and Virology, Newton, United States, ^2^Dana‐Farber Cancer Institute, Cancer Immunology and Virology, Boston, United States, ^3^Beth Israel Deaconess Medical Center, Harvard Medical School, Center for Virology and Vaccine Research, Boston, United States, ^4^Université de Montréal, Department of Microbiology, Infectiology and Immunology, Montreal, Canada, ^5^Duke Human Vaccine Institute, Department of Medicine, Durham, United States, ^6^Dana‐Farber Cancer Institute, Cancer Immunology and VIrology, Boston, United States, ^7^Duke University Medical Center, Dept of Medicine, Durham, United States, ^8^University of Pennsylvania, Chemistry, Philadelphia, United States, ^9^Université de Montréal, 6Department of Microbiology, Infectiology and Immunology, Montreal, Canada, ^10^Duke Human Vaccine Institute, Department of Immunology, Durham, United States, ^11^Beth Israel Deaconess Medical Center, Boston, United States, ^12^Oak Crest Institute of Science, Monrovia, United States

**Background:** Preventing sexual transmission of human immunodeficiency virus (HIV‐1) is a global priority. HIV‐1 envelope glycoproteins (Env) mediate virus entry through a series of conformational changes triggered by binding to the receptors, CD4 and CCR5/CXCR4. Broadly neutralizing antibodies that recognize conserved elements of the closed Env are potentially protective, but are elicited inefficiently during natural HIV‐1 infection or by vaccination. Small‐molecule CD4‐mimetic compounds (CD4‐mc) engage the CD4‐binding pocket on the gp120 exterior Env, directly inactivate HIV‐1, and induce Env epitopes that are highly sensitive to neutralization by vaccine‐induced antibodies.

**Methods:** For cell culture studies, viruses containing primary HIV‐1 Envs were incubated with the different concentrations of BNM‐III‐170, a CD4‐mc. Virus neutralization by various antibodies or sera was tested in the presence or the absence of CD4‐mc. Three groups of monkeys were used in the present study. The monkeys were boosted with human serum albumin (HSA) (Group 1) or HIV‐1CH505 gp120 (Groups 2 and 3) either two weeks (Challenges 1 and 2) or four weeks (Challenge 3) before the SHIV‐C5 challenge. Group 1 and 3 were also treated with 300 uM CD4‐mc.

**Results: ** Small‐molecule CD4‐mimetic compounds (CD4mc) bind the HIV‐1 gp120 Env and promote conformational changes similar to those induced by CD4, exposing conserved Env elements to antibodies. Our results show that a CD4mc synergizes with antibodies elicited by monomeric HIV‐1 gp120 to protect monkeys from multiple high‐dose intrarectal challenges with a heterologous simian‐human immunodeficiency virus (SHIV). The protective immune response persists for at least six months after vaccination.

**Conclusions: **CD4‐mimetic compounds directly interrupt HIV‐1 infection and dramatically enhance the neutralizing activity of antibodies that can be elicited in monkeys with currently available Env immunogens. CD4mc should increase the protective efficacy of any HIV‐1 Env vaccine that elicits antibodies against CD4‐induced conformations of Env. Based on these results, macaque‐sized intravaginal rings for sustained‐release topical delivery of CD4‐mc are being developed and evaluated in vitro in preparation for pharmacokinetics and efficacy studies in a macaque model. Used as microbicides, CD4‐mimetic compounds might increase the protective efficacy of HIV‐1 vaccines. Our results set the stage for clinical studies in humans at risk of sexually acquired HIV‐1 infection.

## TUAA0103

### Oral MVA/protein HIV vaccination with a needle‐free injector induces robust systemic and mucosal antibody responses in rhesus macaques

**A. Jones^1^; R. Das^2^; L. Wyatt^3^; C. LaBranche^4^; X. Shen^5^; G. Tomaras^5^; D. Montefiori^5^; B. Moss^3^; J. Clements^6^; D. Barouch^7^; P. Kozlowski^8^; R. Varadarajan^2^ and R.R. Amara^1^**

^1^Emory University, Yerkes National Primate Research Center, Decatur, United States, ^2^Indian Institue of Science, Bangalore, India, ^3^Laboratory of Viral Diseases, NIAID, NIH, Bethesda, United States, ^4^Duke University, Department of Surgery, Durham, United States, ^5^Duke University, Durham, United States, ^6^Tulane University School of Medicine, New Orleans, United States, ^7^Beth Israel Deaconess Medical Center, Harvard Medical School, Boston, United States, ^8^Louisiana State University Health Sciences, New Orleans, United States

**Background:** In the immediate hours and days post mucosal transmission, HIV‐1 is considered to be at a vulnerable state due to localized replication and low or unestablished viral reservoirs. Thus, HIV vaccines should induce a strong and long‐lasting mucosal immune response and mucosal vaccination would be an ideal route to achieve this. Here we evaluate the immunogenicity and efficacy of oral vaccination and compare it with systemic vaccinations in rhesus macaques (RM).

**Methods:** For oral vaccination, we immunized a group of RM (n = 5) via sublingual and buccal tissue (SL/B) routes using a modified needle‐free injector. Animals were immunized twice with modified vaccinia Ankara (MVA) expressing HIV‐1 Gag, Pol and envelope antigens, followed by two immunizations with a recombinant trimeric gp120 immunogen along with the mucosal adjuvant dmLT. A second group (n = 6) received the immunizations via the conventional intradermal (MVA) and subcutaneous (protein) routes (ID/SC). All animals were challenged intrarectally at around five months after the final immunization with a pathogenic SHIV162P3 for a maximum of six challenges.

**Results: ** Systemic immunization (ID/SC) induced strong IgG responses in serum and mucosal secretions (rectal, vaginal, and salivary secretions) but failed to induced IgA responses. Impressively, needle‐free oral immunization generated a robust HIV Env‐specific IgG and IgA antibody response both in blood and mucosal compartments that are at least tenfold higher compared to responses in ID/SC immunized animals. The vaccine induced IgG responses showed a strong cross‐reactivity to a global panel of gp70‐V1V2 scaffolds. Following intrarectal challenge, all five controls became infected by three challenges and we observed a significant delay in acquisition of infection in both vaccinated groups (*p* = 0.02 for oral, *p* = 0.007 for ID/SC and *p* = 0.002 for combined) compared to unvaccinated controls. Two of the six ID/SC animals remained uninfected at the end of six challenges.

**Conclusions: **Our results show that needle‐free injection of the sublingual and buccal tissues acts as an effective and practical route to generate both systemic and mucosal antibodies via vaccination. They also show that MVA prime followed by a gp120 trimer boost can provide a significant protection against intrarectal SHIV challenges.

## TUAA0104

### Long‐term data from APPROACH: phase 1/2a randomized, double‐blind, placebo‐controlled study evaluating safety/tolerability and immunogenicity of vaccine regimens using combinations of Ad26.Mos.HIV, MVA‐mosaic and gp140 envelope protein

**F. Tomaka^1^; D. Stieh^2^; D. Barouch^3^; M. Robb^4,5^; N. Michael^4^; G. Tomaras^6^; G. Alter^7^; J. McElrath^8^; L. Lavreys^9^; S. Nijs^9^; K. Callewaert^2^; J. Hendriks^2^; Z. Euler^2^; M. Pau^2^ and H. Schuitemaker^2^**

^1^Janssen Research & Development LLC, Titusville, United States, ^2^Janssen Vaccines & Prevention B.V., Leiden, Netherlands, ^3^Beth Israel Deaconess, Medical Center, Harvard Medical Center, Boston, United States, ^4^Military HIV Research Program, Walter Reed Army Institute of Research, Silver Spring, United States, ^5^Henry M. Jackson Foundation for the Advancement of Military Medicine, Bethesda, United States, ^6^Duke Human Vaccine Institute, Duke University, Durham, United States, ^7^Ragon Institute of MGH, MIT, and Harvard, Cambridge, United States, ^8^Vaccine and Infectious Diseases Division, Fred Hutchinson Cancer Research Center, Seattle, United States, ^9^Janssen Infectious Diseases B.V., Beerse, Belgium

**Background:** Globally, 1.8 million new HIV infections in 2016 demonstrate the need for a prophylactic HIV vaccine, but none currently exist. APPROACH (NCT02315703) investigates various vaccine regimens (comprising viral vectors with global mosaic HIV‐1 Env, Gag and Pol transgenes and a soluble clade C gp140 trimeric envelope protein), that aim to elicit protective immunity against multiple clades of HIV‐1. Week 28 and 52 data showed Ad26.Mos.HIV double prime, and Ad26.Mos.HIV or MVA‐Mosaic boost regimens combined with gp140 Env protein were immunogenic and well tolerated. We here present data on durability of immune responses.

**Methods:** Healthy, uninfected participants were randomized into seven vaccine regimens, or a placebo and administered Ad26.Mos.HIV double prime (Weeks 0 and 12) and a double boost of either Ad26.Mos.HIV or MVA‐Mosaic, with high‐ or low‐dose aluminium‐phosphate adjuvanted gp140 Env protein (Weeks 24 and 48). Vaccine responders were participants exhibiting an immunological response >LLOQ (if baseline is <LLOQ/missing) or threefold increase from baseline (if ≥LLOQ). Week 78 and 96 endpoints were immunogenicity and safety/tolerability.

**Results: ** 393 participants from the US, East‐Africa, South Africa and Thailand were randomised and received ≥1 dose of study vaccine (n = 48 to 50/group; see figure for regimens). Median age 29 years; 54% male; 54% Black, 27% White and 16% Asian.

Participants in all vaccine regimens showed humoral response rates >92% at Week 78 (30 weeks after fourth dose). Rates of antibody decay after the fourth vaccination exhibited regimen‐independent decrease in magnitude. Groups boosted with Ad26.Mos.HIV+gp140 Env (high‐ or low‐dose) maintained 100% response rate (high‐dose (n = 44); 95% CI=91.96% to 100%; low‐dose (n = 39); 95% CI=90.97% to 100%).

Bridging with a parallel non‐human primate (NHP) challenge study showed for Ad26.Mos.HIV+gp140 Env high‐dose boost group, autologous ELISA responses at Week 78 were 4.3‐fold higher in humans than in partially protected NHPs at time of challenge.

During the post‐fourth vaccination period, safety appeared to remain favorable for all groups.

**Conclusions: **All participants in Ad26.Mos.HIV prime with Ad26.Mos.HIV+gp140 Env (high‐ and low‐dose protein) groups achieved high and persistent immune responses that were maintained until Week 78 (30 weeks after fourth dose). Follow‐up of participants that received Ad26 + gp140 Env boosted regimens will continue (five‐year).

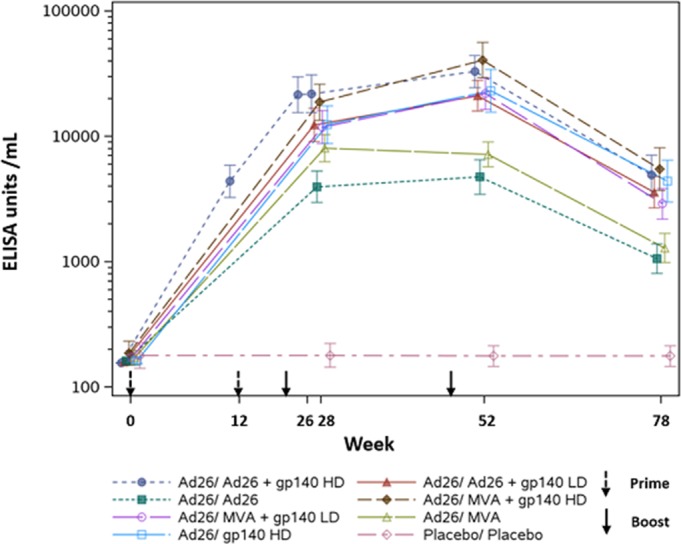

**Abstract TUAA0104‐Figure 1. ELISA ‐ Total IgG gp140 Env Clade C.**

## TUAA0105

### HPX1002/IPCAVD010: a randomized controlled trial evaluating the safety and immunogenicity of shorter and simpler vaccine schedules using Ad26.Mos.HIV combined with gp140 Env protein

**K. Stephenson^1,2^; J. Ansel^1^; S. Walsh^1^; C.S. Tan^1^; D. Ananos^1^; A. Yanez^2^; L. Peter^1^; F. Tomaka^3^; D. Stieh^3^; J. Hendriks^3^; S. Nijs^3^; C. Truyers^3^; M. Grazia Pau^3^; M. Seaman^1^; B. Walker^2^; H. Schuitemaker^3^ and D. Barouch^1,2^**

^1^BIDMC/Harvard, Center for Virology & Vaccine Research, Boston, United States, ^2^Ragon Institute of MGH, MIT and Harvard, Cambridge, United States, ^3^Janssen Vaccines AG, Bern, Switzerland

**Background:** A large Phase 2b proof‐of‐concept study called “Imbokodo” was initiated in 2017 to assess the preventive vaccine efficacy of a prime/boost regimen using mosaic antigens encoded by Ad26 and gp140 Env protein in HIV‐uninfected women in sub‐Saharan Africa. The vaccine schedule in Imbokodo involves four vaccination visits over 48 weeks, a long regimen that may be a factor in limiting adherence. We explored in HPX1002/IPCAVD010 whether shorter, simpler regimens might be equally immunogenic to an Imbokodo‐like regimen.

**Methods:** HPX1002/IPCAVD010 was a randomized, placebo‐controlled, double‐blind Phase 1 study in 36 HIV‐uninfected adults (12 per arm) to evaluate the safety and immunogenicity of three different vaccine regimens with Ad26 vectors expressing mosaic Env and Gag/Pol antigens (Ad26.Mos.HIV) and aluminium‐phosphate adjuvanted Clade C gp140 trimeric envelope protein (gp140 Env). Group 1 received Ad26 double prime at Weeks 0 and 12 and a double boost with Ad26 + gp140 at Weeks 24 and 48. Group 2 received Ad26 + gp140 at weeks 0, 12 and 24. Group 3 received Ad26 at Wk 0, and Ad26 + gp140 at weeks 8 and 24. The study was conducted at Beth Israel Deaconess Medical Center in Boston, MA, USA. Data from Baseline, 28 and 52 were analyzed.

**Results: ** All vaccine regimens appeared to be well tolerated. Pain and fatigue were the most frequently reported solicited events. The shortened regimens (Groups 2 and 3) elicited equivalent antibody titers against autologous Clade C Env at peak immunity to the Imbokodo‐like regimen (41,007 and 49,243 GMT vs. 44,590 GMT, respectively), with this peak occurring earlier in the shortened regimens. Antibody responses remained elevated (>5000 GMT) in Groups 2 and 3 at week 52. ADCP, Env‐specific IgG3, tier 1A neutralizing activity and broad cellular immune responses were detected in all groups.

**Conclusions: **In this Phase 1 study, we demonstrate that Ad26.Mos.HIV combined with gp140 Env protein can elicit HIV‐specific immune responses in shortened, 24 week vaccine schedules that appeared to be similar to responses elicited in a longer, 48 week vaccine schedule that is currently being evaluated in a clinical efficacy study. Further studies are required to test the protective efficacy of these shortened vaccine regimens.

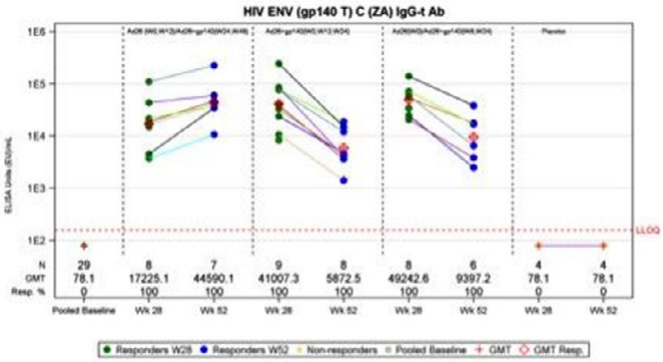

**Abstract TUAA0105‐Figrue 1. Antibody Titers Against Autologous Clade C Env in IPCAVD010.**

## TUAA0203

### Dominant HIV DNA populations present in different T‐cell subsets before stem cell transplantation persist in tissues early after transplantation with CCR5Δ32 stem cells

**A. Wensing^1^; K. Bosman^1^; A. Bruns^2,3,4^; P. Ellerbroek^2^; T. de Jong^1^; K. Tesselaar^4^; A. Stam^1^; M. Salgado^5^; G. Hutter^6^; L. Brosens^7^; M. Kwon^8^; J. Diez Martin^8^; J. Boelens^4,9^; J. Martinez‐Picado^5,10^; J. Kuball^3,4^; M. Nijhuis^1^ and IciStem Consortium**

^1^University Medical Center Utrecht, Translational Virology, Department of Medical Microbiology, Utrecht, Netherlands, ^2^University Medical Center Utrecht, Department of Internal Medicine and Infectious Diseases, Utrecht, Netherlands, ^3^University Medical Center Utrecht, Department of Hematology, Utrecht, Netherlands, ^4^University Medical Center Utrecht, Laboratory of Translational Immunology, Utrecht, Netherlands, ^5^AIDS Research Institute IrsiCaixa, Badalona, Spain, ^6^Cellex, Dresden, Germany, ^7^University Medical Center Utrecht, Department of Pathology, Utrecht, Netherlands, ^8^Hospital General Universitario Gregorio Maranon, Madrid, Spain, ^9^University Medical Center Utrecht, Blood and Marrow Transplantation Program, Department of Pediatrics, Utrecht, Netherlands, ^10^ICREA, Badalona, Spain

**Background:** Cure of HIV infection was observed in the Berlin patient following stem cell transplantation (SCT) with homozygous CCR5Δ32 donor cells. In contrast, in the Boston patients, transplanted with cells from regular CCR5WT donors, HIV rebound occurred after treatment interruption despite loss of detectable HIV‐DNA in PBMCs. It is unknown which reservoir fueled HIV rebound.

**Methods:** IciStem is an International collaboration to guide and investigate the potential for HIV cure in stem cell transplantation. In IciStem patient #5 SCT was performed using homozygous CCR5Δ32 cord blood combined with a third party donor. Before SCT we performed: (1) Phenotypic and genotypic coreceptor tropism analysis; (2) HIV reservoir quantification using ddPCR and viral characterization using deep‐sequencing of PBMCs, CD4^+^‐T‐cell subsets (Tn, Tcm, Ttm, Teff) and bonemarrow; (3) Single copy assay (SCA) on plasma. Post‐SCT viral dynamics were analyzed using ddPCR and SCA. The post‐mortem viral reservoir was quantified using ddPCR and characterized using deep‐sequencing.

**Results: ** Patient #5 was on effective cART for five years harboring subtype B CCR5‐tropic HIV‐1 (FPR: 68.8% to 96.2%). Before SCT, HIV‐RNA could be detected in plasma (15 copies/mL). HIV‐DNA LTR copies were detected in PBMCs (1967 copies/10^6), Tn cells (1270 copies/10^6), all memory T‐cells (Tcm, Ttm and Teff, 3074, 5564 and 6924 copies/10^6) and bonemarrow (1130 copies/10^6). Deep‐sequencing revealed that two viral variants dominate all T‐cell populations and bonemarrow (variant 1: FPR 87.2%; variant 2: 89.7%). Four weeks post‐SCT, complete donor chimerism was observed in PBMC, HIV‐DNA diminished to undetectable levels (<1 copies/10^6) and no HIV‐RNA could be detected in plasma. Ten weeks post‐SCT patient #5 deceased. Post‐mortem analysis revealed presence of HIV‐DNA LTR copies in ileum (549 copies/10^6), liver (54 copies/10^6), spleen (44 copies/10^6) and lung (62 copies/10^6), whereas no HIV‐DNA LTR copies could be detected in PBMCs (<7 copies/10^6). HIV‐sequences obtained from ileum and lung revealed the dominance of sequence variant two in both tissues.

**Conclusions: **In the neutropenic phase early post‐SCT, HIV‐DNA could no longer be detected in PBMCs. In contrast, dominant HIV‐DNA populations as present in different T‐cell subsets before SCT persisted in tissues indicating that tissue reservoirs may play an important role as long‐standing viral reservoirs.

## TUAA0204

### Rapid rebound of a highly replication competent preexisting CXCR4‐tropic HIV variant after allogeneic stem cell transplantation with CCR5Δ32 stem cells

**J. Verheyen^1,2^; A. Thielen^2^; N. Lübke^3^; M. Dirks^1^; M. Widera^1^; U. Dittmer^1^; L. Kordales^4^; M. Däumer^2^; D. de Jong^5^; A. Wensing^5^; R. Kaiser^6^; M. Nijhuis^5^ and S. Esser^7^**

^1^University of Duisburg‐Essen, University Hospital, Institute of Virology, Essen, Germany, ^2^Institute of Immunology and Genetics, Kaiserslautern, Germany, ^3^Heinrich‐Heine‐University, University Hospital, Institute of Virology, Düsseldorf, Germany, ^4^University Hospital, University of Duisburg‐Essen, Department of Bone Marrow Transplantation, Essen, Germany, ^5^University Medical Center Utrecht, Medical Microbiology, Utrecht, Netherlands, ^6^University of Cologne, Institute of Virology, Cologne, Germany, ^7^University Hospital, University of Duisburg‐Essen, Clinic for Dermatology, Essen, Germany

**Background:** To date, the case of the Berlin patient provides the only evidence of an intervention that has been able to cure HIV infection. The procedure involved an allogeneic stem cell transplantation (SCT) with donor cells lacking the CCR5 coreceptor (CCR5Δ32). Interestingly, in the Berlin patient no viral rebound was observed despite the fact that cART was stopped at the day of transplantation. In a similar setting in the Essen patient, cART was stopped before initiation of myeloablative therapy and a rapid viral rebound was observed after SCT. To fully understand the underlying mechanism of viral breakthrough in the Essen patient we retrospectively analyzed the genotypic and phenotypic characteristics of the viral population.

**Methods:** RNA was isolated from plasma and total DNA was isolated from PBMCs at different time points before (‐287d: RNA, ‐103d: RNA/DNA, ‐18d: DNA) and after (+20d: RNA, +373d: RNA/DNA) SCT. HIV coreceptor tropism was genotypically assessed (geno2pheno) after deep‐sequence analysis of the viral envelope (gp120‐V3). The observed gp120‐V3 sequences were cloned in our shuttle vector pHXB2‐Δgp120‐V3 and chimeric viruses were tested for replication capacity and coreceptor usage in primary cells (PBMCs).

**Results: ** Viral breakthrough was observed three weeks after SCT. Every single viral RNA sequence detected with deep‐sequence analysis at time of breakthrough was predicted to be CXCR4‐tropic (FPR: 0.2% to 0.7%). These sequences are genetically distinct from the pre‐SCT viral variants predicted to be CCR5‐tropic (FRP: 8.5% to 10.5%). Interestingly, the most dominant viral variant rebounding after SCT (FPR 0.4%) could already be detected as a minority variant in the proviral DNA 103 days before transplantation. This dominant variant, once cloned in our HIV shuttle vector, demonstrated a high replication capacity in primary cells and is completely dependent on the alternative CXCR4 coreceptor for replication.

**Conclusions: **In this study we demonstrate that the rapid rebound after SCT was related to a highly replicative CXCR4‐tropic HIV variant, which was already present prior to SCT. These data indicate that in‐depth HIV coreceptor analysis is essential for future CCR5‐based stem cell transplantation and gene therapy studies.

## TUAA0205

### Modular gene therapy vectors for gene therapy cure in resting immune cells

**A. Wong^1^; A. Aggarwal^1^; O. Atthi^1^; B. Hao^1^; H. Macrae^1^; M. Churchill^2^; A. Kelleher^1^ and S. Turville^1^**

^1^University of New South Wales, Kirby Institute, Sydney, Australia, ^2^RMIT University, School of Health and Biomedical Sciences, Melbourne, Australia

**Background:** Conventional gene therapy vectors warrant extensive cellular activation of the target population (defined as resting CD4 T cells, and macrophages) to increase gene delivery outcomes. However, cellular departure from the resting state lowers stemness and therefore long‐term therapeutic potential. There are two barriers to the genetic modification of target cells: particle delivery/fusogenicity, and ability to perform reverse transcription/integration. We designed a platform where vectors are customised to overcome these limitations.

**Methods:** To increase lentiviral vector fusion into CD4 T cells, over 1000 envelopes were surveyed, yielding a shortlist of one dozen candidate pseudotypes. These pseudotypes were previously characterised by the HIV Affinofile assay and distinguished by an ability to attain cellular entry despite low CD4 levels. For the enhancement of gene delivery, over 200 Vpx variants were surveyed, creating a shortlist of 37 candidate variants. These variants were validated by firstly determining their capacity to enhance HIV NL43 infection, before further measurement of gene delivery enhancement using lentiviral vectors.

**Results: ** From the many prospective pseudotypes, one lead candidate was identified. This pseudotype consistently enabled cellular entry in greater than 95% of untouched resting CD4 T cells. Six lead Vpx variants were identified that enhanced gene transfer up to 20‐fold and 10‐fold in macrophages and T cells, respectively. Combining both approaches resulted in an excess of 95% and 45% gene delivery in macrophages and T cells, respectively, accomplished using low MOIs (0.04). Whilst a majority of Vpx variants still enhanced gene delivery for greater than two weeks, we identified variants that possessed contracted enhancement durations in T cells (<2 weeks). This would alleviate cellular vulnerabilities to HIV upon reinfusion into hosts.

**Conclusions: **We have designed a lentiviral vector platform that targets resting cell types by leveraging the fusogenic potential of the lead pseudotype and enhancing potential of Vpx. We achieved gene delivery into a challenging cell type using limited inocula, and benchmarked at levels conducive to clinical applications.

## TUAB0101

### Comparative effectiveness of first‐line antiretroviral therapy regimens: results from a large real‐world cohort in Brazil after the implementation of Dolutegravir

**M.V. Meireles^1,2^; A.R. Pascom^1^; F. Perini^1^; F. Rick^1^ and A. Benzaken^1^**

^1^Ministry of Health of Brazil, Department of STI, AIDS and Viral Hepatitis, Brasilia, Brazil, ^2^University of Brasilia, Faculty of Medicine, Brasilia, Brazil

**Background:** In early 2017, the Ministry of Health of Brazil (MoH) released new antiretroviral treatment (ART) guidelines, which set Lamivudine+Tenofovir+Dolutegravir as the preferred first‐line regimen for HIV treatment. In this study, we used real‐world programmatic data from Brazil aiming to describe the observed effectiveness of different regimens in the initial response to ART, using the six‐month viral load (VL) count.

**Methods:** Programmatic data from two information systems from the MoH were used; they gather data on every VL and CD4 counts performed within the country's public health system, and on every ART dispensation. Patients aged 15 and over, who started ART from January 2014 to June 2017 and had a six‐month VL (180 ± 90 days after treatment initiation) were included. The outcome was failure to achieve initial virologic suppression (VS), defined as presenting the six‐month VL above 50 copies/mL. Univariable and multivariable analyses were performed, with unconditional logistic regression models assessing the likelihood of the outcome according to the initial ART regimen, controlling for adherence level, sex, age and CD4 and VL at treatment initiation. Adherence level was calculated using pharmacy refill data.

**Results: ** Of the 103,240 patients included in the analysis, 67.6% were male; median values of age, baseline CD4, baseline VL and adherence were 34 years old, 394 cells/mm^3^, 38,057 copies/mL and 96.2%, respectively. Overall, 76.9% achieved a VL<50 copies/mL. The most common regimens were 3TC+TDF+EFZ (74.0%), 3TC+TDF+DTG (7.2%), 3TC+AZT+LPV/r (4.9%), 3TC+TDF+ATV/r (4.6%), 3TC+AZT+EFZ (3.5%) and 3TC+TDF+LPV/r (2.0%). VS ranged from 63.7% with 3TC+TDF+LPV/r to 85.2% with 3TC+TDF+DTG. In the multivariable analysis, with 3TC+TDF+DTG as the reference, aOR (95% CI) of failing to achieve VS were 1.42 (1.32 to 1.52) for 3TC+TDF+EFZ, 1.51 (1.35 to 1.68) for 3TC+AZT+EFZ, 2.11 (1.91 to 2.32) for 3TC+TDF+ATV/r, 2.41 (2.18 to 2.66) for 3TC+AZT+LPV/r and 2.62 (2.32 to 2.95) for 3TC+TDF+LPV/r.

**Conclusions: **The observed effectiveness of 3TC+TDF+DTG was markedly superior after controlling for possible confounders, with all other regimens showing 42% to 162% higher odds of not achieving initial virologic suppression. Our results support the decision made by the MoH to switch its recommendations for preferred first‐line ART from Efavirenz to Dolutegravir‐containing regimens.

**Abstract TUAB0101‐Table 1. Baseline characteristics and results of the multivariable logistic regression model for VL>50 copies/mL (n = 103,240)**

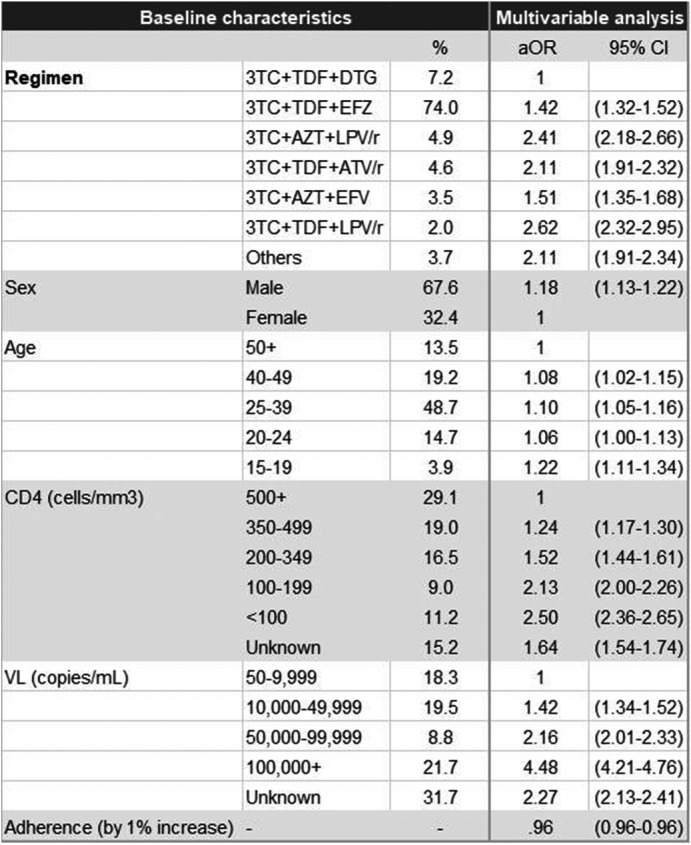

## TUAB0102

### Simplification to dolutegravir monotherapy is non‐inferior compared to continuation of combination antiretroviral therapy in patients who initiated combination antiretroviral therapy during primary HIV infection: a randomized, controlled, non‐inferiority trial

**D.L. Braun^1,2^; T. Turk^1^; B. Hampel^1,2^; C. Grube^1^; P. Schreiber^1^; M. Greiner^1^; D. Steffens^1^; F. Tschumi^1^; C. Bayard de‐Torronté^1^; C. Depmeier^1^; K. Metzner^1,2^; B. Bertisch^3^; J. Böni^2^; R. Kouyos^1,2^; H. Günthard^1,2^ and for the Zurich Primary HIV Infection Study**

^1^University Hospital Zurich, Division of Infectious Diseases and Hospital Epidemiology, Zurich, Switzerland, ^2^Institute of Medical Virology, University of Zurich, Zurich, Switzerland, ^3^Checkpoint Zurich, Zurich, Switzerland

**Background:** Patients who started combination antiretroviral therapy (cART) during primary HIV‐1 infection (PHI) show a smaller HIV‐1 reservoir size compared to patients who started cART during chronic infection. Thus, we hypothesized that a smaller HIV‐1 reservoir size translates in sustained virological suppression after simplification of cART to dolutegravir monotherapy.

**Methods:** In this randomized, open‐label, non‐inferiority trial, we recruited patients >18 years with documented PHI who started cART <180 days after estimated date of infection (EDI) and were fully suppressed for >48 weeks. Exclusion criteria were previous virological failure or treatment interruption and major resistance associated mutations (RAM) to integrase inhibitors. We randomly assigned patients 2:1 to monotherapy with dolutegravir 50 mg once daily or to continuation of cART. Primary endpoint was virological response, defined as HIV‐1 RNA <50 copies/mL plasma at week 48, in the per‐protocol‐population, with a non‐inferiority margin of 10% (NCT02551523).

**Results: ** Between November 2015 to March 2017, we randomly assigned 101 patients (68 to dolutegravir monotherapy, 33 to continuation of cART). At week 48 in the per‐protocol‐population, 67/67 (100%) had virological response in the dolutegravir monotherapy group versus 31/31 (100%) in the cART group (difference 0%, 95%‐CI (−1, 0.047)), showing non‐inferiority at the prespecified level (Figure 1). In the intention‐to‐treat population, 1 patient in the dolutegravir monotherapy group experienced viral failure at week 36 (viral load 382 cp/mL) and two patients in the cART group left the study before week 48 because they moved abroad. The patient who experienced viral failure was found to be chronically infected at the start of first cART and therefore violated entry criteria. Resistance test at time of viral failure revealed no RAMs and he was re‐suppressed on cART. Overall, 14 severe adverse events occurred (dolutegravir monotherapy 10 (15%); cART 4 (12%)), none related to study‐drugs.

**Conclusions: **In our randomized simplification trial, monotherapy with once daily dolutegravir was effective, safe, and non‐inferior to cART in patients with a documented PHI who initiated cART <180 days after EDI and were virologically suppressed for at least 48 weeks. Our results suggest that future simplification studies should use a stratification according to time of infection at start of first cART.

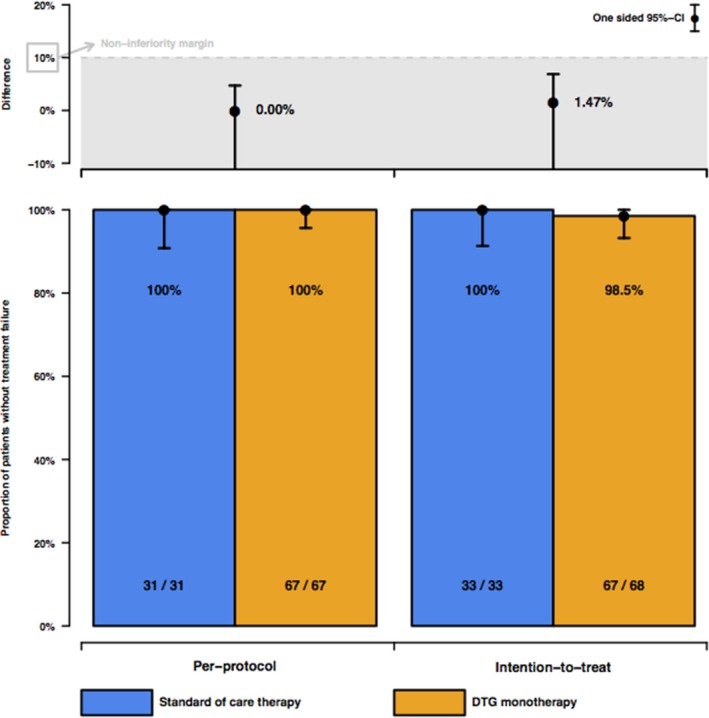

**Abstract TUAB0102‐Figure 1. HIV‐1 RNA <50 copies per mL plasma at week 48 for the per‐protocol and intention‐to‐treat populations. Error bars are 95% CI.**

## TUAB0103

### Dolutegravir monotherapy versus dolutegravir/abacavir/lamivudine for HIV‐1‐infected virologically suppressed patients: results from the randomized non‐inferiority MONCAY trial

**L. Hocqueloux^1^; C. Allavena^2^; T. Prazuck^1^; L. Bernard^3^; S. Sunder^4^; J.‐L. Esnault^5^; D. Rey^6^; G. Le Moal^7^; M. Roncato‐Saberan^8^; M. André^9^; E. Billaud^2^; V. Avettand‐Fènoël^10^; A. Valéry^11^; F. Raffi^2^; J.‐J. Parienti^12^ and MONCAY Study Group**

^1^CHR d'Orléans ‐ La Source, Infectious and Tropical Diseases, Orléans, France, ^2^CHU de Nantes, Infectious Diseases, Nantes, France, ^3^CHU de Tours, Infectious Diseases, Tours, France, ^4^CHG, Infectious Diseases, Niort, France, ^5^CHD, Infectious Diseases, La Roche sur Yon, France, ^6^CHU de Strasbourg, Infectious Diseases, Strasbourg, France, ^7^CHU de Poitiers, Infectious Diseases, Poitiers, France, ^8^CHG, Infectious Diseases, La Rochelle, France, ^9^CHU de Nancy, Infectious Diseases, Nancy, France, ^10^CHU Necker ‐ Enfants Malades, APHP, Virology, Paris, France, ^11^CHR d'Orléans ‐ La Source, Biostatistics, Orléans, France, ^12^CHU de Caen, Clinical Research and Biostatistics, Caen, France

**Background:** We investigated whether dolutegravir alone was able to maintain virological suppression in HIV‐1‐infected patients on a successful dolutegravir‐based standardized triple‐therapy.

**Methods:** MONCAY was a 48‐week multicentric, randomized, open‐label, 12% non‐inferiority margin study. Inclusion criteria were: age ≥18 years, CD4 nadir >100/μL, no previous AIDS event, plasma HIV‐RNA (pVL) <50 copies/mL for ≥12 months, stable regimen with once daily dolutegravir/abacavir/lamivudine (DTG/ABC/3TC) and no failure or resistance to any integrase inhibitor (INI). Patients were 1:1 randomized to continue DTG/ABC/3TC or to simplify to DTG monotherapy. The primary endpoint was the proportion of patients with pVL <50 copies/mL at week (W) 24 in intention‐to‐treat (ITT), missing or switch equals failure (M=F); modified ITT (mITT) excluding patients who had non‐inclusion criteria; Per‐protocol (PP) excluding from mITT patients with major protocol deviation. Virologic failure (VF) was defined as two consecutive pVL >50 copies/ml within two weeks apart.

**Results: ** Seventy‐eight patients were assigned to DTG and 80 to continue DTG/ABC/3TC. Of these 158 patients, 3 had non‐inclusion criteria and six had major protocol deviation in the DTG arm; two had non‐inclusion criteria and 1 had major protocol deviation in the DTG/ABC/3TC arm. By W24, two patients in DTG group experienced VF (both at W24) without resistance to the INI class; 1 patient stopped DTG/ABC/3TC due to adverse event (at W4). In ITT (n = 158), the success rate was 73/78 (93.6%) in the DTG arm and 77/80 (96.3%) in the DTG/ABC/3TC arm; difference 3.9%, 95% CI: −5.0 to 10.8. This figure was 1.4%; 95% CI: −4.5 to 8.1 in mITT (n = 153) and 1.6%; 95% CI: −4.5 to 8.8 in PP (n = 146). During subsequent follow‐up, three additional patients in the DTG arm experienced VF (2 at W36 and 1 at W48) with emerging resistance mutations to INI in two cases, whereas none occurred in the DTG/ABC/3TC group (difference 6.5%, 95% CI: −1.8 to 15.6). The DSMB recommended to re‐intensify the DTG arm with standardized triple‐therapy.

**Conclusions: **Although non‐inferior to DTG/ABC/3TC at W24, DTG monotherapy was not a valid option to maintain virological suppression overtime in HIV‐1‐infected patients on a successful DTG/ABC/3TC triple‐therapy and favoured emergence of INI resistance.

## TUAB0104

### A phase 3b, open‐label, pilot study to evaluate switching to elvitegravir/cobicistat/emtricitabine/tenofovir alafenamide (E/C/F/TAF) in virologically‐suppressed HIV‐1 infected adult subjects harboring the NRTI resistance mutation M184V and/or M184I (GS‐US‐292‐1824)

**I. Perez Valero^1^; J.M. Llibre^2^; A. Lazzarin^3^; G. Di Perri^4^; F. Pulido^5^; J.‐M. Molina^6^; S. Esser^7^; I. McNicholl^8^; R.‐P. Lorgeoux^9^; N. Margot^8^; Y. Shao^8^; D. Piontkowsky^8^; M. Das^8^ and R. Haubrich^8^**

^1^Unidad VIH ‐ Hospital Universitario La Paz, Madrid, Spain, ^2^Fundación Lucha contra el SIDA, Barcelona, Spain, ^3^Fondazione IRCCS San Raffaele del Monte Tabor, Milan, Italy, ^4^Dipartimento di Malattie Infettive e Tropicali, Turin, Italy, ^5^Unidad VIH, Hospital Universitario 12 de Octubre, imas12, UCM, Madrid, Spain, ^6^Department of Infectious Diseases, Saint‐Louis Hospital and University of Paris, Paris, France, ^7^Universitätsklinikum Essen, Essen, Germany, ^8^Gilead Sciences, Inc, Foster City, United States, ^9^Gilead Sciences, Inc, Montreal, Canada

**Background:** Treatment with once‐daily E/C/F/TAF in HIV‐1‐infected therapy‐naïve patients was shown to be effective and safe through 144 weeks in two randomized, double‐blinded trials, which excluded participants whose HIV‐1 harbored the M184V and/or M184I mutation.

**Methods:** This ongoing, prospective open‐label, single arm, multicenter, 48‐week trial is evaluating the efficacy and safety of switching suppressed participants to E/C/F/TAF from a stable regimen (≥6 months) of a third agent plus either F/tenofovir disoproxil fumarate or abacavir/lamivudine. Participants had a historical genotype report showing M184V and/or M184I and no evidence of previous virologic failure (VF) or resistance to boosted PIs or INSTIs. At screening, HIV‐1 RNA <50 copies/mL was required as well as absence of additional NRTI or PI resistance mutations based on sequencing of integrated HIV DNA (GenoSure Archive, Monogram Biosciences). The primary objective is to evaluate the efficacy of switching to E/C/F/TAF in maintaining HIV‐1 RNA <50 copies/mL at Week 12 using pure virologic response (PVR). Participants with discontinuation or missing values were considered responders if they never had HIV‐1 RNA ≥50 copies/mL at two consecutive visits and the last HIV‐1 RNA was <50 copies/mL. This report presents the Week 24 data.

**Results: ** Thirty‐seven participants were enrolled and switched to E/C/F/TAF. Mean age was 50 years (range 22 to 76), 73% White, 19% Black, 22% women, median CD4 count 724 cells/μL and 100% HIV RNA <50 copies/mL at baseline. Through Week 24, all 37 participants (100%) had HIV‐1 RNA <50 copies/mL based on PVR (Table 1). Three participants who discontinued prior to Week 24 with last recorded HIV‐1 RNA <50 copies/mL were not considered VF. Four serious adverse events occurred (none were study drug‐related): 1 each of squamous cell carcinoma, acute kidney injury (with poorly controlled hypertension and diabetes), transient proteinuria (resolved on study drug) and pulmonary embolism. Twenty‐two percent (8/37) of participants experienced a study drug‐related AE (grade 1 or 2); one participant discontinued due to grade 2 muscle spasms.

**Conclusions: **E/C/F/TAF offers an effective, well tolerated switch option for patients with pre‐existing M184V and/or M184I mutations. These data on continued virologic suppression despite resistance are encouraging though longer term data are needed.

**Abstract TUAB0104‐Table 1.**

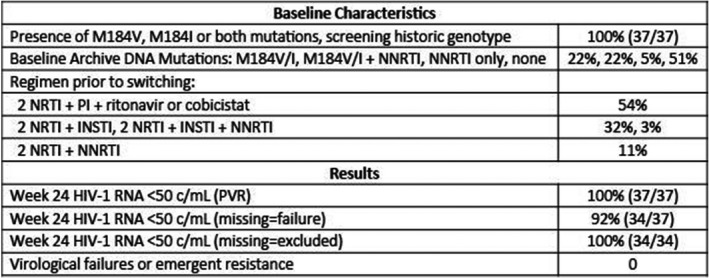

## TUAB0201

### Durability and effectiveness of isoniazid preventive therapy in Lesotho, southern Africa

**E. Mugomeri^1^; D. Olivier^2^ and W.M.J. van den Heever^2^**

^1^National University of Lesotho, Department of Pharmacy, Maseru, Lesotho, ^2^Central University of Technology, Department of Health Sciences, Bloemfontein, South Africa

**Background:** The southern African country of Lesotho, which has the highest tuberculosis (TB) incidences in the world, is facing a catastrophic syndemic of HIV and tuberculosis. In 2011, the government of Lesotho launched isoniazid preventive therapy (IPT) as a once‐off intervention with no follow‐up booster doses, to reduce the occurrence of TB in people living with HIV (PLHIV). However, the effectiveness and durability of this intervention remains obscure in this setting. This study evaluated the effectiveness of IPT and the durability of its protection in Lesotho.

**Methods:** The study was based on 2955 records which met the inclusion criteria out of 4122 HIV‐positive medical records randomly sampled from eight health institutions in six districts of Lesotho. Univariate Kaplan‐Meier function, Wilcoxon's log‐rank test and Cox regression analyses were used to select factors into the model. Cox's proportional hazards regression analysis was performed, with data formatted as discrete‐time survival data with interval date as the time variable and the occurrence of TB as the ‘failure’ outcome.

**Results: ** The overall TB incidence rate was 2.0 per 100 person‐years in 12 208 person‐years. Thirty‐nine (15.9%, *n = *246) patients developed TB after IPT. TB incidences per 100 person‐years by timing of IPT were as follows: (a) IPT before ART (1.7); (b) IPT after ART (1.8); (c) no IPT (2.6); (d) IPT within one year of ART commencement (1.3) and (e) IPT three to five years after ART initiation (2.3). IPT effectiveness rapidly deteriorated after four years in patients given IPT within one year of ART commencement (Figure 1). Gender, baseline WHO clinical stage, district category and time to IPT relative to ART commencement emerged as significant predictors of TB occurrence. Increasing time to IPT by one six‐month interval increased the risk of contracting TB by between 6% and 59%, depending on the cohort.

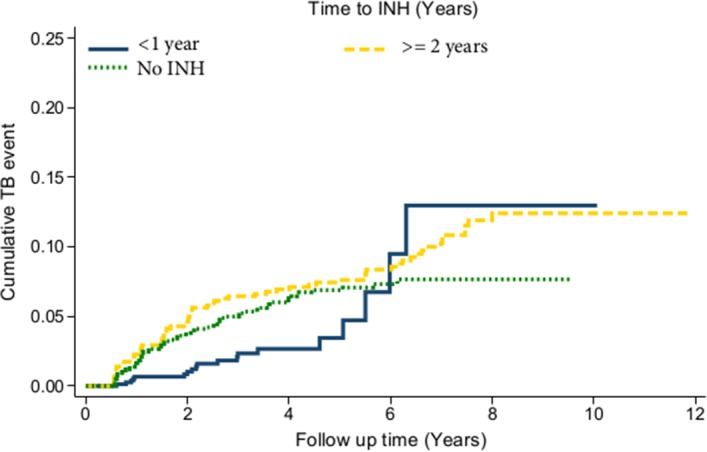

**Abstract TUAB0201‐Figure 1.**

**Conclusions: **While IPT significantly reduces the risk of TB, delayed IPT after ART commencement significantly affects the effectiveness of this intervention. The apparent loss of protection four years after IPT indicates the need for booster doses of IPT in this population. Patient characteristics significantly associated with higher risk of TB are important for policy making.

## TUAB0202

### Drug susceptibility testing, HIV‐coinfection and outcomes in patients treated for tuberculosis in low‐ and middle‐income settings

**K. Zürcher^1^; M. Ballif^1^; L. Fenner^1^; S. Borrell^2,3^; P.M. Keller^4,5^; J. Gnokoro^6^; M. Yotebieng^7^; L. Diero^8,9^; E.J. Carter^8,9^; N. Rockwood^10,11^; R.J. Wilkinson^10,11,12^; H. Cox^13^; N. Ezati^14,15^; A.G. Abimiku^14,15^; J.L. Collantes^16^; A. Avihingsanon^17,18^; K. Kawkitinarong^18^; M. Reinhard^2,3^; R. Hömke^4,5^; R. Huebner^19^; E.C. Böttger^4,5^; S. Gagneux^2,3^; M. Egger^1,20^ and on behalf of the International Epidemiology Databases to Evaluate AIDS (IeDEA)**

^1^Universty of Bern, Institute of Social and Preventive Medicine ^ISPM^, Bern, Switzerland, ^2^Swiss Tropical and Public Health Institute, Basel, Switzerland, ^3^University of Basel, Basel, Switzerland, ^4^University of Zurich, Institute of Medical Microbiology, Zurich, Switzerland, ^5^Swiss National Center for Mycobacteria, Zurich, Switzerland, ^6^Centre de Prise en Charge de Recherche et de Formation, Yopougon, Cote D'Ivoire, ^7^Ohio State University, College of Public Health, Columbus, United States, ^8^Moi University School of Medicine, Department of Medicine, Eldoret, Kenya, ^9^Moi Teaching and Referral Hospital, Department of Medicine, Eldoret, Kenya, ^10^University of Cape Town, Welcome Centre for Infectious Diseases Research in Africa, Cape Town, South Africa, ^11^Imperial College, London, United Kingdom, ^12^Francis Crick Institute, London, United Kingdom, ^13^University of Cape Town, Division of Medical Microbiology and the Institute for Infectious Disease and Molecular Medicine, Cape Town, South Africa, ^14^Institute of Human Virology, Abuja, Nigeria, ^15^National Tuberculosis and Leprosy Training Center, Saye, Zaria, Nigeria, ^16^Universidad Peruana Cayetano Heredia, Instituto de Medicina Tropical Alexander von Humboldt, Lima, Peru, ^17^HIV‐NAT/Thai Red Cross AIDS Research Centre, Bangkok, Thailand, ^18^Chulalongkorn University, Department of Medicine, Bangkok, Thailand, ^19^National Institutes of Health, National Institutes of Allergy and Infectious Diseases, Bethesda, United States, ^20^University of Cape Town, Centre for Infectious Disease Epidemiology & Research, School of Public Health & Family Medicine, Cape Town, South Africa

**Background:** Drug resistance and HIV‐coinfection are major challenges for the global control of tuberculosis (TB).

**Methods:** We collected *Mycobacterium tuberculosis (Mtb)* isolates from adult TB patients in Côte d′Ivoire, Democratic Republic of the Congo, Kenya, Nigeria, South Africa, Peru, and Thailand, stratified by HIV status and TB drug resistance. Drug susceptibility testing (DST) was performed locally (Xpert MTB/RIF, line probe assay or culture) and at the Swiss National Center for Mycobacteria (MGIT liquid culture). We categorized drug regimens into adequate treatment, under‐treatment and over‐treatment, based on WHO and local guidelines. We used multivariate logistic regression adjusted for age, sex, sputum microscopy, HIV status, and treatment adequacy, accounting for clustering at site‐level, to examine mortality during treatment according to DST results and treatment adequacy.

**Results: ** 634 TB patients were included; 272 (42.9%) were HIV‐positive, with a median CD4 cell count at TB treatment start of 192 cells/μL (IQR 78 to 369 cells/μL). 175 (64.3%) of HIV‐positive patients were on ART at the start of TB treatment or initiated ART within three months. Based on reference MGIT DST, 394 (62.2%) isolates were pan‐susceptible, 45 (7.1%) mono‐resistant, 163 (25.7%) multidrug‐resistant (MDR‐TB), and 30 (4.7%) pre‐extensively or extensively drug‐resistant (pre‐XDR/XDR‐TB). In 126 (19.9%) patients, local and reference DST results were discordant. For any drug resistance, the sensitivity of local DST was 84% (95% CI 80% to 88%); specificity was 89% (95% CI 84% to 92%).Treatment was inadequate (under‐/over‐treatment) in 25/126 (19.8%) patients with discordant DST results, and 16/508 (3.1%) with concordant DST (*p* < 0.001). Mortality was 13.6% (24/176) if DST results were concordant, but 26.6% (17/64) if DST results were discordant (*p* = 0.019). The corresponding risk ratio was 1.95 (95% CI 1.12 to 3.38), and the population attributable fraction 20.2%. In multivariate logistic regression, mortality was determined by TB drug resistance and adequacy of treatment, but not by HIV status, gender or sputum positivity.

**Abstract TUAB0202‐Table 1. Factors associated with mortality during treatment in patients diagnosed with tuberculosis complete case analysis**

**Table:** 

	No. of patients (n = 542)	No. of deaths (%)	Unadjusted OR (95% CI)	Adjusted OR (95% CI)
Sex				
Female	206	19 (9.2)	1	1
Male	336	45 (13.4)	1.52 (0.86 to 2.68)	1.40 (0.75 to 2.59)
Age (per 1 year increase)	542	64 (11.8)	1.03 (1.01 to 1.05)	1.04 (1.02 to 1.06)
HIV status				
Negative	326	41 (12.6)	1	1
Positive	216	23 (10.7)	0.82 (0.48 to 1.42)	1.15 (0.50 to 2.67)
Treatment adequacy				
Pan‐susceptible adequate	319	19 (6.0)	1	1
Pan‐susceptible inadequate	24	4 (16.7)	2.36 (0.64 to 8.65)	2.75 (0.46 to 16.47)
Any resistance adequate	180	36 (20.0)	3.93 (2.18 to 7.10)	4.82 (2.40 to 9.64)
Any resistance inadequate	19	5 (26.3)	7.26 (2.28 to 21.23)	7.40 (2.57 to 21.33)

**Conclusions: **Inaccurate DST testing leading to inappropriate treatment, but not HIV infection, contributed to mortality during treatment of drug‐resistant TB. Increasing capacity for DST and adequate drug‐resistant TB treatment is a priority in low‐ and middle‐income countries with high TB burden.

## TUAB0203

### Xpert MTB/Rif Ultra for earlier diagnosis of TB meningitis in HIV‐positive adults

**F. Cresswell^1,2^; N. Bahr^3^; A. Bangdiwala^4^; A. Akampuria^5^; K. Ssemambulidde^5^; J. Rhein^4,5^; D. Williams^4,5^; R. Kwizera^6^; E. Nuwagira^7^; P. Orikiriza^7^; C. Muzoora^7^; D. Meya^1,6^; D. Boulware^4^ and A. Elliott^8^**

^1^Infectious Diseases Institute, Clinical Research, Kampala, Uganda, ^2^London School of Hygiene and Tropical Medicine, Infectious and Tropical Diseases, London, United Kingdom, ^3^University of Kansas, Kansas City, United States, ^4^University of Minnesota, Minneapolis, United States, ^5^Infectious Diseases Institute, Kampala, Uganda, ^6^Makerere University, Kampala, Uganda, ^7^Mbarara University of Science and Techology, Mbarara, Uganda, ^8^London School of Hygeine and Tropical Medicine, London, United Kingdom

**Background:** TB meningitis (TBM) mortality is 40% to 60% in HIV‐positive individuals, in part due to diagnostic delay. Earlier diagnosis and initiation of treatment are needed to improve outcomes. The re‐engineered Xpert Ultra (Ultra) has an eightfold lower limit of detection than Xpert (Xpert) and requires evaluation in clinical settings.

**Methods:** We obtained samples of cerebrospinal fluid (CSF) from HIV‐positive adults presenting with suspected meningitis to Mbarara (since Feb 2015) and Mulago Hospitals (since Dec 2016), in Uganda. CSF was tested for cryptococcal antigen, then if negative, centrifuged and tested with Xpert, Ultra and culture. The performance of Ultra was measured against:(1)Composite reference standard of any positive CSF test(2)Uniform case definition of “probable” or “definite” TBM.

**Results: ** CSF was collected from 406 patients. After exclusion of cryptococcal meningitis (57%, 231/406), CSF from 206 patients was tested for TBM, of which 37 (18.0%) patients had microbiologically confirmed TBM (33 Ultra, 16 Xpert and 17 culture positive, Figure 1). 47 met the criteria for “probable” or “definite” TBM by the uniform case definition.

Against composite reference standard the sensitivity of Ultra was 89% (33/37), versus 43% (16/37) for Xpert and 46% (17/37) for culture. Against uniform case definition sensitivity of Ultra was 70% (33/47), versus 34% (16/47) for Xpert and 36% (17/47) for culture.

Specificity of Ultra was 100% and negative predictive value was 98% (95% CI 94% to 99%) against the composite reference standard and was 91% against then uniform case definition.

13 were positive only by Ultra, of which we tested 10/13 samples with a multiplex meningoencephalitis PCR assay and did not identify other potential aetiology. In these HIV‐positive adults with meningitis we believe these are true positive results. Four patients were negative by Ultra but positive on culture.

**Conclusions: **Ultra detected significantly more cases than either Xpert or culture in this HIV‐positive population. A diagnostic test with ˜90% sensitivity and a 90 minute turnaround time holds the potential to improve early diagnosis. Whilst 98% NPV in microbiologically proven TBM is an vast improvement, the NPV of 91% against uniform case definition means that Ultra cannot be used as a stand‐alone rule‐out test for TBM.

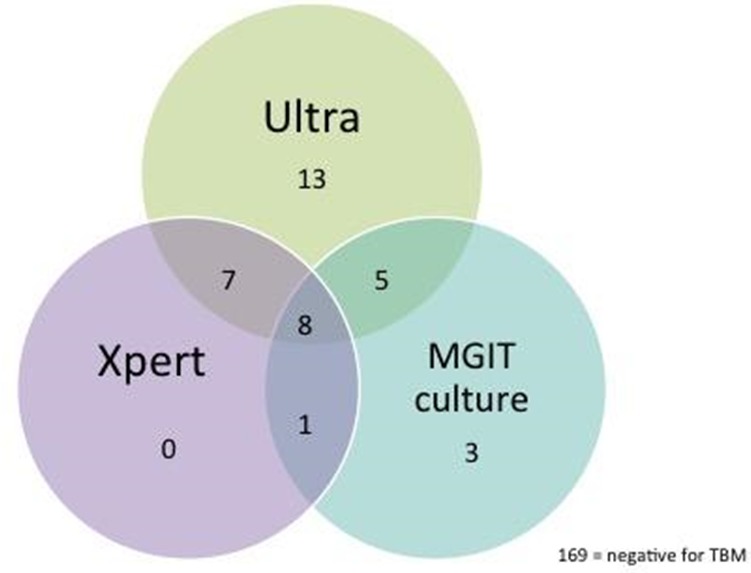

**Abstract TUAB0203‐Figure 1. Venn diagram illustrating the method of diagnosis of the 36 microbiologically confirmed cases of TBM in HIV‐positive Ugandan adults.**

## TUAB0204

### Risk factors of recurrent TB disease in a setting of high HIV prevalence

**S. Hermans^1,2^; N. Zinyakatira^3,4^; J. Caldwell^5^; F. Cobelens^1,6^; A. Boulle^3,4^ and R. Wood^2,7^**

^1^Amsterdam Institute for Global Health and Development, Amsterdam, Netherlands, ^2^Desmond Tutu HIV Centre, Institute of Infectious Disease and Molecular Medicine, University of Cape Town, Cape Town, South Africa, ^3^School of Public Health and Family Medicine, University of Cape Town, Cape Town, South Africa, ^4^Western Cape Government Health, Cape Town, South Africa, ^5^City Health, Department of Health, Cape Town, South Africa, ^6^KNCV Tuberculosis Foundation, The Hague, Netherlands, ^7^Department of Medicine, University of Cape Town, Cape Town, South Africa

**Background:** The incidence of retreatment tuberculosis (TB) disease in high‐prevalence settings such as Cape Town is very high. We previously showed that risk of recurrence increases with every subsequent episode. It is unclear what mechanisms underlie this increasing risk over time. We evaluated the risk factors of recurrent TB disease after previous TB treatment completion by subsequent episode.

**Methods:** All recorded TB episodes from January 2003 to April 2016 in the City of Cape Town were included, and linked to individuals by deterministic linkage of personal identifiers. Among a cohort of individuals whose first episode was notified in Cape Town we calculated recurrence rates after previous treatment completion which were adjusted to population HIV‐ and non‐HIV associated mortality rates sourced from the THEMBISA HIV model of the Western Cape. We used multivariable Cox proportional hazards regression to estimate risk factors of recurrent TB disease per subsequent episode.

**Results: ** A total of 245,533 individuals experienced 21,297 episodes of recurrent disease. The rate of recurrent TB after previous treatment completion was 14.5 (95% CI 14.2 to 14.7) per 1000 person‐years of follow‐up and increased per subsequent episode: the HIV‐negative rate increased sevenfold from episode 2 to episode 5 (from 11.3 (95% CI 11.0 to 11.7)/1000 to 84.1 (95% CI 62.4 to 113.5)/1000 respectively), and the HIV‐positive rate increased sixfold (from 22.1 (95% CI 21.6 to 22.6)/1000 to 123.7 (95% CI 99.5 to 153.8)/1000). HIV infection was the strongest risk factor for recurrence, reducing per subsequent episode (Table 1). Other factors identified were male gender and older age. Antiretroviral treatment (ART) use was associated with a reduced rate of recurrence for a second episode but not for subsequent episodes. CD4 count was not associated with recurrence.

**Conclusions: **We identified very high TB recurrence rates after successful previous treatment in Cape Town, especially after multiple episodes. As expected, HIV was the strongest risk factor and ART had a protective effect. However, the importance of HIV as a risk factor declined over subsequent episodes, suggesting additional mechanisms underlying the escalating rate of TB recurrence. Further investigation into increased risks of reinfection or progression to disease through clinical, microbiological, immunological and socio‐economic factors is warranted.

Abstract TUAB0204‐Table 1. Adjusted hazard ratios (95% confidence intervals) per subsequent episode of TB recurrence. *Among HIV‐positives only (adjusted for age and gender)

**Table:** 

Episode	2	3	4	5
Gender				
Female	1	1	1	1
Male	1.35 (1.30 to 1.40)	1.15 (1.06 to 1.26)	0.99 (0.83 to 1.20)	0.66 (0.45 to 0.98)
Age				
Per 10 years′ increase	1.02 (1.01 to 1.04)	1.04 (1.00 to 1.07)	0.97 (0.89 to 1.05)	1.15 (0.95 to 1.39)
HIV status				
Negative	1	1	1	1
Positive	1.89 (1.82 to 1.96)	1.44 (1.31 to 1.57)	1.26 (1.04 to 1.52)	1.37 (0.92 to 2.04)
CD4 count				
<200/μL	1	1	1	1
≥200/μL	1.03 (0.95 to 1.12)	1.00 (0.85 to 1.18)	0.90 (0.65 to 1.25)	1.18 (0.60 to 2.31)
ART use at previous episode				
No	1	1	1	1
Yes	0.84 (0.74 to 0.95)	1.10 (0.85 to 1.18)	1.46 (1.04 to 2.04)	1.47 (0.74 to 2.90)

## TUAB0205

### Clinical outcomes with bedaquiline use when substituted for second‐line injectable agents in multidrug resistant tuberculosis: a retrospective cohort study

**Y. Zhao^1^; K. Manning^1^; A. Stewart^2^; T. Fox^1^; N. Tiffin^3^; A. Boulle^3^; V. Mudaly^4^; Y. Kock^4^; G. Meintjes^5^ and S. Wasserman^5^**

^1^University of Cape Town, Department of Medicine, Cape Town, South Africa, ^2^University of Cape Town, Clinical Research Centre, Cape Town, South Africa, ^3^University of Cape Town, School of Public Health and Family Medicine, Cape Town, South Africa, ^4^Provincial Government of the Western Cape, Department of Health, Cape Town, South Africa, ^5^University of Cape Town, Wellcome Centre for Infectious Diseases Research in Africa, Department of Medicine, Cape Town, South Africa

**Background:** Second‐line injectable drugs (SLIs), core agents in the treatment of multidrug resistant tuberculosis (MDR‐TB), are associated with substantial toxicity and treatment discontinuations. Bedaquiline is being widely used as a substitute in MDR‐TB regimens for patients unable to tolerate SLIs, but the efficacy and safety of this strategy is unknown.

**Methods:** We conducted a retrospective cohort study to evaluate outcomes at 12‐months for MDR‐TB patients who substituted bedaquiline for SLIs. We included consecutive adult MDR‐TB patients who had bedaquiline substitutions in the Western Cape Province of South Africa between May 2015 and May 2016, as well as MDR‐TB controls who did not receive bedaquiline, matched for location and time of treatment initiation. Data were extracted from the electronic TB register. The composite primary outcome measure was the proportion of patients with death, loss to follow up, or failure to achieve sustained culture conversion at 12 months of treatment.

**Results: ** Data from 330 patients with laboratory‐confirmed pulmonary MDR‐TB were analyzed; 162 with bedaquiline substitution and 168 controls. Baseline characteristics were similar between the groups, except for CD4 cell count which was lower in the bedaquiline group (Table 1). SLIs were stopped at a median of 54 days (interquartile range, IQR 25 ‐ 82), with a 44 day (IQR 29 ‐ 71) delay to starting bedaquiline. The primary outcome, ascertained in 200 individuals, occurred in 63

(55.3%) patients in the bedaquiline group versus 54 (62.8%) patients in the control group (odds ratio, 0.73; 95% confidence interval (CI), 0.41 to 1.23; *p* = 0.285). Rates of sustained culture conversion (48.6% vs. 47.8%), loss to follow up (10.5% vs. 12.5%), and death (6.8% vs. 6.6%) at 12 months were similar between the groups. There was a trend towards earlier sputum culture conversion in the bedaquiline group (hazard ratio, 1.33; 95% CI, 0.94 to 1.88; *p* = 0.104; Figure 1).

**Conclusions: **Substituting bedaquiline for SLIs in the treatment of MDR‐TB does not result in inferior outcomes at 12 months compared with patients who remain on SLIs, supporting the use of this strategy in MDR‐TB therapy. The substantial delay between interrupting SLIs and initiating bedaquiline needs to be addressed.

**Abstract TUAB0205‐Table 1. Baseline demographic and clinical characteristics**

**Table:** 

	Bedaquiline (n = 162)	Control (n = 168)
Age, years	42 (35 to 49)	35 (28 to 42)
Male sex	93 (57.4)	97 (58.1)
Weight, kg	54 (45.3 to 61.6)	No data
HIV positive	110 (67.9)	94 (74.0)
CD4 count, cells/mm3	92.5 (46 to 185)	222.5 (54 to 375)
HIV viral load lower than detectable limit	46 (63.0)	50 (72.5)
Previous TB (any)	88 (63.3)	95 (56.6)
Extra‐pulmonary TB	18 (11.4)	13 (7.8)
Sputum smear positivity	98 (60.5)	112 (66.7)

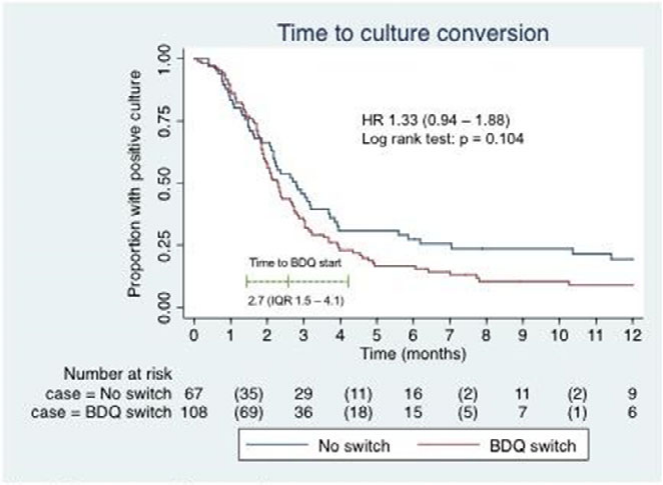

**Abstract TUAB0205. Figure 1. Time to sputum culture conversion.**

## TUAB0206

### Safety and efficacy of dolutegravir‐based ART in TB/HIV co‐infected adults at week 48

**K. Dooley^1^; R. Kaplan^2^; T. Mwelase^3^; B. Grinsztejn^4^; E. Ticona^5^; M. Lacerda^6^; O. Sued^7^; E. Belonosova^8^; M. Ait‐Khaled^9^; K. Angelis^10^; D. Brown^11^; R. Singh^12^; C. Talarico^13^; A. Tenorio^13^; M. Keegan^9^ and M. Aboud^9^**

^1^Johns Hopkins University School of Medicine, Baltimore, United States, ^2^Desmond Tutu HIV Foundation, Cape Town, South Africa, ^3^Clinical HIV Research Unit, Johannesburg, South Africa, ^4^4Instituto de Pesquisa Clínica Evandro Chagas FIOCRUZ, Rio de Janeiro, Brazil, ^5^Hospital Dos de Mayo, Lima, Peru, ^6^Fiocruz/Tropical Medicine Foundation Dr Heitor, Manaus, Brazil, ^7^Fundación Huésped, Buenos Aires, Argentina, ^8^Regional Center For Prevention and Treatment of AIDS and Infectious Diseases, Moscow, Russian Federation, ^9^ViiV Healthcare, Brentford, United Kingdom, ^10^GlaxoSmithKline, Stockley Park, United Kingdom, ^11^ViiV Healthcare, Melbourne, Australia, ^12^GlaxoSmithKline, Upper Merion, United States, ^13^ViiV Healthcare, Research Triangle Park, United States

**Background:** Concurrent treatment of tuberculosis (TB) and HIV is challenging owing to drug interactions, overlapping toxicities, and immune reconstitution inflammatory syndrome (IRIS). The efficacy and safety of dolutegravir (DTG) in adults with HIV/TB co‐infection was assessed.

**Methods:** INSPIRING (NCT02178592) is a Phase 3b, non‐comparative, active control, randomised, open‐label study in HIV‐1‐infected ART‐naïve adults (CD4+ 350 cells/μL) with drug‐sensitive TB. Subjects on rifampicin‐based TB treatment ≤8 weeks were randomised (3:2) to receive DTG (50 mg twice daily during and two weeks post‐TB therapy, followed by 50 mg once daily) or EFV (600 mg once daily), with two NRTIs for 52 weeks. The Week 48 primary endpoint was the proportion of DTG subjects with plasma HIV‐1‐RNA <50 copies/mL (responders) using the FDA Snapshot algorithm (intent‐to‐treat exposed (ITT‐E) population). An independent committee adjudicated IRIS episodes. The study was not powered to show a difference between arms; no formal statistical hypothesis was tested.

**Results: ** Subjects were randomised to DTG (n = 69) or EFV (n = 44). Median baseline HIV‐1‐RNA and CD4+ counts were 5.10 log10 copies/mL and 208 cells/μL for DTG and 5.24 log10 copies/mL and 202 cells/μL for EFV. The proportions of Week 48 responders (ITT‐E) were 52/69 (75%) (95% CI: 65%, 86%) for DTG and 36/44 (82%) (95% CI: 70%, 93%) for EFV. The DTG non‐response rate was primarily driven by non‐treatment‐related discontinuations: eleven subjects (16%) for DTG and three (7%) for EFV discontinued due to non‐treatment‐related reasons whilst suppressed (mainly loss to follow‐up). There were two protocol‐defined virological failures (PDVF) and no treatment‐emergent resistance‐associated mutations (RAMs) for DTG and one PDVF in EFV with NRTI and NNRTI RAMs. Week 48 median CD4 +  increases were 220 cells/μL (IQR: 111, 271) for DTG and 190 cells/μL (IQR: 104, 252) for EFV. Two EFV subjects discontinued due to AEs. TB‐associated IRIS rates were low (DTG, n = 4 (6%); EFV, n = 4 (9%)). No subjects discontinued due to IRIS or liver events. TB treatment success was 61/69 (88%) and 39/44 (89%) in DTG and EFV, respectively. Median DTG trough concentrations during twice daily dosing with rifampicin was like that with DTG once daily without rifampicin.

**Conclusions: **These results show that DTG is effective and well‐tolerated in HIV/TB co‐infected adults receiving rifampicin‐based TB treatment.

## TUAC0101

### HIV incidence trends among the general population in Eastern and Southern Africa 2000 to 2014

**E. Slaymaker^1^; J. Todd^1^; M. Urassa^2^; A.J. Herbst^3^; N. McGrath^3,4^; R. Newton^5,6^; D. Nabukalu^7^; A. Crampin^8,9^; C. Nyamukapa^10,11^; K. Tomlin^1^; K. Risher^1^; G. Reniers^1^; M. Marston^1^ and B. Zaba^1^**

^1^London School of Hygiene & Tropical Medicine, Population Health, London, United Kingdom, ^2^National Institute for Medical Research, Mwanza, Tanzania, United Republic of, ^3^Africa Health Research Institute, Durban, South Africa, ^4^University of Southampton, Southampton, United Kingdom, ^5^MRC/UVRI and LSHTM Uganda Research Unit, Entebbe, Uganda, ^6^University of York, York, Uganda, ^7^Rakai Health Sciences Project, Entebbe, Uganda, ^8^Malawi Epidemiology and Intervention Research Unit, Lilongwe, Malawi, ^9^London School of Hygiene & Tropical Medicine, Infectious Disease Epidemiology, London, United Kingdom, ^10^Imperial College, London, United Kingdom, ^11^Biomedical Research and Training Institute, Harare, Zimbabwe

**Background:** Studies from Kenya, Malawi, Tanzania, South Africa, Uganda and Zimbabwe comprise the Network for Analysing Longitudinal, Population‐based HIV/AIDS data on Africa (ALPHA). We used data from six studies to assess whether HIV incidence has changed over time.

**Methods:** Individual participants consented to research HIV tests at regular intervals, typically around two years apart. Person‐time under observation started at the first HIV negative test recorded while resident in the study area. Participants were followed until study exit or seroconversion. Seroconversion dates were estimated using multiple imputation assuming a uniform distribution of seroconversion dates between the last negative and first positive study test result.

We estimated incidence rates and Poisson confidence intervals, by study, sex and age (15 to 19, 20 to 24, 25 to 29, 30‐ to 34, 35 to 39 and 40 to 49) and calendar year between 1995 to 2014. We fitted piecewise exponential models for 2000 to 2014 to estimate age‐adjusted hazard ratios for non‐linear change in HIV incidence over time with random intercepts for study and individual and a random slope for change over time by study.

To acount for exposure to infection we estimated age, sex and period‐specific untreated HIV prevalence among potential heterosexual partners using observed data on age‐mixing between sexual partners and HIV status and treatment status among the opposite sex. We included this in the regression model as an explanatory variable.

**Results: ** There were 1475 seroconversions among 163,613 male person‐years of observation and 3302 among 218,233 female person‐years. Men and women aged 15 to 49 experienced clear incidence rate declines in Rakai and Manicaland; elsewhere women's incidence appeared stable or rising whilst men's was stable or declining (Figure 1).

Adjusted for age, men's incidence declined between 2000 to 2004 and 2010 to 2014 (HR 0.78, Table 1). Adjusting for untreated opposite sex prevalence decreased the effect of calendar year.

Female incidence trends diverged so three models were fitted showing: decline in Manicaland and Rakai, increase in uMkhanyakude and stable elsewhere (Table 1). Trends over time were greater after adjusting for untreated prevalence.

**Conclusions: **Incidence has declined among men, but not among women in all studies, most probably due to higher treatment coverage among women reducing their infectivity to men but not vice versa.

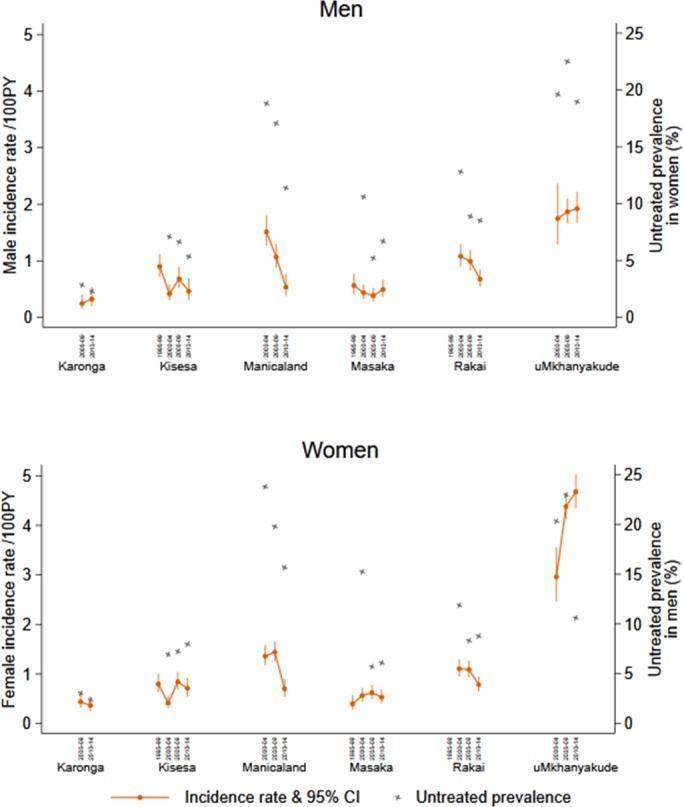

**Abstract TUAC0101‐Figure 1. Crude incidence rates by site, sex and calendar year period and the estimated mean untreated prevalence among sexual partners of the opposite sex.**

**Abstract TUAC0101‐Table 1. Adjusted hazard ratios, 95% confidence intervals and *p*‐values from poisson regression models for men and women adjusted for age and study**

**Table:** 

	MEN	MEN	WOMEN	WOMEN	WOMEN
	All studies	All studies, with untreated prevalence	Manicaland and Rakai	uMkhanyakude	Karonga, Kisesa, Masaka
	Adjusted HR (95% CI)	Adjusted HR (95% CI)	Adjusted HR (95% CI)	Adjusted HR (95% CI)	Adjusted HR (95% CI)
Calendar year					
1995 to 1999	1.01 (0.78 to 1.31)	0.94 (0.73 to 1.19)	0.23 (0.11 to 0.46)	‐	0.78 (0.59 to 1.03)
2000 to 2004	1	1	1	1	0.65 (0.50 to 0.85)
2005 to 2009	0.93 (0.80 to 1.09)	0.96 (0.81 to 1.13)	1.18 (1.00 to 1.41)	1.40 (1.15 to 1.69)	1
2010 to 2014	0.78 (0.62 to 0.98)	0.87 (0.68 to 1.12)	0.78 (0.62 to 0.97)	1.90 (1.51 to 2.38)	0.85 (0.69 to 1.06)
					
Prevalence of untreated HIV in opposite sex partners	‐	1.02 (1.01 to 1.03)	1.03 (1.01 to 1.05)	1.02 (1.01 to 1.03	1.01 (0.98 to 1.03)

## TUAC0102

### Population viral load and recent HIV‐1 infections: findings from population‐based HIV impact assessments (PHIAs) in Zimbabwe, Malawi, and Zambia

**M. Farahani^1^; E. Radin^1^; S. Saito^1^; K. Sachathep^1^; J. Manjengwa^1^; S. Balachandra^2^; A. Low^1^; Y. Duong^1^; S. Jonnalagadda^2^; H. Patel^2^; D. Voetsch^2^; W. Hladik^2^; A. Hakim^2^; N. Ahmed^1^; G.N. Musuka^1^; B.A. Tippett Barr^2^; N. Wadonda‐Kabondo^2^; A.F. Auld^2^; A. Jahn^2^; D.B. Williams^2^; D. Barradas^2^; D. Payne^2^; G. Bello^3^; O. Mugurungi^4^; B. Parekh^2^; D. Hoos^1^ and J. Justman^1^**

^1^Columbia University, ICAP, New York, United States, ^2^U.S. Centers for Disease Control and Prevention ^CDC^, Atlanta, United States, ^3^International Training and Education Center for Health ^I‐TECH^, Lilongwe, Malawi, ^4^Zimbabwe Ministry of Health and Child Care, Harare, Zimbabwe

**Background:** Population viral load (PVL) reflects antiretroviral therapy (ART) program effectiveness and transmission risk in a population. Using nationally representative data from household surveys conducted in Zimbabwe, Malawi and Zambia in 2015‐16, we examined the association between PVL and viral load suppression (VLS) and the probability of at least one recent HIV‐1 infection in the surveys’ smallest geographic sampling unit, an enumeration area (EA).

**Methods:** Viral load (VL) and limiting‐antigen avidity enzyme immunoassay (LAg‐Avidity EIA) testing were performed on all HIV‐1 positive (+) samples. Recent HIV cases were defined by World Health Organization criteria (LAg‐Avidity EIA < 1.5 ODn and HIV RNA > 1000 c/mL), and VLS as HIV RNA < 1000 c/mL. PVL was defined as the arithmetic mean of log10 HIV RNA of HIV+ individuals in the EA, and ART coverage as prevalence of self‐reported current ART use. We used logistic regression adjusted for EA‐level variables, e.g., HIV prevalence, population size and mean age of the female population, to estimate the probability of one recent HIV‐1 infection.

**Results: ** Among 1,510 EAs across the three surveys, a total of 58,366 adults aged 15‐59 years resided in 1,374 (91%) EAs that had at least one HIV+ adult consenting to an interview and blood draw. Among the 1,374 EAs, 92.65%, 6.99% and 0.04% had 0, 1 and 2 recent HIV‐1 cases, respectively. Mean VLS prevalence across these EAs was 63.5% (95% confidence intervals (CI) 62‐65%).

In multivariable analysis, PVL, particularly among those unaware of their HIV+ status, was associated with a recent HIV‐1 case in that EA (adjusted odds ratio [AOR]: 1.44, 95% CI 1.22‐1.70, p < 0.001). VLS prevalence was inversely correlated with recent infections (AOR: 0.17, 95% CI 0.08‐0.37, p < 0.001). On average, every 1% increase in VLS in an EA decreased the predicted probability of one recent infection by 8%.

**Conclusions: **We found a strong association between PVL and VLS prevalence with recent HIV‐1 infection at the EA level in three southern African countries with generalized HIV epidemics. These results suggest expanding and maintaining high levels of VLS may be key to HIV epidemic control in these three countries.

## TUAC0103

### Temporal trends of population viral suppression in the context of Universal Test and Treat: results from the ANRS 12,249 TasP trial in rural South Africa

**J. Larmarange^1,2^; M.H. Diallo^1^; N. McGrath^3,4,5^; C. Iwuji^2,5,6^; M. Plazy^7^; R. Thiébaut^7^; F. Tanser^3^; T. Bärnighausen^2,8,9^; J. Orne‐Gliemann^7^; D. Pillay^2,10^; F. Dabis^7^ and TasP ANRS 12,249 Study Group**

^1^Ceped, Institut de Recherche pour le Développement ^IRD^, Université Paris Descartes, Inserm, Paris, France, ^2^Africa Health Research Institute, Mtubatuba, South Africa, ^3^Africa Health Research Institute, University of KwaZulu‐Natal, School of Nursing and Public Health, Durban, South Africa, ^4^Faculty of Medicine and Faculty of Social, Human and Mathematical Sciences, University of Southampton, Southampton, United Kingdom, ^5^Research Department of Infection and Population Health, University College London, London, United Kingdom, ^6^Department of Global Health & Infection, Brighton and Sussex Medical School, Brighton, United Kingdom, ^7^Bordeaux University, School of Public Health ^ISPED^, Inserm, Bordeaux Population Health Research Center, UMR 1219, Bordeaux, France, ^8^Department of Global Health & Population, Harvard School of Public Health, Harvard University, Boston, United States, ^9^Heidelberg University, Institute of Public Health, Faculty of Medicine, Heidelberg, Germany, ^10^University College London, Division of Infection and Immunity, London, United Kingdom

**Background:** The universal test‐and‐treat strategy (UTT) aims to maximize the proportion of all people living with HIV (PLWHIV) on antiretroviral treatment (ART) and virally suppressed in a community, i.e. to reach population viral suppression (PVS). The ANRS 12,249 TasP trial did not demonstrate an impact of universal ART on HIV incidence at population level (Lancet HIV 2017). Here, we investigated whether PVS improved during the course of the trial: differentially by arm, according to trial interventions or contextual changes.

**Methods:** The TasP cluster‐randomized trial (2012 to 2016) implemented six‐monthly repeated home‐based HIV counselling and testing (RHBCT) and referral of PLWHIV to local HIV clinics in 2×11 clusters opened sequentially. ART was initiated according to national guidelines in control clusters vs. regardless of CD4 count in intervention clusters.

Test results, clinic visits, ART prescriptions, viral loads, CD4 counts, migrations and deaths were used to produce information on residency status, HIV status and HIV care status for each participant. PVS was computed daily and per cluster among all resident PLWHIV (≥16, including those not in care). We used a mixed linear model to explore the relation between PVS with calendar time, time since cluster opening, trial arm and interaction between arm and time since cluster opening, adjusting on sociodemographic changes at cluster level.

**Results: ** 8646 PLWHIV were observed. Between 1 January 2013 and 1 January 2016, PVS increased significantly in both arms (intervention: 29.0% to 46.2%, +17.2, *p* < 0.001; control: 32.4% to 44.6%, +12.2, *p* < 0.001), but difference in temporal variation (+5.0%) was not significant (*p* = 0.175).

According to adjusted model (figure) this increase was mainly attributable to RHBCT (measured by time since cluster opening). They were also some effect due to contextual changes (measured by calendar time). The effect attributable to universal ART (interaction term) was limited.

**Conclusions: **Although suboptimal, the UTT strategy implemented in TasP trial improved PVS over time. As it was mainly due to RHBCT rather than universal ART, it did not induce differences between arms, explaining the null effect observed on cumulative incidence, the main trial finding. Changes in ART initiation guidelines alone are not enough to significantly increase PVS.

## TUAC0104

### Trends in percent time spent viremic among persons newly diagnosed with HIV, San Francisco, CA, USA, 2008 – 2016

**A. Hughes; L. Hsu and S. Scheer**

San Francisco Department of Public Health, San Francisco, United States

**Background:** The risk of sexual HIV transmission increases when HIV viral load (VL) is above 1500 copies/mL. As such, persons newly diagnosed with HIV are at greater risk of transmission until they initiate ART and achieve sustained viral suppression. We sought to examine trends in time spent above three viral thresholds among persons newly diagnosed with HIV in San Francisco (SF).

**Methods:** We analyzed data from the HIV surveillance registry. Persons were included if they were diagnosed with HIV during 2008 to 2016, were SF resident at time of diagnosis, alive 12 months after HIV diagnosis and had ≥2 VL tests within 12 months after diagnosis. Consecutive VL pairs were used to calculate percent of person‐time (pPT) spent above 200 copies/mL (pPT>200), 1500 copies/mL (pPT>1500) and 10,000 copies/mL (pPT>10,000) for the 12 months after HIV diagnosis. Multivariate zero‐inflated negative binomial regression was used to assess trends in year of diagnosis and time spent above each viral threshold, while controlling for covariates (gender, transmission category, race/ethnicity, age, housing status, CD4+ lymphocyte count, health insurance type, and time from HIV diagnosis to ART initiation).

**Results: ** Of the 3336 new HIV diagnoses from 2008 to 2016, 2556 (77%) met inclusion criteria for analysis. Overall, persons newly HIV diagnosed spent 53.6% of pPT>200, 44.1% pPT>1500, and 31.7% pPT>10,000. By year, pPT>200 decreased from 70.3% in 2008 to 31.9% in 2016, pPT>1500 decreased from 62.3% in 2008 to 24.8% in 2016 and pPT>10,000 decreased from 46.0% in 2008 to 17.0% in 2016 (*p* < 0.0001 for each threshold; see Figure). In adjusted regression, significant differences for all three pPT thresholds were found by transmission category, age, CD4 count, and time from HIV diagnosis to ART initiation. PWID (including MSM‐PWID) and younger age were associated with increased pPT viremic. Persons with lower CD4 count and shorter time to ART initiation had decreased pPT viremic.

**Conclusions: **The percent time spent above each viremic level decreased significantly among newly diagnosed persons from 2008 to 2016. Thus, the possibility that persons with a recent HIV diagnosis could transmit HIV in more recent years decreased and likely contributed to the decreased HIV incidence observed in SF.

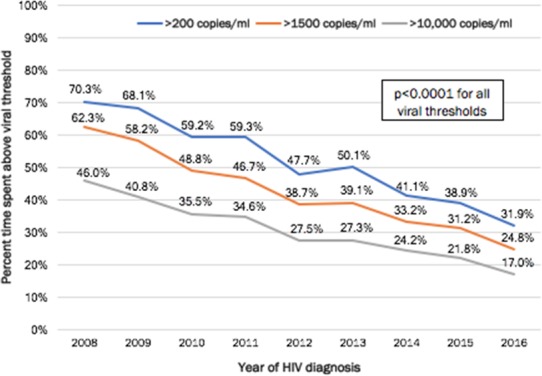

**Abstract TUAC0104‐Figure 1. Percent time spent above each viral threshold during 12 months after HIV diagnosis by year of HIV diagnosis, San Francisco, CA, 2008 to 2016.**

## TUAC0105

### HIV prevention in a Fast Track City: trends in time‐dependent HIV cascade indicators among gay and bisexual men attending high HIV caseload testing services in Melbourne, Australia

**M. Stoove^1,2^; J. Asselin^1^; C. El‐Hayek^1^; S. Lewin^3,4^; B. Allan^5^; J. Hoy^6^; E. Wright^1,6^; S. Ruth^7^; J. Mills^6,8^; C. Carter^9^; M. West^10^; J. Manwaring^10^; A. Fischer^4^; A. Wilkinson^1,11^; R. Guy^12^; B. Donovan^12^; D. Calendar^12^; N. Higgins^10^; P. Locke^7^; N. Roth^13^; B. Tee^14^; J. Wilcox^15^; L. Nguyen^1^; K. Ryan^1,2^ and M. Hellard^1,2,6^**

^1^Burnet Institute, Disease Elimination Program, Public Health Discipline, Melbourne, Australia, ^2^Monash University, School of Population health & Preventive Medicine, Melbourne, Australia, ^3^Peter Doherty Institute for Infection and Immunity, Melbourne, Australia, ^4^The Peter Doherty Institute for Infection and Immunity, University of Melbourne, Melbourne, Australia, ^5^International Council Of AIDS Service Organizations, Toronto, Canada, ^6^The Alfred Hospital and Monash University, Department of Infectious Diseases, Melbourne, Australia, ^7^Victorian AIDS Council, Melbourne, Australia, ^8^Burnet Institute, Melbourne, Australia, ^9^North‐West Melbourne Public Health Network, Melbourne, Australia, ^10^Victoria Department of Health and Human Services, Melbourne, Australia, ^11^Cancer Council Victoria, Melbourne, Australia, ^12^Kirby Institute, University of New South Wales, Sydney, Australia, ^13^Prahran Market Clinic, Melbourne, Australia, ^14^Centre Clinic, Victorian AIDS Council, Melbourne, Australia, ^15^Northside Clinic, Melbourne, Australia

**Background:** The Fast‐Track Cities partnership focuses on translating global HIV goals into local strategies, with progress towards 90‐90‐90 targets used as key indicators. Melbourne is Australia's only Fast Track City and, alongside Amsterdam, has reported achieving 90‐90‐90 targets. Sentinel surveillance in Melbourne also enables monitoring of individuals’ progress through the HIV cascade, producing indicators highly sensitive to change. With 78% of HIV diagnoses in Melbourne occurring among gay and bisexual men (GBM), we present trends in HIV testing, diagnosis rates, and time to viral suppression among GBM attending three general practices specialising in GBM health and one GBM peer‐led testing service in Melbourne.

**Methods:** HIV testing data was extracted from patient management systems through the Australian Collaboration for Coordinated Enhanced Sentinel Surveillance (ACCESS) between Jan 2012‐Sep 2017. Using anonymised unique identifiers, ACCESS prospectively links data on individuals’ clinic attendances and laboratory test results. We calculated annualised trends in repeat testing (follow‐up tests within three, six and 12‐months), HIV positivity, the proportion of GBM with undetectable viral loads (<200 copies/ml) within 12 months of diagnosis and median time between diagnosis and undetectable viral load.

**Results: ** 11,607 GBM received 47,722 HIV diagnostic tests between 2012 and 2017. There were significant increases in 12‐month (56% to 63%; *p* < 0.01), six‐month (28% to 44%; *p* < 0.001) and three‐month (10% to 22%; *p* < 0.001) repeat testing. Between 2012 and 2017, 292 GBM were newly diagnosed with HIV; HIV positivity was 1.5% in 2012, peaked at 2.2% in 2014 then declined significantly to 0.4% in 2017 (*p* < 0.001). Among GBM newly diagnosed between 2012 and 2016, the percentage with undetectable viral loads (<200 copies/ml) within 12 months of diagnosis increased from 59% to 97% (*p* < 0.001) and the median time between diagnosis and undetectable viral load declined from 162 days to 50 days (*p* < 0.001).

**Conclusions: **Considerable declines in time between HIV diagnosis and viral suppression among GBM attending specialist HIV testing services in Melbourne have coincided with substantial declines in HIV diagnosis rates at these services. The most recent declines in diagnoses also coincided with a substantial scale up of PrEP among GBM in 2016 to 2017 as part of a multi‐site implementation project.

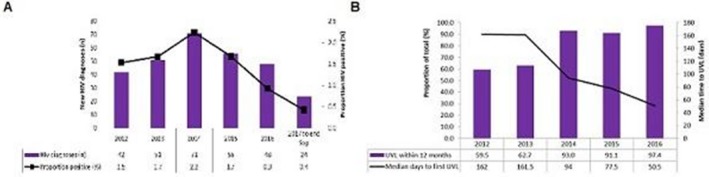

**Abstract TUAC0105‐Figure 1. HIV diagnoses and post‐diagnosis viral suppression among GBM attending specialist HIV service in Melbourne, 2012 to 2017.**

## TUAC0201

### Impact of HIV combination prevention in men who have sex with men, Bangkok, Thailand

**S. Pattanasin^1^; B. Cadwell^2^; D.K. Smith^2^; W. Sukwicha^1^; P.A. Mock^1^; W. Wimonsate^1^; C. Ungsedhapand^1^; P. Sirivongrangson^3^; M.C. Thigpen^1,2^ and E.F. Dunne^1,2^**

^1^Thailand MOPH‐U.S. CDC Collaboration, Nonthaburi, Thailand, ^2^Division of HIV/AIDS Prevention, Centers for Disease Control and Prevention, Atlanta, United States, ^3^Ministry of Public Health, Department of Disease Control, Nonthaburi, Thailand

**Background:** PrEP is recommended for HIV prevention among at risk men who have sex with men (MSM) in Thailand. Anticipated PrEP impact on reduction of HIV infections would support the expansion of programs implementing PrEP in combination with consistent condom use. We used a deterministic model to assess PrEP and condom use and estimated HIV infections among MSM in Bangkok, Thailand using different adherence attributes.

**Methods:** For this deterministic model previously developed by Smith et al, we used three parameters from published data: (1) PrEP effectiveness from the iPrEx international clinical trial among MSM having < 50% adherence and ≥90% adherence based on self‐reported medication adherence and pill counts (2) condom effectiveness from self‐reported condom use during anal sex among MSM reporting at least one HIV‐positive partner in two prospective HIV prevention trials and (3) annual HIV incidence in the absence of PrEP from the Bangkok MSM Cohort study, overall and stratified by characteristics. Among different age groups and risk behaviors, and adherence categories, the number of HIV infections per 10,000 MSM each year was calculated. The estimates assumed that PrEP and condom use efficacy were independent.

**Results: ** Among MSM who had ≥90% PrEP adherence, an estimated 46 (19 to 110) HIV infections would occur among those who used condoms consistently; and an estimated 590 (520 to 680) HIV infections would occur among those not taking PrEP and not using condoms (a reduction of 544 HIV infections). Among MSM who had < 50% PrEP adherence, an estimated 116 (52 to 260) HIV infections would occur if condoms were used consistently. Combination HIV prevention used in MSM who were ages 18 to 21 years, or who engaged in sex parties, or who used club drugs with sex resulted in the greatest reduction of HIV infections (a reduction of 811, 922, and 1493 HIV infections) compared to those not using PrEP and condoms.

**Conclusions: **Models predict an estimated >10 fold reduction of HIV infections each year for MSM using PrEP and condoms in Bangkok, Thailand. Adherent combination prevention strategies in the highest risk MSM could result in the most substantial reductions in HIV infection.

## TUAC0202

### Changes in rectal STI incidence and behavioral HIV risk before, during, and after PrEP in a national sample of gay and bisexual men in the United States

**J. Parsons^1^; H.J. Rendina^1^; T. Whitfield^2^ and C. Grov^3^**

^1^Hunter College‐CUNY, Psychology, New York, United States, ^2^Center for HIV Educational Studies and Training, New York, United States, ^3^CUNY School of Public Health, New York, United States

**Background:** There has been mixed evidence about the extent to which gay and bisexual men (GBM) who use PrEP engage in greater condomless anal sex (CAS) and acquire STIs more frequently. Many studies rely on between‐group comparisons of PrEP users and non‐users or lack longitudinal data before and after PrEP initiation. The current study sought to examine within‐person changes among men who electively initiated—and in some cases, discontinued—PrEP while enrolled in an observational study.

**Methods:** Data were taken from a longitudinal study of 1071 HIV‐negative GBM from across the U.S. Participants were tested annually for rectal gonorrhea and chlamydia and reported on PrEP use and CAS. We examined the odds of diagnosis with a rectal STI, number of CAS acts with casual male partners, and receptive CAS acts with serodiscordant casual male partners by PrEP status using general estimating equations; models were adjusted for visit year, relationship status, and race.

**Results: ** We analyzed all data from the 281 (26.2%) GBM reporting PrEP use at one or more visits—1012 person‐visits of data were examined, of which 517 (51.1%) were prior to initiation, 406 (40.1%) were while on PrEP, and 89 (8.8%) were after discontinuation. Overall prevalence of rectal STIs was 8.6%, with no significant changes in the odds while on PrEP (AOR = 1.28, *p* = 0.36) or after discontinuing (AOR = 0.28, *p* = 0.08) compared to pre‐uptake. There were significant increases in CAS with casual male partners (AOR = 2.49, *p* < 0.001) and receptive CAS with serodiscordant male partners (AOR = 8.02, *p < *0.001) while on PrEP compared to before uptake; no significant differences were observed in CAS comparing pre‐PrEP and post‐PrEP assessments.

**Conclusions: **Despite substantial within‐person increases in CAS, including receptive CAS with serodiscordant partners, we failed to observe statistically significant increases in rectal STI incidence. It is likely that the desire for CAS was a motivator for initiating PrEP and GBM feel more comfortable having CAS while on PrEP. At discontinuation, however, CAS returned to pre‐PrEP use levels, suggesting GBM are quite capable of other HIV prevention strategies when no longer on PrEP.

## TUAC0203

### Reducing risk of male sex partners: HIV testing, treatment, and VMMC of men in PEPFAR‐supported DREAMS districts

**C. Cooney^1^; K. Sato^2^; S. Allen^1^; N. Toiv^1^; D.H. Watts^1^ and J. Saul^1^**

^1^U.S Department of State, Office of the U.S. Global AIDS Coordinator and Health Diplomacy, Washington, D.C., United States, ^2^U.S. Peace Corps, Washington, D.C., United States

**Background:** The U.S. President's Emergency Plan for AIDS Relief's (PEPFAR) launched the Determined, Resilient, Empowered, AIDS‐Free, Mentored and Safe (DREAMS) Public‐Private Partnership in 2014 with the goal of preventing HIV in adolescent girls and young women (AGYW) in ten high HIV burdened African countries. PEPFAR complemented DREAMS activities for AGYW with increased funding to ensure saturation of HIV services for young adult men in DREAMS districts, including HIV testing (HTS), treatment, and voluntary medical male circumcision (VMMC).

Here we compare DREAMS and similar non‐DREAMS districts in five countries to examine changes in HTS, VMMC, and treatment results after two years of DREAMS implementation (2015 to 2017).

**Methods:** Countries were selected for inclusion if they met the following criteria: there were comparable non‐DREAMS districts that were originally considered for DREAMS or will begin DREAMS implementation in 2018; and districting remained consistent from 2015 to 2017.

PEPFAR indicators were used to analyze total number of VMMCs completed, clients tested, and new clients on treatment for males 15 to 49 years in DREAMS and non‐DREAMS districts. Annual results from 2015 and 2017 were compared and a percent change was calculated. A series of generalized linear mixed models, based on a Poisson distribution, were used to assess the difference between DREAMS vs. non‐DREAMS districts. We controlled for false discovery rate for multiple hypothesis testing.

**Results: ** The generalized linear mixed models showed significant positive differences (*p*‐value <0.001) between DREAMS vs. non‐DREAMS districts in three countries for an increase in new on treatment, three countries for increase in total number tested, and two countries for an increase in VMMCs supported. Results are shown in the table.

**Conclusions: **Change in VMMC, HTS, and treatment results for men ages 15 to 49 in DREAMS vs. non‐DREAMS districts varied. In DREAMS districts that showed a greater percent increase vs. non‐DREAMS districts in testing and treatment indicators, data suggest that focusing on treatment as prevention for male partners during DREAMS implementation may be associated with an increase in these services. Variation in results emphasizes the need for continued efforts in reaching young adult men and in reaching AGYW with structural and combination prevention to interrupt the cycle of heterosexual HIV transmission.

**Abstract TUAC0203‐Table 1. Percent Change in DREAMS vs. Non‐DREAMS PEPFAR Districts in VMMC, HTS, and Treatment Results for Males Ages 15 to 49 (2015 to 2017)**

**Table:** 

	VMMC			HIV Testing			New on Treatment		
Country	DREAMS	Non‐DREAMS	Coefficient (Standard Error)	DREAMS	Non‐DREAMS	Coefficient (Standard Error)	DREAMS	Non‐DREAMS	Coefficient (Standard Error)
Kenya	−11%	−41%	0.449 (0.016)***	123%	50%	0.396 (0.003)***	1%	5%	−0.042 (0.022)
Malawi	23%	−55%	0.967 (0.025)***	99%	−17%	0.874 (0.007)***	21%	−24%	0.462 (0.031)***
Mozambique	2%	77%	−0.554 (0.019)***	N.D.	N.D.	N.D.	1739%	1608%	0.455 (0.080)***
Zambia	112%	16%	0.605 (0.012)***	74%	86%	−0.064 (0.005)***	55%	50%	0.028 (0.021)
Zimbabwe	−4%	−11%	0.068 (0.016)***	93%	78%	0.083 (0.006)***	606%	302%	0.644 (0.042)***

ND, not determined; Mozambique did not report age/sex disaggregated data for HIV testing in 2015. ***p < 0.001; **p < 0.01; *p < 0.05.

## TUAC0204

### Profile of adverse events in a national VMMC program in Mozambique (2009 to 2017): reduction in AE with a national scale‐up, but three events require further attention

**H. Muquingue^1^; S. Ndimande^2^; E. Necochea^2^; S. Wei^3^; R. Frescas^2^; I. Malimane^3^; A. Jaramillo^2^; M. Mahomed^4^; C. Lee^5^ and M. Canda^3^**

^1^Jhpiego Mozambique, MER, Maputo, Mozambique, ^2^Jhpiego Mozambique, Maputo, Mozambique, ^3^CDC Mozambique, Maputo, Mozambique, ^4^Jhpiego South Africa, Durban, South Africa, ^5^Jhpiego, Baltimore, United States

**Background:** Adverse events (AE) in male circumcision are “any injuries, harm, or undesired outcomes occurring during or following male circumcision (MC) that would not have occurred if the client had not undergone the procedure” (USAID). AEs are categorized by severity (mild, moderate and severe), timing (intra or post‐operative), nature (bleeding, etc), and surveillance (passive or active). AE occurrence is a proxy for quality management and provision, and influences MC acceptability. Thus, efforts try to both understand the program‐ and beneficiary‐related etiologies of AE and monitor AE, particularly in large scale MC programs, given the potential risks involved.

**Methods:** We report on AE identified within males circumcised in Mozambique by Jhpiego, from 2009 to 2017 with more than 700,000 VMMC procedures provided through a national program. Data on AE events in Mozambique VMMC program were analyzed, using descriptive statistics and trends. AEs were calculated per age group and severity (moderate or severe).

**Results: ** Out of 718,090 procedures, there were 1826 moderate and severe AEs (0.25% rate, peaking at 2.3%, in the first month), with 1650 moderate (90.4%) and 176 severe (9.6%). The three most common AEs (80% of AE) were infection (41%), hematoma (23%) and excessive bleeding (16%). Damage to the penis and pain accounted for 6% each. Most moderate AEs were infections (44.2%), and most severe AE were hematomas (59.1%). The most affected age was 10 to 14 years, with 40% of the AEs, followed by 15 to 19, and 20 to 24 years, with 24% each. By age strata, AE were 0.2% (10 to 14), 0.22% (15 to 19; 35 to 49), 0.8% (50+); 0.29% (30 to 34); 0.37% (25 to 29); 0.57% (20 to 24).

**Conclusions: **A high volume VMMC program has the potential to dramatically reduce AE occurrence, overtime. The high prevalence of infection as an AE may be related to both client and provider factors, which require further consideration; parents and caregivers need to understand proper wound care; providers need to assure proper technique during MC procedures. A challenging limitation is that AE reporting depends on providers recognizing, documenting and addressing adverse events, therefore results should be carefully backed by an active surveillance system to improve the reliability of data and seek those opportunities for quality improvement.

## TUAC0205

### Results from a cluster‐randomized trial to evaluate a microfinance and peer health leadership intervention for HIV and intimate partner violence prevention among social networks of young Tanzanian men

**S. Maman^1^; M. Mulawa^2^; P. Balvanz^1^; H.L. McNaughton‐Reyes^1^; M. Kilonzo^3^; T. Yamanis^4^; B. Singh^5^ and L. Kajula^3^**

^1^University of North Carolina at Chapel Hill Gillings School of Global Public Health, Health Behavior, Chapel Hill, United States, ^2^Duke University, Global Health Institute, Durham, United States, ^3^Muhimbili University of Health and Allied Sciences, Department of Psychiatry and Mental Health, Dar es Salaam, Tanzania, United Republic of, ^4^American University, School of International Service, Washington, United States, ^5^Medical University of South Carolina, Department of Psychiatry and Behavioral Sciences, Charleston, United States

**Background:** Despite calls to engage men in HIV and intimate partner violence (IPV) prevention efforts, effective approaches to reach and engage men in low‐resource, high‐HIV prevalence settings are limited. We identified and engaged social networks of mostly young men, locally referred to as “camps,” in Vijana Vijiweni II, a cluster‐randomized trial to evaluate the efficacy of a combined microfinance and peer health leadership intervention for HIV and IPV prevention among 59 camps in Dar es Salaam, Tanzania.

**Methods:** Thirty camps (n = 621 men) were randomly assigned to the two‐year intervention condition and 29 camps (n = 628 men) were randomized to the control. Behavioral assessments were conducted at baseline and 30‐months post‐intervention launch, with biological samples drawn at 30‐months to test for sexually‐transmitted infections (STIs). Primary outcomes included prevalence of STIs and past‐year IPV perpetration. Secondary outcomes included STI sexual risk behaviors and past‐year HIV testing. Proximal intervention targets included inequitable gender norm attitudes and hope. We compared outcomes among intervention vs. control participants by computing covariate‐adjusted risk ratios (aRR) with robust confidence intervals (CI) using an intention‐to‐treat approach.

**Results: ** Of 1249 men enrolled in the trial, 1029 (82.4%) completed the 30‐month follow‐up. There was no evidence that the intervention reduced STI prevalence (aRR 1.05, 95% CI 0.86 to 1.29), IPV perpetration (aRR 1.15, 95% CI 0.91 to 1.44), or STI risk behaviors. Men in the intervention condition reported greater levels of past‐year HIV testing, controlling for HIV testing status at baseline, (aRR 1.13, 95% CI 1.00 to 1.28, *p* = 0.04) as well as significantly lower levels of inequitable gender norm attitudes at the 30‐month follow‐up (adjusted effect −0.11, 95% CI −0.21 to 0.00, *p* = 0.04), while there was no significant difference in hope across condition.

**Conclusions: **We successfully engaged and retained social networks of men in this multilevel HIV and IPV prevention study. The combined microfinance and peer health leadership intervention successfully improved HIV testing and reduced inequitable gender norm attitudes. We did not see an effect on the primary outcomes or STI sexual risk behaviors. Additional analyses will examine whether there were effects for particular subgroups and whether there were differential effects as a function of intervention exposure.

## TUAC0301

### Retention in care for HIV pre‐exposure prophylaxis (PrEP) among sex workers of four public health centers in Senegal

**O. Diouf^1^; M. Sarr^2^; D. Gueye^1^; M. Mane^1^; A. Mboup^1^; C. Toure Kane^1^; S.E. Hawes^3^; C. Suarez^2^; M.D. Bousso Bao^1^; F. Jones^2^; J. Presley^4^; G. Gottlieb^3^ and S. Mboup^1^**

^1^IRESSEF : Institut de Recherche en Santé, de Surveillance Epidemiologique et de Formations, Dakar, Senegal, ^2^Westat, Rockville, United States, ^3^University of Washington, Seattle, United States, ^4^Bill and Melinda Gates Foundation, Seattle, United States

**Background:** Recent breakthroughs in antiretroviral (ARV)‐based prevention provide new opportunities to rethink HIV prevention strategies, especially for key populations such as female sex workers (FSWs). As pre‐exposure prophylaxis (PrEP) demonstration projects are increasingly initiated, more information is needed about the correlates of PrEP retention when implemented in Ministry of Health (MoH)‐run clinics such as in Senegal.

**Methods:** The Senegal PrEP Demonstration Project is a prospective, open‐label cohort study assessing the delivery of oral Truvada (emtricitabine/tenofovir DF) PrEP to FSWs in 4 Ministry of Health (MoH)‐run clinics in Dakar, Senegal. We assessed retention in PrEP care at 6 and 12‐months follow‐up. Repeated measures analysis using Generalized Estimating Equation (GEE) models were used to identify the predictors of PrEP retention

**Results: ** Overall, out of 325 eligible FSWs, 271 (83.4%) were initially enrolled at baseline. The average age of those enrolled was 38 years (STD = 8.7). Most FSWs were Senegalese (96.7%), and approximately half of them never attended school (44.8%). Among the 267 participants who were prescribed PrEP, 70.4% were retained in care at six months (Pikine: 68.5%, Mbao: 78.8%, Rufisque: 71.2%, Diamniadio: 65.8%; *p* = 0.439) and 67% were retained in PrEP care at twelve months (Pikine: 69.9%, Mbao: 61.5%, Rufisque: 72.7%, Diamniadio: 63.2%; *p* = 0.483). Older age among FSW was found to be a significant predictor of higher PrEP retention (*p* = 0.0012). Compared to the 18 to 24 year age group, the 25 to 34 (OR = 2.53, 95%CI = 1.22 to 4.99), 35 to 44 (OR = 3.24, 95%CI = 1.57 to 6.23), and 45+ year age groups (OR = 3.85, 95%CI = 2.13 to 10.27) were significantly more likely to be retained in PrEP. We did not find significant differences in retention by site, education, registration as sex worker status, condom use or HIV risk perception.

**Conclusions: **Our results showed evidence of good PrEP retention rates among FSWs at 12‐months follow‐up when offered in Ministry of Health (MoH)‐run clinics in Dakar, Senegal, with older age as the only significant predictor of higher PrEP retention. Further research is needed to identify the factors that may optimize retention in PrEP care in public health settings.

## TUAC0302

### Key population‐led health services (KP‐LHS) critical to PrEP introduction among MSM and TG in Thailand

**R. Vannakit^1^; M. Kim^1^; S. Charoenying^2^; S. Mills^2^; M. Avery^2^; N. Phanuphak Pungpapong^3^; P. Phanuphak^3^; D. Rinjongrat^4^; S. Janyam^5^; P. Chanlearn^6^; S. Sittikarn^7^ and T. Nakpor^8^**

^1^Office of Public Health, U.S. Agency for International Development, Regional Development Mission Asia, Bangkok, Thailand, ^2^FHI 360 and USAID LINKAGES Project, Bangkok, Thailand, ^3^Thai Red Cross Aids Research Center, Bangkok, Thailand, ^4^Rainbow Sky Association of Thailand, Bangkok, Thailand, ^5^Service Workers in Group, Bangkok, Thailand, ^6^Mplus Foundation, Chiang Mai, Thailand, ^7^Caremat, Chiang Mai, Thailand, ^8^Sisters Foundation, Pattaya, Thailand

**Background:** PrEP is a necessary component of a national AIDS program in many settings, but particularly in countries with concentrated epidemics among key populations (KPs). There are to date only a few countries who have approved PrEP and even fewer who have attempted rapid scale‐up for epidemic control.

**Methods:** PrEP is available through multiple providers in Thailand, including 13 government hospitals, 5 public‐ and private‐sector clinics, and 7 community‐based organizations working under a key population‐led health services (KP‐LHS) model. We examined data from sites to assess the contribution of KP‐LHS as part of a national strategy to increase PrEP uptake.

**Results: ** Between October 2016 and January 2018, the cumulative number of reported PrEP users in Thailand from all sources increased by more than 200%. PrEP uptake as a percentage of all clients tested HIV‐negative was slightly higher at government facilities than through community‐based providers (7% vs. 5%) but the total number of KP clients receiving PrEP under the KPLHS model was six times higher (1299 vs. 183). PrEP uptake at a private‐sector clinic targeting men who have sex with men was significantly higher (1205 PrEP clients, 16%) but PrEP clients at that clinic were significantly more likely to be non‐Thai clients compared with clients at community‐based services (88% vs. 8%). Over the 15‐month period, none of the clients who accessed PrEP through hospital services seroconverted; four clients who accessed community‐based PrEP later tested HIV‐positive (0.2%), though it is impossible to ascertain whether these clients were actively using PrEP at the time of infection.

**Conclusions: **Our study on the scale‐up of PrEP showed that the most significant increase of clients requesting PrEP were at KP‐LHS sites as well as private clinics whose services are provided by KPs. These organizations appear to be the most critical for rapid scale‐up, particularly where epidemic growth is concentrated among KPs. Government health facilities are still needed for sustainability and coverage. However, our data suggest that in the early stages of introduction, KP‐LHS can quickly add new products such as PrEP and obtain high uptake through their trusted relationships with KPs.

## TUAC0303

### Comparison of measures of adherence to HIV pre‐exposure prophylaxis (PrEP) among men who have sex with men (MSM) and transgender women (TGW): results from the PrEP Brasil study

**L.M.S. Marins^1^; T.S. Torres^1^; I. Costa Leite^2^; R.I. Moreira^1^; B. Hoagland^1^; E.G. Kallas^3^; J.V. Madruga^4^; P.L. Anderson^5^; A.Y. Liu^6^; B. Grinsztejn^1^; V. Veloso^1^ and PrEP Brasil Study Team**

^1^Fundação Oswaldo Cruz, Instituto Nacional de Infectologia Evandro Chagas, Rio de Janeiro, Brazil, ^2^Fundação Oswaldo Cruz, Escola Nacional de Saúde Pública, Rio de Janeiro, Brazil, ^3^Universidade de São Paulo, School of Medicine, São Paulo, Brazil, ^4^Centro de Referência e Treinamento em DST/AIDS, São Paulo, Brazil, ^5^University of Colorado, Skaggs School of Pharmacy and Pharmaceutical Sciences, Denver, United States, ^6^San Francisco Department of Public Health, Bridge HIV, San Francisco, United States

**Background:** Adherence is a critical factor for efficacy of daily emtricitabine/tenofovir (FTC/TDF) for PrEP. We examined the concordance between three adherence measures contrasted to protective drug levels among participants retained through 48 weeks in the PrEP Brasil Study.

**Methods:** PrEP Brasil was a prospective, multicentre, open‐label demonstration project assessing PrEP delivery for MSM and TGW at higher risk for HIV infection in the context of the Brazilian Public Health System. Three adherence measures were obtained at week 48: Self‐report (in‐person interview; 30‐days recall), Pill count (tablets dispensed in prior visit minus tablets returned at week 48, divided by the days between the two visits) and Medication possession ratio (MPR) (ratio between tablets dispensed in prior visit and days between the two visits). TFV‐DP was measured using LC‐MS/MS at week 48. Areas under the ROC curve (AUC) were used to evaluate the concordance between achieving protective drug levels (TFV‐DP≥700 fmol/punch) and the adherence measures. The optimal cut‐off points for discriminating between those with/without protective drug levels were found based on Youden index and distance to corner. Sensitivity, specificity, negative (NPV) and positive (PPV) predictive values for the cut‐off points were calculated. Finally, we carried out the DeLong test to verify whether the curves are different from each other.

**Results: ** From April/2014 to July/2016, 450 participants initiated PrEP, 375(83.3%) were retained through 48 weeks. Of these, FTC/TDF was provided to 354(94.4%) in the previous 3 months and those participants were included in this analysis. Median age was 30 years (IQR: 25 to 35); 84(23.7%) were aged 18 to 24 years, 19(5.4%) TGW, 41(11.8%) black and 83(24.5%) had < 12 years of education. At week‐48, 77.4% (274/375) had TFV‐DP ≥700 fmol/punch. All adherence measures were able to discriminate between participants with and without protective drug levels (AUC>0.5) (Table 1). High recorded adherence was predictive of protective drug levels (PPV>0.8) while low recorded adherence was fairly predictive of lack of protective drug levels (NPV< 0.4). No statistical differences were found between the adherence methods curves (*p* = 0.38) (Figure 1).

**Conclusions: **Low‐burden measurements such as MPR and self‐report can be used to predict PrEP adherence in a public health context in Brazil.

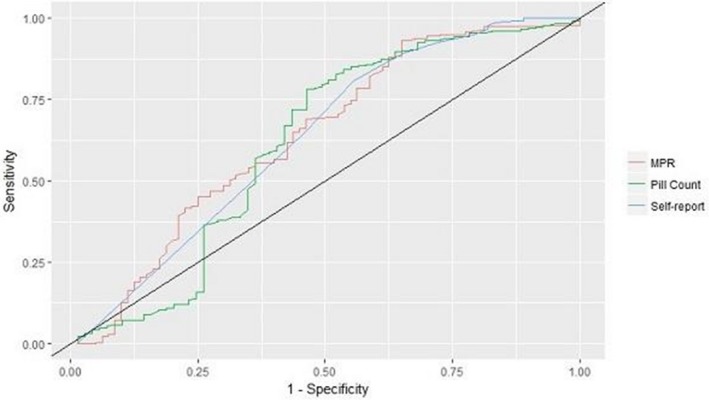

**Abstact TUAC0303‐Figure 1. ROC Curves for protective drug level versus adherence measures.**

**Abstact TUAC0303‐Table 1. Association between adherence measures and protective drug levels**

**Table:** 

Adherence measures	Median adherence (IQR)	AUC (95%CI)	*p*‐value	Cut‐off point	Sensitivity	Specificity	PPV	NPV
MPR	1.16 (1.00 to 1.31)	0.64 (0.56 to 0.71)	<0.001	1.07	0.69	0.54	0.84	0.34
Pill count	96.82 (88.15 to 100.00)	0.62 (0.53 to 0.70)	0.01	90.24	0.77	0.54	0.86	0.38
Self‐report	100.00 (96.67 to 100.00)	0.64 (0.56 to 0.71)	<0.001	100.00	0.65	0.54	0.84	0.29

## TUAC0304

### Uptake of PrEP within clinics providing integrated family planning and PrEP services: results from a large implementation program in Kenya

**K. Mugwanya^1^; J. Pintye^1^; J. Kinuthia^1,2^; F. Abuna^3^; H. Lagat^3^; M. Serede^3^; J. Sila^3^; G. John‐Stewart^4^; J.M. Baeten^4^; PrEP Implementation for Young Women and Adolescents (PrIYA) Program**

^1^University of Washington, Department of Global Health, Seattle, United States, ^2^Kenyatta National Hospital, Nairobi, Kenya, ^3^University of Washington‐Kenya, Nairobi, Kenya, ^4^University of Washington, Departments of Global Health, Epidemiology and Medicine, Seattle, United States

**Background:** Integrating PrEP within family planning (FP) clinics could serve as a platform for reaching young women at‐risk for HIV in high burden settings. The PrEP Implementation for Young Women and Adolescents (PrIYA) Program is conducting real‐world delivery of PrEP to at‐risk women seeking routine FP services in Western Kenya.

**Methods:** PrIYA is part of the DREAMS Innovation Challenge funded by PEPFAR managed by JSI Research & Training Institute, Inc. We approached HIV‐uninfected women seeking routine FP services at 16 clinics in Kisumu County, Kenya from June to December 2017. At each encounter, women were screened for behavioral risk factors and willingness to consider PrEP following the national PrEP guidelines. Eligible women who were interested in PrEP were offered PrEP at the same visit.

**Results: ** Overall, we conducted 493 assessments among FP clients for behavioral risk factors and willingness to consider PrEP. Among all women, median age was 25 years (IQR 21 to 29); 78% were married and 39% did not know their male partner's HIV status. The most frequently used FP methods were injectables (53%), implants (32%) and oral contraception (7%); 4% used intrauterine devices (IUDs), 4% used condoms alone and 3% used other methods. Overall, 110 (22%) of encounters led to PrEP initiation. Frequency of PrEP initiation differed by male partner HIV status (HIV‐negative 11%, unknown 30%, HIV‐positive 82%, *p* < 0.001). Younger women were as likely to initiate PrEP as older women (< 24 vs. ≥24 years, OR = 1.53, 95% CI 0.93 to 2.51, *p* = 0.09). Initiating PrEP was associated with having an STI (OR = 5.33, 95% CI 1.28 to 22.09, *p* = 0.021) and experiencing intimate partner violence in the last 6 months (OR = 3.17, 95% CI 1.27 to 7.88, *p* = 0.01). Likelihood of PrEP initiation did not differ between women using a non‐barrier FP method (hormonal contraception or IUDs) and women using condoms alone (OR = 2.02, 95% CI 0.43 to 9.48, *p* = 0.37). Frequently reported reasons for declining PrEP included the perception that HIV risk was low (30%) and the partner was HIV‐negative (30%).

**Conclusions: **Among FP clinic attendees in Kenya, PrEP initiators were more likely to have HIV risk factors and just as likely to use FP methods other than condoms as those who declined PrEP.

## TUAC0305

### Adolescent use of Truvada (FTC/TDF) for HIV pre‐exposure prophylaxis (PrEP) in the United States (2012 to 2017)

**D. Magnuson^1^; T. Hawkins^2^ and R. Mera^1^**

^1^Gilead Sciences, Epidemiology, Foster City, United States, ^2^Gilead Sciences, Medical Affairs, Foster City, United States

**Background:** Truvada (FTC/TDF) for PrEP has been approved for use in adults (≥18 years of age) at high risk of sexually acquired HIV infection in the US, EU, and other countries. Regulatory reviews are ongoing to expand the indication to include adolescents. There are limited data on the efficacy of FTC/TDF for PrEP in adolescents and there are no data on drug utilization in US or EU. This study describes FTC/TDF for PrEP use in adolescents in US from January 2012 through September 2017.

**Methods:** We used a nationally representative sample of de‐identified data from a US prescription claims database to quantify the number of unique individuals who received a FTC/TDF for PrEP prescription, representing >80% of retail pharmacies in the US. Data included medical claims, diagnosis, diagnostic procedures and both patient and provider demographics. A validated algorithm was used to exclude FTC/TDF for non‐PrEP use (e.g. chronic HIV treatment, post‐exposure prophylaxis and chronic Hepatitis B treatment).

**Results: ** From January 2012 to September 2017, 148,147 unique individuals began FTC/TDF for PrEP: 2388 (1.6%) were 12 to 17 yrs; 20,409 (13.8%) were 18 to 24 yrs; 114,279 (77.1%) were 25 to 54, and 11,071 (7.5%) were ≥ 55 yrs. The total number of adolescents increased annually from 266 in 2012 to 805 in 2015, but decreased to 216 in 2016. Females given FTC/TDF for PrEP accounted for 86.0% of adolescents while comprising only 18.4% of adults given prescriptions (*p < *0.0001). Medicaid provided coverage for FTC/TDF in 59.1% of adolescents as compared to 13.5% of adults. Adolescents received FTC/TDF for PrEP prescriptions most commonly from pediatricians (31.6%), emergency medicine (22.6%), and family medicine physicians (12.3%).

**Conclusions: **While adolescent use of FTC/TDF for PrEP is not approved in the US, 1.6% of FTC/TDF PrEP users were < 18 years of age. In contrast to adults on FTC/TDF for PrEP, adolescents on FTC/TDF for PrEP were predominantly female and commonly used Medicaid coverage. Pediatricians and emergency medicine doctors were most common prescribers in adolescents. Adolescents and young adults are disproportionately affected by HIV and efforts to increase access to PrEP for adolescents at high risk are important, including expanding the indication.

## TUAD0101

### Study on drug consumption rooms on current practice and future capacity to address communicable diseases like HCV

**E. Schatz^1^ and V. Belackova^2^**

^1^De Regenboog Groep, Correlation Network, Amsterdam, Netherlands, ^2^Uniting MSCI, Sydney, Australia

**Background:** Drug consumption rooms (DCRs) target the most vulnerable people who inject drugs (PWID). While decreases in risky injecting behaviours are an outcome of DCR use, specific HCV prevention & treatment in these settings haven't been described. There are no international DCR standards for HCV practice and research is yet to address sero‐prevalence status of DCR clients and their access to HCV prevention, treatment or supportive services.

**Methods:** An online survey providing a “snapshot” of DCR clients’ HCV status, their access to HCV services and the needs in expanding these was conducted in 2016. 49 of the 91 DCRs from Australia, Canada, Denmark, France, Germany, Luxembourg, Netherlands, Norway, Spain and Switzerland participated in the survey (54%). Each country where a DCR operates was represented.

**Results: ** An estimate of clients ration that were tested for HCV (mean = 72%) and that were HCV positive (mean = 60%) was provided by 39 DCRs. Most DCRs provided HCV testing onsite (n = 30) via blood samples (n = 19) or finger prick/saliva (n = 10). Also, several DCRs referred clients offsite for testing (n = 23).

Only three European DRCs provided HCV treatment onsite; one providing DAAs. Several offered disease self‐management support (n = 21) or monitoring of liver health (n = 10). Overall, HCV support (n = 41) as well as new treatment (n = 42) or interferon (n = 24) were available to DCR clients offsite, and the majority of DCRs referred their clients into that treatment (n = 35).

To provide further HCV‐related services, DCRs would need more staff time (n = 23) and training (n = 21), expanded staff qualifications (n = 13) and funding for equipment and services (n = 18). A change in national HCV treatment guidelines for active drug users was also identified as a need (n = 11).

**Conclusions: **DCR involvement in HCV prevention and treatment is crucial. With access to most marginalised populations, DCRs staff need to be supported to provide an entry point to HCV treatment.

## TUAD0102

### You don′t feel freedom inside: causes and factors influencing the adherence to the substitution therapy program in Khujand, Tajikistan

**N. Ilhomjon^1^ and A. Sarang^2^**

^1^NGO Khujand, Khujand, Tajikistan, ^2^SKOSH, Amsterdam, Netherlands

**Background:** The substitution therapy program in Khujand (Tajikistan) started in 2011 with the support of UNDP/GFATM. 345 PWID passed through the program, however, during six years 283 patients (82%) have fallen out. Currently, 62 clients are enrolled, or 6% or PWID registered in the region ‐ coverage far below the targets recommended by WHO for universal access to HIV prevention, treatment and care for PWID. Low coverage and high drop out rate undermine the program effectiveness in preventing HIV. Our qualitative study aimed to identify the factors that influence adherence.

**Methods:** In 2017 we carried out in‐depth interviews with twelve men and six women aged between 34 and 46 who used to participate in the program (15) or are current clients (3). We also interviewed two doctors.

**Results: ** Analysis revealed the factors that affect low retention:

1) The discrepancy between expectations and reality: many participants said they expected the program to be a short‐term intervention that would fully rid them of dependency.

2) Neglect: respondents felt the program failed to consider their needs and fully excluded them from decision making regarding own treatment, in particular concerning the dosage, their psychological needs were not addressed.

3) Stringent rules and insufficient information. Often patients signed their contract without understanding the terms and regulations.

4) Loss of control. The patients are required to visit OST daily which impedes resocialization and employment. Many withdrew from the program as they felt lack of personal freedom and control. In general, people felt disempowered by the program even more than by their previous drug addiction and wanted the exit to regain control over their lives.

**Conclusions: **To make the program an empowering environment for people to regain control over their lives, not visa versa we need to:

Ensure more patient involvement in decision making regarding the overall program design and individual treatment plans;

● Allow take‐home methadone, subject to individual conditions;

● Make methadone available at pharmacies, hospitals and penal institutions;

● Include patient representatives in the program governance and staff;

● Give more attention to patient expectations before admission and ensure more psychosocial support in achieving their goals.

## TUAD0103

### Increased methadone dose reduces illicit drug injection among HIV negative methadone clients in Myanmar

**S. Tun^1,2^; V. Balasingam Kasinather^2^ and D. Singh Mahinder Singh^2^**

^1^Myanmar Medical Association, Yangon, Myanmar, ^2^University Sains Malaysia, University of Science, Centre for Drug Research, Penang, Malaysia

**Background:** HIV prevalence rate among People who inject drugs (PWID) in Myanmar is high at 28.5%. The transmission of HIV can be reduced by reducing unsafe needle sharing among injecting drug uses and Opioid substitution. National Drug Abuse Prevention and Control Programme in Myanmar has increased financial and programmatic support for Methadone Maintenance Therapy as an Opioid Substitution Therapy (OST), paralleling with Needle Syringes Exchange Programme (NSEP). Out of 83,000 estimated people who inject drugs, more than 12,550(15%) had taken methadone for Opioid substitution in 2017. Evaluation of methadone programme is vital for the efficient service delivery.

**Methods:** A cross‐sectional study was conducted in five cities with stratified random sampling from all State/ Regions of Myanmar, where methadone services delivered. A sample of 42 persons from each site with minimum 6‐month duration on methadone, total 210 respondents were recruited to answer survey questionnaires relating to methadone profile, drug use history for 30 days and urine sample collection for methadone and illicit drug use detection (Morphine, Cannabis, Methamphetamine, Amphetamine, Benzodiazepam).

**Results: ** Findings showed that 93 (45%) didn't inject within 30 days and 116 (55.5%) admitted that they injected heroin. Those respondents had average methadone dose of 83 mg ranging from 20 to 300 mg and reported HIV is 74 out of 200, 37% of who answered. An increase in methadone dose proved that reduced in the reported drug injection within 30 days especially among HIV negative respondents (*p* = 0.00) in Cox regress analysis. This finding is also consistent with less use of Morphine in the urine samples (*p* = 0.034) and less use of Cannabis in the urine findings (*p* = 0.032). However, there is association of methadone dose and Benzodiazepam in the urine (*p* = 0.014).

**Conclusions: **High dose of methadone maintenance therapy more than 80 mg is crucial in reducing of illicit drug injection among HIV negative individuals which can subsequently reduce the unsafe practice of sharing needle among injecting drug uses. The increased methadone dose can further prevent HIV transmission among people who inject drugs. This methadone evaluation finding from Myanmar will be useful for other similar settings where methadone is used for Opioid substitution worldwide.

## TUAD0104

### Reducing harm caused by drugs: HIV prevention among people who inject drugs in South Africa

**E.M. Sibanda**

Right to Care, Global Fund, Pretoria, South Africa

**Background:** The overall aim of the Global Fund (GF) prevention programme is to reduce the number of new HIV infections and further spread of HIV among people who inject drugs (PWID). This was necessitated by rapid escalation of injection drug use which coincided with synergistic rise in HIV prevalence. A 2013, UNODC study in major metropolitan cities to assess HIV prevalence and risk practices among PWID found that 1 in 6 (14%) PWID where HIV infected. National prevalence rate is estimated at 19%. When comparing PWID to people in the general population from similar socio‐economic contexts (using race as a proxy), the HIV burden among PWID is between two and ten times higher, (Scheibe et al 2014).

**Description:** The programme is guided by WHO comprehensive package of services for PWID and utilises combination prevention methods that include behavioural, biomedical and structural interventions. Peer outreach workers act as a link to biomedical services and conduct community awareness around drug use. Key interventions include HIV Testing Services (HTS), Antiretroviral therapy (ART), Opioid Substitution Therapy (OST), and Needle and syringe programme (NSP). Of the WHO comprehensive package the GF programme doesn't support diagnosis and treatment of Hepatitis C virus (HCV), a common occurrence among PWID due sharing of drug paraphernalia. A study by Semugona et al 2017 showed a 27% HCV positivity rate among men who have sex with men and injecting drugs in Cape Town. The programme is implemented in 4 cities, namely, Durban, Cape Town, Port Elizabeth and Johannesburg by Right to Care partners, Anova and TB HIV Care Association under Global Fund.

**Lessons learned:** Attainment of results was necessitated by stakeholder engagement in planning, community mapping, and implementation. From April 2016 to date the programme achieved the following:

**Abstract TUAD0104‐Table 1Programme Results**

**Table:** 

Intervention	Output	Comment
PWID reached with comprehensive prevention package	2349	Harm reduction messaging, information, education and communication
PWID tested for HIV and know their status	2410	Positives referred for onward management
PWID HIV+	449	Denotes 19% positivity rate
Needles and syringes distributed	542,634	To minimise sharing and re‐use of needles
PWID initiated on Opioid Substitution Therapy (OST)	63	17 lost to follow up denoting about 30% ‐ in line with global trends

● HIV Positivity rate is in line with previous studies results.

● Use of peer outreach workers from target population helps in mapping areas and roll‐out of interventions.

● Need to include Hepatitis C in programmes for PWID as co‐infection is common

**Conclusions/Next steps:** Results show increased access to HIV prevention services and early enrolment in HIV care and treatment including medically assisted therapy (OST).

## TUAD0105

### Combatting the HIV epidemic among people who inject drugs in ground zero of the war on drugs ‐ the Afghan experience

**A.R. Rejaey**

Bridge Hope and Health Organisation, Programme Director, Kabul, Afghanistan

**Background:** Afghanistan is at a cross‐roads; the country is emerging from more than twenty years of political and social unrest as the leading global producer of opium in a geographic region widely affected by drugs and HIV. The number of drug users in Afghanistan is growing and currently is estimated at between 1.3 and 1.6 million. Drug treatment capacity in Afghanistan still covers only 7.8 per cent of opium and heroin users. The community consultation identified the rapid increase in the use of methamphetamine and overall drug‐related risks being compounded by low knowledge levels.

**Description:** Bridge was formed by people who use drugs in 2014, and over time PLHIV and other key populations have mobilised together under the Bridge banner.

Bridge's team of peer advocates and peer educators have skilled up to undertake a number of different activities including:

(1) Peer research

(2) Peer education

(3) Wound care management

(4) Outreach

(5) Multi and social media advocacy

(6) High level advocacy

All this has been set against working in a very high risk context where people who use drugs face daily beatings, extortion, heavy criminalisation and the threat of being rounded up and forced in to a compulsory detox centre. The drug scene is also extremely volatile and only Bridge is prepared to undertake outreach in the midst of the open drug scenes.

**Lessons learned:** Peer workers can take harm reduction commodities and life saving settings where traditional harm reduction and HIV NGOs fear to tread.

Bridge has the ability to identify and respond to changing drug trends with creativity and applying international best practice

Bridge is able to identify failings in service provision from traditional providers including highlighting corruption, poor quality services and human rights abuses.

Bridge has been unable to secure sustainable funding as an HIV representative organisation or as a harm reduction provider despite repeatedly demonstrating its unique ability and capacity.

**Conclusions/Next steps:** There is an urgent need for international donors to step up and support Bridge so it can realise its potential and support the urgent scale up in the quality and coverage of the HIV response with PWID.

## TUAD0201

### The new AIDS denialism: How criminal courts’ dismissal of modern science perpetuates HIV stigma, discrimination and criminalisation

**E.J. Bernard and S. Cameron**

HIV Justice Network, Brighton, United Kingdom

**Background:** People living with HIV continue to be charged with HIV‐related offences, often in cases where the likelihood of transmission is remote or transmission is not possible.

**Methods:** The HIV Justice Network undertook a global audit of HIV‐related arrests, investigations, prosecutions and convictions on behalf of HIV Justice Worldwide (October 2015 to September 2017: with final data to April 2018 to be presented).

**Results: ** More than 200 prosecutions were identified. The vast majority related to a perceived risk of HIV acquisition associated with sexual activity, with a minority relating to biting, spitting and breastfeeding. Convictions were common, usually resulting in incarceration, including in cases where no harm was intended, where HIV transmission did not occur, and/or where HIV transmission was extremely unlikely or not possible. Often courts doubted or ignored contemporary scientific evidence of the preventative effectiveness of condoms, antiretroviral treatment and low viral load. (Non‐transmission) cases include: incarceration for a single instance of breastfeeding; incarceration and sex offender registration for consensual sex with a low viral load; and denial of an appeal against a 30‐year sentence for consensual sex, using a condom while having a low viral load. Disaggregated data showed gendered and heteronormative values strongly influence prosecutions, as do issues of race and social marginalisation.

**Conclusions: **Courts’ dismissal of scientific evidence and corruption of legal principles has resulted in overt miscarriages of justice and the perpetuation of HIV‐related stigma. Not only have individuals received long jail terms where HIV exposure was not possible, the ongoing application of HIV exceptionalism in courts of law undermines the human rights of all people living with HIV. Despite remarkable scientific advancements, many people living with HIV remain vulnerable to the risk of unjust prosecutions because up‐to‐date science on HIV‐related risks and harm has not been effectively and/or consistently recognised in criminal law and associated policy. This issue requires the development of new strategies including greater involvement of HIV‐expert scientists in structural interventions, and training and support of expert witnesses, defence lawyers and judges in their analysis of modern HIV science.

## TUAD0202

### Decriminalizing HIV: how people living with HIV translated quantitative research into community action and legislative transformation

**H. Turk^1,2^; A. Ochoa^3^ and A. Hasenbush^4^**

^1^L.A. HIV Law & Policy Project, Los Angeles, United States, ^2^UCLA School of Law, Los Angeles, United States, ^3^UCLA, Luskin School of Public Affairs, Los Angeles, United States, ^4^UCLA School of Law, Williams Institute, Los Angeles, United States

**Background:** On 1 January 2018, four HIV‐specific criminal statutes were repealed or modified, making California the third state in the U.S. to transform and reduce the harsh impact of criminal laws targeting people living with HIV. This victory hinged on the strategic dissemination by PLWH of quantitative research and their role in effectively communicating the disparate impact of HIV criminalization on sex workers, women, and people of color.

**Description:** Beginning in 2015, The Williams Institute and the California HIV/AIDS Policy Research Center issued findings from California statewide data on the use of criminal laws targeting PLWH. The Los Angeles HIV Law and Policy Project and their collaborative partners trained and engaged PLWH to present these data. Working in partnership with academic researchers, HIV‐positive activists translated quantitative figures into compelling evidence demonstrating that HIV‐specific laws were disparately enforced against HIV‐positive women and people of color, especially those engaged in commercial sex work. At every step throughout the movement, the experiences of PLWH were centered not just in the data but also, and most importantly, in the voices of the HIV‐positive leaders who shaped the movement.

**Lessons learned:** An important best practice underscored by this successful decriminalization is the meaningful involvement of and engagement with PLWH. Throughout the multi‐year campaign, HIV‐positive people of color, women, and current and former sex workers were authentically engaged in the work, thereby providing a uniquely qualitative aspect to an otherwise quantitative presentation of facts and figures. And as a direct result of their engagement throughout this movement, and scaffolding provided to ensure their success, PLWH engaged in this work were able to further develop their individual and collective capacities to shape and direct the future of HIV‐specific policies.

**Conclusions/Next steps:** HIV criminalization remains the norm across the United States and in many parts of the world. Where it is safe to do so, PLWH are uniquely positioned to lead and facilitate transformative conversations with community members as well as lawmakers. Beyond the decriminalization movement, the California example shows that people living with HIV are best positioned to reform policies and laws that impact them most.

## TUAD0203

### Step by step: ending unjust HIV criminalization in Canada through community advocacy based on science and rights

**R. Elliott^1,2^; N. Caivano^1,2^; C. Kazatchkine^1,2^; A. McClelland^2,3^; C. Clarke^4^; L. Pelletier‐Marcotte^5,6^; N. Self^7,8^ and V. Nicholson^7,8^**

^1^Canadian HIV/AIDS Legal Network, Toronto, Canada, ^2^Canadian Coalition to Reform HIV Criminalization, Toronto, Canada, ^3^Concordia University, Montreal, Canada, ^4^Canadian Coalition to Reform HIV Criminalization, London, Canada, ^5^Coalition des Organismes Communautaires Québécois de lutte contre le Sida, Montreal, Canada, ^6^Canadian Coalition to Reform HIV Criminalization, Montreal, Canada, ^7^Positive Living Society of British Columbia, Vancouver, Canada, ^8^Canadian Coalition to Reform HIV Criminalization, Vancouver, Canada

**Background:** Canada has the third‐largest number of recorded criminal prosecutions for alleged HIV non‐disclosure in the world. There is no HIV‐specific criminal law. *Aggravated sexual assault* is the charge most commonly laid; conviction carries a maximum penalty of life imprisonment and mandatory registration as a sex offender. In cases where no transmission has occurred, people have been convicted even when they used a condom or had an undetectable viral load, or solely on the basis of oral sex.

**Description:** Canadian advocates have long pursued multiple strategies to combat unjust prosecutions, including court interventions, lobbying for prosecutorial guidelines, mobilizing scientific experts, and building feminist critiques of HIV criminalization. In 2015, the country‐wide Canadian Coalition to Reform HIV Criminalization (CCRHC, http://www.HIVcriminalization.ca) was created, including advocates with lived experience of HIV criminalization. In May 2017, CCRHC organized a think tank to discuss pros and cons of *Criminal Code* amendments as a strategy in a legal context of no HIV‐specific law and the wide interpretation of sexual assault law. CCRHC developed a Community Consensus Statement on HIV criminalization through a countrywide consultation process. Endorsed by over 150 organizations, the statement was released shortly before World AIDS Day 2017.

**Lessons learned:** Engaging in multiple strategies, adapting advocacy to the changing political landscape and involving people with lived experience are essential. On 1 December 2017, the Ontario Attorney General issued a directive to prevent prosecutions in that province in cases where a person accused of non‐disclosure had a “suppressed viral load” (< 200 copies/ml) for six months. The federal Justice Minister issued a report going further, recommending the criminal law should also not apply when people are on ARV treatment, use condoms, or engage only in oral sex (unless aware of other risk factors present).

**Conclusions/Next steps:** Although significant, these steps are insufficient to end unjust criminalization in Canada. Advocates must continue to seek concrete actions from both levels of government to implement the Community Consensus Statement recommendations. These include: amending the *Criminal Code* to remove or limit the use of sexual assault charges; better prosecutorial guidelines; and training for judges and other actors in the criminal justice system.

## TUAD0204

### Marginalized women living with HIV at increased risk of viral load suppression failure: implications for prosecutorial guidelines regarding criminalization of HIV non‐disclosure in Canada and globally

**A. Krüsi^1,2^; K. Deering^1,2^; F. Ranville^1^; L. Gurney^1^; M. Braschel^1^; B. Simpson^1^; M. Kestler^3^; K. Shannon^1,2^ and SHAWNA Project Team**

^1^Gender and Sexual Health Initiative, Vancouver, Canada, ^2^University of British Columbia, Faculty of Medicine, Vancouver, Canada, ^3^Oak Tree Clinic, BC Women's Hospital, Vancouver, Canada

**Background:** The criminalization of HIV non‐disclosure continues to supersede human rights and public health imperatives in many settings globally. Canada stands out in its assertive approach to criminalizing HIV non‐disclosure, despite recent recommendation by the Federal Government to limit prosecution of HIV non‐disclosure cases. In some jurisdictions, such as Ontario, Canada, prosecutorial guidelines limiting prosecution to people living with HIV who do not have a sustained suppressed viral load for six months have been implemented. We examined factors correlated with not meeting this legal test of achieving sustained viral suppression for 6 months among women living with HIV (WLWH) in Vancouver, Canada.

**Methods:** Prospective data (2010 to 2016) were drawn from SHAWNA (Sexual health and HIV/AIDS: Women′s Longitudinal Needs Assessment). SHAWNA is a community‐based participatory open cohort study with WLWH (cis and trans women) who access HIV services in Metro Vancouver; the cohort includes data linkages to comprehensive HIV clinical monitoring. Participants completed semi‐annual interviewer administered questionnaires and visits with a study nurse. Multivariable logistic regression using generalized estimating equations (GEE) was used to prospectively model correlates of viral load suppression failure over a seven‐year period.

**Results: ** Of 277 WLWH (Median age 43) 61% would not meet the legal test of achieving sustained viral suppression for at least one six‐month period over the seven year follow up. Over half of participants (58%) identified as Indigenous, 33% identified as White and 9% identified as African/Caribbean/Black. In multivariable GEE analyses, younger age (AOR: 0.97 per year older, 95% CI: 0.95 to 0.99), recent homelessness (AOR: 1.89, 95% CI: 1.30 to 2.75), recent sex work (AOR: 1.88, 95% CI: 1.38 to 2.55) and recent incarceration (AOR: 2.17, 95% CI: 1.15 to 4.10) were correlated with increased odds of viral load suppression failure.

**Conclusions: **These findings highlight that approaches exempting people from criminal liability for HIV non‐disclosure, conditional on maintaining an undetectable viral load for 6 months, continue to put a large proportion of WLWH in Canada at risk for criminal prosecution. Those at highest risk of prosecution are the most marginalized WLWH, including those who are younger, unstably housed, sex workers and those who have been recently incarcerated. Further action to limit the criminalization of HIV non‐disclosure is urgently needed.

## TUAD0205

### How punitive laws have encouraged human rights violations and increased HIV/AIDS transmission among gays and other men who have sex with men in Nigeria

**C. Chiaha**

International Centre for Advocacy on Right to Health, Human Rights Unit, Abuja, Nigeria

**Background:** HIV infection has been on the increase among gays and other men who have sex with men (MSM) in Nigeria where same sex relationships are criminalised. Gays and MSM encounter legal barriers to access healthcare due to discrimination in healthcare facilities forcing many of them to go underground to save their faces, resulting in the spread of HIV/AIDS and STIs. Again, organisations rendering services to this vulnerable groups reduced there publicity and stopped having group meetings but instead engaged in interpersonal one‐on‐one meetings with the target group for fear of being prosecuted under the Same Sex Marriage Prohibition Act (SSMPA) 2013, which prohibits same sex relationships including civil union/partnerships and even criminalises activities of organisations rendering any form of services to same sex persons in Nigeria, as provided in sections 4 and 5 of the Act.

**Methods:** The international Centre for Advocacy on Right to Health (ICARH) carried out documentation of cases of Human rights violations of LGBTI persons in ABUJA Nigeria from March 2011 till December 2017. ICARH also recorded data of Gays and MSM clients’ inflow in its clinic within the same period. A total of 160 of these clients were interviewed at the entry phase using a structured questionnaire. These data were analysed using both descriptive and inferential methods to arrive at the results shown below.

**Results: ** ICARH recorded 160 cases of human rights violation from 2011 till date. These are: denial of medical services, forced HIV test, disclosure of HIV status, wrongful termination of employment arbitrary arrest. Of the 160 cases, 18 occurred between 2011 and 2012 while the most occurred between 2013 to 2017 when SSMPA was passed and signed into law. Analysis shows that 90% of the clients experienced denial of medical services in public healthcare facilities. New HIV infections among gays and MSM increased after the passage of the law. Clinical data from 2011 to 2012 shows only 30% of the clients tested positive while from 2013 to 2017 80% tested positive.

**Conclusions: **Need for high level stakeholders advocacy on the effect of discriminatory laws against sexual minorities in the fight against HIV/AIDS.

## TUAD0301

### How loss of PEPFAR support for outreach puts the 90‐90‐90 targets at risk: results from a mixed methods evaluation in Kenya and Uganda

**M. Qiu^1^; L. Paina^2^; D. Rodriguez^2^; J. Wilhelm^2^; H. Zakumumpa^3^; C. Mackenzie^4^; F. Ssengooba^3^; E. Eze‐Ajoku^2^ and S. Bennett^2^**

^1^Johns Hopkins Bloomberg School of Public Health, International Health, Washington, United States, ^2^Johns Hopkins Bloomberg School of Public Health, International Health, Baltimore, United States, ^3^Makerere University, School of Public Health, Kampala, Uganda, ^4^Ipsos Kenya, Nairobi, Kenya

**Background:** PEPFAR recently implemented Geographic Prioritization (GP), where support for HIV services was prioritized according to disease burden at sub‐national level. Central support (CS) units, due to lowest burden and prevalence, were intended to transition to government support. We share findings from a mixed methods evaluation of the GP in CS facilities in Kenya and Uganda, focusing on the implications for outreach and the 90‐90‐90 targets.

**Methods:** Our study draws from national level stakeholder interviews, longitudinal, in‐depth case studies, and a cross‐sectional facility survey. Qualitative respondents included government officials, facility staff, district/county health staff, implementing partners, and patients. Qualitative data were analyzed in Atlas.ti. Facility surveys were conducted in 230 facilities in Kenya and 262 facilities in Uganda. Each country's survey data were analyzed in Stata to compare facilities that report transition from PEPFAR support to those maintained.

**Results: ** Findings indicate that outreach‐related services in CS facilities in both Kenya and Uganda largely ceased due to loss of PEPFAR support at a high proportion of affected facilities. In Uganda, survey results found that the odds of discontinuing outreach was 26.3 times higher in CS facilities than in Maintenance. In Kenya, 39% of CS facilities and 36% of Maintenance reported discontinuing outreach services. Case study respondents in both countries frequently discussed loss of support for conducting community‐based HIV testing (first 90), loss of support for informal health workers to link new patients into care (first 90), and support for tracing defaulters (third 90). Respondents described various avenues of adapting, including piggybacking HIV‐related outreach onto other funded outreach activities such as immunization, while noting that government support has not been forthcoming.

**Conclusions: **Early results indicate that loss of PEPFAR support for outreach‐related services may be hindering efforts across the treatment cascade in CS regions in both Kenya and Uganda. Despite low prevalence and burden of HIV, decreased outreach may compound local contextual factors such as poor infrastructure and high stigma that limit access to care. Decreasing outreach across facilities raises concern for countries’ ability to reach their 90‐90‐90 goals as health facilities’ links into communities are lost.

## TUAD0302

### What shall be done if donor funding to fight HIV drastically decreases. Transition to sustainable funding of social care services for PLHIV through regional budgets in Ukraine

**I. Kovalchuk**

All‐Ukrainian Network of PLWH, Advocacy, Kyiv, Ukraine

**Background:** From 2004 to 2015, social care services for over 60,000 people living with HIV (PLHIV) in Ukraine have been funded by the Global Fund to Fight AIDS, Tuberculosis and Malaria (GF) and other donors. State and regional budgets did not provide funding for social care services for PLHIV. As donor funding is projected to decrease drastically in 2020 according to the agreement between the GF and Ukrainian government, ways and mechanisms of funding of essential social services had to be developed and implemented to allow for domestic funds from state and regional budgets to substitute donor funds.

**Description:** In 2017 All‐Ukrainian Network of PLHIV has designed a portfolio of interventions aimed at creating environment for regional budgets funding of social care services for PLHIV.

Those interventions included development of regional policies to include funding for social services for PLHIV in regional targeted and integrated programs covered by regional budgets. This allowed development of mechanisms for direct purchases of social care services from regional NGOs through the system of electronic public procurement. The first group of services to benefit from this mechanism is social support for PLHIV.

**Lessons learned:** Proactive role of NGOs can ensure sustainability of vital social care services for PLHIV and implement the plan for the transition from donor funding to domestic regional funding. Direct purchasing is the optimal mechanism to be used by the government sector to procure social care services as it allows engaging NGOs.

In 2017, after new regional policies introduction, 10 regions allocated funding in amount of $60.251 in total from regional budgets for purchases of social care services for PLHIV delivered by regional NGOs.

Funding in amount of $126,423 for procurement of social care services for PLHIV in 7 regions is already allocated in 2018. Furthermore, approx. $143,857 in 8 regions are planned in 2018 as well.

**Conclusions/Next steps:** Interventions made by All‐Ukrainian Network of PLHIV allowed creating environment for transition to sustainable funding of social care services for PLHIV through regional budgets.

Final target is to cover 100% of regions of Ukraine and provide funds needed for social care services for PLHIV from state and regional budgets.

## TUAD0303

### Development of impactful advocacy arguments for domestic investments in HIV response among key population: experience from EECA region

**I. Varentsov^1^; G. Dovbakh^1^ and L. Serebryakova^2^**

^1^Eurasian Harm Reduction Association, Vilnius, Lithuania, ^2^Caucasus University, Tbilisi, Georgia

**Background:** Donors funding for HIV in low and middle‐income countries is decreasing, especially in EECA region. Governments are more likely to invest in HIV drugs procurement, than in prevention services. The following trends have been observed: funding for HIV programs was reduced, share of NGO‐based services for key populations was decreased and overall, meaningful involvement of the communities was diminished. Disruptions in services became common. Role of communities to advocate for domestic funding is vast and information about arguments that work or fail in this process is important to multiply the effect.

**Description:** Based on the systematic analysis of case studies, experiences and data accumulated as a result of regional advocacy programs Eurasian Harm Reduction Association has developed a systematic approach to arguments used for national and sub‐national advocacy. Each sub‐group of arguments has different research data requirements and can be used to plan effective advocacy campaigns for domestic investment in HIV.

**Lessons learned:** Advocacy arguments and their impact for HIV investments can be grouped as following:

(1) Services for key populations as basic human rights: this argument in EECA does not work because of low political support to human rights values.

(2) Service funding gap and other cost estimation exercises: is often requested, but rarely used for public budgeting proposes, which are constructed using historical costing models. However, availability of arguments strengthened by budget/cost figures positively impacts the image of the community and gives them access to policy discussions of HIV program budgeting.

(3) International or donor requested commitments/conditionality for domestic funding: those prove to be effective only in case of strong watchdoging from local communities.

(4) Potential cost saving impact of prevention versus treatment: there is a limited local data to strengthen arguments, and health authorities are more favourable and trustful towards funding services in healthcare facilities (often publicly owned), rather then hard‐to‐measure prevention activities.

**Conclusions/Next steps:** Regional and national advocacy for sustainable HIV funding from domestic sources lacks not only capable and trained advocates, but also a systematic approach to advocacy argument and targeted evidence‐collection.

## TUAD0304

### Challenges in implementing domestic funding policies for HIV prevention for key populations

**S. Talawat^1^; R. Rahman^2^ and P. Panitchpakdi^2^**

^1^Raks Thai Foundation, Program, Phayathai, Thailand, ^2^Raks Thai Foundation, Bangkok, Thailand

**Background:** The policy of domestic funding for HIV prevention for key populations in Thailand aims to strengthen the national AIDS strategies for ending AIDS by 2030 by using RRTTR approaches. It began in the fiscal year of 2016. The funding allocates 200 million baht per year.

**Description:** In 2016, the domestic fund was mainly allocated to health care services nationwide though it intended to engage CSOs & NGOs in the response. The fund could not be given to CSO due to the limitations of the National Health Security Act. However, with the Article 44 of the National Council for Peace and Order (NCPO), CSOs were made eligible to access this fund to implement HIV prevention services in 2017. As it is the first year that the NHSO can directly signed contact with CSOs, there were many challenges and lessons learned in establishing a well‐functioning system for the country. Through this process, all gaps and barriers were reflected to the policy makers as well as the recommendations for further improvement.

**Lessons learned:** Clear guidelines of fund management, financial regulation, and the sharing of information is very important in bridging policy to practice. Close monitoring networks with tangible collaboration of key stakeholders can shape meaningful policy.

**Conclusions/Next steps:** The challenges of 2017 serve as good guidance for establishing a mechanism for domestic funding of HIV prevention initiatives. NHSO has developed a manual for implementing and managing the HIV Prevention Fund.

## TUAD0305

### Assessing and overcoming human rights‐related barriers to HIV in 20 countries

**R. Jürgens^1^; H. Lim^1^; A. Iovita^1^; D. Burrows^2^; R. Armstrong^3^; A. Stangl^4^; S. Gruskin^5^; L. Ferguson^5^; S. Baral^6^; C. Lyons^6^; N. Poku^3^ and C. Schutte^3^**

^1^The Global Fund to Fight AIDS, TB and Malaria, Community, Rights and Gender Department, Geneva, Switzerland, ^2^APMG Health, Sydney, Australia, ^3^HEARD, Johannesburg, South Africa, ^4^ICRW, Washington DC, United States, ^5^University of Southern California, Institute for Global Health, Los Angeles, United States, ^6^Johns Hopkins University, Bloomberg School of Public Health, Baltimore, United States

**Background:** Stigma and discrimination, gender inequality and other human rights‐related barriers continue to impede access to HIV services and attainment of global HIV goals. Despite UN member states′ commitments to support programs known to reduce these barriers, the programs have nowhere been sufficiently scaled up. A five‐year initiative of the Global Fund to Fight AIDS, TB and Malaria aims to change this, first by assessing human rights‐related barriers to HIV services and then supporting and evaluating the scale‐up of evidence‐based programs to reduce those barriers.

**Description:** An extensive consultation identified 20 countries for this effort ‐ Benin, Botswana, Cameroon, Côte d'Ivoire, DR Congo, Ghana, Honduras, Indonesia, Jamaica, Kenya, Kyrgyzstan, Mozambique, Nepal, Philippines, Senegal, Sierra Leone, South Africa, Tunisia, Uganda and Ukraine. In each country, research teams assessed existing barriers to HIV services and effectiveness of programs to address them, but also what programs and interventions will be required over a five‐year period to comprehensively address barriers. Retrospective costs of existing services were estimated, as were prospective costs of the proposed comprehensive response.

**Lessons learned:** Results of the assessments confirmed that human rights‐related barriers continue to impede access to services for many. Few effective programs are being funded to address these barriers, undermining access to services and representing a serious gap in the HIV response. Even where laws and policies on the books seemingly protect key and vulnerable populations, practices by law enforcement agents and health care providers continue to subject people to discrimination and violence. There is an urgent need to significantly scale up UNAIDS’ seven key interventions to reduce stigma and discrimination and increase access to justice, which, when implemented strategically, will increase uptake of and retention in services. Substantially larger investment in these programs will be needed, but the expected benefits are great.

**Conclusions/Next steps:** Multi‐stakeholder consultations in the 20 countries are finalizing five‐year plans to scale up programs to reduce rights‐related barriers to HIV services, with intensive evaluation and costing of programs over that period. The programmatic experience of this initiative will provide unprecedented, well‐evaluated and soundly costed models for rights‐based approaches to HIV services for years to come.

## TUAD0401

### Data, stories, advocacy, leadership: the positively trans approach

**B. McBridea and C. Chung**

Transgender Law Center, Positively Trans, Oakland, United States

**Background:** In 2014, Transgender Law Center launched Positively Trans, the first program to focus specifically on increasing advocacy and empowerment by and for transgender people living with HIV, especially transgender women of color living with HIV. Through a series of dynamic and innovative approaches, Positively Trans works to increase the capacity of transgender people living with HIV to advocate for their care, their lives, and the livelihoods of their communities.

**Description:** Positively Trans works with four key points in mind: data, stories, advocacy, and leadership. For data, Positively Trans completed a national needs assessment of transgender people living with HIV in 2015, which resulted in three reports, focusing primarily on social determinants of health. Positively Trans has facilitated a series of Digital Storytelling Workshops, in which transgender people living with HIV learn the story‐telling and technical skills to create their own digital story. Data and stories are brought together in innovative ways to create new strategies by transgender people living with HIV to fight for self‐determination in health care and in policy. Positively Trans also works with a National Advisory Board of transgender people living with HIV, mostly transgender women of color living with HIV, who are each leaders in advocacy in their local communities, to build their leadership skills and capacities and provide technical assistance.

**Lessons learned:** An intersectional approach that focuses on building leaders from within specific communities, combined with evidence‐ and story‐based advocacy, is effective in organizing transgender people living with HIV organizing for their own health, well‐being, and self‐determination. Members of the National Advisory Board have started their own organizations for transgender people living with HIV and have been able to connect transgender people to the services they need and fight for better policies on a local, state, and national level.

**Conclusions/Next steps:** The conversation in the United States on HIV has been shifted by Positively Trans to include transgender people at a national level, including Positively Trans's collaboration on federal skill building projects. Next steps include exploring if Positively Trans's approaches can be replicated in other countries and regions internationally and how they would need to change based on local needs.

## TUAD0402

### Addressing the needs of people with situational gender and ′sexual orientation′ in Tajikistan by HIV services

**A. Sarang**

SKOSH, Almere, Netherlands

**Background:** The Western European/North American (WE/NA) understanding of gender and sexuality reflected in the relevant LGBTQ/MSM vocabulary has become a dominant model shaping the global response to HIV. We often neglect other understandings of gender and sexuality that differ from this dominant model. Consequently, HIV programs do not adequately address the needs of individuals outside of the LGBTQ/MSM paradigm. Anthropological research explores the understanding of gender and sexuality in Tajikistan among people with situational gender and ′sexual orientation′.

**Methods:** The study employed ethnography and historical analysis. The later was done through literature review of concepts of gender and sexuality in the Persian/Tajik tradition affected by the Russian colonization and Sovietization. The ethnographic fieldwork (2016) employed participant observations, 17 interviews and a focus group. The transcripts were coded in MaxQDA11 and analyzed thematically. The decolonial optics were applied as the theoretical framework.

**Results: ** Literature reveals rich homo‐erotic tradition in the Persian/Tajik culture and understanding of gender and sexuality based on a “gender/sexuality conflation model” (when gender is defined by the position in sex, rather than the biological sex). Unlike in the WE/NA “gay model”, an individual is not required to adhere to a solid inflexible gender and “sexual orientation” identity. People with variable gender find it difficult to decide which identity slot offered by the western vocabulary they should fit in. They are not “MSM” or “gay” as they often identify as women while having sex. They also don′t identify as “transgender women,” and in the conservative Tajik society, they prefer to stick to male social roles without manifesting their female selves. The identity problem messes up with sentinel surveillance/indicators, also based on the WE/NA vocabulary that people don't understand.

**Conclusions: **The results problematize usage of “universal identity language” in designing the HIV interventions. E.g. in some contexts, the WHO recommendation to separate services for trans* people from MSM is problematic as there is no clear divide. The new requirements/indicators may lead to aggravation of stigma and homo/transphobia. These problems should be given a better thought and be better reflected in international guidelines stressing the importance of local concepts and identity language.

## TUAD0403

### Breaking the wall: transgender people and Islamic religious leaders

**E. Kor**

Public Health and Welfare Organization, EXCO, Kuala Lumpur, Malaysia

**Background:** Malaysia is a predominantly Muslim majority country, where Islam has an influential role in society, policies and law. Transgender people in Malaysia are often stigmatised and discriminated against; in the past this has been exacerbated by words and actions of some Islamic religious leaders. This creates distrust and tension between the transgender community and religious leaders, both of which are stakeholders in HIV prevention. For many transgender people in Malaysia, faith acts as an important coping mechanism and being excluded from their religion adds a further layer of isolation, which need not be the case.

The purpose of this project is to minimise the schism between Islamic religious leaders and Muslim transgender people in Kuala Lumpur, engage in dialogue to rehumanise transgender people.

**Description:** This was a grassroots unfunded project undertaken by PKKUM, a local NGO based in Kuala Lumpur, Malaysia. The initial engagement was with top level Islamic religious leaders via a written petition submitted to the Mufti, consisting of over 100 signatories from the transgender community. A pilot set of workshops were undertaken with these religious leaders and members of the transgender community, female sex workers, PLHIV and MSM comprising of more than 100 participants. This provided a safe space where all parties could share experiences, discuss areas of concern and engage in meaningful dialogue.

**Lessons learned:** There was a genuine willingness of all groups to engage with one another. A pivotal moment was when the Mufti personally apologised for the manner in which transgender people were treated in the past, a key step in bridge‐building. There was an identification of the disparity in language terminology used and assurance that transgender people would be welcomed n the mosque.

One outcome of the project was that the Mufti subsequently gave a Friday sermon (khutbahs) to congregants, urging worshipers not to judge or stigmatise the transgender community.

**Conclusions/Next steps:** The role of Islamic religious leaders towards the transgender community has been seen as unhelpful, however this can be transformed to a positive influence, as advocates to counteract stigma and discrimination.

## TUAD0404

### Progressive Botswana court affirms that legal recognition of gender identity is at core of human dignity – transgender persons are fully entitled to constitutional protection

**T.K. Esterhuizen**

Southern Africa Litigation Centre (SALC), LGBTI and Sex Workers Rights Programme, Johannesburg, South Africa

**Background:** For many transgender persons throughout Africa, obtaining an identity document that matches their expressed gender is virtually impossible. Without an identity document that reflects their gender, they are exposed to not only stigma and discrimination but ongoing distress when they are required to explain intimate details of their life to strangers to merely access routine services such as health care and treatment. However, the Botswana High Court recently took a significant step towards protecting the dignity of transgender people when it ordered the Registrar to change the gender marker on the identity document of a transgender man from female to male. The case also highlights advocacy and litigation strategies that LGBT activists and their lawyers can use to assert their basic rights, even within the most criminalised environments.

**Description:** The case was brought before the courts after sustained advocacy and legal strategy by brave LGBT activists, who publically sought the recognition of their constitutional and human rights. When meaningful engagement with the government of Botswana failed, a transgender man took his plight to the judiciary and has successfully sought to uphold the fundamental rights of LGBT persons throughout the country.

**Lessons learned:** The Court acknowledged that recognition of a person's gender identity constitutes the core of one's sense of being and is an integral (part) of a person's identity. Legal recognition of the gender identity is therefore part of the fundamental right to dignity and freedom to express oneself in a manner he or she feels comfortable with. The High Court emphasised that the State (and society) has a duty to respect and uphold the individual right to human dignity despite opposing and different views it might hold with regards to the applicant's gender identity.

**Conclusions/Next steps:** This legal recognition is an important step towards the realisation of the fundamental rights of transgender persons. However, much more work needs to be done to ensure the respect and recognition of the fundamental rights of transgender people in Botswana, including providing access to adequate gender‐affirming treatment and care and the implementation of non‐discriminatory healthcare, HIV policies, and employment and education policies.

## TUAD0405

### Developing and pilot testing TRANScending love, a multi‐methods arts based workshop with African, Caribbean and Black transgender women

**C.H. Logie^1^; Y. Persad^2^; T.B. Ferguson^3^; S. Ryan^3^; D.M. Yehdego^3^ and A. Guta^4^**

^1^University of Toronto, Toronto, Canada, ^2^519 Community Centre, Toronto, Canada, ^3^Black Coalition for AIDS Prevention, Toronto, Canada, ^4^University of Windsor, Windsor, Canada

**Background:** Transgender women's HIV vulnerabilities are shaped by contexts of social, economic and healthcare exclusion. In Canada, African, Caribbean and Black (ACB) women are also overrepresented in HIV infections; vulnerabilities are similarly shaped by social and structural inequities. There are a scarcity of HIV interventions developed by and for ACB transgender women in Canada who are at the nexus of ACB and transgender women's HIV disparities. We aimed to develop HIV intervention strategies with ACB transgender women in Toronto, Canada.

**Description:** This community‐based study with an ACB AIDS service organization and ACB transgender women community leaders involved a focus group with ACB transgender women (n = 8) to explore HIV prevention/care priorities. Findings were presented to a second focus group with ACB transgender community leaders (n = 4) to brainstorm next steps. Focus group findings informed the development of the TRANScending Love (T‐Love) arts‐based workshop that included: (1) hand‐held mirrors with glass markers to write messages of self‐love; (2) anatomical drawings of hearts to write/draw emotional blocks and coping strategies; and (3) blank “you are loved” affirmation cards to write messages to other ACB transgender women. We conducted 3 T‐Love workshops with 18 ACB transgender women, workshops were followed by a focus group to examine art products and processes. Focus groups were digitally recorded and transcribed verbatim, and analyzed using thematic analyses.

**Lessons learned:** The first focus group highlighted ACB transgender women′s priorities were not HIV: participants called for researchers to address transgender women′s lack of self‐love produced by social and structural exclusion, and to move away from the focus on transgender women′s sexual practices and bodies. The second focus group articulated the need for strengths and arts‐based approaches to build self‐love and acceptance. Workshop participants described T‐Love as empowering in documenting journeys to self‐acceptance, and also in building connections and solidarity with other ACB transgender women.

**Conclusions/Next steps:** Findings highlight the potential for arts‐based approaches to foster dialogue and reflection on individual and collective strengths among transgender women. We produced a “deck” of T‐Love cards and disseminated these online and in community forums. Findings can inform in‐depth, sustained arts‐based engagement to build self‐love and community with ACB transgender women.

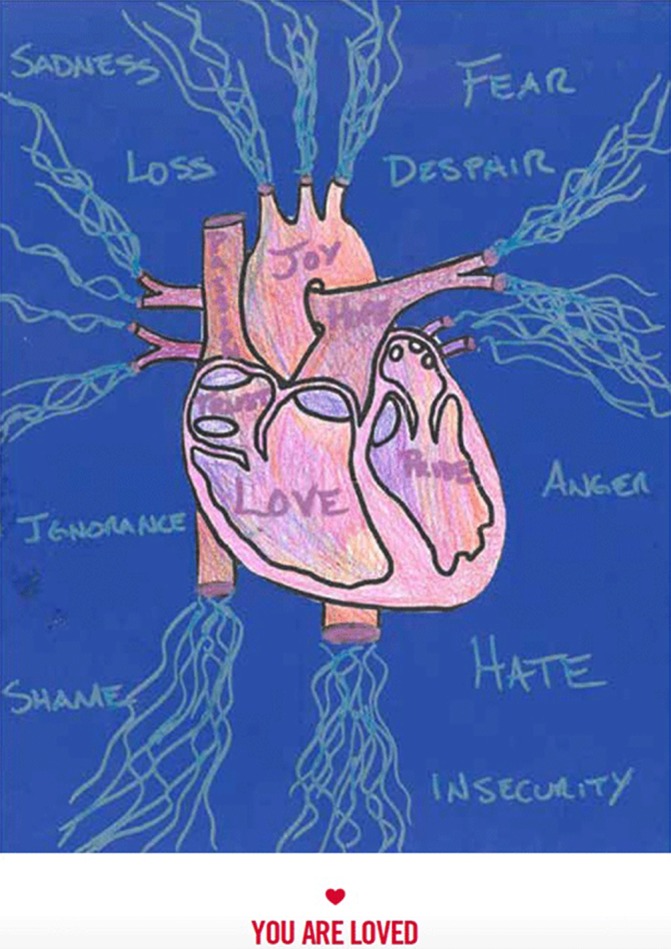

**Abstract TUAD0405‐Figure 1. TRANScending Love Arts‐Based Research with African, Caribbean and Black Trans Women.**

## TUAE0101

### The impact of performance‐based financing on the delivery of HIV testing, prevention of mother to child transmission and antiretroviral delivery in the Cameroon health system

**D. de Walque^1^; P.J. Robyn^2^; H. Saidou^2,3^; G. Sorgho^2^ and M. Steenland^4^**

^1^The World Bank, Development Research Group, Washington, United States, ^2^The World Bank, Health Nutrition and Population, Washington, United States, ^3^University of Paris Dauphine, Economics, Paris, France, ^4^Harvard University, Department of Global Health and Population, School of Public Health, Cambridge, United States

**Background:** At scale provision of key services to persons living with HIV is challenging in low resources settings. Performance‐based financing (PBF) is a complex health systems intervention aimed at improving coverage and quality of care. An impact evaluation was conducted in Cameroon seeking to isolate the role of specific components of the PBF approach on the delivery of three services at facility‐level: HIV testing, prevention of mother to child transmission and antiretroviral delivery.

**Methods:** The evaluation used a cluster random design at the health facility level to separate the different components of the PBF approach. Facilities were randomly allocated in four evaluation groups to measure the effects of each component. Facilities in group T1 implemented the full PBF package: payments linked to results (quantity and quality of services) including bonus for health workers, management autonomy, enhanced supervision and monitoring. Facilities assigned to group C1 received a fixed per capita budgetary supplement that matches the per capita budgetary allocation for T1 facilities, but was not linked to performance. C2 facilities received no additional resources but the same supervision and monitoring as T1 and C1 facilities. C3 facilities were the “business as usual” facilities and did not receive any additional resources or inputs. Facility register data documenting facility provision of HIV‐related services was collected during a baseline and endline surveys and analyzed.

**Results: ** We found large and statistically significant effects of both PBF and additional financing on HIV testing. An average of 61 more patients (*p* < 0.01) were tested for HIV in PBF facilities than control facilities, and 51 more patients ((*p* < 0.01)) were tested monthly in the additional financing arm compared to the control. There was very little change in HIV testing in the additional supervision group, and the effect of PBF was greater than the effect of additional supervision (*p* < 0.01). However, we found no impacts on PMTCT and ART provision.

**Conclusions: **PBF increased the delivery of HIV testing. A similar improvement was however measured in the additional financing group, suggesting that the complex PBF mechanism might not necessarily add value, even though the comparison between both interventions is delicate.

## TUAE0102

### Diagnosing and treating more infants, faster: findings from the first multi‐country evaluation of routine point‐of‐care early infant diagnosis in eight sub‐Saharan countries

**F. Bianchi^1^; R. Machekano^2^; J.‐F. Lemaire^3^; E. Sacks^2^; R. Bailey^3^; V. Nzima^4^; P. Fassinou^5^; A. Mataka^6^; C. Odhiambo^7^; A. Chadambuka^8^; G. Nyoni^9^; M.C. Sabonete^10^; G.F. Ndayisaba^11^ and J. Cohn^3^**

^1^Elizabeth Glaser Pediatric AIDS Foundation, Strategic Information and Evaluation, Washington, United States, ^2^Elizabeth Glaser Pediatric AIDS Foundation, Research, Washington, United States, ^3^Elizabeth Glaser Pediatric AIDS Foundation, Geneva, Switzerland, ^4^Elizabeth Glaser Pediatric AIDS Foundation, Yaounde, Cameroon, ^5^Elizabeth Glaser Pediatric AIDS Foundation, Abidjan, Cote D'Ivoire, ^6^Elizabeth Glaser Pediatric AIDS Foundation, Maseru, Lesotho, ^7^Elizabeth Glaser Pediatric AIDS Foundation, Nairobi, Kenya, ^8^Elizabeth Glaser Pediatric AIDS Foundation, Harare, Zimbabwe, ^9^Elizabeth Glaser Pediatric AIDS Foundation, Mbabane, Swaziland, ^10^Elizabeth Glaser Pediatric AIDS Foundation, Maputo, Mozambique, ^11^Elizabeth Glaser Pediatric AIDS Foundation, Kigali, Rwanda

**Background:** Point‐of‐care early infant diagnosis (POC‐EID) may improve the care of HIV‐exposed infants compared to conventional testing. POC‐EID is being implemented in eight sub‐Saharan African countries at POC (“testing”) and near‐POC (“spoke”) sites. A program evaluation was undertaken to assess the impact of POC‐ or near POC‐EID implemented as part of routine care, compared with conventional testing.

**Methods:** Using a pre‐post intervention design, key EID outcomes were compared in each country. Pre‐intervention conventional EID data were collected retrospectively from registers across a purposively sampled sub‐set of sites. Post‐intervention data for specimens processed between December 2016 and December 2017 were collected prospectively using a POC‐EID testing form. Median turnaround times (TATs) were compared using Wilcoxon rank‐sum test, and proportions with Pearson chi‐square test. Kaplan–Meier Estimator was used for the proportion of caregivers who received results within 30 days of sample collection. Prospective data were analyzed by entry point and compared between testing/hub and spoke sites. The cost per test result returned was calculated using Global Fund's total cost of ownership estimates for POC and conventional EID.

**Results: ** POC‐EID resulted in a significantly higher percentage of results returned to caregiver and percentage of infants started on treatment sooner, as compared to conventional EID (Table 1). There were no significant differences in percent results returned between testing and spoke sites (Figure 1). Valid, non‐confirmatory tests from different entry points revealed 3.2%, 19.6%, 15.5%, and 2.5% HIV‐positivity rates from prevention of mother‐to‐child transmission entry points, pediatric inpatient, outpatient, and vaccination clinics, respectively. The cost per test result returned (regardless of TAT) was $20 to 38 for POC and $21 to 33 for conventional.

**Abstract TUAE0102‐Table 1. service delivery outcomes**

**Table:** 

	Conventional EID	POC‐EID	*p*‐value
% Results received by caregiver within 30 days	21.18%	99.13%	*p* < 0.001
Median TAT from blood sample collection to result returned to caregiver	55 days (IQR: 31 to 76)	0 days (IQR: 0 to 1)	*p* < 0.001
% HIV‐infected infants started on treatment	67.33%	90.6%	*p* < 0.001
Median TAT from result returned to caregiver to ART initiation for HIV‐infected infants	0 days (IQR: 0 to 4.5)	0 days (IQR: 0 to 1)	NS
Median TAT from blood sample collection to ART initiation for HIV‐infected infants	49.5 days (IQR: 32 to 68)	0 days (IQR: 0 to 2)	*p* < 0.001

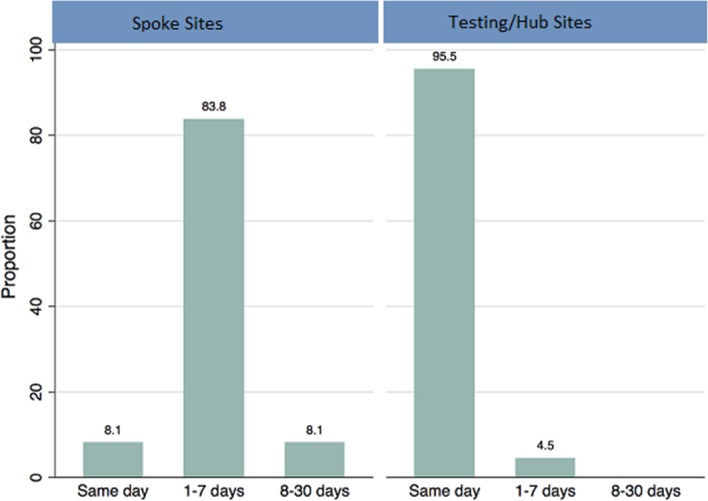

**Abstract TUAE0102‐Figure 1. Distribution of POC in testing/hub and spoke sites by median turnaround time from sample collection to results received by caregivers.**

**Conclusions: **Routine use of POC‐EID in sub‐Saharan Africa is feasible and significantly improves key patient outcomes. Spoke sites can expand access to POC‐EID, with minimal differences in patient‐level outcomes. POC‐EID is particularly important for high‐yield entry points such as pediatric wards, where patients may be less likely to receive results from conventional testing with long TATs. Given similar costs per test result returned to caregiver for conventional testing, POC‐EID represents an efficient and effective way to identify HIV‐infected infants and initiate antiretroviral treatment.

## TUAE0103

### Improving technical efficiency: reaching first 90 through community index HIV sexual network testing in Zimbabwe. The case of FHI 360 Zimbabwe

**A. Muchedzi^1^; N. Mahachi^1^; T. Moga^1^; T. Tafuma^1^; P. Mawora^1^; D. Harbick^2^; T. Nyagura^3^ and K. Reichert^3^**

^1^FHI 360, Technical, Harare, Zimbabwe, ^2^FHI 360, Harare, Zimbabwe, ^3^USAID, Harare, Zimbabwe

**Background:** Zimbabwe has made huge progress towards the UNAIDS first 90 target; 74.2% of people living with HIV know their status: with less men‐69.7% compared to women‐77.1% knowing their status. Reaching the remaining 16% and men is challenging and requires innovative approaches with technical efficiencies. FHI360 is responding with community HIV‐index‐testing; an approach which involves tracking down sexual contacts of HIV+ index‐cases identified at health facilities.

**Description:** Since October 2016, FHI360 through a strengthened community health system comprising: a team of trained nurses and community cadres working in collaboration with health facilities identifies HIV+ index‐cases. The community cadre obtains consent from the index‐cases, makes appointments and prepares the household for a visit by the nurse. The nurse provides HIV‐testing and other health services to children and primary sexual contacts of the index‐case at the household. With consent from the index‐case the nurse tracks and tests other sexual partners and the sexual network as illustrated in Figure 1.

**Lessons learned:** Between October 2016 and September 2017, 14,818 HIV+ index‐cases were identified from 330 health facilities and followed to 12,865 households for community index‐testing. In the community, 22,147 people were HIV tested, 8367 (38%) were HIV+, and 5771 (69%) were linked to care and treatment. FHI360′s community index‐testing has an average HIV yield rate 10 times more than the health facility yield rate (4%). The case in Figure 1 is one of the many sexual network testing conducted where several PLHIV are identified. In this case 7 PLHIV were identified from one index‐case. FHI360 has reached 11,754 men with HIV testing services of which 6127 (72%) were between 25 and 49 years.

**Conclusions/Next steps:** FHI360 achieves and sustains a high yield among individuals tested for HIV using the community HIV index‐testing model. This model is reaching people living with HIV who would not ordinarily access HIV testing services (HTS) from the conventional health facility HTS modalities. By going into the community, FHI360 is identifying a critical group of people who are living with HIV and facilitating their treatment initiation. FHI360 is leveraging its community platform to reach a high proportion of men of reproductive age with HIV services.

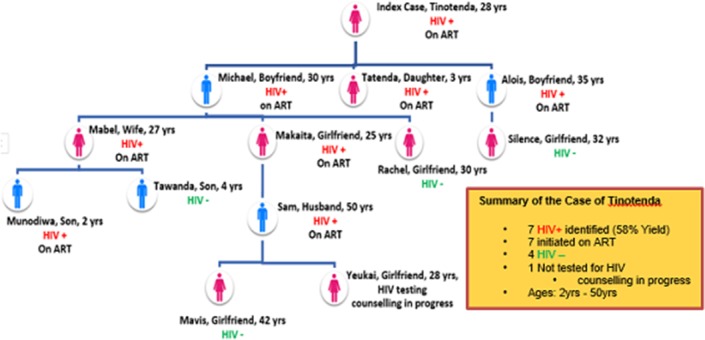

**Abstract TUAE0103 Figure 1. FHI360 Zimbabwe Sexual Network HIV Testing.**

## TUAE0104

### Cost‐of‐testing‐per‐new‐HIV‐diagnosis as a metric for monitoring cost‐effectiveness of testing programmes in low income settings in Southern Africa: health economic modelling analysis

**A. Phillips^1^; V. Cambiano^1^; L. Bansi‐Matharu^1^; F. Nakagawa^1^; D. Wilson^2^; I. Jani^3^; T. Apollo^4^; M. Sculpher^5^; T. Hallett^6^; C. Kerr^7^; J. van Oosterhout^8,9^; J. Eaton^6^; J. Estill^10^; B. Williams^11^; N. Doi^12^; F. Cowan^13,14^; O. Keiser^10^; D. Ford^1^; K. Hatzold^15^; R. Barnabas^16^; H. Ayles^17,18^; G. Meyer‐Rath^19,20^; L. Nelson^21^; C. Johnson^22^; R. Baggaley^22^; A. Fakoya^23^; A. Jahn^24^ and P. Revill^5^**

^1^UCL, London, United Kingdom, ^2^Burnet Institute, Melbourne, Australia, ^3^National Institute of Health, Maputo, Portugal, ^4^Ministry of Health and Child Care, Harare, Zimbabwe, ^5^University of York, York, United Kingdom, ^6^Imperial College London, London, United Kingdom, ^7^University of Sydney, Sydney, Australia, ^8^Dignitas International, Zomba, Malawi, ^9^College of Medicine, University of Malawi, Department of Medicine, Zomba, Malawi, ^10^University of Geneva, Geneva, Switzerland, ^11^Stellenbosch University, Stellenbosch, South Africa, ^12^CHAI, New York, United States, ^13^CeSHHAR Zimbabwe, Harare, Zimbabwe, ^14^Liverpool School of Tropical Medicine, Liverpool, United Kingdom, ^15^PSI Zimbabwe, Harare, Zimbabwe, ^16^University of Washington, Seattle, United States, ^17^London School of Hygiene & Tropical Medicine, London, United Kingdom, ^18^Zambart, Lusaka, Zambia, ^19^Boston University, Boston, United States, ^20^University of the Witwatersrand, Johannesburg, South Africa, ^21^CDC, Kampala, Uganda, ^22^WHO, Geneva, Switzerland, ^23^The Global Fund, Geneva, Switzerland, ^24^Ministry of Health, Lilongwe, Malawi

**Background:** As prevalence of undiagnosed HIV declines, it is unclear whether testing programmes will continue be cost‐effective. The cost‐of‐testing‐per‐new‐HIV‐diagnosis is a potentially useful metric for monitoring country programmes.

**Methods:** We simulated 1000 setting‐scenarios for adult HIV epidemics and ART programmes typical of southern Africa using an individual‐based model, and projected 50 years from 2018, during which a minimum package of “core” testing in pregnant women, for diagnosis of symptoms, in sex workers, and in men coming forward for circumcision was assumed to be conducted. For each setting scenario we compared this policy of core testing only with a policy of also having an additional programme of testing (in men only, women only, or both genders). For each setting scenario we randomly selected from various possible rates of testing and degrees to which those with HIV are more likely to test than those without, and considered a range of a unit costs. Our aim was to asses the relationship between the cost‐of‐testing‐per‐new‐HIV‐diagnosis and the cost‐per‐DALY averted (the incremental cost‐effectiveness ratio; ICER) of the additional testing programme. Cost‐effectiveness of the programme was defined by an ICER below US$500. Discount rate 3%/annum.

**Results: ** There was a strong relationship between the cost‐of‐testing‐per‐new‐HIV‐diagnosis and the ICER (illustrated for testing programmes in men in Table 1). In general, the ICER was below US$500 per DALY averted so long as the cost‐of‐testing‐per‐new‐HIV‐diagnosis was below US$315. Results were similar when we restricted to setting‐scenarios with specific epidemic and programmatic features, such as prevalence of undiagnosed HIV, HIV incidence and the proportion of HIV diagnosed people with viral suppression. When the testing programme was restricted to testing in women beyond the core testing this was not cost effective. However, for over 50% of setting scenarios testing programmes in men were cost‐ effective when the cost‐of‐testing‐per‐new‐HIV‐diagnosis was <US$585 (and 80% when the cost‐of‐testing‐per‐new‐HIV‐diagnosis was <US$312), regardless of unit cost of testing.

**Conclusions: **The cost‐of‐testing‐per‐new‐HIV‐diagnosis can be used to monitor the cost‐effectiveness of testing programmes. Programmes aimed at men in low income settings in southern Africa are likely to be cost‐effective if they cost below US$585 per new diagnosis.

**Abstract TUAE0104‐Table 1.**

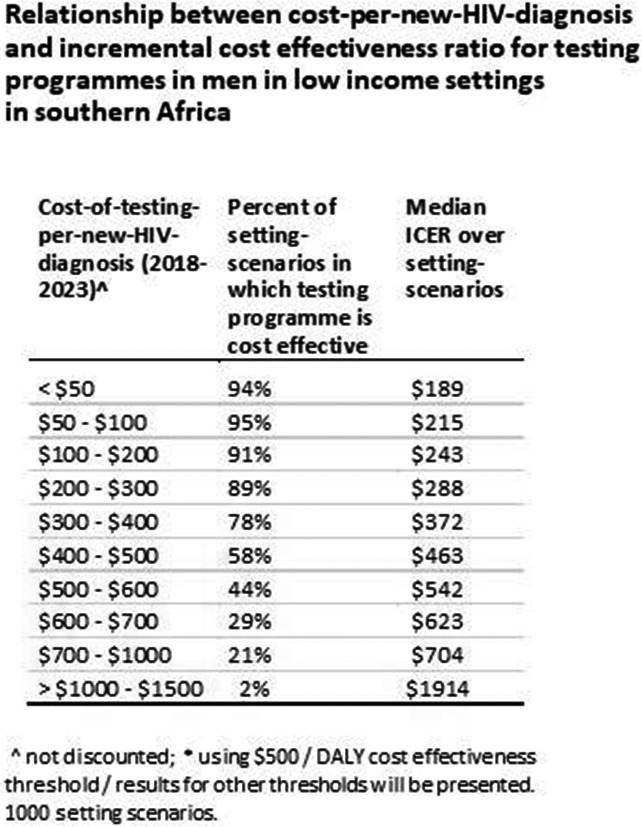

## TUAE0105

### Facility‐based HIV self‐testing for outpatients dramatically increases HIV testing in Malawi: a cluster randomized trial

**K. Dovel^1,2^; M. Nyirenda^2^; F. Shaba^2^; O.A. Offorjebe^3,4^; K. Balakaksi^2^; B.E. Nichols^5,6^; K. Phiri^2^; A. Schooley^1,2^; R.M. Hoffman^1^ and EQUIP Innovations for Health**

^1^University of California, Division of Infectious Diseases, Los Angeles, United States, ^2^Partners in Hope, Lilongwe, Malawi, ^3^David Geffen School of Medicine at UCLA, Los Angeles, United States, ^4^Charles R. Drew University of Medicine and Science, Los Angeles, United States, ^5^Boston University School of Public Health, Department of Global Health, Boston, United States, ^6^University of Witwatersrand, Health Economics and Epidemiology Research Office, Department of Internal Medicine, Johannesburg, South Africa

**Background:** HIV self‐testing (HIVST) increases testing coverage in community settings in sub‐Saharan Africa, but scalability is a challenge due to resource constraints. Outpatient departments provide an ideal space to integrate HIVST into low‐resource health systems due to high client volume and long wait‐times. We evaluated an HIVST intervention in outpatient waiting‐spaces of hospitals and health centres in Malawi.

**Methods:** A cluster randomized trial was conducted at 15 health facilities in Central/Southern Malawi between September 2017 to January 2018. Facilities were randomized 1:1:1:

(1) routine provider initiated testing and counseling (PITC);

(2) Optimized PITC (additional provider trainings and job‐aids); and

(3) HIVST (including Oraquick HIV self‐test^®^ demonstration, distribution, and kit use in outpatient waiting‐spaces, private spaces for interpretation, and optional post‐test counseling).

The primary outcome was HIV‐testing among outpatients. Exit surveys were conducted with a random sample of outpatients.

**Results: ** 5675 outpatients completed an exit survey. There were no differences by arm (Table 1). 52% of outpatients in the HIVST arm tested for HIV compared to 14% in Optimized PITC (AOR: 6.6 *p* < 0.001) and 12% in PITC (AOR: 7.6 *p* < 0.001). For HIVST, 60% of outpatients in need of testing (defined as tested >12 months ago and never tested HIV‐positive) were tested compared to 18% in Optimized PITC and 16% in PITC. There was no significant difference in the proportion of clients tested who reported previously testing HIV‐positive (≤1% for all arms). Positivity rates did not differ by arm, however, HIVST was associated with a higher absolute number of new positives identified compared to Optimized PITC (AOR: 2.9, *p* = 0.01) and PITC (AOR: 4.1, *p* = 0.002). Participants who were tested by HIVST were more likely to want to test again using the same method and more likely to recommend testing to others compared to those tested by Optimized PITC or PITC. No adverse events were reported in the HIVST arm.

**Conclusions: **Facility‐based HIVST in outpatient waiting‐spaces dramatically increased HIV testing and identification of HIV‐infected persons among outpatients in Malawi, with minimal risk for loss of confidentiality or adverse events. Analyses for linkage to care are underway. Evaluations of routine program implementation are needed to determine best strategies to take facility‐based HIVST to scale.

**Abstract TUAE0105‐Table 1. Participant characteristics and outcomes across three arms of a HIVST study targeting outpatient clients (n = 5675)**

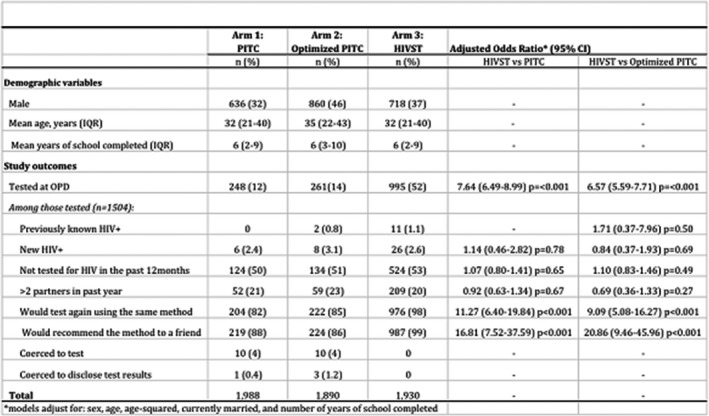

## WEAA0101

### Chidamide reactivates and diminishes latent HIV‐1 DNA in patients on suppressive antiretroviral therapy

**Y. Sun^1^; J. Li^1^; J. Ma^2^; C. Wang^1^; F. Bai^1,3^; K. Zhao^1,4^; Z. Yu^1,5^; W. Kang^6^; Y. Zhuang^1^; N. Yao^1^; Q. Liu^1^; B. Dang^1^; B. Wang^1^; Q. Wei^1^; Z. Liu^1^; L. Wang^1^; W. Kang^1^; L. Wang^1^; J. Xia^1^; T. Wang^7^ and T. Zhu^8^**

^1^Fourth Military Medical University, Xi'an, China, ^2^Xi'an Jiaotong University, Xi'an, China, ^3^323th Hospital of PLA, Xi'an, China, ^4^Xi'an Communication Institute, Xi'an, China, ^5^Political Work Department of People's Republic of China Central Military Commission, Beijing, China, ^6^Fourth Military Medical University, Department of Infectious Diseases, Tangdu Hospital, Xi'an, China, ^7^Jinan University, Institute of Life and Health Engineering, College of Life Science and Technology, Guangzhou, China, ^8^University of Washington, Department of Laboratory Medicine, Seattle, United States

**Background:** A proposed strategy to purge HIV reservoir is to reactivate provirus transcription with latency‐reversing agents (LRAs), inducing viral antigen expression and allowing immune‐mediated clearance of reservoir cells in the presence of combination antiretroviral therapy (cART). Here we evaluated the safety and efficacy of chidamide, a benzamide histone deacetylase inhibitor, in patients on suppressive cART.

**Methods:** Seven aviremic HIV‐1‐infected patients received eight oral doses of 10 mg chidamide twice a week (Tuesday/Friday) for four weeks while maintaining baseline cART. Safety was evaluated at each visit and plasma concentrations of chidamide was measured by liquid chromatography‐mass spectrometry. Histone acetylation levels in CD4^+^ T cells were analyzed by flow cytometry. Plasma HIV RNA was determined using Cobas Taqman HIV‐1 Test, v2.0. Cell‐associated HIV RNA (CA‐HIV RNA) and total HIV DNA (CA‐tHIV DNA) were quantified by the SupBio PCR test in PBMCs. Thirteen plasma biomarkers of inflammation were evaluated by luminex multiplex assays and ELISA. Changes from baseline to specific time points were compared using Wilcoxon matched‐pairs signed‐rank tests, and a two‐sided *p*‐value of less than 0.05 was considered significant.

**Results: ** All participants (6 male, 1 female) completed full chidamide dosing, and showed acceptable drug tolerance with only grade 1 adverse events presented. No drug accumulation effects were detected per chidamide dosing. In addition, the cyclic increase of histone acetylation in CD4^+^ T cells was observed. All participants showed robust and cyclic viremia (peak viremia range 147 to 3850 copies/mL) as well as increased CA‐HIV RNA (median peak increase 9.4‐fold vs. baseline, range 2.0‐fold to 34.9‐fold) during chidamide treatment. At day 56, plasma HIV RNA of all participants recovered to undetectable level. Furthermore, we discerned the significant reduction of CA‐tHIV DNA (day 27 vs. baseline, *p* = 0.018, and day 56 vs. baseline, *p* = 0.028). Equally important was that chidamide exhibited an anti‐inflammatory property as evidenced by inhibition of pro‐inflammatory cytokines: MCP‐1, MMP‐9, IP‐10, LBP, P‐selectin, and CD40 ligand.

**Conclusions: **Chidamide can safely disrupt the latency of HIV DNA resulting in the clearance of reactivated reservoirs, which makes it a promising candidate toward the eradication of HIV reservoir.

## WEAA0102

### The antiretroviral CCR5‐inhibitor maraviroc effectively reverses HIV latency by phosphorylation of Nf‐κB

**J. Symons^1^; S.F.L. vanLelyveld^2^; W. deSpiegelaere^3^; A.M.J. Wensing^4^; J.M. Zerbato^1^; P.M. vanHam^4^; J. Drylewicz^5^; A. Middel^2^; A.I.M. Hoepelman^2^; P.U. Cameron^1,6^; H.K. Lu^1^; A.I. Dantanarayana^1^; J.A.M. Borghans^5^; L. Vandekerckhove^3^; S.R. Lewin^1,6^; K. Tesselaar^5^ and M. Nijhuis^4^**

^1^The Peter Doherty Institute for Infection and Immunity, The University of Melbourne, Melbourne, Australia, ^2^University Medical Centre Utrecht, Department of Internal Medicine and Infectious Diseases, Utrecht, Netherlands, ^3^Ghent University, Department of Internal Medicine, Ghent, Belgium, ^4^University Medical Centre Utrecht, Department of Medical Microbiology, Virology, Utrecht, Netherlands, ^5^University Medical Centre Utrecht, Department of Immunology, Utrecht, Netherlands, ^6^Alfred Hospital and Monash University, Department of Infectious Diseases, Melbourne, Australia

**Background:** One strategy to eliminate latently infected cells in HIV‐infected individuals on antiretroviral therapy (ART) is to induce HIV transcription with latency reversing agents (LRAs). Potent non‐toxic LRAs are urgently warranted. The clinically approved CCR5‐inhibitor maraviroc (MVC) demonstrated an increase in HIV transcription *in vitro* and may therefore act as an LRA. We investigated the effect of MVC on HIV transcription and its mechanism of action *in vitro*,* ex vivo* and *in vivo* during a MVC‐intensification trial.

**Methods:** Activated PBMCs were infected with HXB2 (MOI 0.01) and cultured with MVC. Seven days post‐infection p24 was measured in supernatant by ELISA (n = 9). Changes in NF‐κB phosphorylation were assessed by densitometry Western‐Blot (n = 2). 5 million resting CD4 T‐cells were isolated from HIV infected individuals on ART, treated with MVC or vorinostat and unspliced (US) and multiply spliced (MS) HIV‐RNA were quantified by qPCR (n = 6). In a double‐blind, placebo‐controlled trial, MVC or placebo was added to suppressive ART in immune non‐responders (MVC = 10, Placebo = 5). Changes in cell‐associated (CA)‐US HIV‐RNA and NF‐κB regulated gene mRNA were quantified by droplet‐digital‐PCR (ddPCR). Mann‐Whitney‐U‐test and paired T‐test were performed using GrapPad‐Prism.

**Results:** *In vitro,* a significant increase in HIV production was observed when MVC (1 pmol/L to 1 μmol/L (*p* < 0.02) to infected PBMC. A 2.5‐fold increase in phosphorylated NF‐κB was observed in uninfected MVC treated CD4+ T‐cells. Additionally, *in vivo,* a significant difference, between MVC and placebo, in NF‐kB regulated gene expression, including IFN‐g, IL6 and TNF‐a, was observed (*p* = 0.02, 0.03, 0.05 respectively). Patients baseline characteristics did not differ between the MVC‐intensification and placebo‐group. A significant difference in CA‐US HIV‐RNA expression was detected between baseline and week eight (MVC 1.8‐fold increase; placebo 2.5‐fold decrease; *p* = 0.0121). *Ex vivo,* MVC induced a 3, 5 and 1.7‐fold increase in US HIV‐RNA compared to DMSO (*p* = 0.0004) and vorinostat (*p* = 0.0496) respectively. Additionally*,* MVC induced a 2‐fold increase in transcription of MS HIV‐RNA compared to DMSO (*p* = 0.0245).

**Conclusions: **MVC activates phosphorylation of NF‐κB and increases HIV‐RNA transcription in resting CD4 T‐cells. Potency for latency reversal *ex vivo* was more effective than vorinostat. Given the excellent safety profile of MVC, further studies of MVC as an LRA are warranted *in vivo*.

## WEAA0103

### Activation of latent HIV and SIV RNA transcription *in vitro* and *in vivo* in ART suppressed SIV‐infected rhesus macaques by the Ingenol‐based protein kinase C agonist, GSK445A

**A. Okoye^1,2^; R. Fromentin^3,4^; J. Brehm^5,6^; A. Maxwell^1,2^; M. Vaidya^1,2^; M. Pardons^3,4^; V. Tai^5^; J. Tang^5^; J. Smedly^2^; M. Axthelm^1,2^; J. Lifson^7^; L. Picker^1,2^; L. Trautmann^8,9^; D. Favre^5,6^ and N. Chomont^3,4^**

^1^Oregon Health and Science University, Vaccine & Gene Therapy Institute, Beaverton, United States, ^2^Oregon National Primate Research Center, Beaverton, United States, ^3^Centre de Recherche du CHUM, Montreal, Canada, ^4^Université de Montréal, Microbiology, Infectiology and Immunology, Montreal, Canada, ^5^GlaxoSmithKline, HIV Discovery Unit, Durham, United States, ^6^University of North Carolina, HIV Cure Center, Chapel Hill, United States, ^7^Frederick National Laboratory, Leidos Biomedical Research Inc., AIDS and Cancer Virus Program, Frederick, United States, ^8^Henry M. Jackson Foundation for the Advancement of Military Medicine, Bethesda, United States, ^9^Walter Reed Army Institute of Research, U.S. Military HIV Research Program, Silver Spring, United States

**Background:** Induction of HIV gene expression in latently‐infected CD4+ T cells is an essential step for the clearance of proviral reservoirs in virally suppressed individuals. Activation of NF‐kB signaling pathway by Protein kinase C agonists (PKCa) is a potent mechanism for HIV latency disruption *in vitro*. However, significant toxicity risks and the lack of evidence supporting their activity *in vivo* have prevented further evaluation of PKCa. Extending prior results, we sort to confirm that GSK445A, a stabilized Ingenol‐B PKCa derivative, can induce HIV/SIV transcription *in vitro*, and demonstrate pharmacological activity *in vivo* in ART suppressed SIV‐infected RM.

**Methods:** CD4+ T cells from 3 virally suppressed humans were exposed to increasing concentrations of GSK445A for 30 minutes to measure cell‐associated multiply spliced (tat/rev) RNA after 18 hours. Next, CD4+ T cells from virally suppressed humans (n = 5) and RM (n = 3), were exposed for 30 minutes at an optimal dose of 25 nmol/L GSK445A to quantify cell‐associated RNA and cell free RNA in the supernatant at 18 hours. Pharmacological activity and tolerability of GSK445A IV was assessed in 5 healthy RM at doses from 10 to 20mg/kg. Finally, 4 adult RM were IV inoculated with SIVmac239, and placed on ART (tenofovir/emtricitabine/dolutegravir) starting 56 days post‐infection. After 34 weeks, RM received 3 biweekly doses of GSK445A, IV at 15mg/kg. SIV DNA and RNA in cells and plasma were quantified by qPCR/qRT‐PCR.

**Results: ** CD4+ T cells exposed to GSK445A produced unspliced HIV and SIV RNA (gag) and viral particles, indicating that GSK445A efficiently reverses HIV/SIV latency *in vitro*. In vivo, GSK445A tolerability was established around 10 to 15 mg/kg and pharmacological activity demonstrated by CD69 upregulation in CD4+ T cells in blood. In suppressed RM, 3 of 4 individuals showed blips in plasma viral loads approximately 0.5 to 1 log above threshold (15 RNA copies/mL). All but 1 RM showed increases in unspliced SIV RNA in PBMC and increases in SIV RNA/DNA ratio (average transcription per infected cell) following each dose of GSK445A.

**Conclusions: **These results indicate that GSK445A is a potent latency‐reversing agent *in vitro* and is amenable to testing latency disruption strategies *in vivo* in RM models of HIV cure/remission.

## WEAA0104

### The RNA‐binding proteins, SRP14 and HMGB3 play a crucial role in controlling HIV replication and latency

**G. Khoury^1^; M.Y. Lee^1^; S. Ramarathinam^2^; S. Sonza^1^; A.W. Purcell^2^ and D.F.J. Purcell^1^**

^1^Peter Doherty Institute, Microbiology & Immunology, Melbourne, Australia, ^2^Biomedicine Discovery Institute, Monash University, Biochemistry and Molecular Biology, Clayton, Australia

**Background:** HIV latency is known to be reinforced by many impediments to RNA transcription, however translational blocks to HIV replication are less well characterised. The transactivator of transcription Tat protein is essential for progeny virion production in natural infection of HIV. RNA‐binding proteins that facilitate translation of Tat may be absent or downregulated in resting CD4+ T cells, the main reservoir of latent HIV. In this study, we examined the role of Tat RNA‐binding factors in expression of Tat and control of latent and productive infection.

**Methods:** Affinity purification‐mass spectrometry analysis (nanoLC‐MS/MS) was used to detect binding partners of MS2‐tagged *tat* mRNA in a T cell‐line model of HIV latency (J‐Lat6.3). 243 interactions were identified with high confidence using the MiST three parameter scoring system at a threshold cutoff of 0.7. 13 proteins were chosen for follow‐up. The effect of knockdown and overexpression of the proteins of interest on Tat transactivation and translation was assessed by luciferase‐based reporter assays, and infections with a dual colour HIV reporter virus allowed investigation of the dynamics of latent (BFP+/mCherry+) and productive (EGFP+BFP+/EGFP+mCherry+) infection. Changes over time in the levels of mRNA and protein in activated CD4+ and resting CD4+ T cells after NL4.3‐eGFP infection were determined.

**Results: ** After preliminary studies, two candidate proteins, SRp14 and HMGB3 were selected for detailed investigation. Knockdown of SRP14 negatively affected translation of Tat and Tat‐mediated transactivation, which led to an increase in latent infection (BFP+ expression), while the knockdown of HMGB3 resulted in an increase in Tat transactivation and translation as well as an increase in productive infection (EGFP+BFP+/mCherry+ expression). Interestingly, these effects correlated with the levels of the proteins in rCD4+ T cells following HIV‐1 infection as we observed a decrease in SRp14 levels while HMGB3 peaked very quickly after infection in rCD4+.

**Conclusions: **Our study revealed that SRp14 is a positive regulator of Tat expression and negative regulator of latent infection, whereas HMGB3 is a negative regulator of Tat expression and positive regulator of latent infection. The role of these proteins in controlling HIV‐gene expression during latency will be further assessed as potential drug targets.

## WEAA0105

### Using the PPARg antagonism to block/lock HIV reactivation in Th17 cells

**Y. Zhang^1^; D. Planas^1^; H. Chen^1^; A. Gosselin^1^; J.‐P. Routy^2^ and P. Ancuta^3^**

^1^Universite de Montreal and CHUM Research Centre, Microbiology, Infectiology and Immunology, Montreal, Canada, ^2^McGill University Health Centre, Montreal, Canada, ^3^Universite de Montreal and CHUM Research Centre, Montreal, Canada

**Background:** The Th17‐polarized cells represent a subset of CD4+ T‐cells that orchestrate mucosal immunity against pathogens. The transcriptional profile of Th17 cells is compatible with optimal HIV replication. Th17 are strategically located at mucosal barrier surfaces and represent the first HIV infection targets during sexual transmission. Finally, Th17 cells are long lived and support HIV reservoir persistence during antiretroviral therapy (ART). Of note, the nuclear receptor PPARγ is a negative regulator of HIV replication and a transcriptional repressor of the Th17 master regulator RORγt. In an effort to identify novel Th17‐targeted therapies, we investigated the potential use of PPARγ antagonism for viral latency reversal and/or boosting Th17 functions.

**Methods:** Memory CD4+ T cells were isolated from peripheral blood by negative selection. Cells of HIV‐uninfected individuals were stimulated via CD3/CD28 during three days, exposed to replication‐competent or single round VSVG‐pseudotyped HIV, and cultivated in presence/absence of PPARγ antagonist T0070907 for 12 days. HIV reactivation from CD4+ T‐cells of ART‐treated individuals was measured using a viral outgrowth assay. HIV replication was monitored by HIV‐DNA real‐time PCR and ELISA/FACS. CCR5 expression was measured by FACS. Genome‐wide tanscriptional profiling was performed using the Illumina RNA‐Seq technology followed by RT‐PCR validations.

**Results: ** The PPARγ antagonist T0070907 increased IL‐17A production, but unexpectedly inhibited HIV replication by acting at multiple levels including CCR5‐mediated entry; HIV transcription; and lipid metabolism. Transcriptional profiling and RT‐PCR validations revealed that T0070907‐mediated effects coincided with the induction of cholesterol‐25‐hydroxylase (CH25H), an enzyme converting cholesterol into 25‐hydroxycholesterol (25HC), a broad inhibitor of viral replication and also an intrinsic ligand of RORγt. Finally, T0070907 inhibited HIV reactivation and increased IL‐17A production in CD4+ T cells from ART‐treated HIV+ individuals.

**Conclusions: **Together, our results identify PPARγ as a new therapeutic target to reduce HIV replication/reactivation in CD4+ T‐cells, while boosting the Th17 effector functions. These effects are explained by the capacity of T0070907 to induce the synthesis of 25HC, while preventing PPARγ‐mediated RORγt repression. These findings demonstrate the possibility to disconnect HIV replication from effector functions in Th17 cells and open the path for new therapeutic interventions to restore Th17‐mediated mucosal immunity in HIV‐infected individuals receiving ART.

## WEAA0201

### Increase in restriction factor expression in response to viral rebound after analytical treatment interruption in HIV‐infected patients

**M.‐A. De Scheerder^1,2^; C. Van Hecke^1,2^; N. De Langhe^1^; M. Sips^1,2^ and L. Vandekerckhove^1,2^**

^1^UZ Gent, Algemene Inwendige Ziekten, Gent, Belgium, ^2^UGent, HCRC, Gent, Belgium

**Background:** Restriction factors are host proteins interfering at different steps of HIV replication cycle in an attempt to limit viral production and spreading. We conducted a longitudinal analysis of the expression profile of antiviral restriction factors and cofactors in a cohort of eleven HIV‐1 infected, long‐term treated patients who underwent analytical treatment interruption (ATI), the HIV‐STAR study(NCT02641756). The expression of known HIV‐1 restriction factors (APOBEC3G, SAMHD1, MX2, PAF1, SLFN11, TRIM5α and BST2/tetherin), cofactors (NLRX1 and PSIP1) and interferon stimulated genes (ISGs) IFIT1 and MX1 were evaluated at four well‐defined time points: on cART (T1), after ATI (VL <20 copies/mL, T2), at rebound (VL >1000 copies/mL, T3) and after treatment restart (VL <20 copies/mL, T4).

**Methods:** Peripheral blood mononuclear cells were isolated from all patients at the four time points. Quantitative real‐time PCR was performed to determine the expression of these HIV‐1 restriction factors, cofactors and ISGs. Statistical Friedman's and post hoc Dunn's analysis were performed and Spearman correlation was employed to identify associations between restriction factor/cofactor, ISG expression levels and patient clinical characteristics.

**Results: ** For two restriction factors a significant increase in expression between T1 and T2 (APOBEC3G, SLFN11) was observed. Furthermore, upregulation of MX2 and both ISGs were observed between T1 and T3 (*p* < 0.05). Significant positive correlations between ISG and restriction factor/cofactor expression were identified at T1, T3 and T4. In addition, a correlation between high HIV DNA load and IFIT1 at T2 and high viral load zenith and SLFN11 at T4 was found, suggesting that these virological characteristics drive a more robust restriction factor response. Furthermore, a low CD4 nadir was correlated with a higher expression of the viral co‐factor PSIP1 at T2. No associations were found between the expression of restriction‐ or co‐ factors and time to viral rebound.

**Conclusions: **A significant difference in restriction factor expression between at least two time points was identified for SLFN11, APOBEC3G and MX2 and a trend towards difference in expression levels for most of the other restriction factors studied. In contrast to the ISGs, our data indicate that restriction factors increase earlier after ATI before time of viral rebound, potentially implicating them as markers/predictors of rebound.

## WEAA0202

### RhCMV‐induced, SIV‐specific MHC‐E‐restricted T cells recognize SIV through the T cell receptor

**S. Abdulhaqq^1^; B. Bimber^2^; H. Wu^1^; A. Ventura^1^; K. Wisskirchen^3^; A. Legasse^2^; M. Axthelm^2^; U. Protzer^3^; S. Hansen^1^; D. Douek^4^; L. Picker^1^ and J. Sacha^1^**

^1^Oregon Health and Science University, Vaccine and Gene Therapy Institute, Beaverton, United States, ^2^Oregon Health and Science University, Oregon National Primate Research Center, Beaverton, United States, ^3^Technical University of Munich, Munich, Germany, ^4^National Institutes of Health, Vaccine Research Center, Bethesda, United States

**Background:** RhCMV68‐1 vaccine vectors expressing SIV antigens demonstrate a profound ability to control post‐challenge viremia with subsequent SIV clearance in over half of all vaccinated rhesus macaques (RM). The T cells induced by these vectors are effector‐memory T cells unconventionally MHC restricted by MHC‐II or MHC‐E molecules. To effectively deploy these responses against HIV/SIV it is essential to garner a better understanding of how these cells function. However, the unique nature of these terminally differentiated cells complicates conventional analytical methods, e.g. antigen‐specific T cell lines. Here, we employ bulk and single‐cell sorting with downstream mRNA sequencing of SIV‐specific MHC‐E‐restricted CD8+ T cells to derive full length, paired α/β T cell receptor (TCR) sequences and to characterize their transcriptional phenotype.

**Methods:** We stimulated PBMC from strain 68‐1 RhCMV/Gag vaccinated RM with optimal peptide epitopes from previously identified T cell responses in the presence of TAPI‐0. Antigen‐specific T cells dually expressing CD69 and membrane‐bound TNF‐α were sorted using a FACS Aria. Whole transcriptome sequencing was used to identify the CDR3 amino acid sequence and V(D)J usage. Paired TCR chains were exogenously expressed in allogeneic CD8+ T‐Cells from RhCMV/Gag‐naïve RM.

**Results: ** Using our system we were able to identify, sequence, and subsequently express paired α/β TCRs from MHC‐E‐restricted, SIV‐specific CD8+ T cells. We confirmed that allogeneic CD8+ T cells transduced with these exogenous TCRs secreted effector cytokines upon encountering antigen in the context of MHC‐E. MHC‐E TCR transductants also recognized SIV infected CD4+ T cells derived from autologous or allogeneic sources, demonstrating the presence of MHC‐E‐bound minimal optimal epitopes on the surface of infected cells. Total transcriptome analysis revealed distinct populations within the MHC‐E restricted cells, and expression of canonical Th1 markers, consistent with conventional MHC‐Ia‐restricted SIV‐specific CD8+ T cells.

**Conclusions: **Our data demonstrate that Mamu‐E‐restricted T cell specificity is derived from its cognate TCR, and that the mRNA profiles of these cells resemble conventionally MHC‐restricted T cells. This new system facilitates downstream transcriptomic analysis of antigen‐specific T cells independent of knowledge of the restricting allele and tetramers, and will shed light on the phenotype and function of unconventionally MHC‐restricted CD8+ T cells induced by RhCMV vaccination.

## WEAA0203

### Genetic factors leading to loss of viral control in HIV elite controller patients

**J.M. Benito^1,2^; M. García^1,2^; V. de Santisteban^1,2^; R. Ramos^3^; C. Restrepo^1,2^; M.A. Navarrete‐Muñoz^1,2^; A. León^4^; E. Ruiz‐Mateos^5^; A. Cabello^6^; J. Alcamí^7^; M. Górgolas^6^; N. Rallón^1,2^ and On behalf of ECRIS integrated in the Spanish AIDS Research Network**

^1^Instituto de Investigación Sanitaria Fundación Jiménez Díaz, Universidad Autónoma de Madrid (IIS‐FJD, UAM), Madrid, Spain, ^2^Hospital Universitario Rey Juan Carlos, Móstoles, Spain, ^3^Unidad de Genómica Parque Científico de Madrid, Madrid, Spain, ^4^Hospital Clinic‐IDIBAPS, HIVACAT, Universidad de Barcelona, Barcelona, Spain, ^5^Hospital Virgen del Rocío, Sevilla, Spain, ^6^Hospital Universitario Fundación Jiménez Díaz, Madrid, Spain, ^7^Unidad de Inmunopatología del SIDA, Centro Nacional de Microbiología, Instituto de Salud Carlos III, Madrid, Spain

**Background:** Despite the ability of HIV elite controller patients (EC) to spontaneously maintain undetectable HIV plasma viral load (pVL), some of them lose this ability over time. The mechanisms underlying this phenomenon are not completely understood. We investigated the association of genes related to HIV pathogenesis, with the loss of spontaneous virological control in EC.

**Methods:** A retrospective longitudinal study was performed in 13 EC: Six experienced loss of virological control (at least two consecutive measurements of VL above 50 copies/mL over 12 months of follow‐up), named transient‐controllers (TC), and seven maintained virological control during follow‐up (named persistent‐controllers, PC). Cell samples were obtained at two timepoints: one to two years before (T1) and 1 to two years after (T2) loss of virological control for TC, and separated 1 to 2 years for PC. We assessed the expression of 43 genes related to HIV pathogenesis by real‐time qPCR. Differential gene expression between TC and PC at the two timepoints and between timepoints was analyzed using StatMiner software (Applied Biosystems) applying a false discovery rate (FDR) <0.05. Principal component analysis (PCA) was employed to discriminate between TC and PC at different timepoints.

**Results: ** PCA was able to discriminate between TC and PC at T1 when both groups presented undetectable pVL. However, the discrimination was not so clear at T2 when TC already had detectable levels of pVL. On the other hand, PCA discriminated T1 and T2 samples from TC but it was not for T1 and T2 samples from PC. Interestingly, several genes involved in HIV control such as CDKN1A (*p* = 0.024), CTR9 (*p* = 0.006) and IFI16 (*p* = 0.046) were down‐regulated in TC compared to PC at T1, whereas there were no significant differences at T2. Moreover, gene expression did not change between T1 and T2 in PC, whereas several genes (ABCA1, IL10, IL21, PAF1,TRIM26) significantly increased its expression at T2 compared to T1 in TC.

**Conclusions: **Our results demonstrate a down‐modulation of different genes with anti‐HIV activity in EC patients that precede the loss of natural HIV replication control. These genes could be considered potential biomarkers of loss of natural control and could provide new insights for the clinical management of these exceptional patients.

## WEAA0204

### Frequent generation of HIV broadly neutralizing antibodies in infected children is associated with both increased help and regulation within germinal centers

**J. Roider^1,2,3^; T. Maehara^4^; A. Ngoepe^2^; D. Ramsuran^2^; M. Muenchhoff^5,6^; E. Adland^1^; T. Aicher^4^; S. Kazer^4^; P. Jooste^7^; F. Karim^2^; W. Kuhn^8^; A. Shalek^4,9,10^; T. Ndung'u^2,3,4^; L. Morris^11,12,13^; P. Moore^11,12,13^; S. Pillai^4^; P. Goulder^1,3^; H. Kloverpris^2,14^ and A. Leslie^2^**

^1^University of Oxford, Department of Pediatrics, Oxford, South Africa, ^2^Africa Health Research Institute, Durban, South Africa, ^3^HIV Pathogenesis Programme, Durban, South Africa, ^4^Ragon Institute of Massachusetts General Hospital, Massachusetts Institute of Technology and Harvard University, Cambridge, United States, ^5^Max von Pettenkofer‐Institute, Muenchen, Germany, ^6^German Center for Infection Research (DZIF), München, Germany, ^7^Kimberley Hospital, Kimberley, South Africa, ^8^Stanger Hospital, Stanger, South Africa, ^9^Broad Institute of MIT and Harvard, Cambridge, United States, ^10^Institute for Medical Engineering & Science (IMES) and Department of Chemistry,, Cambridge, United States, ^11^National Institute for Communicable Diseases of the National Health Laboratory Service, Johannesburg, South Africa, ^12^University of the Witwatersrand, Johannesburg, South Africa, ^13^Center for the AIDS Programme of Research in South Africa, Durban, South Africa, ^14^University of Copenhagen, Department of Immunology and Microbiology, Copenhagen, Denmark

**Background:** Understanding the T cell parameters supporting the generation of broadly neutralizing antibodies (bnAbs) against HIV‐1 will help to optimize and target future vaccine strategies. Unlike HIV infected adults, who rarely make bnABS, most vertically infected children generate broad and potent neutralizing antibodies, suggesting the immune environment in these infants supports their generation.

**Methods:** Using blood and lymph node samples from infants and adults, we performed flow cytometry, confocal imaging and analysis of plasma markers by ELISA/ multiplex assays.

**Results: ** As in the rare adults that make bnABs, circulating T follicular helper cells (TFH) correlate with neutralization breadth in infected infants, but are much more frequent than in their adults counterparts. This is reflected in lymph nodes (LN), were germinal center TFH are double the frequency in infants, irrespective of HIV status. Moreover, HIV infected infants have a clear Th2 bias in their LN TFH response, with a high frequency of HIV‐specific IL‐21 producing TFH, compared to adults, whose low frequency HIV‐specific TFH make IFN‐gamma. Unlike in adults, TFH in HIV infected infants are subject to increased regulation in LN, where the ratio of regulatory TFH to TFH is double that of infected adults and HIV‐specific CXCR5+ CD8 T cells are frequently detected. Finally, breadth in infants correlates with plasma levels of IL‐5, a cytokine that supports the induction of regulatory T and B cells.

**Conclusions: **The existence of differences in the immune response to natural infection of infants and adults is important, as it argues that vaccination strategy should be tailored to the group being targeted. For example, a vaccine that sought to break tolerance controls in adults might not be appropriate in infants. Furthermore, the general observation that infants make better anti‐HIV antibodies should raise the question of whether infants are potentially a better target for HIV vaccination. Understanding the role of T‐cell help and regulation in the process of bNab generation in infants can inform the development of vaccines targeted at this age group.

## WEAA0205

### Initiation of antiretroviral therapy during hyperacute HIV infection preserves germinal center T follicular (GCTfh) helper cell function

**O. Baiyegunhi^1^; F. Laher^1^; K. Dong^2^; B. Walker^2^; T. Ndungu^1,3^ and Z. Ndhlovu^1,2,3^**

^1^University of KwaZulu Natal, Nelson Mandela School of Medicine, Durban, South Africa, ^2^Ragon Institute of MGH, MIT and Harvard, Cambridge, United States, ^3^Africa Health Research Institute, Durban, South Africa

**Background:** T follicular helper (Tfh) cells are important for the development of antibody responses against HIV infection. Paradoxically, the pathologic expansion of Tfh is associated with hypergammaglobulinemia and B cell dysfunction. Many HIV induced immune dysfunctions are reversed or attenuated by antiretroviral therapy (ART) but it is unclear if early treatment restores germinal center (GC) Tfh delivery of help to B cells leading to the establishment of GC reactions and effective antibody responses.

**Methods:** Excisional lymph node and paired blood samples were collected from 20 treated persons at the onset of plasma viremia, in many when viral loads are less than 1000 RNA copies/mL plasma. 10 HIV negative and 8 untreated individuals were included as controls. GCTfh and B cell phenotype and *in‐situ* microscopy was performed on all study subjects. MHC class II tetramer staining, digital droplet PCR and intracellular cytokine staining were used to quantify and phenotype HIV‐specific GCTfh responses is a subset of donors based on MHC class II haplotype expression and sample availability.

**Results: ** Despite prompt plasma virus suppression there was significant expansion of GCTfh frequencies in lymph nodes from early treated persons compared to HIV negative donors. Class II tetramer staining and intracellular flowcytometry analysis revealed 1% to 9% of expanded GCTfh cells was HIV‐specific. Expansion of plasmablasts (*p* = 0.0336) and GC B cells (*p* = 0.0571) correlated with GCTfh cell frequencies (*p* = 0.0003, r = 0.9321). GCTfh/B cell cultures induced higher IgG production in early treated compared to untreated donors. HIV Gag p24 antigen was detected in CXCR3^+^ GC Tfh and follicular dendritic cells, almost exclusively within GCs.

**Conclusions: **These results demonstrate that GCTfh responses induced during treated hyperacute are qualitatively superior compared to responses untreated hyperacute HIV infection. Our results also provide hints of persistent low‐level viral replication in the lymph nodes of early treated individuals despite sustained plasma viral suppression. These results have implications for HIV cure and for vaccine strategies aimed at inducing long lasting anti‐HIV antibody responses.

## WEAB0101

### Evaluation of a national cryptococcal antigen screening program for HIV‐infected patients in Uganda: a cost‐effectiveness modeling analysis

**R. Rajasingham^1^; D. Meya^2^; G. Greene^3^; A. Jordan^3^; T. Chiller^3^; D. Boulware^1^ and B. Larson^4^**

^1^University of Minnesota, Minneapolis, United States, ^2^Infectious Diseases Institute, Kampala, Uganda, ^3^CDC, Atlanta, United States, ^4^Boston University, Boston, United States

**Background:** Cryptococcal meningitis accounts for 15% of AIDS‐related mortality. Cryptococcal antigen (CrAg) is detected in blood weeks before onset of meningitis, and CrAg positivity is an independent predictor of meningitis and death. CrAg screening for patients with advanced HIV is recommended by the World Health Organization, though implementation remains limited. Our objective was to evaluate costs and mortality reduction (lives saved) from a national CrAg screening program across Uganda.

**Methods:** We created a decision analytic model to evaluate CrAg screening. CrAg screening was considered for those with a CD4 <100 cells/μL per international guidelines, and in the context of a national HIV test and treatment program where CD4 testing may not be available. Costs (2016 USD) were estimated for screening, preemptive therapy, hospitalization, and maintenance therapy. Parameter assumptions are based on large prospective CrAg screening studies in Uganda, and clinical trials from sub Saharan Africa.

**Results: ** In the base case for 1 million persons with a CD4 test annually, 128,000 with a CD4 <100 cells/μL were screened, and 8233 were asymptomatic CrAg+ and received preemptive therapy. Compared to no screening, CrAg screening and treatment in the base case costs $3,356,724, and saves 7320 lives, for a cost of $459 per life saved.

Within a national HIV test and treat program, of 1 million HIV‐infected persons, 5920 were incident CrAg positive (CrAg prevalence 1.5%). The total costs of a CrAg screening and treatment program was $4.12 million dollars, with 2229 known deaths. Conversely without CrAg screening, the cost of treating meningitis was $5.45 million dollars with 6712 deaths. Thus, despite the very low CrAg prevalence at about 1.5% in the general HIV‐infected population, CrAg screening saved over $1.32 million (i.e. 13% of total costs) and averted 67% of deaths, saving $295 per death averted.

**Conclusions: **CrAg screening and treatment programs are cost saving and lifesaving and could be adopted and implemented by ministries of health to reduce mortality in those with advanced HIV disease. Even within HIV test and treat programs where CD4 testing is not performed, and CrAg prevalence is only 1.5%, CrAg screening is a worthy investment.

**Abstract WEAB0101‐Table 1. Input parameters for base case model)**

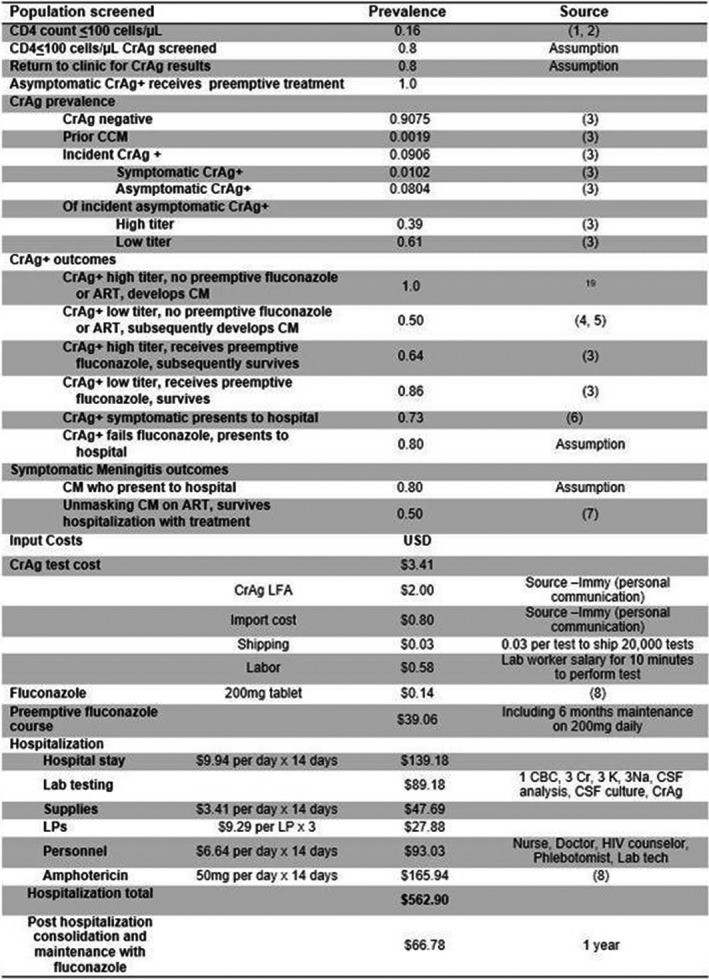

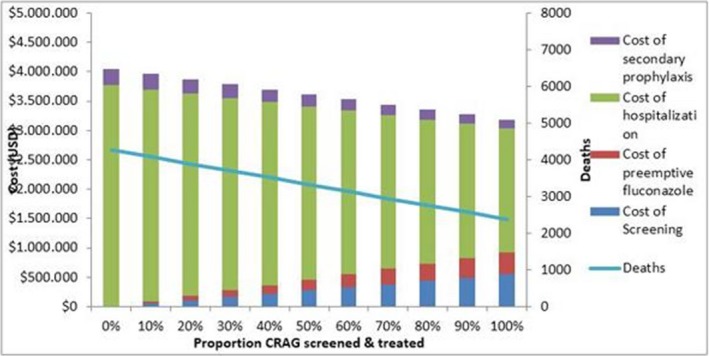

**Abstract WEAB0101‐Figure 1. Cost of CrAg screening and preemptive treatment with differential levels of implementation. With 100% CrAg screening and treatment, 1900 lives are saved (44%) and $860,000 dollars, compared to no screening.**

## WEAB0102

### HIV‐associated central nervous system infections in Indonesia: a cohort study examining etiology, presentation and outcome

**D. Imran^1^; R. Estiasari^1^; K. Maharani^1^; S. Sucipto^1^; D.C. Lestari^2^; R. Eddy^3^; E. Yunihastuti^4^; T. Harjono^4^; D. Oei^1^; I.S. Timan^5^; D. Wulandari^5^; R. Wahyuningsih^6^; A. Bahri^6^; A. Kurniawan^6^; R. Mulyadi^3^; A. Karuniawati^2^; U.A. Jaya^7,8^; D. Safari^7^; A. van Laarhoven^9^; B. Alisjahbana^10^; S. Dian^10^; L. Chaidir^10^; A.R. Ganiem^10^; D.N. Lastri^1^; K.S.A. Myint^7^ and R. van Crevel^8,9^**

^1^Cipto Mangunkusumo Hospital ‐ Faculty of Medicine Universitas Indonesia, Department of Neurology, Jakarta, Indonesia, ^2^Cipto Mangunkusumo Hospital ‐ Faculty of Medicine Universitas Indonesia, Department of Microbiology, Jakarta, Indonesia, ^3^Cipto Mangunkusumo Hospital ‐ Faculty of Medicine Universitas Indonesia, Department of Radiology, Jakarta, Indonesia, ^4^Cipto Mangunkusumo Hospital ‐ Faculty of Medicine Universitas Indonesia, Department of Internal Medicine, Jakarta, Indonesia, ^5^Cipto Mangunkusumo Hospital ‐ Faculty of Medicine Universitas Indonesia, Department of Clinical Pathology, Jakarta, Indonesia, ^6^Cipto Mangunkusumo Hospital ‐ Faculty of Medicine Universitas Indonesia, Department of Parasitology, Jakarta, Indonesia, ^7^Eijkman Institute for Molecular Biology, Jakarta, Indonesia, ^8^Eijkman‐Oxford Clinical Research Unit, Jakarta, Indonesia, ^9^Radboud Center for Infectious Diseases, Radboud University Medical Center, Department of Internal Medicine, Nijmegen, Netherlands, ^10^Faculty of Medicine Universitas Padjadjaran, TB/HIV Research Center, Bandung, Indonesia

**Background:** HIV infection leads to increased susceptibility and worse outcome of central nervous system (CNS) infections. We examined etiology and outcome of CNS infections and the effect of HIV in Indonesia. which is witnessing the second fastest growing HIV epidemic in Asia.

**Methods:** We prospectively included adults with suspected CNS infections during 15 months in a referral hospital in Jakarta. Systematic screening included HIV testing, routine cerebrospinal fluid (CSF) examination, neuroimaging, and paired HIV‐RNA measurement in blood and CSF.

**Results: ** 274 patients with suspected CNS infection (median age 26) presented with headache (77%), fever (78%), seizures (27%), loss of consciousness (71%) and focal neurological signs (40%). HIV infection was found in 147 (54%) patients with 56% newly diagnosed. Those with previously diagnosed HIV infection, 50% had a history of prior or current ART use and 18% reported cotrimoxazole PCP‐prophylaxis. Lumbar puncture in HIV‐positive subject was done in 80 patients (54%), and brain CT scan in 116 patients (79%). Among HIV‐infected patients, we diagnosed cerebral toxoplasmosis (33%), tuberculous meningitis (22%), cryptococcal meningitis (9%), viral encephalitis (5%), brain abscess (2%), neurosyphilis (0.5%) and cerebral lymphoma (0.5%) while no diagnosis could be made in 28% of patients. HIV‐RNA was done in blood and CSF of 65/147 patients (44%). Six patients have undetected HIV‐RNA in their blood and CSF. Blood HIV‐RNA range was 80 ‐ 7.26.10^6^ and in CSF was 138 to 1.69.10^6^ Follow‐up was 97% complete. Mortality was strongly associated with HIV‐infection; 37% of those with and 26% of those without HIV died during hospitalisation, and 67% respectively 45% had died after six months follow‐up (*p* < 0.01). Compared to patients with previously diagnosed HIV infection, those with newly diagnosed HIV had a similar CD4 cell count (median 29/mL vs. 30/mL), but a higher mortality (log‐rank test 0.03).

**Conclusions: **In this setting, patients with CNS infections came very late and with severe disease. HIV infection was very common, very advanced and associated with very poor outcome. Although 50% have ART history, only six patients have undetected HIV‐RNA in their blood and CSF. These data underline the need to step up efforts to improve testing, ART and opportunistic infection management.

## WEAB0103

### Burden of sexually transmitted infections and prevalence of HIV among key population individuals presenting with STIs in Nepal

**D.P. Bhandari; K.P. KC; H. Subhani; R.K. Shrestha; B.M. Shrestha; P.K. Thakur; I. Adhikary and B. Shrestha**

FHI 360, Nepal, Kathmandu, Nepal

**Background:** Under the current global vision to control the HIV epidemic the through expanded provision of antiretroviral medications, relatively little attention has been devoted to sexually transmitted infections (STIs). Unfortunately, STIs remain a major cause of morbidity among people living with HIV, and STIs are associated with increased risk for HIV infection. This analysis assessed the burden of STIs among key populations (KPs), as well as prevalence of HIV among STI patients attending LINKAGES/Nepal‐supported clinics.

**Description:** LINKAGES/Nepal — led by FHI 360 with support from the U.S. Agency for International Development and the U.S. President's Emergency Plan for AIDS Relief — provides HIV and STI diagnostic and case‐management services to female sex workers (FSWs), clients of FSWs, men who have sex with men (MSM), male sex workers (MSWs), and transgender people in 16 districts of Nepal. The STI case‐management services include counseling and syndromic management, syphilis screening using the rapid plasma reagin (RPR) test and treatment with benzathine penicillin, condom distribution to all KP members, and presumptive treatment of cervicitis for FSWs.

**Lessons learned:** From October 2016 to September 2017, 23,454 individuals (49% males, 50% females, and 1% trans people) were screened for STIs, of whom 5475 (23%) were diagnosed with any STI. The most common diagnoses were vaginal discharge syndrome (69%) followed by syphilis (14%), urethral discharge syndrome (9%), and genital warts (4%). HIV prevalence among STI patients was 0.8%. HIV prevalence was highest among patients with syphilis at 3%; followed by patients with genital warts, at 2%; and vaginal discharge syndrome, at 0.4%. Out of 42 HIV‐positive cases among STI patients, 21 (50%) had syphilis, 16 (38%) had vaginal discharge syndrome, 4 (10%) had genital warts, and one (2%) had urethral discharge syndrome.

**Conclusions/Next steps:** The STI burden among KP program beneficiaries is high, and many HIV‐positive KP members are co‐infected with an STI. Providing integrated services helps to link a large number of KP individuals to needed STI services in this resource‐limited setting.

## WEAB0104

### Natural history of anogenital HPV infection and related disease among HIV‐positive men: Findings from a Cohort Study in South Africa

**A. Chikandiwa^1^; P.T. Pisa^1^; C. Tamalet^2^; E.E. Muller^3^; R. Kularatne^3^; P. Michelow^4^; M.F. Chersich^1^; P. Mayaud^1,5^ and S. Delany‐Moretlwe^1^**

^1^University of the Witwatersrand, Wits Reproductive and HIV Institute, Johannesburg, South Africa, ^2^Timone University, Clinical Microbiology IHU and CNRS‐URMITE, Marseille, France, ^3^National Health Laboratory Service, National Institute for Communicable Diseases, Johannesburg, South Africa, ^4^University of the Witwatersrand, Anatomical Pathology, Johannesburg, South Africa, ^5^London School of Hygiene and Tropical Medicine, Clinical Research, London, United Kingdom

**Background:** Persistent anogenital HPV infection causes anogenital warts (AGW), penile and anal cancers. We determined persistence of anogenital HPV infection, squamous intraepithelial lesions (SIL) and AGW among men living with HIV (MLHIV).

**Methods:** We enrolled 304 sexually‐active MLHIV ≥18 years from Johannesburg. We collected socio‐behavioral data, blood (CD4+ counts and HIV‐1 plasma viral load (PVL)), anal, genital swabs (HPV DNA genotyping with Roche Linear Array and HPV16/18 viral load (VL) with RT‐PCR on 22 swabs), and anal smears and examined for AGW at enrolment and 6‐monthly follow‐up visits for 18 months. Time to AGW incidence or clearance was estimated by Kaplan‐Meier method. Correlates of persistent HPV infection, SIL and AGW clearance were evaluated with generalized estimating equations, logistic and ox regressions respectively. ROC analysis evaluated performance of HPV16/18 VL in predicting persistent SIL.

**Results: ** A total of 260 (86%) and 259 (85%) MLHIV had anal and genital HPV results at both enrolment and final visits respectively. The median age was 38 (IQR: 22 to 59) years, 25% reported ≥1 sexual partners in the past three months and 5% reported ever having sex with other men. Most participants (65%) were on ART, with median CD4+ count 445 cells/μL (IQR: 328 to 567). Prevalence of anal and genital HPV infection was 39% (88/227) and 79% (224/283) respectively. The prevalence of anal SILs and AGW were 49% (120/244) and 12% (36/304) respectively. Persistence for anal and genital HPV infection were 26.2% (21/80) and 35.4% (68/192) respectively. HPV persistence was strongly associated with low enrolment CD4+ count (<200 vs. >500 cells/μL, aOR = 6.58; 95% CI: 2.41 to 17.94). Anal SIL incidence and persistence were 27.4% (34/124) and 30% (36/120) respectively. Prevalent anal HPV infection (aOR = 5.08; 95% CI: 2.04 to 12.66) was associated with persistent SILs. AGW incidence was 1.4 per 100 person‐years. Median time to AGW clearance was 0.7 (IQR, 0.5 to 1.1) years. AGW clearance was strongly associated with CD4+ count (<350 vs. ≥350 cells/μL, aOR = 3.69; 95% CI: 1.44 to 9.47). Enrolment HPV16/18 VL performed poorly in predicting persistent SILs.

**Conclusions: **MLHIV have high persistence of anogenital HPV infection and related disease. HPV vaccination among boys and effective use of ART with immunological reconstitution could reduce this burden.

## WEAB0105

### Kaposi disease in HIV‐infected patients with suppressed HIV viremia: the experience of the French national multidisciplinary committee ONCOVIH

**R. Palich^1^; M. Veyri^2^; M.‐A. Valantin^1^; A.‐G. Marcelin^3^; F. Boué^4^; A. Guihot^5^; C. Solas^6^; H. Ait‐Mohand^1^; V. Martinez^1^; I. Poizot‐Martin^7^; D. Costagliola^8^; J.‐P. Spano^2^; C. Katlama^1^ and ONCOVIH Study Group**

^1^Pitié‐Salpêtrière Hospital, AP‐HP, Infectious Diseases, Paris, France, ^2^Pitié‐Salpêtrière Hospital, AP‐HP, Oncology, Paris, France, ^3^Pitié‐Salpêtrière Hospital, AP‐HP, Virology, Paris, France, ^4^Antoine‐Béclère Hospital, AP‐HP, Clinical Immunology, Clamart, France, ^5^Pitié‐Salpêtrière Hospital, AP‐HP, Immunology, Paris, France, ^6^La Timone Hospital, AP‐HM, Pharmacology, Marseille, France, ^7^Saint‐Marguerite Hospital, AP‐HM, Clinical Immunology, Marseille, France, ^8^Pierre Louis Institute of Epidemiology and Public Health (IPLESP, UMRS 1136), Paris, France

**Background:** Kaposi disease (KD) is among the most frequent HIV associated cancers, classically occurring in HIV‐replicating individuals. Since 2014, the ONCOVIH national multidisciplinary committee (MDC) registers cancers in HIV infected patients. We report our experience of KD occurring in patients despite sustained virological suppression.

**Methods:** This observational and national study enrolled all cases of individuals with a first episode or a relapse of KD, on ART for at least 12 months, with a plasma viral load (pVL) <50 copies/mL at the time of KD diagnosis.

**Results: ** The French ONCOVIH MDC registered a total of 72 KD cases between May 2014 and November 2017. We included for analysis the 22/ 72 (31%) who fulfilled inclusion criteria, whereas 38/72 (53%) had pVL >50 copies/mL at the time of KD diagnostic, and 12 had missing data. They were 18 male and 4 female, born in France for 10 of them and in Africa for 12, with a median age of 51 years (IQR 34 to 61). HIV infection was diagnosed 12 years earlier (IQR 5 to 14). CD4 nadir was 200/mm^3^ (IQR 73 to 290), and median duration of virological suppression four years (IQR 2 to 5). KD was a relapse in 59% of cases, and a first episode in 41%. KD localisations involved skin (100% of cases), lymph nodes (27%), bronchi (18%), oesophagus/stomach (14%), bone (14%), and/or palate (5%). Median CD4 count was 478/mm^3^ (IQR 269 to 630) with a CD4/CD8 ratio of 0.58 (IQR 0.34 to 0.75). At time of MDC, KD therapy had included anthracyclines in 36% of cases and/or taxanes in 27% of cases. In November 2017, from the follow up of 16/22 patients, all were alive, with a KD in partial remission in 31% of cases, stability in 38% and progression in 31%.

**Conclusions: **Kaposi disease is observed in aging patients with suppressed viremia either as relapse or new case. HHV‐8 as main cause for KD need to be further investigated. Follow up should bring information towards treatment response. Anti PD1 may deserve a pilot investigation in patients failing standard anti‐KD chemotherapies.

## WEAB0106

### Kaposi sarcoma incidence remains unchanged among African American males in the Southern United States: U.S. Cancer Statistic Data, 2000 to 2014

**E. Chiao; T. Aaron; Y. Dong; R. Kathryn; J. Kramer and W. Donna**

Baylor College of Medicine, Houston, United States

**Background:** Kaposi sarcoma (KS) is the most common neoplasm of people living with HIV today. Although the overall incidence of KS has been reported to be declining in the US, KS has strong racial/ethnic, age, and regional diversity in incidence trends.

**Methods:** We analyzed KS incidence data from the US Cancer Statistics (USCS) registry for the years 2000 to 2014. The USCS registry is the official data source for federal government‐reported cancer incidence statistics and covers 97% of the US population. Women were excluded because of the low numbers of KS cases in certain geographic regions, and our analyses only included 20 to 54 year‐old age‐group as prior validation studies indicated that ˜94% of KS cases in this age‐range are HIV‐related. We calculated adjusted incidence rates and assessed annual trends among sociodemographic and geographic subgroups using joinpoint regression analysis.

**Results: ** During the study period, 12,549 men were diagnosed with KS. The overall incidence of KS among men decreased from 1.42/100,000 in 2000 to 0.95/100,000 in 2014, decreasing by 3.60% (95% confidence interval (CI), −4.00% to −3.13%) annually. The overall annual percent change (APC) for men significant decreased (−6.27%, *p* < 0.05) from 2007 to 2010 and again (−2.13%, *p* < 0.05) from 2010 to 2014. Among African American, non‐Hispanic Caucasian, and Hispanic men, the incidences in 2014 were 2.37/100,000, 0.49/100,000, and 1.22/100,000, respectively. Although there was a decrease in the APC among African American men from 2000 to 2014 (−3.31%, *p* < 0.05), there were differences in the rate of change among African American men by region. In the Northeast, the APC was noted to have 3 joinpoints, with non‐significant decreases in incidence in years 2009 to 2012 (APC = −0.23%, *p* < 0.05), followed by a significant decrease in years 2012 to 2014 (APC = −26.17%, *p* < 0.05). In the Midwest and West there were significant decreases throughout years 2000 to 2014 (APC = −3.4%, *p* < 0.05, and APC = −5.59%, *p* < 0.05, respectively). However, in the South, there has been no significant change in incidence (APC = −0.86%, *p*>0.05) of KS among African American men.

**Conclusions: **Geographic disparities in KS incidence remain for African American men in the U.S. Between the years 2000 to 2014, unlike other regions, the incidence of KS has remained unchanged in the southern U.S.

## WEAB0201

### Developmental and cognitive effects of type of antepartum and postpartum ARV exposure for Ugandan and Malawian IMPAACT PROMISE HIV‐exposed versus unexposed children at age 12, 24 and 48 months

**M. Boivin^1,2^; L. Maliwichi‐Senganimalunje^3,4^; L. Wambuzi Ogwang^5^; R. Kawalazira^3^; A. Sikorskii^6^; I. Familiar‐Lopez^7^; A. Kuteesa^5^; M. Nyakato^5^; A. Mutebe^5,8^; M. Makitende^5^; M. Mallewa^3^; H. Ruisenor‐Escudero^7^; J. Aizire^9^; T. Taha^9^ and M.G. Fowler^10^**

^1^Michigan State University, Psychiatry and Neurology & Ophthalmology, East Lansing, United States, ^2^University of Michigan, Psychiatry, An Arbor, United States, ^3^Malawi College of Medicine ‐ Johns Hopkins University, Blantyre, Malawi, ^4^University of Malawi, Chancellor College, Zomba, Malawi, ^5^Makerere University ‐ Johns Hopkins University, Kampala, Uganda, ^6^Michigan State University, Psychiatry and Statistics & Probability, East Lansing, United States, ^7^Michigan State University, Psychiatry, East Lansing, United States, ^8^Global Health Uganda, Kampala, Uganda, ^9^Johns Hopkins University, Bloomberg School of Public Health, Baltimore, United States, ^10^Johns Hopkins University, Pathology and Infectious Diseases, Baltimore, United States

**Background:** Triple ARVs during pregnancy and breastfeeding dramatically decrease the risk of HIV transmission from mothers to infants. However, prolonged antepartum and postpartum exposure to Triple‐ARV prophylaxis may disrupt infant neurodevelopment. The present study evaluates developmental outcomes for HIV‐exposed uninfected (HEU) and unexposed uninfected (HUU) Ugandan and Malawian children enrolled in the IMPAACT PROMISE RCT study.

**Methods:** Pregnant HIV‐infected mothers were randomized to Triple‐ARV prophylaxis (3TC‐ZDV/LPV‐RTV or FTC‐TDF/LPV‐RTV), versus Zidovudine (ZDV). Postpartum, the mother/newborn dyads were randomized to maternal Triple‐ARV or infant Nevirapine (NVP) during breastfeeding. 942 children were enrolled between nine and twelve months of age: 465 were unexposed/uninfected (HUU) (49%) and 454 (48%) were girls. HEU and age‐matched HUU children were enrolled at the two IMPAACT PROMISE study sites: 465 (49%) in Blantyre, Malawi, and 477 (51%) in Kampala, Uganda. Mullen Scales of Early Learning (MSEL) was used for developmental assessment at 12, 24, and 48 months of age, and the Kaufman Assessment Battery for Children (KABC) for cognitive assessment at 48 months only.

**Results: ** Controlling for sex, study site, and age at assessment, there were no significant MSEL differences among PMTCT ante‐ and post‐partum treatment arms at 12 and 24 months (Table 1). There were significant differences among the treatment arms at 48 months for the MSEL composite cognitive score (*p* = 0.04) and Fine Motor scale (*p* = 0.001). For the KABC at 48 months, there were significant differences among the study groups for all the global scales except the nonverbal index. However, the maternal Triple‐ARV (antepartum and postpartum) children were not at a significant disadvantage to the HUU group on any of the pairwise comparisons. Among all significant *p* values in Table 1, only MSEL fine motor remained significant after a Bonferroni adjustment for multiple comparisons. Again, the maternal Triple‐ARV exposed children were not at a significant disadvantage.

**Conclusions: **Both ante‐ and postpartum maternal triple‐ARV exposure did not result in greater developmental or cognitive risk for their HEU children through 48 months of age compared to HUU children. Overall, HUU and HEU children were developmentally comparable. These findings are reassuring as PMTCT programs using maternal ART are widely rolled out in resource‐constrained settings.

**Abstract WEAB0201‐Table 1 of the developmental outcomes for the Mullen Scales of Early Learning and the Kaufman Assessment Battery for Children) (inlinegraphic)**

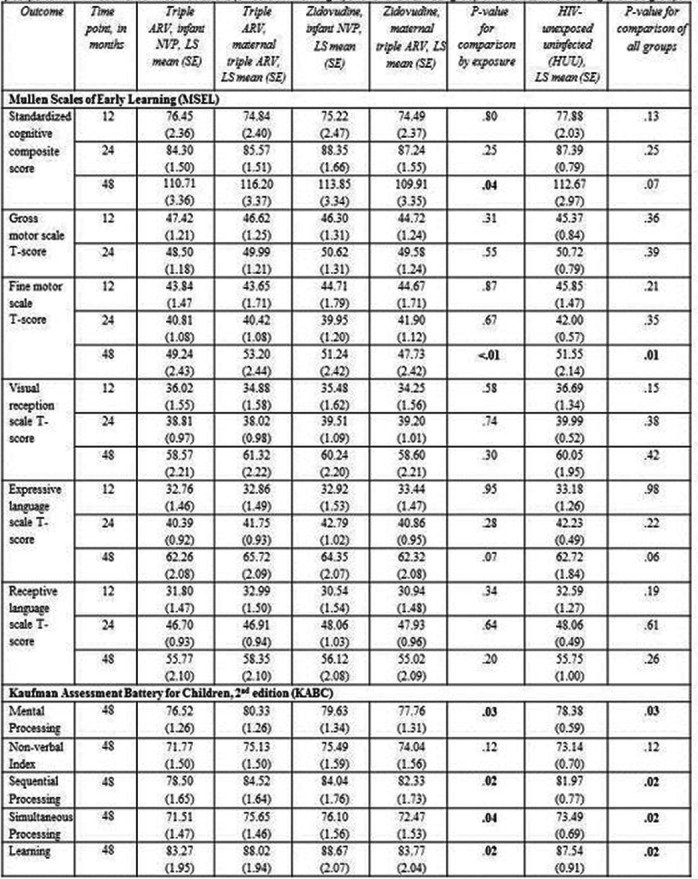

## WEAB0202

### Central nervous system toxicity of efavirenz in HIV‐infected children in Tanzania

**L. Van deWijer^1^; D.N. Mchaile^2^; A.F.A. Schellekens^3,4^; N.N.J. Lambregts‐Rommelse^3^; Q. de Mast^1^; B.T. Mmbaga^5^; A.J.A.M. van derVen^1^ and G.D. Kinabo^2^**

^1^Radboud University Medical Center, Department of General Internal Medicine, Nijmegen, Netherlands, ^2^Kilimanjaro Christian Medical Centre, Department of Pediatrics, Moshi, Tanzania, United Republic of, ^3^Radboud University Medical Center, Department of Psychiatry, Nijmegen, Netherlands, ^4^Nijmegen Institute for Scientist Practitioners in Addiction, Nijmegen, Netherlands, ^5^Kilimanjaro Clinical Research Institute, Moshi, Tanzania, United Republic of

**Background:** The World Health Organization recommends efavirenz as part of the first‐line combination antiretroviral therapy (cART) for HIV‐infected children. Awareness of central nervous system (CNS) side effects in adults is increasing. Reliable data on CNS toxicity in children, however, remain sparse. We compared neuropsychological symptoms, cognitive performance as well as adherence between long‐term treated HIV‐infected Tanzanian children on efavirenz vs. control regimens.

**Methods:** Cross‐sectional observational study among HIV‐infected children (6 to 12 years) on cART for ≥6 months and with viral loads ≤1000 copies/mL in Kilimanjaro, Tanzania. We used the Child Behavior Checklist (CBCL6‐18) to evaluate behavioral and emotional problems. Cognitive performance was assessed using the Raven's Colored Progressive Matrices and the Digit Span test. Non‐adherence was defined as any reported missed doses over the previous three days or <100% adherence since the last clinical visit. Our study was powered to show a group difference of 0.5 SD in CBCL6‐18 total problem scores. MANCOVA and logistic regression were used to assess differences between groups. Analyses were adjusted for age, sex, being treatment naïve, duration of cART, history of TB treatment, parental loss, and HIV disclosure.

**Results: ** One‐hundred‐forty‐one children were enrolled of whom 72 (51%) used efavirenz. Groups did not differ in age, sex, nadir CD4+ or general demographics. We found no differences in the CBCL6‐18 behavorial and emotional problem scores (total/internalizing/externalizing), cognitive performance tests or adherence. Efavirenz‐treated children had lower CBCL 6 to 18 competence scores (*p* = 0.025), which was mainly due to lower scores on school performance with mean (SD) 4.1 (1.4) and 4.7 (0.9) (*p* = 0.001) for efavirenz and controls respectively.

**Conclusions: **Overall, we did not see differences in emotional and behavioral problems, cognitive performance scores or adherence between efavirenz‐treated children and controls, which is in contrast to earlier studies in adults. The lower school performance scores in efavirenz‐treated children, however, warrant further study.

## WEAB0203

### Outcomes of second‐line antiretroviral therapy (ART) in HIV‐infected children: a CIPHER cohort collaboration global analysis

**K. Patel^1^; C. Smith^2^; I.J. Collins^2^; R. Goodall^2^; E. Abrams^3^; A. Sohn^4^; T. Mohamed^5^; R. Van Dyke^6^; P. Rojo^7^; K. Wools‐Kaloustian^8^; J. Pinto^9^; A. Edmonds^10^; I. Marete^11^; M. Paul^12^; H. Nuwaqaba‐Biribonwoha^13^; V. Leroy^14^; M.‐A. Davies^15^; R. Vreeman^16^; Collaborative Initiative for Paediatric HIV Education and Research (CIPHER) Cohort Collaboration Duration of Second‐line Proj**

^1^Harvard T.H. Chan School of Public Health, Epidemiology, Boston, United States, ^2^University College London, MRC Clinical Trials Unit, London, United Kingdom, ^3^Columbia University Medical Center, Epidemiology and Pediatrics, New York, United States, ^4^TREAT Asia/amfAR, Foundation for AIDS Research, Bangkok, Thailand, ^5^Hospital Kuala Lumpur, Paediatric Institute, Kuala Lumpur, Malaysia, ^6^Tulane University School of Medicine, Pediatrics, New Orleans, United States, ^7^Hospital Universitario 12 de Octubre, Pediatrics, Madrid, Spain, ^8^Indiana University School of Medicine, Infectious Diseases, Indianapolis, United States, ^9^School of Medicine, Federal. University of Minas Gerais, Pediatrics, Belo Horizonte, Brazil, ^10^UNC Gillings School of Global Public Health, Epidemiology, Chapel Hill, United States, ^11^Moi University School of Medicine, Child Health and Paediatrics, Eldoret, Kenya, ^12^Baylor College of Medicine, Pediatrics, Houston, United States, ^13^Columbia University, Mailman School of Public Health, ICAP, New York, United States, ^14^Institut de Santé Publique, d'Epidémiologie et de Développement (ISPED), Université Bordeaux, Centre de Recherche Inserm U 897, Bordeaux, France, ^15^University of Cape Town, Centre for Infectious Disease Epidemiology and Research, Cape Town, South Africa, ^16^Indiana University School of Medicine, Pediatrics, Indianapolis, United States

**Background:** There are limited data describing characteristics at second‐line ART initiation and subsequent outcomes among children, particularly in resource‐limited settings.

**Methods:** Data through 2015 on HIV‐infected children aged <18 years initiating ART from 11 cohort networks were pooled. Characteristics at second‐line ART initiation and immunological and clinical outcomes measured at one and two years after initiation were summarized by region: North America, Latin America (Caribbean, Central & South America), Europe, Asia, Southern Africa (South Africa & Botswana) and the rest of sub‐Saharan Africa (SSA). Results were not adjusted for censoring due to loss to‐ or end of‐ follow‐up.

**Results: ** Of 85,389 children who started first‐line ART, 3555 (4%) switched to second‐line, primarily with protease inhibitors (92%). Median (interquartile range (IQR)) age at second‐line ART initiation varied from 4.1 (1.9, 7.5) years in North America to 10.3 (6.7, 13.8) years in Latin America (Table 1). The lowest CD4 counts at second‐line initiation were in SSA and Latin America (235 (81, 561) and 239 (63, 661) cells/mm^3^, respectively).

Overall, the median (IQR) follow‐up after second‐line ART initiation was 29 (12, 51) months, with the shortest follow‐up in SSA (21 (8, 39) months) and the longest in North America (63 (32, 101) months). In the first year after initiation of second‐line ART, observed mortality was higher in Latin America (4.9% (1.8, 10.6)) and SSA (2.8% (2.0, 4.0)) compared to Southern Africa (0.7% (0.3, 1.4)); progression to AIDS was highest in SSA (12.1% (9.4, 15.4)) followed by Asia (4.6% (2.2, 8.4)). Median CD4 counts one year after second‐line initiation improved and were >500 cells/mm^3^ in all regions.

No deaths were observed between one and two years of follow‐up after second‐line ART initiation in North or Latin America, while there were increases in cumulative mortality through two years in the other regions. There were continued improvements in CD4 counts in most regions at two years of follow‐up.

**Conclusions: **We found wide regional variations in age and CD4 count at second‐line ART initation among children. Immunological restoration was observed in all regions after switch to second‐line. However, deaths continued to be observed in some regions through two years of follow‐up.

**Abstract WEAB0203‐Table 1.**

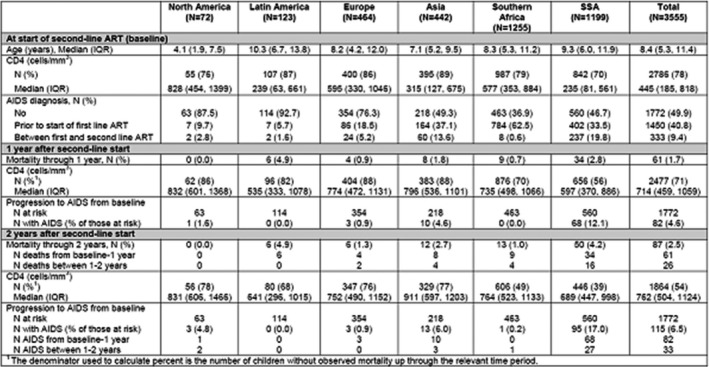

## WEAB0204

### Pellets’ formulation of Lopinavir/ritonavir in children: 48‐week evolution of viral suppression across age categories in the LIVING study

**I. Andrieux‐Meyer^1^; O. Salami^2^; R. Omollo^3^; T. Egondi^3^; M. Waweru^3^; S. Odiambo^3^; M. Wasunna^3^; V. Musiime^4^; E. Obimbo^5^; E. Bukusi^6^; P. Oyaro^6^; J. Mwanga‐Amumpaire^7^; J. Mbuthia^8^; J. Lee^1^; F. Kyhomuhendo^3^; F. Simon^1^; W. Nyandiko^9^; A. Kekitiinwa^10^; M. Lallemant^1^; D. Wamalwa^5^ and LIVING Study Group**

^1^Drugs for Neglected Diseases Initiative, Geneva, Switzerland, ^2^Drugs for Neglected Diseases Initiative, Paediatric HIV, Nairobi, Kenya, ^3^Drugs for Neglected Diseases Initiative, Nairobi, Kenya, ^4^Joint Clinical Research Centre, Kampala, Uganda, ^5^University of Nairobi, Nairobi, Kenya, ^6^Kenya Medical Research Institute, Nairobi, Kenya, ^7^EPICENTRE, Mbarara, Uganda, ^8^Gertrude's Children's Hospital, Nairobi, Kenya, ^9^AMPATH, Moi University, Eldoret, Kenya, ^10^Baylor Centre of Excellence in Pediatric HIV, Kampala, Uganda

**Background:** The pellets’ formulation of LPV/r which is palatable, heat‐stable and easy‐to‐administer has received tentative USFDA approval for use in infants and young children. However, there is a paucity of clinical data on its effectiveness and safety in routine care. The LIVING study is evaluating the effectiveness, safety, PK and acceptability of LPV/r pellets + ABC/3TC (or AZT/3TC) dispersible tablets, in HIV+ children unable to swallow tablets in Kenya and Uganda.

**Methods:** An open‐label, single‐arm, prospective, multi‐centre, phase‐3b implementation study. Inclusion criteria: ARV naïve, on liquid LPV/r‐based or failing NNRTI based ART; Weight ≥3 and <25 kg. ART dosing based on WHO weight bands. Children assessed at baseline, 1 month then 3‐monthly. AEs were graded using DAIDS tables. We evaluated viral suppression across four age categories (months): 5 to 11, 12 to 24, 25 to 48 and ≥49.

**Results: ** As of 31/10/2017, 723 patients had been enrolled, of whom 459 and 303 had reached WK24 and WK48 respectively, with a cohort retention of 88.6% (follow‐up on going, seven deaths,). Baseline and WK 48 VL available in 266 children (136 (51.0%) females, median age 43 months (95% CI 25 to 62), 5 (9.4%) ART naïve, 229 (86.1%) switched from LPV/r syrup, and 12 (4.5%) from NVP based ART.

At baseline, Viral load parameters were as follows: median (IQR) (log10 copies/mL) and proportion with VL<50 and <400 copies/mL across age categories were 4.7 (2.7 to 5.8), 14.0% and 28.5% in the 5 to 11 months, 3.4 (1.7 to 5.3), 25.0% and 45.0% in the 12 to 24 months, 2.1 (1.6 to 4.2) 27.0% and 57.0% in the 25 to 48 months and 2.1 (1.5 to 4.3), 36.0% and 57.0% in the ≥49 months.

At WK48, VL parameters were 1.6 log (1.3 to 2.3), 52.0% and 76.0% in the 5 to 11 months, 1.9 (1.3 to 3.5) 52.5% and 62.5% in the 12 to 24 months, 1.8 (1.3 to 2.4), 49.0% and 80.0% in the 25 to 48 months and 1.5 (1.3 to 2.2) 60.0% and 81.0% in the ≥49 months. 36 children had 103 AEs grade 3/4, 2 leading to treatment stoppage.

**Conclusions: **LPV/r Pellets‐based ART in children is associated with very levels of HIV viral suppression regardless of age at initiation.

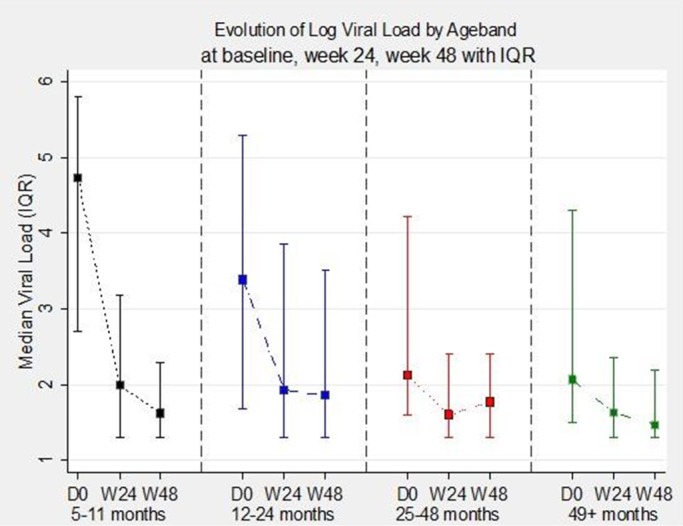

**Abstract WEAB0204‐Figure 1. Viral suppression stratified by age at enrolment.**

## WEAB0205

### Pharmacokinetics, safety, and efficacy of bictegravir/emtricitabine/tenofovir alafenamide (B/F/TAF) single‐tablet regimen in HIV‐1‐infected children (6 to <12 years)

**M. Cotton^1^; A. Liberty^2^; C.A. Rodriguez^3^; K. Chokephaibulkit^4^; P. Kosalaraksa^5^; E. Hellstrom^6^; E. Natukunda^7^; P. Wong^8^; S.R. Majeed^8^; E. Quirk^8^; H. Graham^8^ and C. Pikora^8^**

^1^Stellenbosch University, Tygerberg Hospital, Cape Town, South Africa, ^2^Chris Hani Baragwanath Academic Hospital, Soweto, South Africa, ^3^University of South Florida, Morsani College of Medicine, Tampa, United States, ^4^Siriraj Hospital, Mahidol University, Bangkok, Thailand, ^5^Khon Kaen University, Khon Kaen, Thailand, ^6^Be Part Yoluntu Centre, Western Cape, South Africa, ^7^Joint Clinical Research Centre, Kampala, Uganda, ^8^Gilead Sciences Inc., Foster City, United States

**Background:** Bictegravir (BIC, B), a novel, unboosted integrase strand transfer inhibitor (INSTI) with a high barrier to resistance and low potential for drug interactions, has been coformulated with the recommended NRTI backbone of emtricitabine (F, FTC) and tenofovir alafenamide (TAF) (B/F/TAF) into a once‐daily (QD), single‐tablet regimen (STR). We report pharmacokinetics (PK), safety and efficacy in children who switched from a stable antiretroviral regimen to B/F/TAF.

**Methods:** We conducted a prospective, single‐arm, open‐label, 2‐part, 48‐week (W) clinical trial to evaluate switching to the adult formulation of B/F/TAF (50/200/25 mg) QD in virologically suppressed children (6 to <12 years) weighing ≥25 kg. Intensive PK was evaluated at W2 or W4. PK parameters were compared to B/F/TAF‐treated adults to confirm BIC dose. Adverse events (AE), laboratory tests, HIV‐1 RNA, were assessed. We report follow up data through W12.

**Results: ** 25 children enrolled; median (range) age 10 (6 to 11) years, median (range) weight 28.4 (25.0 to 39.0) kg, 52% female, 64% Black, median CD4 count 928 cells/μL. BIC AUCtau was similar, Cmax 77% higher, and Ctau 22% lower in children ≥25 kg than adults. BIC Ctau was well above protein‐adjusted effective concentration for wild‐type virus (162 ng/mL) in all children. FTC and TAF exposures were within safe and efficacious ranges of adults (table). Through median (Q1, Q3) exposure to study drug of 16.1 (15.9, 17.7) weeks, most common AEs were grade 1 diarrhea and upper respiratory tract infection (each 16%, 4/25); no other AE occurred in >2 participants. No participant discontinued for an AE. All (100%) had HIV‐1 RNA <50 copies/mL at W12; none met criteria for resistance testing.

**Conclusions: **B/F/TAF maintained virologic suppression and was well tolerated in children through at least 12 weeks. Similar to adults, therapeutic plasma concentrations of all B/F/TAF components of B/F/TAF were achieved. Efficacy and safety in children are consistent with phase 3 B/F/TAF results in adults and adolescents, showing high proportions with viral suppression, excellent tolerability, and no resistance. B/F/TAF may be an important unboosted INSTI, STR option for HIV‐infected children due to its small tablet size, high barrier to resistance and lack of food requirement.

**Abstract WEAB0205‐ Table 1. PK parameters of BIC, FTC and TAF after B/F/TAF single‐tablet regimen administration in children and adults**

**Table:** 

	Parameter	Children^a^	Adult^b^	% GLSM Ratio (90% CI)
BIC	AUCtau, ng*h/mL	121,034 (36.4)	102,001 (26.9)	116 (104, 130)
	Cmax, ng/mL	10,989 (28.3)	6146 (22.9)	177 (162, 194)
	Ctau, ng/mL	2367 (78.8)	2610 (35.2)	78.3 (63.4, 96.7)
FTC	AUCtau, ng*h/mL	17,565 (36.9)	12,294 (29.2)	142 (127, 159)
	Cmax, ng/mL	3888 (31.0)	2127 (34.7)	185 (162, 210)
	Ctau, ng/mL	227 (323)	96.0 (37.4)	95.0 (69.9, 129)
TAF	AUCtau, ng*h/mL	435 (94.9)	229 (63.0)	175 (136, 226)
	Cmax, ng/mL	582 (99.9)	277 (62.4)	170 (120, 241)

Parameters are presented as arithmetic mean (% CV); GLSM, geometric least‐squares mean. ^a^n = 25 from intensive PK substudy in current cohort of children (6 to <2 years) weighing ≥25 kg. ^b^From population PK modeling (BIC, n = 1193) or pooled intensive PK (FTC and TAF, n = 74 to 77) data from 4 Phase 3 Studies in HIV‐infected adults Statistical comparisons of the PK parameters in children (test) versus adults from Phase 3 studies (reference) were made using GLSM ratios and associated 90% confidence intervals (CI) with PK equivalence boundary of 70% to 143%.

## WEAC0101

### Reaching out to hidden population in Malaysia: MSMs, transgender and sex workers in Muslims majority setting where sex is a crime

**Y. Md Yusralhakim**

Malaysian AIDS Council, Global Fund, Kuala Lumpur, Malaysia

**Background:** Malaysia has 31 million populations with 61.3% Muslims, Homosexuality and sex out of marriage are illegal and criminalised under both civil and Shariah Laws. Because of this criminalisation, MSM, Sex Workers and Transgender go hidden. In 2016, Malaysia estimated number of MSM is 170,000 and number of transgender remains unknown, however a Delphi‐exercise in 2014 determined that there were 24,000 transgender sex workers and 21,000 female sex workers. MSM saw HIV prevalence rise from 3.9 to 8.9% between 2009 and 2014. Sexual transmission of HIV in general, has recorded a 2.5‐fold increase, accounting for 84% of new HIV infection in 2016 compared to 32% in 2006.

**Description:** Reaching out to MSMs,Transgender, PWID and sex workers is one of the biggest challenges. In response to this, The Malaysian AIDS Council (MAC) implemented the Case Management Program for Key Populations funded by The Global Fund that aims to reach out to these hard‐to‐reach populations and refer them to services at government clinics by using a peer‐to‐peer approach. 85 peer caseworkers were trained to link KP clients to 52 government clinics which been branded as Community Friendly Clinics. The programme involves series of empowerment trainingfor caseworkers, desensitisation workshops for healthcare providers and removing legal barriers advocacy.

**Lessons learned:** Through this programme, from January to December 2017, 10,656 KP clients were reached either through online or venue based approaches, of this number, 5461 went for HIV screening, and 467 were diagnosed positive and 131 has initiated HIV treatment. 56% were from sexual transmission groups (MSM, SW and TG) and of those, 57% Malays (Islam), 18%, Chinese, 11% Indian and 12% others, this never been captured before since sexual transmission took over IDU as the main driver for HIV infection.

**Conclusions/Next steps:** In ensuring the sustainability of HIV response, a continuous creation of enabling environment in every angles for KPs should be in place, peer‐to‐peer and personalised care for KPs should come hand in hand with removing legal barriers and community friendly services that is stigma and discrimination free.

## WEAC0102

### Geosocial networking app use among men who have sex with men in China: findings from the T2T study

**H. Zou^1,2,3^; X. Meng^4^; Y. Lu^2^; Z. Wang^2^; T. Jia^4^; H. Yin^4^; Z. Luo^5^; Y. Ding^5^; W. Chen^5^; S. Huang^6^; H. Zheng^6^; B. Yang^6^; A. Grulich^3^ and The T2T Study**

^1^Sun Yat‐sen University, School of Public Health (Shenzhen), Shenzhen, China, ^2^Sun Yat‐sen University, School of Public Health, Guangzhou, China, ^3^University of New South Wales, Kirby Institute, Sydney, Australia, ^4^Wuxi Municipal Centre for Disease Control and Prevention, Wuxi, China, ^5^Shenzhen Nanshan Center for Chronic Disease Control, Shenzhen, China, ^6^Dermatology Hospital, Southern Medical University, Guangzhou, China

**Background:** Men who have sex with men (MSM) are increasingly using geosocial networking (GSN) mobile applications (app) to socialize in the community. Our study aimed to describe app use among MSM in China to provide reference for app‐based HIV interventions.

**Methods:** MSM aged ≥18 years old who had ≥2 male sex partners were recruited between January and August, 2017 in 3 metropolitan cities: Guangzhou, Shenzhen and Wuxi. A self‐completed tablet‐based questionnaire was collected about socio‐demographic characteristics, sexual behavior characteristics and app use. A blood sample was collected to test for HIV and syphilis, and an rectal swab and urine sample were collected to test for gonorrhea and chlamydia. Anogential warts were checked by a clinician.

**Results: ** A total of 603 subjects were enrolled (mean age = 27.9 years, SD = 7.8). Some 80% of MSM had ever used an app, 76.7% of whom had been using an app for more than 12 months, and 41.6% spent >30 minutes per day on apps. The proportion of app use increased significantly with increasing age, longer time of stay in the study city, higher educational level and more frequent alcohol use (*p < *0.05 for all). MSM engaging in receptive anal intercourse were more likely to use an app over the past 6 months (*p < *0.001). There was no difference in the proportion of patients with or without regular partner who used an app (*p* = 0.348). App users had more sex partners (*p < *0.001), higher usage of Rush (*p* = 0.006) and marijuana (*p* = 0.046), and more HIV tests (*p < *0.001) in the past six months, compared to non app users. Prevalence of HIV (10.7% vs. 7.1%), syphilis (9.9% vs. 5.8%), urethral chlamydia (5.2% vs. 8.3%), rectal gonorrhea (3.5% vs. 2.4%), urethral gonorrhoeae (3.1% vs. 4.1%) and anogenital warts (5.0% vs. 3.3%) were similar in the two groups (*p *>* *0.1 for all). App users had significantly more rectal chlamydia (15.6% vs. 6.6%, *p* = 0.035).

**Conclusions: **The majority of MSM had frequent and long‐term use of GSN app. Partnered status did not affect men's app use behaviors. GSN app should be used as a platform to carry out interventions aimed at reducing HIV and rectal sexually transmitted infections.

## WEAC0103

### Extended risk network testing to find HIV cases among key populations in Ukraine: predicting recruitment of HIV‐positive clients with machine learning

**P. Smyrnov^1^; Y. Sereda^2^; I. Sazonova^1^; K. Boiko^1^; Y. Kuznetsova^1^; K. Slobodianiuk^1^; O. Denisiuk^1^ and S.R. Friedman^3^**

^1^Alliance for Public Health, Kyiv, Ukraine, ^2^Independent Consultant, Kyiv, Ukraine, ^3^National Development and Research Institutes, New York, United States

**Background:** Alliance for Public Health implements extended risk network testing during community‐based outreach to improve HIV case‐finding among key populations. We aimed to develop a prediction model for finding HIV‐positives in the recruitment network using machine learning.

**Methods:** Since 2016, 51,069 people who inject drugs and their extended risk network peers were recruited in 12 regions of Ukraine. Initial recruitment started from HIV positive cases found in harm reduction program. Enrollment criteria for seeds were 14+ years, people who inject drugs, HIV positive rapid test. Participants got coupons to invite their peers defined as an injecting partner, sexual partner, sexual or injecting partner of somebody from the social network who can be also at risk of HIV. Recruitment was stopped if there were two HIV‐negative cases next to each other in a chain. Data on recruitment chains and participants’ characteristics were collected in real‐time using Syrexcloud mobile application. Random Forest machine learning algorithm was used for predicting presence of HIV‐positives within two waves of recruitment among the subset of participants who received coupons (N = 22,236).

**Results: ** Among participants who received coupons, 80% of them (n = 17,872) recruited at least one peer and 35% of them (n = 7678) recruited at least one HIV‐positive participant within two waves of recruitment network. The full random forest model with 17 predictors yielded an accuracy of 84.9% for classification of HIV‐positives and negatives (sensitivity of 83.2% and specificity of 85.8%). The most informative predictors of recruitment of HIV‐positives included size of recruited network for each participant (mean minimal depth (MMD) = 1.33), result of HIV rapid test at screening (MMD = 2.00), region (MMD = 2.12), experience of HIV testing before screening (MMD = 2.78), and age (MMD = 2.95). Sex (MMD = 4.37), group of key population (MMD = 3.97) and marital status (MMD = 3.56) had the lowest contribution to prediction.

**Conclusions: **High level of prediction model accuracy suggests that application of Random Forest machine learning algorithm during recruitment could improve HIV‐positive yield among recruited participants. Further validation of Random Forest prediction algorithm includes its implementation as a decision‐making tool for improving recruitment strategy, such as distributing more coupons to participants with high probability of recruitment of HIV‐positives.

## WEAC0104

### When and why? Timing and determinants of post‐migration HIV acquisition among sub‐Saharan immigrants in France

**A. Gosselin^1,2^; A. Ravalihasy^1,2^; J. Pannetier^1^; F. Lert^1^; A. Desgrées du Loû^1,2^ and Parcours Study Group**

^1^CEPED (Université Paris Descartes, IRD, Inserm), Paris, France, ^2^Institut de Recherche pour le Développement, Marseille, France

**Background:** Mounting evidence has recently highlighted the fact that many HIV‐positive immigrants in Europe acquired their infections post migration, in relation to experiences of social hardship that entail at‐risk sexuality (e.g. transactional). However, the timing of these infections is not known. This study aims to estimate the timing of post‐migration HIV infection among Sub‐Saharan immigrants in France, who are particularly affected by HIV, and to understand the correlates of post‐migration infection.

**Methods:** Life‐event and clinical information were collected in 2012 to 2013 from a random sample of 277 HIV‐infected outpatients infected after arrival in France and 431 patients not diagnosed with HIV, born in Sub‐Saharan Africa and living in the Paris area. The sixth year in France was chosen as the settlement delay based on previous analysis. We assumed HIV infection after six years (i.e. after the settlement period) in France if at least one of the following criteria was fulfilled: (1) HIV diagnosis at least 11 years after the six years in France, (2) at least one negative HIV test in the 6 first years in France, (3) sexual debut after six years in France. Otherwise, time of HIV infection was based on statistical modeling of first CD4 T‐cell count. We assessed the determinants of HIV acquisition after six years in France using multinomial logistic regression models.

**Results: ** Overall, 58% of Sub‐Saharan immigrants who acquired HIV in France had been infected during the first six years in the country (55% of men and 61% of women). Conversely, about 42% of immigrants had contracted HIV after settlement. Factors associated with post‐settlement infection were arrival in France at a younger age (between 18 and 27 years old versus later (OR = 2.40 (1.08 to 5.31)) and arriving with a long‐term permit versus being undocumented (OR = 2.23 (1.12 to 4.43)). Bivariate models showed that post‐settlement infection was associated with occasional and transactional relationships (OR = 1.98 (1.04 to 3.79)) and concurrent partnerships (OR = 1.91 (1.01 to 3.60)).

**Conclusions: **The majority of post‐migration HIV acquisition occurs during the settlement period. Therefore, HIV prevention efforts should target newly arrived immigrants. However, long‐time immigrants are also at risk for HIV, and specific prevention tools and interventions should be directed at this population.

**Abstract WEAC0104‐Table 1. Factors associated with the probability to be infected after settlement (in reference to the non infected group)**

**Table:** 

	Bivariate models		Multivariate models	
	RRR [IC 95%]	*p*	RRR [IC 95%]	*p*
Legal permit at arrival in France				
Undocumented	ref	ref	
Short term permit	1.21 [0.63 to 2.34]	0.557	1.11 [0.62 to 2.00]	0.724
Long term permit or French nationality	2.60 [1.43 to 4.74]	0.002	2.23 [1.12 to 4.43]	0.023
Had at least one casual or transactional partnership after six years in France (ref. No)				
Yes	1.98 [1.04 to 3.79]	0.039	1.49 [0.64 to 3.51]	0.348
Had at least one concurrent partnership after six years in France (ref. No)				
Yes	1.91 [1.01 to 3.60]	0.047	1.31 [0.67 to 2.56]	0.416

## WEAC0105

### Patterns of substance use among young men who have sex with men and their associations with HIV risk behavior and sexually transmitted infections

**M. Newcomb; G. Swann and B. Mustanski**

Northwestern University, Medical Social Sciences, Chicago, United States

**Background:** Young men who have sex with men (YMSM) carry a heavy burden of HIV in the United States. Substance use predicts condomless anal sex (CAS) and HIV incidence, but little is known about patterns of polysubstance use and individual differences in patterns of use, particularly among YMSM under age 21. The current study used longitudinal data to describe patterns of polysubstance use, examine demographic differences in patterns of use, and predict HIV risk across groups of substance users.

**Methods:** We utilized an analytic sample of 601 YMSM aged 16 to 20 from the RADAR cohort study of YMSM in Chicago (N = 1125, age range 16 to 29). Participants completed study visits every six months between 2015 and 2017, at which time we assessed past six‐month substance use and sexual behavior. STI testing was conducted annually. We used latent class analysis (LCA) to empirically derive groups of individuals who tend to use similar substances. We included the most frequently endorsed substances as latent class indicators (binge‐drinking, chronic marijuana use, stimulants, ecstasy, prescription drugs). We then conducted one‐way ANOVA and chi‐square analyses to examine demographic differences in class membership and negative binomial and logistic regression models to predict HIV risk.

**Results: ** LCA revealed four latent classes of substance users. Binge‐Drinkers (N = 166) were more likely to be White and gay‐identified; Binge‐Drinkers and Prescription Drug Users (N = 144) were more likely to be White; Polysubstance Users (N = 70) were more likely to be gay‐identified and the least likely to be Black; and Low Substance Users (N = 221) were the most likely to be Black, younger and bisexual. Polysubstance Users had the highest rates of CAS and STIs concurrently and longitudinally, followed by Binge‐Drinkers and Polysubstance Users, Binge‐Drinkers, and Low Substance Users (all comparisons *p* < 0.05).

**Conclusions: **The YMSM groups reporting use of multiple substances had the highest HIV risk. Polysubstance use has consistently been linked to HIV, but these analyses point to prescription drug use as another important target for HIV prevention among YMSM. Importantly, low substance use and CAS among Black YMSM contrast with their high HIV incidence in Chicago. Patterns of substance use cannot fully explain racial disparities in HIV.

## WEAD0101

### Innovative paralegal and advocacy program enhancing access to justice and harm reduction services in Mombasa county

**H. Tayab^1^; A. Badhrus^1^; F. Jeneby^1^; M. Fahmy^1^; F. Abdulrahman^1^ and M. Handulle^2^**

^1^Muslim Education and Welfare Association (MEWA), Health and Harm Reduction, Mombasa, Kenya, ^2^Mainline, Harm Reduction, Amsterdam, Netherlands

**Background:** Criminalization of drug use, denial of health services, gross human right violations by law enforcers and community increases PWUD risk of premature death and morbidity.

Kenya has an estimated 18,327 People Who Inject Drugs (PWIDs), of whom 8500 are in Coast region. Their HIV prevalence is 18.6% compared to 5.6% for general population.

In 2015, growing threat of terrorism in Kenya triggered Presidential directive to control drugs menace in Mombasa. This led to massive indiscriminate arrests of PWUDs and also threating the safety and security of Outreach workers, thereby triggering disruption in service delivery.

Scarcity of drugs led to shift in to injecting while shortage of HIV preventive commodities resulted it to sharing and unprotected sex.

Over 300 PWUDs incarcerated in three days, some of whom were under HIV, TB, treatment and care and on OST/MAT Program.

**Description:** MEWA initiated Paralegal and Advocacy Program, whereby its staff, Judiciary and law enforcers were trained on provision of harm reduction services, health and human rights of PWUDs in accordance to Kenya Constitution.

MEWA also provided hygiene kits and legal aid, set up support groups and lobbied for alternative sentencing for PWUDs in prison, with assistance to secure national identity cards.

MEWA with Judiciary and Kenyan Prison Services established a framework for improved PWUDs access to justice, fair trials, alternative sentencing and access to MAT.

**Lessons learned:** The Climax of our achievement is the issuance of court order obliging the Prison service, the MAT Clinic and MEWA to ensure accused PWUDs are enrolled on MAT and access all other relevant Harm Reduction services.

A total of 2493 (F:365M:2128) PWUDs reached with biomedical and harm reduction services, 305 (F:53M:252) with legal aid and 194 (F:14M:180) economic empowerment and 99 (F:15M:84) supported to receive identity cards and 5 male children with birth certificates.

Hundreds of PWUDs understand their constitutional rights, can now vote, access revolving funds and freely move without police harassment.

**Conclusions/Next steps:** However, rogue law enforcers and routine public servants transfer undermines effective service delivery.

There is need to scale up Paralegal training to enforce respect of health and human rights of PWUD in Mombasa County.

## WEAD0102

### Paralegal community in the epidemic of injustice: the role of paralegals from drug users communities in the fulfillment of drug users rights

**A. Qisthi**

Persaudaraan Korban Napza Indonesia, Legal Advocacy, Jakarta, Indonesia

**Background:** In the beginning 2015, President Joko Widodo declared the “War on Drugs” as respond to “Drugs Emergency Situation” in Indonesia. This declaration further enhances repressive actions and punitive policies for drug users. the lack of access to justice (access to legal aid) resulted in the increasing number of drug users ending up in prison. This resulted in overloading in prisons, which in turn resulted in further larger impacts. The lack of access to health toward drug users with special needs, for example, the lack of Anti Retroviral Therapy (ART) for drug users living with HIV/AIDS, lack of Opiate Substitution Therapy (OST), and sterile needles has resulted in the deteriorating health of drug users and resulting in deaths from other infectious diseases.

**Description:** In 2017, Persaudaraan Korban Napza Indonesia (PKNI) as a national network of 26 drug user communities spread across the country implement community paralegal program in 10 cities. as an effort in breaking the barriers, community paralegals are giving legal assistance to drug users when they are dealing with the law to promote the access to justice. In addition to legal assistance, paralegals are also trying to meet access to health services for drug users with special needs such as with HIV / AIDS and Hepatitis C, and drug users who require OST. From 145 drug users assisted, there are 133 men and 12 women, and the age average of drug user was 18 to 35 years old. Paralegals have a role to minimize the use of a criminal approach. During the program execution of 53 completed paralegal cases succeeded in stopping criminal proceedings in 37 cases with details of 19 cases declared free in the absence of evidence and 13 cases successfully stopped by encouraging the authority of the investigator to place the client into the rehabilitation site.

**Lessons learned:** The existence of paralegals contributes significantly to the fulfillment of the right to justice and the right to health of drug users.

**Conclusions/Next steps:** Community paralegal must remain sustainable, surely with the increasing role and number of paralegals in the region in Indonesia as the front guard of primary justice.

## WEAD0103

### Street lawyers from the harm reduction project at the ARF and their contribution to the fight for the right to health of PWUD and people living with HIV

**M. Malyshev^1,2^**

^1^Andrey Rylkov Foundation for Health and Social Justice, Harm Reduction, Street Social Work, Moscow, Russian Federation, ^2^Tver Government University, Tver, Russian Federation

**Background:** The street lawyers program offers social support and rights protection to PWUD and people living with HIV – based on the only harm reduction project in Moscow, a city of 12 million.

**Description:** Several years after the launch of the Harm Reduction Moscow project, the problem of stigmatization of drug users and people living with HIV in various spheres of public life in Russia has become more pressing. As a result, rights violations, denial of medical care and criminal prosecution became widespread. To counteract that, a project was launched in 2013 to provide legal assistance and support.

Street lawyers are social workers engaged in outreach work and social support who also motivate representatives of vulnerable groups to protect their rights and dignity.

The work of street lawyers includes several consecutive stages:

● Informing

● Mediation

● Official appeals to the authorities

● Judicial protection.

In 2017, 651 clients received legal advice, 149 violations of rights were documented, a total of 58 appeals to the authorities were filed and 44 clients achieved the desired result.

**Lessons learned:** Four years of work have shown that social workers play a key role in protecting the rights of vulnerable groups. They motivate, offer support and provide a link between lawyers, legal knowledge in general and stigmatized groups. During the course of the project, an algorithm was developed to protect the rights of clients, which proved an effective model of interaction between representatives of vulnerable groups, social workers and lawyers. Important and successful cases are those where the client feels motivated to resist stigmatization and stand up for their rights when making decisions and acting on them.

**Conclusions/Next steps:** The project showed that the most effective way to provide legal assistance to vulnerable groups is to link clients with social workers and lawyers, where the main role for the protection of rights lies with the client and the social worker. This approach helps achieve positive results in rights protection at the same time empowering clients and the community. That's why we recommend this approach for use in other projects aimed at protecting the rights of of PWUD and PLHIV.

## WEAD0104

### A novel method of working with judges to build their capacity on HIV and human rights in Africa

**D. Patel^1^; P. Patel^2^; A. Saha^1^; C. Grant^3^ and D. Owolabi^1^**

^1^UNDP, Istanbul, Turkey, ^2^Consultant, Istanbul, Turkey, ^3^Consultant, Johannesburg, South Africa

**Background:** In 2014, judges in Africa with the United Nations Development Programme (UNDP) created the Regional Judges’ Forum for Africa. The goal of the Forum, led by a small number of senior judges from African countries, was to develop the HIV/TB and related human rights expertise of a core group of judges, who would then sensitise fellow judges nationally and regionally, and support institutionalizing this expertise.

**Description:** The Forum has met annually since 2014, created a database to provide judges with relevant reference materials, and developed an online space for the judges to communicate and share information. Annual meetings cover many topics, including the rights of key populations. Each topic includes discussion of the relevant scientific information; personal experiences from members of the affected population, and latest judicial rulings. The sessions consist of presentations, with time allotted for discussion among the judges. Resource people include HIV/TB clinicians and scientists, PLHIV, members of key populations, lawyers, and judges.

The number of judges in the Forum and the countries represented in the Forum increased from 11 to 33 and from 8 to 19, respectively.

**Lessons learned:** Such Forums should be convened and run by judges with the support of non‐judicial organisations. Judges were most interested in learning about the developments in HIV‐related jurisprudence; the science related to HIV and TB and their treatments; and hearing directly from key and vulnerable populations about their lived realities.

The Forum resulted in numerous, rights‐respecting judicial decisions; training of judicial officers by members of the Forum; increased personal awareness and development on key HIV legal issues; greater judicial access to reference materials; a stronger relationship between judges and civil society; and the organisation of judicial oversight of prisons to address overcrowding.

**Conclusions/Next steps:** To ensure sustainability, UNDP and the South African Judicial Education Institute (SAJEI) are working to institutionalise the HIV‐related judicial capacity building within the SAJEI and other national judicial training institutions.

This model is a low‐cost method for effectively building the capacity of judges, and it can and should be replicated in other regions.

## WEAD0105

### Assessing HIV key populations’ participation in seeking justice to test the effectiveness of human rights redress mechanism in Indonesia

**A. Wirya and F. Aotari**

LBH Masyarakat (Community Legal Aid Institute), Jakarta, Indonesia

**Background:** Indonesia has established several redress mechanisms to address human rights violations; many of which are relevant to the problems faced by HIV key populations. However, the effectiveness of these systems is still in questions. This research documented human rights violations withstood by HIV key populations (people living with HIV, drug users, sex worker, and men who have sex with men) and their efforts to seek justice in order to understand the potentials and weaknesses of utilizing Indonesian redress mechanism.

**Methods:** This research used quantitative data collected by thirty enumerators in seventeen districts in Indonesia. They were selected from HIV key population groups who had received training on human rights, redress mechanism, and documentation method. They were tasked to document human rights violations in their districts which took place between January 2016 and October 2017 using structured interview. In total, this research documented 151 human rights violations.

**Results: ** Only 46 out of 151 victims seek remedy after violations occurred (30.46%). Thirty of them (65.22%) reported their cases to non‐governmental organizations (NGO), where twenty eight reports were then followed up to the relevant state agencies by asking clarification, condemning the abuse, reprimanding the perpetrators, or demanding compensation. Seven victims directly complained to the institution whose employees violated their rights. The rest sought help to independent governmental bodies, such as Provincial Health Department, Provincial AIDS Commission, and police. Around 39.13% of victims felt satisfied, 39.13% felt unsatisfied, and 21.74% felt neutral toward the result. Victims are disappointed because their cases which were not followed up seriously, the perpetrator were not caught, or they were unable to gain remedy. Victims are satisfied when they got information about the follow‐up, they could access antiretrovirals which they were unable to obtain before, and discriminations ceased.

**Conclusions: **Key populations who experienced human rights abuses mostly did not pursue remedy or protection. They believed that redress would be useless and even harmful – a belief that is proven true by more than one third of victims who sought justice. However, many victims were satisfied with NGOs which did not hesitate to reprimand the government and demand compensation.

## WEAD0201

### The association between incarceration and transactional sex among HIV‐positive young men who have sex with men (YMSM) in the United States

**M. Philbin^1^; E. Kinnard^1^; A. Tanner^2^; S. Ware^2^; B. Chambers^3^; A. Ma^4^ and J.D. Fortenberry^5^**

^1^Columbia University Mailman School of Public Health, Sociomedical Sciences, New York, United States, ^2^University of North Carolina Greensboro, Greensboro, United States, ^3^University of California, San Francisco, United States, ^4^Southern Illinois University, Edwardsville, United States, ^5^Indiana University School of Medicine, Indianapolis, United States

**Background:** Criminal justice practices in the United States disproportionately affect sexual and racial/ethnic minority men, who are at higher risk of incarceration. Previous research demonstrates associations between incarceration and sexual risk behaviors for men who have sex with men (MSM). However, little of this work focuses on young MSM (YMSM), particularly HIV‐positive YMSM, even though one‐third report having ever engaged in sexual risk behaviors such as transactional sex. We therefore explored the association between incarceration and transactional sex among HIV‐positive YMSM.

**Methods:** As part of the CATCH study, we recruited 97 HIV‐positive YMSM across 14 Adolescent Trials Network clinical sites from August 2015 to February 2016. Following consent, youth completed an ACASI survey on psychosocial characteristics and medical/behavioral history. We used multivariate logistic regression to examine the association between incarceration (i.e., ever been in jail/prison) and transactional sex (i.e., ever exchanged sex for money or drugs) among YMSM; the Minority Stress Model informed control variable selection.

**Results: ** The majority of YMSM were 24 years old (78%), identified as racial/ethnic minority (95%), not in school (54%), single (74%), and earned <$12,000/year (67%); nearly half had ever been homeless (41%). Additionally, 42% had been incarcerated and 28% had engaged in transactional sex. In the multivariate model, having ever been incarcerated remained independently associated with having engaged in transactional sex (aOR = 3.20; 95% CI: 1.07 to 9.63). Being 24 years old versus younger (aOR = 9.68; 95% CI: 1.42 to 65.78) and having ever been homeless (aOR = 3.71; 95% CI: 1.18 to 11.65) also remained independently associated with having engaged in transactional sex.

**Conclusions: **Incarceration may represent a particularly severe stressor for young men with multiple marginalized identities—HIV‐positive, MSM, and racial/ethnic minority—and put them at higher risk for engaging in transactional sex. Tracing the relationship between incarceration and transactional sex highlights a potential key source of health disparities among HIV‐positive YMSM; it also identifies important targets for subsequent intervention studies—e.g., housing, mental health, employment—that place this population at risk of incarceration and transactional sex. Facilitating HIV‐positive YMSM's engagement with community‐level and HIV clinic‐specific services may serve as a key strategy to promote health and mitigate harms related to incarceration and transactional sex.

## WEAD0202

### Gendered powerlessness in at‐risk adolescent and young adult women: an examination of condom use behavior

**D. Chiaramonte^1^; R.L. Miller^1^; C.B. Boyer^2^; K.S. Lee^3^; O.J. Santiago‐Rivera^4^ and Adolescent Medicine Trials Network for HIV/AIDS Interventions**

^1^Michigan State University, Psychology Department, East Lansing, United States, ^2^University of California, Department of Pedatrics, San Francisco, United States, ^3^Michigan State University, University Outreach and Engagement, East Lansing, United States, ^4^Michigan State University, East Lansing, United States

**Background:** The burden of HIV among adolescent women remains unacceptably high. Although researchers have established that adult women's social powerlessness influences their ability to negotiate the use of condoms effectively, researchers have seldom examined this effect using multidimensional higher‐order models or among adolescents. Informed by Connell's Theory of Gendered Powerlessness, we derived a developmentally appropriate model of gendered powerlessness for adolescents. Connell proposes three components of powerlessness: cathexis (e.g., gendered social norms and expectations), sexual division of power (e.g., gendered subordination in sexual situations), and division of labor (e.g., gendered economic subordination).

**Methods:** Anonymous ACASI surveys were administered in community venues in 14 U.S. cities to at‐risk adolescent women aged 12 to 24 years (N = 1101). The young women in this sample were primarily Black (82%) and heterosexual (87%). We used exploratory and confirmatory factor analyses to determine the dimensionality of gendered powerlessness and structural equation models to examine its association to condom use.

**Results: ** Three latent factors (financial independence, sexual division of power, structure of cathexis) provided an optimal fit to the data (ppp = 0.118 with 95% CI (−25.06, 107.63)). Controlling for age, sexual division of power and structure of cathexis were negatively associated with using condoms with main partners (ppp = 0.135 with 95% CI (−32.84, 113.75)); as powerlessness increased, as indicated by the sexual division of power and structure of cathexis, condom use declined.

**Conclusions: **We find general support for Connell's theory of gendered powerlessness among adolescents. Our results provide convincing evidence for a three‐component model of gendered powerlessness for adolescent women and that two of these components are highly relevant to predicting their condom use with their primary sexual partner. Prevention efforts targeting at‐risk adolescent women would benefit from overturning sexual divisions of power and countering the structure of cathexis that undermines young women's sexual agency.

## WEAD0203

### Ethnographic study for HIV prevention reveals a typology consisting of five distinct types among South African adolescents and young women

**K. Crowley^1^; J. Piper^2^; J. Cummins^2^; G. Gao Guodong^3^; L. Burn^4^; K. Igumbor^5^; R. Agarwal^6^ and F. Veronese^7^**

^1^University of Maryland Robert H. Smith School of Business, Center for health Information and Decision Systems, College Park, United States, ^2^National Institute of Allergy and Infectious Diseases, Division of AIDS, Prevention Science Program, Rockville, United States, ^3^University of Maryland Robert H Smith School of Business, Center for Health Information and Decision Systems, College Park, United States, ^4^KantarTNS, Cape Town, South Africa, ^5^Kantar TNS, Cape Town, South Africa, ^6^University of Maryland Robert H. Smith School of Business, Center for Health Information and Decision Systems, College Park, United States, ^7^National institute of Allergy and Infectious Diseases, Division of AIDS, Prevention Science Program, Rockville, United States

**Background:** The uptake of HIV prevention products among adolescents and young women in Sub‐Saharan Africa has been problematic. Suboptimal product use in recent prevention trials highlights the challenges for conferring protection to targeted users. It has become clear that current methods of assessing acceptability are not useful at predicting actual use by this population. New approaches are needed to better understand the sociocultural context of young women in Sub‐Saharan Africa.

**Methods:** We conducted a detailed ethnographic study among low income South African girls and women between the ages of 14 and 25 in three urban and peri‐urban locations (Living Standards Measure 4 to 7). Data were collected for each participant through a qualitative study consisting of interviews with 11 industry experts, 32 depthnographic interviews and observations with low income females across both in‐home and out of home contexts, 8 auto‐ethnographic interactions (including digital and social media autobiographies), 12 hostess focus groups and influencer in depth interviews, and finally six partner in‐depth interviews. Data were analyzed using qualitative methods including discursive and thematic analysis.

**Results: ** Our data revealed a psychographic typology derived out of the complex dynamic between female vulnerability and socio‐economic privilege:”The Good Girl,” “Responsible Mother,” “Material Girl,” “Protection Savvy,” and “Gender Prisoner.” Each type carries a distinctive psychographic profile, home life, critical events related to sexual activity and family life, and relationship statuses. Together, the unique attributes that define a type are associated with different forms and levels of vulnerabilities to sexually transmitted infections. The data also suggest that each type may require differentiated and targeted education reflecting the variability across the typology with regards to sexual education, partners, products, and influences.

**Conclusions: **Current HIV prevention product promotion strategies have generally adopted a “one‐size‐fits‐all” approach that does not include a nuanced understanding of the context, needs, and motivations underlying the behavior of different user types. Development of typologies for target populations may allow a more targeted approach for designing, marketing, and promoting HIV prevention products to adolescents and young women, and their partners.

## WEAD0204

### Should I take PrEP? A mental models assessment of young African women's motivations for and barriers to PrEP initiation and adherence

**N. Argo^1^; T. Krishnamurti^2^; B. Fischhoff^1^; L.‐G. Bekker^3^; S. Delany‐Moretlwe^4^; E. Bukusi^5^; L. Myers^3^; J. Imrie^6^ and J. Odoyo^7^**

^1^Carnegie Mellon University, Engineering and Public Policy, Pittsburgh, United States, ^2^University of Pittsburgh, Pittsburgh, United States, ^3^Desmond Tutu HIV Foundation, Cape Town, South Africa, ^4^3Wits Reproductive Health and HIV Institute, Johannesburg, South Africa, ^5^Kenya Medical Research Institute, Nairobi, Kenya, ^6^Wits Reproductive Health and HIV Institute, Johannesburg, South Africa, ^7^Kenya Medical Research Institute, Nairobi, South Africa

**Background:** Young women in sub‐Saharan Africa have among the highest HIV incidence rates globally, yet in previous trials they have exhibited some of the lowest PrEP uptake and adherence. What are the motivations for and barriers to PrEP usage for young African women? How can such knowledge be used to effectively design scalable PrEP demonstration projects?

**Methods:** Employing the mental models methodology, our team conducted surveys with 15 experts to characterize young women's decision making about initiation and adherence to PrEP, followed by in‐depth interviews with 48 young women (age 16 to 25) and 45 men (age ≥18 p) in Kisumu, Kenya and Cape Town and Johannesburg, South Africa. Interviews were coded, theme frequencies were calculated, and linked themes were diagrammed into “mental models.” Expert and women's models were compared. Lastly, a follow‐up survey (n = 444; 243 F, 201 M) was conducted at each field site to establish prevalence of beliefs and attitudes identified in the interviews and identify demographic correlates to those beliefs.

**Results: ** While the expert mental models focused on more rational decision themes such as PrEP's role in health outcomes, the young women's mental models were driven by present bias (valuing the costs and benefits of now), particularly pertaining to relationships and influenced by affect or feelings. Overall, women rated the benefits of taking PrEP (e.g., feelings of safety, individual and community empowerment, health) as more influential on their decision to try PrEP than potential costs (side effects, clinic visits, relationship uncertainties, and daily pill‐taking). Factors predictive of women's interest in PrEP included: living in Cape Town, previous knowledge of PrEP, the woman's perceived risk of HIV in the next year, PrEP efficacy beliefs, and the expectation that she would use a condom less if she takes PrEP.

**Conclusions: **PrEP messaging should highlight the immediate and positive emotional benefits of PrEP usage. Findings support the creation of a decision tool that would provide women with personalized recommendations based on risk and preferences, allowing them to answer the question of whether PrEP would be good for them before introducing questions about how it will work in their life.

## WEAD0205

### Experiences and outcomes of group psychotherapy as an antiretroviral adherence support intervention among young people failing on ART at Newlands Clinic, Harare, Zimbabwe

**B.M. Kasimonje^1^; T. Shamu^2^ and C. Chimbetete^2^**

^1^Newlands Clinic, Mental & Social Health, Harare, Zimbabwe, ^2^Newlands Clinic, Harare, Zimbabwe

**Background:** Adherence to antiretroviral therapy (ART) is a major challenge faced by young people living with HIV (YPLHIV). We examined the experiences and outcomes of an Enhanced Adherence Counselling Group Intervention (EACGI) prior to regimen switch among adolescents failing first line ART.

**Description:** We analysed records for (YPLHIV) aged between 13 and 25 years with confirmed virological failure (VF) who were invited to EACGI. This intervention was a 12‐week curriculum of weekly 1.5‐hour sessions which accommodated 8 to 15 people per group. The aim of the intervention was to facilitate readiness to switch treatment to second line ART, and improve adherence through Phenomenological, Motivational Interviewing and Cognitive Behavioural Therapy principles. Each participant had an HIV viral load (VL) test pre and post EACGI and at three, six, nine and twelve months post switch to assess virological outcomes.

**Lessons learned:** Fifty‐nine patients (57.6% female, n = 34) were followed up for 46.8 person‐years. Median duration of first line ART was six years (IQR: 4 to 8) at time of invitation to EACGI. Twenty‐two patients (37.3%) did not attend a session, 8 were female and 14 were male. The most common reasons for not attending were lack of interest and school or work schedules. The main reasons for poor adherence among the 37 attendees were hopelessness, family dysfunction, lack of illness, an aversion to a daily routine attached to stigma, and medication side effects. Among patients who attended >75% of sessions, 76%, 94%, 94% and 100% achieved viral suppression (VL<50 copies/mL) at three, six, nine and twelve months, respectively, compared to 50%, 55%, 55% and 50% among those who attended at least one but ≤75%. Those who did not attend any session had suppression rates of 32%, 41%, 41% and 39%, respectively.

**Conclusions/Next steps:** Patients who attended >75% EACGI had better second‐line virological outcomes compared to those who attended less or none in this small cohort. EACGI is a promising tool for preparing patients within this key population for second line treatment, increasing the likelihood for improved adherence and improved treatment outcomes for second line therapy.

## WEAD0301

### A political economy of HIV treatment policy: drivers of health policy diffusion

**M. Kavanagh^1^; S. Gupta^2^ and K. Parish^3^**

^1^Georgetown University, O'Neill Institute for National & Global Health Law, Washington, United States, ^2^International Association of Providers in AIDS Care, Delhi, India, ^3^University of Pennsylvania, Political Science, Philadelphia, United States

**Background:** Why do some countries rapidly adopt policies suggested by scientific consensus while others are slow to do so? HIV treatment is a particularly salient case in point. Scientists, physicians, and the World Health Organization (WHO) spend significant effort identifying the optimal standards of medical care—yet the guideline policies that govern public health and medical practice often lag far behind evidence. Efforts to address differences in adoption of “evidence‐based” policy focuses on variation in the capacities for adoption and interpretation of scientific evidence. We challenge this view, hypothesizing that socio‐political institutions are often decisive in how quickly countries will adapt science into policy.

**Methods:** We used a mixed methods strategy. First, we constructed a database of national HIV treatment guidelines, collecting 290 published national ART guidelines for adults and adolescents from 122 countries (98% of global HIV burden). Using this database, we built a model to reflect the epidemiologic, economic, and political context and used a Cox‐Proportional Hazards Model to test our hypothesis. Second we conducted interviews with 24 key informants with direct knowledge of guidelines processes to establish causality in our study.

**Results: ** Neither HIV prevalence nor national wealth is a significant driver of policy change in our quantitative or qualitative results. Qualitative analysis shows neither interpretation of medical evidence nor formal cost‐benefit analysis explain differences. Instead, the formal structures of government and the degree of ethnic cleavages predict the speed with which new medical science is translated into policy. More veto points in government is strongly associated with faster policy adoption—with a full swing in the data associated with a 275% increase in adoption speed. HIV policy change is slower in contexts with complex racial/ethnic divisions—as much as 60%, *ceteris paribus*.

**Conclusions: **Our findings challenge expectations in scholarship and practice that policy divergence and inequities are primarily addressed through greater evidence and dissemination channels. That political factors are systematic, rather than random, suggests a new approach is needed by agencies such as the WHO and UNAIDS. Building diffusion strategies, messages, and policy networks that are tailored to national political context is possible when systematic socio‐political factors are identified.

## WEAD0302

### Using TRIPS‐flexibilities as a leverage to improve access to HIV and hepatitis C medicines in Ukraine

**S. Kondratyuk**

All‐Ukrainian Network of People Living with HIV/AIDS, Advocacy Team, Kiev, Ukraine

**Background:** Significant treatment gaps persist for HIV (63%) and hepatitis C (>99%) in Ukraine. High prices for patented ARVs and DAAs contribute to inefficiency of country HIV and hepatitis C response.

**Description:** To ensure better affordability of HIV and hepatitis C medicines, the All‐Ukrainian Network PLWHA (the ‘Network’) used patent oppositions and compulsory licensing (CL) request as leverage in negotiations with patent holders. The national HIV program experienced a funding gap in HIV medicine procurement in 2016.

In February 2016, in response to potential drug shortages the Network organized a meeting with the Ministry of Health and originators (Abbvie, GlaxoSmithKline, MSD, and Janssen). Network shared information about treatment gap, called the companies to lower the prices and called government consider using CL. As the response to this action, ViiV included Ukraine in the MPP DTG license (April 2016). In addition, the Network requested GSK/ViiV to include Ukraine in the license for ABC for adults in May 2016. The negotiations with GSK were conducted simultaneously with preparation of a patent opposition on ABC.

The Network repeatedly emphasized to MSD and GSK representatives that the patent monopolies on TDF/FTC/EFV, ABC and ABC/3TC are unjustified, as the patents do not comply with patentability requirements and were already opposed in other countries. All these efforts may have encouraged MSD and GSK to provide the Network with a patent rights non‐enforcement letters in May to July 2016 and procurement of generic products began in 2017. This has reduced the price by 83%, 59% and 56% on TDF/FTC/EFV, ABC and ABC/3TC respectively, generating annual savings up to 13,9 mln USD.

Successful pre‐grant patent oppositions in relation to SOF filed by the Network and consequent temporary generic competition for SOF may have encouraged originator to add Ukraine to the VL and caused price decrease for 77%.

**Lessons learned:** When patent holders provide non‐enforcement letters or include country in VL, it causes significant price reduction as it creates an opportunity for generic competition.

**Conclusions/Next steps:** Patients′ organizations work on patent oppositions and compulsory licensing is a powerful leverage in efforts to improve access to HIV and hepatitis C treatment.

## WEAD0303

### America's $10 billion overspend on sofosubvir‐based hepatitis C treatment resulting from unmerited patents

**A. Pattillo; J. Fortunak; D. Ravicher; M.H. Hughey; T. Amin and P. Radha Krishtel**

Initiative for Medicines, Access and Knowledge, New York, United States

**Background:** About one quarter of the estimated 1.2 million HIV‐infected persons in the United States are also infected with Hepatitis C virus (HCV). Co‐infection of HCV is especially common (50% to 90%) among HIV‐infected injection drug users. With the introduction of direct‐acting antiviral sofosbuvir (Sovaldi™, SOF) in 2013 and additional SOF‐based combination therapies since, the cure for HCV became reality. To date, SOF‐based therapies have accounted for approximately three out of four of all DAA prescriptions written for HCV patients in the U.S. However the high price of the SOF‐based therapies has resulted in payer rationing and limited access to the medicines, particularly among HCV patients on Medicaid and those in the correctional systems.

**Methods:** Our team of patent lawyers, scientists and health market experts

a) examined the patent portfolio of sofosbuvir,

b) analyzed the commercial market dynamics for sofosubivr‐based products,

c) assessed the current landscape of patient access to HCV medicines in the U.S., and

d) modeled the projected overspend on SOF‐based products in the U.S.

**Results:**

1 Nearly all (26 of the 29) of the patents on Sovaldi™ are secondary patents that relate to various prodrugs, processes, and crystalline forms that are distinct from the base compound sofosubvir. These along with the three patents on the base compound were analyzed by technical experts and found to be likely unmerited given obviousness and prior art in the field.

2 The average net price paid in the U.S. across all SOF‐based products by public and private payers from Q316 to Q317 was $40,200. Our analysis suggests that net prices will continue a downward trend toward $30,000 for 2018.

3 85% of patients in the U.S. that have been diagnosed with HCV will not get access to treatment in the coming year.

4 U.S. payers are projected to spend an excess of $10 billion for branded SOF‐based products in 2021 to 2034 as compared to treatment costs with generic sofosbuvir.

**Conclusions: **Unmerited patents have been identified on Sovaldi™ resulting in prolonged exclusivity periods that prevent generic product entry, burden payers with billions in overspend, and generate significant access problems for HCV patients in the U.S.

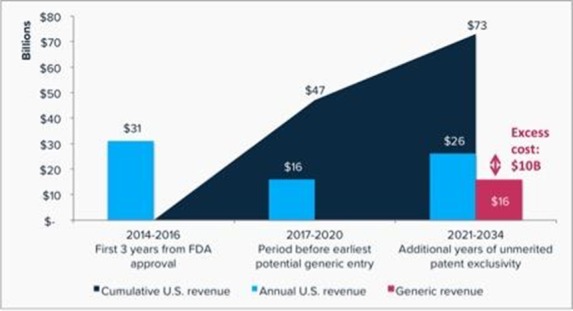

**Abstract WEAD0303‐Figure 1. Summary of U.S. revenue from Sovaldi^®^‐based combination drugs and estimated excess costs for branded versus generic products**

## WEAD0304

### Dolutegravir and the universal antiretroviral regimen: good may be the enemy of perfect

**R. Granich^1^; S. Gupta^2^; L. Kosicki^3^; B. Williams^4^ and M. Ruffner^5^**

^1^Senior Public Health Consultant, San Francisco, United States, ^2^Independent Consultant, Delhi, India, ^3^SGAC/PEPFAR, Washington DC, United States, ^4^SACEMA, Stellenbosch, South Africa, ^5^SGAC/PEPFAR, Washington, United States

**Background:** Tenofovir, Lamivudine and Dolutegravir (TLD) is the best potential global universal treatment regimen. Dolutegravir's tolerability, durability, effectiveness, simplicity, cost‐savings and high resistance barrier could accelerate progress towards 90‐90‐90.

**Methods:** We searched Internet, PubMed, national surveillance reports, UNAIDS/WHO reports, President′s Emergency Plan for AIDS Relief (PEPFAR) 2017 operational plans, and conference abstracts for nationally representative information regarding DTG. We describe policy/implementation status for the 94 low‐ and middle‐income countries (LMICs) with published national guidelines (92% global HIV burden). We compare PEPFAR 2019 and 2018 to 2019 cost savings for Tenofovir‐Lamivudine‐Efavirenz (TLE) 600 vs. TLD for people living with HIV on ART for 16 PEPFAR supported countries in sub‐Saharan Africa. A similar comparison was done assuming achievement of 90‐90‐90.

**Results: ** DTG is already widely used in the higher income settings and increasingly a first‐line drug in LMICs (Table 1). DTG is recommended in all of the high‐income countries as part of an optimal first line regimen, as an alternate option in WHO 2015 guidelines, and in 17 LMICs including Botswana, Lesotho, Nigeria, Uganda and Zambia. Despite not having formal published recommendations, DTG is already being procured in Kenya, Zimbabwe and other LMICs. Although 2018 transition to TLD will likely be incomplete, transitioning all patients supported by PEPFAR in 16 countries in sub‐Saharan Africa to TLD ($75 pppy) when compared with current TLE costs ($79 pppy), could save $63,107,532 and $175,925,064 for 2020 and 2018 to 2020, respectively. Assuming achievement of the 90‐90‐90 target, cost‐savings for 2020 for the 16 countries would be $42,411,600. DTG and alternative service delivery models (e.g., reduced clinic visits, laboratory testing and viral load costs), could reduce overall treatment costs to well below $200 per person per year in Sub‐Saharan Africa. Cost savings that would accompany the decrease in the development and transmission of resistant virus are likely to be even greater.

**Conclusions: **Rapidly transitioning from current 1st line treatment to the DTG universal regimen represents an opportunity to make a major impact on the epidemic of HIV if done swiftly. Other disease control efforts have made concerted efforts across multiple regions and countries when faced with the need for rapid action, why not HIV?

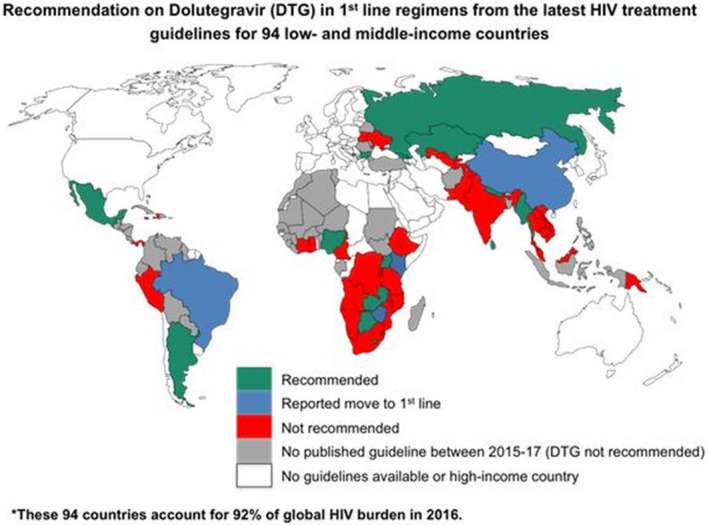

**Abstract WEAD0304‐Figure 1. Global Dolutegravir 1st Line Recommendations for 94 Countries.**

## WEAD0305

### Review of national guidelines in 16 sub‐Saharan African countries for inclusion of the adolescent HIV care continuum

**J. Kottutt^1^; E. Rivadeneira^2^ and S. Hrapcak^2^**

^1^Emory University, Rollins School of Public Health, Hubert Department of Global Health, Atlanta, United States, ^2^Centers of Diseases, Control and Prevention, Division of Global HIV and TB, Center for Global Health, Atlanta, United States

**Background:** Adolescents living with HIV (ALHIV) have poorer HIV‐related outcomes than adults due to unique developmental, psychosocial, and clinical needs that are often not addressed. The 2016 WHO consolidated HIV guidelines recommend provision of adolescent‐friendly HIV services to improve these poor outcomes. The purpose of this review was to assess national HIV/ART guidelines from sub‐Saharan African countries for inclusion of recommendations targeting services for ALHIV along the HIV cascade.

**Methods:** National ART/HIV guidelines from PEPFAR‐supported countries in sub‐Saharan Africa that have pediatric/adolescent HIV programs were reviewed. All documents were publically available and in English. Pertinent information on five areas (Table 1) were abstracted by two authors (JK and SH) and inconsistencies resolved.

**Results: ** Data were available and abstracted for 16 countries (Table 1). Kenya was the only country whose guidelines included all five recommendations. Recommendations on frequency of HIV testing in sexually active adolescents were included in 50% of countries (n = 8); however, the recommended frequency was not consistent (annual testing n = 6, every six to twelve months n = 1, every three to six months n = 1). Although 14 countries (88%) included a section on disclosure to children/adolescents, only 8 (50%) included recommended age of full disclosure. The age varied across countries: 8 to 10 years (n = 1), ≥10 years (n = 3), 10 to 12 years (n = 1), 10 to 14 years (n = 1), 11 to 14 years (n = 1), and 13 years (n = 1). Age of consent for treatment initiation was clearly defined in only 3 countries (19%), all recommending 12 years old. The majority (n = 15; 94%) included recommendations on frequency of viral load (VL) testing, with 9 (56%) following WHO recommendations for testing at six and twelve months after ART initiation, then annually; however, 5 (31%) recommended more frequent VL testing every six months in ALHIV due to higher rates of virologic failure. 44% (n = 7) included guidance on transition to adult services, although only 25% (n = 4) included recommended age of transition which varied from 15 to 20 years.

**Conclusions: **There is currently a lack of consistency in national HIV/ART guidelines to address the unique needs of ALHIV. More specific guidance should be included in national guidelines for healthcare workers to provide appropriate adolescent‐friendly HIV services to improve clinical outcomes in ALHIV.

**Abstract WEAD0305‐ Table 1. Inclusion of the Adolescent HIV Care Continuum in National HIV/ART Guidelines in 16 countries in sub‐Saharan Africa**

**Table:** 

Recommendation	Countries including recommendation in national HIV/ART guidelines
Frequency of HIV testing in sexually active adolescents	Lesotho, Kenya, South Africa, Tanzania, Rwanda, Swaziland, Uganda, Zambia
Recommended age of full disclosure	Lesotho, Cameroon, Ethiopia, Kenya, Namibia, Tanzania, Rwanda, Zambia
Age of consent for treatment initiation	Kenya, Rwanda, Swaziland
Frequency of routine viral load (VL) in adolescents	Malawi, Lesotho, Cameroon, Kenya, Namibia, Nigeria, South Africa, South Sudan, Tanzania, Rwanda, Zimbabwe, Swaziland, Botswana, Uganda, Zambia
Recommended age for transition from adolescent to adult services	Kenya, Namibia, Uganda, Zambia

## WEAE0101

### Multiple disease screening to destigmatize HIV testing and increase identification of persons living with HIV in Kisumu, Kenya

**K. Ndede^1^; D. Naitore^2^; F. Lugalia^1^; C. Wells^3^; B. Senyana^3^; J. Otieno^4^; R. Fayorsey^3^; M. Hawken^2^ and E. Abrams^3^**

^1^ICAP at Columbia University, Kisumu, Kenya, ^2^ICAP at Columbia University, Nairobi, Kenya, ^3^ICAP at Columbia University, New York, United States, ^4^Jaramogi Oginga Odinga Teaching and Referral Hospital, Kisumu, Kenya

**Background:** There are approximately 1.5 million people living with HIV in Kenya, and approximately 1 million are on ART. Attainment of the 90:90:90 goals depends on identifying the remaining HIV‐infected population, the majority of which are asymptomatic and do not access health services routinely. Adult HIV Testing Services (HTS) at Jaramogi Oginga Odinja Teaching and Referral Hospital (JOOTRH) in Kenya, have predominately been offered via universal opt‐out provider‐initiated testing and counseling (PITC) of out‐patients. This has resulted in testing a predominance of women and a yield of <1%. To de‐stigmatize HIV testing and reach more men, ICAP initiated a strategy of providing HTS as part of screening for multiple diseases.

**Description:** In March 2017, a multiple disease screening program was established at JOOTRH, offering a package of wellness services including screening for obesity (weight, height), hypertension (blood pressure), and symptomatic TB and HIV testing. The service was advertised by radio and billboard as a free health check. Located at the entrance to JOOTRH, clients and visitors (>15 years old) were eligible to access the service which was available from 8am to 9 pm daily including weekends.

**Lessons learned:** From July to December 2017, 5645 adults were screened. The mean age was 32.9 years (SD 11.7, range 15 to 78). Of those screened 100% were eligible for an HIV test (never tested or tested >3 months ago) and 2% (113/5645) tested HIV‐positive. Of those tested, 46% (n = 2604) were female and 54% (n = 3041) were male (*p* < 0.01), with a positivity yield of 2.2% (58/2604) in women and 1.8% (55/3041) in men. The extended hours were utilized, with 15.4% (n = 867) being tested after 5 pm on weekdays and 15.4% (n = 868) being tested on weekends. Amongst those tested on the weekends, 54% (465/868) were men and 46% (403/868) were women (*p* < 0.01).

**Conclusions/Next steps:** Providing HTS within a multiple disease screening package is an innovative approach that successfully tested a high number of men, a group that does not routinely access health services. Proportionally, more HIV‐positive individuals were identified with this strategy than out‐patient PITC. A higher testing yield may be possible if the project targets high‐risk populations and first time testers.

## WEAE0102

### Cracking the code to increase men's uptake of HIV testing: providing convenient and confidential outreach HIV testing services through mobile clinics

**E. Geoffroy^1^; N. Khozomba^2^; J. Jere^2^; E. Schell^3,4^; T. Schafer^3^; J. Goldman^5^ and K. Kabwere^6^**

^1^Global AIDS Interfaith Alliance (GAIA), Programs, Marblehead, United States, ^2^Global AIDS Interfaith Alliance, Blantyre, Malawi, ^3^Global AIDS Interfaith Alliance, San Rafael, United States, ^4^University of California, San Francisco, United States, ^5^Elizabeth Taylor AIDS Foundation, Beverly Hills, United States, ^6^Mulanje District Hospital, Mulanje District Health Office, Mulanje, Malawi

**Background:** In Malawi only 66% of men age 15 to 64 have been tested for HIV compared with 76% of women. With funding from the Elizabeth Taylor AIDS Foundation, in July 2014, Global AIDS Interfaith Alliance (GAIA) introduced a program to increase rates of male testing by reaching out to men with weekend HIV testing services (HTS) at convenient times and locations.

**Description:** Working in partnership with the District Health Offices, sites in need of HTS are identified. The mobile clinic is staffed by a driver and three trained health providers who travel to where men (and their partners) gather, including markets, tea estates, and churches/mosques, to provide HIV testing. Program data is collected using Magpi, a mobile app on a secured phone. Encrypted data is uploaded in real time using wi‐fi or cell connection.

**Lessons learned:** Through December 2017, there have been 106 male targeted testing events, testing 6166 people, of which 71% were male, compared to 26% males tested at weekday mobile clinics over the same period. Notably, 38% of males tested were aged 15 to 24 and of these, 42% had not previously been tested. Across all ages, 30% of males had not been previously tested. Three percent of men were found positive, with 5% of men over 25 testing positive, and all were referred to the nearest clinic providing antiretroviral therapy. The percentage of men attending these events has increased over time from 69% in 2014 to 78% in 2017. Convenience of location (38%), publicity surrounding the event (35%) and convenience of time (23%) were cited as primary reasons for attending.

**Conclusions/Next steps:** Men can effectively be reached for HIV testing in order to achieve the first UNAIDS “90” target by making testing convenient, providing a gender‐segregated space, and providing male counselors. Using a secure mobile data collection app allows for real time data analysis to improve program performance and target geographical areas where incidence is high and enables rapid in home follow up visits for those who consent. The next phase of the project will link treatment initiation and adherence data with testing data to assess time from diagnosis to treatment to adherence.

## WEAE0103

### Engaging young men in health care and HIV testing: the SHAZ! HUB youth drop‐in‐centre

**M.S. Dunbar^1^; G. Chapwanya^1^; G. Kumalo‐Sakutukwa^2^; I. Mahaka^1^; A. Mpakati^1^ and M. Lightfoot^2^**

^1^Pangaea Zimbabwe AIDS Trust, Harare, Zimbabwe, ^2^Center for AIDS Prevention Studies, UCSF, San Francisco, United States

**Background:** HIV testing efforts in sub‐Saharan Africa have focused on young women. However, studies show that young men face challenges in accessing preventative health care, including HIV testing, tending to be diagnosed late and presenting for HIV treatment with advanced disease. At the SHAZ! HUB, a youth drop‐in center and clinic in urban Zimbabwe, the majority of the clients are male. We explored factors contributing to this high uptake of services among young men.

**Methods:** We summarized information from clinical intake forms and conducted four focus group discussions, two with males aged 16 to 19 years and two with males aged 20 to 24, on topics related to health seeking behavior, facilitators and barriers to accessing services, and favorable and facilitative aspects of service delivery.

**Results: ** From July 2016, the SHAZ! HUB provided sexual health and HIV services to 2243 clients, 1332 (60%) of whom were male. Seventy percent were between the ages of 16 to 19 years, and the majority of clients sought HIV testing. The main barriers mentioned to seeking services generally were lack of confidentiality, fears of being embarrassed or made to feel uncomfortable, and the notion that clinics are “for women and babies”. When asked about favorable aspects of the SHAZ! HUB, participants cited factors focusing on environment, health care workers, and additional recreational services/benefits offered. For example, participants highlighted the privacy afforded by the HUB, and that services were “male friendly” – meaning the young men were not judged and given factual information and support on how to reduce risk that focused on the male perspective (e.g., no judgement when disclosing multiple sex partners). Participants also appreciated that they were allowed to be “loud,” and that they were not chastised for “misbehaving.” Other appealing aspects of the HUB included being able to access wi‐fi, relax with friends and watch satellite TV, and access workshops on life skills and financial literacy.

**Conclusions: **These lessons learned suggest that programs seeking to engage young men could consider combining health services with recreational opportunities, non‐judgmental information and support within a private, youth friendly environment. Such factors create openings for young men to engage in prevention and care.

## WEAE0104

### Home‐based testing identifies more previously undiagnosed older men than mobile testing in Botswana

**M. Roland^1^; L. Block^2^; P. Bachanas^2^; M.G. Alwano^1^; W. Abrams^1^; K. Wirth^3^; T. Gaolathe^4^; J. Makhema^4^; M. Mmalane^4^; S. Lockman^3^; S. El‐Halabi^5^ and J. Moore^2^**

^1^Centers for Disease Control & Prevention, Gaborone, Botswana, ^2^Centers for Disease Control & Prevention, Division of Global HIV/AIDS & TB, Atlanta, United States, ^3^Harvard T.H. Chan School of Public Health, Boston, United States, ^4^Botswana Harvard Partnership, Gaborone, Botswana, ^5^World Health Organization, Geneva, Switzerland

**Background:** UNAIDS estimates that 85% of HIV‐positive individuals in Botswana know their status. As countries approach UNAIDS 95‐95‐95 targets, it is important to employ the most effective HIV testing strategies to reach the remaining undiagnosed people of all ages and sexes.

**Methods:** The Botswana Combination Prevention Project (BCPP) is a randomized controlled trial in 30 matched rural or semi‐urban communities. We describe newly identified HIV‐positive community residents by age, sex and testing strategy in the 15 intervention communities where mobile and home testing strategies were employed between October 2013 and September 2017. Chi‐square tests account for possible community‐level intra‐cluster correlations.

**Results: ** 49,693 participants did not know or have documentation of their negative HIV status in the prior three months and were tested for HIV with home (19,349; 39%) or mobile (30,344; 61%) testing. Among these, 1870 (3.8%) were newly diagnosed. Similar absolute numbers of women and men, 956 and 916 respectively, were diagnosed (Table 1). The proportion of new diagnoses was higher in the oldest age categories as compared to the 16 to 24 year olds (*p* < 0.0001). Older males yielded the highest proportion of new diagnoses among all categories (*p* < 0.0001). When examining testing by venue, home testing represented 39% of the total tests conducted and yielded 46% of the new positives identified. The testing yield for new HIV diagnoses in the home (4.5%) was significantly higher than the 3.3% yield with mobile testing (*p* < 0.0001). Similar absolute numbers of males and females were newly diagnosed with the 2 testing modalities. However, home testing yielded more new diagnoses among older persons compared to mobile (*p* < 0.0001).

**Conclusions: **In this rural and semi‐urban population, home testing yields were greater than mobile testing yields particularly among older males, suggesting that this strategy remains important for identifying HIV‐infected individuals in a country with high HIV identification coverage. While a similar absolute number of males and females overall were identified with each strategy, absolute numbers and proportionate yield varied across age‐sex categories and testing approach. Similar granular data may be used to select testing strategies specific to the first 95 gap across age‐sex categories within local geographic areas.

**Abstract WEAE0104‐Table 1. Newly Diagnosed Participants Identified in 15 Rural and Semi‐Urban Botswana Communities, By Age, Sex, and Testing Venue**

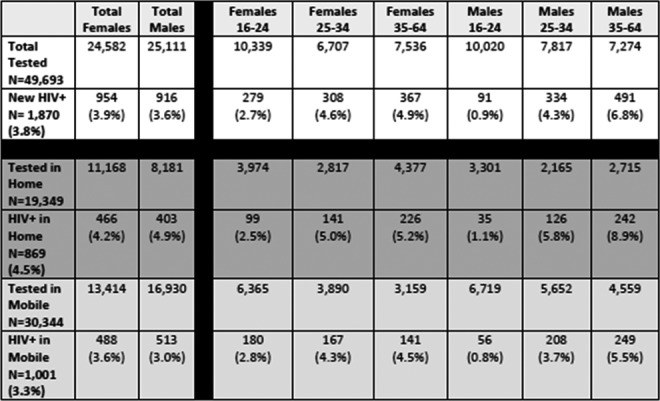

## WEAE0105

### Male engagement works to improve HIV services uptake among men

**T.A. Lekhotsa^1^; B. Oguntoyinbo^2^; M. Mputsoe^3^; M.G. Mefane^4^; V. Tukei^5^ and E. Tumbare^1^**

^1^Elizabeth Glaser Pediatric AIDS Foundation, Programs, Maseru, Lesotho, ^2^Elizabeth Galser Peddiatric AIDS Foundation, Research, Maseru, Lesotho, ^3^Elizabeth Glaser Pediatric AIDS Foundation, Strategic Information and Evaluation, Maseru, Lesotho, ^4^Ministry of Health, Sexual and Reproductive Health Ministry of Health Lesotho, Maseru, Lesotho, ^5^Elizabeth Galser Pediatric AIDS Foundation, Research, Maseru, Lesotho

**Background:** HIV testing services (HTS) are a critical entry point to HIV care and treatment. However, in Lesotho uptake of HTS is lower among men than women (36% of men were tested compared to 58% of women: DATIM, October 2015 to September 2016) because cultural and social barriers can prevent or delay men from getting an HIV test. Elizabeth Glaser Pediatric AIDS Foundation (EGPAF) established Men's Clinics in June 2017 for the provision of comprehensive health services for men in health facilities as part of the DREAMS initiative to increase uptake of HIV services among men.

**Methods:** Routine program data were analyzed to examine changes in HIV testing and antiretroviral treatment (ART) uptake among men before (January to March 2017) compared to after (July to September 2017) implementation of the men's health services in seven selected health facilities (two hospitals and five clinics) in Lesotho. Data on HIV testing and ART initiation were abstracted from routine aggregate program data and downloaded into Stata for review and analysis. The Men's Clinics intervention consisted of dedicated clinic space for male clients only to receive services by male nurses and counsellors to help men feel more comfortable accessing services. Comprehensive clinical services (STI Screening and treatment, HTS, care and treatment, Pre‐P and PEP, TB Services, treatment for other co‐infections, Condom distribution, and counselling for HIV prevention, Education on PMTCT and Index family testing, and Referral Male Medical Circumcision) were offered at unconventional hours of service (morning, evening, and weekend hours), and through innovative appointment scheduling to reduce waiting times and improve client satisfaction.

**Results: ** The results indicate that since Men's Clinics were introduced in June 2017 there was a 49% increase in the number of men tested for HIV; a 29% increase in the number of men diagnosed as HIV‐positive; and a 63% increase in the number of men initiated on ART by the end of September 2017.

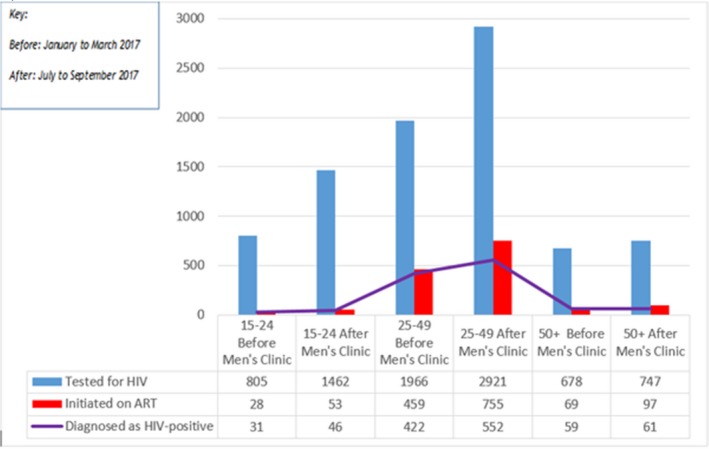

**Abstract WEAE0105‐Figure 1. Number of men tested for HIV, diagnosed as HIV‐positive, and initiated on ART before and after introduction of the Men's Clinic, by age.**

**Conclusions: **Providing male‐only comprehensive clinical services is a promising strategy for increasing men's access to HIV testing and linkages to care. Future scale‐up of Men's Clinics could help in identifying large numbers of men who are living with HIV.

## WEAE0201

### Impact of the PEPFAR geographic pivot on HIV & non‐HIV health services in Uganda

**J. Wilhelm^1^; L. Paina^1^; M. Qiu^1^; M. Makuru^2^; F. Ssengooba^2^ and S. Bennett^1^**

^1^Johns Hopkins Bloomberg School of Public Health, Department of International Health, Baltimore, United States, ^2^Makerere University School of Public Health, Health Policy & Management, Kampala, Uganda

**Background:** In Uganda, PEPFAR implemented a geographic prioritization (GP), which discontinued support for outreach, training, supervision, and staff incentives to roughly 730 health facilities, shifting them to government central support (CS). CS facilities are located in low HIV‐prevalence districts or did not provide high‐volume HIV care.

**Methods:** In order to assess the impacts of the GP, we fielded a survey at 262 health facilities across Uganda in mid‐2017. The survey collected information on PEPFAR support, service delivery, commodities, laboratory, time‐allocation, motivation, and human resources. We also obtained DHIS2 records on select HIV and non‐HIV services (2013 to 2017) and HRHIS staffing data (2015 to 2017). We conducted difference‐in‐difference analysis comparing CS facilities to those maintained on PEPFAR before and after GP using random intercept regression models.

**Results: ** Relative to maintained facilities, CS facilities are significantly more likely to report termination of workers, declining frequency of supervision, and worsening financial status. CS facilities are also significantly more likely to report discontinuing HIV outreach (52% vs. 4%, *p* < 0.001), worsening quality of HIV care (42% vs. 0%, *p* < 0.001), less improvement in the quality of maternal neonatal and child health care (34% vs. 70%, *p* < 0.001), and increased disruption of viral load (23% vs. 5%, *p* = 0.002) and sputum (22% vs. 6%, *p* = 0.026) testing. More workers at CS facilities report less time on HIV care (32% vs. 11%, *p* = 0.004) and declines in satisfaction (27% vs. 1%, *p* < 0.001) since transition.

However, the HRHIS data shows that adjusted difference‐in‐difference in staffing ratios for CS facilities relative to maintained facilities was small and not statistically significant. According to DHIS2 data, there were no significant differential trends in volume of HIV tests, current on ART, OPD visits, ANC visits, Facility‐based deliveries, or DPT/Pentavalent‐third dose.

**Conclusions: **Early results suggest that the PEPFAR GP in Uganda has had limited immediate impacts on service delivery and staffing levels at CS facilities, despite the impacts reported in our survey. However, discontinuation of outreach, reduced access to supervision, weakened links to lab hubs, and declining time‐allocation for HIV care may ultimately hinder the ability to identify and retain HIV patients and jeopardize Uganda's progress towards the 90‐90‐90 goals.

## WEAE0202

### From 90‐90‐90 towards HIV elimination with boosted‐integrated active HIV case management (B‐IACM) in Cambodia

**P.S. Ly^1^; V. Ouk^1^; C. Kaoeun^2^; S. Samreth^3^; B. Ngauv^3^; S. Seng^4^; D. Nhim^5^ and S. Wignall^5^**

^1^NCHADS, NCHADS, Phnom Penh, Cambodia, ^2^NCHADS, AIDS Care Unit, Phnom Penh, Cambodia, ^3^NCHADS, Technical Bureau, Phnom Penh, Cambodia, ^4^FHI 360, LINKAGES Project, Phnom Penh, Cambodia, ^5^FHI 360, LINKAGES, Phnom Penh, Cambodia

**Background:** The National HIV Program in Cambodia has been successful in reducing HIV prevalence in the general adult population from 1.7% in 1998 to 0.6% in 2016 with strong prevention programs, expansion of HIV testing, and optimization of the continuum of care with ART coverage reaching more than 80% PLHIV. In 2016 AEM modeling, 70,498 individuals were estimated to be HIV infected; 58,338 (83%) knew their status; and, 56,754 (97%) of these on ART1 but 12,000 PLHIV do not yet know their HIV status and new approaches are needed to find them. Cambodia's HIV program, having achieved UNAIDS 90‐90‐90 targets, is focused on elimination of HIV transmission by 2025 and B‐IACM is central to that plan.

1 Spectrum/AEM modelling 2016.

**Description:** The BIACM strategy, implemented at the Operational District (OD) level, involves an HIV Case Management Coordinator (CMC) assisted by a Case Management Assistant (CMA) work daily with HIV Case Management Service Providers (CMPs) who coordinate, communicate, and share information concerning HIV cases. Their efforts ensure timely enrollment in care and initiation of ART and support adherence and achievement of viral load suppression. Pregnant women, HIV‐exposed infants, and PLHIV partners are targeted for testing, enrollment in HIV care and treatment if HIV+. Coordination meetings between CMC and CMPs and broader stakeholders review site performance and identify solutions to improve care and retention. Direct data reporting to the National Center for HIV/AIDS, Dermatology and Sexually Transmitted Infections (NCHADS) is used to generate a dashboard that allows offsite monitoring and intervention as necessary.

**Lessons learned:** B‐IACM has doubled previous yield, increasing new HIV case detection from 444 (pre‐B‐IACM from February 2014 to September 2015) to 753 (B‐IACM from October 2015 to May 2017) in Battambang and Siem Reap. The B‐IACM strategy has: improved targeting for HIV testing and the achievement of the first 90% (2); improved ART initiation rates to 96% (1,577/1,647), the second 90; and, increased adherence achieving viral load suppression to undetectable levels in 97% of cases, the third 90.

(2) http://www.unaids.org/en/resources/presscentre/pressreleaseandstatementarchive/2017/July/20170720_PR_Global_AIDS_Update_2017

**Conclusions/Next steps:** NCHADS will scale up B‐IACM implementation to all provinces in Cambodia as it moves toward achieving 95‐95‐95 by 2025.

## WEAE0203

### Reaching the first “90”: Decentralizing and strengthening provider initiated testing services at primary health care facilities in Ukraine

**M. Makovetska; A. Vasylkova; N. Davydenko; O. Yaremenko and N. Avaliani**

Deloitte Consulting, LLP, USAID HIV Reform in Action Project, Kiev, Ukraine

**Background:** As of October 2017, an estimated 98,600 people living with HIV (PLWH) in Ukraine are not aware of their diagnosis. To achieve the first “90” of the UNAIDS targets, it is necessary to diagnose 74,806 PLWH. However, the current HIV detection system in Ukraine has several gaps and limitations, including a limited number of “entry points” into the cascade of HIV prevention and care. The USAID HIV Reform in Action project sought to decentralize HCT services to increase HIV service entry points and accelerate progress towards the first “90” target.

**Description:** Decentralizing HTC services from regional level AIDS centers and local hospitals to the expanded network of primary health care centers (PHC) sites was implemented in 14 cities across seven regions of Ukraine between March 2016 and December 2017. The decentralization strategies included allocation of funds for rapid HIV tests from local budgets; training for PHC personnel in HIV testing services; and removing local procedural barriers for the provision and scale‐up of HCT services through the introduction of policies to use two rapid tests for HIV diagnosis.

**Lessons learned:** By the end of the pilot, all sites allocated funds for procurement of rapid test kits resulting in a 6‐time increase in rapid test supply compared to the previous year. The number of PHCs providing HCT increased from 25 PHCs across 14 cities in 2015 to 225 PHCs in 2017. The comprehensive strategy, including workforce training, funding allocations and decentralization of patient's entry points, was critical to achieving successful outcomes.

**Conclusions/Next steps:** Increasing the number of HTC service delivery and patient entry points to the health care system will expand testing services beyond traditional entry points and help detect HIV patients coming to HCF for regular primary health care services. Plans are underway to disseminate pilot results to all seven study regions, including districts and cities, to share lessons learned. To sustain the outcomes, local authorities need to invest in training of human resources, infrastructure, and other health‐system‐strengthening components, as well as increase local budget allocations for HIV services.

## WEAE0204

### Monitoring viral load for the last mile: what will it cost?

**B.E. Nichols^1,2^; S.J. Girdwood^2^; T. Crompton^3^; L. Stewart‐Isherwood^4,5^; L. Berrie^4,5^; D. Chimhamhiwa^3^; C. Moyo^6^; J. Kuenhle^7^; S. Rosen^1,2^ and on behalf of EQUIP**

^1^Boston University, Global Health, Boston, United States, ^2^Health Economics and Epidemiology Research Office, Department of Internal Medicine, School of Clinical Medicine, Faculty of Health Sciences, University of the Witwatersrand, Johannesburg, South Africa, ^3^Right to Care, GIS Mapping Department, Johannesburg, South Africa, ^4^National Health Laboratory Service, Johannesburg, South Africa, ^5^Wits Health Consortium, Johannesburg, South Africa, ^6^EQUIP Zambia, Lusaka, Zambia, ^7^United States Agency for International Development, Lusaka, Zambia

**Background:** Routine viral load testing is the WHO‐recommended method for monitoring HIV‐infected patients on ART, and many countries are rapidly scaling up testing capacity. Providing testing access to the most remote populations (the “last mile”) is especially challenging. Using a geospatial optimization model, we estimated the incremental costs of reaching the hardest‐to‐reach 20% of patients in Zambia by expanding the transportation network required to bring blood samples from ART clinics to centralized laboratories and return results to clinics.

**Methods:** The model first optimized a sample transportation network (STN) that can transport 80% of anticipated sample volumes to centralized viral load testing labs on a daily or weekly basis, in line with the Zambia 2020 viral load targets. Data incorporated into the model included the location and infrastructure of all 2500 Zambian health facilities and laboratories, measured distances and drive times, expected future viral load demand by health facility, and local cost estimates. We then continued to expand the modeled STN in 5% increments until 100% of sample volumes were met.

**Results: ** The cost per viral load test when reaching 80% patient volumes using centralized viral load testing was a median of $23.43 (IQR $21.92‐$25.42). With an expanded STN, the incremental cost per test rose to $24.72 ($23.08‐$26.90) for 80% to 85% and $24.82 ($23.28‐$26.94) for 85% to 90%. Above 90% coverage, the incremental cost per test increased substantially to $35.17 ($33.17‐$38.35) for 90% to 95% and $57.23 ($52.67‐$63.16) for 95% to 100%. The high numbers of kilometers driven per sample transported increases the costs dramatically for reaching the clinics that serve the last 5% of patients.

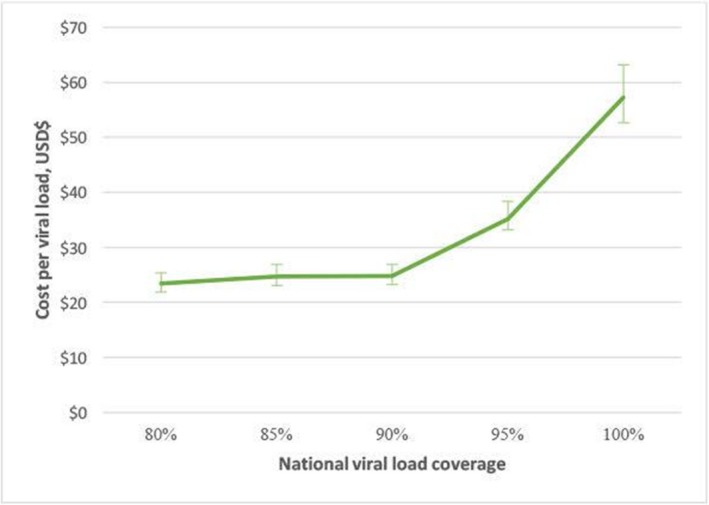

**Abstract WEAE0204‐Figure 1. Median incremental cost per viral load (and interquartile range) in Zambia when increasing coverage in 5% increments ($US).**

**Conclusions: **Providing sample transport services to the most remote clinics in low‐ and middle‐income countries is likely to be cost‐prohibitive. Other strategies are needed to reduce the cost and increase the feasibility of making viral load monitoring available to the last 10% of patients. The cost of alternative methods for reaching the last patients, such as optimal point‐of‐care viral load equipment placement and usage, dried blood spot specimen utilization, or use of drones in geographically remote facilities, should be evaluated.

## WEAE0205

### The impact of PEPFAR PMTCT funding on reduced infant mortality and improved ANC care in Kenya: a quasi‐experimental evaluation

**D.A. Barnhart^1^; I. Tsikhutsu^2,3^; F. Sawe^2,3^; J. Muli^2,3^; D. Kirui^2,3^; W. Sugut^2,3^; N. Abboud^4^; T. Hamm^5,6^; P. Coakley^5,6^; P.W. Hickey^5^; V. Wolfman^5,6^; E. Lee^5,6^ and D. Spiegelman^7^**

^1^Harvard T.H. Chan School of Public Health, Department of Epidemiology, Boston, United States, ^2^Walter Reed Program‐Kenya, Kericho, Kenya, ^3^U.S. Military HIV Research Program, Silver Spring, United States, ^4^Office of the U.S. Global AIDS Coordinator and Health Diplomacy, Washington, United States, ^5^U.S. Military Research Program, Walter Reed Army Institute of Research, Silver Spring, United States, ^6^Henry M. Jackson Foundation for the Advancement of Military Medicine, Bethesda, United States, ^7^Harvard T.H. Chan School of Public Health, Departments of Epidemiology, Biostatistics, Nutrition, and Global Health, Boston, United States

**Background:** From 2004 to 2014, the President's Emergency Plan for AIDS Relief (PEPFAR) invested over $240 million in Prevention of Mother to Child Transmission of HIV (PMTCT) in Kenya. During this same time, child mortality in Kenya decreased by half. The extent to which this decrease is attributable to PEPFAR is unknown.

**Methods:** We mapped annual PEPFAR funding for PMTCT to Kenyan provinces using 2004 to 2014 Country Operational Reports and linked funding to Demographic and Health (DHS) and AIDS Indicator Surveys (AIS). We used a quasi‐experimental dose‐response analysis to evaluate the impact of annual (ANN‐PC) and cumulative (CUM‐PC) per capita PEPFAR funding for PMTCT on infant mortality, neonatal mortality, and HIV counseling, testing, and receipt of test results during antenatal care (HIV testing at ANC). Risk ratios were estimated using generalized estimating equations, and regression models were adjusted for year, province, and respondent characteristics.

**Results: ** Our secondary analysis included 30,424 infants and 21,048 mothers. We found that a $0.33 increase in ANN‐PCF, or the difference between the 75th and 25th (IQR) percentiles of annual funding levels, was significantly associated with an 11% (95% CI: 1% to 21%) reduction in infant mortality after a 1‐year lag. This reduction was sustained after a 2‐year lag. An $0.83 increase in CUM‐PCF, or the IQR of cumulative funding levels, was significantly associated with a 31% decrease in infant mortality (95% CI: 11% to 46%), with the estimated associations attenuating with successive lags. A $0.33 increase in ANN‐PCF was also associated with a 6% increase in HIV testing at ANC after a 3‐year lag (95% CI: 2% to 10%), with similar findings for CUM‐PCF. As expected, funding was not associated with neonatal mortality.

**Conclusions: **We found evidence that PEPFAR funding for PMTCT may be causally associated with reduced infant mortality and increased HIV testing at ANC in Kenya. The full impact of PMTCT funding may not be felt for several years after it is allocated. Our methods, paired with routine, publicly available data sets like DHS and AIS, can be extended to other countries and health challenges to demonstrate the impact of large‐scale donor programs like PEPFAR and to inform resource allocation by policymakers.

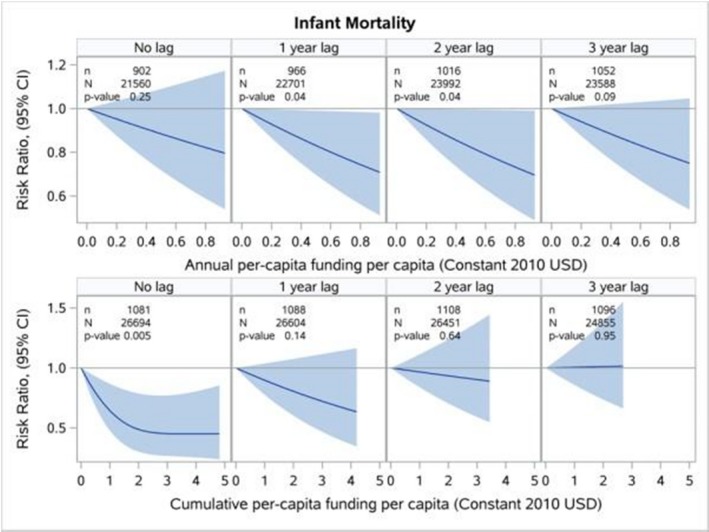

**Abstract WEAE0205‐Figure 1. Infant mortality and per capita PEPFAR funding for PMTCT in Kenya.**

## WEAE0301

### Global optimization of the response to HIV

**J. Stover and Y. Teng**

Avenir Health, Center for Modeling and Analysis, Glastonbury, United States

**Background:** Over $20 billion is spent annually on HIV programs in low‐ and middle‐income countries. The allocation of resources by country and intervention depends on a mixture of need, funding sources, capacity, effectiveness, policy, politics and social factors. We investigated how well the current allocation is optimized for cost‐effectiveness.

**Methods:** We applied an HIV simulation model, Goals, to 55 countries accounting for about 90% of all new infections to determine the cost per infection, death and DALY averted for each of 13 interventions. Units costs were based on Investment Cases for each country. The models were fit to surveillance and survey data and UNAIDS official estimates. Cost‐effectiveness ratios were calculated by country and intervention by scaling up each intervention, one‐at‐a‐time, over five years and recording the incremental costs, infections, deaths and DALYs compared to a counterfactual of no scale up of any interventions. The results comprise a database of total cost and cost‐effectiveness measures for 716 country/intervention pairs.

**Results: ** Cost‐effectiveness varies widely across countries and interventions as shown in Figure 1 for cost per infection averted. Six percent of these combinations are cost savings because the total cost of the intervention is less than the savings generated due to treatment costs averted. ART dominates cost per death and DALY averted and also ranks high in cost‐effectiveness for infections averted. The most cost‐effective prevention interventions are generally VMMC, PMTCT, outreach to sex workers and condom promotion. These programs currently receive about 14% of direct intervention funding, which is about two‐thirds of the need. The most cost‐effective programs are in East and Southern Africa where incidence is high and costs are generally low. Currently almost 60% of all resources and 70% of donor resources are focused on ESA. About 15% of donor resources go to West and Central Africa where cost‐effectiveness can be considerably worse.

**Conclusions: **Resources for HIV prevention and treatment are generally targeted appropriately but more focused allocation of resources could improve cost‐effectiveness by about a quarter. Resource allocations should be continually assessed because cost‐effectiveness can change significantly as incidence patterns change.

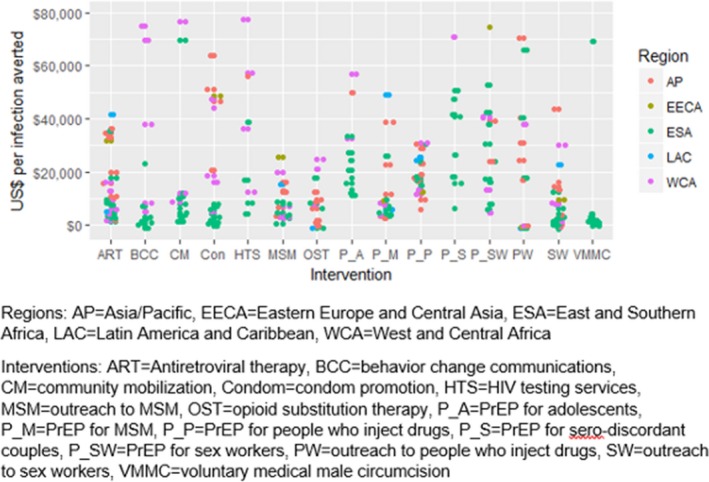

**Abstract WEAE0301‐Figure 1.**

## WEAE0302

### How does domestic HIV/AIDS financing respond to declines in development assistance for HIV/AIDS?

**A. Haakenstad^1^; M. Moses^2^; T. Tao^2^; A. Chapin^2^ and J. Dieleman^2^**

^1^Harvard T.H. Chan School of Public Health, Boston, United States, ^2^Institute for Health Metrics and Evaluation, University of Washington, Seattle, United States

**Background:** Development assistance for HIV/AIDS aims to complement and enhance domestic spending. However, prior analyses have shown that development assistance displaces government financing: as aid grows, increases in government spending are not as large as they would be in the absence of aid. Furthermore, when aid retracts, government funds do not necessarily fill the gap in financing. While this dynamic has been studied for the health sector overall, we know little about whether it extends to the HIV/AIDS sector. We analyze this relationship using newly available HIV/AIDS financing data.

**Methods:** Our data consist of estimates of HIV/AIDS financing by source and treatment generated by IHME. The number of people living with HIV/AIDS and ten‐year lag‐distributed income data are sourced from the Global Burden of Disease Study 2016. General government expenditure estimates are drawn from the World Bank's World Development Indicators. We use fixed effects and system generalized methods of moments regression to analyze the relationship between external and government financing for HIV/AIDS overall, broken down by treatment, and with an interaction capturing the distinct effect of declines of aid.

**Results: ** Unlike the broader health sector, the development assistance disbursed to fight HIV/AIDS is associated with increases in the rate of change of government spending on HIV/AIDS – for each additional dollar of development assistance for HIV/AIDS, an estimated 9 (95% UI: 2 to 16) additional cents are spent by governments on HIV/AIDS. This effect is even stronger for spending on HIV/AIDS treatment. However, when external assistance declines, so does domestic financing. And declines in domestic expenditure transpire at approximately the same rate – or even faster – than increases.

**Conclusions: **Our analysis suggests that the declines in HIV/AIDS aid over the past five years are associated with decreases in the domestic financing available for HIV/AIDS. Domestic sources of HIV/AIDS funding are not filling the financing gaps created. If the decreases in development assistance continue, and are accompanied by sustained declines in government financing, treatment and prevention efforts and the major reductions in incidence and mortality achieved over nearly two decades may be at risk.

## WEAE0303

### Forecasting the cost of financing ART in sub‐Saharan Africa under differential funding scenarios

**T. Frank and A. Carter**

Institute for Health Metrics and Evaluation, Seattle, United States

**Background:** Amid stagnated international funding for HIV and longer lifespans for people living with HIV (PLHIV), quantifying future antiretroviral therapy (ART) needs is vital for HIV program design and financing. With the present study we summarize the expected cost of financing ART for each country through 2040 by predicting the price of ART, projecting funding under different scenarios, and modeling the HIV epidemic for each scenario.

**Methods:** We estimated ART prices using a frontier analysis based on Global Price Reporting Mechanism data. We projected current levels of government health expenditure and development assistance for health for HIV treatment based on 15^th^ (pessimistic), 50^th^ (reference), and 85^th^ (optimistic) percentile rates of change across 46 countries, which then informed ART coverage projections by translating predicted funding into expected treatment using our cost projections. We used each scenario as inputs into Spectrum, a cohort component model that applies disease progression parameters to an age‐ and sex‐specific population over time. Spectrum results provided a full time series of HIV prevalence by country through 2040.

**Results: ** Estimated median annual ART price per patient was projected to decrease from $82.51 USD in 2016 to $26.74 in 2040. Country‐specific ART cost varied considerably, from $21.03 to $41.64, with the highest prices predominantly in western sub‐Saharan Africa (SSA). In the reference scenario, the number of PLHIV in SSA increased from 14.3 million in 2016 to 40.5 million in 2040, a trend primarily fueled by population growth and extended survival of PLHIV. Conversely, incidence was projected to decrease from 1.4 per 1000 population in 2016 to 0.75 per 1000 in 2040. Compared with the reference scenario, by 2040, the optimistic scenario estimated 29.4 million PLHIV in SSA whereas the pessimistic scenario projected 81.5 million. Cumulatively, the estimated cost of purchasing ART for SSA exceeded $30 billion for the 2016 to 2040 reference scenario.

**Conclusions: **The large disparity in the number of PLHIV in 2040 between the optimistic and pessimistic scenarios underscores the vital impact of HIV funding. Despite declines in projected HIV incidence, demographic factors and greater survival stresses the importance of sustained – if not heightened – HIV funding in the future.

**Abstract WEAE0303‐Table 1. Forecasted ART Price and number of PLHIV by Sex of Highest Burden Countries in 2040 (Reference Scenario)**

**Table:** 

Location	Male PLHIV (thousands)	Female PLHIV (thousands)	Price of ART (USD)
South Africa	3602.1	5326.4	21
Nigeria	2342.4	3437.3	28.6
Tanzania	1358.6	1886.8	26.6
Uganda	1291	1858.8	27.3
Kenya	1170.8	1743.1	23.8
Mozambique	1130.8	1731.3	29.3
Zimbabwe	1039.7	1456.3	22.4
Malawi	953.4	1411.9	23.7
Zambia	928.2	1294.7	27.8

## WEAE0304

### Achieving sustainable Workplace HIV/AIDS Programmes through a phased out catalytic financing model: case of Swedish Workplace HIV/AIDS Programme in sub‐Saharan Africa

**D.M. Mwaura**

International Council of Swedish Industries (NIR), Swedish Workplace HIV/AIDS Programme, Nairobi, Kenya

**Background:** The Swedish Workplace HIV and AIDS Programme (SWHAP) is a joint initiative by the International Council of Swedish Industry (NIR) and the Swedish Industrial and Metalworkers’ Union (IF Metall). It is a strategy to contribute to the establishment and/or support of HIV workplaces programmes in sub‐Saharan Africa; an example of how management and employees can contribute to a successful intervention that saves lives and secures future markets. The programme helps companies invest in workplace programmes that reverse the negative impact of HIV and AIDS making the programmes sustainable and best practice. It is a case of how transitional funds can establish sustainable programmes and catalyse further investments from private sector into HIV/AIDS and wellness programmes.

**Description:** The funding model entails a three phase formula where after the start‐up engagement, the activity budgets are funded 60% during the first phase, 50% in the second phase and 40% during the third and final phase of the co‐funding. This scaled‐down approach ensures commitment to continuation of the programme through mainstreaming into the company sustainability plan. The funding is also on reimbursable claims based on actual implementation and expenditure thus a result based financing.

**Lessons learned:** During 2016, 361 workplaces in 116 companies benefited from the transitional financing. A total of 29,705 employees got information and awareness with HIV testing at 52% average. 9793 family members were educated on HIV/AIDS and tested. 8704 key populations reached while 367,830 condoms distributed. On transition, 99% of the companies funded have sustained the programmes becoming best practice examples in contribution to the National frameworks of HIV/AIDS responses and winning various awards; Examples of workplaces that uphold rights of infected employees and non‐discrimination practices. The steering committees in the various workplaces have promoted social dialogue even in addressing matters pertaining to management/workers relationship leading to harmonious working environment.

**Conclusions/Next steps:** This transitional financing model exemplifies how to start sustainable programmes past the partnership, speaks to the business benefits, leading to increased funding from the companies and into sustainability plans.

Next is to share the model widely with development partners in Public‐Private Partnerships. The model infrastructure is applicable for other programmes for sustainability.

## WEAE0305

### Optimizing resource allocation for HIV prevention programs: proof of concept of an analytical framework using data from Mexico

**A. Salas‐Ortiz^1^; G. La Hera‐Fuentes^1^ and S.A. Bautista‐Arredondo^1,2^**

^1^National Institute of Public Health, Centre for Health Systems Research, Cuernavaca, Mexico, ^2^UC Berkeley, School of Public Health, Berkley, United States

**Background:** Annually, the National Centre for the Prevention and Control of HIV/AIDS of Mexico (CENSIDA) invites Civil Society Organizations (CSOs) to apply for funding to implement HIV prevention projects. Given Mexico's concentrated epidemic in key populations, CSOs play a fundamental role in delivering prevention services to these communities. Even though this program has been implemented for a long time, there is no evidence regarding its performance. The present analysis shows how decision‐makers can use monitoring data to improve the allocation of domestic resources to HIV prevention interventions.

**Methods:** The conceptual framework proposed by Bautista‐Arredondo et al., (2008) was used to evaluate the program's efficiency. The approach assesses the level of efficiency considering three dimensions: cost‐effectiveness, targeting and technical efficiency. The approach consists of comparing an observed indicator to a normative one (benchmark). Data from 142 funded projects in 2016 were analysed from the CENSIDA's monitoring tool. Data abstracted from the records included: input costs by category, number of outputs by key populations and interventions delivered by each project. These facets were identified through content analysis and used to calculate unit costs. Benchmarks for the cost‐effectiveness and targeting dimensions were constructed based on prior literature and HIV prevalence data; minimum unit costs were considered the benchmark for the technical efficiency dimension. Observed indicators were compared with each respective benchmark.

**Results: ** Current literature suggests that interventions to diagnose HIV are the most cost‐effective in contexts of concentrated epidemics; yet, only 24% of interventions implemented in the sample focused on testing. Regarding targeting, men who have sex with men and male sex workers received less funds proportional to their HIV prevalence and size. There was considerable heterogeneity in unit costs within interventions, suggesting significant inefficiencies in service delivery.

**Conclusions: **These findings allowed stakeholders to re‐design the 2017 call to incentivize CSOs to: propose a higher number of the required interventions, intervene in difficult‐to‐reach key populations, and carry out cost‐effective prevention services. This study highlights how monitoring data has the potential to better inform the decision‐making process. Moreover, it is an example for middle‐income countries in which a financial transition from international donors to domestic funding is occurring.

## WEAE0401

### Factors influencing initiation, continuation & discontinuation of oral PrEP at selected facilities in South Africa

**D. Pillay^1^; S. Jenkins^2^; M. Murire^1^; K. Stankevitz^3^; H. Subedar^4^ and S. Mullick^1^**

^1^Wits Reproductive Health and HIV Institute, Johannesburg, South Africa, ^2^Clinton Health Access Initiative, Pretoria, South Africa, ^3^FHI 360, Durham, United States, ^4^South Africa National Department of Health, Pretoria, South Africa

**Background:** Sex workers (SW) and men who have sex with men (MSM) in South Africa are at substantial risk of HIV. Hence oral pre‐exposure prophylaxis (PrEP) was launched for SW in 2016 and MSM in 2017. Programmatic data shows variability in initiation and continuation between these populations. This study examines factors related to PrEP initiation, continuation, and discontinuation during the national PrEP rollout.

**Methods:** A cross‐sectional survey was administered September 2017 to January 2018 among clients (ages 18 to 62) and providers at 9 facilities implementing oral PrEP in South Africa. The client survey captured PrEP initiation, continuation and discontinuation. The provider survey captured knowledge, attitudes and practiced behaviors towards PrEP. Descriptive analyses were performed on survey data. Continuation and discontinuation questions allowed for multiple responses.

**Results: ** 288 clients (152 SW, 68 MSM, 68 other) and 30 providers (3 clinicians, 13 nurses, 6 counselors, 8 peer‐educators) participated. Of 152 SW, 57 (37.5%) self‐identified as current PrEP users and 46 (30.2%) as past users. Of 68 MSM, 25 (36.8%) self‐identified as current PrEP users and 34 (50%) as past users. Primary reasons current and past users initiated PrEP included: being sexually active (SW 33%; MSM 18.6%), having multiple sexual partners (SW 26.2%; MSM 8.5%), and perceiving HIV risk (SW 22.3%; MSM 8.5%). Reasons current users continued PrEP were similar to initiation reasons: being sexually active (SW 50.9%; MSM 76.0%), having multiple sexual partners (SW 35.1%; MSM 24%), and perceiving HIV risk (SW 40.4%; MSM 40%). The primary reason for past users discontinuing PrEP was that side effects were too great (SW 71.7%; MSM 79.4%). The majority of providers (n = 29; 96.7%) said that participants experienced minimal side effects on PrEP and only 8 (26.7%) identified side effects as a barrier.

**Conclusions: **SW and MSM in South Africa identify their sexual behavior and perception of HIV risk as reasons to initiate oral PrEP. However, side effects appear to be a challenge among users for oral PrEP continuation, and appear underestimated by providers. This highlights the need to better sensitize providers on user perceptions about side effects in order to inform their counseling messages and side effect management.

## WEAE0402

### PrEP uptake among pregnant and postpartum women: results from a large implementation program within routine maternal child health (MCH) clinics in Kenya

**J. Kinuthia^1,2^; J. Pintye^2^; F. Abuna^3^; H. Lagat^3^; K. Mugwanya^2^; J. Dettinger^2^; M. Serede^3^; J. Sila^3^; J.M. Baeten^4^; G. John‐Stewart^4^; PrEP Implementation for Young Women and Adolescents (PrIYA) Program**

^1^Kenyatta National Hospital, Nairobi, Kenya, ^2^University of Washington, Department of Global Health, Seattle, United States, ^3^University of Washington‐Kenya, Nairobi, Kenya, ^4^University of Washington, Departments of Global Health, Epidemiology and Medicine, Seattle, United States

**Background:** Very few examples of PrEP delivery to pregnant and postpartum women have been reported. The PrEP Implementation for Young Women and Adolescents (PrIYA) Program provides real‐world evidence on delivering PrEP to pregnant and postpartum women in Kenya.

**Methods:** PrIYA is part of the DREAMS Innovation Challenge funded by PEPFAR managed by JSI Research & Training Institute, Inc. We approached HIV‐uninfected pregnant women seeking routine antenatal (ANC) and postnatal (PNC) services at 16 maternal and child health clinics in Kisumu, Kenya from June to December 2017. At each patient encounter, screening for behavioral risk factors and willingness to consider PrEP was conducted per national PrEP guidelines. Those who were willing to consider PrEP were assessed for medical eligibility and those eligible were offered PrEP at the same visit. Logistic regression models determined correlates of PrEP initiation.

**Results: ** In total, we conducted 9704 assessments among pregnant/postpartum clients for behavioral risk factors and willingness to consider PrEP. The median age was 24 years (IQR 21 to 28); 31% did not know their male partner's HIV status and 84% were married. Overall, 1856 (19%) of encounters led to PrEP initiation; only six women (<0.01%) were medically ineligible (creatinine clearance <50 min/mL). Frequency of PrEP initiation differed by male partner HIV status (HIV‐negative 7%, unknown 43%, HIV‐positive 79%, *p* < 0.001). PrEP initiation was more common in the postpartum period than during pregnancy (23% vs. 16%, *p* < 0.001). Women younger than 24 years of age were more likely than older women to initiate PrEP (OR = 1.18, 95% CI 1.08 to 1.28, *p* < 0.001). Initiating PrEP was also associated with having an STI (OR = 2.66, 95% CI 1.48 to 4.77, *p* = 0.001) and being forced to have sex in the last six months (OR = 3.69, 95% CI 1.69 to 8.06, *p* = 0.001). The most frequently reported reasons for declining PrEP were the perception that HIV risk was low (46%) and the partner was HIV‐negative (43%); few women accepting PrEP feared intimate partner violence as a result (2%).

**Conclusions: **In this pregnant and postpartum population, a substantial number of women desired and started PrEP. PrEP initiators were younger and more likely to have HIV risk factors than those who declined PrEP.

## WEAE0403

### How long will they take it? Oral pre‐exposure prophylaxis (PrEP) retention for female sex workers, men who have sex with men and young women in a demonstration project in Kenya

**J.K. Kyongo^1^; M. Kiragu^2^; R. Karuga^1^; C. Ochieng^1^; A. Ngunjiri^1^; C. Wachihi^3^; H. Musyoki^4^; L. Digolo^1^; L. Otiso^2^; L. Gelmon^3^; N. Kilonzo^5^ and W. Mukoma^6^**

^1^LVCT Health, Research & Strategic Information, Nairobi, Kenya, ^2^LVCT Health, HIV Services, Nairobi, Kenya, ^3^Partners for Health and Development in Africa, Nairobi, Kenya, ^4^National AIDS and STIs Control Program ^NASCOP^; Nairobi, Kenya, ^5^National AIDS Control Council ^NACC^; Nairobi, Kenya, ^6^LVCT Health, Directorate, Nairobi, Kenya

**Background:** Female sex workers (FSW), men who have sex with men (MSM) and young women (YW) account for majority of new infections in Kenya. Oral PrEP effectively protects against HIV infection but data on PrEP retention and influencing factors in real‐world settings is limited. We present data on retention on PrEP in a prospective oral PrEP demonstration project in Kenya.

**Methods:** Between August 2015 and October 2016, we enrolled 1585 participants; 528 (33%) FSW, 438 (28%) MSM and 619 (39%) YW; on oral PrEP. Two public and four private health facilities were used as points of care, with monthly PrEP refill and adherence/risk behavior counselling visits. Follow up duration was one year and participants did not receive reimbursements. Retention was defined as returning for PrEP refill as scheduled and assumed to imply PrEP use. Reasons for drop‐out and continuous use were documented through facility registers and in‐depth interviews and focus group discussions with PrEP users and health care workers (HCWs). Data was analysed using STATA (quantitative) and NVivo (qualitative).

**Results: ** Retention at one, three and six months was 40.3%, 26.3% and 14% for FSW; 32.9%, 21.7% and 14.8% for MSM and; 25.7%, 16.5% and 9.5% for YW. For all populations, the instantaneous hazard rate of terminating PrEP use was lowest at twenty weeks. FSW <23 years were more likely to drop out compared to older FSW (HR 0.76 (95% CI: 0.60 to 0.97) *p* < 0.029). No age variations were observed among MSM and YW. Reasons for choosing to stop using PrEP included reduced self‐perception of risk, sexual partner on successful antiretroviral therapy, community stigma, PrEP myths and misconceptions, risk of social harm, negative attitude from HCWs, challenges accessing study site and tedious procedures at health facilities. Motivators for continuous PrEP use include peer/guardian/partner support, access to combination HIV prevention services and social responsibility.

**Conclusions: **In our context, we observed high attrition from oral PrEP use by all populations but especially by young women. There are individual, community and health system barriers and enablers of continuous PrEP use. Most of these are modifiable and need to be considered by countries and programs scaling up oral PrEP.

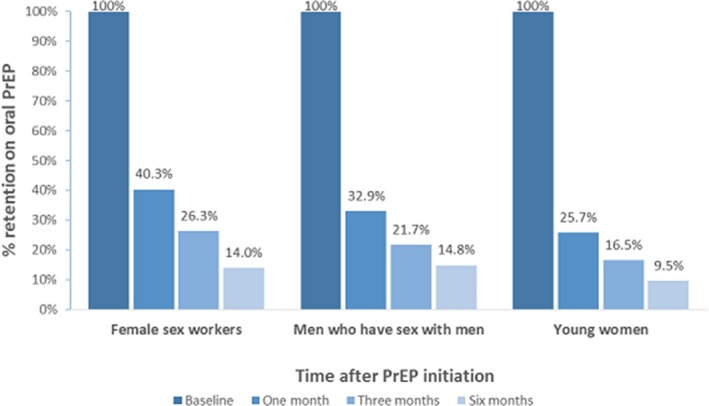

**Abstract WEAE0403‐Figure 1. Retention on oral pre‐exposure prophylaxis (PrEP).**

## WEAE0404

### Cost and impact of PrEP implementation in Haiti to adopt WHO recommendations: results from EQUIP

**R. Kolesar^1^; K. Rebe^2^; D. Lauture^3^; S.N. Magwaza^4,5^; W.J. Domercant^3^; E. Emmanuel^3^; M. Leonard^3^; M.‐L. Excellent^6^; F.J. Louis^3^ and EQUIP PrEP Technical Working Group**

^1^University of Boston (Affiliation), Public Health (Health Economics), El Malecón, Dominican Republic, ^2^ANOVA Health Institute, Health4Men, Cape Town, South Africa, ^3^Maternal Adolescent and Child Health Systems, EQUIP Innovation for Health, Port au Prince, Haiti, ^4^Maternal Adolescent and Child Health Systems, EQUIP Innovation for Health, Durban, South Africa, ^5^University of Witswaterand, School of Public Health, Johannesburg, South Africa, ^6^Maternal Adolescent and Child Health Systems, EQUIP Innovations for Health, Port au Prince, Haiti

**Background:** The World Health Organization expanded its recommendation on the provision of Pre‐Exposure Prophylaxis (PrEP) to include all people at substantial risk of HIV exposure to achieve the UNAIDS’ “Fast‐Track” strategy. The Haitian National AIDS Control Program included PrEP for key populations (KP) in the HIV/AIDS national strategic plan (2018 to 2023). Hence, EQUIP‐Haiti, conducted PrEP impact and cost modeling studies to measure effects of PrEP implementation to inform policy decision in Haiti.

**Methods:** *Goals* module of *Spectrum*, the UNAIDS analytical software, was used for estimating the effects of interventions on HIV infections and resource requirements. Key parameters included, unit cost, population size estimates, epidemiological and behavioral data, intervention coverage by sex, risk group, year, and effectiveness. The Haiti *Integrated Biological and Behavioral Surveillance* survey for KPs was used.. Comparison made of three scenarios for annual direct service costs between 2019 and 2023; proportion of KP‐initiated on PrEP and HIV infections averted by 2023. A 3‐step process estimated potential costs and impact of PrEP implementation:

(1) A cost component‐based approach identifying the specific cost elements to estimate an annual unit cost per person on PrEP;

(2) Simulations with different adherence rate measures and HIV treatment coverage assumptions with increments from 2018 to 2023;

(3) Estimating the impact and cost of scaling up condom use in the general population, and comparing result against the current status

**Results: ** The estimated first year PrEP unit cost per client is $228.87 that declines to $173.85 annually thereafter. Of the three scenarios, the second was feasible for Haiti assuming 80% adherence and 5% coverage in 2017 with a 10% increase from 2018 to 2023. The total direct cost was estimated at $2.9 to $3.1 million annually for direct service provision to 10% of KPs initiated within two years between the 2018 to 2023 period. This translates to 16,263 people on PrEP with a projected HIV aversion of 2, 528 cumulative infections by 2023.

**Conclusions: **The study results informed policy decision for PrEP implementation in Haiti. In the future, the proposed scenarios must consider integrated services and health system issues affecting implementation.

## WEAE0405

### The cost‐effectiveness of multi‐purpose HIV and pregnancy prevention technologies in South Africa

**M. Quaife^1,2^; F. Terris‐Prestholt^1^; R. Eakle^2^; M.A Cabrera Escobar^2^; M. Kilbourne‐Brook^3^; M. Mvundura^3^; G. Meyer‐Rath^4,5^; S. Delany‐Moretlwe^2^ and P. Vickerman^6^**

^1^London School of Hygiene and Tropical Medicine, Global Health and Development, London, United Kingdom, ^2^University of the Witwatersrand, Wits RHI, Johannesburg, South Africa, ^3^PATH, Seattle, United States, ^4^Boston University, Center for Global Health and Development, Boston, United States, ^5^University of the Witwatersrand, Health Economics and Epidemiology Research Office, Johannesburg, South Africa, ^6^University of Bristol, Bristol, United Kingdom

**Background:** A number of antiretroviral HIV prevention products are efficacious in preventing HIV infection. However, the sexual and reproductive health needs of many women extend beyond HIV prevention and research is ongoing to develop multi‐purpose prevention technologies (MPTs) that offer dual HIV and pregnancy protection. We do not know if these products will be an efficient use of constrained health resources. In this paper we estimate the cost‐effectiveness of combinations of candidate multi‐purpose prevention technologies (MPTs), in South Africa among general population women and female sex workers (FSWs).

**Methods:** We combined a cost model with a static model of product impact based on incidence data in South Africa to estimate the cost‐effectiveness of five candidate co‐formulated or co‐provided MPTs: oral PrEP, intravaginal ring, injectable ARV, microbicide gel, and SILCS diaphragm used in concert with gel. We accounted for the preferences of end‐users by predicting uptake using a discrete choice experiment (DCE). Product availability and protection were systematically varied in five potential rollout scenarios.The comparator for each scenario was current levels of male condom use, while a health system perspective was used to estimate discounted lifetime treatment costs averted per HIV infection. Product benefit was estimated in disability‐adjusted life years (DALYs) averted. Benefits from contraception were incorporated through adjusting the uptake of these products based on the DCE and through estimating the costs averted from avoiding unwanted pregnancies.

**Results: ** At central incidence rates, all single‐ and multi‐purpose scenarios modelled were cost‐effective among FSWs and women aged 16 to 24, at a governmental willingness‐to‐pay threshold of $1,175/DALY averted (range: $214 to $810/DALY averted among non‐dominant scenarios), however none were cost‐effective among women aged 25 to 49 (minimum $1,706/DALY averted). The cost‐effectiveness of products improved with additional protection from pregnancy. Estimates were sensitive to variation in incidence assumptions, but robust to other parameters.

**Conclusions: **To our knowledge, this is the first study to estimate the cost‐effectiveness of a range of potential MPTs; suggesting that MPTs will be cost‐effectives amongst higher incidence FSWs or young women but not lower incidence older women. More work is needed to make attractive MPTs available to potential users who could use them effectively.

## WEAE0501

### Ten years of Community ART Groups (CAG): retention and viral load uptake in Tete, Mozambique

**L. Haldna and A.W. Torrens**

Medecins Sans Frontieres, Operational Center Brussels, Brussels, Belgium

**Background:** Community ART groups (CAG), peer support groups involved in community ART distribution and mutual psychosocial support, were piloted by MSF in 2008 to respond staggering ART attrition in Tete, Mozambique. Ten years later, outcomes of CAG were analyzed – whether community‐based care in rural setting has increased retention and improved clinical outcomes.

**Methods:** Retrospective cohort design was used with data from HIV electronic register and CAG group register. A total of 2167 patients from 2008 to 2017 ever registered in CAG in Changara and Marara districts were included in the analysis. Kaplan‐Meier techniques were used for estimating mortality and lost to follow‐up (LTFU) rates per 100 person‐years. Individual level predictors of attrition were assessed using logistic regression and chi‐square tests.

**Results: ** Mortality, LTFU and returning to individual care rates among 2167 CAG members were, respectively, 2.3, 1.6 and 1.6 per 100 person‐years. Long‐term retention in HIV care was found high: 93.1% at two years, 90.2% at four years and 87.5% at nine years. Retention did not decrease significantly after 4th year (*p* = 0.4006) in care. Patients who never had viral load (VL) monitored (*a*OR 4.266, 95% CI 3.34 to 5.46) or had unsuppressed VL (*a*OR 3.954, 95% CI 2.58 to 6.07) were at higher risk of LTFU or death. 53.9% of patients with VL ≥1000 copies/mL were part of CAG, while unsuppressed VL is CAG exclusion criteria. 15.32% of patients had joined CAG with advanced HIV (CD4 <200 cells/μL), these patients were at higher risk of attrition (*a*OR 1.861, 95% CI 1.36 to 2.54).

**Conclusions: **Long‐term retention was exceptionally high, especially for a rural population, confirming positive results from previous studies. Nevertheless, finding about outcomes indicate that to reduce attrition, efforts are needed to strengthen the detection of PLWHA on earlier stage and to ensure clinical follow‐up and VL routine monitoring. Risk factors associated with attrition demonstrate crucial added value of CAG model as peer‐to‐peer support and not only as community provision of ART. Results of this study have potential benefit to the global HIV response to provide out‐of‐clinic treatment to increasing number of patients, moreover it is a comprehensive insight how community‐based care has functioned over a long period of time.

## WEAE0502

### Increasing retention in care through community systems strengthening: lessons learned from 15 districts in South Africa

**M.R. Manganye^1^; S. Berrada^1^; A. Wagner^1^; S. Gombarume^2^; C. Wylie^1^ and A. Make^1^**

^1^Hospice Palliative Care Association of South Africa, CaSIPO – Care and Support for Improved Patient Outcomes, Cape Town, South Africa, ^2^USAID/Southern Africa, Care and Treatment, Pretoria, South Africa

**Background:** The United States Agency for International Development‐funded Care and Support to Improve Patients Outcomes Project (CaSIPO) aims to maximize the quality of life for patients on antiretroviral treatment (ART) through provision of comprehensive care and support services in the community. As the South African Government vows to provide access to treatment to all people living with HIV and AIDS, adherence to treatment and retention in care are critical to the sustainability of the ART program.

**Description:** CaSIPO works with 15 high burden Districts to strengthen community systems to promote patients’ retention in care. Community adherence clubs (CACs) offer a much‐needed adherence support structure to stable patients on ART along convenient access to their medications. CaSIPO uses innovative approaches to adapt to local context and establish CACs in rural, semi‐rural and urban settings. Working closely with community based organizations (CBOs), ward based outreach teams and clinics, CaSIPO developed their skills and knowledge to provide adherence support, health promotion, nutrition assessment counselling and support, screening for tuberculosis and sexually transmitted infections, family planning education and active referral.

**Lessons learned:** CaSIPO undertook various capacity development strategies to strengthen the CBOs’ systems and structures and empower them with the necessary tools to provide patients‐centred sustainable adherence support. Between June 2016 and December 2017, CaSIPO empowered 194 CBOs across 15 Districts to provide quality comprehensive care and support services to 110,994 HIV stable patients attending 4526 CACs. Quality is a key driver of retention in care. Through training, mentorship and intensified technical assistance program, CaSIPO established a quality assurance system for community based services resulting in 96% retention in CACs (n = 106,781; d = 110,994; December 2017). Over 18‐month period, 4213 of 110,994 CACs patients had exited the clubs, with 69% (2,907) transferred to other decanting modalities and only 7% (295) lost to follow up.

**Conclusions/Next steps:** South Africa unique and complex public health dynamic led CaSIPO to adopt a mixed approach to provide sustainable and scalable community adherence support structure to ART patients. Strengthening the community system and facilitating linkages between community and health facilities has proven to be critical in promoting retention in care.

## WEAE0503

### Same day ART initiation does not reduce 12‐month retention among HIV‐infected children in Uganda

**A. Kekitiinwa^1^; P. Elyanu^2^; E. Nazziwa^3^; G.P. Kisitu^2^; R.N. Ssebunya^2^; C. Katureebe^4^; G. Namayanja^5^; I. Lukabwe^4^; E.N. Magongo^4^; A. Namale^3^; P.N. Ntege^2^ and M.R. Adler^3^**

^1^Baylor College of Medicine Children's Foundation, Pediatrics and Child Health, Kampala, Uganda, ^2^Baylor College of Medicine Children's Foundation, Kampala, Uganda, ^3^Centre for Disease Control, Entebbe, Uganda, ^4^Ministry of Health, Kampala, Uganda, ^5^Centre for Disease Control, Kampala, Uganda

**Background:** Countries have adapted the WHO Test and START guidelines for antiretroviral therapy (ART) initiation, but there is limited evidence on how soon to start ART in children without jeopardizing retention. Uganda has implemented Test and START guidelines for children which included same day initiation since 2014. We compared 12‐month probability of retention in HIV‐infected children aged <15 years initiated on ART on the same day of diagnosis versus two to fourteen days or >14 days from diagnosis during HIV Test and START policy implementation in Uganda.

**Methods:** We retrospectively reviewed clinic charts for HIV‐infected children diagnosed and initiated on ART from June 2014 to March 2015 in 42 health facilities in Uganda. Retention was defined as being alive and on ART during the 12th month on ART. Kaplan‐Meier estimates were used to calculate the 12‐month probability of retention (overall and stratified by health facility level and age at diagnosis) and the log‐rank test to compare groups.

**Results: ** Of 899 HIV infected children, 115 (12.8%) were excluded for missing diagnosis date and 784 (87.2%) were included in the analysis. Of these 784 children, 56% were girls, median ages (IQR) at diagnosis and ART initiation was 3 (1, 7) years and 4 (1, 7) years respectively. Three hundred and seventeen (40.4%) children started ART on the same day they were diagnosed, 155 (19.8%) started within two to fourteen days and 312 (39.8%) started after 14 days. The overall probability of one‐year retention was 89.9%; Retention was similar among children who initiated ART on the same day (89.7%) compared to those initiated two to fourteen day's (94.0%) and >14 days (90.2%), *p* = 0.3. Retention was highest in those diagnosed at age five to fourteen years (93.5%) and at health centres (96.0%); and lowest in children under two years of age (85.9%) and regional referral hospitals (89.7%). Retention in each age and health facility stratum did not differ by time from diagnosis to initiation (table 1).

**Conclusions: **Starting ART in children on the same day of diagnosis does not jeopardize retention on treatment.

**Abstract WEAE0503‐Table 1. 12 month ART retention by time from diagnosis to ART initiation in HIV infected children aged under 15 years, Uganda 2017 (N = 784)**

**Table:** 

Characteristic		All		Same Day N = 317	2 to 14 days N = 312	>14 days N = 312	*p*‐Value*
		# Retained/Total	Incidence (95% CI)	Incidence (95% CI)	Incidence (95% CI)	Incidence (95% CI)	
Overall	Crude	713/784	89.9% (87.5%, 91.8%)	89.7% (85.8%, 92.6%)	94.0% (88.8%,96.9%)	90.2% (86.3%, 93%)	0.3
Health Facility	Regional referral hospital	395/435	89.7% (86.6%, 92.1%)	90.6% (85.3%, 94.1%)	92.6% (85.2%, 96.4%)	89.1% (82.8%, 93.2%)	0.6
	General Hospital	192/208	90.3% (85.8%, 93.4%)	90.7% (81.4%, 95.4%)	97.0% (81.0%, 99.0%)	91.8% (84.2%, 95.8%)	0.5
	Health centres	126/141	96.0% (88.6%, 98.8%)	85.6% (73.3%, 92.5%)	95.5% (71.9%, 99.0%)	90.3% (79.7%, 95.5%)	0.4
By age at diagnosis	under two years	220/255	85.9% (81.0%, 89.7%)	85.7% (77.1%, 91.3%)	90.4% (76.4%, 96.3%)	84.4% (76.1%, 90%)	0.7
	Two to four years	195/211	92.3% (87.8%, 95.2%)	90.9% (82.6%, 95.3%)	95.6% (83.4%, 98.9%)	92.0% (83.0%, 96.3%)	0.6
	Five to fourteen years	298/318	93.5% (90.1%, 95.8%)	92.0% (85.6%, 95.8%)	95.3% (86.0%, 98.4%)	94.2% (88.2%, 97.2%)	0.7

## WEAE0504

### Community patient tracking by Lay Community Health Workers (CHWs) is an effective strategy towards the second & third 90

**B.M. Morapedi; G. Morineau; C. Lesedi; J.M. Irige; B. Mantu; O. Sokwe; C. Pheko and K. Poloko**

FHI 360, APC Botswana Project, Gaborone, Botswana

**Background:** In June 2017, Botswana introduced “Treat all” to facilitate universal coverage of antiretroviral treatment (ART) towards the 90‐90‐90 targets. Treat all provides access to ART to Botswana citizens irrespective of CD4 count. FHI 360, Advancing Partners and communities (APC) project, funded by PEPFAR through USAID in Botswana built the capacity of communities towards epidemic control by introducing community health workers (CHWs) to support health facilities trace patients with known sero‐positive status who never started ART and defaulters, in eight districts. Health facility staff traditionally trace defaulters through phone calls.

**Description:** Working in collaboration with the Ministry of Health & Wellness (MoHW) District Health Management Teams (DHMTs), CHWs were trained on community HIV care including patient tracking strategies and provided standard operating procedures (SOPs) for guidance. Senior Community Health Workers (SCHWs) were posted in health facilities and together with nurses in the infectious Disease Control Centres (IDCC) identified patients that were in the registers but never started ART or defaulted.

A team of CHWs was assigned a caseload of patients to track in the community. Successfully traced patients were linked to facilities through unaccompanied and accompanied referrals. They were also provided information on “Treat All” to support them to opt for ART initiation.

**Lessons learned:** During the period October 2016 to September 2017, out of 1277 clients referred to APC CHWs for tracing, 584 (46%) included sufficient information in their clinical file for tracing. From those traced, 317 (54%) linked back to health facilities and initiated on ART, 146 (25%) were found already on ART, 46 (8%) were referred but did not link, 40 (7%) had changed physical address, 27 (5%) declined to be linked back to care, and eight were reported deceased.

**Conclusions/Next steps:** Implementation of community patient tracking contributed to linkage of PLHIV to ART and updating patient facility records. Home visits by CHWs increases uptake of ART services. CHWs are critical to fill the human resource constraints within the health system and their knowledge of their community ensures successful tracing of those lost by the health system. Tracking patients that have defaulted from ART is resource intensive but worth it.

## WEAE0505

### “My best friends and my worst enemies:” understanding the roles of families in retaining South African adolescents living with HIV in care

**T.D. Ritchwood^1^; N. Ntlapo^2^; M. Atujuna^2^; S. Letoao^3^; A. Oduro^2^ and L.G. Bekker^2^**

^1^Medical University of South Carolina, Public Health Sciences, North Charleston, United States, ^2^University of Cape Town, Desmond Tutu HIV Centre, Observatory, South Africa, ^3^Information Health Measurement, Mbabane, Swaziland

**Background:** Adolescents living with HIV (ALWH) are less likely than their child or adult peers to be retained in HIV care and adhere to their medication regimens. While familial involvement is critical to the treatment success of ALWH, most of interventions for this group focus only on individual‐level factors and do not include family members. This current study investigated the role of families in ALWHs’ treatment retention and adherence and solicited strategies from ALWH, their caregivers, and local stakeholders to better integrate families in intervention efforts.

**Methods:** Fifty‐nine semi‐structured, in‐depth interviews were conducted to qualitatively determine how the families of ALWH support or hinder their treatment retention and adherence and to determine how best to include them in intervention efforts. Participants were ALWH (n = 20; 13 to 19 years of age), their caregivers (n = 19), and local stakeholders (n = 20) from Cape Town, South Africa. Data were coded and analyzed using inductive content analyses. We then grouped codes into perceived positive and negative familial roles, and suggestions on how families could help to improve ALWH treatment retention and adherence.

**Results: ** Findings revealed several positive roles that family members served in supporting ALWH, including: reminding them to take their pills; reinforcing notions of personal accountability; providing informational, instrumental, and emotional support; assisting youth when their primary caregiver was unavailable; and normalizing pill taking. While most participants identified their families as sources of support, a number of participants expressed negative familial roles, including being sources of discrimination, ridicule, and discord; and lacking a role entirely. Moreover, participants reported that HIV disclosure within families is often challenging, leaving many ALWH with no one with whom they can confide within their social networks, limiting their family's ability to provide critical support. ALWH suggested that their families assist with retrieving medications from pharmacies and commit to unconditional acceptance, regardless of whether they adhere to their treatment regimens.

**Conclusions: **Families play an important role in the adolescent HIV treatment cascade and could both facilitate and derail treatment retention and adherence. This study highlights the need for systematic, intergenerational approaches that incorporate socio‐structural factors in efforts to retain ALWH in HIV care.

## THAA0101

### HIV genotyping and phylogenetics in the HPTN 071 (PopART) study: validation of a high‐throughput sequencing assay for viral load quantification, genotyping, resistance testing and high‐resolution transmission networking

**D. Bonsall^1,2^; T. Golubchik^1^; B. Kosloff^3,4^; M. Limbada^3,4^; M. de Cesare^2^; A. Schaap^3,4^; M. Hall^1^; C. Wymant^1^; G. Macintyre‐Cockett^2^; A. Brown^5^; M.A. Ansari^5^; S. Floyd^4^; R. Hayes^4^; H. Ayles^3,4^; S. Fidler^6^; C. Fraser^1^ and HPTN 071 (PopART) Study Group**

^1^Big Data Institute, University of Oxford, Nuffield Department of Medicine, Oxford, United Kingdom, ^2^Wellcome Trust Centre for Human Genetics, University of Oxford, Nuffield Department of Medicine, Oxford, United Kingdom, ^3^The Zambia AIDS Related Tuberculosis (ZAMBART) Project, Lusaka, Zambia, ^4^London School of Hygiene and Tropical Medicine, London, United Kingdom, ^5^Peter Medawar Building for Pathogen Research, University of Oxford, Nuffield Department of Medicine, Oxford, United Kingdom, ^6^Imperial College, Department of Clinical Medicine, London, United Kingdom

**Background:** Next‐generation sequencing has been transformative to molecular epidemiology of viruses, though technical complexities have slowed adoption in clinical settings. We developed a quantitative genotyping method optimised for a large‐scale HIV phylogenetic study linked to the HPTN 071 (PopART) cluster‐randomised trial of antiretroviral treatment as prevention in Zambia.

**Methods:** To date, 4319 HIV‐positive patients (ART‐naive or >1 year since last ART) from 9 health care facilities have consented to viral sequencing using residual blood from CD4 testing collected at recruitment. Nucleic acid extracted from 0.5 mL plasma from 292 male and 357 female HIV‐infected participants was used to produce sequencing libraries without virus‐specific PCR. Oligonucleotide‐baits, designed to capture the full HIV epidemic diversity enriched libraries for sequencing on MiSeq (Illumina) and portable MinION (Oxford Nanopore) sequencers. Optimizations focused on the simplest, most cost‐effective means of maximizing numbers of unique viral RNA templates, including samples with low viral load, while preserving quantitative information and minimizing oversampling of short‐RNA fragments. Merged paired‐end reads were assembled using SHIVER, depleted of PCR‐duplicates and contaminants, then submitted for high‐resolution transmission‐mapping (phyloscanner) and drug resistance profiling (HIVdb, Stanford). Quantitative sequencing controls were used to estimate viral load. Clinical viral loads were obtained for a random subset of samples (n = 126) for cross validation.

**Results: ** Whole genomes were obtained (with minimum depth of 5x) for 80% of all samples and for 97% of samples with clinical viral load >1,000 copies/mL (fig A). Sequence‐based viral load correlated with clinical viral load (R2  = 88% and n = 126, fig B). By minimizing PCR‐amplification of short fragments, a median of 49% of inserts were longer than 350 b.p providing sufficient phylogenetic resolution to assess intrahost diversity, identify transmission pairs and potentially estimate recency of infection. The total processing time from RNA extraction to sequencing can be completed in 48 hours per batch of 90 samples.

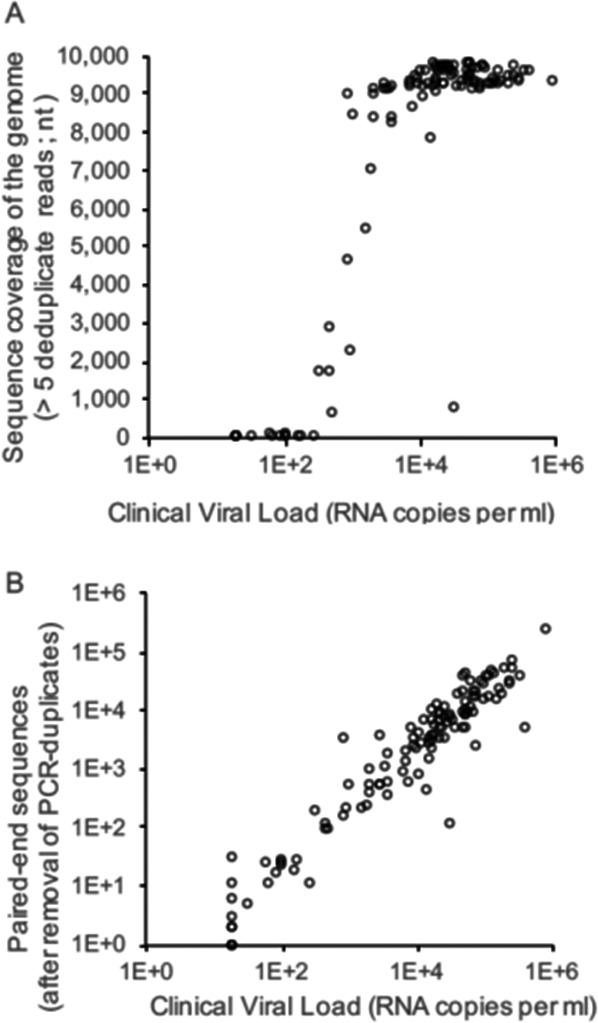

**Abstract THAA0101‐Figure 1. Quantitative sequencing of HIV.**

**Conclusions: ** Our novel laboratory and informatics pipeline provides robust viral genetic, viral load, and minority variant information. Processing times, cost and capabilities for handling low viral load samples are highly‐competitive compared to routine viral load or polymerase drug resistance testing and suitable for clinical use.

## THAA0102

### High levels of adaptation to host cellular immunity in a concentrated North American HIV epidemic

**N.N. Kinloch^1^; S. Sanche^2^; A. Wong^3^; E. Martin^1^; K.D. Cobarrubias^1^; P. Sandstrom^4^; P. Levett^5,6^; P.R. Harrigan^7,8^; J.B. Joy^7,8^ and Z.L. Brumme^1,7^**

^1^Simon Fraser University, Faculty of Health Sciences, Burnaby, Canada, ^2^University of Saskatchewan, College of Medicine, Saskatoon, Canada, ^3^University of Saskatchewan, College of Medicine, Regina, Canada, ^4^National HIV Retrovirology Laboratories, Public Health Agency of Canada, Winnipeg, Canada, ^5^Saskatchewan Disease Control Laboratory, Regina, Canada, ^6^University of Regina, Department of Biology, Regina, Canada, ^7^British Columbia Centre for Excellence in HIV/AIDS, Vancouver, Canada, ^8^University of British Columbia, Faculty of Medicine, Vancouver, Canada

**Background:** Globally, the spread of viral HLA‐associated escape mutations in HIV epidemics has been linked to rapid disease progression and poor clinical outcomes. HIV diagnosis rates in the Canadian province of Saskatchewan are the highest nationwide, and reports of unusually rapid progression have also emerged from the province. Accelerated progression among individuals expressing certain HLA alleles, including the typically protective B*51, has also been reported in neighbouring areas; moreover, regional HLA surveys reveal HLA‐B*51, B*35 and B*15 as the most frequently observed HLA‐B alleles in at‐risk populations in the region. Here, we tested the hypothesis that HIV adaptation to common HLA alleles, in particular B*51, is elevated in Saskatchewan.

**Methods:** We analyzed 1144 partial HIV subtype B Pol sequences from unique Saskatchewan residents collected between 2000 and 2016 for drug resistance genotyping, alongside >6500 published Pol sequences from elsewhere in Canada and the USA from the same period, for the presence of 70 published HLA‐associated Pol mutations. Overall HIV adaptation levels to 34 individual HLA alleles were also compared between these regions. Phylogenetic methods were used to identify putative HIV transmission clusters and the distribution of HLA‐associated adaptations within and external to these clusters was investigated.

**Results: ** HIV molecular epidemiology in Saskatchewan is unique: >75% of sequences resided within 26 large phylogenetic clusters (largest >200 sequences). HIV adaptation to numerous HLA alleles, notably B*51, B*15 and others, was also significantly elevated. For example, the Saskatchewan Pol consensus sequence differs from the North American consensus at 9 codons, 7 of which represent major HLA‐associated escape mutations. Adaptation levels are also increasing over time. HLA‐adapted HIV strains are significantly enriched in Saskatchewan transmission clusters, indicating that these are being widely and preferentially transmitted within the province.

**Conclusions: **Results indicate that a substantial proportion of at‐risk individuals in Saskatchewan have a high probability of acquiring HIV that is pre‐adapted to host immunity, providing a plausible explanation for reports of accelerated progression. Results also identify Saskatchewan as the first North American HIV epidemic featuring significant circulating HLA adaptation and highlight the urgent need to expand HIV prevention, testing and treatment in this region.

## THAA0103

### HIV‐1 exposure enhances sexual transmission of hepatitis C virus by human mucosal Langerhans cells

**B.M. Nijmeijer^1^; R. Sarrami Forooshani^1^; G.S. Steba^2^; R.R.C.E. Scheurs^1^; S.M. Koekkoek^2^; R. Molenkamp^2^; J. Schinkel^3^; P. Reiss^4,5^; M.L. Siegenbeek vanHeukelom^5,6^; M. van derValk^5^; C.M.S. Ribeiro^1^ and T.B.H. Geijtenbeek^1^**

^1^Academic Medical Center – University of Amsterdam – Amsterdam Infection and Immunity Institute, Experimental Immunology, Amsterdam, Netherlands, ^2^Academic Medical Center, University of Amsterdam, Department of Medical Microbiology, Clinical Virology Laboratory, Amsterdam, Netherlands, ^3^Academic Medical Center, University of Amsterdam, Department of Medical Microbiology, Clinical Virology laboratory, Amsterdam, Netherlands, ^4^Academic Medical Center – Amsterdam Institute for Global Health and Development, Department of Global Health, Amsterdam, Netherlands, ^5^Amsterdam Infection and Immunity Institute, Academic Medical Center – University of Amsterdam, Department of Internal Medicine, Division of Infectious Diseases, Amsterdam, Netherlands, ^6^Academic Medical Center – University of Amsterdam, Department of Dermatology, Amsterdam, Netherlands

**Background:** Sexual transmission of Hepatitis C virus (HCV), until recently, was thought to be rare. However, there has been a significant rise in the incidence of HCV infection among HIV‐infected men‐who‐have‐sex‐with‐men (MSM) and studies suggest that HCV can be sexually transmitted within this population. The mechanisms underlying this sexual transmission are unclear. Syndecans, a familiy of cell surface heparan sulfate proteoglycans have been shown to act as attachment receptors to transmit viruses. In this study we investigated the role of Syndecans on LCs in HCV infection and transmission. We hypothesized that HIV‐1 replication in HIV‐1‐infected MSM leads to mucosal changes that allow HCV entry and subsequent dissemination to hepatocytes via Syndecan‐4 on activated LCs.

**Methods:** Therefore, we analyzed the immune cells within mucosal anal biopsies from HIV‐1 infected MSM individuals as a potential entry route for HCV during sexual contact. We investigated the role of LCs in HCV infection and transmission using human primary isolated LCs and the *ex vivo* tissue transmission model.

**Results: ** Notably, we detected Langerhans cells (LCs) within the mucosal anal tissue. Immature LCs were neither infected nor transmitted HCV to hepatocytes *in vitro* and *ex vivo*. As sexual transmission is mostly observed within HIV‐1 infected individuals, we pre‐exposed tissues with HIV‐1 and, strikingly, HIV‐1 pre‐exposure significantly increased HCV transmission by LCs. Active HIV‐1 replication is crucial for the increased HCV transmission as treating *ex vivo* tissue with HIV‐1 replication inhibitors significantly decreased HIV‐1‐induced HCV transmission. Activation of LCs did not lead to infection by HCV but these activated LCs, in contrast to immature LCs from same donor, were efficient in transmitting HCV to hepatocytes. Notably, B cell‐line expressing Syndecan‐4 in contrast to other Syndecans was very efficient in transmitting HCV. Furthermore, silencing of Syndecan‐4 on activated LCs decreased HCV transmission.

**Conclusions: **Thus, our data strongly suggest that HIV‐1 replication in mucosal tissues in HIV‐1 infected MSM, changes LC function, allowing Syndecan‐4 to capture and subsequently transmit HCV to hepatocytes. This novel transmission mechanism by LCs implicates also that the activation state of LCs is an important determinant for HCV susceptibility after sexual contact.

## THAA0104

### Prophylactic and therapeutic efficacy of broadly neutralizing antibody PGDM1400 against HIV‐1 in humanized mice

**Y. van derVelden^1^; J. Villaudy^1,2^; E. Siteur – van Rijnstra^1^; C. van derLinden^1^; E. Frankin^1^; M. Vink^1^; P. van derWoude^1^; E. Schermer^1^; B. Berkhout^1^; R.W. Sanders^1^ and M.J. van Gils^1^**

^1^AMC, University of Amsterdam, Amsterdam, Netherlands, ^2^AIMM Therapeutics, Amsterdam, Netherlands

**Background:** Broadly neutralizing antibodies (bNAbs) show promise as prophylactics and therapeutics against HIV‐1 in macaques and humans. The introduction of new approaches in B cell isolation reinvigorated the field and led to identification of unusually broad and potent bNAbs. One example is PGDM1400, which neutralizes around 83% of virus strain, and at very low concentrations, making it a very attractive antibody for prophylactic and therapeutic applications.

**Methods:** A hu‐HSC NSG mouse model, which has the complete human immune system, was used to study the protective and therapeutic efficacy of the bNAb PGDM1400. Mice were passively immunized (i.v.) with different concentration of PGDM1400 and challenged high dose i.p. with HIV‐1JRCSF 24 hours later. In a second experiment mice were infected with viruses from clades A, B or C (HIV‐1BG505, HIV‐1REJO, HIV‐1AMC008, or HIV‐1 MJ4) and 12 weeks later, a high dose of PGDM1400 was given (i.v.) once a week for four weeks. Viral populations were sequenced before and after the administration of PGDM1400.

**Results: ** In the passive immunization study, all animals in the 10 mg/kg and 3 mg/kg groups, and four out of seven animals in the 1 mg/kg group had undetectable levels of viral RNA, while all animals of the PBS group and the antibody control group had high levels of viral RNA seven days after challenge. Subsequently, PGDM1400 administered during chronic infection, caused a modest decrease in viral load and also an increase in CD4^+^ T cell counts in the first one to two weeks of administration in some animals, however these levels did not persist, suggesting that the virus escaped from PGDM1400, quickly after administration of the antibody. This was confirmed by the sequence analysis of the viral population after PGDM1400 therapy.

**Conclusions: **We showed that PGDM1400 is a promising component of a future prophylactic to prevent HIV‐1 infection. In agreement with studies on other bNAbs, PGDM1400 monotherapy did not fully suppress chronic HIV‐1 infection for a prolonged period, however considering that PGDM1400 is one of the most potent and broad bNAbs known, it can be an important component in therapeutic combination of different bNAbs.

## THAA0105

### A single dose of anti‐HIV‐1 antibodies can protect macaques from repeated mucosal SHIV exposures for six months

**R. Gautam^1^; Y. Nishimura^2^; N. Gaughan^2^; A. Gazumyan^3^; T. Schoofs^3^; A.‐B. White^2^; M. Seaman^4^; B. Swihart^2^; D. Follmann^2^; M. Nussenzweig^3^ and M. Martin^2^**

^1^NIH, Molecular Microbiology, Bethesda, United States, ^2^NIH, Bethesda, United States, ^3^Rockefeller University, New York, United States, ^4^BIDMC, Boston, United States

**Background:** In the absence of an effective and safe vaccine against HIV‐1, the administration of broadly neutralizing antibodies (bNAbs) represents a logical alternative approach to prevent virus transmission. bNAbs are capable of neutralizing most circulating strains, targeting different non‐overlapping epitopes on the HIV‐1 envelope spike.

**Methods:** We introduced two amino acid mutations (M428L and N434S (referred to as “LS”)) into the Fc domains of the highly potent HIV‐specific 3BNC117 and 10‐1074 bNAbs to increase their half‐lives and evaluated their efficacy in blocking infections following repeated low dose mucosal challenges of rhesus macaques with the Tier 2 SHIVAD8‐EO. The protective efficacy of 3BNC117‐LS or 10‐1074‐LS was assessed following a single intravenous infusion of each mAb (20 mg/kg body weight) to six animals. The macaques were challenged beginning one‐week after bNAb administration, and, in addition to viral RNA, we measured serum bNAb concentrations, anti SHIV‐neutralizing titers, and anti‐bNAb responses.

**Results: ** The most striking result obtained in this study was the long period of protective efficacy conferred by a single injection of Fc modified human anti‐HIV‐1 neutralizing antibodies in macaques compared to the previous studies. A single intravenous infusion of 10‐1074‐LS mAb markedly delayed virus acquisition for 18 to 37 weeks (median = 27 weeks) whereas the protective effect of the 3BNC117‐LS bNAb was more modest (protection for 11 to 23 weeks; median = 17 weeks). Serum concentrations of the 10‐1074‐LS mAb gradually declined and became undetectable in all recipients between weeks 26 to 41 whereas the 3BNC117‐LS bNAb exhibited a shorter half‐life. To model immunoprophylaxis against genetically diverse and/or neutralization resistant HIV‐1 strains, a combination of the 3BNC117‐LS plus 10‐1074‐LS mAbs was injected into macaques by the more clinically relevant subcutaneous route. Even though nearly three‐fold less of each bNAb in the mixture was administered, compared to the amount of single mAb injected in the intravenous infusions, the mAb combination still protected macaques for a median of 20 weeks.

**Conclusions: **The extended period of protection observed in macaques for the 3BNC117‐LS plus 10‐1074‐LS combination could translate into an effective semi‐annual or annual immunoprophylaxis regimen for preventing HIV‐1 infections in humans.

## THAB0101

### Low prevalence of calcified coronary plaque among Ugandans with and without HIV infection: comparison with a United States cohort and associations with biomarkers of inflammation

**C.T. Longenecker^1,2^; B. Alencherry^2^; G. Erem^3,4^; G. Mirembe^5^; J. Nansimbe^5^; I. Ssinabulya^4,6^; C.‐H. Yun^7^; C.‐L. Hung^7^; M.J. Siedner^8,9^; C. Kityo^5^ and G.A. McComsey^1,2^**

^1^Case Western Reserve University School of Medicine, Cleveland, United States, ^2^University Hospitals Cleveland Medical Center, Cleveland, United States, ^3^Nsambya St. Francis Hospital, Kampala, Uganda, ^4^Makerere University School of Medicine, Kampala, Uganda, ^5^Joint Clinical Research Centre, Kampala, Uganda, ^6^Uganda Heart Institute, Kampala, Uganda, ^7^Mackay Memorial Hospital, Taipei, Taiwan, Province of China, ^8^Massachusetts General Hospital, Boston, United States, ^9^Harvard Medical School, Boston, United States

**Background:** Little is known about subclinical coronary disease among people living with HIV (PLWH) in sub‐Saharan Africa. We sought to compare prevalence of calcified coronary plaque between people with and without HIV in Uganda and the United States, and to explore associations with HIV‐specific factors and biomarkers.

**Methods:** 100 Ugandan PLWH on antiretroviral therapy over 40 years old were prospectively age‐ and sex‐matched to 100 HIV‐uninfected controls. All had ≥1 major cardiovascular risk factor (hypertension, diabetes, smoking, or high cholesterol). Coronary artery calcium (CAC) scores were obtained from gated non‐contrast computed tomography scans of the heart. Biomarkers were measured from cryopreserved plasma. Ugandan subjects were compared to PLWH on antiretroviral therapy (n = 167) and uninfected controls (n = 63) over 40 years old from a research database in Cleveland, USA.

**Results: ** Compared to US subjects, Ugandans were older (mean age 56 vs. 52 years), had higher cholesterol (mean fasting cholesterol 216 vs. 176 mg/dL), more diabetes (36 vs. 3%), and more hypertension (85 vs. 36%); but were less likely to be male (38 vs. 74%), smokers (4 vs. 56%) or statin users (6 vs. 13%; all *p* < 0.01). Ugandan PLWH had lower current CD4+ (mean 570 vs. 675, *p* = 0.015), but similar nadir CD4 +  compared to US PLWH. The unadjusted prevalence of CAC>0 was higher in the US vs. Uganda and among PLWH vs. uninfected controls (Figure 1). After adjustment for HIV serostatus, age, sex, and traditional risk factors, Ugandans had over 13x lower odds of CAC>0 (*p* < 0.001). In multivariable‐adjusted models, soluble intercellular adhesion molecule (*p* = 0.044), soluble CD163 (*p* = 0.004), and oxidized LDL (*p* = 0.043) were each associated with CAC>0 among Ugandans. Soluble CD163 and oxidized LDL remained associated (*p* < 0.025) in models of Ugandan PLWH that further adjusted for PI use and current CD4 + . Among all PLWH (n = 267), nadir CD4 +  was the only HIV‐specific variable associated with CAC>0 in adjusted models.

**Conclusions: **Despite a high burden of risk factors, this Ugandan cohort of PLWH and controls had substantially lower rates of calcified coronary plaque compared to a US cohort. Lower nadir CD4 +  count and higher systemic levels of inflammation and immune activation were associated with CAC>0.

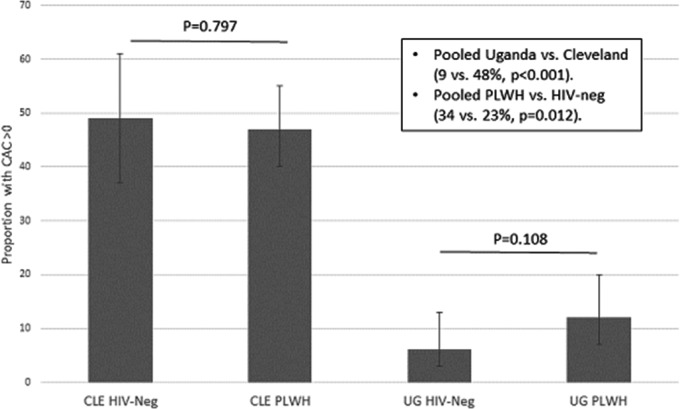

**Abstract THAB0101‐Figure 1.**

## THAB0102

### Cardiac chamber abnormalities and left ventricular mass in people living with HIV and matched uninfected controls assessed by multidetector computed tomography

**L. Demmer^1^; A. Ronit^1^; P.E. Sigvardsen^2^; A.‐M. Lebech^3^; J. Gerstoft^1^; A.D. Knudsen^1^; A. Fuchs^2^; J.T. Kühl^2^; J. Lundgren^1^; B.G. Nordestgaard^4^; K.F. Kofoed^2^ and S.D. Nielsen^1^**

^1^Rigshospitalet, Viro‐Immunology Research Unit, Copenhagen, Denmark, ^2^Rigshospitalet, Heart Center, Copenhagen, Denmark, ^3^University of Copenhagen, Department of Infectious Diseases, Hvidovre, Denmark, ^4^Herlev Hospital, Copenhagen General Population Study, Herlev, Denmark

**Background:** People living with HIV (PLWH) have increased risk of cardiovascular disease (CVD). Previous studies using echocardiography have reported higher prevalence of cardiac abnormalities including left atrial enlargement, left ventricle dysfunction, right ventricle dilatation and greater left ventricular mass in PLWH. Multidetector computed tomography (MDCT) allows a precise estimate of cardiac chamber volumes. We aimed to assess cardiac structural abnormalities and factors associated with cardiac abnormalities in PLWH and uninfected controls using MDCT.

**Methods:** A total of 592 PLWH from the Copenhagen co‐morbidity in HIV‐infection (COCOMO) study and 1184 age and sex matched uninfected controls from the Copenhagen General Population Study were included. Left atrial volume (LAV), left ventricular diastolic volume (LVDV), right ventricular diastolic volume (RVDV) and left ventricular mass (LVM) were assessed with semi‐automated MDCT software and indexed by body surface area (LAVi, LVDVi, RVDVi and LVMi). Linear regression models adjusted for demographic variables (model 1) and additionally for cardiac risk factors (model 2) were used to assess association between HIV and MDCT dimensions.

**Results: ** Most PLWH were men (88.2%) with mean (SD) age 53.7 (8.8) years, and had undetectable viral replication (96.1%) and CD4 count >500 cells/mL (77.0%). PLWH had smaller mean (SD) LAVi (40 mL/m^2^ (8) vs. 41 mL/m^2^ (9), *p* = 0.002) and LVDVi (61 mL/m^2^ (13) vs. 65 mL/m^2^ (14), *p* < 0.0001). However, RVDVi was larger in PLWH (89 mL/m^2^ (18) vs. 86 mL/m^2^ (17), *p* < 0.001) than uninfected controls. After adjustment for age, sex, body mass index, ancestry, smoking pack‐years, systolic blood pressure, high density lipoprotein, low density lipoprotein, triglyceride and non‐fasting blood glucose, HIV was associated with −6 mL (95% CI: −10; −3) lower LVDV, 11 mL (95% CI: 7;16) larger RVDV, and 4 g (95% CI: 1;7) larger LVM. Finally, CD4 nadir (per 100 cells/mL) was associated with 2 mL larger LVDV (95% CI: 0;3) and 3 mL larger RVDV (95% CI: 0;5).

**Conclusions: **HIV was independently associated with smaller LVDV, larger RVDV and greater LVM. However, absolute differences were small and no major structural cardiac abnormalities were found in a well‐treated population of PLWH. Thus, the clinical impact is uncertain, and it is unlikely that structural cardiac abnormalities explain the increased risk of CVD previously observed in PLWH

**Abstract THAB0102‐Table 1. Uni‐ and multivariate associations between HIV status and cardiac chamber volumes and left ventricular mass**

**Table:** 

CVD risk factors	Univariate β (95% CI)	*p*‐value	Model 1 β (95% CI)	*p*‐value	Model 2 β (95% CI)	*p*‐value
LAV (mL)						
HIV+/−	−5 (−7; −3)	<0.0001	−2 (−4; 1)	0.146	−1 (−3; 2)	0.618
LVDV (mL)						
HIV+/−	−12 (−15; −9)	<0.0001	−7 (−10; −3)	<0.0001	−6 (−10; −3)	<0.001
RVDL (mL)						
HIV+/−	1 (−3; 5)	0.605	9 (5; 13)	<0.0001	11 (7; 16)	<0.0001
LVM						
HIV+/−	−4 (−7; −1)	0.008	4 (2; 7)	0.002	4 (1; 7)	0.005

## THAB0103

### HIV infection independently increases the risk of developing heart failure: the HIV HEART study

**A.S. Go^1^; M. Horberg^2^; K. Reynolds^3^; W.J. Towner^3^; H. Avula^1^; R.C. Hechter^3^; D. Klein^1^; S. Vupputuri^2^; K.K. Lee^1^; T.K. Leong^1^; W. Leyden^1^; R. Neugebauer^1^; S.H. Sung^1^ and M.J. Silverberg^1^**

^1^Kaiser Permanente Northern California, Division of Research, Oakland, United States, ^2^Kaiser Permanente Mid‐Atlantic States, Mid‐Atlantic Permanente Research Institute, Rockville, United States, ^3^Kaiser Permanente Southern California, Department of Research and Evaluation, Pasadena, United States

**Background:** HIV infection has been associated with excess atherosclerotic events, but limited data exist about its link to developing heart failure (HF) and possible contributing factors. In a large, multi‐institutional, community‐based population, we evaluated the independent association of HIV infection with incident HF.

**Methods:** Within 3 large U.S. integrated healthcare delivery systems, we identified all eligible HIV(+) adults (≥21 years) between 2000 and 2016 without prior HF and frequency‐matched up to 10:1 to HIV(−) subjects without prior HF based on entry year, age (±1 year), gender, race, and primary treating facility. Through 2016, we identified cases of incident HF based on validated algorithms using electronic health records (EHR). Demographic features, cardiovascular risk factors, pertinent medical history and medication use were ascertained from EHR and other health system databases. We evaluated the independent association of HIV infection with incident HF through a series of multivariable Cox regression models that sequentially adjusted for: health system and calendar era, demographics, lifestyle factors, cardiovascular history, other comorbidities, and cardiopreventive and other medication use. In the final model, we adjusted for acute coronary syndrome events during follow‐up as a potential explanatory variable.

**Results: ** We identified 38,868 HIV(+) and 386,586 matched HIV(−) adults during the study period. HIV(+) patients were more likely to have low neighborhood‐level educational attainment and household income, prior cancer, dementia or depression but less likely to have prior cardiovascular conditions or cardiovascular risk factors. The rate (per 100 person‐years) of incident HF was higher in HIV(+) (0.24, 95% CI: 0.22 to 0.26) vs. matched HIV(−) (0.16, 95% CI: 0.15 to 0.16) patients (*p* < 0.0001) (Figure 1). In multivariable analyses, HIV infection was associated with an increased rate of developing HF that strengthened after serial adjustment for demographic characteristics; cardiovascular and medical history; and cardiopreventive medication, antidiabetic therapy and NSAID use, with an 75% increased rate in the fully adjusted model (Table, Models 1 to 3). Further adjustment for acute coronary syndrome events during follow‐up only modestly attenuated the association of HIV infection with incident HF (Model 4).

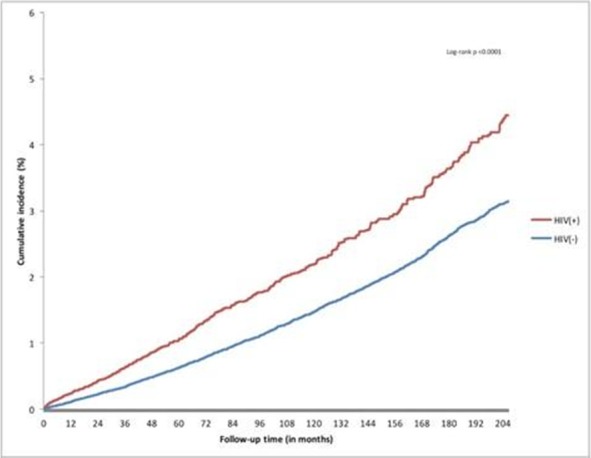

**Abstract THAB0103‐Figure 1. Cumulative incidence of newly‐diagnosed heart failure by HIV status.**

**Abstract THAB0103‐Table 1. Multivariable association of HIV infection with incident heart failure**

**Table:** 

	Model 1	Model 2	Model 3	Model 4
	Health System, Entry Year and Demographics	Model 1 + Cardiovascular and Non‐Cardiovascular History	Model 2 + Cardiopreventive Medications, Antidiabetic therapy and NSAIDs	Model 3 + Acute Coronary Syndrome Events During Follow‐Up
Adjusted Hazard Ratio (95% CI) for HIV(+) vs. HIV(−)	1.54 (1.40 to 1.69)	1.69 (1.54 to 1.86)	1.75 (1.59 to 1.93)	1.66 (1.50 to 1.83)

**Conclusions: **HIV infection independently increases the risk of developing HF and this excess risk does not appear to be mediated through atherosclerotic disease pathways or differential use of cardiopreventive therapies.

## THAB0104

### Malignancy and all‐cause mortality: incidence in teenage young adults living with perinatally acquired HIV

**S. Chhabra^1^; S. Fidler^1^; M. Bower^2^; S. Ayers^3^; H. Lyall^3^ and C. Foster^3^**

^1^Imperial College London, London, United Kingdom, ^2^Chelsea and Westminster Hospital NHS Trust, London, United Kingdom, ^3^Imperial College Healthcare NHS Trust, London, United Kingdom

**Background:** The incidence of malignancy between 10 and 24 years of age in the general UK population is 0.2/1000 person‐years. Adults living with HIV have an increased risk of malignancy yet there is a paucity of data for teenage young adults (TYA) living with perinatally acquired HIV (PaHIV).

**Methods:** Retrospective cohort analysis of all‐cause mortality and malignancies in TYAPaHIV aged 10 to 24 years attending a tertiary unit from 01.01.2004 to 31.12.2017, assessing mortality, cancer presentation, immunology, virology and comparing incidence and mortality to aged‐matched UK general population rates.

**Results: ** 290 TYAPaHIV contributed 2644 person‐years of follow up. 2/290 (0.7%) were lost to follow‐up, 14/290 (4.8%) transferred care and 6/290 (2.0%) died within the study period at a median age of 17 years (inter‐quartile range (IQR) 15 to 19). Cause of death; 3 with malignancy (non‐Hodgkin's lymphoma, hepatocellular carcinoma (HCC), gastrointestinal adenocarcinoma), 2 with end stage HIV with poor adherence to antiretroviral therapy (ART) and 1 with cryptococcal meningitis. Overall mortality rate was 2.3/1000 person‐years, 9.4 times the age‐matched general population (incidence rate ratio (IRR) 9.4, 95% confidence interval (CI) 3.4 to 20.4, *p* < 0.0001). 8/290 (2.8%) were diagnosed with a malignancy aged 10 to 24 years; 7/8 males; six with lymphoma (3 Hodgkin's, 1 Burkitt's, 2 B‐cell) and one each with HCC and gastrointestinal adenocarcinoma. At cancer diagnosis the median age was 19 years (IQR 14 to 23), median CD4 count 453 cells/uL (IQR 231 to 645) and 4/8 had undetectable HIV viral load (<50 copies/mL). Median length of HIV viraemia pre‐cancer diagnosis was 15 years (IQR 12 to 17). 4/6 lymphomas presented with advanced disease (Ann Arbor stage III/IV). The incidence of a malignancy was 3.0/1000 person‐years in TYAPaHIV, IRR to the age‐matched general population 12.9 (95% CI 5.6 to 25.5, *p* < 0.0001), driven by lymphomas (IRR 44.2, 95% CI 16.1 to 96.7, *p* < 0.0001).

**Conclusions: **In this cohort, TYA living with PaHIV had nearly a ten‐fold increased risk of all‐cause mortality and of malignancy compared to their uninfected peers, with the excess in malignancy driven by lymphomas. It is hoped that earlier access to antiretroviral therapy will mitigate some of the risk for future generations.

## THAB0105

### Increased risk of both mortality and incident comorbidity among frail HIV‐positive and HIV‐negative participants in the AGEhIV Cohort Study, and increased risk of frailty progression in those with HIV.

**E. Verheij^1,2^; G.D. Kirk^3^; F.W. Wit^1,2,4^; R.A. van Zoest^1,2^; S.O. Verboeket^1,2^; M.F. Schim van der Loeff^5^; P. Reiss^1,2,4^ and AGEhIV Cohort Study**

^1^Academic Medical Center, Department of Global Health and Division of Infectious Diseases¨, Amsterdam, Netherlands, ^2^Amsterdam Institute for Global Health and Development, Amsterdam, Netherlands, ^3^Johns Hopkins University, Department of Epidemiology, Bloomberg School of Public Health, Baltimore, United States, ^4^HIV Monitoring Foundation, Amsterdam, Netherlands, ^5^Public Health Service of Amsterdam, Department of Infectious Diseases, Amsterdam, Netherlands

**Background:** Frailty is associated with morbidity and mortality in the general geriatric population. We assessed both the impact of frailty on mortality/incident comorbidity risk and factors associated with frailty progression.

**Methods:** Longitudinal data from 598 people living with HIV (PLWH) and 550 demographically comparable HIV‐uninfected AGEhIV Cohort Study participants were analyzed (Table 1). The presence of ≥3 criteria among 5 domains (weight loss, low physical activity, exhaustion, decreased grip strength, and slow gait speed), defined frailty. Impact of time‐updated frailty status on all‐cause‐mortality in all, and on incident comorbidity among those (497 HIV‐positive and 479 HIV‐negative) contributing ≥1 consecutive visit pairs (n=1833 pairs) was assessed. Factors associated with frailty progression were analyzed among a subset of 488 participants with ≥2 consecutive biennial follow‐up visits (Table 1). Kaplan‐Meier plots, multivariable Cox or logistic regression models with generalized estimated equation were used, as appropriate.

**Results: ** Among HIV‐positive and HIV‐negative participants respectively, 8.5 and 3.4 percent were frail, and during 4,423 person‐years of follow‐up (PYFU) 12 and 5 persons died, for an all‐cause mortality rate of 5.2/1000 and 3.8/1000 PYFU. Time to death was shorter among frail persons. Adjusted for HIV‐status, age and number of pre‐existing comorbidities, frailty was independently associated with mortality (hazard ratio: 12.3, 95%CI 4.5 to 33). 342 incident comorbidities were diagnosed among 307 participants. Adjusted for HIV‐status, age, gender, ethnicity, education and number of pre‐existing comorbidities, frail participants had higher odds of developing ≥1 comorbidity (OR 1.93, 95%CI 1.15 to 3.322). No interaction was found between frailty and HIV‐status on mortality or incident comorbidity risk.

In the subset of 488 participants, 60 demonstrated frailty progression. HIV‐status was associated with frailty progression (OR 2.2, 95%CI 1.3 to 3.8), partly mediated by higher waist‐to‐hip ratio (WHR), comorbidity burden and depression.

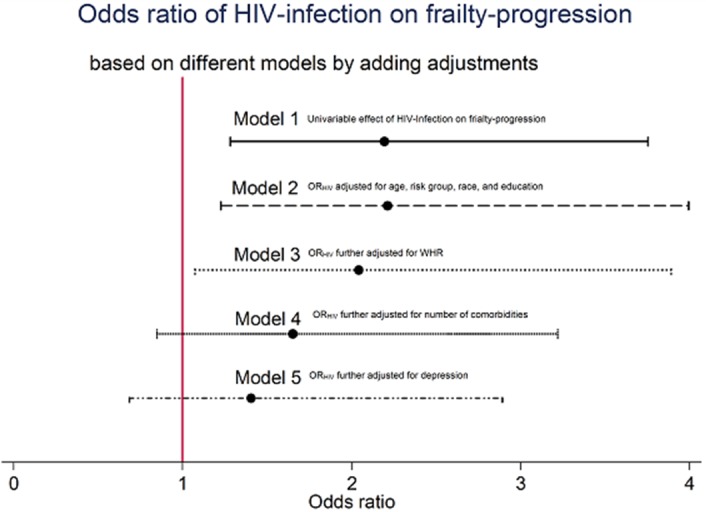

**Abstract THAB0105‐Figure 1. Showing the odds ratio and 95% CI of HIV‐infection on frailty‐progression based on different models.**

**Conclusions: **Frailty was a strong predictor of mortality and incident comorbidity, regardless of HIV‐status. Frailty was more prevalent among PLWH and HIV‐status was associated with frailty progression, which was partly mediated by higher WHR and pre‐existing comorbidity burden and depression. These results provide guidance to clinicians in recognizing patients at risk for developing frailty and associated adverse health outcomes, and the importance of maintaining physical and mental health.

**Abstract THAB0105‐Table 1. Baseline characteristics of participants of the AGEhIV Cohort**

**Table:** 

	Part A			Part B		
	HIV‐uninfected (n = 550)n (%) or median (IQR)	HIV‐infected (n = 598)n (%) or median (IQR)	p‐value	HIV‐uninfected (n = 284)n (%) or median (IQR)	HIV‐infected (n = 204)n (%) or median (IQR)	p‐value
Age, years	52.1 (47.9 to 58.1)	52.7 (48.3 to 59.4)	0.34	52.7 (48.2 to 57.9)	52.8 (48.2 to 59.2)	0.7
Risk group: MSM male, Non‐MSM male, Female	386 (70.2%), 79 (14.4%), 85 (15.5%)	454 (75.9%), 70 (11.7%), 74(12.4%)	0.09	203 (71.5%) 44 (15.5%) 37 (13.0%)	157 (77%) 28 (13.7%) 19 (9.3%)	0.3
Non‐black race	526 (95.8%)	522 (87.6%)	<0.001	274 (96.5%)	186 (91.2%)	0.013
Higher education	289 (55.8%)	220 (41.3%)	<0.001	166 (59.5%)	91 (46.0%)	0.003
WHR	0.91 (0.87 to 0.96)	0.97 (0.92 to 1.0)	<0.001	0.91 (0.88 to 0.96)	0.97 (0.92 to 1.00)	<0.001
Number of diagnosed comorbidities1	0 (0 to 1)	1 (0 to 1)	<0.001	0 (0 to 1)	1 (0 to 1)	<0.001
Depression; CES‐D ≥16^2^	71 (13.6%)	106 (19.8%)	0.006	21 (7.7%)	24 (12.5%)	0.08

Part A: Population analyzed for mortality and incident comorbidity. Part B: Population analyzed for frailty progression (284 HIV‐uninfected contributing to 442 visit‐pairs and 204 HIV‐infected contributing to 300 visit‐pairs) Abbreviations: WHR, waist‐to‐hip ratio; CES‐D, Center for Epidemiologic Studies Depression scale; Higher education; attained at least a bachelors degree. 1 Comorbidities; included are chronic obstructive pulmonary disease (defining obstruction as having lower than 1.64 z‐score for FEV1/FVC‐ratio using Global Lung Initiative guidelines), diabetes (HbA1c = 48 mmol/mol and/or elevated blood glucose (non‐fasting = 11.1 mmol/L or fasting = 7.0 mmol/L) or on antidiabetic medication), hypertension (use of antihypertensive medication or measured grade 2 hypertension following European Guidelines (systolic blood pressure 160 mmHg and/or diastolic blood pressure 100 mmHg in all 3 measurements, renal insufficiency (eGFR <60 mL/min/1.73 m2), osteoporosis (having a T score of −2.5 SD or lower, in men aged <50 years and premenopausal women; a Z score of −2 SD or lower in men aged =50 years and postmenopausal women), self‐reported and validated heart‐failure, non‐AIDS associated cancer, cardiovascular disease (myocardial infarction, angina pectoris, peripheral artery disease, ischemic cerebrovascular disease). 2 CES‐D scale, two questions used in the frailty scale are excluded from CES‐D score calculation.

## THAB0201

### Effectiveness of hepatitis A virus (HAV) vaccination among people living with HIV during an hepatitis A outbreak in Taiwan, 2015 to 2017

**K.‐Y. Lin^1,2^; H.‐Y. Sun^3^; S.‐M. Hsieh^3^; H.‐Y. Cheng^4^; Y.‐C. Lo^4^; W.‐H. Sheng^3^; Y.‐C. Chuang^3^; A. Cheng^3^; S.‐C. Pan^3^; G.‐J. Chen^3^; C.‐T. Fang^2^; C.‐C. Hung^3^ and S.‐C. Chang^3^**

^1^National Taiwan University Hospital Jin‐Shan Branch, Department of Medicine, New Taipei City, Taiwan, Province of China, ^2^Institute of Epidemiology and Preventive Medicine, College of Public Health, National Taiwan University, Taipei, Taiwan, Province of China, ^3^National Taiwan University Hospital and National Taiwan University College of Medicine, Department of Internal Medicine, Taipei, Taiwan, Province of China, ^4^Centers for Disease Control, Taipei, Taiwan, Province of China

**Background:** Outbreaks of hepatitis A virus (HAV) infection have reemerged among men who have sex with men (MSM) across the Asia‐Pacific region, the United States, and several European countries since 2015. The suboptimal response to HAV vaccine among MSM living with HIV raises serious concerns about the personal‐level and population‐level effectiveness of HAV vaccination. We estimated the transmissibility of HAV during an hepatitis A outbreak among MSM living with HIV in Taiwan, 2015 to 2017, and measured the effectiveness of HAV vaccine in this population

**Methods:** We developed a mathematical model of HAV transmission to estimate the basic reproductive number (*R*0) of HAV in this outbreak. A case of acute hepatitis A was defined as a documented positive anti‐HAV IgM in an HIV‐positive patient who presented with clinical symptoms, elevated aminotransferases, or jaundice. We conducted a 1:4 nested case‐control study to assess the effectiveness of HAV vaccine in MSM living with HIV.

**Results: ** Given 30% of HIV‐positive patients having mild symptoms after acquiring acute hepatitis A, we estimated the *R*0 of HAV was as high as 6.37 in this outbreak. During study period (from 1 June 2015 to 30 June 2017), 55 cases of acute hepatitis A occurred among 1533 initially HAV seronegative HIV‐positive patients. All case patients were MSM with a median age of 30 years and baseline CD4 count of 545 cells/μL, and 60% had recent syphilis within six months prior to the onset of acute hepatitis A. HAV vaccination protected recipients from acute hepatitis A (adjusted odds ratio, 0.03; 95% CI, 0.001 to 0.12), with an overall vaccine effectiveness of 97.4% (Table). The effectiveness of single dose and two doses of HAV vaccine was 96.1% and 99.7%, respectively. This high personal‐level vaccine effectiveness in MSM living with HIV might explain the rapid control of this hepatitis A outbreak in Taiwan Conclusions

Our findings strongly support the implementation of HAV vaccination to control hepatitis A outbreak among MSM living with HIV.

**Abstract THAB0201‐Table 1. Factors associated with acquiring acute hepatitis A**

**Table:** 

	Univariable analysis		Multivariable analysis	
	Odds ratio (95% CI)	*p* value	Odds ratio (95% CI)	*p* value
At least one dose of HAV vaccination	0.04 (0.01 to 0.13)	<0.001	0.03 (0.001 to 0.12)	<0.001
Age, per 1‐year increase	1.53 (0.63 to 3.70)	0.349		
HBsAg positivity	1.28 (0.27 to 4.98)	0.913		
Anti‐HCV positivity	2.03 (0.58 to 6.57)	0.302		
Receiving cART at baseline	1.19 (0.52 to 2.70)	0.685		
CD4 count at baseline, per 1‐cell/mm^3^ increase	0.999 (0.998 to 1.001)	0.316		
PVL at baseline, per 1‐log10 copies/mL increase	1.03 (0.88 to 1.21)	0.710		
Syphilis during follow‐up	3.93 (2.04 to 7.60)	<0.001	13.52 (1.40 to 130.26)	0.024

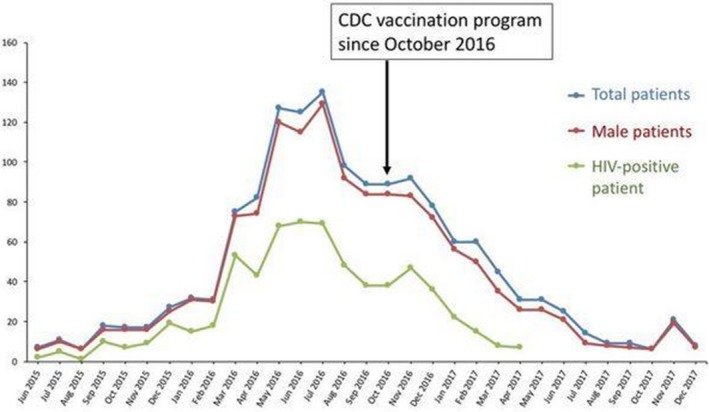

**Abstract THAB0201‐Figure 1. Number of indigenous cases of acute hepatitis A reported to the Taiwan CDC during the outbreak.**

## THAB0202

### Less severe but prolonged course of acute hepatitis A in HIV‐positive patients than HIV‐negative patients during an outbreak: a multicenter observational study

**Y.‐L. Li^1^; C.‐E. Liu^1^; C.‐C. Hung^2^; P.‐L. Lu^3^; N.‐Y. Chen^4^ and Taiwan HIV Study Group**

^1^Changhua Christian Hospital, Changhua, Taiwan, Province of China, ^2^National Taiwan University Hospital, Taipei, Taiwan, Province of China, ^3^Kaohsiung Medical University, Kaohsiung, Taiwan, Province of China, ^4^Chang Gung Memorial Hospital, Taoyuan, Taiwan, Province of China

**Background:** This multicenter retrospective cohort study aimed to compare the clinical presentations and evolution of acute hepatitis A (AHA) between HIV‐positive patients and HIV‐negative counterparts during the AHA outbreak.

**Methods:** Information on the demographics, clinical presentations, serial laboratory data, and abdominal imaging were collected from the medical records of the patients who received a diagnosis of AHA at the 14 designated hospitals for HIV care around Taiwan between May 2015 and May 2017.

**Results: ** A total of 297 adult patients with AHA were included during the 2‐year study period. With a mean age of 31.4 years (range, 19.0 to 76.1), 93.4% were males and 58.6% MSM. Of 265 patients with known HIV serostatus, 166 (62.6%) were HIV‐positive. Compared with HIV‐negative patients, HIV‐positive patients had a lower peak alanine aminotransferase (ALT) level (median, 1312 vs. 2014 IU/L, *p* = 0.003), less coagulopathy (6.0% vs. 16.2%, *p* = 0.007), less hepatomegaly or splenomegaly on imaging studies, but delayed resolution of hepatitis (40.9% vs. 21.3%, *p* = 0.005). In the subgroup analysis, HIV‐positive patients with good HIV viral suppression (plasma RNA load (PVL) <1000 copies/mL) by combination antiretroviral therapy (cART) had a higher peak ALT level (median, 1420 vs. 983 IU/L, *p* = 0.012) and less delay in resolution of hepatitis (22.6% vs. 51.0%, *p* < 0.001) than patients without cART or with higher PVL.

**Conclusions: **During an AHA outbreak, we found that HIV‐positive patients had a lower severity, but delayed resolution of AHA than HIV‐negative patients. Receipt of cART with better viral suppression alleviated the impact of HIV infection on the clinical manifestations of AHA in HIV‐positive patients.

## THAB0203

### Shared HCV transmission networks among HIV‐1 positive and HIV‐1 negative men having sex with men in Paris

**T. Nguyen^1^; C. Delaugerre^2,3^; M.‐A. Valatin^4^; E. Netzer^5^; P.‐M. Girard^6^; N. Day^7^; G. Kreplak^7^; G. Pialoux^8^; J.‐M. Molina^3,9^; V. Calvez^1^; A.‐G. Marcelin^1^ and E. Todesco^1^**

^1^Sorbonne Université, INSERM, Institut Pierre Louis d'Epidémiologie et de Santé Publique ^iPLESP^, AP‐HP, Hôpital Pitié‐Salpêtrière, Laboratoire de Virologie, Paris, France, ^2^Saint‐Louis, Assistance Publique Hôpitaux de Paris, Paris, France, ^3^INSERM UMR 941, Université de Paris Diderot, Sorbonne Paris Cité, Paris, France, ^4^Sorbonne Université, INSERM, Institut Pierre Louis d'Epidémiologie et de Santé Publique ^iPLESP^, AP‐HP, Hôpital Pitié‐Salpêtrière, Services de Maladies Infectieuses et Tropicales, Paris, France, ^5^INSERM SC10, Villejuif, France, ^6^Saint‐Antoine Hospital, Assistance Publique‐Hôpitaux de Paris, Paris, France, ^7^Cerballiance Laboratory, Paris, France, ^8^Hôpital Tenon, APHP, Université Pierre et Marie Curie, Department of Infectious Diseases, Paris, France, ^9^Maladies Infectieuse, Hôpital Saint‐Louis, Assistance Publique Hôpitaux de Paris, Paris, France

**Background:** Infection of acute hepatitis C (AHC) among men having sex with men (MSM) has become an outbreak in several high‐income countries from 2000. Several studies reported existence of specific HCV transmission network among MSM communities in Europe and especially a spread of HCV strains from HIV‐HCV co‐infected MSM toward HCV mono‐infected MSM. We aimed to characterize HCV transmission clusters in HIV positive and HIV negative MSM communities in Parisian region by ultra‐deep sequencing (UDS).

**Methods:** Illumina (Miseq) deep‐sequencing of NS5B fragment was performed on plasma samples of 50 AHC HIV‐positive and 18 AHC HIV‐negative individuals including 13 from the Prep IPERGAY ANRS study. UDS data were analysed by Geneious (version 10.1.3). Phylogenetic trees were realized using Fasttree 2.1 and local support value of >80% was chosen to define a robust tree. Trees were submitted to ClusterPicker (version 1.2.3) to determine transmission cluster at different thresholds of maximum genetic distance (MGD). We compared results of Sanger at 3%, UDS at 3%, and at 4.5% of MGD.

**Results: ** Of 68 acute hepatitis C patients, 15 were cases of recontamination. HCV genotyping showed genotype 1a (47%), 4d (41%), 3a (9%), and 2k (3%). Sanger at 3%, UDS at 3% and at 4.5% of MGD allowed detection of 10, 17, and 18 clusters, respectively. By UDS, more clusters were detected but fewer subjects (median: 2 subjects) were identified within each cluster than Sanger did (median: 3 subjects). Furthermore, mixed clusters including HIV‐positive and HIV‐negative MSM were observed in 8/10 clusters by Sanger, in 10/17 by UDS at 3%, and in 10/18 by UDS at 4.5% of MGD. Overall, the number of HIV‐negative individuals clustering with HIV‐positive ones was 8/18 by Sanger, 8/18 by UDS at 3%, and 9/18 by UDS at 4.5% of MGD.

**Conclusions: **By Sanger or UDS, our study allowed the detection of HCV transmission clusters in MSM communities in Parisian region. Particularly in this population, the HIV‐positive MSM shared the HCV transmission network with HIV‐negative MSM, which in turn alerts the public health for surveillance and prevention measures in these communities, regardless of their HIV status.

## THAB0204

### Will hepatitis C transmission be eliminated by 2025 among HIV‐positive men who have sex with men in Australia?

**L. Salazar‐Vizcaya^1^; D.C. Boettiger^2^; G.J. Dore^2^; R. Gray^2^; M. Law^2^; A. Rauch^1^; T. Lea^3^ and G. Matthews^2^**

^1^University Hospital Bern, Inselspital, Department of infectious diseases, Bern, Switzerland, ^2^Kirby Institute, UNSW, Sydney, Australia, ^3^Centre for Social Research in Health, UNSW, Sydney, Australia

**Background:** Australia was among the first countries to offer universal access to direct‐acting antiviral (DAA) therapy for hepatitis C virus (HCV) to HIV‐positive men who have sex with men (MSM). Rapid scale‐up of DAA therapy has been ongoing in Australia since 2016 and has the potential to interrupt HCV transmissions. However, concerns have been raised that behavioural changes may counterbalance the effect of treatment. We assessed the potential effect of treatment and risk behavioral changes on HCV incidence among HIV‐positive MSM up to 2025.

**Methods:** Mathematical model of HCV transmission parameterized with Australian data collected between 2000 and 2016. The model was set to reproduce observed data and to assess the future impact of a range of changes in behavior that facilitate HCV transmission (i.e. high‐risk sex and injecting drug use‐IDU) in the context of increasing and decreasing rates of DAA use. Baseline DAA‐treatment rate was based on data from the Control and Elimination within Australia of Hepatitis C from people living with HIV (CEASE) study.

**Results: ** The Figure 1 summarizes model outcomes. If the rate of DAA use increased from 65%/year among eligible patients in 2016 to 100%/year by 2025, HCV incidence would drop from 3.7/100 person‐years (py) in 2016 to 0.4/100py by 2025 providing rates of risk behavior remained at current levels (20% and 45% for IDU and high‐risk sex respectively). Even in the setting of substantial increases in the rates of high‐risk sex and IDU (e.g. 90% and 85%) HCV incidence would drop to 0.8/100py. Moreover, if treatment rate remains stable after 2016, incidence would also drop regardless of risk behavior (results not shown in the Figure 1). Conversely, HCV incidence only increased from that in 2016 when rates of IDU also increased substantially (above 69%) alongside a drop in DAA use (to 20% by 2025).

**Conclusions: **The model suggests that HCV transmission among Australia's HIV‐positive MSM population will continue, but decline substantially with DAA treatment upscale, even in the context of increased risk behaviour. If treatment rate continues to increase, by 2025 Australia could meet the 80% reduction goal formulated by the WHO.

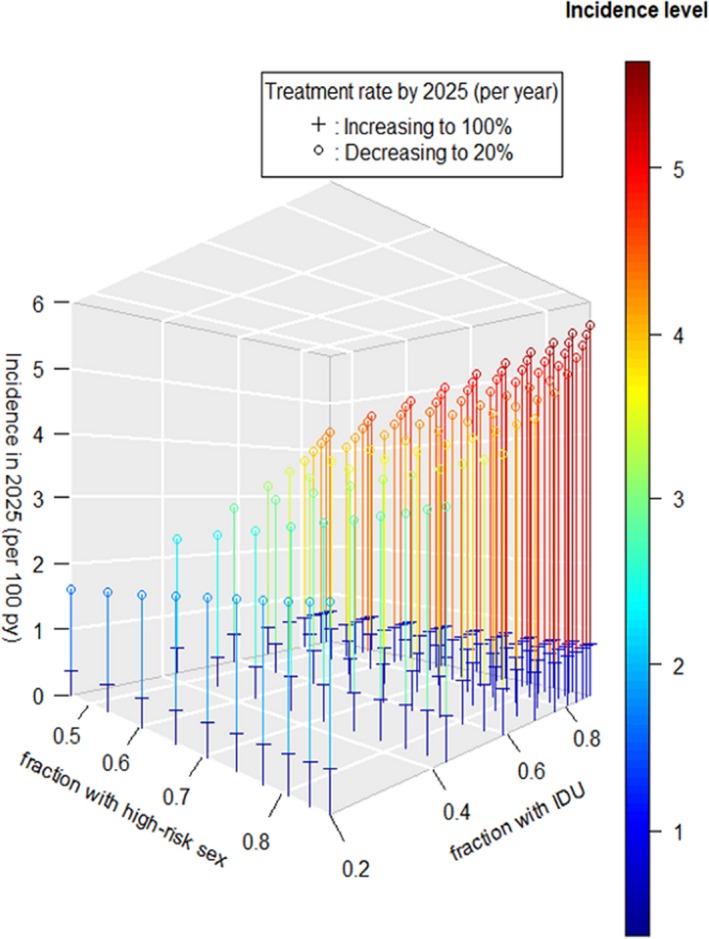

**Abstract THAB0204‐Figure 1. Projected incidence of HCV among HIV‐positive men who have sex with men in Australia. Colors indicate incidence values as depicted in the colorued bar.**

## THAB0205

### HIV infection is an independent risk factor for liver steatosis: a study in HIV mono‐infected patients compared to uninfected paired controls and associated risk factors

**A. Pacheco^1^; H. Perazzo^2^; S. Cardoso^2^; M.J. Fonseca^3^; R. Griep^4^; P. Lotufo^5^; I. Bensenor^5^; J. Mill^6^; R. Moreira^1,2^; R. Moreira^2^; R. Friedman^2^; M. Santini‐Oliveira^2^; V. Veloso^2^; D. Chor^3^ and B. Grinsztejn^2^**

^1^Fundação Oswaldo Cruz (FIOCRUZ), PROCC, Rio de Janeiro, Brazil, ^2^Fundação Oswaldo Cruz (FIOCRUZ), LAPCLIN‐AIDS, Rio de Janeiro, Brazil, ^3^Fundação Oswaldo Cruz (FIOCRUZ), Departamento de Epidemiologia e Métodos Quantitativos em Saúde, Rio de Janeiro, Brazil, ^4^Fundação Oswaldo Cruz (FIOCRUZ), Laboratory of Health and Environment Education, Rio de Janeiro, Brazil, ^5^University of São Paulo, School of Medicine, São Paulo, Brazil, ^6^Federal University of Espírito Santo, Departamento de Ciências Fisiológicas, Vitoria, Brazil

**Background:** Several studies have been reporting the burden of metabolic liver disease in HIV‐infected individuals. However, the impact of HIV infection on prevalence of liver steatosis and associated risk factors still lack. We aimed to evaluate the prevalence and factors associated with liver steatosis in HIV mono‐infected patients compared to uninfected subjects paired for confounding factors.

**Methods:** 649 HIV mono‐infected patients from the INI‐ELSA cohort were eligible. To test the association of HIV infection with steatosis, non‐infected subjects from the Brazilian Longitudinal Study of Adult Health (ELSA‐Brasil) cohort (n = 15,087) were paired by demographic, metabolic and inflammatory characteristics. Nearest neighbor matching with a 0.05 caliper on logistic regression‐based scores were used for matching and balance was checked with usual procedures. The variables used for the matching and the ones used in the final model for risk factors for steatosis in HIV‐infected individuals were selected through a genetic algorithm that searched for the best model fit. Fatty Liver Index (FLI) was calculated as previously validated (Bedogni BMC‐Gastroenterology 2006) and liver steatosis was defined as FLI ≥ 60. Logistic regression analysis was performed to assess risk of steatosis in HIV‐patients compared to controls and to identify factors associated with fatty liver in HIV‐infected individuals.

**Results: ** HIV‐patients (58% male; median (IQR) age = 44 (36 to 51) years; body mass index (BMI) = 24.4 (21.9 to 27.5) kg/m^2^; 33% with metabolic syndrome) were paired with 333 uninfected controls, with good balance on demographic and clinical characteristics between patients and controls. HIV infection was an independent factor associated with liver steatosis (OR = 2.1 (95% CI 1.49 to 2.95), *p* < 0.001). In a multivariate analysis with all HIV patients included, the following variables were independently associated (OR (95% CI)) with presence of steatosis: male gender (5.36 (2.41 to 11.94)); Black/*Pardo* ethnicity (0.22 (0.09 to 0.55)); hypertension (2.56 (1.25 to 5.26)); diabetes (5.79 (2.58 to 13)); dyslipidemia (2.57 (1.27 to 5.21)); BMI (1.91 (1.67 to 2.18)); CD4 count (per cell/mm^3^, 1.13 (1.01 to 1.27)) and cumulative HIV viral load (1.25 (1.02 to 1.54)).

**Conclusions: **HIV‐infected individuals had two‐fold higher odds for presence of steatosis compared to uninfected paired controls. Traditional and HIV‐specific risk factors were also associated with steatosis. Prevention of metabolic factors should be integrated to HIV care to decrease the burden of liver diseases in HIV‐infected individuals.

**Abstract THAB0205‐Table 1. Risk factors associated with liver steatosis in HIV mono‐infected patients**

**Table:** 

HIV mono‐infected (n = 649)	OR (95% CI)	*p*‐value
Male gender (yes vs. no)	5.36 (2.41 to 11.94)	< 0.001
Black/Pardo ethnicity (yes vs. no)	0.22 (0.09 to 0.55)	0.001
Hypertension (yes vs. no)	2.56 (1.25 to 5.26)	0.010
Type 2 diabetes (yes vs. no)	5.79 (2.58 to 13)	< 0.001
Dyslipidemia (yes vs. no)	2.57 (1.27 to 5.21)	0.009
BMI (kg/m2)	1.91 (1.67 to 2.18)	< 0.001
Poor health management (yes vs. no)	0.36 (0.17 to 0.79)	0.011
CD4 count (per 100 cells/mm^3^)	1.13 (1.01 to 1.27)	0.036
Cumulative HIV viral load (per 10 log(copies/mm^3^)*year)	1.25 (1.02 to 1.54)	0.031

## THAB0301

### High frequency of unintended pregnancy and predictors of contraceptive choice among HIV‐infected African women on life‐long antiretroviral therapy (ART). The US‐PEPFAR PROMOTE Cohort Study

**J. Aizire^1^; N. Yende^2^; T. Nematadzira^3^; M.E. Nyati^4^; S. Dadabhai^1^; L. Chinula^5^; C. Nakaye^6^; M. Naidoo^7^; M.G. Fowler^8^; T. Taha^1^ and US‐PEPFAR PROMOTE Cohort Study**

^1^Johns Hopkins Bloomberg School of Public Health, Epidemiology, Baltimore, United States, ^2^Centre for the AIDS Programme of Research in South Africa (CAPRISA), Data Management Centre (DMC), Durban, South Africa, ^3^University of Zimbabwe College of Health Sciences, Harare, Zimbabwe, ^4^Perinatal HIV Research Unit (PHRU), Chris Hani Baragwanath Hospital University of the Witwatersrand, Johannesburg, South Africa, ^5^University of North Carolina – Lilongwe, Lilongwe, Malawi, ^6^Makerere University Johns Hopkins University (MUJHU), Kampala, Uganda, ^7^Centre for The AIDS Research in South Africa (CAPRISA), Umlazi Clinical Research Site, Nelson R. Mandela School of Medicine, Durban, South Africa, ^8^Johns Hopkins University, School of Medicine, Pathology‐Medical Microbiology, Baltimore, United States

**Background:** About 90% of unintended pregnancies among African women are attributed to non‐use of effective family planning (EFP) methods (injectable, oral, intra‐uterine device (IUD), implant, or tubal‐ligation). Long acting reversible contraceptives (LARC), which include implants or IUDs, are the most effective reversible contraception for an extended period without requiring user action. We report frequency of unintended pregnancy and determinants of contraceptive choice among African women on life‐long ART.

**Methods:** The US‐PEPFAR PROMOTE is a long‐term cohort study of HIV‐infected women (n = 1986) and their children, enrolled from December 2016 to June 2017, as an extension to a completed mother‐to‐child transmission prevention trial from eight sites in Malawi, South Africa, Uganda, and Zimbabwe. Standardized questionnaires were used to collect demographic and reproductive health data. Baseline enrollment data were analyzed using chi square and Wilcoxon Rank‐Sum tests for group comparisons, and multivariable logistic regression to identify determinants of contraceptive choice.

**Results: ** Overall, among 1984 women included in this analysis, 49.9% (n = 990) reported that their last pregnancy was unintended (Figure 1), and >50% had no desire for more children. Among sexually‐active, non‐pregnant women, 81.6% (1,050/1,287) reported EFP use; while 19.0% (227/1,197) without permanent contraception reported LARC methods. Injectables were the commonest method (39%) – especially at the South African sites (>50%), followed by implant (14.4%). Oral pills were popular in Zimbabwe and tubal‐ligations were common in Malawi and South Africa. Non‐pregnant women whose last pregnancy was unintended versus intended were more likely to report current EFP use, adjusted odds ratio (95% CI), 1.44 (1.10 to 1.96), *p* = 0.02; but not LARC use, 1.25 (0.92 to 1.70), *p* = 0.15. Women with no formal employment were less likely to report LARC use, 0.64 (0.43 to 0.96), *p* = 0.03, but not EFP‐use, 0.92 (0.62 to 1.35), *p* = 0.60. All multivariable models included age, marital‐status, attained desired number of children, clinic travel‐time, household water, and electricity, which were not associated with contraception choice.

**Conclusions: **Unintended pregnancy is common among HIV‐infected African women. LARCs are acceptable contraceptive options in these settings, though under‐utilized. Programmatic research should explore integrated ART and LARC delivery in consideration of the unique reproductive health challenges among HIV‐infected African women on universal ART.

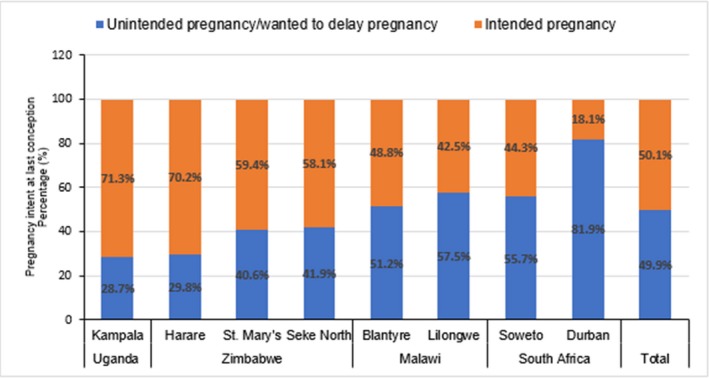

**Abstract THAB0301‐Figure 1. Fertility desire at last conception among HIV‐infected women on Antiretroviral Therapy (ART) reported at study entry.**

## THAB0302

### Tenofovir alafenamide pharmacokinetics with and without cobicistat in pregnancy

**J.D. Momper^1^; B. Best^1^; J. Wang^2^; A. Stek^3^; T.R. Cressey^4^; S. Burchett^5^; R. Kreitchmann^6^; D.E. Shapiro^2^; E. Smith^7^; N. Chakhtoura^8^; E.V. Capparelli^1^; M. Mirochnick^9^ and IMPAACT P1026s Protocol Team**

^1^University of California, San Diego, La Jolla, United States, ^2^Harvard University, Boston, United States, ^3^University of Southern California, Los Angeles, United States, ^4^Chiang Mai University, Chiang Mai, Thailand, ^5^Boston Children's Hospital, Boston, United States, ^6^Irmandade da Santa Casa de Misericordia de Porto Alegre, Porto Alegre, Brazil, ^7^National Institute of Allergy and Infectious Diseases, Bethesda, United States, ^8^National Institute of Child Health and Human Development, Bethesda, United States, ^9^Boston University, Boston, United States

**Background:** Tenofovir alafenamide (TAF), a prodrug of tenofovir (TFV), has enhanced stability in plasma and improved safety compared to tenofovir disoproxil fumarate (TDF). TAF is administered as part of a fixed‐dose combination either with or without the pharmacokinetic (PK) booster cobicistat (COBI). As COBI inhibits transporters involved in the disposition of TAF, co‐administration of TAF with COBI results in higher TAF exposure. The PK of TAF have not been studied in pregnant women. This study described TAF exposure when administered with and without COBI during pregnancy and postpartum.

**Methods:** IMPAACT P1026s is an ongoing, nonrandomized, open‐label, multi‐center study of antiretroviral PK in HIV‐infected pregnant women. Steady‐state PK profiles of TAF following once‐daily dosing of either TAF/FTC/RPV (25/200/25 mg, Odefsey^®^) or TAF/FTC/EVG/COBI (10/200/150/150 mg, Genvoya^®^) were obtained during the 2nd and 3rd trimesters (2T/3T) and 6 to 12 weeks postpartum (PP). TAF plasma concentrations were measured by a validated LC‐MS/MS method. A two‐tailed Wilcoxon signed rank test (α = 0.10) was employed for paired within‐subject comparison of PK parameters.

**Results: ** A total of 42 subjects from the US were enrolled with a median age at delivery of 30.4 years (range 19.1 to 38.8). For the TAF 25 mg arm, TAF exposure was lower and oral clearance was higher in 3T compared to PP (Table 1). For the TAF/COBI arm, no differences were seen between any ante‐partum and post‐partum PK parameters. HIV RNA at delivery was <50 copies/mL for 10/11 women in the TAF 25 mg arm and 24/27 women in the TAF/COBI arm. Median infant gestational age and weight at birth were 38.9 weeks and 3.24 kg, respectively. Congenital anomalies considered possibly related to study drugs included a ventral septal defect in one infant and congenital pseudoarthrosis of the left clavicle and neonatal compartment syndrome in another infant. Overall 27/41 infants were HIV‐negative and 14 were indeterminate/pending.

**Conclusions: **In women taking TAF without COBI, TAF exposure was lower in 3T compared to PP, whereas no differences were seen between pregnancy and PP in women taking TAF with COBI. Before TAF can be recommended for use in pregnancy additional safety and outcome data as well as intracellular PK data are needed.

**Abstract THAB0302‐Table 1. Tenofovir Alafenamide Pharmacokinetic Parameters, Median (IQR)**

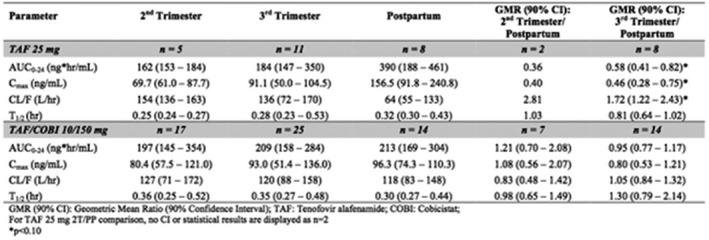

## THAB0303

### Effect of pregnancy on raltegravir free concentrations

**Y. Zheng^1^; D. Hirt^1^; S. Benaboud^1^; J. Lechedanec^2^; J.‐M. Tréluyer^1^; S. Delmas^2^; E. Arezes^2^; J. Warszawski^2^; J. Ghosn^3^ and ANRS 160 RalFe Study Group**

^1^Hopital Cochin Laboratoire Pharmacologie, Paris, France, ^2^Hôpital Kremlin‐Bicêtre, Paris, France, ^3^Hôpital Hôtel‐Dieu, Infectious Diseases, Paris, France

**Background:** Raltegravir can be used for the prevention of mother‐to‐child HIV transmission, especially when a rapid decline of HIV RNA load is necessary. Physiological changes during pregnancy have an impact on raltegravir elimination. Indeed, exposure of total raltegravir were shown to decrease from 29% to 50% during the third trimester of pregnancy compared to postpartum. However albumin level is known to be lowered during pregnancy which could increase the active free fraction of the drug and reduce this effect. The objective of this study was to describe the unbound, total and glucuronide raltegravir pharmacokinetics during pregnancy.

**Methods:** The RalFe ANRS160 study was a non‐randomized, open label, multicenter phase II trial in HIV‐infected pregnant women receiving raltegravir 400 mg twice daily. Samples were collected during pregnancy (between 30 and 37 weeks of amenhorrea), at delivery and at postpartum (four to six weeks after delivery). None of these women received an antiretroviral drug which could interact with raltegravir. Free raltegravir, total raltegravir and raltegravir glucuronide concentrations were measured using a validated liquid chromatography‐tandem mass spectrometry and ultrafiltration methods. A population pharmacokinetic model was developed by using NONMEM software.

**Results: ** A total of 414 samples were collected from 43 women, in which free, total and glucuronide raltegravir concentrations were measured. Free raltegravir was described by a one‐compartment model with first order absorption and lag time, evolving either to bound raltegravir (by a linear binding to albumin), or metabolism to raltegravir glucuronide (through an additional compartment) or to a first order elimination. Pregnancy increased free raltegravir clearances : by 26% for glucuronide formation and 17% for other elimination. During pregnancy, trough concentrations and exposures decreased by 28% and 37% for total raltegravir and by 25% and 22% for free raltegravir. The decrease was low for the glucuronide form.

**Conclusions: **This is the first data reporting raltegravir free and glucuronide pharmacokinetics during pregnancy. Pregnancy effect is moderate on the active raltegravir free fraction, especially when compared to its intersubject variability. This effect is not considered to be of clinical importance; raltegravir doses do not need to be modified during pregnancy.

## THAB0304

### Lack of viral load testing in pregnancy is a barrier to breastfeeding among HIV‐infected women in Botswana

**G.K. Mayondi^1^; R. Zash^1,2,3^; S. Moyo^1,4^; M. Diseko^1^; J. Mabuta^1^; G. Mogomotsi^5^; E. Dintwa^5^; J. Makhema^1^; R. Falama^6^; M. Mmalane^1^; S. Lockman^1,2,7^; M. Essex^1,2^ and R. Shapiro^1,2^**

^1^Botswana Harvard AIDS Institute Partnership, Gaborone, Botswana, ^2^Harvard T.H. Chan School of Public Health, Boston, United States, ^3^Beth Israel Deaconess Medical Center, Boston, United States, ^4^Harvard T.H. Chan School of Public Health, Department of Immunology and Infectious Diseases, Boston, United States, ^5^Ministry of Health and Wellness, Gaborone, Botswana, ^6^Kanye Seventh Adventist Hospital, Kanye, Botswana, ^7^Brigham and Women's Hospital, Boston, United States

**Background:** Breastfeeding reduces child morbidity and mortality, including among HIV‐infected women. Botswana updated its antiretroviral treatment (ART) guidelines in May 2016 to support breastfeeding for HIV‐infected women on ART who have documented viral suppression during pregnancy. We describe infant feeding choices among HIV‐infected mothers since new guidelines were implemented.

**Methods:** We abstracted data from obstetric records of all HIV‐infected women discharged with a live infant from eight government maternity wards from September 2016 to December 2017. We validated the reported feeding method using the PMTCT counselor report, formula dispensing log or direct observation. We evaluated obstetric card documentation of any viral load in pregnancy. All pregnant women were eligible to start ART and continue life‐long, regardless of CD4 cell count.

**Results: ** Among 6845 HIV‐infected women with a live infant, 6473 had a validated feeding method at discharge from maternity and were included in the analysis. ART coverage was high (95%) among all women: 58% started ART prior to conception, 37% started in pregnancy, and 96% with a known ART start date had received at least eight weeks of ART prior to delivery. At discharge, 2553 (39%) of the HIV‐infected women were confirmed to be breastfeeding (Table 1). Of 4573 records assessed, only 412 (9%) women had viral load result (VL) documented at any time during pregnancy; of these, 400 (97%) were suppressed to <400 copies/mL. Among women with documented suppressed VL during pregnancy, 228 (57%) chose breastfeeding compared with 1519 (37%) of women without documented viral load testing in pregnancy (*p* < 0.0001). Four (33%) of 12 women with VL>400 copies/mL and 72 (27%) of 270 women with no reported ART use in pregnancy, chose to breastfeed.

**Conclusions: **The low rate of breastfeeding among HIV‐infected women in Botswana underscores the need to address barriers affecting feeding choices. Although the majority of pregnant women tested have a suppressed viral load and might be eligible for breastfeeding per Botswana guidelines, few women currently receive viral load testing in pregnancy. The viral load testing requirement in HIV‐infected pregnant women is therefore unlikely to prevent a substantial amount of HIV transmission during breastfeeding, but likely contributes to low rates of breastfeeding in Botswana.

**Abstract THAB0304‐Table 1. Characteristics of HIV+ Women in Botswana, by Infant Feeding Choice at Discharge from Maternity Ward)**

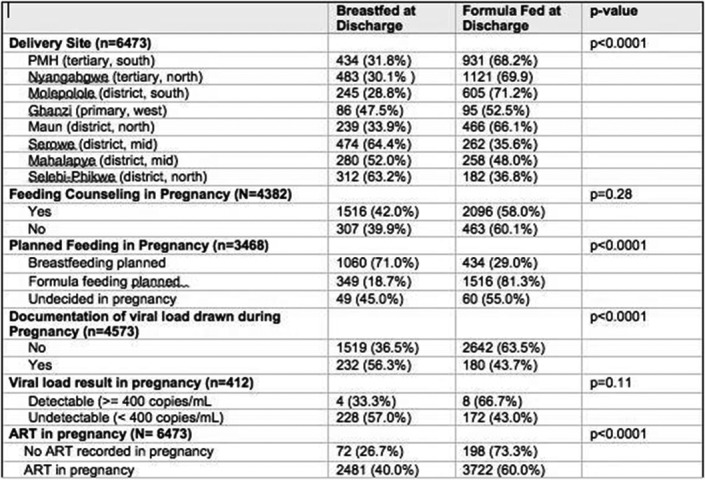

## THAB0305

### Safety and nevirapine concentrations of 6‐week triple antiretroviral prophylaxis in high risk HIV‐exposed tnfants

**S. Anugulruengkitt^1,2^; P. Suntarattiwong^3^; P. Ounchanum^4^; U. Srirompotong^5^; W. Jantarabenjakul^1,2^; J. Sophonphan^6^; S. Punnahitanon^1^; C. Pancharoen^1,2^; K. Chokephaibulkit^7^; T.R. Cressey^8,9,10^; T. Puthanakit^1,2^ and on behalf of CIPHER_AEPEP Study Team**

^1^Chulalongkorn University, Department of Pediatrics, Faculty of Medicine, Bangkok, Thailand, ^2^Chulalongkorn University, Center of Excellence in Pediatric Infectious Diseases and Vaccines, Faculty of Medicine, Bangkok, Thailand, ^3^Queen Sirikit National Institute of Child Health, Department of Pediatrics, Faculty of Medicine, Bangkok, Thailand, ^4^Chiang Rai Prachanukroh Hospital, Chiang Rai, Thailand, ^5^Khon Kaen Hospital, Khon Kaen, Thailand, ^6^The Thai Red Cross AIDS Research Centre, HIV Netherlands Australia Thailand Research Collaboration ^HIV‐NAT^, Bangkok, Thailand, ^7^Siriraj Hospital, Mahidol University, Department of Pediatrics, Faculty of Medicine, Bangkok, Thailand, ^8^Chiang Mai University, Program for HIV Prevention and Treatment, Department of Medical Technology, Faculty of Associated Medical Sciences ^IRD/174^, Chiang Mai, Thailand, ^9^Harvard T.H. Chan School of Public Health, Boston, United States, ^10^University of Liverpool, Department of Molecular & Clinical Pharmacology, Liverpool, United Kingdom

**Background:** Triple‐drug antiretroviral prophylaxis of zidovudine(AZT)/lamivudine(3TC)/nevirapine(NVP) is preferred among HIV‐exposed neonates with high‐risk of transmission in many countries, including Thailand. This study aimed to assess safety of triple‐drug neonatal prophylaxis and NVP trough concentrations (C24) over the first 28 days of life.

**Methods:** A prospective cohort of 200 HIV‐exposed infants was conducted at five clinical sites in Thailand. We enrolled 100 high‐risk HIV‐exposed neonates (maternal HIV RNA >50 copies/mL prior to delivery or received antiretroviral therapy (ART) <12 weeks) who received AZT/3TC twice daily, plus NVP (4 mg/kg/dose) once daily, from birth for six weeks, and 100 standard‐risk HIV‐exposed neonates who received a 4‐week regimen of AZT. Blood tests to assess hematologic and liver toxicities were performed at birth, one, two and four months of life. Sparse plasma NVP concentrations were collected at day 1, 2, 7, 14, and 28 and assayed by a validated liquid chromatography‐mass spectrometry assay.

**Results: ** From October 2015 to November 2017, 200 infants were enrolled. Median (IQR) gestational age and birth weight were 38 (37 to 39) weeks and 2873 (2590 to 3184) g, respectively. Common maternal ART regimens were TDF/3TC or FTC (58%), AZT/3TC(32%) in combination with EFV (45%), ritonavir boosted protease inhibitor (28%). There was no significant difference of adverse events between triple prophylaxis and AZT alone (Table 1). Median (IQR) hemoglobin level among infants who received triple prophylaxis were 9.9 (9.0 to 11.4) g/dL, 10.1 (9.3 to 11.0), and 11.7 (11.0 to 12.3) at aged one, two and four months, respectively, which did not significantly differ between groups. No infants required blood transfusion. NVP concentrations were available from 48 infants (135 samples); median predicted NVP C24 were 1336 ng/mL, 2241, 2782, 2197, and 812 on days 1, 2, 7, 14, and 28 of life, respectively (Figure 1). All infants maintained NVP concentrations above the proposed prophylactic target threshold of 100 ng/mL during the first four weeks. Maternal EFV treatment did not affect infant NVP levels.

**Conclusions: **Six‐weeks of AZT/3TC/NVP in HIV‐exposed infants did not increase the risk of toxicity compared with an AZT regimen. Administration of 4 mg/kg of NVP from birth provided adequate NVP concentrations for prophylaxis during the first four weeks of life.

**Abstract THAB0305‐Table 1. Adverse event rates between triple and AZT prophylaxis**

**Table:** 

Adverse event rates+	6‐week AZT/3TC/NVP prophylaxis (n = 100)	4‐week AZT prophylaxis (n = 100)	*p*‐value*
All grade anemia	41.8%	39.3%	0.69
Grade 3 to 4 anemia	3.7%	3.3%	0.80
All grade neutropenia	5.3%	6.2%	0.58
Grade 3 to 4 neutropenia	1.1%	0.5%	0.39
Elevated aspartate transaminase (AST)	1.6%	1.6%	0.97
Elevated alanine transaminase (ALT)	3.7%	3.1%	0.66

+According to DAIDS Grading toxicity table 2014.

*p‐value calculated by Chi‐Square test.

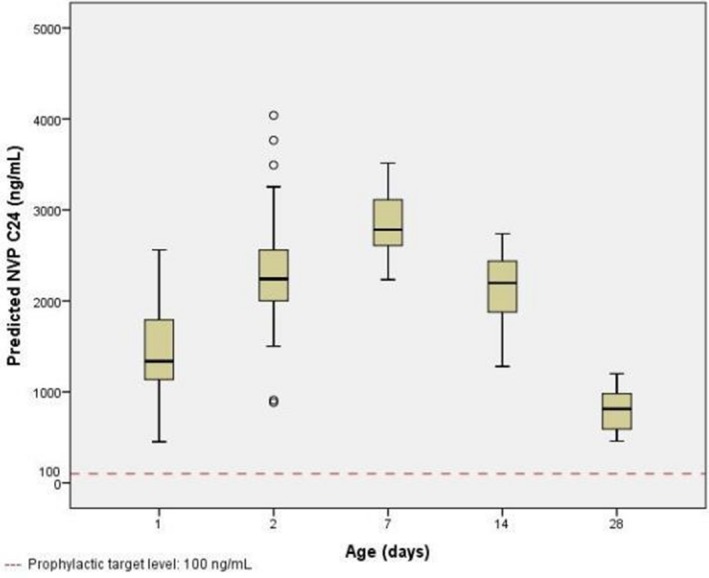

**Abstract THAB0305‐Figure 1. Predicted nevirapine trough concentrations (C24) (ng/mL) versus post‐natal age in days.**

## THAC0101

### The identification of a micro‐epidemic in a hyper‐endemic HIV setting using molecular epidemiology

**C. Coltart^1,2^; F. Tanser^2^; D. Gareta^2^; S. Ntuli^2^; T. de Oliveira^3^; D. Pillay^2^; A. Johnson^1^ and S. Hue^4^**

^1^University College London, Institute for Global Health, London, United Kingdom, ^2^Africa Health Research Institute, KwaZulu‐Natal, South Africa, ^3^University of KwaZulu‐Natal, Durban, South Africa, ^4^LSHTM, London, United Kingdom

**Background:** Understanding transmission dynamics of infectious diseases is critical in developing effective interventions. Traditional epidemiological analyses are the bedrock of many public health decisions, but can be resource‐intensive and may be limited by incomplete/inaccurate datasets. Since HIV gene sequences can provide information not contained in traditional case data, considerable efforts are now invested in generating genomic data. However, the value of such data for epidemiological surveillance remains unclear. We illustrate how incorporating genomic data with traditional epidemiological analyses provides additional insight for surveillance, with potential to inform prevention strategies.

**Methods:** We used routinely‐collected AHRI data to undertake a combined phylogenetic and epidemiological investigation.

A phylogeny of 2179 HIV‐1 subtype‐C partial pol sequences was reconstructed by maximum‐likelihood inference. This included 1376 local sequences (2010 to 2014, 15% of the HIV‐positive population) and 803 publicly‐available South African control sequences (2000 to 2014). A dated phylogeny was inferred using Beast2 and reproduction numbers (Re) estimated. We geo‐located individuals to their residence and undertook space‐time analyses to identify variations in HIV incidence.

**Results: ** Phylogenetic reconstruction revealed a previously undetected distinct monophyletic cluster (75 local sequences; branch support>90%). The dated phylogeny suggests this outbreak emerged from a single common source, experienced two bursts of infection (2012&2013/4; Re>1.8) and was still expanding at the time of sampling (2014) (Figure 1). Geo‐locating individuals/sequences revealed over 40% resided within a rural area adjacent to a recent (2008) coal mine development. Baseline characteristics of the cluster suggest demographics compatible with those working in the mine (higher proportion of employed males). Space‐time analysis confirmed the emergence of this rural cluster by identifying increased HIV incidence in the locality of the mine.

To our knowledge, this is the first time molecular methods have detected a micro‐epidemic not identified via traditional epidemiological surveillance.

**Conclusions: **By uncovering a micro‐epidemic our findings demonstrate that molecular epidemiology enhances traditional epidemiological analysis. Genomic data are a valuable addition to routine surveillance data, particularly in allowing detection of emerging micro‐epidemics in a hyper‐endemic region. Although, at present, implementation may be unrealistic in resource‐poor settings, sequence data is becoming increasingly affordable, and molecular methods should be considered to enhance surveillance and guide the development of optimal intervention strategies.

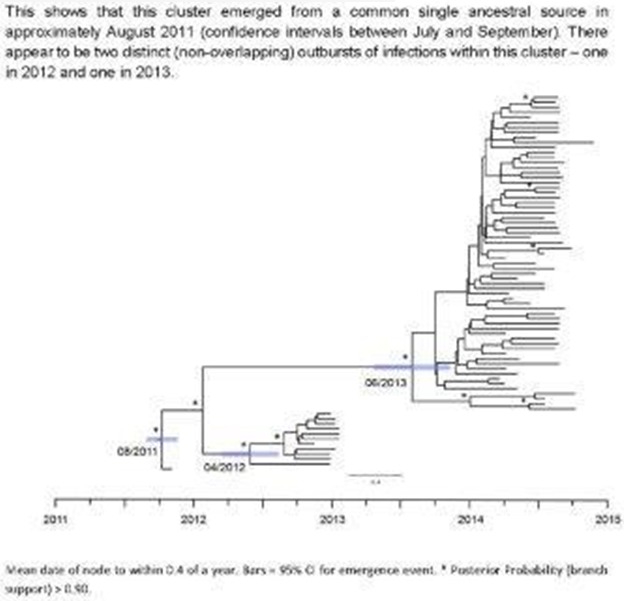

**Abstract THAC0101‐Figure 1. Dated Phylogeny of identified cluster.**

## THAC0102

### High prevalence fishing communities are not a major source of new HIV infections to the inland populations in Rakai District, Uganda: implications for geo‐spatially targeted HIV prevention interventions

**O. Ratmann^1,2^; M.K. Grabowski^3,4^; M. Hall^5^; T. Golubchik^5^; C. Wymant^2,5^; J. Kagaayi^4^; G. Kigozi^4^; T. Quinn^3,6^; M. Wawer^4,7^; O. Laeyendecker^3,6^; D. Serwadda^4,8^; R. Gray^3,4,7^; C. Fraser^5^; PANGEA‐HIV Consortium and Rakai Health Sciences Program**

^1^Imperial College London, Mathematics, London, United Kingdom, ^2^Imperial College London, School of Public Health, London, United Kingdom, ^3^Johns Hopkins Bloomberg School of Public Health, Medicine, Baltimore, United States, ^4^Rakai Health Sciences Program, Entebbe, Uganda, ^5^University of Oxford, Oxford Big Data Institute, Li Ka Shing Centre for Health Information and Discovery, Nuffield Department of Medicine, Oxford, United Kingdom, ^6^National Institute of Allergy and Infectious Diseases, Division of Intramural Research, Bethesda, United States, ^7^Johns Hopkins Bloomberg School of Public Health, Epidemiology, Baltimore, United States, ^8^Makerere University School of Public Health, Kampala, Uganda

**Background:** Targeting combination HIV prevention (CHP) to areas of high HIV prevalence is considered a cost‐efficient and essential strategy for reducing HIV incidence in sub‐Saharan Africa. Since 2014, the Ugandan National Antiretroviral Therapy Guidelines recommend targeted CHP to fishing communities on Lake Victoria, with an estimated HIV prevalence 25% to 40%, partly based on the assumption that fishing sites are a major source of HIV transmissions to the inland populations; however the validity of this assumption has not been empirically evaluated.

**Methods:** Between August 2011 and Oct 2014, individuals aged 15 to 49 years in 40 communities in Rakai District, Uganda, were tested for HIV and interviewed (sociodemographic, behavioral and health information). Households were geocoded, and communities were classified as Lake Victoria fishing (n = 4), agrarian (n = 27), or main road trading (n = 9) communities. Viral RNA from newly diagnosed participants was deep sequenced via *Illumina* instruments. The *phyloscanner* software package (https://github.com/BDI-pathogens/phyloscanner) was used to reconstruct HIV transmission networks, and to infer the direction of HIV transmission from deep sequence data. Transmission flows between fishing sites and agragrian/trading communities were estimated with Bayesian multi‐level models.

**Results: ** Of 23,719 individuals surveyed, 6205 were HIV‐positive, 4309 (69%) were antiretroviral naive, of whom 2803 (65%) were sequenced. 359 phylogenetically likely transmission events involving 676 individuals were reconstructed, with an estimated, expected 16% (11% to 23%) of false reconstructions. Direction of transmission could be inferred in 241 likely transmission events, with an estimated, expected 14% (7% to 26%) of false directions. Only 3/241 transmission events occurred from fishing sites to agrarian/trading communities. Adjusting for differences in participation and sequence sampling by age and community, an estimated 34.3% (28.6% to 40.5%) of transmissions occurred within fishing sites, 58.0% (51.2% to 64.6%) within agrarian/trading communities, 3.4% (1.7% to 6%) from fishing sites to agrarian/trading communities, and 4.0% (2% to 7.2%) from agrarian/trading communities to fishing sites.

**Conclusions: **HIV is infrequently transmitted from four high‐prevalence fishing sites to the population in 36 agrarian/trading communities further inland, based on population‐level NGS viral phylogenetic analysis. Our results suggest that targeted CHP to Lake Victoria fishing sites would not mitigate the broader HIV epidemic. Further studies in sub‐Saharan Africa are needed to assess the strategy of targeting CHP to various high prevalence hotspots.

## THAC0103

### An RNA‐seq gene expression signature identifies early HIV infection in rural Mozambique

**M. Judge^1^; E. Parker^1^; L. Pastor^2,3^; L. Fuente‐Soro^2^; C. Jairoce^4^; K. Carter^5^; D. Anderson^5^; D. Naniche^2,4^ and P. Le Souëf^1^**

^1^University of Western Australia, School of Paediatrics and Child Health, Perth, Australia, ^2^ISGlobal, Barcelona Centre for International Health Research (CRESIB), Barcelona, Spain, ^3^AIDS Research Institute – IrsiCaixa, Barcelona, Spain, ^4^Centro de Investigação em Saúde da Manhiça (CISM), Manhiça, Mozambique, ^5^Telethon Kids Institute, Perth, Australia

**Background:** Accurate measurement of HIV incidence is crucial in determining the effectiveness of preventative and therapeutic interventions, yet remains a global challenge. This study employed RNA‐seq to investigate changes in human gene expression during early HIV‐1 infection with the objective of identifying a transcriptomic signature able to differentiate early from long‐standing infection.

**Methods:** Individuals presenting to Manhiça District Hospital, Mozambique were screened for acute HIV infection (AHI), defined by positive viral load prior to seroconversion, with follow‐up over 18 months. Uninfected subjects, plus those with longstanding HIV, with and without anti‐retroviral therapy (ART), were recruited as controls. Peripheral blood mononuclear cells were collected and RNA extracted using RNeasy MinElute Clean‐up kit (Qiagen). Illumina 50 bp, single‐end RNA‐seq was performed. Read quality was assessed using FastQC. Reads were aligned to human reference genome hg19 using HISAT. Alignment quality was assessed using Samstat. Quantification and normalisation of read counts was performed using summarizeOverlaps (*GenomicAlignments* package) and voom (*limma* package), both from Bioconductor. Random Forest assessed the predictive accuracy of sample classification. Statistical analyses were performed using R version 3.2.0.

**Results: ** Fifty‐three AHI individuals had a median estimated time since infection of nine weeks (IQR 7.8 to 19.3 weeks). RNA‐seq was performed on 164 longitudinal samples from the AHI cohort, as well as 54 HIV negative, 24 chronic without ART, and 24 chronic with ART samples, resulting in an average of 23 million reads per sample, with 80% of reads mapped at high accuracy. All samples passed pre‐and post‐alignment QC. Fifteen genes were significantly differentially expressed during AHI compared with all controls (Figure 1). Random Forest analysis was able to predict biological group with a sample‐level error rate of 0.26.

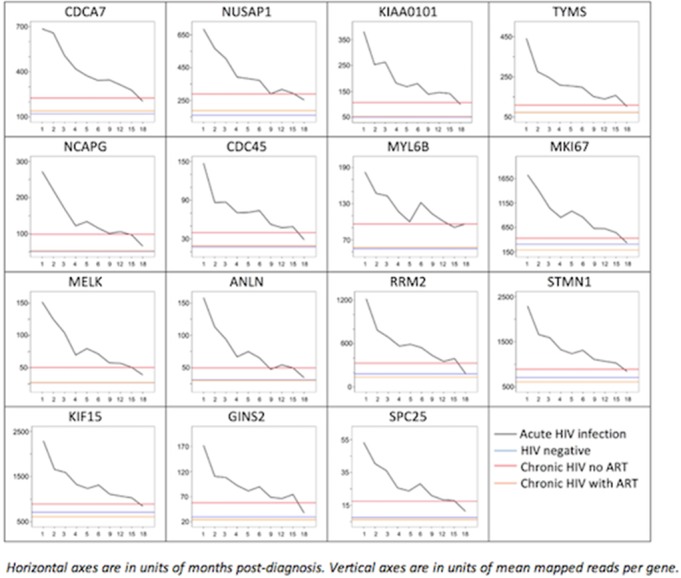

**Abstract THAC0103‐Figure 1. Expression patterns of the fifteen gene that are differentially expressed during early HIV infection compared to all controls.**

**Conclusions: **This exploratory transcriptomic analysis was able to identify a gene expression signature unique to early HIV infection, most effective at distinguishing the initial three months from longer‐term infections. For individual samples, acute infections and chronic infections with ART had high identification accuracy, however chronic untreated infection samples were harder to distinguish from early infection. The gene candidates in Figure 1 are clearly differentially expressed during early infection, compared with all controls, indicating useful pathways for future incidence assay optimisation.

## THAC0104

### Size estimation of key populations for HIV in Singapore using the network scale‐up method

**A.K.J. Teo^1^; K. Prem^1^; M.I.‐C. Chen^1,2^; A. Roellin^3^; M.L. Wong^1^; H.H. La^4^ and A.R. Cook^1^**

^1^Saw Swee Hock School of Public Health, National University of Singapore, Singapore, Singapore, ^2^Tan Tock Seng Hospital, Department of Clinical Epidemiology, Singapore, Singapore, ^3^National University of Singapore, Department of Statistics and Applied Probability, Singapore, Singapore, ^4^Center for High Impact Philanthropy and Center for Public Health Initiatives, University of Pennsylvania, Philadelphia, United States

**Background:** The size of populations at‐risk for HIV in Singapore has yet to be systematically estimated. Using the network scale‐up (NSU) approach, we developed a localised instrument and Bayesian statistical method to generate size estimates—adjusted for transmission error—of at‐risk populations in Singapore.

**Methods:** We conducted nine in‐depth interviews and four focus groups discussions with key stakeholders to guide the development of the survey questionnaire. In total 199 participants recruited from the Singapore Population Health Studies cohort reported their socio‐demographic information, opinions about certain behaviours and the social standing of different groups of people, and quantified the number of people whom they knew. We developed a Bayesian hierarchical NSU model to estimate the number of individuals in four hidden populations at‐risk of HIV in Singapore. The method accounted for both transmission error and barrier effects using social acceptance measures and demographics.

**Results: ** The adjusted size estimate of the population of male clients of female sex workers was 72,000 (95% CI: 51,000 to 100,000), of female sex workers 4200 (95% CI: 1600 to 10,000), of men who have sex with men 210,000 (95% CI: 140,000 to 300,000), and of intravenous drug users 11,000 (95% CI: 6500 to 17,000). Average estimated personal network sizes were 140 (95% CI: 82 to 238).

**Conclusions: **The network scale‐up method with adjustment for attitudes and demographics allows national‐level estimates of multiple priority populations to be determined from simple surveys of the general population, even in relatively conservative societies.

## THAC0105

### Challenges ahead: the impact of future demographic transitions on HIV epidemic control among adolescents and youth in sub‐Saharan Africa

**A. Khalifa^1^; C. Lwamba^1^; T. Porth^1^; S. Essajee^2^; D. Walker^2^; L.D. Tsage^3^; A. Bains^4^ and C. Luo^2^**

^1^United Nations Children's Fund, Data, Research and Policy, New York, United States, ^2^United Nations Children's Fund, Programme, New York, United States, ^3^United Nations Children's Fund, West and Central Africa Regional Office, Dakar, Senegal, ^4^United Nations Children's Fund, Eastern and Southern Africa Regional Office, Nairobi, Kenya

**Background:** Despite progress in the HIV response, new infections among adolescents and youth (AY) aged 15 to 24 in Sub‐Saharan Africa (SSA) have only reduced by an average 3%/year since 2010. By 2050, the number of AY in SSA will increase by 85%. This demographic transition may impact the HIV epidemic and service needs for future generations. We used population statistics and recent epidemic trends to characterize the future of the HIV epidemic in SSA.

**Methods:** For 46 countries, we organized UNAIDS HIV estimates and UN population projections into five‐year age groups by sex. HIV incidence and prevalence was calculated from 2010 to 2016. After analyzing trends, 2014 to 2016 data reflecting recent progress in disease control were selected to inform regression analyses by country, age and sex. An exponential curve was applied for downward incidence trends and a linear curve for upward incidence trends and prevalence. From each regression, HIV incidence and prevalence was predicted until 2050. Incidence and prevalence were applied to projected populations in each year to determine new infection numbers. Results were analysed to assess feasibility of epidemic control among AY 15 to 24, defined as 95% reduction in new infections since 2016.

**Results: ** By 2050, new HIV infections will decrease by over 70% in Eastern and Southern Africa and by 2% in West and Central Africa (Figure 1). Overall, the SSA region will observe a 53% reduction in new HIV infections. None of 46 SSA countries will achieve epidemic control among AY by 2030. Botswana, Mozambique, Uganda, Zimbabwe, and Swaziland may reach epidemic control by 2050. Of these five countries, Botswana, Swaziland, and Zimbabwe will reduce new infections among boys and men at least two years before they will in girls and young women. Between 2017 and 2050, 9.6 million AY will become newly infected with HIV in SSA, 67% of which will occur among girls and young women.

**Conclusions: **While the world set a bold target of ending AIDS by 2030, epidemic control is unlikely among AY in SSA. Turning this demographic transition into a dividend will require better access to HIV prevention, sexual and reproductive health, and targeted testing services.

## THAC0201

### Delivering a high‐quality comprehensive package of HIV prevention, care, and treatment for key populations is possible: experience from two years of the FHI 360 LINKAGES Malawi project

**G. Kamanga^1^; L. Banda^1^; L. Banda^1^; C. Banda^1^; M. Mkandawire^1^; S. Kalyati^1^; E. Mpunga^1^; M. Gondwe^1^; M. Ruberintwari^1^ and C. Akolo^2^**

^1^FHI 360, Malawi, Global Health, Population and Nutrition, Lilongwe, Malawi, ^2^FHI 360, Global Health, Population and Nutrition, Washington DC, United States

**Background:** In Malawi, HIV prevalence is 8.8% among the general population but higher among key populations (KPs): 62.7% among female sex workers (FSW) and 17.5% among men who have sex with men (MSM). FHI 360, through the USAID/PEPFAR‐funded LINKAGES project, provides comprehensive HIV prevention, care, and treatment services for KPs. We present our experience implementing this project over a two‐year period.

**Description:** We engaged government structures at all levels, KPs, and civil society organizations (CSOs) to get the project running. Programmatic mapping of hot spots and size estimation were conducted through engagement of KPs. Using a peer‐led model, KPs were recruited to support others with HIV prevention services, linkage to care, antiretroviral therapy (ART), and retention. We built the capacity of peer leaders through trainings and microplanning, created safe spaces, and trained health care workers to mitigate stigma and discrimination.

**Lessons learned:** From October 2016 to June 2017, the project reached 9601 FSWs, 3609 (38%) of whom were already HIV positive, and 5136 of whom were eligible for HIV testing; of the latter, 2068 (40%) tested HIV positive and 1862 (90%) were initiated on ART. The total number of HIV‐positive cases detected, 5677/9601 (59.1%), is close to the 62.7% estimated HIV prevalence among FSWs in Malawi. A total of 3025 HIV‐positive FSWs were enrolled in community care. We screened 13,827 FSWs for STIs, diagnosed 5119 (37%) cases, and treated 5108. Of 2696 MSM reached with services, 2561 were tested for HIV, 188 (7%) tested HIV positive, and 114 (61%) were initiated on ART. We screened 4726 MSM for sexually transmitted infections, diagnosed 1585 (34%) cases, and treated 1507. Eighty‐seven FSW and 39 MSM reported gender‐based violence and received services. We identified 239 transgender women and are now receiving HIV prevention, care, and treatment.

**Conclusions/Next steps:** Empowered KP members positively contribute to their health. In addition, engagement with government, health care workers, and peer leaders is key to ensuring a successful KP program. Efforts are ongoing to document and scale up some of the best practices emanating from the program.

## THAC0202

### Key population‐led health services: community‐based organizations and lay health workers transform HIV testing in Vietnam

**H. Ngo^1^; N.B. Vu^2^; K. Green^2^; H. Phan^3^; H.S. Vo^3^; M.T. Ngo^4^; M.S. Le^5^; T. Le^6^; H.A. Doan^2^ and A. Bao^7^**

^1^PATH, Healthy Markets, Ha Noi, Vietnam, ^2^PATH, Ha Noi, Vietnam, ^3^Ministry of Health, Ha Noi, Vietnam, ^4^USAID, Ha Noi, Vietnam, ^5^G3VN, Ho Chi Minh City, Vietnam, ^6^G‐LINK, Ho Chi Minh City, Vietnam, ^7^PATH, Ho Chi Minh City, Vietnam

**Background:** Vietnam became the first Asian country to adopt global 90‐90‐90 targets, despite low annual uptake (˜30%) of HIV testing among key populations (KPs). Up until late 2015, lay providers were not allowed to offer HIV testing, although studies indicated KP preference for community testing services.

**Description:** The USAID/PATH Healthy Markets project and Ministry of Health (MOH) piloted HIV lay provider testing in four provinces. From October 2015‐September 2017, services were delivered by KP‐led community‐based organizations (CBOs) in urban areas and village health workers (VHWs) in rural areas, using blood‐ and oral fluid‐based rapid diagnostic tests (RDTs). This was the first time CBOs were allowed to offer HIV services in Vietnam.

Trained CBOs/VHWs administered the RDT, interpreted results, provided post‐test counseling supported those that were HIV‐reactive to seek confirmation testing, and their enrollment in treatment. Coaching was provided to trained lay providers to reinforce service quality. 59,333 clients were HIV‐tested, 2503 (4.2%) were newly diagnosed HIV‐positive and 90% enrolled in treatment.

**Lessons learned:** The pilot generated four key lessons: First, that CBOs/VHWs were capable of offering HIV testing with exceptional skills, and engagement in lay testing significantly boosted CBO confidence and pride in their work. Second, CBOs/VHWs were very effective in facilitating ART enrollment among those HIV‐diagnosed. Third, among those tested by lay testers, more than half were first time testers, and 4.2% were newly HIV‐diagnosed (compared to 1.6% among KPs testing in public sites). This suggests trust among KP for HIV lay provider services. Last, fostering close MOH engagement and observation of CBO/VHW testers rapidly translated into national trust and support for their work.

**Conclusions/Next steps:** Stepwise rollout of HIV lay provider testing positively disrupted the status quo, and has opened the door for KP‐led HIV services. The pilot led to the development of national HIV community testing guidelines and training package, and rapid adoption of the approach by the MOH, PEPFAR partners and Global Fund. For the future, it will be critical to target HIV lay testing to localities that need it most, and secure domestic financing sustained service delivery.

## THAC0203

### Respondent‐driven sampling more efficiently identifies undiagnosed HIV‐infected people who inject drugs (PWID) than PWID‐targeted community integrated care centers in India

**A.M. McFall^1^; S.S. Solomon^2^; A.K. Srikrishnan^3^; S. Anand^3^; C.K. Vasudevan^3^; G.M. Lucas^2^ and S.H. Mehta^1^**

^1^Johns Hopkins Bloomberg School of Public Health, Epidemiology, Baltimore, United States, ^2^Johns Hopkins University School of Medicine, Baltimore, United States, ^3^YR Gaitonde Centre for AIDS Research and Education, Chennai, India

**Background:** Injection drug use drives HIV epidemics in many low‐resource settings. Yet, many people who inject drugs (PWID) are inadequately engaged in HIV services, resulting in low awareness among HIV‐infected PWID. Respondent‐driven sampling (RDS), a method using peer connections, is widely used in research among key populations. We assessed the ability of RDS to identify undiagnosed HIV‐infected PWID compared to integrated care centers (ICCs) in India.

**Methods:** In 6 Indian cities from 2014 to 2017, ICCs provided PWID‐tailored services such as HIV counseling/testing and needle exchange; ICC utilization was voluntary. In these same cities from 2016 to 2017, an RDS sample of 1000 PWID/city was conducted; RDS participants were compensated for time and referrals. The number needed to recruit (NNR) (number of PWID screened in order to find one undiagnosed person living with HIV (PLWH)) and the identification rate (number of undiagnosed PLWH identified per week) assessed the efficiency of RDS vs. ICCs. Undiagnosed PLWH were individuals who tested positive and denied a prior diagnosis. Multinomial logistic regression was used to explore characteristics associated with identification by RDS only and RDS & ICC, both in comparison to ICC only.

**Results: ** Across the six cities, there were 10,759 ICC clients and 6012 RDS participants; 40% of RDS participants were ICC clients (confirmed via biometrics) resulting in 14,397 unduplicated PWID, of which 753 (5%) were undiagnosed PLWH. The RDS NNR ranged from five to twenty‐seven and the ICC NNR ranged from 10 to 74. The NNR was lower for RDS versus the ICC in all but one city, Bilaspur (Figure 1). The RDS identification rate (1.7 to 2.8/week) was faster than the ICC identification rate (0.2 to 1.0/week) in all cities (Figure 1). PWID identified by RDS vs. the ICC only were more likely to be male (adjusted odds ratios (aOR) RDS only: 6.8, both: 2.7) and HIV‐infected but undiagnosed (aOR RDS only: 2.5, both: 1.5).

**Conclusions: **In India, RDS required screening fewer PWID and more rapidly identified undiagnosed PLWH as compared to ICCs. Network‐driven recruitment strategies with moderate compensation could be considered to identify and engage groups of PWID not currently visiting venues where HIV and harm reduction services are available.

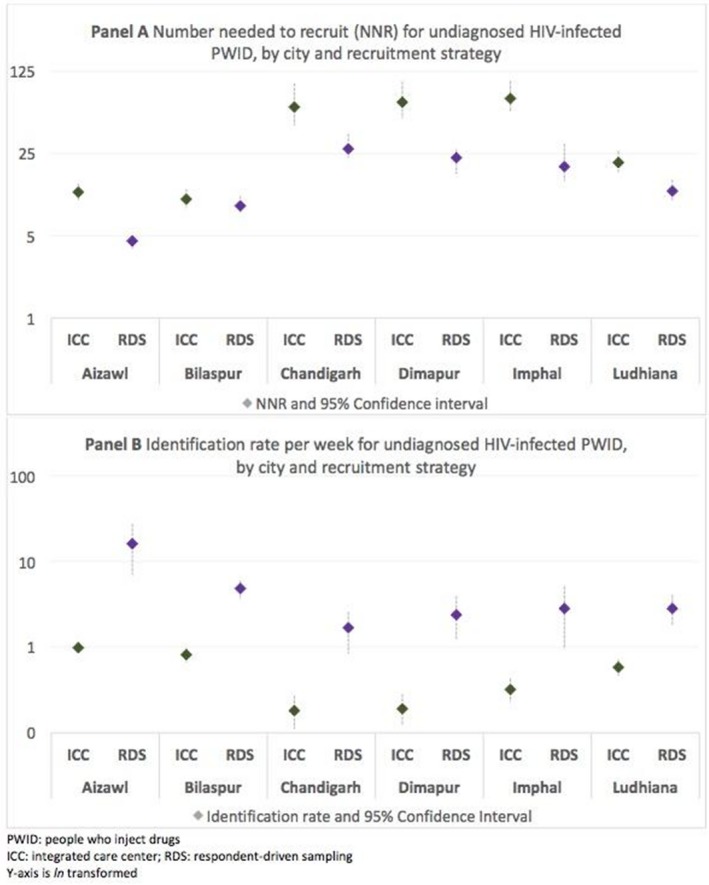

**Abstract THAC0203‐Figure 1. Number needed to recruit (NNR) and identification rate for undiagnosed HIV‐infected PWID in India.**

## THAC0204

### Integrated gender affirmative hormone treatment services improve access to and retention in HIV testing, syphilis testing and pre‐exposure prophylaxis (PrEP) service uptake among transgender women in Thailand

**R. Janamnuaysook^1^; K. Samitpol^1^; P. Ketwongsa^1^; A. Chancham^1^; J. Kongkapan^1^; P. Mingkwanrungruang^1^; R. Meksena^1^; P. Rattakitvijunnanakorn^1^; N. Teachatanawat^1^; S. Mills^2^; S. Charoenying^2^; S. Umasa^2^; R. Vannakit^3^; P. Phanuphak^1^ and N. Phanuphak^1^**

^1^Thai Red Cross AIDS Research Centre, Prevention, Bangkok, Thailand, ^2^FHI 360 and USAID LINKAGES Project, Bangkok, Thailand, ^3^United States Agency for International Development, Office of Public Health, Bangkok, Thailand

**Background:** Despite the growing recognition of gender affirmative hormone treatment (GAHT) as an essential part of comprehensive health care for transgender women, evidence is limited on the roles of GAHT‐services in HIV programming for transgender women. We examined the potential role of GAHT‐services as an entry‐point to facilitate uptake of HIV and other sexual health services.

**Methods:** Established in 2015, the Tangerine Community Health Center is the first clinic in Asia where fee‐based GAHT‐services are fully integrated with HIV services. Characteristics of transgender women clients and their access to GAHT‐services and other health services were recorded. We compared the uptake of HIV and other sexual health services between transgender women who accessed or did not access GAHT‐services.

**Results: ** Of 972 transgender women who attended the clinic between November 2015‐December 2017, median (IQR) age was 25.4 (22.5 to 30) years, 55% had education below bachelor's degree, 25% were unemployed, 56% used alcohol, and 10% used amphetamine‐type stimulants. GAHT‐services were used by 34% of transgender women. At baseline, 91% received HIV testing, and HIV prevalence was 12%. Antiretroviral therapy initiation was successful in 80%. Transgender women who did not use GAHT‐services had lower income (65% vs. 45% earned <$571/month, *p* < 0.001), higher HIV prevalence

(13% vs. 3%, *p* < 0.001), and a trend toward higher HIV incidence than those who used GAHT‐services. Compared to clients not accessing GAHT‐services, GAHT‐service clients were more likely to re‐visit the clinic (50% vs. 34%, *p* < 0.001), had higher rates of repeat HIV testing (32% vs. 25%, *p* = 0.019), repeat syphilis testing (14% vs. 9%, *p* = 0.026), PrEP uptake (10% vs. 6%, *p* = 0.015), and use of other sexual health services, including hepatitis B testing/vaccination and sexually transmitted infection treatment (50% vs. 34%, *p* < 0.001).

**Conclusions: **Integration of GAHT‐services into HIV programming in this cohort showed improvement of retention in the program and subsequent increase of sexual health service utilization, including PrEP. Transgender women not accessing GAHT‐services appeared to be highly vulnerable to HIV infection. HIV program managers, policymakers, and institutional donors should consider investing in well‐tailored, subsidized, and comprehensive HIV and sexual health service packages that fully integrate GAHT‐services for transgender women.

## THAC0205

### Continuing structural barriers to HIV/STI testing for migrants attending a community‐based HIV testing service in Melbourne, Australia

**K. Ryan^1^; A. Wilkinson^2^; D. Leitinger^3^; P. Locke^3^; A. Pedrana^1^; M. Hellard^1^ and M. Stoove^1^**

^1^Burnet Institute, Public Health Discipline, Melbourne, Australia, ^2^Cancer Council Victoria, Centre for Behavioural Research in Cancer, Melbourne, Australia, ^3^Victorian AIDS Council, Melbourne, Australia

**Background:** In February 2016 PRONTO!, a peer‐led community‐based rapid HIV testing service, introduced STI (gonorrhoea, chlamydia, syphilis) testing and SMS reminders to improve return HIV testing. HIV testing is free for all clients. Clients eligible for Australia's universal healthcare system, including Australian, New Zealand and UK citizens (eligible), receive free STI testing. Citizens of other countries (ineligible) pay up to $158AUD upfront. We determined changes to return‐HIV testing pre‐ and post‐service changes among eligible and ineligible clients to explore ongoing barriers to accessing sexual healthcare among migrants.

**Methods:** All HIV tests conducted between February 2014 and September 2017 with country of birth recorded were included in the analysis (February‐July2014 allowed lead time to assess returning). We describe STI testing uptake and HIV/STI positivity among eligible and ineligible clients. Return HIV testing was compared between pre‐intervention (August 2014‐January 2016) and post‐intervention (February 2016‐September 2017) periods. Segmented linear regression of monthly aggregate data assessed changes in the percentage of tests that returned within 183 days (six month return testing) among eligible and ineligible clients across pre‐ and post‐intervention periods. We report changes in six month return testing pre‐ to post‐intervention (change in slope, β3), *p* < 0.05 was considered significant.

**Results: ** This analysis included 2679 eligible and 1619 ineligible clients. Post intervention there was a significant difference in the proportion of HIV tests accompanied by STI tests among eligible clients (82%) and ineligible clients (49%; *p* < 0.01); however among those accessing testing, STI positivity was similar (12% and 12%, respectively). Four (0.3%) eligible and nine (1.1%) ineligible clients were diagnosed with HIV. Post‐intervention, six month HIV return testing increased significantly among eligible (β3 0.7% per month, 95% CI: 0.1 to 1.3, *p* = 0.02) clients from 22% to 50% and ineligible (β3 0.9% per month, 95% CI: 0.4 to 1.5, *p* < 0.01) clients from 19% to 44% (Figure 1).

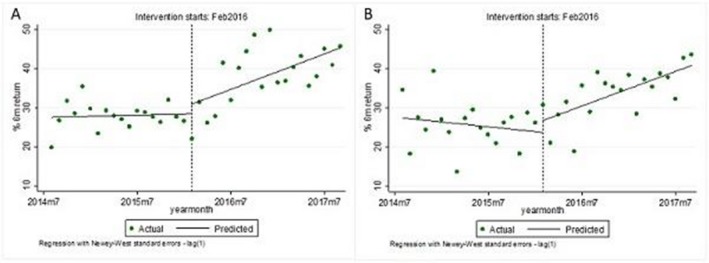

**Abstract THAC0205‐Figure 1. Six month return testing among eligible(A) & ineligible(B) clients pre(Aug2014‐Jan2016) & post(Feb2016‐Sept2016) service level changes.**

**Conclusions: **Increases in six month HIV return testing were observed among eligible and ineligible clients following the introduction of STI testing and SMS reminders. However, the significantly lower uptake of fee‐for‐service STI tests among overseas‐born clients compared to uptake of free STI tests among eligible clients suggests ongoing financial barriers to comprehensive HIV and STI testing in this group.

## THAC0301

### HIV and pregnancy outcomes from the Sakh'umndeni Safer Conception Clinic

**S. Schwartz^1^; J. Bassett^2^; M. Mudavanhu^2^; N. Yende^2^; R. Phofa^2^; L. Mutunga^2^; I. Sanne^3^ and A. Van Rie^4^**

^1^Johns Hopkins University, Epidemiology, Baltimore, United States, ^2^Witkoppen Health and Welfare Centre, Johannesburg, South Africa, ^3^Clinical HIV Research Unit, University of the Witwatersrand, Johannesburg, South Africa, ^4^University of Antwerp, Antwerp, Belgium

**Background:** Safer conception strategies–ART for HIV‐positive partners, PrEP for HIV‐negative partners, timed condomless sex, self‐insemination and male medical circumcision–empower couples affected by HIV trying to conceive to minimize HIV transmission risk to partners and potential children. We report outcomes from the Sakh'umndeni demonstration project, one of the first safer conception clinics in sub‐Saharan Africa.

**Methods:** Adults trying to conceive and in relationships in which at least one partner was HIV‐positive were enrolled into safer conception care at Witkoppen Clinic in Johannesburg, South Africa, between July 2013‐July 2017. Patients were provided tailored safer conception care by a nurse. Time‐to‐first pregnancy was estimated using Kaplan‐Meier curves; women who did not conceive were censored at date of termination or last follow‐up visit.

**Results: ** 526 individuals (334 women/192 men) from 334 partnerships participated. Couples were serodifferent (n = 164, 49%), seroconcordant (n = 147, 44%) or in relationships with one unknown status partner (n = 23, 7%). Median ages of women and men were 34 (IQR: 30 to 38) and 37 (IQR: 33 to 42) respectively. At baseline, 64% of HIV‐positive women and 45% of HIV‐positive men were virally suppressed (< 50copies/ml). It took couples a median of 4.0 months (IQR: 1.7 to 7.7) to be given the go‐ahead to start trying to conceive. In total, 98 pregnancies among 88 women were observed. Pregnancy incidence was 47.9/100 person‐years (95% CI: 38.9, 59.1). HIV‐positive women were 52% less likely to conceive as HIV‐negative women (IRR: 0.48, 95% CI: 0.28,0.87). Median time‐to‐pregnancy was 0.8 years for HIV‐negative and 2.1 years for HIV‐positive women (Figure 1). At time of pregnancy, most HIV‐positive women were virally suppressed (63/75 (84%) <50 copies/mL and 74/75 (99%) <1000 copies/mL). Of the 98 pregnancies, 66 (67%) delivered a baby, 24 (25%) had a miscarriage or ectopic pregnancy, 5 (5%) were still pregnant and 3 (3%) unknown. No horizontal or vertical HIV transmissions were observed.

**Conclusions: **HIV‐positive women were less likely to conceive than HIV‐negative women and risk of miscarriage was high. Prolonged attempted conception highlights the need for approaches to reduce onward transmission risks, particularly as viral suppression among patients trying to conceive on ART cannot be assumed. Safer conception strategies can help couples successfully conceive an HIV‐free child without jeopardizing their own or partner's health.

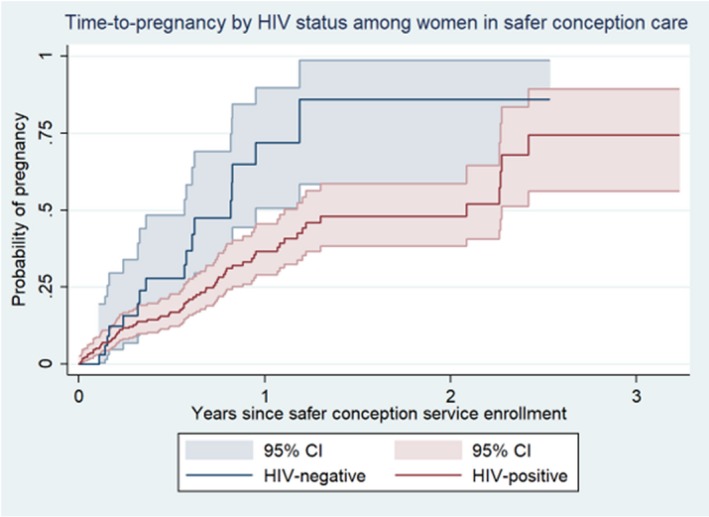

**Abstract THAC0301‐Figure 1. Time‐to‐pregnancy among women enrolled in safer conception care, Johannesburg, South Africa.**

## THAC0302

### HIV mother‐to‐child transmission in Cameroon: early infant diagnosis positivity rates by entry point and key risk factors

**P. Tchendjou^1^; S. Lekeumo^1^; T. Binde^1^; A.C. Bissek^2^; G. Dongmo^1^; J. Cohn^3^; E. Sacks^4^; C. Kengne^5^; A. Beng Amougou^5^ and V. Nzima^1^**

^1^Elizabeth Glaser Pediatric AIDS Foundation, Yaounde, Cameroon, ^2^Ministry of Health/Division of Operational Research, Yaounde, Cameroon, ^3^Elizabeth Glaser Pediatric AIDS Foundation, Geneva, Switzerland, ^4^Elizabeth Glaser Pediatric AIDS Foundation, Washington D.C., Cameroon, ^5^National AIDS Control Committee, Yaounde, Cameroon

**Background:** Prevention of mother‐to‐child transmission (PMTCT) programs aimed at reducing pediatric HIV infections are frequently assessed by the MTCT rate collected from PMTCT entry points, but this misses positivity rates in other entry points. Using the opportunity of the introduction of point of care early infant diagnosis (POC EID) in Cameroon, we extended infant HIV testing to several entry points of health facilities. We assessed HIV positivity by entry point and key risk factors.

**Methods:** A cross‐sectional study nested within the POC EID project implemented in four regions was conducted in 58 health facilities of varying levels. Clinical history of the mother‐baby pair, assessment of HIV status of the mother were used as eligibility criteria of infants. In each health facility, all entry points were considered and categorized as either a PMTCT entry point or a non‐PMTCT entry point. Eligible infants presenting to these facilities between December 2016 and December 2017 were tested by POC EID. Variables including demographics, antiretroviral use, and breastfeeding history were extracted from the EID request form. Data were analyzed using multivariate analysis with backward elimination (*p* > 0.20).

**Results: ** Overall, 2254 HIV‐exposed infants were tested using POC EID as first HIV diagnosis. The median age at sample collection was 7.3 weeks (IQR (6.3;19.0)). The main entry points were PMTCT (48.7%), immunization unit (14.3%), Pediatric ward (13.8%). Of the 2254 infants tested, 8.7% (197/2,254) were HIV‐positive. This rate varied according to entry points (outpatient department, 19.2%; emergency/pediatric ward, 17.7%; PMTCT/antiretroviral treatment (ART), 5.7%). In univariate analysis, positive cases were more likely to be found at non‐PMTCT entry point, among females, and infants delivered to HIV‐positive women who received incomplete ARVs for PMTCT. In multivariate analysis, risk of being HIV‐positive was higher when the infant was found at non‐PMTCT entry point (OR: 2.09; 95% CI: 1.47 to 2.99; *p < *0.001), was on mixed feeding mode (OR: 3.74; 95% CI: 2.43 to 3.47; *p < *0.001).

**Conclusions: **Only 48% of infected infants came from PMTCT‐entry point. EID positivity rates were highest in non‐PMTCT entry points. Strengthening testing in non‐PMTCT entry points help to address missed opportunities of PMTCT programs and link more children into ART care.

## THAC0303

### Prevention of mother to child transmission and early infant diagnosis in Malawi: accomplishments of a mature Option B+ program in a resource‐limited setting

**E. Kim^1^; J. Cuervo‐Rojas^2^; T. Kalua^3^; A. Jahn^3^; D. Payne^4^; C. West^4^; M. Nzima^5^; N. Wadonda‐Kabondo^4^; S. Jonnalagadda^6^ and MPHIA Study Team**

^1^Centers for Disease Control and Prevention, Division of Global HIV and TB, Lilongwe, Malawi, ^2^ICAP at Columbia University, New York, United States, ^3^Ministry of Health, Lilongwe, Malawi, ^4^Centers for Disease Control and Prevention, Lilongwe, Malawi, ^5^UNAIDS, Lilongwe, Malawi, ^6^Centers for Disease Control and Prevention, Atlanta, United States

**Background:** Malawi spearheaded the development and implementation of the Option B+ policy for prevention of mother to child transmission of HIV (PMTCT). From mid‐2011, all HIV‐infected pregnant and breastfeeding women were eligible for life‐long ART. Routine aggregate program data indicated successful rapid scale‐up, but some concerns about uptake and retention remained. We measured PMTCT and early infant diagnosis (EID) coverage in the 2015 to 2016 Malawi Population‐based HIV Impact Assessment (MPHIA).

**Methods:** MPHIA was a nationally representative household survey; eligible women were consented and interviewed on pregnancies and outcomes, including self‐reported HIV status during their most recent pregnancy, uptake of PMTCT, and EID testing. Women aged 15 to 49 years reporting a live birth in the 36 months before the survey were included in this analysis. Descriptive and multivariable logistic regression analyses were weighted to account for the complex survey design.

**Results: ** A total of 3598 women reported a live birth in the 36 months before the survey; mean age was 26.9 years and mean parity was 3.0. Of these, 96.2% (95% confidence intervals (CI): 95.5 to 97.0) reported being tested for HIV and receiving their results or knowing their HIV status during their last pregnancy; 7.4% (95% CI: 6.5 to 8.4) self‐reported their HIV‐positive status during pregnancy. Of the 302 women self‐reporting their HIV‐positive status, 98.1% (95% CI: 96.5 to 99.6) reported being on ART during pregnancy and 81.0% (95% CI: 75.6 to 86.4) reported that their child was tested for HIV; 50.6% (95% CI: 43.1 to 58.1) reported EID testing within two months of birth. Of those reporting that their child was tested for HIV, 3.1% (95% CI: 0.5 to 5.7) reported a HIV‐positive result.

Adjusting for age and urban/rural residence, EID testing within two months of birth was associated with secondary or higher education (adjusted odds ratio (aOR) 3.1, 95% CI: 1.5 to 6.2), disclosure of mother′s HIV‐positive status to family (aOR 2.4, 95% CI: 1.3 to 4.6), and uptake of cotrimoxazole (aOR 6.2, 95% CI: 2.7 to 14.2).

**Conclusions: **Data from the 2015 to 2016 MPHIA demonstrate the success of Malawi′s PMTCT Option B+ program at a population level, with high uptake across the PMTCT cascade. However, challenges remain in the timeliness of EID testing for HIV‐exposed infants according to recommended guidelines.

## THAC0304

### Birth outcomes and HIV‐free survival with Option B+ in Lesotho: results from an observational prospective cohort study

**A. Tiam^1,2^; S. Kassaye^3^; R. Machekano^1^; V. Tukei^4^; M. Gill^1^; M. Mokone^4^; S. Mohale^4^; M. Letsie^5^; M. Tsietso^6^; I. Seipati^7^; J. Barisa^4^; A. Isavwa^8^ and L. Guay^1,9^**

^1^Elizabeth Glaser Pediatric AIDS Foundation, Research, Washington, United States, ^2^University of Bergen, Centre for International Health, Bergen, Norway, ^3^George Town University, Medicine and Infectious Disease, Washington, United States, ^4^Elizabeth Glaser Pediatric AIDS Foundation, Research, Maseru, Lesotho, ^5^Ministry of Health, Disease Control, Maseru, Lesotho, ^6^Ministry of Health, Laboratory Services, Maseru, Lesotho, ^7^Ministry of Health, Family Health, Maseru, Lesotho, ^8^Elizabeth Glaser Pediatric AIDS Foundation, SI & E, Maseru, Lesotho, ^9^George Washington University, Milken Institute School of Public Health, Washington, United States

**Background:** Combination antiretroviral therapy (cART) reduces mother‐to‐child transmission of HIV and improves maternal health. Since introduction of option B+, there are scant data on birth outcomes of HIV‐exposed compared to unexposed infants. We assessed birth outcomes and six‐week HIV free survival among HIV‐exposed infants (HEI) and HIV‐unexposed infants (HUI).

**Methods:** 941 HIV‐negative and 653 HIV‐positive pregnant women were enrolled in an observational cohort to evaluate effectiveness of universal maternal cART (Option B+) rolled out within routine programs in 13 health facilities in Lesotho. Birth outcomes included infant birth weight (IBW), maturity, congenital anomalies, and mortality. Infant HIV birth testing by DNA PCR within two weeks of birth was introduced at study sites alongside routine six‐week testing. Data were analysed to determine birth outcomes, HIV transmission, and HIV‐free survival rates at six weeks.

**Results: ** HIV‐positive women were older, 28.7 versus 24.4 years (*p* < 0.001) and presented for antenatal care earlier at 23 weeks versus 25.3 weeks gestation (*p* < 0.001). Mean IBWs were similar: 3.0 kgs for HEIs vs. 3.1 kgs for HUI. HEI were more likely to be premature, 8.3% vs. 4.0% (*p* = 0.001). Neither Age (median age: 26 vs. 25) nor parity (median: 1 vs. 1) was associated with prematurity. No differences in stillbirths or congenital anomalies were noted. Six infants were HIV‐infected by six weeks: cumulative HIV transmission was 0.9% (N = 4) at birth (95% CI: 0.25% to 2.36%) and 1.03% (N = 6) (95% CI: 0.38% to 2.23%) by six weeks. Infant mortality was 4.4% and 4.3% for HUI and HEI respectively (*p* = 0.93). The estimated six‐week HIV free survival was 91.5% (95% CI: 89.1% to 93.6%) for HEI. Survival for HUI was 94.1% (95% CI: 92.4% to 95.6%). Excluding stillbirths, six‐week HIV free survival for HEI was 95.2% (95% CI: 93.2% to 96.8%) compared to survival rate of 97.5% (95% CI: 96.2% to 98.4%) among HUI (*p* = 0.02).

**Conclusions: **A low HIV transmission rate by six weeks was found among mother‐infant pairs enrolled in a universal cART prevention of mother‐to‐child transmission program, though there were higher rates of prematurity; six‐week survival among HIV‐exposed infants was comparable to HIV‐unexposed infants. It will be important to explore if this trend continues at 12 months and 24 months.

## THAC0305

### Cohort study of HIV+ children in Southern Africa returning to care after being lost to follow up: effect of interrupting care on mortality

**C. Davies^1^; S. Sawry^2^; C. Chimbetete^3^; B. Eley^4^; M. Vinikoor^5^; K. Technau^6^; J. Ehmer^7^; H. Rabie^8^; S. Phiri^9^; F. Tanser^10^; K. Malisita^11^; G. Fatti^12^; M. Osler^1^; R. Wood^13^ and M.‐A. Davies^1^**

^1^University of Cape Town, Centre for Infectious Disease Epidemiology and Research, School of Public Health and Family Medicine, Cape Town, South Africa, ^2^Chris Hani Baragwanath Academic Hospital, Harriet Shezi Children's Clinic, Soweto, South Africa, ^3^Newlands Clinic, Harare, Zimbabwe, ^4^University of Cape Town, Red Cross Children's Hospital and School of Child and Adolescent Health, Cape Town, South Africa, ^5^Centre for Infectious Disease Research in Zambia, Lusaka, Zambia, ^6^Rahima Moosa Mother and Child Hospital and University of the Witwatersrand, Johannesburg, South Africa, ^7^SolidarMed, Lucerne, Switzerland, ^8^University of Stellenbosch, Department of Paediatrics and Child Health, Tygerberg Academic Hospital, Stellenbosch, South Africa, ^9^Kamuzu Central Hospital, Lighthouse Trust Clinic, Lilongwe, Malawi, ^10^University of KwaZulu‐Natal, Africa Centre for Health and Population Studies, Somkhele, South Africa, ^11^Queen Elizabeth Central Hospital, Umodzi Family Centre, Blantyre, Malawi, ^12^Kheth'Impilo AIDS Free Living, Cape Town, South Africa, ^13^University of Cape Town, Gugulethu HIV Programme and Desmond Tutu HIV Centre, Cape Town, South Africa

**Background:** Although patients initiating antiretroviral therapy (ART) should ideally have sustained engagement in care after ART start, it is increasingly recognized that a large proportion of patients experience care interruptions for a range of reasons. However, few studies have assessed the long‐term outcomes of children with a care interruption (CI), during which the child's health status and use of medication is unknown. We evaluated the characteristics and outcomes of HIV‐infected children that have care interruptions (i.e. >180 days without a clinic visit).

**Methods:** We included data on children <16 years old initiating antiretroviral therapy (ART) since 2004 at an International Epidemiologic Databases to Evaluate AIDS (IeDEA) Southern Africa (IeDEA‐SA) cohort with >180 days potential follow‐up. Children who died within 180 days of ART start were excluded. A CI was defined as a >180 days without a clinic visit and loss to follow‐up (LTFU) was defined as no visit within 180 days of database closure. The main outcome was all cause mortality. Two exposed groups were considered: those with a first CI within the first six months on ART, and those with a first CI after >6 months on ART. Adjusted rate ratios were determined using a Poisson regression model with robust standard errors.

**Results: ** Among 46,356 children included, 24,280 (52%) had a CI, of which 10,998 (45%) had a first CI within six months on ART and 13,282 (55%) had a first CI after six months on ART. Having a CI within the first six months on ART was associated with increased mortality (adjusted rate ratio (ARR) = 2.70, 95% CI 2.13 to 3.43), but there was no association between a first CI after six months on ART and mortality (ARR  = 1.01, 95% CI 0.77 to 1.31)

**Abstract THAC0305‐Table 1. Adjusted rate ratios for the effect of a care interruption on mortality using a Poisson model**

**Table:** 

Variable	ARR* (95% CI)	*p*‐value
Care Interruption status		
No Care Interruption	1.00	<0.001
Care Interruption before six months	2.70 (2.13 to 3.43)	
Care Interruption after six months	1.01 (0.77 to 1.31)	
Age at ART initiation		
<2 years	1.00	<0.001
2 to 5 years	0.98 (0.75 to 1.27)	
6 to 9 years	1.83 (1.26 to 2.66)	
≥10 years	4.18 (2.71 to 6.45)	

ARR adjusted for variables in the table, and also adjusted for gender, current age, year of ART initiation, time in/ out of care, CD4% at ART initiation.

**Conclusions: **The findings suggest that strengthening retention of children in care in the early period after ART initiation is critical to improving paediatric ART outcomes.

## THAC0401

### Rapid ART initiation and index client testing outcomes of commlink, a community‐based, HIV testing, mobile HIV care, and peer‐delivered, Linkage Case Management Program – Swaziland, 2017

**D. Williams^1^; D. Mackellar^1^; M. Dlamini^2^; N. Simelane^2^; S. Mlambo^2^; P. Mamba^2^; J. Byrd^3^; S. Mazibuko^4^; I. Pathmanathan^1^; N. Lukhele^5^; L. Dube^5^; M. Pasipamire^5^; V. Nxumalo^2^; A. Beyer^2^ and C. Ryan^4^**

^1^U.S. Centers for Disease Control and Prevention, Division of Global HIV/AIDS and Tuberculosis, Atlanta, United States, ^2^Population Services International Country Program, Mbabane, Swaziland, ^3^ICF International, Atlanta, United States, ^4^U.S. Centers for Disease Control and Prevention Country Office, Mbabane, Swaziland, ^5^Swaziland Ministry of Health, Swaziland National AIDS Programme, Mbabane, Swaziland

**Background:** Few persons diagnosed in community settings receive antiretroviral therapy within seven days of diagnosis (rapid ART) in accordance with World Health Organization recommendations. To improve rapid ART for clients diagnosed in community settings in Swaziland, we implemented CommLink, an integrated community‐based HIV testing (CBHTS), mobile HIV care, and peer‐delivered linkage case management (LCM) program.

**Description:** In urban and rural settings in three regions of Swaziland, consenting HIV‐positive, out‐of‐care clients identified through CBHTS receive mobile‐unit CD4 testing, baseline clinical assessment, and cotrimoxazole. Medical charts and test results are transferred to referral HIV care facilities. ART‐adherent, expert‐client counselors provide LCM for up to 90 days. Services include escort and treatment navigation at referral facilities, regular telephone contact for psychosocial support, and counseling on HIV care and disclosure. Beginning in April 2017, index‐client testing services (IDTS) were initiated for partners, family members, and associates (PFAs) of CommLink clients.

**Lessons learned:** From April through December 2017, of 498 eligible clients aged ≥15 years, 488 (98%) participated in LCM. Of 361 (74%) closed cases (completed program/discontinued participation/timed‐out), from the date of diagnosis, 351 (97%) clients enrolled in facility‐based care within a median (IQR) of two days (1 to 6) and 349 (97%) were initiated on ART within a median (IQR) of 2.5 days (1 to 6); 276 (76%) clients received rapid ART. At least 95% of clients in all gender and age groups were initiated on ART (Table). During LCM, 94% of clients disclosed their HIV status to at least one partner or family member, and 42% had at least one PFA tested. Of 209 PFAs tested, 128 (61%) tested HIV positive (40% of 128 newly diagnosed), and 117 subsequently participated in CommLink. Disclosure of HIV status and participation in IDTS was similar across demographic groups.

**Conclusions/Next steps:** CommLink achieved near‐universal early and >70% rapid ART initiation among all persons diagnosed in community settings in Swaziland, including men and young adults. As an integral component of LCM, index client testing was pivotal for active case finding. In 2018, CommLink has been approved by the Swaziland Ministry of Health to pilot community‐based point‐of‐diagnosis ART initiation and is being scaled‐up nationally.

**Abstract THAC0401‐Table 1. CommLink Enrollment in Care, ART Initiation, and Index Client Testing Outcomes**

**Table:** 

	CommLink Enrollment in Care, ART Initiation, Disclosure, and Partner, Family Member, and Associate (PFA) Testing Outcomes						Index Client Testing Outcomes	
	Clients	Enrolled in HIV Care	Initiated on ART	Rapid ART Initiation	Disclosed HIV status to ≥1 PF	≥1 PFA Tested	PFA Tested	PFA HIV+
	n	n (%)	n (%)	n (%)	n (%)	n (%)	n	(%)
All	361	351 (97)	349 (97)	276 (76)	340 (94)	153 (42)	209	128 (61)
Sex Male	166	159 (96)	157 (95)	117 (70)	152 (92)	63 (38)	104	56 (54)
Female	195	192 (98)	192 (98)	159 (82)	188 (96)	90 (46)	105	72 (69)
Age <15	–	–	–	–	–	–	20	6 (30)
15 to 24	68	67 (99)	67 (99)	56 (82)	66 (97)	33 (49)	35	26 (74)
25 to 34	153	149 (97)	148 (97)	117 (76)	144 (94)	57 (37)	85	59 (69)
>34	140	135 (96)	134 (96)	103 (74)	130 (93)	63 (45)	69	37 (54)

## THAC0402

### Community‐based HIV testing and assessment for same‐day ART reaches men for HIV care

**S. Asiimwe^1^; H. van Rooyen^2^; T. Schaafsma^3^; A. van Heerden^2^; B. Turyamureeba^1^; M. Krows^3^; A. Szpiro^4^; J. Baeten^3^; C. Celum^3^; R. Barnabas^3^ and The Delivery Optimization of Antiretroviral Therapy (DO ART) Study**

^1^Integrated Community Based Initiatives, Kabwohe, Uganda, ^2^Human Sciences Research Council, Sweetwaters, South Africa, ^3^University of Washington, Department of Global Health, International Clinical Research Center, Seattle, United States, ^4^University of Washington, Biostatistics, Seattle, United States

**Background:** Integrating same‐day ART start into community‐based HIV testing has the potential to increase ART coverage, particularly among hard to reach priority populations, such as men. The coverage and feasibility of community‐based same‐day ART initiation requires evaluation.

**Methods:** The Delivery Optimization for ART (DO ART) Study is an ongoing randomized study of community‐based compared to clinic‐based ART initiation and monitoring in the Sheema District, Uganda and KwaZulu Natal, South Africa. Lay counselors conduct community‐based HIV testing and counseling at home or through a mobile van. To reach men, testing was conducted at trading posts and in the evenings. HIV‐positive persons are assessed for same‐day ART by a clinical questionnaire and point‐of‐care testing for CD4 count, pregnancy, and creatinine. Nurses received standardized government training on ART initiation and HIV care, and assess ART eligibility. Participants are eligible for same‐day ART start if they are clinically stable (have a CD4 count >100 cell/mL and no evidence of current or past WHO stage 3 or 4 HIV conditions), have no symptoms of active TB, are not pregnant, and have normal renal function. Eligibility results are presented for Uganda, where enrollment is complete.

**Results: ** Between June 2016 and November 2017, 398 HIV‐positive persons were identified by community‐based HIV testing in Uganda, of whom 320 (80%) were eligible for same‐day ART start. Among the 98 participants who were not eligible for same‐day ART start, the leading reasons were CD4 count >500 cells/mL (46%, who were not eligible by the Ugandan national guidelines for ART initiation at the time), CD4 count <100 cells/mL (17%), any of four symptoms suggestive of TB (14%), and a positive pregnancy test (9%). One participant had a creatinine >1.5 mg/dL and was not eligible. Of those eligible for same‐day ART start, 169 (53%) were men.

**Conclusions: **Among HIV‐positive persons identified through community‐based HIV testing in a rural setting in Uganda, 80% were eligible for same‐day ART start. Notably, men accounted for more than half the persons eligible for same‐day ART start. Community‐based HIV testing and counseling and same‐day ART start has the potential to increase coverage of ART among priority populations.

## THAC0403

### Same‐day ART initiation in HIV/STI testing center in Bangkok, Thailand: initial results from an implementation research

**P. Seekaew^1^; N. Teeratakulpisarn^1^; P. Surapuchong^1^; S. Teeratakulpisarn^1^; S. Amatavete^1^; P. Jomja^1^; C. Prabjunteuk^1^; C. Hanaree^1^; W. Chaison^1^; P. Plodgratoke^1^; K. Singhaseni^1^; D. Lingjongrat^2^; S. Janyam^3^; P. Na Nakorn^1^; S. Charoenying^4^; S. Mills^4^; R. Vannakit^5^; P. Phanuphak^1^ and N. Phanuphak^1^**

^1^Thai Red Cross AIDS Research Centre, Prevention, Bangkok, Thailand, ^2^Rainbow Sky Association of Thailand, Bangkok, Thailand, ^3^Service Workers in Group Foundation, Bangkok, Thailand, ^4^FHI 360 and USAID LINKAGES Project, Bangkok, Thailand, ^5^Office of Public Health, U.S. Agency for International Development Regional Development Mission Asia, Bangkok, Thailand

**Background:** Despite WHO's recommendation on Universal ART, one‐third of persons living with HIV in Thailand are without antiretroviral therapy (ART). Attrition from care due to delays in initiating treatment may contribute to this gap. There is strong evidence to suggest clinical and practical benefits of rapid ART initiation, including Same‐Day ART. However, data on safety and feasibility in real‐world settings is limited.

**Methods:** Data was collected among HIV‐seropositive Thai clients at Thai Red Cross Anonymous Clinic in Bangkok, Thailand. Clients were categorized as newly diagnosed or reengaged. Assessment of acceptability, logistical eligibility (no previous ART and could return for follow‐up visits), and first date of known HIV‐seropositive status were self‐reported. Baseline laboratory tests (creatinine/ALT/syphilis/HBsAg/anti‐HCV/CD4/CrAg if CD4 < 100) and chest x‐ray were performed according to national guidelines. Physicians determined clinical eligibility of Same‐Day ART based on signs/symptoms and only results of CD4 and chest x‐ray to rule out tuberculosis, cryptococcal meningitis, and serious illnesses. Mean days from HIV diagnosis/reengagement to ART initiation were calculated. Referral rate to long‐term ART continuation site, after 10 weeks of ART, was also determined.

**Results: ** From July‐December 2017, 86.8% of 1062 HIV‐seropositive clients were logistically eligible for Same‐Day ART service and 90% (95% newly diagnosed; 85% reengaged) accepted the service. Median (IQR) CD4 was 295.5 (203 to 415) cells/mm^3^. 82% of those who accepted the service were clinically eligible and initiated ART. Among these, 79% received ART on the day of HIV diagnosis/reengagement. Mean (SD) days from HIV diagnosis/reengagement to ART initiation was 1.4 (5.2) days for newly diagnosed clients and was 1.1 (4.0) days for reengaged clients. Immune reconstitution inflammatory syndrome was not seen. Referral to long‐term ART site was successful among 88.2%. Virologic suppression was achieved by 100% of 12 clients who reached month six after ART.

**Conclusions: **Same‐Day ART initiation in an HIV/STI testing center in Thailand is highly feasible and safe, with high rates of client eligibility and acceptability. Preliminary data on referral to long‐term ART site and short‐term virologic suppression are encouraging. Same‐Day ART should be immediately scaled‐up, with rigorous and longitudinal evaluations to further inform HIV guidelines.

## THAC0404

### Fast‐track ART initiation in Botswana is associated with high rates of ART initiation, retention in care, and virological suppression

**R. Lebelonyane^1^; P. Bachanas^2^; W. Abrams^3^; M. Roland^3^; J. Theu^1^; M. Kapanda^1^; S. Matambo^3^; S. Lockman^4^; J. Moore^2^; L. Block^5^; T. Gaolathe^6^; J. Makhema^6^ and J.N. Jarvis^6,7^**

^1^Botswana Ministry of Health and Wellness, Gaborone, Botswana, ^2^Centers for Disease Control and Prevention, Division of Global HIV/AIDS and TB, Atlanta, United States, ^3^Centers for Disease Control and Prevention, Gaborone, Botswana, ^4^Harvard T.H. Chan School of Public Health, Boston, United States, ^5^Intellectual Concepts, Atlanta, United States, ^6^Botswana Harvard Partnership, Gaborone, Botswana, ^7^Botswana‐UPenn Partnership, Gaborone, Botswana

**Background:** The Botswana Combination Prevention Project (BCPP) started in 2013 and has offered “fast‐track” ART initiation at the first clinic visit since June 2016. We examined treatment outcomes pre‐ and post‐ fast‐track ART initiation.

**Methods:** BCPP is a cluster‐randomized trial evaluating a combination HIV prevention package in 15 intervention and 15 control communities. In the intervention communities, we compared the cumulative proportion of individuals initiating ART (using Kaplan Meier estimates), as well as retention in care and viral suppression in patients, comparing outcomes in two periods: following the introduction of fast‐track ART (June 2016‐November 2017), versus pre‐ fast track ART initiation (October 2013‐May 2016).

**Results: ** Overall 3622 HIV‐infected individuals not on ART were identified through community testing activities and referred for treatment, and 3315 (92%) linked to care. At data censoring in November 2017, 91% (2682/2943) of linked, eligible individuals had initiated ART. The cumulative probability of initiating ART within one year of linkage was 84% and 87% in pre‐ and post‐fast track groups respectively. ART initiations occurred more quickly after implementation of fast‐track ART with 63% (626/990) initiating ART within seven days of linkage and 73% (723/990) initiating within 30 days, compared to 12% (237/1953) and 44% (851/1953) prior to fast‐track. Retention in care after six months on ART was 93% in both groups. However, viral suppression rates within the first year of ART were higher in the fast‐track group; 82% of those on ART for at least six months (510/621) had a viral load (VL) performed of whom 97% (494/510) were suppressed. In the pre fast‐track group, 80% (1415/1784) had a VL performed of whom 93% (1318/1415) were suppressed (*p* = 0.04). Median time from linkage to first viral suppression was significantly shorter following introduction of fast track ART (108 days vs. 288 days, *p* < 0.001).

**Conclusions: **ART initiation, retention in care and viral suppression rates were high in HIV‐infected individuals who initiated fast track ART. Time from linkage to viral suppression was significantly shorter with fast‐track ART, reducing the period of potential HIV‐transmission risk. These data support programmatic ART initiation efforts designed at starting ART quickly in stable patients.

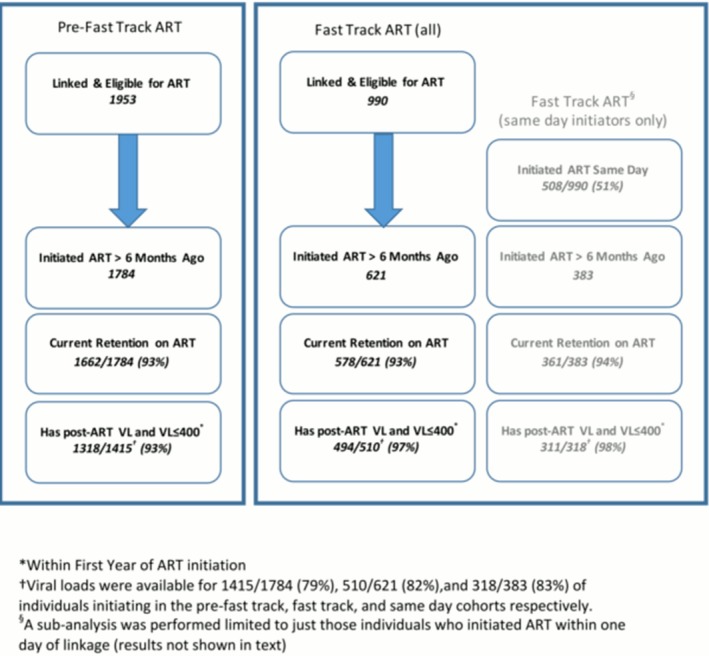

**Abstract THAC0404‐Figure 1. Retention in Care and Viral Suppression in the Botswana Combination Prevention Project.**

## THAC0405

### Towards the third 90: factors associated with adolescent antiretroviral adherence and viral suppression

**P. Wekesa^1^; L. Nyabiage^2^; K. Owuor^1^; J. Kataka^1^; J. Oliech^1^; A. Bisera^1^; E. Ouma^1^ and S. Omondi^3^**

^1^Centre for Health Solutions – Kenya (CHS), Nairobi, Kenya, ^2^Division of Global HIV & TB (DGHT), US Centers for Disease Control and Prevention (CDC), Nairobi, Kenya, ^3^Ministry of Health, County Department of Health, Siaya, Kenya

**Background:** Globally, adolescents have lower rates of adherence to antiretroviral therapy (ART) and viral suppression (VS) compared to adults and children. We sought to understand predictors of adherence and VS among adolescents in Siaya County, western Kenya.

**Methods:** We conducted a retrospective analysis of patient files for adolescents aged 10 to 19 years initiated on ART between 2000 and 2017 at 119 sites. Outcomes were self‐reported adherence (SRA) to ART and VS (<1000 copies/mL) based on the latest 2017 VL results. Adherence was based on missed doses per month (good <2, fair 2 to 4, poor >4). Variables were: age, sex, ART regimen, VS, SRA, school attendance, and frequency of clinic visits. Caregiver variables included HIV status, VS, SRA, stable caregiver and support group (SG) attendance within the last three months. Descriptive, bivariate and multivariable binary logistic regression analyses were done. Unadjusted and adjusted odds ratios (OR) and 95% confidence intervals (CI) were calculated to assess associations at the 5% level

**Results: ** Records of 2814 adolescents were included in the analysis; of these 1389 (49%) were female and the mean age was 13.3 years (SD ±2.7). Good adherence was reported among 2192 (78.8%), while 389 (14%) had fair and 201 (7.2%) poor adherence. In total, 1615 (61.6%) achieved VS. Among caregivers 1568 (55.7%) attended SG, 1440 (51%) had HIV, 1425 (99%) were on ART, and of these, 1087 (88%) reported good adherence. Factors associated with good SRA were: caregiver good SRA (aOR = 9.05 (95% CI: 4.96 to 16.52)), caregiver VS (aOR = 11.11 (95% CI 8.33 to 14.28)), caregiver attendance of SG (aOR = 1.38 (95% CI: 0.97 to 1.98)) and clinic visit intervals of two months or more (aOR = 3.76 (95% CI: 1.72 to 8.22)). Viral suppression was associated with good SRA (aOR = 11.34 (95% CI: 8.56 to 15.01)), clinic visit intervals of two months or more (aOR = 2.82 (95% CI: 1.75 to 4.53)) and Tenofovir‐based ART regimen versus an Abacavir (aOR = 0.64 (95% CI: 0.48 to 0.85)) or Zidovudine (aOR = 0.65 (95% CI: 0.5 to 0.86))‐based regimen.

**Conclusions: **Caregiver factors were associated with good SRA to ART among adolescents. Regimen type was a significant predictor of VS. The potential role of these factors in improving adolescent adherence to ART and VS should be explored.

## THAC0501

### The post‐intervention effects of conditional cash transfers for HIV/STI prevention: a randomized trial in rural Tanzania

**D. de Walque^1^; W.H. Dow^2^; R. Nathan^3^ and The RESPECT Study Team**

^1^The World Bank, Development Research Group, Washington, United States, ^2^University of California, School of Public Health, Berkeley, United States, ^3^Ifakara Health Institute, Dar es Salaam, Tanzania, United Republic of

**Background:** Incentive‐based policies have been shown to be powerful in many areas of behavior, but have rarely been tested in the sexual domain. The Rewarding Sexually Transmitted Infection Prevention and Control in Tanzania (RESPECT) study is a randomized controlled trial testing the hypothesis that a system of rapid feedback and positive reinforcement that uses cash as the primary incentive can be used to reduce risky sexual activity among young people, male and female, who are at high risk of HIV infection. Recognizing that such an intervention would be difficult to sustain over the length of individuals’ sexual lives, we evaluated its long‐term effects using a post‐intervention follow‐up survey conducted one year after discontinuing the intervention.

**Methods:** The study enrolled 2399 participants in 10 villages in rural southwest Tanzania. The intervention arm received conditional cash transfers ($10 or $20 every four months) that depended on negative results of periodic screenings for sexually transmitted infections, an objectively measured marker for risky sexual behavior. One year after discontinuing the CCT intervention, at month 24, we revisited the 10 study villages and retested and re‐interviewed study participants to assess the long‐term impacts of the intervention.

**Results: ** Overall, the CCT interventions seemed to have had a sustained impact in reducing the STI prevalence among the study population: when we combine all 7 STIs tested, both the high and the low value CCT intervention have relative risks significantly lower than 1, corresponding to 18 (*p* < 0.1) to 20% (*p* < 0.01) risk reduction compared to the control group, respectively. These effects are stronger among males than among females.

**Conclusions: **Those results from the one‐year post intervention follow‐up indicate that the CCT interventions might have sustained effect even after the cash payments have been discontinued and suggest a learning effect. They do not suggest that CCTs might destroy the intrinsic motivation to adopt safe sexual practices since no increased risk was reported in the intervention groups. Those are important results when considering the potential feasibility at scale and sustainability of our CCT intervention.

## THAC0502

### Changes, patterns and predictors of sexually transmitted infections in gay and bisexual men using PrEP; interim analysis from the PrEPX demonstration study

**M. Traeger^1^; J. Asselin^1^; B. Price^2^; V. Cornelisse^3,4^; N. Roth^4^; J. Wilcox^5^; B.K. Tee^6^; C. Fairley^3^; C. Chang^2^; J. Armishaw^2^; O. Vujovic^2^; M. Penn^7^; P. Cundill^7^; G. Forgan‐Smith^8^; J. Gall^8^; C. Pickett^9^; L. Lal^2^; A. Mak^2^; T. Spelman^1,10,11^; L. Nguyen^1^; D. Murphy^12^; K. Ryan^1^; C. El‐Hayek^1^; S. Ruth^13^; C. Batrouney^13^; J. Lockwood^2^; J. Hoy^10^; R.M. Grant^14^; M. Hellard^1,2,10^; M. Stoové^1,10^; E. Wright^1,2,10^ and PrEPX Study Team**

^1^Burnet Institute, Disease Elimination Program, Melbourne, Australia, ^2^Alfred Health, Melbourne, Australia, ^3^Melbourne Sexual Health Centre, Melbourne, Australia, ^4^Prahran Market Clinic, Melbourne, Australia, ^5^Northside Clinic, Melbourne, Australia, ^6^Centre Clinic, Melbourne, Australia, ^7^PRONTO! clinic, Melbourne, Australia, ^8^ERA Health, Melbourne, Australia, ^9^Ballarat Community Health Centre, Ballarat, Australia, ^10^Monash University, School of Public Health and Preventative Medicine, Melbourne, Australia, ^11^Peter Doherty Institute for Infection and Immunity, Melbourne, Australia, ^12^University of New South Wales, Centre for Social Research in Health, Sydney, Australia, ^13^Victorian AIDS Council, Melbourne, Australia, ^14^Gladstone Institute for Virology and Immunology, San Francisco, United States

**Background:** While there is growing evidence of increasing sexually transmitted infections (STIs) among gay and bisexual men (GBM) using PrEP, few studies have been able to measure STI incidence before PrEP commencement. We compare STI incidence among GBM before and after enrolment in the Pre‐exposure Prophylaxis Expanded (PrEPX) study, a population‐level, multi‐site, PrEP implementation project in Melbourne, Australia, and predictors of STI diagnoses while taking PrEP.

**Methods:** STI testing and behavioural data from PrEPX participants attending five clinics specializing in GBM health were extracted prior to study enrolment and at scheduled three‐monthly PrEP visits between July 2016‐December 2017 through the Australian Collaboration for Co‐ordinated Enhanced Sentinel Surveillance (ACCESS). We calculated participants’ gonorrhea, chlamydia and syphilis incidence in the year before PrEPX enrolment and during PrEPX follow‐up. Incidence comparisons were calculated overall and by participants’ pre‐enrolment testing frequency and self‐reported pre‐study PrEP use. Kaplan‐Meier estimating methods and Cox proportional hazards regression explored associations between baseline and longitudinal behaviours and STI diagnosis after PrEP commencement.

**Results: ** 2490 participants contributed 1040.3 person‐years (PY) pre‐PrEPX and 1899.7 PY during PrEPX. During PrEPX, 41% of participants were diagnosed with ≥1 STI and 455 (18%) participants with multiple STIs accounted for 68% of all infections. Rectal infections were commonest (62.4/100PY). STI incidence among all participants increased significantly from 78.4/100PY pre‐PrEPX to 96.1/100PY during PrEPX. Significantly elevated STI incidence was detected among participants with ≥3 test visits in the year preceding enrolment, but not among participants with ≥4 visits. STI incidence increased most among participants reporting no pre‐study PrEP use. STI diagnosis and condomless receptive anal intercourse prior to enrolment were associated with increased STI risk during PrEPX (*p* < 0.001). Younger age (aHR = 1.02/year, 95% CI = 1.01 to 1.03), >10 casual anal sex partners (aHR = 1.76, 95% CI = 1.30 to 2.39) and group sex (aHR = 1.49, 95% CI = 1.10 to 2.03) in the past six months were associated with increased STI risk.

**Conclusions: **STI incidence increased among GBM in PrEPX following PrEP initiation, driven largely by GBM experiencing repeat infections. High partner turnover and group sex elevated STI risk. Our findings support ongoing and frequent STI screening alongside education on early identification of STI symptoms for PrEP users, especially among those exhibiting multiple STIs and high‐risk behaviours.

**Abstract THAC0502‐Table 1. Incidence of chlamydia, gonorrhea and syphilis among gay and bisexual men before and after enrolling in the PrEPX study. IRR = Incidence Rate Ratio**

**Table:** 

	1 Year Before Enrolment		During Follow‐up			
	Median Follow‐up (months)	Incidence Rate (per 100 person‐years)	Median Follow‐up (months)	Incidence Rate (per 100 person‐years)	IRR (95% CI)	*p*‐value
All Participants (n = 2490)	11.9	78.4	9.9	96.1	1.23 (1.13 to 1.33)	<0.001
GBM with ≥2 visits in the 12 months before enrolment (n = 1003)	12.0	93.6	10.2	113.2	1.21 (1.10 to 1.33)	<0.001
GBM with ≥3 visits in the 12 months before enrolment (n = 692)	12.0	106.5	10.3	120.7	1.13 (1.02 to 1.26)	0.022
GBM with ≥4 visits in the 12 months before enrolment (n = 447)	12.0	122.6	10.6	135.1	1.10 (0.97 to 1.25)	0.122
GBM reporting ever using PrEP before enrolment (n = 649)	11.9	99.0	10.7	108.0	1.09 (0.97 to 1.23)	0.156
GBM reporting never using PrEP before enrolment (n = 1841)	11.8	63.7	9.5	90.9	1.43 (1.28 to 1.60)	<0.001

## THAC0503

### How can programmes better support female sex workers to avoid HIV infection in Zimbabwe? A prevention cascade analysis

**E. Fearon^1^; A. Phillips^2^; S. Mtetwa^3^; S. Chabata^3^; P. Mushati^3^; V. Cambiano^2^; J. Busza^1^; S. Napierala^4^; B. Hensen^5^; S. Baral^6^; S.S. Weir^7^; B. Rice^1^; F.M. Cowan^3,8^ and J.R. Hargreaves^1^**

^1^London School of Hygiene and Tropical Medicine, Department of Public Health, Environments and Society, London, United Kingdom, ^2^University College London, Institute for Global Health, London, United Kingdom, ^3^Centre for Sexual Health and HIV AIDS Research Zimbabwe, Harare, Zimbabwe, ^4^RTI International, Women's Global Health Imperative, San Francisco, United States, ^5^London School of Hygiene and Tropical Medicine, Department of Clinical Research, London, United Kingdom, ^6^Johns Hopkins University, Bloomberg School of Public Health, Baltimore, United States, ^7^University of North Carolina at Chapel Hill, Department of Epidemiology, Chapel Hill, United States, ^8^Liverpool School of Tropical Medicine, Department of International Public Health, Liverpool, United Kingdom

**Background:** The “HIV prevention cascade” has been proposed as a tool to measure prevention coverage and guide programming by identifying gaps in demand, supply and capability to adhere to HIV prevention tools. Here, we use a prevention cascade framework to explore coverage of condom use and Pre‐Exposure Prophylaxis (PrEP) among female sex workers (FSW) in Zimbabwe, and make recommendations for programming.

**Methods:** We conducted secondary analysis of seven respondent‐driven sampling (RDS) surveys from the intervention sites of a cluster‐randomised trial in Zimbabwe in 2016. Women were tested for HIV, and completed a questionnaire detailing their socio‐demographic and sex work characteristics, their experience of PrEP (available during the trial 2014 to 2016) and reported condom use with clients and partners. We operationalised measures of “demand,” “supply” and “adherence” to using condoms and PrEP. We used logistic regression to identify determinants of adherence. Differences were examined across sites and data then pooled. We weighted by site‐normalised inverse degree and dropped seeds.

**Results: ** There were 611 HIV‐negative women included in our analysis. Approximately half of these women (54.7%) reported adherence to condoms and/or to PrEP. While women knew that condoms prevented HIV and reported good access (both 94.0%), only 45·5% reported no episodes of condomless sex in the previous month. For PrEP, there were gaps across all three domains of demand, supply and adherence (Figure 1). Alcohol use of women and of their clients was associated with lower condom adherence (among women drinking alcohol 4 +  times per week, 38/115 28.9% adhered, compared to no alcohol, among whom 139/262 50.9% adhered, aOR = 0.34 95% CI 0.08 to 0.41, adjusted for socio‐demographic and sex work characteristics). Newer entrants to sex work, and younger women were less likely to report taking PrEP every day (aOR = 1.05, 95% CI 1.01 to 1.10 for each year of age).

**Conclusions: **After 21 months of intensive community mobilisation for PrEP and condoms during the trial intervention, almost half of HIV‐negative FSW reported inadequate prevention coverage, worse in the context of heavy alcohol use, which programmes could address. Interventions aimed at younger women are important. HIV prevention cascades should consider different prevention tools together, not in isolation.

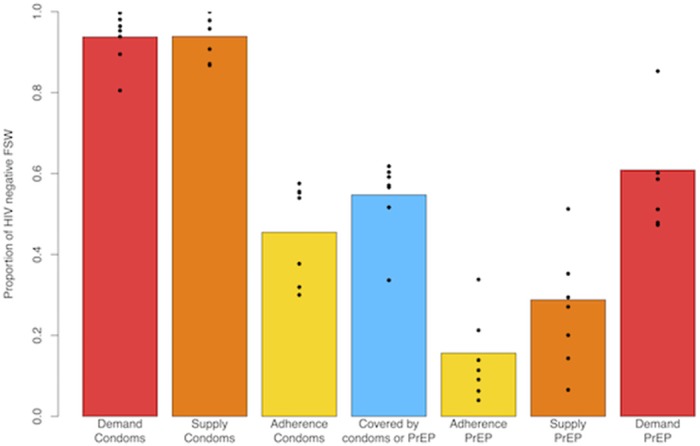

**Abstract THAC0503‐Figure 1. Demand, Supply, Adherence and Coverage by Condoms and/or PrEP amongst 611 HIV‐negative FSW from seven sites.**

## THAC0504

### HIV “condom cascade” to monitor prevention among female sex workers in Uganda

**S. Weir^1^; F. Ssengooba^2^; L. Ayutambe^3^; S. Kasasa^2^ and G. Mulholland^1^**

^1^University of North Carolina, Epidemiology, School of Public Health, Chapel Hill, United States, ^2^Makerere University, School of Public Health, Kampala, Uganda, ^3^Makerere University, Kampala, Uganda

**Background:** Recently there has been interest in HIV prevention cascades. We applied a proposed prevention cascade framework (*Garnett, 2016, Lancet*) to data collected for a PLACE mapping and biobehavioral survey in 30 districts in Uganda.

**Description:** The client‐centric and program centric cascades were measured for condom use among 9207 HIV‐negative female sex workers from 30 districts. The client centric cascade showed that over half of the female sex workers did not perceive themselves to be at risk for HIV acquisition. Most of those who perceived themselves at risk had ever used condoms but only 64 of the 9207 female sex workers reported consistent use. The program centric prevention cascade found that only two‐thirds of the female sex workers who are at risk have access to condoms. But of those who have ever used condoms, consistent use is very poor. Estimating the condom cascade led us to estimate the number of condoms that would be required per month in order to protect female sex workers. We estimated that 110,889 condoms would be required to for 10,417 female sex workers. We estimated that 1677 acts in the past four weeks were among HIV infected sex workers who did not use a condom. Anal sex was also often not protected.

**Lessons learned:** Both the program centric and the client centric cascades provided compelling evidence of the lack of availability of condoms, the poor uptake of condoms, and the almost negligible consistent use of condoms in this population. It is striking that 4736 of 9207 female sex workers did not perceive they were at risk for HIV acquisition. It is also striking that 2853 female sex workers reported that condoms were not readily available to them. The cascade helped document the condom programming needs for this key population.

**Conclusions/Next steps:** These findings have already been used to advocate to increase cthe accessibility of condoms to those who most need it. Additionally, there is interest in developing HIV prevention cascades at the venue level to characterize venues that have been adequately reached by prevention programs and those where there remain gaps.

## THAC0505

### Making markets work for HIV prevention: a total market approach to condom security in Vietnam

**Y. Vu Yen; Kim; Bao; Trang/USAID; Chieu/VAAC; Minh/CCIHP; Trang/CCIHP; Tung/Lighthouse; Thanh/G‐Link; Son/G3VN and Song deyeu**

PATH, USAID‐PATH/Healthy Markets, Hanoi, Vietnam

**Background:** Donor‐funded free and partially subsidized condoms for key populations (KPs) artificially suppressed a domestic commercial condom market in Vietnam. With significant declines in free/socially marketed condoms from 2014 on, market management was needed to ensure a sustained supply of affordable commercial condoms capable of meeting the needs of populations at‐risk of HIV.

**Description:** From 2015 on, the USAID/PATH Healthy Markets project initiated a condom total market approach (TMA) effort:

(1) generating condom market volume, sales and growth projections that were used to engage local condoms manufacturers and encourage local production of quality, low‐cost condoms that appealed to KP; identify new distribution channels; and increase demand among key populations;

(2) developing a sustainable KP‐focused condom value chain, bringing together the private sector, social enterprises, and KP‐led community‐based organizations (CBOs); and

(3) working with the Ministry of Health to use market data to shape annual condom planning and budgeting. As a result, four KP‐market segmented brands were developed, and commercial condom sales volume in KP hotspots/outlets increased 400% from 5,125,984 in 2015 to 22,823,447 in 2017. Commercial condom market‐share in KP‐preferred outlets, i.e. hotels and guesthouses, significantly increased from 25% in 2013 to 74% in 2016.

**Lessons learned:** Key lessons learned include:

(1) market and consumer insights and brokering relationships with new distributors and retailers were invaluable to engaging and securing commitment from local condom manufacturers to invest in the local market;

(2) increasing the capabilities of KP‐led CBOs and social enterprises to engage in commercial condom sales was essential to developing a sustainable KP‐targeted market;

(3) partnering closely with the MOH from the start increased their ownership and stewardship of more nuanced condom commodity planning.

**Conclusions/Next steps:** The TMA has been a driving force for growing a sustainable condom market that meets the needs of KP in Vietnam. The TMA needs to be extended to other HIV‐commodities, and the MOH will need to take an increasing role as a market manager, not only for condoms, but also for needles and syringes and other critical HIV‐related commodities, as donor funds further decline and domestic financing of all HIV‐commodities must be secured.

## THAD0101

### Police related correlates of client violence among female sex workers in a U.S. city

**K.H.A. Footer^1^; J. Park^2^; S.T. Allen^1^; M.R. Decker^3^; B.E. Silberzahn^1^; S. Huettner^4^; N. Galai^2^; S.G. Sherman^1^ and the SAPPHIRE Study**

^1^Johns Hopkins Bloomberg School of Public Health, Health Behavior and Society, Baltimore, United States, ^2^Johns Hopkins Bloomberg School of Public Health, Epidemiology, Baltimore, United States, ^3^Johns Hopkins Bloomberg School of Public Health, Population, Family and Reproductive Health, Baltimore, United States, ^4^Johns Hopkins School of Medicine, Baltimore, United States

**Background:** Globally, rates of HIV among female sex workers (FSW) remain high. A more nuanced understanding of the role of structural factors is of paramount importance. Law enforcement practices have been identified as an important factor in the HIV risk environment of FSW. This study looks to characterize the frequency and type of interactions that FSW have with the police and explore the implications of cumulative police exposures on experiences of client violence.

**Methods:** Cross‐sectional baseline data examined from a cohort study of 250 street‐based FSW recruited between April 2016 and January 2017 using targeted sampling in Baltimore City, Maryland, U.S. Questionnaires captured a range of patrol/enforcement (e.g. routine stops) and abusive (e.g. verbal/sexual harassment) police encounters, experiences of client violence and other HIV risk factors, including drug use. All women were tested for HIV. Pearson's chi‐square tests and logistic regression models were used to test frequency and type of police interactions experienced by FSW, and the association between police interactions and client violence, accounting for daily heroin use.

**Results: ** 78% of participants (n = 195) reported lifetime abusive police encounters, 41% reported daily/weekly encounters of any type. 22% of participants experienced client violence in the prior three months. FSW that reported heroin use (70%; n = 175) reported more abusive encounters (2.5 vs. 1.6, *p* < 0.001) and more client violence (26% vs. 12%, *p* = 0.02) than their non‐heroin using counterparts. In multivariable analysis, each additional type of abusive interaction was associated with 1.3 times (95% CI: 1.1 to 1.5) increased odds of client violence. For patrol/enforcement encounters this value was 1.3 (95% CI: 1.0 to 1.7).

**Conclusions: **Our findings point to high levels of routine and abusive police exposure experienced by FSW contributing to a layering of risk that promotes an environment in which FSW are at a heightened risk of client perpetrated violence. For FSW who inject drugs, risk of both police exposure and client violence appear amplified. Our results demonstrate the need for greater attention to better understanding the nature and impact of these forms of intersectional risk on FSW HIV risk environment. Structural interventions that seek to address police‐FSW interactions and promote FSW safety are critical to alleviating police's impact on FSW health.

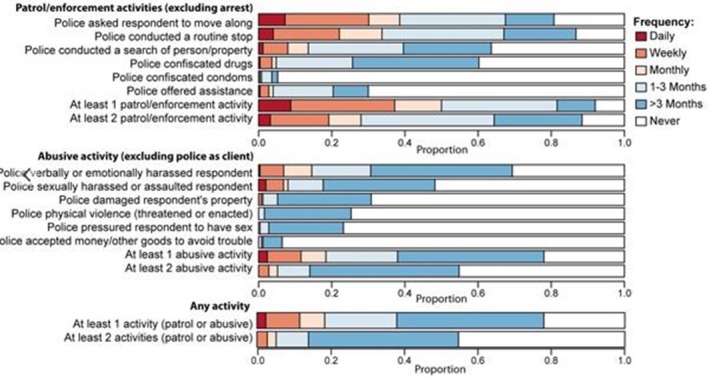

**Abstract THAD0101‐Figure 1.**

## THAD0102

### “They should protect us because that is their job”: an assessment of sex workers’ experiences with police abuse in Lusaka, Zambia

**T.K. Esterhuizen**

Southern Africa Litigation Centre (SALC), LGBTI and Sex workers Rights Programme, Johannesburg, South Africa

**Background:** In Lusaka, police officials often perpetrate violence and abuse against sex workers and target them with the arbitrary and subjective enforcement of overly broad laws. This abuse increases the vulnerability of sex workers pushes them away from important health services and limits their access to information about their sexual and reproductive health needs. This study assesses the challenges faced by Lusaka's sex workers. It is aimed at developing advocacy and litigation strategies to address the systemic abuse of sex workers by the police in Lusaka. It also highlights mechanisms that activists and lawyers can use to hold officials accountable.

**Description:** This study is based on a combination of desk research and limited qualitative research. The desk research focused on the legal framework within which the police interact with sex workers in Zambia. In addition to this, focus group discussions were held with 39 self‐identified female sex works (FSW) in two local languages, Nyanja and Bemba. The FSW, aged between twenty and forty, represented five different geographic districts across Lusaka. The purpose of the focus group discussions was to develop an understanding of the relationship between sex workers and police authorities.

**Lessons learned:** While the act of selling sex for reward is not criminalised in Zambia, police officials often use vagrancy and nuisance laws to arbitrarily arrest and detain sex workers. These offences are overbroad and subjectively applied, which creates a culture of impunity – in which both the clients and police officials can abuse sex workers without consequences. Often, police officials request sex from sex workers in exchange for not arresting, detaining, or fining them. Eighty seven percent (87%) of participants in the study reported that the police have harassed them because they engage in sex work. Sex workers’ experiences with police affect their willingness to open cases at the police station, and therefore, often directly affect the extent to which they can access healthcare services post‐rape, including post‐exposure prophylaxis and emergency contraception.

**Conclusions/Next steps:** In order to protect the fundamental rights and dignity of sex workers, the Zambian Penal Code must be revised to repeal offences that are vague and overly broad.

## THAD0103

### Violence towards sex workers in the Netherlands

**M. Kloek^1^; M. Dijkstra^1^; M. Jonker^1^; D. Bons^2^; L. Dekker^1^; S. Hendriks^1^ and Study Group Consisting of Research Assistents from Sex Worker Led Organisation**

^1^Aids Fonds, Sex Work Program, Amsterdam, Netherlands, ^2^PROUD, Dutch Union for Sex Workers, Amsterdam, Netherlands

**Background:** Violence is one of the most important factors affecting the risk for sex workers around the world to HIV/AIDS. Due to their position in society and criminalising laws, sex workers are at risk for physical, sexual, economic and emotional violence from clients, co‐workers, managers, institutions and others. This may cause inconsistent condom use and prevents sex workers globally from accessing necessary support and health care. This study assesses the current state of violence among sex workers in the Netherlands.

**Methods:** In this mixed methods study (including surveys, in‐depth interviews and FGDs) sex workers were trained as research assistants and were part of the design and roll‐out of the study. A very diverse group of 308 sex workers from all over the Netherlands was included.

**Results: ** Overall violence experienced by sex workers in the Netherlands is high (physical violence 60%, sexual violence 78%, economic violence 58%, emotional violence 93%). Logistic regression analysis shows among others that male sex workers have 2.3 (*p* < 0.05) more odds for sexual violence and transgender sex workers have 4.9 (*p* < 0.05) more odds for emotional violence compared to their female peers. Young sex workers were found to have (e.g. 2.4 (*p* < 0.05) more odds for sexual violence). Sex workers who do not work in a licenced venue have 3.2 (*p* < 0.0001) more odds for sexual violence. Sex workers who work at massage salons have 8.5 (*p* < 0.01) more odds for sexual violence where as sex workers who work in hotel rooms have 4.6 (*p* < 0.05) more odds for emotional violence. Working in a window venue is a strong protective factor (3.1 (*p* < 0.05) less odds for sexual violence). Despite high levels of violence experienced by sex workers in this study most sex workers (79%) did not report any incident of violence to the police in the last 12 months.

**Conclusions: **Even though sex work is legal in the Netherlands, sex workers experience high levels of violence. Most sex workers do not report incidents to the police, which leaves them at higher risk. Decriminalization of sex work is necessary to increase access to justice for sex workers and reduce violence.

## THAD0104

### The impact of end‐demand legislation on sex workers’ utilization of HIV care, health and community‐led support services in a Canadian setting

**E. Argento^1,2^; S.M. Goldenberg^1,3^; M. Braschel^1^; S. Moreheart^1^; S.A. Strathdee^4^ and K. Shannon^1,5^**

^1^BC Centre for Excellence in HIV/AIDS, Gender and Sexual Health Initiative, Vancouver, Canada, ^2^University of British Columbia, Interdisciplinary Studies, Vancouver, Canada, ^3^Simon Fraser University, Faculty of Health Sciences, Burnaby, Canada, ^4^University of California San Diego, Medicine, San Diego, United States, ^5^University of British Columbia, Faculty of Medicine, Vancouver, Canada

**Background:** Despite scientific and legal evidence on the harms of criminalization of sex work, Canada adopted new end‐demand legislation – the Protection of Communities and Exploited Persons Act (PCEPA) – in 2014 that criminalizes new aspects of sex work (e.g. clients, third party advertising). One of the explicit goals of end‐demand approaches is to increase access to services and supports for sex workers, despite substantial evidence that criminalization impedes access. This study aimed to longitudinally evaluate the impact of the PCEPA on sex workers’ access to HIV care, primary care, and community/sex worker‐led services in Vancouver, Canada.

**Methods:** Data were drawn from a prospective community‐based cohort of women sex workers, known as AESHA (An Evaluation of Sex Workers Health Access) represented by experiential team members. Multivariable logistic regression with generalized estimating equations (GEE) examined the independent effect of the post‐PCEPA period (2015 to 2017) versus the pre‐PCEPA period (2010 to 2013) on sex workers’ utilization of HIV care and community‐driven services and supports, using time‐updated data.

**Results: ** In separate multivariable confounding models, the post‐PCEPA period was independently correlated with significantly reduced odds of utilizing community‐driven (e.g. sex worker‐led, Indigenous, migrant/refugee, women or youth‐specific) services and supports (AOR 0.60; 95% CI: 0.49 to 0.73). There was no evidence of increased access to HIV‐specific services among sex workers living with HIV following implementation of the new laws (AOR 1.30; 95% CI: 0.85 to 2.00). The post‐PCEPA period was also correlated with significantly reduced odds of accessing health services when needed (AOR 0.59; 95% CI: 0.45 to 0.78).

**Conclusions: **Findings show no increase in utilization of HIV care or other health services post‐PCEPA, and rather a reduction in odds of accessing community‐driven supports and health services when needed. Findings demonstrate that end‐demand approaches to criminalize sex work may not only reproduce the harms of previous criminalized approaches to sex work in Canada, but may further exacerbate barriers to accessing health and community‐led services that have been proven to be key contributors of better health outcomes. There is a critical evidence‐based need to move away from criminalized approaches to sex work to ensure full labor and human rights for sex workers, including access to health, social, and legal support services.

## THAD0105

### “Ending demand” in France: the impact of the criminalisation of sexworkers′ clients on sexworkers′ health, security and exposure to HIV

**H. Lebail^1^; T. Leicester^2^ and C. Giametta^3^**

^1^CNRS – Sciences Po, Centre de Recherches Internationales, Paris, France, ^2^Médecins du Monde ^Doctors of the World^; France Fieldwork Operations, Paris, France, ^3^Aix‐Marseille University, Laboratory or Mediterranean Sociology, Marseille, France

**Background:** As of April 2016 France criminalized sexworkers’ clients as part of new legislation aimed at “Fighting against the prostitutional system” inspired by the so‐called “Swedish model”. Detractors of the new legislation have cited the risks that the new law increases sexworkers’ vulnerability and exposure to stigma and violence, hinders their access to health and legal services and increases risks of exploitation in the sex industry. Based on these arguments, Médecins du Monde (Doctors of the World) initiated a survey aimed at evaluating the impact of the law on sexworkers’ health, rights and well‐being.

**Methods:** Research was conducted between April 2016 and January 2018 based on mixed‐methodology including qualitative semi‐directive interviews and a questionnaire‐based quantitative survey. Two researchers in social sciences oversaw research methodology and data analysis, and data collection was undertaken with a strong involvement of 13 civil‐society organisations (health NGOs, community‐based health NPOs and sex worker groups). 70 semi‐directive interviews with sex workers, 28 interviews with outreach service‐providers and 583 questionnaires completed by sexworkers were collected in nine different cities and overseas territories.

**Results: ** The most direct effect of the 2016 law on sex workers has been an acute increase in their socio‐economic vulnerability. This vulnerability is interconnected with a multiplicity of factors including: the increase of violence; degrading working conditions; negative impact on sexworkers’ health. Our research also highlights the decrease in condom use and the increasing difficulty for sex workers to negotiate safe‐sex practices as well as the difficulty of accessing treatment for HIV+ sexworkers. Whilst it is still too early to evaluate the impact in terms of HIV infections, the research points to an increase in some STIs, notably of syphilis, amongst sexworkers in France.

**Conclusions: **Sexworkers are a key population in the fight against HIV. Our research clearly demonstrates the negative consequences of the criminalisation of clients on sexworkers’ ability to practice safe sex and prevent HIV infection. The results have far‐reaching implications for policy‐makers considering the legal framework around sexwork and are highly relevant to health workers, activists and sex‐worker groups advocating to improve the rights of sexworkers and mitigate the negative impacts of repressive policy.

## THAD0201

### Criminal justice involvement as a social determinant of sexual risk among male migrant and non‐migrant market vendors in Kazakhstan: implications for HIV prevention and human rights

**P. Marotta^1,2,3^; A. Terlikbayeva^1,2,3^; S. Primbetova^1,2,3^; A. Mandavia^1^; T. Hunt^1,2,3^; L. Gilbert^1,2,3^; L. Beletsky^4^; G. Mergenova^1,2,3^; E. Wu^1,2,3^ and N. El‐Bassel^1,2,3^**

^1^Columbia University, New York City, United States, ^2^Social Intervention Group, New York City, United States, ^3^Global Health Research Center of Central Asia, New York City, United States, ^4^Northeastern University, Boston, United States

**Background:** This longitudinal study assessed associations between criminal justice involvement (CJI) and sexual risk behaviors in a sample of male migrant and non‐migrant vendors in the largest open‐air marketplace in Central Asia. We hypothesized that questioning by market officials and migration police, experiencing arrest and incarceration would be associated with greater likelihood of sex under the influence of drugs or alcohol, more than one sexual partner, condomless sex, transactional sex, condomless sex while traveling, and more than one sexual partner while traveling.

**Methods:** We employed respondent driven sampling (RDS) to recruit 1342 male vendors consisting of external migrants from Tajikistan, Uzbekistan, and Kyrgyzstan, internal migrants and a non‐migrant comparison group from Kazakhstan. Multiple imputation with chained equations (ICE) adjusted for bias introduced from missing data at baseline and 3, 6, and 12‐month assessments (5863 observations). Multi‐level logistic regression with time period fixed effects and random intercepts estimated odds ratios (OR) of associations between CJI and sexual risk behaviors overall and by non‐migrant and migrant groups.

**Results: ** Table 1 provides CJI, and sexual risk behaviors stratified by migration status. In multivariate models, questioning by market officials predicted increased risk of condomless sex (OR = 1.02, SE = 0.01, *p* < 0.05), sex under the influence of drugs/alcohol (OR = 1.03, SE = 0.01, *p* < 0.01), transactional sex (OR = 1.05, SE = 0.01, *p* < 0.001) and more than one sexual partner while traveling (OR = 1.06, SE = 0.02, *p* < 0.001). Contacts with migration police predicted increased risk of sex under the influence of drugs/alcohol (OR = 1.02, SE = 0.008, *p* < 0.01). Arrest predicted increased risk of sex under the influence of drugs/alcohol (OR = 1.91, SE = .31, *p* < 0.001), more than one sexual partner (OR = 1.33, SE = .20, *p* < .10), condomless sex (OR = 1.42, SE = .16, *p* < 0.01), transactional sex (OR = 1.96, SE = .44, *p* < 0.01) and more than one sexual partner while traveling (OR = 2.62, SE = .60, *p* < 0.001). Incarceration predicted increased risk of sex under the influence of drugs (OR = 3.79, SE = 1.08, *p* < 0.001), sex with more than one partner (OR = 1.71, SE = .44, *p* < 0.05) and transactional sex (OR = 4.16, SE = 1.70, *p* < 0.001).

**Abstract THAD0201‐Table 1. Population estimates of CJI and sexual risk by migration status (obs. 5868)**

**Table:** 

	External migrant %(obs.)	Internal migrant %(obs.)	Non‐migrant %(obs.)	Overall %(obs.)
Sex under the influence of drugs/alcohol	11.47 (143)	15.08 (141)	11.60 (219)	12.49 (503)
Transactional sex	7.31 (72)	6.76 (66)	5.99 (101)	6.47 (239)
Unprotected sex	35.53 (609)	32.54 (321)	34.66 (585)	34.28 (1515)
>1 sex partner while traveling	3.16 (31)	7.37 (55)	4.24 (59)	4.84 (145)
Questioning by market officials	1.05 (.21)	.50 (.11)	.51 (.11)	.62 (.08)
Questioning by migration police	3.10 (.50)	.86 (.27)	1.13 (.28)	1.47 (.18)
Arrest	26.58 (711)	10.37 (120)	7.69 (190)	12.37 (1021)
Incarceration	4.27 (125)	2.48 (23)	1.83 (34)	2.51 (182)

**Conclusions: **Findings underscore the need for structural interventions that target law enforcement and other criminal justice policies to facilitate HIV prevention interventions with key populations including male labor migrant market vendors in Kazakhstan.

## THAD0202

### Harms of workplace inspections for im/migrant sex workers in indoor establishments: enhanced barriers to health access in a Canadian setting

**B. McBride; K. Shannon; P. Duff; M. Braschel; S.M. Goldenberg and AESHA Study Team**

BC Centre for Excellence in HIV/AIDS, Gender and Sexual Health Initiative, Vancouver, Canada

**Background:** New end‐demand sex work legislation (PCEPA) was passed in Canada in 2014, the enforcement of which may disproportionately impact im/migrant women in indoor venues due to its focus on third party advertising and conflation of sex work with sex trafficking (forced sexual labour). Im/migrant sex workers face intersecting concerns regarding criminalization, restrictive immigration policies, and poor health access, yet evidence is limited regarding how experiences and perceptions of criminalized enforcement impact health access. This study examined correlates of worrying about workplace inspections by authorities and modeled the independent effect of worrying about inspections on health access amongst indoor sex workers over a 2.5‐year period (2014 to 2017).

**Methods:** Longitudinal data were drawn from AESHA, a community‐based prospective open cohort involving 900 +  sex workers across Metro Vancouver. Experiential (current/former sex workers) and non‐experiential staff guided participants through interviewer‐administered semi‐annual questionnaires. Bivariate and multivariable logistic regression with generalized estimating equations (GEE) were used to investigate factors correlated with worrying about workplace inspections, using time‐updated data at each semi‐annual visit. A separate confounder model was used to examine the independent impact of worrying about inspections on barriers to health access.

**Results: ** Across the 2.5‐year study, of 397 indoor sex workers, 23.9% experienced workplace inspections and 51.6% worried about legal, economic or social consequences of inspections. In multivariable GEE analyses, worry about inspections was correlated with recent im/migration (adjusted odds ratio(AOR) 3.13; 95% confidence interval(CI) 1.77 to 5.53), police harassment (AOR 3.49; 95% CI 1.92 to 6.34), workplace violence (AOR 1.66, 95% CI 1.09 to 2.51), and enhanced work stress (AOR 1.05 per additional point on work stress scale, 95% CI 1.01 to 1.09). In a multivariable GEE confounder model adjusted for key confounders, worry about inspections had an independent effect on enhanced barriers to health access (AOR 1.45, 95% CI 1.06 to 1.98).

**Conclusions: **Im/migrant sex workers had higher odds of worrying about inspections and their consequences, and worrying about inspections was linked to enhanced barriers to health access. Findings suggest that enforcement‐based approaches to sex work may exacerbate poor health access amongst sex workers in indoor venues, particularly recent im/migrants. Legal reforms that decriminalize sex work, avoid conflation of sex work and sex trafficking, and enable safer indoor workspaces are recommended.

## THAD0203

### The influence of immigration law concerns on drug use and HIV risk

**B. Suro Maldonado^1^; C. Galletly^2^ and J. Lechuga^3^**

^1^Lehigh University, Education, Bethlehem, United States, ^2^The Medical College of Wisconsin, Milwaukee, United States, ^3^Lehigh University, Bethlehem, United States

**Background:** Hispanic immigrants are disproportionately impacted by health disparities in many domains. For example, they have the second highest rate of Human Immunodeficiency virus (HIV) infection of any racial or ethnic minority group in the United States (U.S). Although past research has documented the impact of the existence of immigration laws that criminalize immigration on access to health care none of the aforementioned studies examined participants’ perceptions and understanding of the immigration laws that lead them to avoid and the effect of such perception on accessing health care services and HIV testing. The purpose of the present study is to investigate the impact of fear of deportation and perceived enforcement of immigration law on Hispanic immigration law related concerns, access to health care and HIV testing.

**Methods:** Three hundred and thirty‐nine U.S Hispanic immigrants between the ages of 18 to 74 years (*M* = 34.08 years, *SD*  = 9.12) were recruited primarily through Spanish radio ads and referrals from a network of community based organizations serving Hispanics in metropolitan areas in Virginia and North Carolina. The inclusion criteria included the following: be at least 18 years old, engaged in sexual risk behavior in the past 12 months, reported negative or unknown HIV status, and lived in the U.S for at least six months. Data analysis consisted of descriptive statistics to describe the sample and blocked logistic and linear regression analysis.

**Results: ** Results indicated that concern with being a public charge, which refers to concern that seeking publicly funded health services will present a barrier for adjusting one's or a family member's immigration status is associated with reduced HIV testing OR  = 0.07 (.02,.28) *p* < 0.01, fear of seeking healthcare services due to immigration status β  = .22, *p* < 0.01, and more logistical barriers seeking health care services β  = .15, *p* < 0.01.

**Conclusions: **Our findings indicate that immigrant's perceptions of immigration law consequences are significantly associated with health behaviors. In light of the present negative political rhetoric towards immigrants around the world it is important to research the potential negative impact that such climate may have in willingness to seek health care services.

## THAD0204

### Ensuring access to primary care for undocumented migrants in England

**Y. Azad; C. Hicks; K. Smithson and D. Gold**

NAT (National AIDS Trust), London, United Kingdom

**Background:** In the UK, the National Health Service (NHS) is a comprehensive health service providing free healthcare to all UK residents. It is funded by taxation i.e it is not an insurance‐based or contributory system. However, in England undocumented migrants are not eligible for free care and are charged for accessing certain services.

**Description:** Migrants, especially from sub‐Saharan Africa, are amongst the most affected by HIV in the UK. Black African men and women living in the UK making up 1.8% of the UK population but 31% of all people accessing HIV care.

Despite securing free HIV treatment for migrants in 2012, we continued to be concerned about charging migrants for other elements of healthcare, which would deter migrants from accessing healthcare and reduce opportunities for migrants to be tested and diagnosed. Primary care is a key site for HIV diagnosis and in England migrants are more likely to be diagnosed with HIV in primary care than in a sexual health clinic.

The Government has looked to implement primary care charging for a number of years. Most recently, in the Government's 2015 to 2016 consultation on further extensions of charging, the Government canvassed views of stakeholders on charging in primary care. We submitted a consultation response detailing the public health impact of such a move. We also supported a coalition of other NGOs to respond making the case for access to primary care. In early 2017, the Government announced that they were putting off immediate plans to charge in primary care.

**Lessons learned:** We have ensured that, despite the current anti‐immigration policy context in England, primary care remains free for everyone irrespective of residency status. The public health argument, in a political climate where migrants are a marginalised and politically unpopular group, is an argument that policy‐makers have particularly taken note of.

Important to the success of our campaigning has been; a united voice from the HIV sector including clinicians, strategic coalitions with health and migration organisations, mobilising parliamentarians, and utilising the evidence base.

**Conclusions/Next steps:** Advocacy will continue to ensure that migrants can continue to access primary care and other vital parts of the health service.

## THAD0205

### Migrant's perspective on TB and HIV in relation to healthcare services: a qualitative study in Stockholm, Sweden

**V. Tirado^1^; J. Shedrawy^1^; S. Strömdahl^1,2^; C. Hanson^1^; K. Lönnorth^1^; A. Kulane^1^ and A.M. Ekström^1,3^**

^1^Karolinska Institutet, Public Health Sciences, Stockholm, Sweden, ^2^Uppsala University, Uppsala, Sweden, ^3^Karolinska Hospital, Stockholm, Sweden

**Background:** While most refugees are young adults in relatively good physical health, the unprecedented stress and trauma that most are exposed to on‐route affects their mental well‐being and also favors the spread of infectious diseases, such as HIV and tuberculosis (TB) and challenges their health. In Sweden, newly arrived migrants are offered a free‐of‐charge examination to identify and provide treatment of infectious diseases, including HIV and TB. However, little is known about the migrants’ knowledge and attitude towards HIV and TB screening in the host country. We aim to explore the knowledge and attitudes about HIV and TB in relation to the healthcare services through migrant's perspective in Stockholm.

**Methods:** Focus group discussions (FGD) and in‐depth interviews (IDI) with migrants were conducted during late 2017 and early 2018 in various settings; specialized health clinics for migrants and civil societal organizations meeting newly arrived migrants in Stockholm, Sweden. The FGD were organized with homogenous groups of seven individuals based on gender, age range and language. The IDI with six migrant men and women were utilized to allow for sensitive thoughts to emerge. The interviews were done with migrants with latent TB, and migrants have that not tested for HIV since arrived to Sweden. We conducted latent content analysis.

**Results: ** In Sweden, migrants reported the following: experience stigma associated with testing for HIV and TB; fear of positive diagnosis for TB and HIV infection: limited knowledge about available services to screen and treat for HIV/TB; delay timely healthcare seeking due to fear of and stigma; and fear of losing social and family support in the case of HIV/TB diagnosis.

**Conclusions: **HIV and TB screening are perceived as a necessity for newly migrants and receive information upon arrival to Sweden. Crowded living conditions, language barriers, stigma, myths about HIV and TB, as well as fear of deportation also add to reluctance of migrants to seek for screening, delaying diagnosis and treatment. It can be seen as an opportunity to critically evaluate actions taken for newly arrived migrants, and to learn from and to share experiences between policy‐makers and service providers concerning migrant's health in Sweden.

## THAD0301

### Community monitoring in HIV prevention programs with sex workers in Kyrgyzstan

**S. Radchenko and A. Myrzabekova**

Tais Plus, Bishkek, Kyrgyzstan

**Background:** The goal is to regularly monitor the quality of services and legal barriers that prevent sex workers from participating in HIV programs, including monitoring of:

(1) approaches to working with sex workers

(2) adequacy of services in terms of needs and context

(3) work of state agencies

Monitoring in the field is accompanied with technical assistance to NGO.

**Description:** Tais Plus conducted monitoring by the community during 2016 to 2017 in the framework of the Global Fund program, the components “Community Systems Strengthening” and “Removing Legal Barriers” (CSS and RLB).

Monitoring tools have been developed for conducting focus groups with NGOs in the following areas: VCT, STI services, capacity of NGOs to work on CSS and RLB.

For two years, Tais Plus staff has visited 6 NGOs on a quarterly basis, which implement HIV prevention programs with sex workers in Kyrgyzstan. During each visit, focus groups were held with NGO staff, as well as meetings with sex workers, during which participants shared their concerns and feedback on the work of NGOs, and also discussed their barriers and opportunities to actively participate in HIV and rights programs.

Conclusions&recommendations from each visit were agreed with NGOs, and then sent to the principal donors funding HIV and rights programs to inform them about the need to make changes in current activities.

Technical assistance was based on the SWIT.

**Lessons learned:** For two years:

(1) 10 Tais Plus staff participated in monitoring visits, 8 of them – on a voluntary basis.

(2) 5 visits were made to most of the NGOs

(3) 47 employees from 6 NGOs participated in monitoring, including focus groups discussions and on‐the‐job trainings

(4) The proposals to improve the following program areas with sex workers were developed and agreed with NGOs: information work, VCT, legal barriers, community strengthening, STI&SRHR services, and increasing NGO capacity. These proposals are addressed to both NGOs and donors.

**Conclusions/Next steps:**

(1) Monitoring through community should be an integral part of HIV programs, and its results should be used to plan and adjust programs

(2) Ensure training employees who are sex workers of visited organizations to monitor and then form mixed monitoring teams.

## THAD0302

### When situations go from bad to worse: strengthening collective action during periods of crisis through concrete guidance and principles for engagement

**R. Dayton^1^; M. Sundararaj^2^; S. Mellors^3^; J. Keatley^4^; J. Chang^5^ and G.T. Brighton^6^**

^1^FHI 360, LINKAGES, Durham, United States, ^2^Global Forum on Men who have Sex with Men and HIV, Oakland, United States, ^3^International HIV/AIDS Alliance, Brighton, United Kingdom, ^4^Innovative Response Globally for Trans Women and HIV, Oakland, United States, ^5^International Network of People Who Use Drugs, London, United Kingdom, ^6^Network of Sex Work Projects, Bulawayo, Zimbabwe

**Background:** Acute violence, defined as periods of increased severe emotional, physical, sexual, and economic abuse, against key populations—men who have sex with men, people who inject drugs, sex workers, and transgender women—is increasing, including in East Africa. Recent examples include the torture of peer educators, the arrest of mobile outreach teams, and the ransacking of key population‐led community‐based organizations (CBOs)—all of which disregard human rights and undermine efforts to curb the HIV epidemic. While local actors lead the response to acute violence, international and regional stakeholders—such as global and regional key population networks, international non‐governmental organizations (NGOs), and donors—must be able to play an effective role if requested.

**Methods:** In 2017, the Technical Advisory Group on Violence, Stigma, and Discrimination Against Key Populations supported by the PEPFAR and USAID‐funded LINKAGES project, convened CBOs, NGOs, United Nations agencies, donors, security experts, and global and regional key population networks working in East Africa to identify current challenges in acute violence responses, articulate principles for local engagement, and generate recommendations for international and regional actors.

**Results: ** Barriers to appropriate and effective responses by international and regional stakeholders’ include: acting without guidance from those most affected, causing added stress and danger for local actors; a failure to adequately resource program staff most at risk, increasing their vulnerability; and support to individual local partners instead of collectives, resulting in duplicative investments and fractured coalitions. Recommendations include deference to local actors, appropriate resourcing, and pre‐emptively forming local collectives and international and regional coordinating bodies that can act in a unified immediate way. Principles to guide international or regional actors’ engagement reinforce the importance of: embracing “first, do no harm” in every aspect of key population programming, avoiding false dichotomies between human rights and HIV objectives, and striving to take a long‐term view even during a crisis.

**Conclusions: **International and regional actors operating in East Africa and beyond can strengthen their responses to acute violence by taking concrete steps, grounded in principles for engagement, thereby protecting key population members’ human rights and removing barriers to the effective implementation of HIV programs.

## THAD0303

### Monitoring and evaluating technical assistance to key populations and HIV projects in Russia: removing legal barriers and community systems strengthening

**S. Ka Hon Chu^1^; M. Golichenko^1^; A. Bidordinova^2^ and R. Elliott^3^**

^1^Canadian HIV/AIDS Legal Network, Research and Policy, Toronto, Canada, ^2^Canadian HIV/AIDS Legal Network, Consultant, Toronto, Canada, ^3^Canadian HIV Legal Network, Research and Development, Toronto, Canada

**Background:** In 2015 to 2017, non‐governmental organizations in Russia delivered HIV services for people who use drugs, sex workers and men who have sex with men with the support of the Global Fund to Fight AIDS, Tuberculosis and Malaria. The service component was aligned with an advocacy component, which included community systems strengthening activities (CSS) to empower and mobilize key affected populations (KPs) to engage in meaningful dialogue with authorities, and activities to educate KPs and provide them with tools to challenge discrimination and remove legal barriers (RLB) for HIV services. In 2016 the Canadian HIV/AIDS Legal Network provided legal technical assistance (TA) for the advocacy component.

**Description:** A Monitoring, Evaluation and Learning framework was designed to collect and analyze quantitative and qualitative data to assess

(1) how staff of HIV projects and KP representatives rated the TA; and

(2) how the access to legal aid changed as a result of the TA.

**Lessons learned:** Through the TA, access to legal aid significantly improved. Over 500 community legal workers (CLWs) and KPs participated in CSS/RLB capacity‐building and networking activities. CLWs consulted with 7683 clients and the TA team provided direct legal support for 950 clients. Of 1195 documented cases, 929 were partially or fully resolved, including through formal complaint and court procedures (580 cases) and legal mediation. Two shadow reports to two UN treaty bodies resulted in strong recommendations to Russia regarding the discrimination and HIV prevention among SW and PWUD. CLWs and project managers rated the TA as a very important component in vitalising legal advocacy and integrating human rights promotion and protection into national, community‐ based programs.

**Conclusions/Next steps:** Legal TA is important to help CLWs and KPs navigate complex legal environments, encourage and empower them to address discrimination and human rights violations, thus reducing the risks of HIV. With the support of the Elton John AIDS Foundation, the Legal Network will implement the “best practices” of the 2016 TA project in its current 2017 to 2020 legal TA project in St. Petersburg to help people who use drugs access a continuum of HIV care services.

## THAD0304

### The Caribbean civil society shared incident database: a monitoring and reporting mechanism to strengthen community activism to address human rights violations

**M. Thompson; K. Mena and I. Cruickshank**

Caribbean Vulnerable Communities Coalition, Programme Department, Kingston, Jamaica

**Background:** People living with HIV and key populations in the Caribbean frequently experience human rights violations including denial of access to health, housing, employment, as well as social exclusion and violence.

**Description:** In 2016, the Caribbean Vulnerable Communities Coalition (CVC) established the Shared Incident Database (SID), the first regional civil society‐led human rights monitoring mechanism which records, analyses and exchanges information on rights violations. It facilitates comprehensive data collection through standardised intake procedures which enhance the capacity of KPs and CSOs to document rights breaches and enables data sharing to support redress or engagement with policy, public health and legal decision‐makers.Clients identifiable details are only visible at an organizational level and there are varied data access levels within an organization profile. At an organizational, national and regional level the database generates non‐identifiable aggregated data with geographic identifiers. The database is linked to redress opportunities such as Cari‐bono which is a network of lawyers around the Caribbean who will be offering pro‐bono services to cases documented. Technical assistance was provided to civil society organizations to build capacity in the community monitoring of legal rights and use of SID.

**Lessons learned:** As a result of improved community monitoring of legal rights, there have been five legal challenges filed before the courts in Jamaica, Guyana and Trinidad & Tobago respectively.

The database has support the building of CSO capacity to carry out redress for their clients. While CSOs engage their clients around redress, it provides capacity building opportunity for community members to take on self‐advocacy.

For instances where persons are not aware of the official patient complaint mechanism, SID has proven to be viable substitute once there is an understanding between the CSOs documenting the right violations and the health care officials.

**Conclusions/Next steps:** Use one of the incidents entered into SID to undertake strategic litigation for the improvement of the legal enabling environment for PLHIV and key populations. To have CSOs documenting rights violations in all the Caribbean countries and territory

Increase awareness.

## THAD0305

### Strengthening HIV‐related community organizations: evidence from a large‐scale program in India

**M. Battala; S. Patel; M. Walia; S. Mukherjee; B. Mahapatra and N. Saggurti**

Population Council, HIV/AIDS, New Delhi, India

**Background:** Community‐organizations (COs) are at the front lines of combating the AIDS epidemic, particularly among marginalized populations. Yet they often have institutional weaknesses that make their stability uncertain. The Bill and Melinda Gates Foundation, through its Avahan program in India, supported the development and strengthening of COs in high HIV prevalence states to help reduce socioeconomic vulnerabilities among key populations. We assessed the capacities of the COs over a three‐year period in program implementation, resource mobilization, networking, and advocacy.

**Methods:** The training of the COs to deliver quality services was based on four pillars:

(1) strengthening institutional capacity,

(2) improving access to social protection schemes,

(3) enhancing financial security and

(4) providing effective social justice and security efforts.

Further COs’ capacities were strengthened in resource mobilization, advocacy, networking, etc. Data collected in two survey rounds during 2015 and 2017 from 48 COs have been used in the analysis, that includes descriptive statistics, frequencies and bivariate techniques.

**Results: ** More than 42,000 new members were enrolled across the COs in the last three years. A three‐fold increase was found in their corpus fund from an average of US$ 2961 per CO in 2014 to US$ 8844 in 2017. Similarly, the COs have been immensely successful in raising funds from various sources; 71% of COs have generated funds in 2017 as compared to 46% in 2014. COs generated on average US$ 3604 in the last three years, 56% of which was raised in the program's last year. COs engagement with different district level forums, government officials and institutions increased significantly over time; Police (35% vs. 75%), Social welfare department (32% vs. 81%), Judiciary (31% vs. 79%) and with formal financial institutions (37% vs. 77%).

**Conclusions: **Most of the COs demonstrated significant improvement in increasing their corpus fund through resource mobilization, establishing networks with a wide range of stakeholders, and taking up various income generating activities. Strengthening COs to implement focused interventions inculcates ownership among members and an increased probability of organizational sustainability.

## THAE0101

### What do we know about interventions to prevent and reduce gender‐based violence among young people living with, or most affected by, HIV in low‐ and middle‐income countries? A systematic review

**F. Meinck^1,2^; M.T. Little^1^; V. Nittas^3^; V. Picker^1^; A. Bustamam^4^; L. Orza^5^; M. Pantelic^5^ and H. Stöckl^6^**

^1^University of Oxford, Social Policy and Intervention, Oxford, United Kingdom, ^2^North‐West University, School of Behavioural Sciences, Vanderbeijlpark, South Africa, ^3^University of Zurich, Epidemiology, Biostatistics and Prevention Institute, Zurich, Switzerland, ^4^McMaster University, Department of Health Research Methods, Evidence, and Impact, Hamilton, Canada, ^5^International HIV/AIDS Aliance, Brighton, United Kingdom, ^6^London School of Hygiene and Tropical Medicine, Global Health and Development, London, United Kingdom

**Background:** Adolescents and young people are disproportionately affected by gender‐based violence (GBV). GBV is associated with an increased risk of HIV acquisition and can disrupt access to treatment and retention in care, resulting in worse HIV outcomes. This systematic review aims to assess effectiveness of existing GBV interventions evaluated among young people vulnerable to HIV aged 10 to 24 in low‐and middle income countries (LMICs).

**Methods:** Studies were identified by searching databases, grey literature, trial registries, back referencing and contact with researchers and program implementers. Abstracts were screened by two researchers according to the inclusion criteria pre‐specified in the review protocol. Randomized, cluster‐randomized and quasi‐experimental studies with control group were included if they assessed GBV or attitudes towards GBV as an outcome. Data were extracted using a form adapted from the Cochrane Collaboration and narratively synthesized. Study quality was assessed using the Cochrane Risk of Bias tool.

**Results: ** Thirteen studies with 35,322 participants were included. Interventions were structural (5), school‐based (4), community‐based (2), and individual‐focused (2). Interventions aimed to empower young women (5), change behaviors (4), remove economic barriers among young women (2) or change gender norms (2). All interventions had multiple components (e.g. life‐skills and health education, micro‐grants, or social support). One intervention specifically included a key population (sex‐workers); the remaining included young people in high HIV‐prevalence settings. No interventions for HIV+ adolescents were found.

Overall, the studies had a median risk reduction of 15% (range: 4% to 60%) in self‐reported GBV exposure favoring the intervention, and two studies reported a median risk reduction of 7% (range: 1% to 12%) in self‐reported GBV perpetration favoring the intervention. Six studies reported reductions in physical/emotional intimate partner violence, three studies reported reductions in physical coercion/violence, one study reported reduction of verbal assault by opposite sex, and four studies reported reduction of any sexual assault/forced sex. No harmful effects could be observed. Structural and school‐based interventions were most effective.

**Conclusions: **Structural and school‐based interventions targeting behaviors, economic barriers, negative gender norms and promoting empowerment may help to substantially reduce and prevent GBV among young people vulnerable to HIV in LMICs. Evidence on effective GBV interventions for young key populations is urgently needed.

## THAE0102

### WINGS of hope: evaluating effects of integrating a brief gender‐based violence prevention intervention with HIV counseling and testing among women who use drugs in Kyrgyzstan

**T. Musagalieva^1^; D. Nikitin^2^; A. Pugachev^2^; L. Gilbert^3^; T. Hunt^3^; T. Jiwatram‐Negron^3^; O. Rychkova^4^; A. Mukambetov^5^; I. Sadykov^5^; I. Ermolaeva^6^; R. Bayazitova^7^; M. Atabekova^6^; N. Sharonova^8^; M. Akmatova^8^; M. Abduraupova^9^; S. Nazarova^9^; E. Tkacheva^10^ and R. Mazhitov^11^**

^1^ASTERIA Foundation, Bishkek, Kyrgyzstan, ^2^GLORI Foundation, Bishkek, Kyrgyzstan, ^3^Columbia University Social Intervention Group, New York, United States, ^4^Previously Open Society Foundations, New York, United States, ^5^Soros Foundation Kyrgyzstan, Bishkek, Kyrgyzstan, ^6^Asteria Foundation, Bishkek, Kyrgyzstan, ^7^Asteria Foundation & Ganesha Foundation, Bishkek, Kyrgyzstan, ^8^Podruga Foundation, Osh, Kyrgyzstan, ^9^Positive Dialogue Foundation, Osh, Kyrgyzstan, ^10^Chance Crisis Center, Bishkek, Kyrgyzstan, ^11^Plus Center, Osh, Kyrgyzstan

**Background:** Gender‐based violence (GBV) negatively impacts access of women who use drugs (WWUD) to HIV prevention and to drug treatment. Collaboratively with consultants at Columbia University Social Intervention Group, partner Kyrgyz‐based NGOs and GLORI Foundation, we developed WINGS of Hope intervention that includes GBV screening, brief intervention and referral to treatment service (SBIRT) with HIV counseling, testing and linkage to care. We evaluated the feasibility and acceptability of WINGS (Women Initiating New Goals for Safety) GBV‐prevention model adapted for helping WWUD in Kyrgyzstan to access HIV prevention and harm reduction services.

**Methods:** In 2013 to 2016, 213 WWUD in Kyrgyzstan participated in WINGS of Hope intervention study. Each participant attended the 2‐session GBV SBIRT intervention that included raising awareness about different types of GBV and how GBV increases risk for substance misuse and HIV, screening for GBV, safety planning, and identifying goals to increase safety. Women participants were offered voluntary HIV rapid testing and linkage to HIV care when necessary (Gilbert et al., 2016). We used a pre‐post design to evaluate the effects of the intervention.

**Results: ** At 3‐month follow‐up assessment, participants experienced 11% fewer intimate partner and 39% fewer gender‐based violence incidents of any kind than what they had experienced at baseline. Also, there was a 38% increase in women who experienced neither IPV nor GBV “in the past 3 months”. The number of women who completed the gender‐specific HIV testing, increased from 37% to 61%, from 14% to 29% increased number of women who received counseling or group support to deal with GBV. All 10 women who tested positive for HIV, including three new cases, were referred to HIV treatment at the AIDS Centers.

**Conclusions: **The high rates of participation, attendance, and retention, as well as significant reductions in GBV victimization suggest the feasibility and promising effects of this WINGS SBIRT‐based intervention to help WWUD who experience GBV, and link them to appropriate HIV services. The GBV services for WWUD should be integrated into and coordinated with social, medical and legal services. The project findings underscore the need for gender‐sensitive harm reduction services for WWUD.

## THAE0103

### Integrating gender‐based violence screening and support into HIV counselling and testing for adolescent girls and young women accessing PrEP in South Africa and Tanzania – experiences from the EMPOWER study

**M.B. Colombini^1^; L. Ramskin^2^; N. Khoza^2^; A. Stangl^3^; F. Scorgie^2^; S. Harvey^4^; C. Manyamba^2^; C. Watts^4^; S. Kapiga^5^; S. Delany‐Moretlwe^2^ and EMPOPOWER Study group**

^1^London School of Hygiene and Tropical Medicine, Public Health and Policy, Pretoria, South Africa, ^2^Wits Reproductive Health Institute, Johannesburg, South Africa, ^3^International Center for Research on Women, Washington DC, United States, ^4^London School of Hygiene and Tropical Medicine, Public Health and Policy, London, United Kingdom, ^5^Mwanza Intervention Trials Unit, Mwanza, Tanzania, United Republic of

**Background:** Partner violence may undermine oral PrEP use, yet evidence is scarce on how to best to support PrEP use while decreasing vulnerability to violence. We assessed the feasibility and acceptability of integrating gender‐based violence (GBV) screening and support into HIV counselling for adolescent girls and young women (AGYW) accessing oral PrEP.

**Methods:** EMPOWER is an open‐label PrEP demonstration project for AGYW (16 to 24 years) in South Africa and Tanzania. We adapted HIV counselling and testing guidelines for lay counsellors to include five questions about exposure to gender‐based violence (GBV), recommended by the World Health Organisation. Participants were screened at baseline and at each follow‐up visit. We analysed data from counselling session observations (n = 10 in SA only) and in‐depth interviews with participants (n = 39, SA  = 25, Tz  = 14) and clinical staff (n = 13, SA  = 10, Tz  = 3). Themes explored included: comfort with GBV screening sessions, usefulness of risk assessment and safety planning, and appropriateness of referrals to GBV support services.

**Results: ** We screened 619 and enrolled 431 HIV negative AGYW (SA = 379; Tz = 52). 141 (SA = 119; Tz = 22) reported lifetime experiences of violence at baseline. Including GBV screening within HIV counselling sessions was feasible, provided continuous training and staff support was available. Overall, study participants were amenable to GBV screening, provided that the basic principles of confidentiality, staff empathy, and absence of judgment were observed. Participants who reported abuse said that it was reassuring and helpful to talk to friendly, non‐judgemental counsellors. Challenges reported by HIV counsellors included: initial discomfort in asking about violence; facilitating disclosure of suspected cases; length of time taken to complete the sessions; and offering help when participants did not want any referrals. Staff felt supported by regular debriefings, a directory of referral services for GBV, and an on‐site social worker.

**Conclusions: **Overall, our study suggests that integrating GBV screening into HIV counselling and testing for AGYW is acceptable and feasible when appropriate referral, staff debriefing and technical support are offered, and basic principles of empathetic listening and confidentiality are respected. It is essential that counselling for this group is adolescent‐friendly and non‐judgmental.

## THAE0105

### Group therapy for gender‐based violence (GBV): Reducing HIV and GBV risk among adolescent girls and young women in Nairobi's informal settlements

**M. Muia^1^; B. Odero^1^; M. Nderitu^2^; T. Simiyu^3^; C. Bennett^4^; M. Kariuki^2^ and D. Wendo^5^**

^1^National Organization of Peer Educators, Nairobi, Kenya, ^2^IMA World Health, Nairobi, Kenya, ^3^USAID, Nairobi, Kenya, ^4^IMA World Health, Washington, United States, ^5^IMA World Heatlh, Nairobi, Kenya

**Background:** The 2015 to 2018 USAID/Kenya and East Africa funded Afya Jijini program is implementing the Determined, Resilient, Empowered, AIDS free, Mentored and Safe (DREAMS) intervention to help empower adolescent girls and young women (AGYW) and reduce their HIV risk in Nairobi, Kenya's informal settlements. Five in every 10 Kenyan women (about 47%) between ages 15 to 49 have experienced at least one form of violence in their lifetime. Adolescent girls and young women in Nairobi's informal settlements are uniquely vulnerable to HIV. Their vulnerability cuts across behavioral, social and biological factors. AGYW often engage in age‐disparate and/or transactional relationships as a result of poverty and unemployment, putting them at increased HIV risk. The perception that AGYW can negotiate monogamy, condom use, as well as request their male sexual partners (MSPs) to get circumcised, is still largely unheard of for most AGYW living in the project's catchment areas.

**Methods:** Group therapy for GBV, facilitated by a trained trauma counselor allows AGYW to cultivate trust and share GBV experiences during group sessions. This therapy includes recognition of emotional self‐awareness, followed by cognitive autonomy sessions that enable AGYW to take responsibility for their own change. The therapy sessions provided post violence counseling support to 2452 enrolled AGYW at 12 safe spaces across Mukuru Kwa Njenga Ward. Ninety‐five percent (n = 2320) of AGYW accessed counseling for physical violence, with 5% (n = 124) reached with emotional counseling. Afya Jijini worked with the girls and formed post GBV support groups with over 1050 (43%) AGYW.

**Results: ** The need for trauma counseling support and information sharing on GBV‐related matters is pivotal to AGYW transformation. The earlier the AGYW are exposed to GBV prevention information, the increased likelihood of reporting GBV cases. Multiple platforms for GBV prevention awareness is key for communities to shun negative gender norms. Post GBV therapy groups offer a base for advocacy and networking for collective voice against the social issue.

**Conclusions: **Discussing GBV is a taboo issue in Kenya. Through group GBV therapy sessions, AGYW find a platform to discuss these issues. Furthermore, AGYW are able to recognize their roles in mitigating GBV.

## FRAD0101

### The Mexico City Policy and PEPFAR: estimating the impact on NGOs and funding

**K. Moss and J. Kates**

Kaiser Family Foundation, Washington, United States

**Background:** In January 2017, President Trump reinstated and expanded the Mexico City Policy. In the past, it had required foreign non‐governmental organizations (NGOs) to certify that they would not “perform or actively promote abortion as a method of family planning” using funds from any source as a condition for receiving U.S. family planning assistance. In a significant expansion, it now applies to almost all U.S. global health bilateral assistance, including PEPFAR. Among the many questions about the policy's impact is its effect on HIV programs and services. This study sought to estimate the number of NGO recipients of PEPFAR funding who could be subject to the policy as well as the amount of funding they receive.

**Methods:** We analyzed data from ForeignAssistance.gov over the most recent three‐year period for which such data were available (FY 2013 to FY 2015) to identify NGO recipients of bilateral HIV assistance as a proxy for the current number of recipients. We also calculated the amount of funding they receive and number of countries they work in. We further stratified these countries by the legality of abortion.

**Results: ** We identified 470 foreign NGO prime recipients of PEPFAR bilateral HIV funding, who received $873 million. In addition, we identified 274 U.S. NGO prime recipients, accounting for $5.5 billion, who would be required to ensure that any foreign NGO sub‐recipients were in compliance. Overall, this funding supported programs in 61 countries. Of these, 36 allow for legal abortion in at least one case not permitted by the policy and 24 do not; one country was not classifiable. These estimates should be considered minimums since we were unable to identify NGO sub‐recipients of HIV support, who represent a much greater number and are also affected by the policy.

**Conclusions: **The expansion of the Mexico City Policy to encompass almost all U.S. bilateral global health assistance, including PEPFAR, greatly extends its reach. While it is still too early to know its ultimate impact on the ground, our analysis indicates that the expanded policy will likely affect hundreds of NGO recipients of PEPFAR support.

## FRAD0102

### Understanding the global gag rule: how to sustain global health progress amidst the new U.S. policy environment

**C. Cooney^1^; T. Coenen^2^ and J. Rucks^3^**

^1^Planned Parenthood Federation of America, Washington, United States, ^2^Rutgers, Utrecht, Netherlands, ^3^PAI, Washington, United States

**Background:** The global gag rule (also known as the Mexico City Policy) is affecting the global fight against HIV/AIDS on an unprecedented scale by directly applying to PEPFAR‐funded programs for the first time ever.

**Description:** This session will provide conference participants a comprehensive review of the global gag rule, experiences with the policy, expert guidance for implementers, and advocacy opportunities. The presenters will offer a variety of perspectives from both policy experts and implementing organizations and will specifically focus on the expansion to U.S.‐funded HIV/AIDS programs and the disproportionate impact the policy will have on women and girls and integrated service provision. Session co‐sponsors include: amfAR, AVAC, Center for Health and Gender Equity (CHANGE), Marie Stopes International (MSI), PAI, Planned Parenthood Federation of America, Rutgers, and Stop AIDS Alliance.

**Lessons learned:** Under previous iterations of the global gag rule from 2001 to 2009, the policy resulted in a number of harmful impacts, including:

(1) USAID had to end condom shipments to Lesotho at a time when one in four women in the country was infected with HIV because the Lesotho Planned Parenthood Association, the primary conduit for condoms in the country, could no longer receive U.S. funding under the global gag rule; and

(2) Between 2002 and 2006 in Kenya, the global gag rule led to the termination of critical activities run by the Family Planning Association of Kenya and Marie Stopes International (MSI) Kenya—the leading providers of health care to people living in poor and rural communities in the country

This session will highlight these and other examples of the global gag rule's impact, with a focus on both historical and emerging evidence where HIV/AIDS programs and providers are seeing the impact of the policy.

**Conclusions/Next steps:** This comprehensive review of the global gag rule will provide new insight into the harmful impact of the policy on HIV/AIDS programs and engage participants in a dynamic discussion with policy and program experts on the impact of the policy and implications for future HIV/AIDS policy and programs.

## FRAD0103

### A model of dis‐integration: unpacking the impact of the global gag rule on HIV‐SRHR linkages

**L. Orza^1^; L. Stackpool‐Moore^2^; E. Restoy^3^ and J. Davis^3^**

^1^International HIV/AIDS Alliance, Bridport, United Kingdom, ^2^Watipa, London, United Kingdom, ^3^International HIV/AIDS Alliance, Brighton, United Kingdom

**Background:** The HIV community has expressed grave concern for what the expanded Global Gag Rule (GGR) could mean for the health and rights of people most affected by HIV. While the impact of the GGR on the provision of reproductive health services is beginning to be understood, little has been done to document the impact of the rule on communities most impacted by HIV, especially on HIV prevention. The International HIV/AIDS Alliance, with support from Sida, has undertaken research in Ethiopia and Zimbabwe to project the potential impact of the GGR on HIV and key population services, and on HIV‐SRHR integration.

**Methods:** This is a mixed methods study combining desk research, statistical forecasting and community engagement (focus groups, stakeholder meetings and semi‐structured interviews). It has involved the participation of civil society organizations – including those representing key populations – working on HIV, including through delivering a comprehensive, integrated SRHR agenda.

**Results: ** Preliminary findings forecast that loss of funding to civil society partners implementing integrated HIV/SRHR programmes in all study sites will result in:

(1) Reduction in access to HIV prevention, testing and treatment services caused by the disintegration of HIV/SRHR services

(2) Denial of life‐saving treatment and essential services to people living with HIV

(3) The health of people from key and vulnerable populations being jeopardised by restrictions to the package of services they can receive

(4) Fear that advocacy efforts to maintain or liberalise laws which allow access to safe abortion will be stifled due to confusion, uncertainty, and fragmentation of civil society; however, there is also evidence of civil society seeking creative opportunities for new advocacy collaborations

**Conclusions: **Non‐GGR compliant HIV organizations implementing comprehensive integrated SRHR programming have lost tens of millions of dollars in funding. These translate into: cuts to prevention programming for key populations; denial of treatment to people living with HIV; the un‐doing of decades of progress to integrate SRHR and HIV programmes, including for adolescents and young people; and a silencing of civil society on issues of individual choice, rights and agency. Despite additional barriers to accessing HIV and SRHR services, examples of community resilience, partnership, and solidarity were identified.

## FRAD0104

### Caught by ideology: HIV providers in the era of the protecting life in global health assistance policy (AKA Mexico City Policy)

**B. Honermann^1^; B. Roose‐Snyder^2^; T. Gonese^3^; A. Sharp^1^ and J. Sherwood^1^**

^1^amfAR, Public Policy Office, Washington, United States, ^2^Center for Health and Gender Equity ^CHANGE^, Public Policy, Washington, United States, ^3^Southern Africa Litigation Centre, Johannesburg, South Africa

**Background:** On 15 May 2017, the US government formally announced the introduction of the Protecting Life in Global Health Assistance Policy (The Policy) – formerly known as the Mexico City Policy (MCP) or the Global Gag Rule (GGR). The Policy prohibits any foreign‐NGO receiving Global Health Assistance funding to certify that it will not “perform or actively promote abortion as a method of family planning (…) or provide financial support to to any foreign‐NGO that conducts such activities”. This includes what an organization is able to do with non‐USG funding. Distinct from the MCP, however, was a new affirmative defense against the Policy that states: “(…) in the event of a conflict between (the Policy) and an affirmative duty of a healthcare provider required under local law to provide counseling about and referrals for abortion (…), compliance with such law shall not trigger a violation of (the Policy).”

**Description:** The expansion of the Policy has significant implications for organizations delivering HIV services globally. For organizations funded for prevention of mother‐to‐child transmission services, community health worker engagement, and those with integrated family planning and HIV services – most of whom have no prior experience with the MCP – incorporating the Policy is legally complex and ethically dubious. We conducted a legal assessment of the applicability of the affirmative defense in several African countries.

**Lessons learned:** South Africa has particularly strong Constitutional protections for reproductive rights (sections 12 (2)(a) and 27 (1)(a)), strong informed consent requirements in the National Health Act and ethical guidelines, and robust case law that compel healthcare providers – including PEPFAR partners – to continue counseling about and referring for abortion services that cannot be circumvented by the Policy. Evaluations in Mozambique, Zambia, and Zimbabwe have found differing levels of protections.

**Conclusions/Next steps:** The legal and ethical obligations of HIV providers requires assessing the legal landscape in which they are operating and standing up for women's reproductive health choices. South Africa in particular has provided a template that advocates should utilize in pressing for legislation that enables healthcare providers to mitigate some of the substantial harms created by the Policy.

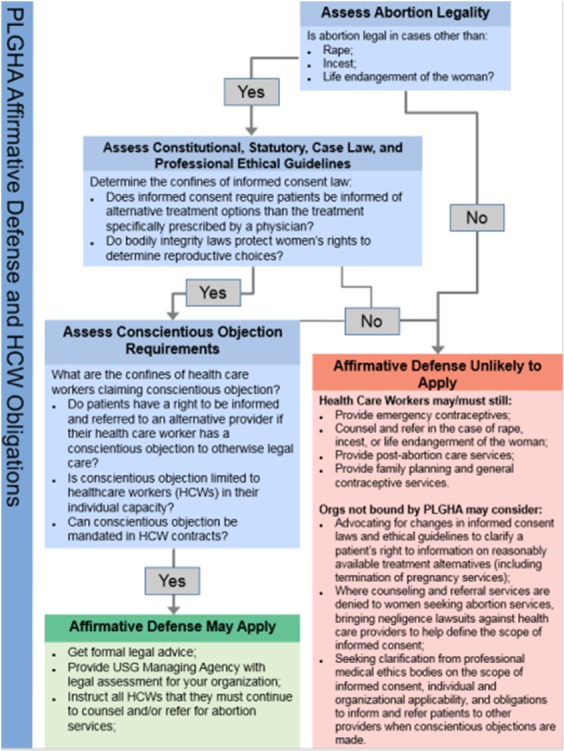

**Abstract FRAD0104‐Figure 1. PLGHA Affirmative Defense and HCW Obligations.**

## FRAD0105

### The impact of the USG Policy Protecting Lives in Global Health Assistance (PLGHA) on Sida's SRHR‐HIV partnerships

**P. Engstrand^1^; M. Hildebrand^2^; S. Thomsen^3^; Å. Andersson^4^ and M.‐T. Bejarano^5^**

^1^Sida, Lead Policy Specialist Health & SRHR, Stockholm, Sweden, ^2^Embassy of Sweden / Lusaka, Regional SRHR Team, Lusaka, Zambia, ^3^Sida, Senior Policy Advisor Health, Stockholm, Sweden, ^4^Sida, Senior Programme Specialist, Health, Stockholm, Sweden, ^5^Sida, Research Advisor, Stockholm, Sweden

**Background:** In January 2017, the USG reinstated the Mexico City Policy (MCP) expanding its scope beyond Family Planning Assistance to include US Global Health Assistance (US GHA). In short, MCP/PLGHA prohibits foreign NGOs receiving US GHA to perform or promote abortion, regardless of funding source. Subsequently, Sida adopted a position and guidance on MCP/PLGHA to assist staff to identify potential risks to Sida‐supported SRHR programmes should partners decide to comply with MCP/PLGHA.

**Description:** Between August‐September 2017, Sida undertook a portfolio assessment to understand the implications of MCP/PLGHA on 30 of Sida's NGO‐partners receiving funding for SRHR incl. HIV. Data was analyzed using descriptive statistics and qualitative thematic analysis.

**Lessons learned:** One third of Sida's SRHR partners had ongoing US GHA agreements. Of those, two US based NGOs indicated intent to comply with MCP/PLGHA. Six Foreign NGOs choose not to comply with MCP/PLGHA; for these, US GHA contributed between 5 to 60 percent of their total budget. At least ten of Sida's partners had sub‐grantees that received US GHA and were considering complying with MCP/PLGHA. In one case a national member association took over program implementation, and will not be required to comply with MCP/PLGHA. HIV organizations providing integrated SRH‐HIV services including some level of counselling and referrals for safe abortion care, but that do not provide abortion services or advocacy, were for the first time affected by the expanded scope of the policy. The potential impact and loss of funding led to uncertainties and confusion for the sector which Sida has had to mitigate choosing various strategies including re‐programming of agreements.

**Conclusions/Next steps:** The assessment found that the MCP/PLGHA may potentially cause disintegration of SRH‐HIV services and systems, jeopardizing gains made in recent decades for comprehensive SHR‐HIV services for adolescent girls and young women who are disproportionate at risk of HIV. MCP/PLGHA may also have a “silencing effect” on broader SRHR policy reform linked to gender equality and the SDGs. Discontinuation of services compounds the vulnerability of those who are already left behind such as the poorest and marginalised people, people in rural areas, LGBT‐communities and people living with HIV, adolescent girls and young women.

## FRAE0101

### Implementation of appointment spacing model of differentiated care in Ethiopia‐successes and challenges

**T. Assefa^1^; Z. Melaku^1^; W. Amdino^1^; A. Abebe^2^; M. Rabkin^3^; K. Hartsough^3^ and R. Fayorsey^3^**

^1^ICAP Ethiopia, Addis Ababa, Ethiopia, ^2^Federal Ministry of Health, Disease Prevention and Control Directorate, Addis Ababa, Ethiopia, ^3^Columbia University, Mailman School of Public Health, New York, United States

**Background:** There are over 420,645 people living with HIV on antiretroviral therapy (ART) in Ethiopia. This number is expected to increase by 30% with the adoption of Test and Treat by the Federal Ministry of Health (FMOH) in 2016. This poses a challenge to the Ethiopian health system, where shortage of clinical staff is a recurring problem.

**Description:** To address this issue, ICAP, in collaboration with the FMOH and the Centers for Disease Control and Prevention conducted a pilot of a differentiated service delivery (DSD) six‐month appointment spacing model (ASM). ICAP established a technical working group on DSD and developed supplemental guidelines, monitoring framework, provider support tools, and training curriculum for regional and facility service providers. Six high‐volume health facilities were included in an ASM pilot, which began in April 2017. Eligible non‐pregnant adult patients (stable per World Health Organization criteria) were enrolled in the pilot, which included six‐ months medication supply, biannual clinical visit, annual viral load testing, psychosocial support, counseling, and encouragement to participate in peer support groups.

**Lessons learned:** Of the 24,657 clients that were currently on ART at the six pilot facilities, 12,649 clients (51%) were eligible for ASM, of whom 49% were enrolled by the 6^th^ month of the pilot. Among the 51% who declined participation, the major reasons cited for refusal to participate in the ASM were fear of inadvertent disclosure due to having a large volume of medication and concerns regarding safety and storage of medication for prolonged periods at home. ASM has been scaled‐up to 765 facilities, with 57,000 clients enrolled as of November 2017.

**Conclusions/Next steps:** Implementation of the ASM in the Ethiopian context demonstrated rapid enrollment of a large number of clients within a short period, and acceptance of the model by clients and service providers. Future work is needed to increase acceptance of ASM model and address common concerns cited by those who declined to take part in pilot. Additionally, work will be needed to evaluate retention on ART and healthcare worker and client satisfaction at 1.5 years on ASM.

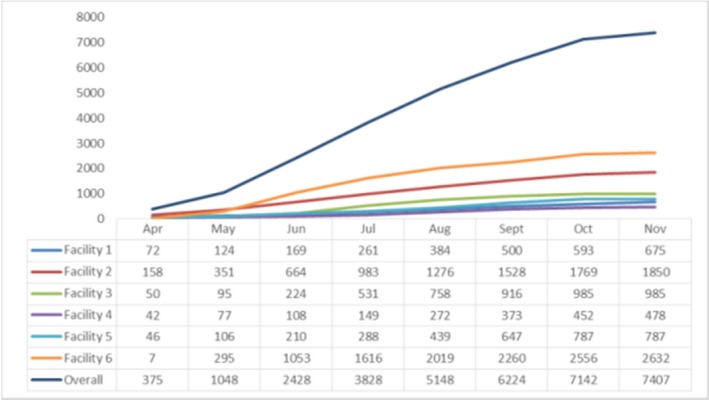

**Abstract FRAE0101‐Figure 1. Cummulative ASM enrollment at the six pilot hospitals, April to November 2017.**

## FRAE0102

### Quick & cheaper: a comparison of patient costs and distance to access care through differentiated models of antiretroviral treatment delivery in Zimbabwe

**F. Chirowa^1^; N. Ngorima‐Mabhena^1^; O. Mugurungi^2^; I. Mlingo^1^; T. Makuve^1^; B. Ndidzano^1^; E. Tarowera^1^; G. Fatti^3^; A. Grimwood^3^ and S. Rosen^4^**

^1^Khethimpilo, Harare, Zimbabwe, ^2^Ministry of Health & Child Care, Aids and TB, Harare, Zimbabwe, ^3^Khethimpilo, CapeTown, South Africa, ^4^HE2RO, Johannesburg, South Africa

**Background:** .Patient transport costs are a significant barrier to accessing antiretroviral treatment (ART), particularly for those living in rural areas of sub‐Saharan Africa. As part of a pragmatic cluster‐randomized trial to assess the effectiveness of differentiated models of ART delivery for stable ART patients, we compared patient‐level costs and travel distance for participants accessing ART at health facilities vs. participants expected to access ART in community ART refill groups (CARGs) in Zimbabwe.

**Methods:** Patient‐level cost data of trial participants were collected at 26 health facilities in both urban and rural areas of five districts between July 2017 and December 2017, using a standardized questionnaire administered by study nurses. At 10 facilities, participants accessed ART directly from the facility, while for the remainder participants were patients in newly formed CARGs, which are groups of 6 to 12 participants who meet in the community for drug pick‐up; patients in CARGs were asked their expectations in joining groups. The average visit cost of accessing care, mean travel distance and time spent per visit were compared. Costs included both direct costs and the opportunity cost of attending the appointment, both to the patient and an accompanying person.

**Results: ** Amongst 473 respondents, 260 (55%) received ART at the health facility and 213 (45%) received ART in CARGs. CARG participants had a 89% shorter travel distance to the selected meeting place for a drug pick‐up. (0.9 km; 95% CI: 0.8 to 1.0 km) compared to facility‐based participants (8.4 km, 95% CI: 6.8 to 9.9 km); *p* < 0.00. The average visit cost for CARG participants is 69.5% lower than facility‐based patients (US$1.24 vs. US$4.06, two‐way respectively. Travel time to the drug pick up point is lower for CARG participants: 178 (91%) reported travel time for <30 minutes, while 100% of facility‐based patients travelled >30 minutes.

**Conclusions: **Collecting ART from CARGs has lower visit costs, and patients travel substantially shorter distances with reduced travel times to access care, compared to participants collecting ART from facilities. CARGs are a differentiated model of care offering important benefits to stable ART patients.

## FRAE0103

### Decentralizing and differentiating HIV care for men who have sex with men living with HIV in Guatemala City: acceptability and retention in care

**C. Barrington^1^; M.I. Loya Montiel^2^; S. Northbrook^3^; J.K. Williams^4^; J.P. Alvis^2^; R. Santa Luce^2^; K. Guzman Guerrera^2^; R. Pinzon Meza^5^; M.E. Anton Urbina^6^ and M.R. Calderon^2^**

^1^University of North Carolina at Chapel Hill Gillings School of Global Public Health, Health Behavior, Durham, United States, ^2^Universidad del Valle de Guatemala, Guatemala, Guatemala, ^3^Centers for Disease Control, Guatemala, Guatemala, ^4^Centers for Disease Control, Atlanta, United States, ^5^Hospital Roosevelt, Guatemala, Guatemala, ^6^Ministerio de Salud Publica y Asistencia Social, Programa Nacional de Prevención y Control de ITS, VIH y SIDA, Guatemala, Guatemala

**Background:** Centralized HIV care causes social, time, and cost burdens, which can threaten achieving optimal HIV clinical outcomes and wellbeing. We conducted the first decentralization pilot in Central America among men who have sex with men (MSM) living with HIV and assessed acceptability, retention in care, and satisfaction.

**Methods:** This is a longitudinal, intervention study with mixed‐methods assessments at baseline, six months, and twelve months. We recruited participants consecutively from January to May 2017. Eligibility criteria included ≥18 years old, male, self‐reported sex with men, diagnosed with HIV, enrolled in care for ≥one year without interruption, viral load ≤1000 copies/mL, and on first‐line treatment. Eligible participants were offered the option to decentralize to one of three clinics with tailored services for key populations (2 NGO and one governmental). Retention in care was defined as attending at least three quarterly appointments during nine months of follow‐up. Evaluation methods included socio‐behavioral surveys, in‐depth interviews, and clinical chart review. Thematic qualitative analysis and descriptive statistics were used to assess indicators and processes related to acceptability and retention in care.

**Results: ** Nearly half (47%) of the 276 participants voluntarily decentralized while 53% opted to stay, with no significant differences between the two groups. Among those who decentralized, 51% (n = 66) chose a gay health NGO, 41% (n = 53) chose an NGO with a history of HIV prevention and care with key populations, and 8% (n = 11) chose a governmental STI clinic. Motivators for decentralization included schedule, location, and type of organization. Only 1 participant opted to re‐centralize due to schedule issues and 98% of decentralized participants were retained in care at the third clinic visit during study follow‐up. Median duration of the decentralized consult was 30 minutes, compared to 4 to 5 hours at the centralized clinic. Over 90% of participants across the three clinics considered the care they received to be “excellent”.

**Conclusions: **Decentralization to key population‐friendly clinics with flexible schedules is acceptable and feasible for MSM. Retention was not negatively affected and participants were highly satisfied with the services provided in the decentralized clinics. These findings are informing policy and practice throughout Central America to differentiate care and improve quality.

## FRAE0104

### Urban adherence clubs in Zambia: findings from model implementation

**M. Roy^1^; C. Bolton^2,3^; I. Sikazwe^2^; M. Mukumbwa‐Mwenechanya^2^; E. Efronson^2^; P. Somwe^2^; E. Kalunkumya^2^; M. Lumpa^2^; A. Sharma^2^; J. Pry^2,4^; N. Padian^5^; E. Geng^1^ and C. Holmes^6,7^**

^1^University of California San Francisco, San Francisco, United States, ^2^Centre for Infectious Disease Research in Zambia, Lusaka, Zambia, ^3^University of Alabama, Birmingham, United States, ^4^University of California Davis, Sacramento, United States, ^5^University of California Berkeley, Berkeley, United States, ^6^Georgetown University, Washington DC, United States, ^7^Johns Hopkins University, Baltimore, United States

**Background:** The urban adherence club (UAC) model is a differentiated service delivery (DSD) model designed to improve on‐time drug pickup and retention in HIV care through off‐hours facility access and group drug distribution. Successes during scale‐up in South Africa have been tempered by a recent report of high loss to follow‐up and transfers back to facility‐based care. We sought to characterize retention among patients enrolled in UACs at five urban health facilities in Zambia to evaluate model implementation outside of South Africa.

**Methods:** As part of a cluster randomized trial, a systematic sample of eligible patients (HIV+, on ART >6 months, not acutely ill, CD4 ≥200/μL) were enrolled in UACs between May 19 & July 29, 2016. Patients were scheduled for bimonthly group drug‐pick up meetings in the first six months and every 3‐month meetings thereafter. Clinical and pharmacy visit data were obtained through the existing electronic medical record. UAC meeting visit attendance, transfer‐outs, and deaths were collected prospectively through October 31, 2017.

**Results: ** Among 592 intervention patients, median age was 41 years (IQR: 35 to 48), 371 (63%) were female, median CD4 count was 411 /μL (IQR: 273 to 559), and median time on ART was 4.0 years (IQR: 2.0 to 7.2). Out of 3756 scheduled UAC visits, 685 (18%) were not attended. In 204 (30%) of the unattended visits, patients still obtained medication on the same day: either via a buddy (151 (74%)) or same day drug‐pick up at the facility outside of the UAC meeting (53 (26%)). Among the 481 (70%) unattended visits where patients did not receive same‐day medication, cumulative incidence of drug‐pick up after a missed UAC visit was 27% at 14 days and 32% at 28 days (Figure 1). At 12 months, cumulative incidence of treatment interruption (>14 days late for drug pick‐up) was 9.8%, transfer out of UAC was 6.8%, and death was 0.51%.

**Conclusions: **Group meetings were generally well‐attended and in nearly one third of missed meetings patients accessed timely drug pick‐up via other means. These findings suggest that group‐based care is a viable model of care, although adaptation and patient‐centeredness should be prioritized in DSD model implementation.

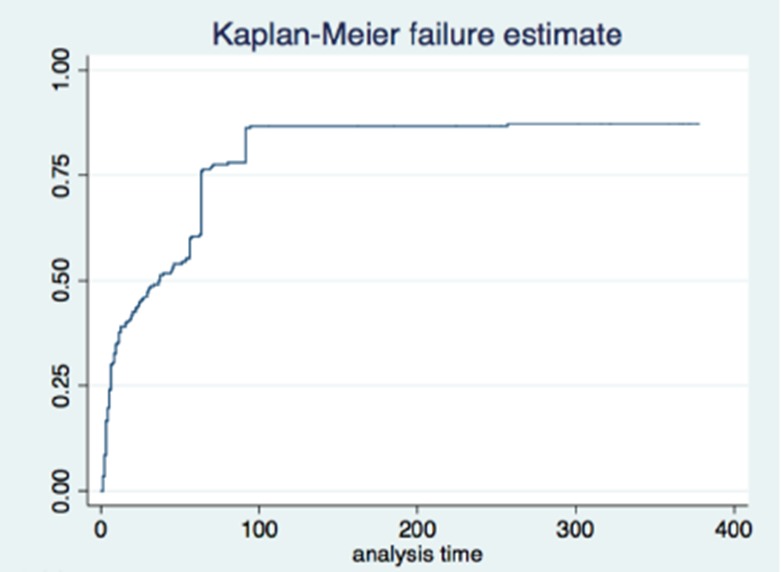

**Abstract FRAE0104‐Figure 1. Kaplan‐Meier survival curve of time to drug pick‐up after first unattended group visit.**

## FRAE0105

### The impact of community delivery of antiretroviral therapy on viral load suppression: findings from a pragmatic randomized non‐inferiority trial in Dar es Salaam, Tanzania

**J. Francis^1^; P. Geldsetzer^2^; N. Ulenga^1^; D. Sando^2^; I. Lema^1^; E. Mboggo^1^; M. Vaikath^2^; S. Lwezaula^3^; H. Koda^1^; J. Hu^4^; R. Noor^2,5,6^; I. Olofin^2^; W. Fawzi^2,5,7^; G. Asmus^8^ and T. Bärnighausen^2,8,9^**

^1^Management Development for Health, Dar es Salaam, Tanzania, United Republic of, ^2^Harvard T.H. Chan School of Public Health, Global Health and Population, Boston, United States, ^3^National AIDS Control Program, Dar es Salaam, Tanzania, United Republic of, ^4^Duke University, School of Medicine, Durham, United States, ^5^Harvard T.H. Chan School of Public Health, Department of Nutrition, Boston, United States, ^6^Africa Academy for Public Health ^AAPH^, Dar es Salaam, Tanzania, United Republic of, ^7^Harvard T.H. Chan School of Public Health, Department of Epidemiology, Boston, United States, ^8^Institute of Public Health, Heidelberg University, Heidelberg, Germany, ^9^Africa Health Research Institute, Mtubatuba, South Africa

**Background:** Delivering antiretroviral therapy (ART) to patients’ homes and other meeting points in the community using community health workers (CHWs) could reduce patient volumes at healthcare facilities, improve ART adherence and retention, and decrease patients’ out‐of‐pocket healthcare expenditures. This randomized trial in Dar es Salaam aimed to assess whether community delivery of ART is non‐inferior to the standard of care in achieving viral suppression.

**Methods:** We randomized 48 healthcare facilities in Dar es Salaam to either the standard of care (facility‐based ART care) or CHW‐led ART community delivery. The CHW cadre is a long‐standing public sector cadre in Dar es Salaam, called home‐based carers. Patients had to be clinically stable on ART to be eligible for ART community delivery. The primary endpoint was the proportion of ART patients in viral failure (viral load >1,000 copies/ml) at the end of the study period. We fitted log‐binomial models clustering standard errors at the facility level and computed *p*‐values through randomization inference.

**Results: ** We recruited 1174 participants at intervention and 998 at control facilities. 521 participants received CHW‐led ART community delivery. Mean follow‐up was 321 days. 24.5% of participants in the intervention and 18.5% in the control arm were lost to follow‐up. The risk ratio (RR) for viral failure in the intervention compared to the control arm was 0.91 (95% CI: 0.72 to 1.12). The *p*‐value for the observed RR being below the margin of non‐inferiority (RR = 1.45) was <0.001. Participants’ healthcare expenditures over the last six months were not significantly different between intervention and control facilities.

**Conclusions: **Community delivery of ART by CHWs in Dar es Salaam did not result in inferior viral suppression compared to the standard of care. While ART community delivery did not reduce participants’ healthcare expenditures by a substantial amount, it is likely to save ART patients substantial amounts of time and may improve long‐term retention in care.

## Poster Discussion Abstracts

## TUPDA0101

### Association between immunogenetic factors and post‐treatment control of HIV‐1 infection. ANRS VISCONTI and PRIMO studies

**A. Essat^1^; D. Scott‐Algara^2^; V. Monceaux^3^; V. Avettand‐Fenoel^4,5^; C. Didier^2^; S. Caillat‐Zucman^6^; S. Orr^1^; I. Theodorou^6,7^; C. Goujard^1,8^; F. Boufassa^1^; O. Lambotte^8,9^; C. Rouzioux^4,5^; L. Hocqueloux^10^; L. Meyer^1,8^; A. Sáez‐Cirión^3^; ANRS PRIMO and VISCONTI studies**

^1^INSERM CESP U1018, University Paris Sud, Le Kremlin Bicêtre, France, ^2^Institut Pasteur, Unité régulation des infections rétrovirales, Paris, France, ^3^Institut Pasteur, Unité HIV inflammation et persistance, Paris, France, ^4^Université Paris Descartes, Sorbonne Paris Cité, EA7327 Paris, France, ^5^AP‐HP, Laboratoire de Virologie, CHU Necker‐Enfants Malades, Paris, France, ^6^Laboratoire d'Immunologie et Histocompatibilité Hôpital St. Louis, Paris, France, ^7^UPMC UMRS CR7 – Inserm U1135 – CNRS ERL 8255, Paris, France, ^8^APHP, Bicêtre Hospital, Univ Paris‐Sud, Le Kremlin Bicêtre, France, ^9^ImVA UMR 1184, Le Kremlin Bicêtre, France, ^10^CHR d'Orléans – La Source, Service des Maladies Infectieuses, Orléans, France

**Background:** Some HIV‐1 infected individuals durably control viremia after interruption of early ART (post‐treatment controllers, PTC). The mechanisms associated with this phenomenon are unclear. Prevalence of HLA‐B*35, associated with rapid progression without therapy, is high among PTC in the VISCONTI study. In contrast, HLA‐B*27/57 alleles, associated with HIV‐control, are not overrepresented in PTC. We investigated the association between HLA‐B*35 and HIV remission.

**Methods:** CD4+ T‐cell counts, plasma viral loads, PBMC‐associated HIV‐DNA levels were analysed in 245 HLA‐typed HIV‐infected individuals from the ANRS PRIMO cohort, on ART for at least 12 months since primary infection. HLA and KIR genotyping and NK cell analyses were done in PTC (n = 10) from the ANRS VISCONTI study. Results were compared to HIV‐infected (viremic, on cART, HIV‐controllers) and non‐infected individuals.

**Results: ** In the PRIMO cohort, despite similar time since infection, HLA‐B*35 individuals (B35, n = 64) had lower CD4+ T‐cell counts (median 398 cells/mm3, *p* = 0.04) and higher HIV‐DNA level (median 3.53 log10 HIV‐DNA copies/million PBMC, *p* = 0.046) than HLA‐B*27/57 (B27/57, n = 21, 543 cells/mm3 and 2.88 log10 HIV‐DNA copies) individuals or those carrying other HLAs (others, n = 160, 481 cells/mm3 and 3.44 log10 HIV‐DNA copies) at the time of treatment initiation. After a similar period on ART (approximately five years) the only difference was B27/57 having lower HIV‐DNA levels (median <1.7 log10 vs. 2.5 and 2.3 for B35 and others, *p* = 0.01). Among those interrupting the treatment, B35 were more likely to maintain viral control than B27/57 or others (*p* = 0.01). B35 who controlled HIV carried more frequently KIR ligands Bw4 or C2/C2 (f = 0.8, *p* = 0.04) than B35 not controllers (f = 0.39). In the VISCONTI study, PTC also carried often the Bw4 epitope (f = 0.85 vs. 0.44 in non‐infected controls, *p* = 0.02) and had high prevalence of the KIR B haplotype (f = 0.86 vs. 0.4). Phenotypical differences and increased anti‐HIV activity of NK cells were observed in PTC compared to other HIV‐infected individuals (*p* = 0.01).

**Conclusions: **Our results suggest that HLA‐B*35 might favour post‐treatment control, despite unfavourable primary HIV‐infection, in some early treated individuals. This might be associated with the presence of KIR Bw4 and C2 ligands in the MHC of PTC, enrichment of KIR B genotype and optimal licensing of NK cells.

## TUPDA0102

### HCV treatment with direct‐acting antivirals (DAAs) in HIV/HCV coinfected subjects affects the dynamics of the HIV‐1 reservoir

**Y. Ghiglione^1^; M.L. Polo^1^; A. Solomon^2^; G. Poblete^3^; M.J. Rolón^3^; P. Patterson^4^; H. Pérez^3^; H. Salomón^1^; F. Quiroga^1^; G. Turk^1^; S.R. Lewin^2,5^ and N. Laufer^1^**

^1^INBIRS Institute (UBA‐CONICET), Buenos Aires, Argentina, ^2^The University of Melbourne, Peter Doherty Institute, Melbourne, Australia, ^3^Hospital Fernández, Infectious Diseases Unit, Buenos Aires, Argentina, ^4^Huésped Foundation, Buenos Aires, Argentina, ^5^Alfred Health and Monash University, Department of Infectious Diseases, Melbourne, Australia

**Background:** The effect of direct acting antiviral (DAA) agents for hepatitis C virus (HCV) on the HIV reservoir in co‐infected individuals is not completely understood. We hypothesized that cure of HCV would reduce persistent interferon (IFN) signaling and would have downstream effects on the frequency of infected cells and their basal transcriptional activity.

**Methods:** Nineteen HIV/HCV coinfected individuals on suppressive ART were treated for HCV with sofosbuvir/daclatasvir ± ribavirine (12 to 24 weeks). Blood samples were obtained at enrollment, immediately before HCV treatment (baseline sample, BSL) and at end‐of‐treatment (EOT) for HCV. HIV‐monoinfected individuals on at least one year of ART were included as controls. Cell‐associated HIV DNA (total, integrated, 2LTR) and unspliced (US) and multiply spliced (MS) RNA were quantified by real‐time PCR. Data was analyzed using non‐parametric statistics. CD4 and CD8 cell frequency was determined by flow cytometry.

**Results: ** In the HIV/HCV coinfected group, 79% were male (mean age = 49 ± 5.9 years old). The mean times of HIV and HCV diagnosis were 16.2 ± 4.8 years and 13.5 ± 7.3 years, respectively. Median CD4+ T‐cell count at enrollment was 291 cells/μL (IQR 231 to 776). All were under cART with undetectable HIV VL. Among the control group, 82% were male, with median CD4+ T‐cell count = 612 cells/μL (IQR 476 to 863). US HIV RNA was significantly higher in CD4+ T‐cells collected at EOT compared to BSL (*p* = 0.01). HIV/HCV individuals had higher US HIV RNA at BSL (*p* = 0.09) and EOT samples (*p* = 0.005), compared to HIV‐only individuals, There were no statistically significant differences in HIV MS RNA, 2LTRs or total and integrated HIV DNA between EOT and BL or between HIV/HCV and HIV‐only individuals. At EOT compared to BSL, HIV/HCV subjects had significantly higher CD4/CD8 ratios (0.42 (IQR 0.29 to 0.71) versus 0.50 (IQR 0.31 to 0.76), *p* = 0.02). This was a result of an increase in CD4^+^ T‐cells but minimal change in CD8^+^ T‐cell counts.

**Conclusions: **HCV/HIV individuals showed higher levels of HIV unspliced‐RNA than HIV monoinfected individuals and this difference was even greater after HCV clearance with DAAs. Thus, HCV clearance, or related downstream effects impact HIV persistence on ART. These changes are consistent with reduced basal transcription in latently infected cells post‐HCV clearance which could be secondary to reduction in chronic IFN signaling.

## TUPDA0103

### IL‐10 contributes to, and is a biomarker for, viral persistence in ART‐treated, SIV‐infected rhesus macaques

**J. Harper^1^; S. Ribeiro^2^; M. Pino^1^; L. Micci^1^; M. Aid^2^; C. Deleage^3^; E. Rimmer^4^; G. Ayanoglu^4^; D. Gorman^4^; J. Estes^5^; R. Sekaly^2^ and M. Paiardini^1,6^**

^1^Emory University, Atlanta, United States, ^2^CASE Western Reserve University, Cleveland, United States, ^3^Frederick National Laboratory for Cancer Research, Frederick, United States, ^4^Merck Biologics Discovery, San Francisco, United States, ^5^Vaccine and Gene Therapy Institute, OHSU, Portland, United States, ^6^Yerkes Nationale Primate Research Center, Atlanta, United States

**Background:** One of the main barrier to designing therapeutic strategies aimed at targeting HIV persistence is our incomplete knowledge of the mechanisms regulating the establishment and maintenance of the HIV reservoir, as well as the lack of biomarkers able to predict its size. Interleukin (IL)‐10 is a critical component of the anti‐inflammatory responses required to dampen the pro‐inflammatory responses activated by the immune system after an encounter with a pathogen. IL‐10 signaling reduces antigen presentation and increases T cell anergy, and IL‐10 deficiency in mice prevented the persistence of pathogens normally inducing chronic infections. We hypothesized that IL‐10 can lead to a status of immunosenescence and favor HIV persistence.

**Methods:** Fifteen RMs were infected with SIVmac239 and started on cART at day 58 post‐infection. cART was maintained for seven months. Blood, lymph node (LN), and rectal biopsy (RB) were collected longitudinally for flow cytometric and DNAscope analyses. SIV‐DNA content was quantified in CD4 T cell subsets, including Tfh. RNAseq analyses were performed on PBMCs.

**Results: ** IL‐10 *in vitro* stimulation significantly upregulated pathways involved in HIV persistence, including the expression of pSTAT3, PD‐1 and CTLA‐4, and histone deacetylases. *In vivo*, (1) IL‐10 levels (plasma and LN) and IL‐10 stimulated genes increase upon SIV infection and do not fully normalize with ART (*p* < 0.01); (2) before ART, IL‐10 and IL‐10 stimulated genes levels significantly correlated with multiple markers of disease progression as well as the frequency of CD4^+^ T‐cells harboring SIV‐DNA in blood, LN (including Tfh cells), and gut (*p* < 0.01); (3) IL‐10 levels and IL‐10 stimulated genes at pre‐ART predict the frequency of LN CD4^+^ Tfh cells (*p* = 0.0002) and SIV‐DNA content in blood CD4 T cells (*p* = 0.0218) and in RB (*p* = 0.0383) after seven months of ART. Finally, among LN follicular SIV‐DNA^+^ cells, those IL‐10^+^ were more stable between the pre‐ and on‐ART time points than their IL‐10 negative counterpart, resulting in 85% of SIV‐DNA^+^ cells being IL‐10^+^ on‐ART (*p* = 0.0095).

**Conclusions: **Altogether, our data highlight IL‐10 signaling as a mechanism of and a biomarker for viral persistence in ART‐treated, SIV‐infected rhesus macaques. Modulation of IL‐10 represents a novel therapeutic avenue towards an HIV cure.

## TUPDA0104

### Follicular CD8+ T‐cells in gut‐associated lymphoid tissue are associated with lower HIV‐1 reservoir in the terminal ileum after ART initiated during primary HIV infection

**J.P. Thornhill^1^; G.E. Martin^2^; J. Hoare^1^; S. Peake^1^; M. Pace^2^; J. Meyerowitz^2^; J. Lwanga^3^; H. Lewis^1^; T. Solano^3^; C. Herrera^1^; J. Fox^3^; S. Fidler^1^; J. Frater^2^ and the CHERUB Investigators**

^1^Imperial College London, London, United Kingdom, ^2^University of Oxford, Oxford, United Kingdom, ^3^Guys and St Thomas’ NHS Trust, London, United Kingdom

**Background:** B cells follicles are considered a site of ongoing viral replication during treated HIV infection due to the exclusion of cytotoxic CD8 T‐cells. Recent work has demonstrated that follicular cytotoxic (CXCR5+CD8+) T‐cells have the ability to migrate to B cell follicles and are important for HIV control. We investigate the frequency and phenotype of CXCR5+CD8 T‐cells in gut‐associated lymphoid tissue (GALT) during treated primary HIV infection (PHI) and examine the association with HIV reservoir.

**Methods:** Gut biopsy samples from terminal ileum (TI) and rectum were collected from HIV+ virally suppressed individuals enrolled in HEATHER, an observational study of treated PHI. Comparisons were made with biopsies from healthy controls (HC). Expression of CD3, CD4, CD8, CXCR5, PD‐1, HLA‐DR, CD38, Perforin, Granzyme B and Bcl‐6 were assessed on gut and peripheral blood mononuclear cells (PBMC) cells by flow cytometry. Total HIV DNA was quantified by qPCR.

**Results: ** GALT samples were analyzed from 21 HIV+ and 8 HC individuals. Longitudinal (baseline & 1‐year) biopsies on ART were available for 10 individuals. CXCR5 + CD8+ T‐cells are found at significantly higher frequency in GALT compared to peripheral blood (mean 9.5% (95% CI 7.5 to 11.4%) versus 2.1% (1.2 to 2.9%) respectively, *p* < 0.001. The frequency of CD8 + CXCR5+ T‐cells was similar in HIV+ and HC GALT tissue (TI *p* = 0.76; rectum *p* = 0.06). In the HIV+ group, no change in CXCR5 + CD8+ T‐cells in GALT was noted between baseline and & one‐year; higher frequency of CXCR5 + CD8+ T‐cells was observed in rectal tissue compared to TI (*p* = 0.006). An inverse correlation between the frequency of CXCR5 + CD8 T‐cells and HIV‐1 DNA was observed in TI (r = −0.6, *p* = 0.01) but not in the rectum (r = −0.03, *p* = 0.9). CXCR5 + CD8+ T‐cell percentage correlated with CD4/CD8 ratio (TI r = 0.8, *p* < 0.0001; Rectum r = 0.5, *p* = 0.03) & T‐follicular helper cell frequency (TI: r = 0.5, *p* = 0.03; rectum: r = 0.5, *p* = 0.02). Higher expression of Bcl‐6, Perforin (*p* < 0.001), Granzyme B (*p* < 0.0001), HLA‐DR (*p* = 0.0003) and PD‐1 (*p* = 0.001) was observed on CXCR5 + CD8+ compared to CXCR5‐CD8 T‐cells in HIV+ GALT.

**Conclusions: **Follicular CD8+ T‐cells in HIV+ GALT persist on ART, exhibit greater cytotoxic potential than CXCR5‐CD8 T‐cells and may have a role in limiting HIV reservoir in terminal ileum during treated PHI.

## TUPDA0105

### HIV‐1 reservoir diversity and genetic compartmentalization in blood and testis

**R. Ponte^1,2^; R.L. Miller^3^; N.N. Kinloch^3^; F.H. Omondi^3^; F.P. Dupuy^1,2^; R. Fromentin^4^; P. Brassard^5^; V. Mehraj^1,2^; N. Chomont^4^; A. Poon^6^; Z.L. Brumme^3,7^; J.‐P. Routy^1,2^ and The ORCHID study group**

^1^The Research Institute of the McGill University Health Centre, Montreal, Canada, ^2^Chronic Viral Illness Service, Montreal, Canada, ^3^Faculty of Health Sciences, Simon Fraser University, Burnaby, Canada, ^4^CR‐CHUM and Université de Montréal, Montréal, Canada, ^5^The Metropolitan Centre for Plastic Surgery, Montreal, Canada, ^6^Western University, Department of Pathology and Laboratory Medicine, London, Canada, ^7^BC Centre for Excellence in HIV/AIDS, Vancouver, Canada

**Background:** HIV latency is the main barrier to cure, but our knowledge of the genetic landscape of latent HIV genomes, particularly in immune‐privileged sites, remains incomplete. Knowing that the testes constitute such a site, we characterize latent HIV diversity in testes and blood in HIV‐infected individuals with suppressed viremia on cART.

**Methods:** PBMC and right/left testicular tissue samples were collected from eight adults undergoing sex reassignment surgery who had maintained viremia suppression on cART for >6 months. HIV proviral Nef sequences were obtained via single‐genome amplification. Phylogenies were reconstructed by maximum‐likelihood (RAxML; GTR substitution model). PBMC HIV DNA levels were measured by qPCR; HIV adaptation to host HLA (determined via sequence‐based typing) was inferred bioinformatically. Genetic compartmentalization was assessed using Wright's measure of population subdivision (FST) and Slatkin‐Maddison (SM) tests.

**Results: ** 377 Nef sequences were isolated from PBMC (n = 235) and testes (n = 142); of these 212/235 (90.2%) and 133/142 (93.7%) were intact/non‐hypermutated (median 24 (IQR 21 to 33) PBMC and 8 (IQR 4 to 37) testis sequences/participant). Within‐host proviral diversity varied markedly (median within‐host patristic distance 2.06e^−2^ (IQR 8.30e^−3^ to 5.43e^−2^) substitutions/site), and total HIV DNA correlated with proviral diversity in PBMC (Spearman rho = 0.77, *p* = 0.1). In some participants a single HIV variant dominated, while others harbored predominantly unique sequences. Proviral diversity in PBMC generally exceeded that in testis, but not significantly so (median within‐host patristic distance 1.56e^−2^ vs. 1.26e^−2^ respectively, *p* = 0.4); inferred HIV immune escape burden was also consistent between sites. Both FST and SM tests identified four of eight participants as having significant HIV genetic compartmentalization between blood and testis (FST scores = 0.89, 0.67, 0.29 and 0.22; all *p* < 0.05; SM all *p* < 0.05).

**Conclusions: **Latent HIV diversity varies widely between individuals; larger reservoirs tend to be more diverse. Detection of identical HIV sequences in blood and testis is consistent with migration of clonally‐expanded descendants of latently HIV‐infected cells throughout the body, including into immune‐privileged sites. Detection of significant genetic compartmentalization between blood and testis in 50% of participants underscores the complexity of the proviral HIV landscape on cART, yet suggests that, at least in some individuals, blood proviral diversity may not be unrepresentative of overall reservoir diversity.

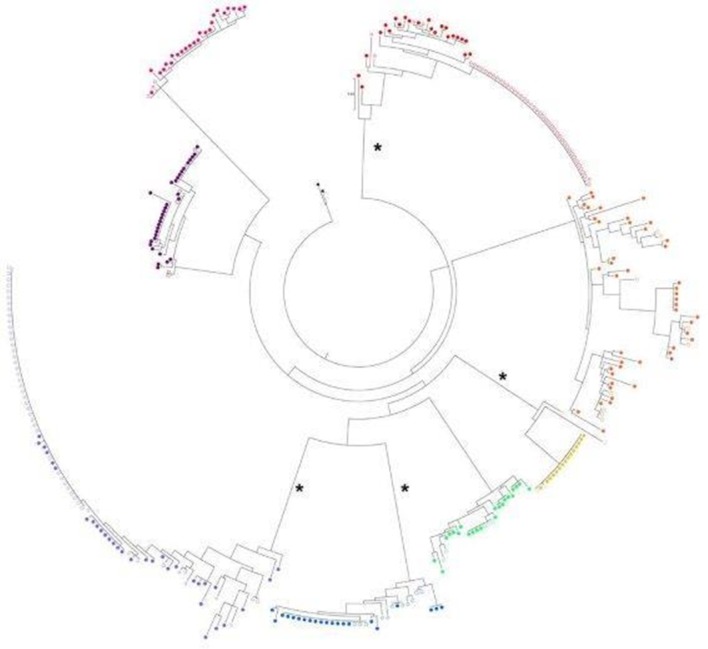

**Abstract TUPDA0105‐Figure 1. Maximum likelihood phylogeny of PBMC and testis sequences.**

## TUPDB0101

### Persistent immune activation and depression in rural Ugandans initiating antiretroviral therapy

**J. Chang^1,2,3^; N. Musinguzi^4^; A. Tsai^1,5,6^; C. Muzoora^4^; M. Bwana^4^; Y. Boum^7^; R. Tracy^8^; J. Martin^9^; J. Haberer^1,6,10^; D. Bangsberg^11^; P. Hunt^12^ and M. Siedner^1,6,13^**

^1^Massachusetts General Hospital, Center for Global Health, Boston, United States, ^2^Duke University School of Medicine, Durham, United States, ^3^University of North Carolina, Chapel Hill, United States, ^4^Mbarara University of Science and Technology, Mbarara, Uganda, ^5^Massachusetts General Hospital, Chester M. Pierce, MD Division of Global Psychiatry, Boston, United States, ^6^Harvard Medical School, Boston, United States, ^7^Epicentre, Mbarara, Uganda, ^8^University of Vermont, Department of Pathology & Laboratory Medicine, Burlington, United States, ^9^University of Scan Francisco, Department of Epidemiology and Biostatistics, San Francisco, United States, ^10^Massachusetts General Hospital, Department of Medicine, Boston, United States, ^11^Oregon Health & Science University, Portland, United States, ^12^University of California, Department of Medicine, San Francisco, United States, ^13^Massachusetts General Hospital, Division of Infectious Diseases, Boston, United States

**Background:** Decreases in kynurenine/tryptophan (KT) ratio have been associated with decreases in depressive symptoms after initiation of antiretroviral therapy (ART); however, the relationship between depression and other markers of immune activation in persons with HIV (PWH) is less clear.

**Methods:** We analyzed data from 393 adult PWH in rural Uganda who were enrolled at ART initiation and observed every three to four months from 2005 to 2015. Our exposures of interest were change in levels of soluble CD14 (sCD14), KT ratio, soluble CD163 (sCD163), D‐dimer, and interleukin‐6 (IL‐6) from before to six months after ART initiation. Our outcome of interest was probable depression, defined by a mean score >1.75 on the Hopkins Symptom Checklist depression subscale. We fit modified Poisson regression models and generalized estimating equations (GEE) Poisson regression models with cluster‐correlated robust standard errors to respectively examine the relationship between: (1) pre‐ART inflammatory marker levels and depression; and (2) six‐month change in marker levels and depression at follow‐up visits within two years of ART initiation. Models were adjusted for pre‐ART depression, year of ART initiation, and tuberculosis co‐infection; and time‐varying demographic characteristics, body mass index, smoking, alcohol use, CD4+ count, ART duration, viral suppression (≤400 copies/mL) and self‐reported physical health.

**Results: ** There were no statistically significant associations between levels of inflammatory markers and depression in the pre‐ART period. However, in multivariable‐adjusted GEE regression models, larger decreases in sCD14 and KT ratio from pre‐ART to six‐months post‐ART were associated with decreased risk of depression (Table 1, Figure 1). These associations remained qualitatively similar when additionally adjusted for six‐month change in the level of the other marker and when restricted to follow‐up visits where the participant was virally suppressed. We estimated no statistically significant associations between sCD163, D‐dimer, or IL‐6 and risk of depression.

**Conclusions: **Greater decreases in sCD14 and KT ratio after ART initiation were independently associated with decreased risk of depression. This may suggest independent sCD14 and KT‐mediated pathways leading to depression in PWH and may be targets of future intervention.

**Abstract TUPDB0101‐Table 1. Association of quartile of log10 inflammatory marker level change from pre‐ART to 6‐months post‐ART and probable depression in first two years**

**Table:** 

Marker	Quartile	Unadjusted IRR (95% CI)	*p*‐value for joint test	Adjusted IRR (95% CI)	*p*‐value for joint test
sCD14	First (smallest decrease)	REF	0.005	REF	0.001
	Second	1.10 (0.68, 1.78)		1.01 (0.68, 1.50)	
	Third	0.44 (0.25, 0.76)		0.44 (0.27, 0.69)	
	Fourth (largest decrease)	0.75 (0.45, 1.24)		0.63 (0.42, 0.97)	
KT ratio	First (smallest decrease)	REF	<0.001	REF	0.008
	Second	0.62 (0.39, 0.99)		0.67 (0.45, 0.98)	
	Third	0.41 (0.25, 0.66)		0.58 (0.38, 0.89)	
	Fourth (largest decrease)	0.39 (0.24, 0.66)		0.48 (0.30, 0.77)

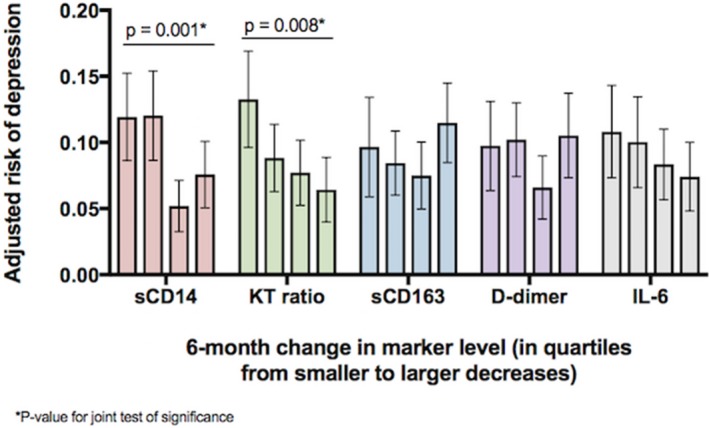

**Abstract TUPDB0101‐Figure 1**

## TUPDB0102

### Variables associated with neuropsychiatric symptoms in PLWH receiving dolutegravir based therapy in phase III clinical trials

**J. van Wyk^1^; J. Oyee^2^; S. Barthel^2^; J. Koteff^3^; B. Wynne^3^; L. Curtis^2^; V. Vannappagari^3^; N. Payvandi^1^; V. Carr^1^; T. Vincent^1^ and M. Aboud^1^**

^1^ViiV Healthcare, Brentford, United Kingdom, ^2^GlaxoSmithKline, Stockley Park, United Kingdom, ^3^ViiV Healthcare, Research Triangle Park, United States

**Background:** Analysis of phIII clinical trials for DTG concluded that selected neuropsychiatric symptoms (NPs) occurred at similar frequencies compared with controls. Some observational cohort data suggest that NPs result in higher rates of discontinuation among DTG users. Potential factors associated with discontinuations due to NPs reported in some cohorts include ABC co‐administration, older age and female gender. We performed a meta‐analysis to assess variables associated with NPs, and explored whether insomnia was associated with subsequent NPs.

**Methods:** Studies included: SPRING‐2, SINGLE, FLAMINGO, ARIA, SAILING. NPs included: Insomnia, anxiety, depression, suicidality, nightmares/abnormal dreams, headache. Exposure adjusted incidence of NPs was calculated from frequencies of reported adverse events (AEs); 95% CIs are based on exact binomial two‐sided CIs. Poisson mixed effects meta‐regression models were used to conduct two analyses of pre‐specified variables in a backward selection on the incidence rate of AEs in patients treated with A) DTG (N = 1672) versus nonDTG (n = 1681) and B) DTG + ABC (n = 943) versus DTG + nonABC (n = 729). Significance level was 10%. Insomnia as a precursor to other NPs was analyzed descriptively.

**Results: ** Identified variables associated with NPs are shown in the figure. Overall, adjusted estimates (SE) for NPs rates per 1000 person years were 5.26 (0.068) with DTG versus 5.21 (0.07) with nonDTG (aRR 1.05 (95% CI 0.9, 1.21, *p* = 0.55)), and 5.4 (0.079) with DTG + ABC versus 5.3 (0.085) with DTG + nonABC (aRR 1.1 (95% CI 0.89, 1.37, *p* = 0.37)). Descriptive analyses of first insomnia events and subsequent non‐insomnia events are in the table. First insomnia events with subsequent non‐insomnia NPs occurred infrequently (2.2%), with higher rates of first occurrence of non‐insomnia NPs (23%).

**Conclusions: **In this meta‐analysis including 3353 participants, the rate of NPs was similar between DTG and non‐DTG treated patients. Variables associated with increased NPs in the DTG versus nonDTG analysis were past psychiatric history, non‐EU residence and, in contrast with previous findings, younger age. Within the DTG versus DTG + ABC analysis, past psychiatric history and country of residence showed significant association. Concomitant ABC use was not a variable associated with NPs. There was no indication that insomnia was associated with subsequent CNS.

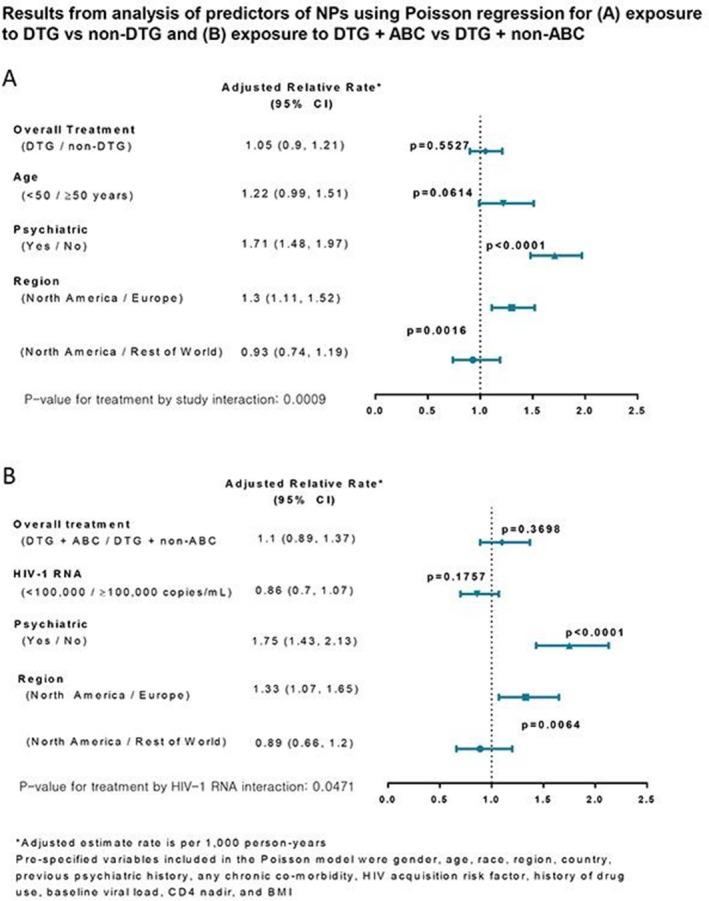

**Abstract TUPDB0102‐Figure 1**

**Abstract TUPDB0102‐Table 1**

**Table:** 

	DTG (N = 1672) n (%)	nonDTG (N = 1681) n (%)	Difference, % (95% CI)
Patients without NPs	1242 (74.3%)	1273 (75.7%)	−1.45 (−4.4, 1.5)
First insomnia event with subsequent non‐insomnia NPs	37 (2.2%)	25 (1.5%)	0.73 (−0.2, 1.6)
Non‐insomnia NPs without prior insomnia event	384 (23.0%)	378 (22.5%)	0.48 (−2.4, 3.3)
Only one NPs event	310 (18.5%)	278 (16.5%)	2.00 (−0.6, 4.6)

## TUPDB0103

### Antiretroviral therapy (ART) interruption is associated with reduced cortical structures compared to uninterrupted ART at age 5 years in HIV‐infected children on early ART

**E.C. Nwosu^1^; M.J. Holmes^1^; M.F. Cotton^2^; E. Dobbels^2^; F. Little^3^; B. Laughton^2^; A. van der Kouwe^4,5^; E.M. Meintjes^1^ and F.C. Robertson^1^**

^1^University of Cape Town, MRC/UCT Medical Imaging Research Unit, Division of Biomedical Engineering, Department of Human Biology, Faculty of Health Sciences, Cape Town, South Africa, ^2^Stellenbosch University, Family Clinical Research Unit, Department of Paediatrics & Child Health, Tygerberg Children's Hospital and Faculty of Health Sciences, Cape Town, South Africa, ^3^University of Cape Town, Department of Statistical Sciences, Faculty of Sciences, Cape Town, South Africa, ^4^Massachusett General Hospital, A.A. Martinos Centre for Biomedical Imaging, Department of Radiology, Charlestown, United States, ^5^Harvard Medical School, Department of Radiology, Boston, United States

**Background:** ART interruption (ATI) has been studied in HIV‐infected (HIV+) children and adolescents and may occur due to poor adherence, stock‐outs and ART intolerance. Although ATI in early treated children may not affect immune health, neurocognition and quality of health in the short term, its effect on brain development is not clear. Here, we investigated effect of ATI on brain morphometry ‐ cortical thickness (CT) and local gyrification indices (LGIs) ‐ in healthy 5 year‐old children who initiated ART before 18 months of age.

**Methods:** MRI scans were acquired from participants in the Children with HIV early antiretroviral therapy (CHER) trial follow‐on study according to protocols approved by the ethics committees of the Universities of Cape Town and Stellenbosch. FreeSurfer software v6.0 (http://freesurfer.net/) was used for automated reconstruction and segmentation. Whole‐brain CT and LGIs ‐ a measure of cortical folding ‐ were compared between HIV+ children and uninfected controls (HIV‐), and between children with ATI who restarted when CD4% < 25% or CDC severe Stage B and children on continuous ART using a linear regression model controlling for sex, age at scan and age at ART initiation.

**Results: ** Forty‐six HIV+ children (24 ART‐interrupted ‐ interruption age (median ± IQR = 49.14 ± 36.18 weeks), ART initiation ‐ median ± IQR = 9.14 ± 2.93 weeks, 22 ART‐uninterrupted, ART initiation ‐ median ± IQR = 11.86 ± 22.14 weeks) and 18 age‐matched uninfected controls (9 boys) (age: mean ± std. = 5.58 ± 0.31 years) were included. HIV+ children showed thicker cortex than controls in bilateral frontal and post central regions and lower gyrification in bilateral anterior cingulate, superior parietal and left superior frontal regions. ATI had thinner cortex than continuous therapy in a left lateral occipital (cluster size: 812.30 mm^2^) region. ATI showed lower gyrification than continuous therapy in bilateral superior parietal (cluster size: left ‐ 2970.70 mm^2^, right ‐ 865.97 mm^2^) regions (figure 1).

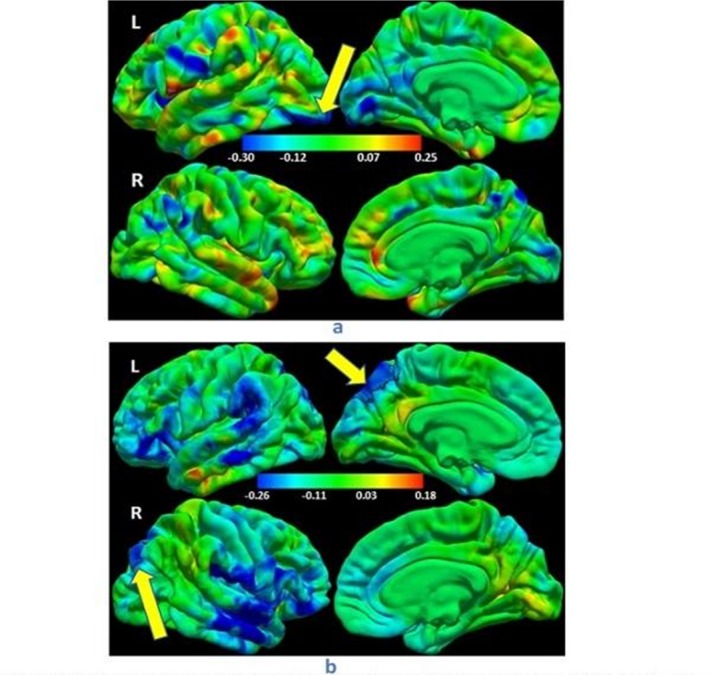

**Abstract TUPDB0103‐Figure 1.**

**Conclusions: **ART interruption at a young age may affect the development of cortical structures, leading to parietal gyrification decrease and occipital cortical thinning at age 5 years. The neuropsychological implications of these effects in HIV+ children requires further investigation.

## TUPDB0104

### Ongoing white matter alterations in HIV infected and HIV exposed children: a DTI study at 9 years

**F. Mberi^1^; M. Jankiewicz^1,2^; F. Little^3^; M. Cotton^4^; A. van der Kouwe^1,5,6^; B. Laughton^4^; E. Meintjes^1,2^ and M. Holmes^1^**

^1^University of Cape Town, Department of Human Biology, Faculty of Health Sciences, Cape Town, South Africa, ^2^University of Cape Town, Cape Universities Body Imaging Centre at UCT, Faculty of Health Sciences, Cape Town, South Africa, ^3^University of Cape Town, Department of Statistical Sciences, Cape Town, South Africa, ^4^University of Stellenbosch, Family Clinical Research Unit, Department of Paediatrics & Child Health, Tygerberg Children's Hospital and Faculty of Health Sciences, Cape Town, South Africa, ^5^Massachusetts General Hospital, A.A. Martinos Centre for Biomedical Imaging, Department of Radiology, Charlestown, United States, ^6^Harvard University, Department of Radiology, Boston, United States

**Background:** An increasing number of perinatally HIV‐infected (HIV+) infants are growing up on ART. Examination of diffusion tensor imaging (DTI) measures over time can identify the impact of HIV exposure, infection and/or treatment on white matter (WM) maturation. DTI studies report WM regions with reduced fractional anisotropy (FA) and increased mean diffusivity (MD) in HIV+ children on ART, which point to demyelination and/or axonal damage.

In the Children with HIV Early Antiretroviral therapy (CHER) substudy, we reported persistent WM abnormalities in the *inferior/superior longitudinal fasciculus* (ILF/SLF), *inferior fronto‐occipital fasciculus* (IFOF), *forceps minor* and *corticospinal tract* (*CST)* at ages 5 and 7, despite early ART. Here, we further investigate voxelwise group differences of MD and FA in this cohort at age 9.

**Methods:** Participants are 51 HIV+ and 36 HIV‐ children (42 Females; mean age±sd: 9.4±0.4; 11 Cape Coloured/76 Xhosa) scanned in Cape Town, South Africa on a 3T Siemens Skyra MRI scanner (Erlangen, Germany).

Structural T1‐weighted images and two DWI sets with opposite phase encodings were acquired. Data were processed using TORTOISE (version 3.1.0) and AFNI. Voxelwise group comparison of FA and MD were performed in FSL using a general linear model (GLM) with gender, age and ethnicity as confounders. Axial diffusivity (AD), and radial diffusivity (RD) were extracted for each subject in surviving clusters (threshold *p*th=0.005 and *α*=0.05) and compared between groups using a student's t‐test.

**Results: ** HIV+ children demonstrated lower FA in bilateral CST, right IFOF and *corpus callosum* (CC) (Fig 1 and Table 1), and higher MD bilaterally in CST and ILF, left IFOF and right *anterior thalamic radiation* (ATR). Differences were largely attributable to higher RD.

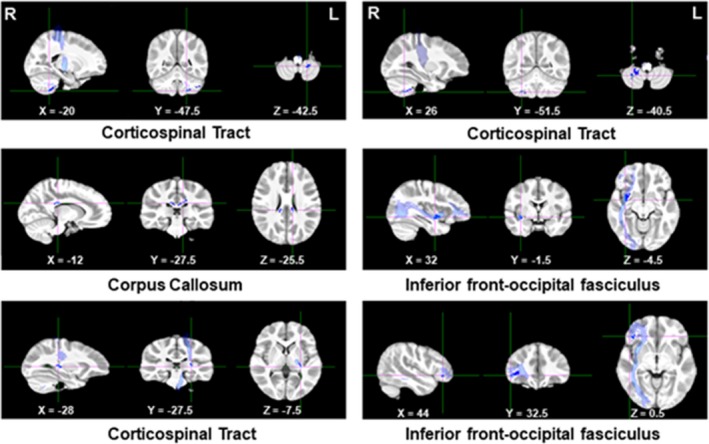

**Abstract TUPDB0104‐Figure 1. Clusters showing structural abnormalities between HIV‐ and HIV+ children.**

**Abstract TUPDB0104‐Table 1. Clusters showing significant differences in FA between HIV‐ and HIV+ children**

**Table:** 

Cluster location	Size	FA		AD			RD		
	(mm3)	HIV‐	HIV+	HIV‐	HIV+	*p*	HIV‐	HIV+	*p*
Corticospinal Tract (L)	528	0,490 (0.095)	0.405 (0.100)	1.023 (0.088)	1.001 (0.106)	0.315	0.448 (0.067)	0.527 (0.116)	<0.001
Corticospinal Tract (R)	424	0.409 (0.080)	0.340 (0.068)	0.984 (0.071)	0.982 (0.081)	0.862	0.517 (0.066)	0.594 (0.086)	<0.001
Corpus Callosum	384	0.613 (0.130)	0.487 (0.156)	1.345 (0.114)	1.258 (0.119)	0.001	0.423 (0.113)	0.536 (0.140)	<0.001
Inferior fronto‐occipital fasciculus (R)	336	0.289 (0.093)	0.224 (0.061)	1.023 (0.094)	0.975 (0.065)	0.005	0.651 (0.058)	0.694 (0.047)	<0.001
Corticospinal Tract (L)	320	0.552 (0.059)	0.486 (0.075)	1.211 (0.065)	1.171 (0.079)	0.013	0.457 (0.043)	0.508 (0.046)	<0.001
Inferior fronto‐occipital fasciculus (R)	248	0.356 (0.115)	0.269 (0.110)	1.003 (0.104)	0.970 (0.086)	0.109	0.579 (0.091)	0.648 (0.100)	0.001
Units of AD, and RD are 10−3 mm2 s−1.									

**Conclusions: **Our results indicate that despite early ART, persistent WM alterations in the IFOF, ILF and CST are present from age 5 to 9. Furthermore, although we did not observe CC abnormalities at 5 or 7 years, microstructural abnormalities in CC at 9 years suggest early ART may not prevent ongoing damage.

## TUPDB0105

### Incidence of stroke in HIV‐positive patients: a population‐based study in Taiwan

**H.‐L. Lin^1,2^ and P.‐C. Chen^3^**

^1^China Medical University, PHD Program for Aging Program, Taichung, Taiwan, Province of China, ^2^Linshin Hospital, Rehabilitation, Taichung, Taiwan, Province of China, ^3^China Medical University, Public Health, Taichung, Taiwan, Province of China

**Background:** Few studies evaluated whether people infected with human immunodeficiencyvirus (HIV) are at an increased risk of stroke in Asian population. This study investigated incidence of stroke in people with HIV infection in comparison with general population in Taiwan.

**Methods:** Using the claims data of a universal health insurance program, we identified5,961 HIV/AIDS patients without previous stroke from 1998 to 2005 and followed them up until 2011. Standardized incidence ratios (SIRs) were calculated to compare the incidence of overall, ischemic and hemorrhage stroke in HIV/AIDS patients with that in the general population by age, sex and duration of follow‐up.

**Results: ** HIV/AIDS patients had higher risk of developing stroke overall (SIR 1.94, 95% confidence interval (CI) 1.58 to 2.35), ischemic stroke (SIR 2.28, 95% CI 1.74 to 2.95) and hemorrhage stroke (SIR 2.09, 95% CI 1.40 to 3.00). The risk remained consistently and significantly higher among all age and sex groups. In all patients and each of age and sex stratifications, the SIR was the highest within one year after diagnosis of HIV/AIDS (SIR (95% CI) for all patients, all stroke, 46.62 (7.63 to 73.68); ischemic stroke, 34.47 (13.86 to 71.02); hemorrhage stroke, 81.92 (30.06 to 178.30)).The risk diminished over time, and no increased risk was observed after eight years of follow‐up.

**Conclusions: **HIV is associated with the highest risk of developing overall, ischemic and hemorrhagic stroke within 1 year after diagnosis among any age groups. This finding may highlight the importance of screening and correcting risk factors for stroke immediately and aggressively after patients diagnosed with HIV and AIDS.

## TUPDB0106

### Longitudinal neurocognitive performance in HIV infected individuals in rural Uganda

**K. Robertson^1^; A. Vecchio^2^; D. Saylor^3^; G. Nakigozi^4^; N. Nakasujja^5^; R. Gray^6^; M. J. Wawer^6^; N. Sacktor^7^ and Rakai Health Sciences Program**

^1^University of North Carolina, Neurology, Durham, NC, United States, ^2^Viata‐Salute San Raffaele University, Infectious Diseases, Milan, Italy, ^3^Johns Hopkins, Neurology, Baltimore, United States, ^4^Rakai Health Sciences Program, Kalisizo, Uganda, ^5^Makerere University, Kampala, Uganda, ^6^Johns Hopkins School of Public Health, Baltimore, United States, ^7^Johns Hopkins University, Neurology, Baltimore, United States

**Background:** Neurocognitive impairment in HIV+ individuals remains prevalent despite effective antiretroviral therapy (ART). There is limited data on longitudinal neurocognitive performance in HIV+ individuals in rural Sub‐Saharan Africa. By implementing neurocognitive exams for both HIV+ and HIV− individuals, we can establish normative data and monitor neurocognitive function in HIV+ persons in an ongoing study in rural Rakai District, Uganda.

**Methods:** Participants were enrolled in the Rakai Community Cohort Study (400 HIV‐, 400 HIV + ART naïve). Personnel were trained to administer standardized neurological and neuropsychological assessments, and participants were stratified by demographic features (age, education, and gender). The HIV+ participants initiated on ART had a follow‐up exam to assess neurological function over time.

**Results: ** At baseline there was a significant difference in neurocognitive performance between HIV− (total z score M −0.01, SD 0.50) and HIV+ (M −0.26, SD 0.72, *p* < 0.0001). The 333 HIV+ participants who returned for a follow‐up exam after two years had a mean age of 37.4 years (SD 8.6), education of 5.5 years (SD 3.3), and 49% were women. There was a significant improvement in the total neurocognitive performance of the HIV+ participants initiated on ART within the two year follow‐up period (F (1332) = 9.88, *p* < 0.005; mean time1 = −0.26; time 2 = −0.16). There was Improved performance in the neurocognitive domains of Fine motor Learning, and Memory (*p* < 0.0001), while there was no change in Executive functioning, Gross motor, or Speed of processing. The performance in the domains of Fluency and Attention decreased (*p* < 0.008). Overall neurocognitive test deficits (<1 SD) improved over time (F (1, 332) = 26.86, *p* < 0.0001); time1 = 3.03, time2 = 2.39).

**Conclusions: **Over the course of two years on ART, HIV+ participants in this rural, resource limited cohort showed an overall improvement in neurological performance. While there were substantial improvements in learning, memory, and fine motor abilities, there was not the expected improvement in executive functioning, gross motor, speed, fluency, and attention. Overall, neurocognitive test deficits significantly improved over time and warrant further follow‐up.

## TUPDB0107

### Integrase inhibitors and neuropsychiatric adverse events in a large prospective cohort

**L. Cuzin^1,2^; P. Pugliese^3^; C. Katlama^4^; I. Ravaux^5^; F. Bani‐Sadr^6^; T. Ferry^7^; D. Rey^8^; J. Lourenco^9^; S. Bregigeon^5^; C. Allavena^10^; J. Reynes^11^ and Dat'AIDS Study Group**

^1^CHU de Martinique, Infectious Diseases, Schoelcher, Martinique, ^2^INSERM UMR 1027, Toulouse, France, ^3^Nice University Hospital, Nice, France, ^4^Pitié Salpetrière University Hospital, Paris, France, ^5^Marseille University Hospitals, Marseille, France, ^6^Reims Universtiy Hospital, Reims, France, ^7^Lyon University Hospital, Lyon, France, ^8^Strasbourg University Hospital, Strasbourg, France, ^9^Cochin University Hospital, Paris, France, ^10^Nantes University Hospital, Nantes, France, ^11^Montpellier University Hospital, Montpellier, France

**Background:** In 2016 an unexpectedly high frequency of dolutegravir (DTG) discontinuation for neuropsychiatric reasons was reported, these effects might be more frequent when DTG was used with abacavir (ABC), in women, or in ageing people. Our objective was to search in our large prospectively collected cohort the patients who were treated with an integrase inhibitor (INSTI) and to analyze the frequency and causes of discontinuation.

**Methods:** The Dat'AIDS cohort is prospectively collected in 18 HIV reference centers in France. Data for all patients starting an INSTI containing regimen between 01 January 2006 and 31 December 2016 were extracted. All causes – chosen by the physician in a limited list of items – of an INSTI containing regimen discontinuation were analyzed, and patients’ characteristics related with discontinuation due to neuropsychiatric side effects were searched for.

**Results: ** INSTI were prescribed to 21,315 patients: 6274 treated with DLT, 3421 with elvitegravir boosted by cobicistat (EVG/c), and 11,620 with raltegravir (RAL), see Table 1. Discontinuation was observed in 12.5%, 20.2% and 50.9% of the DTG, EVG/c, and RAL treated patients, respectively (*p* < 0.001). The main reason for DTG and EVG/c discontinuation was intolerance (respectively 7.1% and 9.4% of the patients, *p* < 0.001). For RAL, treatment simplification (18.7%) was the leading reason. Discontinuation for neuropsychiatric reasons was described in respectively 2.7%, 1.3% and 1.7% of the DTG, EVG/c and RAL treated patients (*p* < 0.001). In multivariate analysis, discontinuation for a neuropsychiatric reason was related to DTG – versus EVG/C (HR = 2.27; 95% CI 1.63 to 3.17; *p* < 0.0001) and versus RAL (HR = 2.46; 95% CI 2.00 to 3.40; *p* < 0.0001), while neither gender (HR for women = 1.19; 95% CI 0.97 to 1.46; *p* = 0.09) nor age (*p* = 0.12) were related. The association with abacavir was not retained in the final model, due to a confusion factor, most of the patients treated by DTG receiving ABC whereas none of the patients treated by EVG/c and few of those treated by RAL did.

**Conclusions: **Although discontinuation for side effects was less frequent with DTG than with EVG/c, neuropsychiatric side effects were more frequent with DTG, but still remained rare (2.7%). No patient's characteristic could be related with these side effects in this very large population.

**Abstract TUPDB0107‐Table 1Patients characteristics at the time of INSTI initiation**

**Table:** 

		DLT N = 6274	EVG/c N = 3421	RAL N = 11,620
Gender (% women)		28.5	26.7	29.7
Age (years)	<40	25.6	40.8	23.1
	40 to 50	30.9	34.0	38.8
	50 to 60	28.3	18.8	25.6
	>60	15.3	6.4	12.5
Associated NRTI	ABC	66.4	0	17.6
	TDF	19.5	100	43.2
	Other/none	14.1	0	39.2
Lenght of known infection	Median (25 to 75IQ)	12 (4 to 20)	7 (1 to 16)	14 (7 to 20)

## TUPDC0101

### Mortality differences after ART initiation in HIV‐positive women from Europe, the Americas and sub‐Saharan Africa 2000 to 2014

**I. Jarrin; Global Mortality Disparities in Women Working Group for IeDEA; EuroSIDA; CASCADE and COHERE in EuroCoord**

Instituto de Salud Carlos III, Centro Nacional Epidemiologia, Madrid, Spain

**Background:** Women account for nearly half of all persons living with HIV/AIDS globally. We aimed to describe geographic disparities in overall mortality after antiretroviral therapy (ART) initiation among HIV‐positive women worldwide.

**Methods:** We pooled data from HIV‐positive women over 18 years of age enrolled from 2000 through 2014 and initiating ART within the following cohort collaborations: CASCADE, COHERE, EuroSIDA and six regions of IeDEA. Data‐contributing regions were categorized as Europe (Eur), East Africa (EAf), West Africa (WAf), South Africa (SAf), South America (SAm), North America (NAm) and Central America and the Caribbean (CAmCRB). Only in NAm were patients required to have a second visit within 12 months of enrollment. Linkages with mortality registries were reported in SAf, NAm and some sites in Eur, and sample tracing of losses to follow‐up was conducted in EAf. Inflation factors using mortality ascertainment data from EAf were used to correct mortality under‐reporting in WAf. Mortality rates were calculated by region at intervals 0 to 3, 3 to 6, 6 to 12, 12 to 24 and 24 to 48 months on ART, and mortality rate ratios estimated at each interval compared to Eur using a piecewise exponential parametric survival model fit through Poisson regression adjusted for age, CD4 T‐cell count and period of ART initiation.

**Results: ** A total of 190,175 women (16% Eur, 47% EAf, 13% WAf, 19% SAf, 1% SAm, 3% NAm and 2% CAmCRB) were included. Median age at ART initiation ranged from 33 years in SAf to 40 years in NAm. The proportion of injecting drug users was highest in NAm (18%) and Eur (7%). Only 16% of the women in NAm were of white ethnicity, while 63% and 17% were Black and Hispanic, respectively. Ethnicity data were available for 45% of European women, 26% of whom were Black, largely migrants. Median CD4 count at ART initiation was close to 250 cells/mm^3^ in Eur and NAm, 141 cells/mm^3^ in SAf and 170 to 190 cells/mm^3^ in other regions. Crude mortality rates and adjusted mortality rate ratios are presented in the figure and table, respectively.

**Conclusions: **Global variations in mortality in HIV‐positive women starting ART show distinct geographical patterns for one‐year and four‐year mortality that may inform context‐specific interventions.

**Abstract TUPDC0101‐Table 1. Adjusted mortality rate ratios (95% CI) compared to Europe by duration on ART**

**Table:** 

	Duration on ART (months)				
	0 to 3	3 to 6	6 to 12	12 to 24	24 to 48
East Africa	7.25 (5.87 to 8.97)	4.24 (3.30 to 5.46)	4.24 (3.35 to 5.37)	3.89 (3.21 to 4.72)	3.63 (3.04 to 4.33)
West Africa	8.95 (7.34 to 10.91)	4.05 (3.21 to 5.11)	4.38 (3.54 to 5.43)	5.37 (4.54 to 6.35)	5.61 (4.84 to 6.51)
South Africa	5.42 (4.43 to 6.64)	3.15 (2.50 to 3.97)	3.77 (3.05 to 4.67)	3.05 (2.56 to 3.63)	3.47 (2.97 to 4.06)
South America	4.47 (2.97 to 6.72)	1.67 (0.84 to 3.32)	2.70 (1.62 to 4.52)	1.69 (1.02 to 2.78)	2.42 (1.65 to 3.55)
North America	0.88 (0.53 to 1.44)	1.25 (0.78 to 2.02)	2.30 (1.61 to 3.27)	3.40 (2.64 to 4.37)	3.72 (2.97 to 4.65)
Central America and Caribbean	9.92 (7.79 to 12.63)	4.12 (2.95 to 5.74)	2.93 (2.07 to 4.15)	2.43 (1.82 to 3.26)	2.50 (1.92 to 3.26)

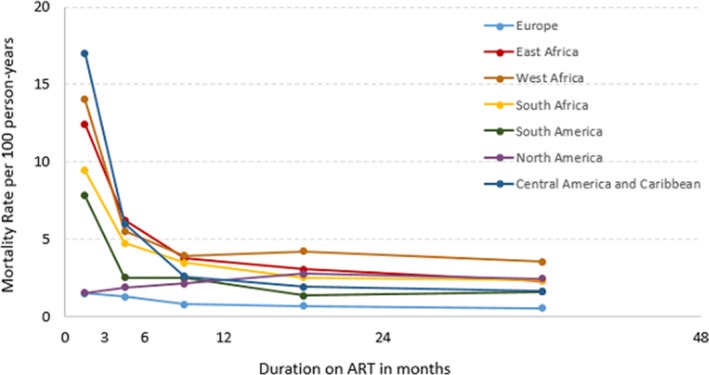

**Abstract TUPDC0101‐Figure 1. Mortality rates per 100 person‐years by time since ART initiation**

## TUPDC0102

### High mortality among women living with HIV enrolled in Canada's largest community‐based cohort study

**A. Kaida^1^; R. Gormley^1,2^; K. Webster^1^; A. Carter^1,2^; V. Nicholson^1,3^; L. Wang^2^; P. Sereda^2^; R. Hogg^1,2^; A. de Pokomandy^4,5^; M. Loutfy^6,7^ and on behalf of the CHIWOS Research Team**

^1^Simon Fraser University, Faculty of Health Sciences, Burnaby, Canada, ^2^BC Centre for Excellence in HIV/AIDS, Vancouver, Canada, ^3^Canadian Aboriginal AIDS Network ^CAAN^, Vancouver, Canada, ^4^Chronic Viral Illness Service, McGill University Health Centre, Montreal, Canada, ^5^McGill University, Department of Family Medicine, Montreal, Canada, ^6^Women's College Research Institute, Women's College Hospital, Toronto, Canada, ^7^University of Toronto, Department of Medicine, Toronto, Canada

**Background:** Despite HIV treatment advances, there remains considerable inequity in mortality risk among women with HIV in Canada. We measured all‐cause and predictors of mortality among women enrolled in the Canadian HIV Women's Sexual and Reproductive Health Cohort study (CHIWOS).

**Methods:** CHIWOS is Canada's largest community‐based study enrolling women with HIV (trans inclusive; ≥16 years) across British Columbia, Ontario, and Quebec. Participants complete a peer‐administered baseline survey (2013 to 2015), with 18‐month (Wave‐2: 2015 to 2017), and 36‐month (Wave‐3: 2017‐ongoing) follow‐up. Among 1422 women enrolled and followed until 1 December 2017, we determined incidence and cause of death via comprehensive study notification and follow‐up procedures (in British Columbia death was also confirmed via linkage to Vital Statistics). We calculated age‐standardized mortality ratios using 2011 Canadian reference populations. Multivariable proportional sub‐distribution hazards model (with loss‐to‐follow‐up as a competing risk) identified predictors of mortality.

**Results: ** Median age was 42.5 (IQR: 35 to 50); 4.4% identified as trans, and 22.4% Indigenous, 29.4% African/Caribbean/Black, and 41.1% white. Most women were engaged in HIV medical care (97.4%) and on ART (82.6%). 54 women died (crude mortality rate = 11.8 per 1000 woman‐years; 95% CI: 9.0 to 15.3). The age‐standardized mortality rate was 4.54 times higher (95% CI: 3.33 to 5.76) than the general female Canadian population. Primary cause of death was unknown for most women (67%), followed by co‐morbidities including cancer and cardiovascular disease (15%), drug/alcohol use (11%), and HIV‐related opportunistic infections (6%). Baseline factors significantly (*p* < 0.05) associated with mortality included older age, personal annual income <$20,000, illicit drug use, hazardous alcohol use, tobacco use, sex work, incarceration, depression, violence, and poorer physical health‐related‐quality‐of‐life. Independent predictors of age‐adjusted mortality included hazardous alcohol use (aHR 4.62, 95% CI = 1.66 to 12.8), current tobacco use (aHR 3.93, 95% CI = 1.45 to 10.7), and depression (aHR 1.95, 95% CI = 0.97 to 3.92). HIV treatment factors (i.e. ART use, VL, CD4) were not predictive of mortality.

**Conclusions: **We found an alarmingly high mortality rate among a community‐based cohort of women with HIV in Canada, a majority of whom were engaged in HIV care. Preventing premature mortality among women with HIV urgently requires women‐centred HIV community outreach services that address social disparities and mental health needs, and integrate harm reduction services inclusive of tobacco and hazardous alcohol use.

## TUPDC0103

### Tracking trends in HIV/AIDS mortality pre‐and‐post ART: South Africa 1997 to 2012

**V. Pillay‐van Wyk^1^; D. Bradshaw^1^; W. Msemburi^1^; R. Dorrington^2^; R. Laubscher^3^ and P. Groenewald^1^**

^1^South African Medical Research Council, Burden of Disease Research Unit, Cape Town, South Africa, ^2^University of Cape Town, Centre for Actuarial Research, Cape Town, South Africa, ^3^South African Medical Research Council, Biostatistics Unit, Cape Town, South Africa

**Background:** South Africa is one of the many countries most affected by the HIV/AIDS epidemic in the world and has one of the most extensive anti‐retroviral (ART) rollouts in sub Saharan Africa. This programme was rolled out in 2005. This paper reports the trends in HIV/AIDS mortality pre‐and‐post ART rollout in South Africa.

**Methods:** Vital registration cause of death data from Statistics South Africa were adjusted for under‐reporting of deaths using demographic methods. Miss‐attributed HIV/AIDS deaths were identified by regressing excess mortality on a lagged indicator HIV antenatal clinic prevalence. Background trends in the source‐cause (causes to which HIV/AIDS deaths were misclassified) mortality rates were estimated from the trend in cause‐specific mortality experienced among 75 to 84 year olds. Death rates were calculated using mid‐year population estimates and the WHO world standard age‐weights.

**Results: ** In 1997, 60 336 (14.5%) of deaths were attributed to HIV/AIDS; this number peaked in 2006 at 283 564 (68.1%) and decreased to 153 661 (29.1%) by 2012. Between the ages of 25 to 34 years females had higher death rates compare to males. This pattern was reversed in those 45 years and older. The male rates for 65 +  years continued to increase until 2010 with little age difference in mortality levels over the age of 45 years by 2012 (Figure 1).

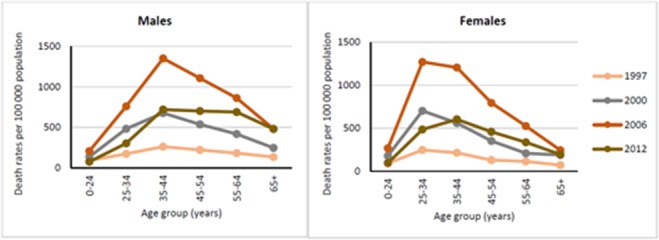

**Abstract TUPDC0103‐Figure 1.**

Female death rates peaked in 2005 while males in 2006. Females and males had similar death rates in the earlier period, however males had higher rates than females. While death rates for Black Africans were similar to the national profile, coloureds, whites and Indians had higher rates among males for the entire study period.

**Conclusions: **HIV/AIDS is the leading cause of death in South Africa even though the number of HIV/AIDS deaths have almost halved since the ART roll out. Our study highlights high male HIV/AIDS mortality rates in the 45+ year olds by 2012. Treatment and prevention programmes should strategize how to target this risk group.

## TUPDC0104

### Mortality trends among HIV infected patients at Newlands Clinic in Harare, Zimbabwe

**T. Shamu^1^; C. Chimbetete^1^; S. Bote^1^; T. Mudzviti^2^ and R. Luethy^2^**

^1^Newlands Clinic, Research, Harare, Zimbabwe, ^2^Newlands Clinic, Harare, Zimbabwe

**Background:** With increasing access to antiretroviral therapy (ART), there is a likely shift in mortality causes among people living with HIV (PLHIV). There are sparse data on mortality patterns among PLHIV in Zimbabwe.

**Methods:** A retrospective cohort study was conducted at Newlands Clinic in which routinely collected data for patients enrolled and followed up between February 2004 and December 2017 were abstracted from the clinic's database. Patient follow up was commenced from the day of the first clinic visit until exit by death, transfer out, loss to follow up or voluntary cessation of ART. A team of doctors grouped causes of death as communicable AIDS related illness (ARI), malignancies, chronic non‐communicable diseases (NCD), non‐AIDS related illness or other. Multinomial logistic regression was used to compare change in mortality cause with year of death as the independent variable.

**Results: ** A cohort of 7845 (62.7% female, n = 4918) patients was followed up for 40,996 person‐years (PY). Median enrolment age was 33 years (IQR 19 to 42). Among 896 patients who died, cause of death was unknown for 130 (14.5%). Male patients had a 28% higher risk of dying than female patients (HR 1.28, CI 1.12 to 1.46, *p* < 0.01). Overall, the most common cause of death was tuberculosis (n = 113, 12.6%), followed by Cryptococcal meningitis (n = 73, 8.1%). The majority of patients died within the first year of enrolment (n = 531, 59.3%), 239 (52.1%) of these of ARI and 82 (17.9%) of NCDs. While ARI remained the most common cause of death throughout the period, NCDs and malignancies increased in relative proportion (Figure 1) (*p* < 0.01, *p* = 0.04 respectively). There was a significant decline in crude mortality rate from 361/100 PY in 2004 to 35/100 PY in 2017 (IRR 0.10, CI 0.05 to 0.20, *p* < 0.01).

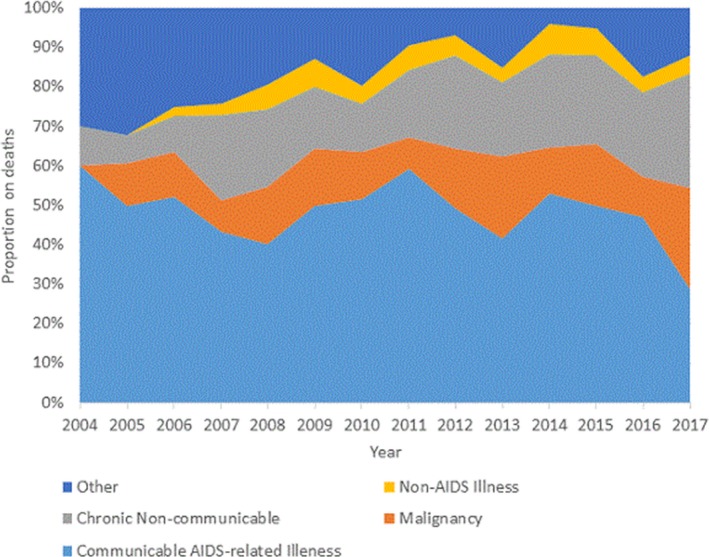

**Abstract TUPDC0104‐Figure 1. Distribution of cause of death over time.**

**Conclusions: **There was a significant decline in mortality rates and change in mortality causes in this cohort. Deaths due to NCDs and malignancies increased over time. ART facilities need to incorporate management of NCDs including cancer in the comprehensive care of PLHIV to reduce mortality.

## TUPDC0105

### Trends in mortality among HIV‐infected subjects: differences by HCV coinfection status

**B. Alejos and Collaboration of Observational HIV Epidemiological Research in Europe (COHERE) in EuroCoord**

Instituto de Salud Carlos III, Centro Nacional de Epidemiología, Madrid, Spain

**Background:** Coinfection by HCV is one of the most common comorbidities in HIV‐infected patients. There are currently limited data on trends in cause‐specific mortality in subjects co‐infected by HCV and HIV compared to subjects only infected by HIV.

**Methods:** We studied trends from 2000 to 2014 in overall and cause‐specific mortality, stratified by HCV status, among HIV‐positive adults within the Collaboration of Observational HIV Epidemiological Research Europe (COHERE). Eligible participants were treatment naïve at start of ART and had at least one anti‐HCV antibody test result at baseline, defined as the date of cohort recruitment for patients with known HCV status at recruitment, or if unknown, the date of first HCV test after recruitment. Follow‐up was divided into calendar periods 2000 to 2007 and 2008 to 2014. Cause‐specific mortality, based on a simplified algorithm adapted from the CoDe coding system, was categorized as: AIDS‐related (AR), Liver‐related (LR), Non‐AIDS malignancies (NADM), Non‐AIDS infections (NADI), cardiovascular, and psychiatric). Adjusted Mortality Rate Ratios (aRR) with 2000 to 2007 as reference were stratified by HCV status using multivariable Poisson regression. We used chained equations multiple imputation of missing data including Cause of Death.

**Results: ** 64,209 patients of whom 2774 died (mortality rate (MR) 8.2/1000 py) were included: 72% males, 48% MSM, 13% HCV‐positive, median age 36 years (IQR 29 to 44), median baseline CD4 383 cells/μL (IQR 207 to 570). The table shows cause‐specific MR per period and the aRR comparing 2008 to 2014 with 2000 to 2007 by HCV status. MR were substantially higher in HCV‐coinfected patients for all causes of death and in both periods. All‐cause, AR and NADI mortality declined from 2000–2007 to 2008–2014 for both mono and co‐infected individuals. Cardiovascular mortality increased almost two‐fold among HCV‐positives whereas it remained practically constant among HCV‐negatives (interaction *p* = 0.022); LR decreased in both populations although the relative decrease was larger among HCV‐negatives (interaction *p* = 0.108).

**Conclusions: **HCV‐coinfection is associated with increased all‐cause and cause‐specific mortality among HIV‐positive patients. Significant relative reductions in all‐cause mortality ‐as well as AR, LR and NADI‐ over time were observed for both mono and coinfected patients. We anticipate that the introduction of new anti‐HCV regimens will significantly impact mortality patterns among co‐infected subjects.

**Abstract TUPDC0105‐Table 1. Mortality Rates (MR) per 1000 py and adjusted effect of calendar period (2008 to 2014 vs. 2000 to 2007) on overall and cause‐specific mortality**

**Table:** 

	HCV NEGATIVE				HCV POSITIVE			
	deaths	2000 to 2007 MR	2008 to 2014 MR	aRR (95% CI)	deaths	2000 to 2007 MR	2008 to 2014 MR	aRR (95% CI)
Overall	1862	7.91	5.67	0.77 (0.70; 0.84)	912	27.89	20.23	0.73 (0.63; 0.83)
AIDS‐related	759	3.93	2.06	0.57 (0.49; 0.66)	265	8.39	5.71	0.71 (0.55; 0.92)
Liver‐related	45	0.25	0.11	0.47 (0.24; 0.90)	159	4.57	3.67	0.86 (0.61; 1.22)
NADM	359	1.12	1.24	1.15 (0.90; 1.47)	80	1.90	2.07	1.07 (0.64; 1.77)
NADI	123	0.62	0.34	0.60 (0.40; 0.89)	76	2.77	1.46	0.55 (0.32; 0.93)
Cardiovascular	155	0.50	0.53	1.12 (0.77; 1.64)	48	0.75	1.45	1.99 (0.97; 4.06)
Psychiatric	128	0.31	0.47	1.41 (0.90; 2.21)	101	3.23	2.15	0.71 (0.46; 1.09)

*Adjusted for age, baseline CD4 count, sex, risk group and cART as time‐updated.

## TUPDC0106

### Mortality and cause of death among HIV patients in London in 2016

**S. Croxford^1^; R. Miller^2^; F. Post^3^; J. Figueroa^4^; I. Harrison^4^; R. Harding^3^; V. Delpech^1^; S. Lucas^5^; S. Dhoot^6^; A. Sullivan^6^ and London Mortality Study Group**

^1^Public Health England, London, United Kingdom, ^2^University College London, London, United Kingdom, ^3^King's College Hospital NHS Foundation Trust, London, United Kingdom, ^4^NHS England, London, United Kingdom, ^5^Guys & St. Thomas NHS Foundation Trust, London, United Kingdom, ^6^Chelsea and Westminster Hospital NHS Foundation Trust, London, United Kingdom

**Background:** Since 2013, the London Mortality Study Group has conducted annual reviews of deaths among people with HIV to reduce avoidable mortality and improve the quality of patient care.

**Methods:** All London trusts commissioned by NHS England to provide HIV care reported 2016 data on patients who died. Data were submitted using a modified Causes of Death in HIV (CoDe) reporting form. Cause of death was categorised by a pathologist and two clinicians.

**Results: ** There were 206 deaths reported across 20 trusts; 77% of these were among men and the median age at death was 56 years. At the time of death, 81% (134/165) of people were on ART, 61% (113/185) had a CD4 < 350 cells/mm^3^ and 24% (47/192) a VL ≥200 copies/mL. Cause was established for 80% (164) of deaths. Non‐AIDS malignancies were the most common cause of death followed by AIDS‐defining illnesses. Where reported (n = 181), risk factors in the year before death included: smoking (37%), excessive alcohol consumption (19%), non‐injecting drug use (IDU) (20%), IDU (7%) and opioid substitution therapy (6%). Co‐morbidities were common (n = 200): 39% had a history of depression, 33% chronic hypertension, 27% dyslipidemia, 18% HBV/HCV co‐infection and 14% diabetes. Almost half of deaths were reported as sudden (44%; 79/177) and 36% (64/178) as unexpected; 60% (63/104) of expected deaths were in hospital. Two thirds of expected deaths (48/72) had a prior end‐of‐life care discussion, though this information was only available for 57%.

**Conclusions: **In 2016, 77% of deaths were due to non‐AIDS conditions and the majority of patients were on ART and virally suppressed. However, a number of preventable deaths were identified. Underlying risk factors, such as smoking and substance misuse were common. Findings also show improvements are necessary in end‐of‐life care planning and in collaborative decision making with patients and other specialties, such as oncology and cardiology.

## TUPDD0101

### Self‐reported violence, perpetrators, and post‐violence care received by key populations in the Integrated MARPs HIV Prevention Program in Cross River State, Nigeria 2016 to 2017

**T. Jaiyebo^1^; R. Abang^2^; A. Yusuf^1^; G. Emmanuel^1^; A. Osilade^2^; O. Olabosinde^1^; C. Trout^3^; B. Ochonye^1^; P. Umoh^1^ and A. Kalaiwo^4^**

^1^Heartland Alliance International, Programs, Abuja, Nigeria, ^2^Heartland Alliance International, Programs, Calabar, Nigeria, ^3^Heartland Alliance International, Programs, Los Angeles, United States, ^4^United States Agency for International Development, Abuja, Nigeria

**Background:** Men who have sex with men (MSM), female sex workers (FSW) and people who inject drugs (PWID), referred to as key populations (KPs), are highly affected by HIV with Cross Rivers state having one of the highest prevalence rates in Nigeria. Violence against KPs can contribute to poor uptake of HIV services. This research explored the occurrence and types of violence experienced, the perpetrators and post‐violence care received.

**Methods:** Between October 2016 and October 2017, the Integrated MARPs HIV Prevention Program (IMHIPP) in Cross Rivers provided comprehensive HIV services including violence services to 20,687 KPs. This was provided in one‐stop‐shops, where KPs report violence and receive post‐violence care from trained counsellors and clinicians. Qualitative methods were used to collate program data, through interviews, questionnaires and focus group discussions to determine the types of violence, perpetrators and post‐violence care required.

Violence was defined as use of force/power that resulted in actual/threatened sexual, physical or emotional harm. Perpetrators were categorized into state actors e.g. police and healthcare workers and non‐state actors e.g. family and sexual partners. Post‐violence care was classified into: mental health, legal services and medical care which included PEP, STI and HIV testing and treatment.

**Results: ** In total, 629 KPs reported violence (FSW: 344, MSM: 118, PWID: 167) with higher numbers of physical (440‐FSW: 236; MSM: 54; PWID: 150) and emotional (333‐ FSW: 102; MSM: 90; PWID: 141) than sexual violence (61‐FSW: 39; MSM: 3; PWID: 19). More non‐state actors than state actors perpetrated violence (395/62.7 : 232/37.8%). This trend was consistent for FSWs (269/78.2% : 75/21.8%) and MSM (64/54.2% : 53/44.9%), but differed for PWIDs (62/37.1 : 104/62.2%).

Most KPs reporting violence received a combination of post‐violence care services: medical‐(223‐ FSW: 52, MSM: 9, PWID: 162), Legal‐ (316‐ FSW: 113, MSM: 69, PWID: 134) and mental health – (546‐ FSW‐336, MSM‐43, PWID‐167).

**Conclusions: **Physical, emotional and sexual violence against KPs are common in Cross Rivers state, Nigeria. Screening, documentation and care for victims of violence, such as legal education/safety and security counselling and security stakeholders’ engagement are essential to promote an enabling environment for KPs to access HIV services.

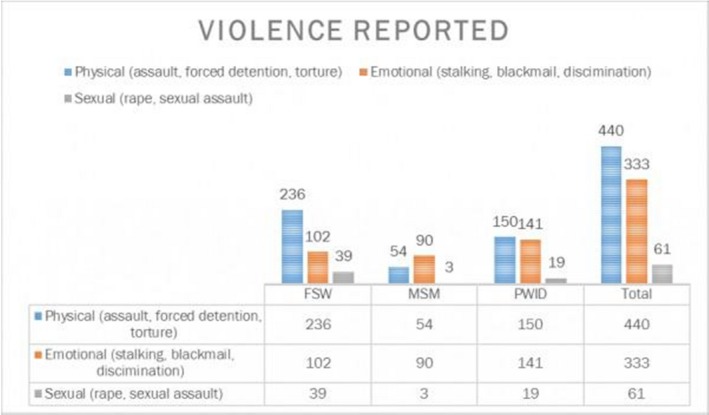

**Abstract TUPDD0101‐Graph 1. Types of violence reported.**

**Abstract TUPDD0101‐Table 1. Table showing the types of perpetrators**

**Table:** 

Perpetrators	Total #	Total %	FSW #	FSW %	MSM #	MSM %	PWID #	PWID %
Perpetrators Non state actors	395	62.7%	269	78.2%	64	54.2%	62	37.1%
State actors	232	37.8%	75	21.8%	53	44.9%	104	62.2%
Both	2	0.3%	0	0%	1	0.8%	1	0.6%
Total #	629		344		118		167	

## TUPDD0102

### Partner violence: A significant part of a syndemic among Black men who have sex with men

**E. Wu; N. El‐Bassel and L. Gilbert**

Columbia University School of Social Work, Social Intervention Group, New York, United States

**Background:** HIV, substance misuse, and partner violence (PV) have long been characterized as a syndemic – interacting and reinforcing epidemics – among key populations such as drug‐involved women and female sex workers. There is a nascent recognition of PV as part of the syndemic among MSM, but there is very limited research specifically focused on the key population of Black men who have sex with men (MSM).

**Methods:** A sample of adult Black MSM (*N* = 1043) completed a screening assessment for a randomized clinical trial of couple‐based HIV intervention for Black MSM in New York City. Respondents provided self‐reported data on experiencing and perpetrating PV (CTS2 supplemented with gay‐ and HIV‐specific forms of PV), and the following outcome variables: self‐reported HIV status, number of male sexual partners, condomless anal intercourse (CAI), and substance misuse. We tested hypotheses that PV would be significantly associated with more adverse levels of each outcome variable.

**Results: ** Table 1 presents prevalence for PV among the sample by type, experiencing versus perpetration, and lifetime versus past 30 days (“current”); 38.3% and 23.2% experienced lifetime and current PV respectively, and 39.2% and 23.5% reported lifetime and currently perpetrating PV respectively. Hypotheses were validated for currently experiencing PV and perpetrating PV as follows: not knowing one's HIV status (AOR = 5.1, 95% CI = 2.1 to 11.7 and AOR = 3.2, 95% CI = 1.4 to 7.3 respectively); greater number of male sexual partners (b = 3.6, 95% CI = 2.6 to 4.6 and b = 3.1, 95% CI = 2.1 to 4.1 respectively); greater number of CAI (b = 6.4, 95% CI = 2.9 to 9.8 and b = 3.7, 95% CI = 0.3 to 7.2 respectively); binge drinking (AOR = 2.1, 95% CI = 1.6 to 2.9 and AOR = 2.1, 95% CI = 1.5 to 2.8 respectively); and illicit substance use (AOR = 2.5, 95% CI = 1.6 to 4.1 and AOR = 3.4, 95% CI = 2.1 to 5.6 respectively). While physical and sexual PV associations were stronger for HIV status, gay‐related PV associations were larger for sexual risk behaviors.

**Conclusions: **The high rates of PV and its multiple associations with HIV risks and substance misuse found in this study underscore the urgent need to address PV as a central factor in the syndemic that underlies Black MSM being and remaining a key, HIV‐affected population. Behavioral aspects of combination prevention need to address PV – paying particular attention to gay‐specific forms of PV – in order to reduce the HIV and health disparities experienced by Black MSM.

**Abstract TUPDD0102‐Table 1. Prevalence of partner violence (PV) among a sample of 1,043 black MSM in New York City**

**Table:** 

Form of PV	Experienced PV		Perpetrated PV	
	Lifetime n (%)	Current n (%)	Lifetime n (%)	Current n (%)
Psychological (severe)	278 (26.7%)	191 (16.4%)	297 (28.5%)	187 (17.9%)
Physical	229 (22.0%)	106 (10.2%)	229 (22.0%)	104 (10.0%)
Sexual	102 (9.8%)	62 (5.9%)	84 (8.1%)	55 (5.3%)
Injurious	140 (13.4%)	50 (4.8%)	134 (12.8%)	52 (5.0%)
Gay‐related	93 (8.9%)	53 (5.1%)	82 (7.9%)	14 (4.5%)
HIV‐related	53 (5.1%)	19 (1.8%)	40 (3.8%)	17 (1.6%)

## TUPDD0103

### Prevalence and predictors of violence against female sex workers in Zambia

**K. Malama^1^; B. Spire^1^; A. Gosset^1^; M. Nishimwe^1^; P.‐J. Coulaud^1^; L. Sagaon‐Teyssler^1^; R. Parker^2^; A. Tichacek^2^; M. Inambao^3^; W. Kilembe^4^ and S. Allen^2^**

^1^Aix‐Marseille University, Marseille, France, ^2^Rwanda Zambia HIV Research Group, Atlanta, United States, ^3^Zambia‐Emory HIV Research Project, Ndola, Zambia, ^4^Zambia‐Emory HIV Research Project, Lusaka, Zambia

**Background:** Violence is a known risk factor for HIV. In Zambia, where the prevalence of HIV is high (13%) and that of gender‐based violence (GBV) even higher (43%), female sex workers (FSW) are particularly susceptible to both outcomes due to their contact with multiple male clients. However, limited data is available on the precise risk factors for violence against Zambian FSW, which would support the implementation of interventions to mitigate against them. We investigated the prevalence and correlates of violence against FSW from their male clients.

**Methods:** The Zambia‐Emory HIV Research Project (ZEHRP) recruited 555 HIV‐negative FSW aged 18 to 45 into a cohort study. FSW were recruited from known hotspots in Ndola and Lusaka between September 2012 and March 2015. At baseline, FSW were asked to provide information on demographics, lifetime sexual history and any previous exposure to violence from their male clients. Forward stepwise logistic regression was used to model the relationship between the outcome (violence from clients, y/n) and potential predictors. The final multivariate model controlled for age, education, age at first sexual encounter, number of years as a sex worker and lifetime number of sexual partners.

**Results: ** From a total of 498 responses, the prevalence of violence against FSW by their male clients was 37% (n = 185). In the fitted model, the highest likelihood of FSW facing violence is noted when sex takes place at the client's home (AOR: 2.47, 95% CI: 1.62 to 3.77, *p* < 0.001). The odds of encountering violence are doubled for FSW whose first ever sexual encounter was physically forced in relation to those who engaged willingly (AOR: 2.09, 95% CI: 1.18 to 3.60, *p* < 0.05). FSW who consume alcohol have an almost two‐fold increase in odds of experiencing violence from clients compared to their counterparts (AOR: 1.94, 95% CI: 1.13 to 3.32, *p* < 0.05).

**Conclusions: **HIV prevention interventions for FSW should broaden their scope to address risk factors such as alcohol consumption, location of sex and history of sexual abuse. This would better inform healthcare workers on how to meet FSW needs. Risk reduction education for both FSW and their clients is required to redress gender norms that heighten the risk of violence and HIV.

## TUPDD0104

### “We′re going to leave you for last because of how you are”: transgender women's experiences of gender‐based violence in healthcare, education, and legal settings in Latin America and the Caribbean

**M. Lanham^1^; K. Ridgeway^1^; R. Dayton^1^; B.M. Castillo^2^; C. Brennan^1^; D.A. Davis^1^; D. Emmanuel^3^; B. Rodriguez^4^; G.J. Morales^1^; C. Parker^1^; J. Cooke^5^; K. Santi^5^ and E. Evens^1^**

^1^FHI 360, Durham, United States, ^2^ASPIDH Arcoíris, San Salvador, El Salvador, ^3^Community Education Empowerment & Development, Bridgetown, Barbados, ^4^Family Planning Association of Trinidad and Tobago, Port of Spain, Trinidad and Tobago, ^5^United Nations Development Programme, Panama City, Panama

**Background:** Transgender women experience gender‐based violence (GBV) from an early age and face barriers to accessing services, resulting in poor health outcomes including the highest HIV prevalence of any key population. To inform HIV policies and services, trans women worked with LINKAGES (led by FHI 360, supported by USAID and PEPFAR), UNDP, and University of the West Indies to document GBV and transphobia in healthcare, education, and legal settings.

**Methods:** Trans women, trained as data collectors, conducted 74 structured interviews in El Salvador, Trinidad and Tobago, Barbados, and Haiti in 2016. We conducted qualitative applied thematic analysis to understand the nature and consequences of GBV and transphobia and descriptive quantitative analysis to identify the proportion who experienced GBV in each setting.

**Results: ** A high proportion experienced GBV in education (85.1%), healthcare (82.9%), police/judicial system (80.0%), and other state institutions (66.1%). Emotional abuse was the most common in all settings; participants also experienced economic, physical, and sexual violence, and other human rights violations. Emotional abuse included gossiping, insults, and refusal to use their chosen name. At school, participants were physically threatened and assaulted, harassed in bathrooms, and denied education. In healthcare, participants were given lower priority and received substandard care. Healthcare workers and police blamed participants’ health and legal problems on them “because of the way I am” and denied them services. From police, participants also experienced physical and sexual assault, theft, extortion for sex or money, and arbitrary arrest and detention. Participants had difficulty obtaining and using identification cards and passports, sometimes being forced to alter their appearance or being denied an identification card. All violence was clearly linked to gender identity and expression.

**Conclusions: **These findings demonstrate a need for policies protecting trans women's rights and interventions addressing GBV against trans women. Given that GBV increases HIV risk, decreases HIV treatment adherence, and prevents trans women from accessing health services, HIV service providers need training to provide nondiscriminatory services and screen for and address GBV among trans clients. Integrated interventions addressing both HIV and GBV could help reduce KPs’ burden of HIV, increase service utilization, and respect, promote and fulfill their human rights.

## TUPDD0105

### Women who use drugs in Estonia: human rights violations as deterrents from HIV treatment

**M. Plotko^1^; A. Kontautaite^1^; D. Matyushina^1^; M. Golichenko^2^; M. Kalvet^3^ and E. Antonova^3^**

^1^Eurasian Harm Reduction Association, Vilnius, Lithuania, ^2^Canadian HIV/AIDS Legal Network, Toronto, Canada, ^3^Estonian Association of People who Use Psychotropic Substances ‘LUNEST’, Johvi, Estonia

**Background:** HIV prevalence in Estonia is one of the highest in Europe (41.9 cases per million), it is primarily spread among people who inject drugs (PWID), and women represent 40% of new HIV cases as of 2013. Despite the WHO treatment guidelines, HIV treatment, especially among PWID, starts on late stages. In August 2017 a qualitative study was organized in Estonia, in order to explore the social factors that impact lives and health of women with HIV and women who use drugs.

**Methods:** Research methodology, developed by EHRA and CHALN, was based on in‐depth interviews carried out by international and local experts. During the field trip to Estonia in August of 2017 38 interviews were conducted, 20 of them were then transcribed and 37 analyzed through thematic content analysis. To ensure personal data protection and participants safety, their names were coded and there is no reference to their real names in the report. All of the respondents were female, aged 26 to 46 years old, mean age 35 years.

**Results: ** The women interviewed reported that they did not want to get tested or start ART because of the stigma associated with HIV and cases of people's HIV status being disclosed at their workplace or at the workplaces of relatives and partners The disclosure of drug dependence and/or HIV status was the main reason for women's unemployment. In 25 cases drug use alone became grounds for the limitation/deprivation of child custody. Low level of protection from violence and the exclusion of drug user women from shelter programs has been discovered. Women also reported regular police abuse, street drug testing and arbitrary arrest. These systematic human rights violations deter women from health services, including HIV and drug treatment, and create mistrust to social protection services and the police.

**Conclusions: **Drug laws and drug enforcement practices, combined with stigma related to drugs and HIV, are the main drivers of systematic and serious violations of the human rights of women who use drugs which undermine Estonia's efforts in HIV prevention, care and treatment.

## TUPDD0106

### Causes and predictors of mortality among people who inject drugs in Tijuana: 2011 to 2017

**B.S. West^1^; D. Abramovitz^1^; P. Gonzalez‐Zuniga^1^; G. Rangel^2^; D. Werb^1^; J. Cepeda^1^; L. Beletsky^1^ and S.A. Strathdee^1^**

^1^University of California San Diego, Division of Infectious Diseases and Global Public Health, La Jolla, United States, ^2^Mexico/US Border Health Commission, Tijuana, Mexico

**Background:** People who inject drugs (PWID) experience multiple risk factors for mortality; yet, we know little about causes and predictors of death among PWID in Tijuana, Mexico, an area with high levels of drug injecting and dynamic changes in policy/law enforcement responses to substance use. This study examines rates, causes, and predictors of mortality among PWID.

**Methods:** Data come from a community‐based cohort of PWID aged ≥18 who injected drugs in the past month. Mortality was confirmed by death certificate over 78 months during 2011 to 2017. Predictors of mortality were identified using Poisson regression with empirical variance estimation, controlling for sex.

**Results: ** Among 863 participants, there were 133 deaths (57 confirmed, 76 unconfirmed), with an incidence rate (IR) of 1.82 deaths per 100 person‐years (95% Confidence Interval (CI) = 1.35, 2.29) for confirmed deaths and 3.94 for unconfirmed deaths (CI = 3.27, 4.60). Confirmed deaths resulted from homicide/trauma (26%), overdose (26%), septic shock (18%), HIV‐related causes (9%), organ failure from chronic substance use (9%), and Hepatitis C (7%). In multivariate analysis of confirmed deaths, being stopped/arrested by police for drug selling/trafficking was a highly significant predictor of mortality (Rate Ratio (RR) = 6.05, CI = 2.22, 16.45); 5.3% of deceased PWID were stopped versus 0.5% of survivors. Baseline HIV seropositivity (7% among deceased vs. 2.7% among survivors) was associated with 4X higher risk of mortality (RR = 4.09, CI = 1.36, 12.29); mortality IR was 2.7X higher among HIV+ PWID. PWID living in Tijuana for longer durations were at increased risk for mortality (RR = 1.03 per year, CI = 1.01, 1.05), while stopping injection for a period of ≥6 months was protective (RR = 0.38, CI = 0.17, 0.85); 14% of PWID who died stopped injecting versus 19.6% among survivors.

**Conclusions: **In addition to overdose and HIV prevention efforts, attention needs to be paid to structural conditions that potentiate mortality risk. Cessation of injection, even for short periods, was associated with lower risk of mortality, indicating a need for improved access to medication‐assisted treatment to reduce injecting. Our results also highlight the potential role for police in reducing PWID mortality and the need to transform police encounters from a source of harm to a source of harm reduction.

## TUPDD0201

### Process as product: implementing participatory, rights‐based research with female sex workers, men who have sex with men, and transgender women

**E. Evens^1^; L. Sinette^2^; K. Santi^3^; J. Cooke^3^; M. Lanham^4^; G. Morales^4^; C. Parker^5^; S. Inniss‐Grant^6^; S. Hunte^7^; M. Mendizabal^8^ and R. Dayton^4^**

^1^FHI 360, Health Service Researech, Chapel Hill, United States, ^2^Friends for Life, Port of Spain, Trinidad and Tobago, ^3^UNDP, HIV, Health and Development, Panama City, Panama, ^4^FHI 360, Research Utilization, Durham, United States, ^5^FHI 360, Behavioral, Epidemiological and Clincal Research, Durham, United States, ^6^Independent Consultant, Bridgetown, Barbados, ^7^University of the West Indies, Institute for Gender and Development Studies, St. Augustine, Trinidad and Tobago, ^8^Asociación Diké de Personas Transgénero y LGBTI+, San Salvador, El Salvador

**Background:** Addressing the HIV‐related needs of key populations (KPs) – men who have sex with men (MSM), female sex workers (FSW), and transgender women – requires new approaches to research. Traditional approaches triggered concerns about human rights violations, data ownership, and unacceptable study questions and methodologies. Meaningful engagement of KPs in research design and implementation addresses these issues and produces high‐quality, applicable findings, but what does meaningful engagement look like and how is it fostered?

**Methods:** The USAID and PEPFAR‐supported LINKAGES project, together with UNDP and The University of the West Indies, collaborated with global, national, and local KP‐led organizations to conduct participatory, rights‐based research on KPs’ experiences of violence and its connections with HIV risk in four Latin American and Caribbean countries. KP organizations provided input on study populations, interview topics, recruitment, participant safety, study instruments, analysis, and use of results. All data collectors were KP members. The study used principles of rights‐based research to promote the human rights of stigmatized communities, including respect, gender equity, strengthening alliances between organizations and government, and ensuring the well‐being of participants.

**Results: ** We identified strategies to design research that meets the needs of study communities and yields results likely to be used in policies and programs: (1) collaboration with KPs to define and reach study populations, with MSM providing input on sample sizes to ensure they reflected MSM's educational and occupational diversity and all peers having access to populations difficult to recruit in stigmatized environments; (2) supporting capacity‐building, including training in research ethics and interviewing to ensure a strong voice in the process and allow KP organizations to advocate using the findings; and (3) inclusive decision‐making through advisory groups to provide feedback on study design/implementation, with each KP group determining the types of violence most important to their community – FSWs focused on workplace violence – resulting in more appropriate results being used to advocate for each groups’ priorities.

**Conclusions: **Meaningful engagement of KPs is an essential requirement of ethical and participatory research and produces better study outcomes more likely to inform policy and practice. Our experiences highlight specific effective methods of meaningful engagement.

## TUPDD0202

### Adolescent and young people's participation and representation in clinical trials: lessons from a community‐wide HIV testing and treatment study, the HPTN 071 (PopART) study

**M. Simwinga^1^; J. Mwate^1^; T. Ng'ombe^1^; S. Belemu^1^; N. Makola^2^; C. Mubekapi‐Musadaidzwa^2^; G. Hoddinott^2^; R. White^3^; K. Shanaube^1^; V. Bond^1,4^ and B. Virginia^1^**

^1^Zambart, Lusaka, Zambia, ^2^Desmond Tutu TB Centre, Cape Town, South Africa, ^3^FHI 360, North Carolina, United States, ^4^LSHTM, London, United Kingdom

**Background:** Community Engagement (CE) is imperative to research for both instrumental and ethical reasons. However, adolescents’ and young people's (AYP) participation in research conducted in Africa is infrequent. It is poorly understood how to meaningfully involve AYP in research, promoting critical dialogue between researchers and AYP. HPTN 071 (PopART) is a community based trial in 21 study communities in Zambia and South Africa. The trial includes a nested ancillary study (2016 to 2017) to evaluate AYP's (15 to 24 years) uptake of HIV‐related services.

**Description:** Formative research was conducted to identify AYP‐specific community stakeholders and AYP possible interventions in consultation with AYP representatives and the existing adult Community Advisory Boards (CABs). Consultations resulted in the creation of 12 AYP‐only CABs (aCABs) in Zambia and one AYP‐only CAB in South Africa. These CABs met monthly. We report on data collected through group discussions (n = 8) and in‐depth interviews (n = 63) conducted among aCAB members in Zambia in 2017 exploring their perceptions of the aCAB's role in the study. We also reflect on our experiences of establishing and maintaining AYP participation and representation processes from the onset to preliminary results dissemination.

**Lessons learned:** AYPs were enthusiastic to serve as representatives. However, their participation was constrained by high mobility and the requirement for parental/guardian permission to attend meetings. They requested for formation of AYP‐only CABs and not just the inclusion of AYP representatives in adult CABs. Some AYP intervention strategies were both suggested and implemented by them. AYP CABs sought to protect the privacy and confidentiality of AYP research participants and respect their autonomy. For example, they recommended waiver of parental consent for 15 to 17‐year‐olds in a survey and rejected the offer of incentives to research participants. AYP leadership, involvement in decision making and determination of specific study activities represented a significant achievement especially in the context of research in Africa where AYP voices are rarely considered.

**Conclusions/Next steps:** AYP in Africa are capable of participating meaningfully in research that directly impacts their lives. While challenges to participation in research exist, researchers should be encouraged to invest in meaningful partnerships with AYPs. CABs with only AYP representatives are one such strategy.

## TUPDD0203

### Diverging perspectives on the role of a community advisory board at a biomedical HIV prevention research centre in South Africa

**A.M. Lesch; A. De Wet; L. Swartz; A. Kagee; Z. Kafaar and N. Hassan**

Stellenbosch University, Psychology, Stellenbosch, South Africa

**Background:** Community engagement is prescribed as crucial to the successful conduct of biomedical HIV prevention trials. The inclusion of a community advisory board (CAB) in research is one of the most common ways of engaging communities in clinical research. CABs constitute a formal stakeholder advisory mechanism: advising researchers, representing the local community and ensuring that the research is sensitive to the local community context. While researchers and CABs have a common goal, power imbalances and a range of other challenges may hamper the relationship between these stakeholder groups. In our research to evaluate the community engagement programme at a biomedical HIV prevention research centre in the Western Cape, South Africa, we documented the perspectives and experiences of multiple stakeholders on the role of the CAB and the relationship between the CAB and research centre staff in the study setting.

**Methods:** We used purposive sampling to generate a sample of 20 participants from multiple stakeholder groups involved in the research in the study setting. We collected our data using semi‐structured interviews. Interviews were audio recorded with the permission of participants and transcribed. We analysed our data using the thematic analysis approach outlined by Braun and Clarke (2006).

**Results: ** The relationship between the CAB and research centre staff was reportedly characterized by feelings of frustration, mistrust and tensions due to the power imbalance between the CAB and the researchers. Research centre staff and CAB members held diverging perspectives on the role of the CAB in the research. While research centre staff viewed the role of the CAB as representing the local community and advising the research team on the operational and ethical aspects of the research, CAB members saw themselves as intermediaries, linking the community to the research centre and facilitating dialogue and engagement between these two entities.

**Conclusions: **The diverging perspectives held by research centre staff and the CAB hampers the development of trust, mutually beneficial partnerships and ongoing dialogue between the researchers, the CAB and the local community. Building mutually beneficial partnerships and establishing mechanisms for transparent, ongoing dialogue between researchers and the local community is crucial to meaningful community engagement.

## TUPDD0204

### Stakeholder engagement for HIV clinical trials: a systematic review of the evidence

**S. Day^1^; M. Blumberg^1^; T. Vu^1^; Y. Zhao^2^; S. Rennie^3^ and J. Tucker^2,4^**

^1^University of North Carolina, Institute for Global Health and Infectious Diseases, Chapel Hill, United States, ^2^SESH Global, UNC Project‐China, Guangzhou, China, ^3^University of North Carolina, Social Medicine, Chapel Hill, United States, ^4^University of North Carolina, School of Medicine, Chapel Hill, United States

**Background:** Stakeholder engagement is an essential component of HIV clinical trials. We define stakeholder engagement as input on the design or conduct of HIV clinical trials by individuals or groups with an interest in these trials. Despite its value, stakeholder engagement is often poorly defined and not rigorously evaluated. The objective of our systematic review is to examine the characteristics of stakeholder engagement for HIV clinical trials.

**Methods:** Four databases (PubMed, Ovid, CINAHL, Web of Science) were searched for English language studies describing stakeholder engagement for HIV clinical trials, with additional studies identified using other methods (e.g. handsearching). Two reviewers independently assessed studies for inclusion, resolving discrepancies via third reviewer. Data were extracted on the location of engagement, engagement methods, stakeholder types, and purpose of stakeholder engagement. Based on UNAIDS Good Participatory Practice (GPP) guidelines, we examined how frequently stakeholder engagement was conducted to inform the following stages: research question development, protocol development, recruitment, enrolment, follow‐up, trial results, and dissemination.

**Results: ** Of 452 citations identified, 108 studies were included in the analysis (93 via database search, 15 via other methods). Forty‐eight studies (44.4%) described stakeholder engagement in high‐income countries, 30 (27.8%) in middle‐income countries, and 9 (8.3%) in low‐income countries (mixed location/indiscernible: 21 studies; 19.4%). Fifteen distinct methods for stakeholder engagement were identified, including individual (e.g. interviews) and group (e.g. community advisory boards) strategies. Thirty‐five types of stakeholders were engaged, with approximately half of studies (59; 54.6%) engaging HIV‐affected community stakeholders (e.g. people living with HIV, at‐risk for HIV, or related populations of interest). We observed greater frequency of stakeholder engagement to inform recruitment (48 studies; 44.4%) and protocol development (46 studies; 42.6%) (Figure 1). Fewer studies described stakeholder engagement to inform post‐trial processes related to trial results (3; 2.8%) and dissemination (11; 10.2%).

**Conclusions: **We found that stakeholder engagement was more robust in the early stages of clinical trials, with less engagement to inform the later stages. This suggests the current level of stakeholder engagement in later stage HIV clinical trials may be insufficient according to UNAIDS GPP guidelines. Further stakeholder engagement across all clinical trial stages is needed.

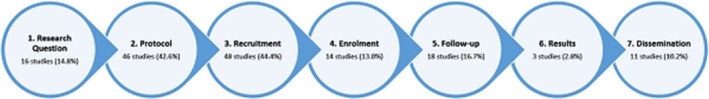

**Abstract TUPDD0204‐Figure 1. Number of studies conducting stakeholder engagement to inform each of the 7 stages of HIV clinical trial research.**

## TUPDD0205

### Toward standardized metrics for the conduct of community engagement in HIV biomedical prevention research studies

**G. Broder^1^; S. Wallace^1^; N. Luthuli^2^ and K. Baepanye^2^**

^1^Fred Hutchinson Cancer Research Center, HIV Vaccine Trials Network, Seattle, United States, ^2^Fred Hutchinson Cancer Research Center, HIV Vaccine Trials Network, Johannesburg, South Africa

**Background:** While Good Participatory Practice standards are in place and there is support for community engagement in clinical HIV prevention research, to date there are no standardized metrics that define its practice and evaluation. The Community Engagement Unit (CEU) of the HIV Vaccine Trials Network sought to create a baseline by which recruitment practices for the two monoclonal antibody AMP Studies could be described, monitored, and assessed.

**Description:** CEU staff developed recruitment strategy descriptors for each study. These were vetted with the studies’ Community Working Groups and edited. Common definitions and time points were established to allow comparisons across sites. Potential participants presenting at the sites were asked, “How did you hear about the AMP Study?” Sites submit their data to the CEU quarterly. CEU staff tabulate it, monitor trends, determine areas where sites need support, and where there are successes to be shared and replicated. Data are presented for 1 January 2017 to 30 November 2017.

**Lessons learned:** All 29 global sites utilize multiple recruitment strategies successfully, but the strategies used vary by region. The most effective recruitment strategy across global sites was referrals (2.4:1 screening to enrollment ratio). The least effective was Print Materials/Ads in Peru/Switzerland/US (5.8:1, 17 sites) and the internet in Africa (6:1, 12 sites). While sites generated a similar number of screening appointments using face to face outreach (719 in Peru/Switzerland/US, 717 in Africa), they represent strikingly different percentages of the total number of persons screened during this period (26% in Peru/Switzerland/US, 50% in Africa), and strikingly different percentages of total enrollment (29% in Peru/Switzerland/US, 56% in Africa). The internet represents the greatest difference between regions, 43% of total enrollments in Peru/Switzerland/US and 0.8% in Africa.

**Conclusions/Next steps:** Standardized metrics enable meaningful comparisons, and support development of evaluation metrics. It is imperative that all global trial sites are supported with resources enabling use of a range of recruitment strategies, and each site must target the strategies to their local environment. Outreach staff must maximize the use of all available resources and outreach strategies for successful research recruitment, and utilize data to target their efforts to improve their screening to enrollment ratio.

**Abstract TUPDD0205 Table 1. Summary of HVTN Screening to Enrollment Recruitment Data for the AMP Studies**

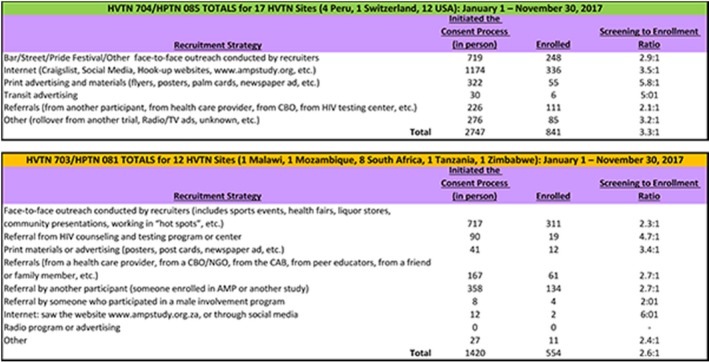

## TUPDD0206

### GIPA in action: PLHIV leadership and guidance in the development of a new PLHIV quality of life scale for the community and policy sector

**G. Brown^1^; A. Cogle^2^; C. Cooper^3^; S. O'Connor^4^; B. Allan^5,6^; S. Malhotra^7^; G. Mikolajczak^8^; J. Power^8^; A. Lyons^8^ and F. Drummond^9^**

^1^La Trobe University, Australian Research Centre in Sex, Health and Society, Melbourne, Australia, ^2^National Association of People Living with HIV, Sydney, Australia, ^3^Positive Life, Sydney, Australia, ^4^Queensland Positive People, Brisbane, Australia, ^5^Australian Society for HIV, Viral Hepatitis and Sexual Health Medicine, Sydney, Australia, ^6^International Council of AIDS Service Organizations, Toronto, Canada, ^7^Living Positive Victoria, Melbourne, Australia, ^8^Australian Research Centre in Sex, Health and Society; La Trobe University, Melbourne, Australia, ^9^ViiV Healthcare Australia, Melbourne, Australia

**Background:** The greater involvement of people with HIV/AIDS (GIPA) has been a foundation of effective responses to HIV. However, in research application of GIPA principles is often limited to consultation rather than full collaboration. The development of the PozQoL quality of life (QoL) scale demonstrates the benefits of full collaboration to achieve high quality research outcomes.

HIV organisations in Australia have prioritised improving the QoL of people living with HIV (PLHIV) in their programs as a strategy to reduce the negative personal impact of HIV and improve treatment uptake and maintenance goals. In order to measure the impact of these efforts, a partnership of PLHIV peer organisations, researchers, and industry was established to pool resources and develop a QoL scale for PLHIV. The scale needed to be short, empirically validated and easy to incorporate into the day‐to‐day practice of health and community services. This partnership was committed to GIPA being at the centre of the process.

**Description:** The PozQoL partnership involved active direction from PLHIV peer leadership in the:

(1) Conceptualisation of the research approach,

(2) Prioritisation of the QoL domains to be included in the scale,

(3) Development and review of scale items,

(4) Engagement and mobilisation of the PLHIV community to support the validation survey, and

(5) Analysis and decisions concerning the refinement of the final scale.

**Lessons learned:** Benefits of the high involvement of PLHIV peer leadership includes:

(1) A deeper understanding of the complex experiences of PLHIV related to QoL,

(2) Ensuring a balance of statistical rigour, conceptual accuracy, and practical use of the PozQoL scale,

(3) Strengthened relationship across research, community and industry,

(4) Opportunity to demonstrate credibility and authenticity within PLHIV community.

**Conclusions/Next steps:** Taking participation with PLHIV peer‐led organisations beyond consultation to active partnership enabled the study to ensure the research rigour was complemented by practical and conceptual considerations, and high community engagement. This contributed to achieving not only statistical validity but also high ecological validity for the real‐world experience of PLHIV. This process has also built scale's credibility across the sector: PozQoL scale is currently being field‐tested by 15 community, support and healthcare programs for PLHIV in Australia

## TUPDE0101

### Where are the HIV positives in Kenya? Unmasking testing yield in a spatial context

**A. Waruru^1,2^; J. Wamicwe^3^; J. Mwangi^2^; L. Ng'ang'a^2^; F. Miruka^2^; T.N.O. Achia^2^; P. Yegon^2^; D. Kimanga^2^; J. L. Tobias^4^; K.M. De Cock^2^ and T. Tylleskär^1^**

^1^University of Bergen, Centre for International Health, Bergen, Norway, ^2^Centers for Disease Control and Prevention, Division of Global HIV/AIDS and TB (DGHT), Nairobi, Kenya, ^3^National AIDS and STI Control Program (NASCOP), Ministry of Health, Nairobi, Kenya, ^4^U.S Centers for Disease Control and Prevention (CDC), Division of Global HIV &TB, Atlanta, United States

**Background:** The UNAIDS 90‐90‐90 targets provide a framework for assessing coverage of HIV testing services (HTS), linkage to care, and viral load suppression. In Kenya, the bulk of HTS targeting is in 5 high HIV‐burden counties: Nairobi, Homabay, Kisumu, Siaya, and Migori, accounting for 45.1% of the estimated people living with HIV PLHIV. Geographic analysis, can help to focus and prioritize to increase diagnosis of PLHIV to reach the “first 90”.

**Methods:** We analyzed routine site‐level HTS data in Kenya to assess spatial distribution of HIV‐positives (yield) within counties (sub‐national units (SNUs)). We used the global Moran′s Index (Moran's I) in ArcGIS™ ver. 10.51 to assess spatial autocorrelation of yield by site. Inverse Euclidean distances were used to conceptualize spatial relationships; the further the sites were from each other, the lesser the impact of spatial relationship(s). We classified sites as having no‐clustering (random distribution), or auto‐correlated neighbors as hotspots with hotspots (HH), hotspots with low‐spots (HL), low‐spots with hotspots (LH), and low‐spots with low‐spots (LL).

**Results: ** In 2016, out of 4021 HTS sites, 3,969 (98.7%) had geo‐coded data. Global Moran's I was 0.023, expected I was −0.00025, Z‐score 33.9 and *p* < 0.001. Most sites showed no‐clustering (3034, 76.4%); others were grouped as: HH (438, 11.0%), HL (66, 1.7%), LH (275, 6.9%), and LL (156, 3.9%). Of the HH sites, 301 (68.7%) were in high HIV‐burden SNUs distributed as follows: Homabay with 78/184 (42.4%), Kisumu 57/137 (41.6%), Siaya 50/145 (34.5%), Migori 43/139 (30.9%), and Nairobi 73/239 (30.5%). HH sites in high burden counties were near water bodies (Homabay, Kisumu, Siaya and Migori) or a large city (Nairobi) and in low HIV‐burden areas near major roads (Figure).

**Conclusions: **We identified wide‐ranging spatial variation of yield clusters. High HIV‐burden SNUs contain most high yielding sites but some are also in low‐burden SNUs. Access to HTS is needed everywhere in Kenya, yet, targeting is difficult in low prevalence areas. Geospatial analyses help to define hotspots and priority areas for enhanced HTS to spatially refine targeting and achieve the “first 90.”

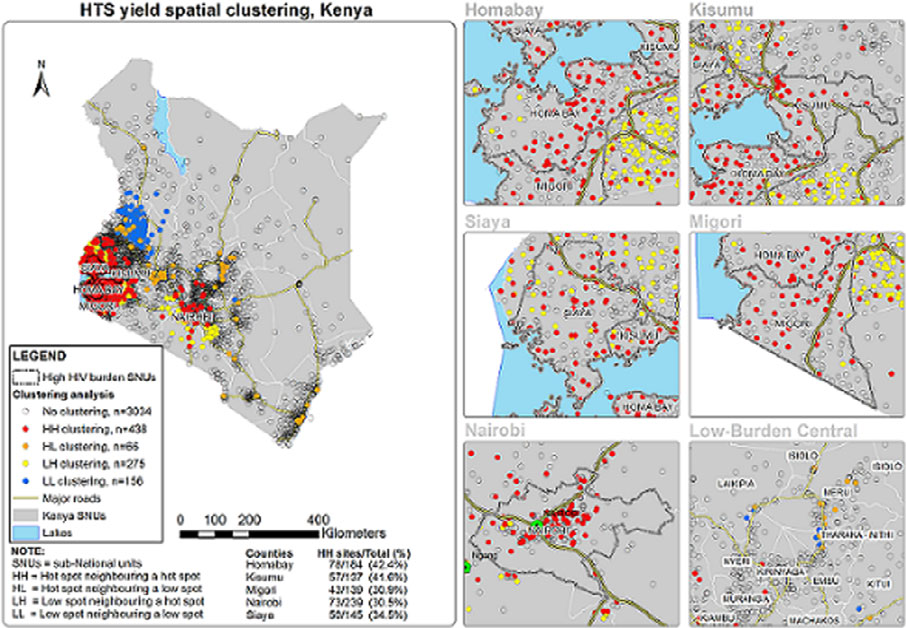

**Abstract TUPDE0101 Figure 1. Spatial distribution of HTS yield in Kenya and in 5 high HIV‐burden counties and 1 low burden region, 2016.**

## TUPDE0102

### Patterns of HIV in the Lake Victoria region, a spatiotemporal analysis

**N. Heard^1,2^; G. Sarfaty^3^; I. Zaidi^4^ and C. Fellenz^5^**

^1^U.S. Department of State, Office of the U.S. Global AIDS Coordinator and Health Diplomacy, Washington, DC, United States, ^2^U.S. Department of State, Humanitarian Information Unit, Washington, DC, United States, ^3^U.S. Agency for International Development, Office of HIV/AIDS, Washington, United States, ^4^U.S. Department of State, Office of the U.S. Global AIDS Coordinator and Health Diplomacy, Washington, United States, ^5^U.S. Department of State, Humanitarian Information Unit, Washington, United States

**Background:** Fisherfolk are a relatively mobile population subject to a variety of socioeconomic and behavioral risk factors that drive HIV infection in the Lake Victoria Basin. Although country‐specific studies have established that populations residing near the lake experience higher HIV prevalence than the general populations of their respective countries, less is known about spatial patterns of HIV in the region as a whole. This study explores routine program data to detect spatial clustering of HIV positivity across Kenya, Tanzania, and Uganda. In addition, the study seeks to characterize the temporal stability of identified clusters of HIV positivity in Lake Victoria Lake Basin.

**Methods:** We evaluated seven reporting quarters of HIV testing data for significant spatial structure. The quarterly data are from 2256 PEPFAR‐supported health facilities within 50 kilometers of the banks of Lake Victoria. We stratified by quarter facility‐level data for 15,841,128 tests, of which 384,473 were positive, and aggregated mean HIV positivity to a mesh of tessellated hexagons. The analysis tested for spatial randomness using the Getis‐Ord Gi* statistic.

**Results: ** We detected four stable clusters of high HIV testing yield, present in each of the seven quarters, and two unstable clusters, appearing in at least three of the seven quarters. In addition, results indicated one potentially emerging cluster of high yield. A stable cold spot crosses the border of Kenya and Uganda.

**Conclusions: **Despite labor‐related population mobility in the region, HIV testing results show persistent patterns in both space and time. High population mobility complicates linkage to care. However, consistently high testing yield in fixed places suggests that a geographically focused programmatic approach remains a valid and important part of this local response. Future studies should leverage routine program data and consider multiple geographic scales to assess both cross‐border and internal disease dynamics.

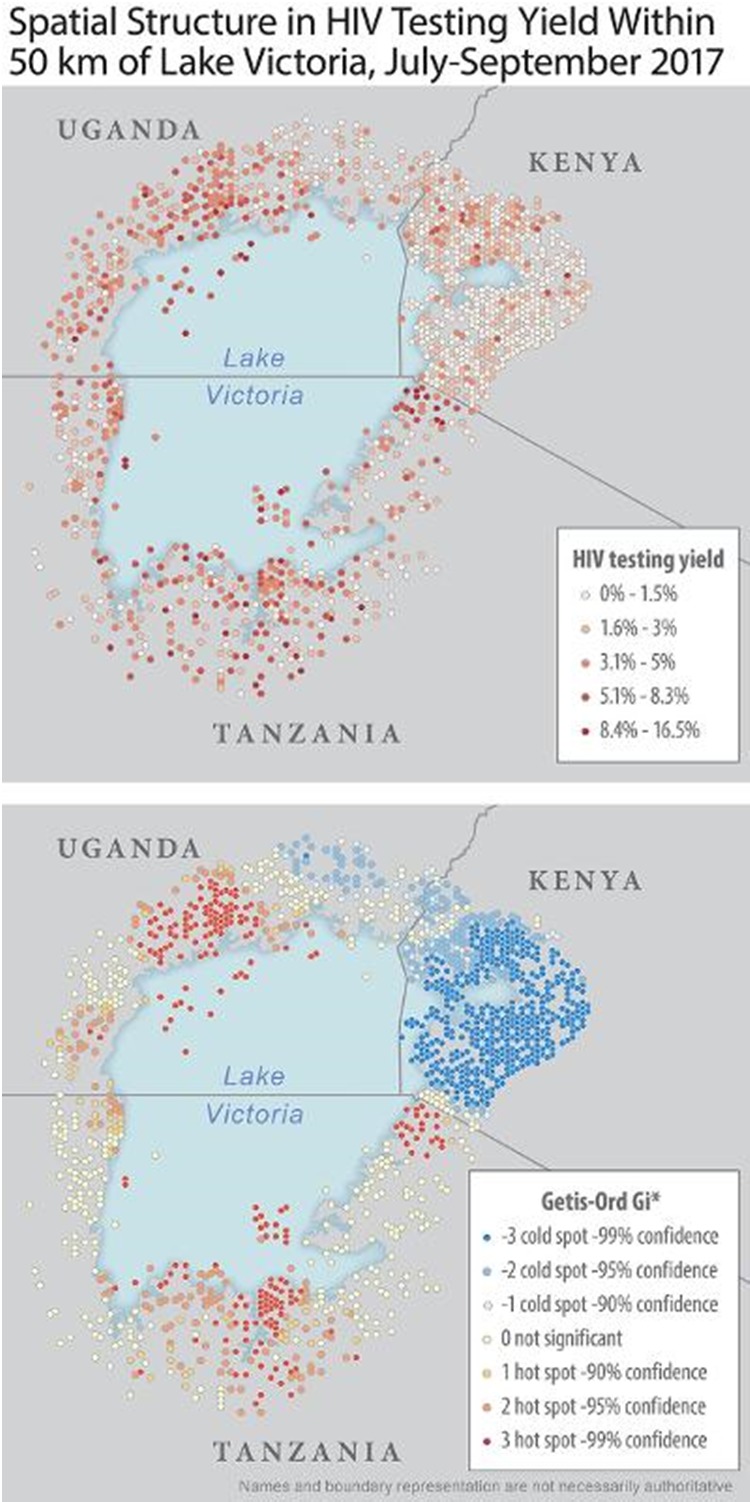

**Abstract TUPDE0102‐Figure 1. Spatial Structure in HIV Testing Yield Within 50 km of Lake Victoria, July to September 2017**

## TUPDE0103

### Density mapping of dating app users across time and space in Mumbai, India

**B. Eveslage^1^; P. Shah^2^; C. Parker^1^; B. George^3^ and J.J. Baishya^4^**

^1^FHI 360, Washington, United States, ^2^FHI 360, LINKAGES, New Delhi, India, ^3^FHI 360, Country Director, New Delhi, India, ^4^USAID, New Delhi, India

**Background:** Dating apps make it easier and seemingly safer for men who have sex with men (MSM) to find partners. How, then, can HIV programs use available data to better align HIV outreach activities to the specific times and locations of MSM dating app usage while maintaining safety and privacy?

**Methods:** The USAID and PEPFAR‐supported LINKAGES project in India created density maps of location‐based dating app users in Mumbai to identify concentrations of users and enhance program coverage. Using ArcGIS software, Mumbai was overlaid with a gridwork of latitude/longitude points each two kilometers apart (374 points), and a second group of points at 1 km distance (410 total). Data collectors used Android phones and an app to set their phone's Global Positioning System (GPS) location for each of the plotted coordinates, then opened Grindr to count the number of nearby online users. The number of online users within one kilometer of each 2‐km separated point was collected three times daily, over five days of one week in October 2017. The process was repeated in December 2017 for online users within 500 meters of each 1‐km separated point over seven days. Excel's 3D maps plug‐in generated short videos animating the changing density of Grindr users across the week and area of Mumbai. These activities never collected individual location data, and data are not granular enough to target people in physical settings.

**Results: ** An average of 3,810 online Grindr users were counted across metropolitan Mumbai. The highest number of online users was counted on midday Saturday and Sunday. On weekdays, counts increased later in the day. The second mapping revealed high‐density locations of dating app use including several time‐bound areas and six consistent areas with 23 or more online users within 500 meters of each coordinate (Figure 1).

**Conclusions: **This activity successfully identified physical clusters of online MSM at specific times of dating app usage, and estimated total online users in Mumbai. The LINKAGES program in India used these results to guide outreach to MSM on Grindr at high density locations through peer‐ and advertisement‐based approaches that will be combined with location‐specific referrals for services.

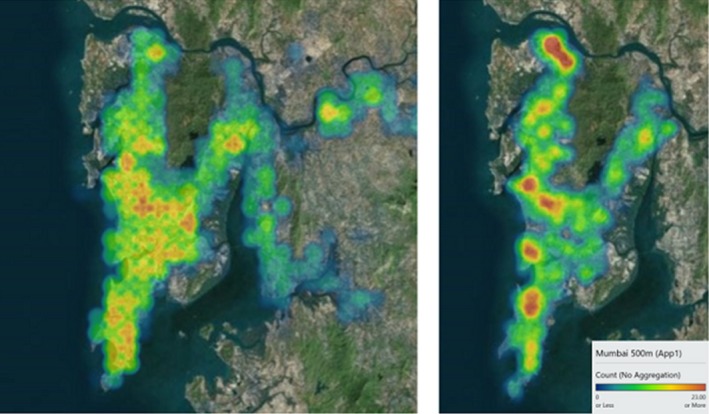

**Abstract TUPDE0103‐Figure 1. Density mapping in Mumbai compared – 1 km radius sampling (left) versus more precise 500 m radius sampling (right) showing clear high‐density.**

## TUPDE0104

### Optimizing access for the last mile: geospatial cost model for point of care viral load instrument placement in Zambia

**S.J. Girdwood^1^; B.E. Nichols^1,2^; C. Moyo^3^; T. Crompton^4^; D. Chimhamhiwa^4^; S. Rosen^1,2^ and on behalf of EQUIP**

^1^Health Economics and Epidemiology Research Office, Department of Internal Medicine, School of Clinical Medicine, Faculty of Health Sciences, University of the Witwatersrand, Johannesburg, South Africa, ^2^Boston University, Global Health, Boston, United States, ^3^EQUIP Zambia, Lusaka, Zambia, ^4^Right to Care, GIS Mapping Department, Johannesburg, South Africa

**Background:** Viral load monitoring programs have been scaled up rapidly throughout Africa, but little attention has been paid to how to optimize viral load access to the most remote healthcare facilities (the “last mile”). For the hardest‐to‐reach facilities in Zambia, we compared the cost of placing point of care (POC) viral load test instruments at or near facilities to the cost of an expanded sample transportation network (STN) to deliver samples to centralized laboratories.

**Methods:** Using data from the Zambian viral load program, we developed a geospatial optimization model that first optimized a STN for centralized labs for 90% of estimated viral load volumes, at a cost per test of $23.86. Exploratory cluster analysis was then conducted to identify ideal facilities for POC instrument placement amongst the remaining 10% of volumes. Optimal placement maximized both viral load coverage and instrument utilization. We evaluated the full cost (test and transport) per test under three scenarios:

(1) POC placement at all facilities identified for POC;

(2) an optimized combination of true on‐site POC placement and “near POC” placement at facilities acting as POC hubs; and

(3) integration into the centralized STN to allow use of centralized labs.

**Results: ** Exploratory cluster analysis yielded optimal POC placement that covered 3.5% of total viral load volumes. Scenario 2 resulted in a cost per test of $37,97, 21% less than the cost per test of true‐POC available at all of the same facilities (Scenario 1, $47.95). This is due to increased POC instrument utilization when an instrument can act as near‐POC (median 23% instrument utilization), compared to only true‐POC (11%). Scenario 3 was the most costly at $53.2 per test, due to high transport costs under the centralized model ($31.40 per test compared to $4.80 per test in scenario 2).

**Abstract TUPDE0104‐Table 1. Cost per viral load test by scenario for hardest‐to‐reach patients**

**Table:** 

	Scenario 1 (POC at all facilities)	Scenario 2 (optimized combination of POC and near‐POC)	Scenario 3 (expanded sample transport network)
Median total cost per test (IQR)	$47.95 (46.83 to 50.54)	$37.97 (36.6 to 40.28)	$53.16 (48.59 to 58.59)
Test (IQR)	$47.95 (46.83 to 50.54)	$33.13 (31.99 to 35.00)	$21.76 (20.33 to 23.58)
Sample transport (IQR)	$0	$4.80 (4.61 to 5.28)	$31.40 (28.26 to 35.01)
Projected annual number of tests conducted (% of total viral load volume)	59,892 (3.5%)		
Total cost of scenario (IQR)	$2.87 million ($2.80 million to $3.03 million)	$2.27 million ($2.19 million to $2.41 million)	$3.19 million ($2.91 million to $3.51 million)
Savings from using optimized POC and near‐POC	$597,465	‐	$912,419

**Conclusions: **Point of care viral load testing may reduce the costs of providing testing services to the hardest‐to‐reach populations, despite the cost of equipment and low patient volumes. An optimal combination of true‐ and near‐POC instruments can reduce the cost per test by 21% to 29% by reducing transport costs and increasing instrument utilization under the POC scenario.

## TUPDE0105

### Transgender resource map

**M. Domingo^1,2^**

^1^Callen‐Lorde Community Health Center, Population Health, New York, United States, ^2^Carto, Brooklyn, United States

**Background:** Transgender people face daily harassment and discrimination, which impacts their ability to access and receive services like healthcare. In addition to this, Transgender people face additional challenges when receiving services due to lack of competence and prejudice from service providers, especially medical care. It is no surprise this group faces the largest burden in new HIV infections in the United States. Very little has been done to ensure Transgender people are connected to quality and competent social and health services. Callen‐Lorde Community Health Center, a leader in Transgender care in the United States, describes its approach to addressing this inequities using an innovative technology approach.

**Description:** Utilizing GIS (geographic information systems) software, Callen‐Lorde's Population Health team mapped the geographic location and distribution of its Transgender patients in New York City's 5 boroughs by census tract. Simultaneously, all transgender specific resources and linkages kept by the clinic for referrals where mapped by address (Figure 1). The map allows individuals to search for specific services available in a given location. Services included things like Behavioral health resources, transgender surgery information, legal assistance and others.

**Lessons learned:** Transgender specific resources were available and abundant in New York, however they were mainly concentrated in the downtown central area of Manhattan, while most patients inhabited the farther regions of the city. Interactive maps and GIS technology allows our supportive staff to link patients to services and come up with outreach strategies to avoid discrimination and harassment. These maps provide an opportunity to invite business into high‐needs areas, helping to bridge inequities. Through this program, we are able to understand how to allocate resources in a strategic and targeted approach.

**Conclusions/Next steps:** GIS technology can be successfully utilized to bridge determinants of health in transgender communities. Innovative approaches are needed to help ensure transgender communities have the tools to connect to service that will support and treat them with dignity.

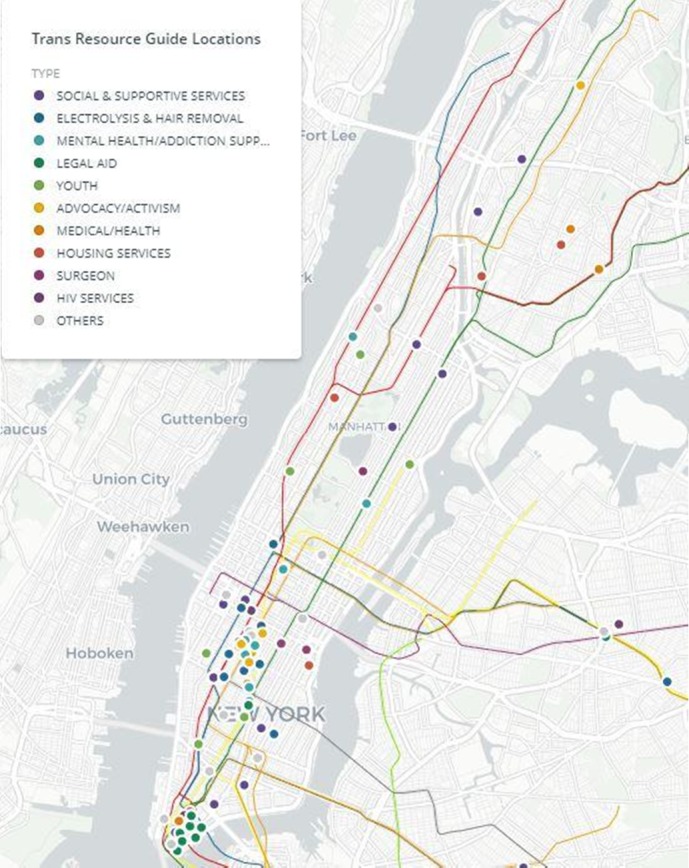

**Abstract TUPDE0105‐Figure 1.**

## TUPDE0106

### Use of geographic information system mapping for scaling‐up voluntary medical male circumcision services in Tanzania

**K. Nyalali^1^; P. Sekule^1^; P. Mwakipesile^1^; M. Swai^1^; J. Brasileiro^2^; C. Brokenshire‐Scott^2^; L. Mphuru^1^; K. Kazaura^3^ and D. Simbeye^3^**

^1^IntraHealth International, Dar es Salaam, Tanzania, United Republic of, ^2^IntraHealth International, Chapel Hill, United States, ^3^Centers for Disease Control and Prevention, Dar es Salaam, Tanzania, United Republic of

**Background:** Scaling‐up voluntary medical male circumcision (VMMC) becomes trickier as coverage increases and the number of eligible men decreases. IntraHealth International, with CDC funding, used geographic information system (GIS) mapping with information from community experts to identify geographical areas in Tanzania with large numbers of uncircumcised men for targeted service delivery.

**Description:** We collected 2016 ward‐level male population projection data in the four IntraHealth‐supported regions and shape files with geo‐referenced points (e.g. ward boundaries, road networks, forests/vegetation, water bodies) from the National Bureau of Statistics. The geo‐coordinates of health facilities were extracted from the national health facility registry and DATIM GIS interface and used to geo‐reference facility locations. We subtracted 2011 to 2016 service delivery data on circumcised men from the PROMIS database to establish estimations of uncircumcised men in the respective wards of the four regions. Geo‐coded data and shape files were overlaid into GIS software (ArcGIS Pro Esri 2017) and analyzed to create maps of facility locations, coverage and areas with high concentration of uncircumcised men where demand creation and VMMC scale‐up were prioritized.

**Lessons learned:**

1) Maps showed over 61% of uncircumcised men were located in 40% of the wards within the four regions.

2) Community experts provided additional socio‐economic factors needed for consideration in planning VMMC outreach campaigns such as accessibility, availability of water/electricity for sterilization of instruments, and availability of lodging for service providers.

3) The number of men circumcised annually increased three‐fold from 67,414 in 2016 to 225,093 in 2017; over 92% were aged 10 to 29 (PEPFAR priority age group), which was three times higher than previous years.

4) The project achieved these results with a $7.1 million budget, an average of US$31.12 per client, significantly lower than the national estimate and previous years at US$50 and US$39.36, respectively.

**Conclusions/Next steps:** Coupled with qualitative socio‐economic information from community experts, interactive GIS mapping ensures efficiency in planning and monitoring for high‐impact large‐scale interventions at a minimum cost. Project designs should consider using interactive GIS maps to make strategic decisions for targeted high‐impact interventions.

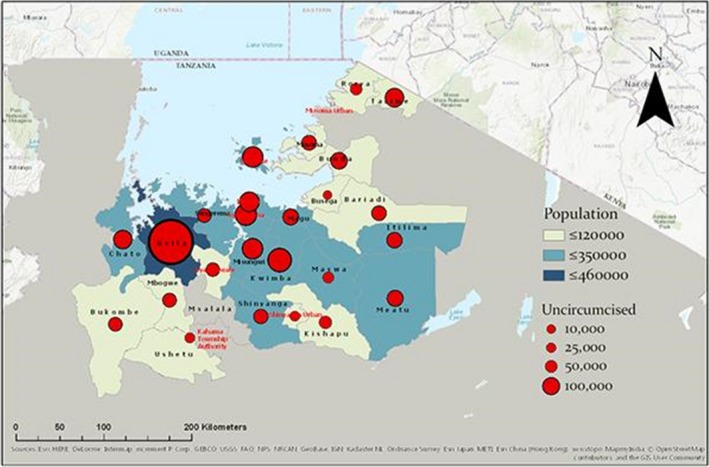

**Abstract TUPDE0106 Figure 1. Distribution of uncircumcised men in IntraHealth‐supported regions in Tanzania.**

## TUPDX0101

### The responsibility of PrEP: a qualitative exploration of men who have sex with men's use of informal PrEP in London

**S. Paparini^1^; W. Nutland^2,3^ and V.‐K. Nguyen^1^**

^1^Graduate Institute of International and Development Studies, Anthropology and Sociology of Development, Geneva, Switzerland, ^2^London School of Hygiene and Tropical Medicine, London, United Kingdom, ^3^PrEPster, London, United Kingdom

**Background:** Concerns have been raised that HIV pre‐exposure prophylaxis (PrEP) will lead to ‘risk compensation’ (that MSM will use condoms less frequently, and increase their sexual risk because of PrEP). In England it has been argued that the drop in HIV incidence can be partially credited to PrEP‐related activism and the initiative taken by MSM as early adopters of informal, online‐purchased, generic PrEP. This suggests the contrary; namely, that PrEP provides an opportunity for men who have sex with men (MSM) to take individual responsibility for their own sexual health. However little research has examined the issue of “responsibility” in PrEP users.

**Methods:** An ethnography of PrEP in the United Kingdom has been carried out since 2016, using interviews, focus groups, and participant observation with policymakers, researchers, clinicians and PrEP users. As part of this, a formative qualitative project was conducted in partnership with PrEP activists to inform an intervention to support informal PrEP users. Focus groups were held with 20 MSM, based in London, who obtain PrEP informally, to explore their accounts and experiences of sourcing and using generic PrEP. This presentation reports findings from the focus groups in the context of the broader ethnography.

**Results: ** In participants’ accounts responsibility was not a single idea but an overarching theme for framing different concerns: control over their own health, relationships with other MSM and the gay community, and citizenship. The responsibility they felt towards themselves created uncertainties about their sexual risk without PrEP and on PrEP. Their responsibility towards their communities was put into practice through information‐sharing and peer support of other potential PrEP‐users. The few clinics that supported their informal PrEP use were seen as taking on the State's responsibility for their health as citizens.

**Conclusions: **The policy conditions in England, and the lack of access to PrEP have given rise to a widespread practice of informal PrEP sourcing and use. This is a self‐directed form of prevention, yet has extended the notion of responsibility for PrEP beyond individual PrEP users to a collective responsibility of PrEP shared by MSM communities, and by PrEP providers to adequately support early adopters and informal users.

## TUPDX0102

### Attitudes regarding HIV, PrEP and condom use jointly predict risk compensation among men who have sex with men – findings from the VicPrEP implementation project, Melbourne

**J. de Wit^1,2^; D. Murphy^2,3^; L. Lal^4,5,6^; J. Audsley^5,7^; C.K. Fairley^8,9^; M. Stoove^4,10^; N. Roth^11^; R. Moore^12^; B.K. Tee^13^; N. Puratmaja^6^; R.M. Grant^14,15,16^ and E. Wright^4,5,6^**

^1^Utrecht University, Department of Interdisciplinary Social Science, Utrecht, Netherlands, ^2^UNSW Sydney, Centre for Social Research in Health, Sydney, Australia, ^3^University of Sydney, Department of Gender and Cultural Studies, Sydney, Australia, ^4^The Burnet Institute, Melbourne, Australia, ^5^The Alfred Hospital, Department of Infectious Diseases, Melbourne, Australia, ^6^Monash University, Department of Infectious Diseases, Melbourne, Australia, ^7^University of Melbourne, The Peter Doherty Institute for Infection and Immunity, Melbourne, Australia, ^8^Melbourne Sexual Health Centre, Melbourne, Australia, ^9^Monash University, Central Clinical School, Melbourne, Australia, ^10^Monash University, School of Population Health and Preventive Medicine, Melbourne, Australia, ^11^Prahran Market Clinic, Melbourne, Australia, ^12^Northside Clinic, Melbourne, Australia, ^13^The Centre Clinic, Melbourne, Australia, ^14^Gladstone Institutes, San Francisco, United States, ^15^University of California‐San Francisco, School of Medicine, San Francisco, United States, ^16^San Francisco AIDS Foundation, San Francisco, United States

**Background:** Potential risk compensation related to PrEP remains a major concern, and may exacerbate high rates of sexually transmissible infections. Recent reports from implementation projects provide initial evidence of reduced condom use amongst among men who have sex with men (MSM) using PrEP in community settings. To increase understanding and inform responses to risk compensation we assessed sociodemographic and attitudinal covariates of trends in condom use with casual partners among VicPrEP participants.

**Methods:** Initiated in 2014, VicPrEP was the first Australian PrEP demonstration project, undertaken through one sexual health clinic and three general practice clinics in Melbourne. A total of 115 participants were enrolled in one year and were offered PrEP for up to 30 months. Participants received baseline and 3‐monthly self‐report questionnaires during the first year of participation. Five‐point rating scales were used to assess condom use (1 = never, 5 = always) and attitudes regarding HIV, PrEP and condoms (1 = low, 5 = high). Prospective data were analyzed using Generalized Estimating Equations.

**Results: ** Frequency of condom use for anal sex with casual partners decreased significantly over one year follow‐up (Baseline Median = 3.0, IQR = 2.0 to 4.0; 12 month Median = 2.0, IQR = 1.0 to 3.0; Wald chi square (df = 4) = 21.03, *p* = 0.000), notably in the first 3 months of using PrEP. Multivariable analysis found that MSM who found HIV a more serious condition and found it more important to remain HIV‐negative were more likely to continue condom use, as were MSM more concerned about adverse effects of PrEP. MSM who considered PrEP critical for personal HIV prevention and MSM who experienced more adverse impacts of condom use on sex were less likely to use condoms.

**Conclusions: **In this community implementation project, condom use with casual partners among MSM decreased markedly upon commencing PrEP. This underscores the importance of stressing PrEP's role as additional HIV prevention tool and that continued condom use contributes to preventing other sexually transmissible infections. Changes in condom use reflect the joint influence of men's attitudes regarding HIIV, PrEP and condoms, offering points of entry for community‐based initiatives to raise awareness and examine risk compensation.

## TUPDX0103

### The new MTV generation: using methamphetamine, Truvada and Viagra to enhance sex and stay safe

**M.A. Hammoud^1^; S. Vaccher^1^; A. Bourne^2^; B. Haire^1^; T. Lea^3^; F. Jin^1^; L. Maher^1^ and G. Prestage^1^**

^1^The Kirby Institute, UNSW Australia, Medicine, Sydney, Australia, ^2^Australian Research Centre in Sex, Health & Society, La Trobe University, Melbourne, Australia, ^3^Centre for Social Research in Health, UNSW, Sydney, Australia

**Background:** Gay and bisexual men (GBM) often use illicit drugs to enhance sexual pleasure, commonly referred to as ‘chemsex’. In particular, the use of methamphetamine and Viagra™ (or other erectile dysfunction medications) both together and separately are strongly predictive of incident HIV infection. Truvada™, as pre‐exposure prophylaxis (PrEP), virtually eliminates HIV risk during condomless anal intercourse (CLAI). When HIV‐negative GBM in intensive sex partying networks add PrEP to their party drug regimen, they actively reduce the possibility of HIV transmission during chemsex. We describe the prevalence and context of concurrent use of methamphetamine (M), Truvada™ (or its generic formulations, T), and Viagra™ (or other erectile dysfunction medication) (V; collectively, MTV).

**Methods:** The *Following Lives Undergoing Change* (FLUX) study is an online prospective observational study of licit and illicit drug use among Australian GBM. Between January and July 2017, 1831 HIV‐negative and untested/unknown status GBM provided details about their use of MTV. Binary logistic multiple regression analyses were used to estimate adjusted odds ratios (aOR) and associated 95% confidence intervals (95% CI).

**Results: ** During 2017, concurrent MTV use was reported by 6.0% of participants; 3.1% used methamphetamine and Viagra™ or other erectile dysfunction medication (‘MV only’) and 11.2% used Truvada™ as PrEP (‘T only’). MTV prevalence in the cohort over time is shown in Figure 1. In multivariate analysis, compared to use of ‘MV only’, MTV was independently associated with CLAI with casual partners (aOR = 6.78; 95% CI = 1.42 to 32.34) and ‘fuckbuddies’ (aOR = 3.47; 95% CI = 1.41 to 8.56) in the previous six months. Compared to use of ‘T only’, MTV was independently associated with being older (aOR = 3.95; 95% CI = 1.55 to 10.03) and engaging in group sex (aOR = 3.31; 95% CI = 1.82 to 6.00). Greater social engagement with other gay men (aOR = 1.44; 95% CI = 1.18 to 1.76) and having more sexual partners (aOR = 2.30; 95% CI = 1.10 to 4.82) were independently associated with use of MTV compared to use of ‘MV only’ or ‘T only’.

**Conclusions: **The addition of PrEP mitigates the increased HIV risk associated with party drug regimens, and these data demonstrate that this harm reduction strategy is being utilised by GBM. Interventions that promote harm reduction strategies, including the use of PrEP during chemsex could help reduce HIV transmissions within this at‐risk population.

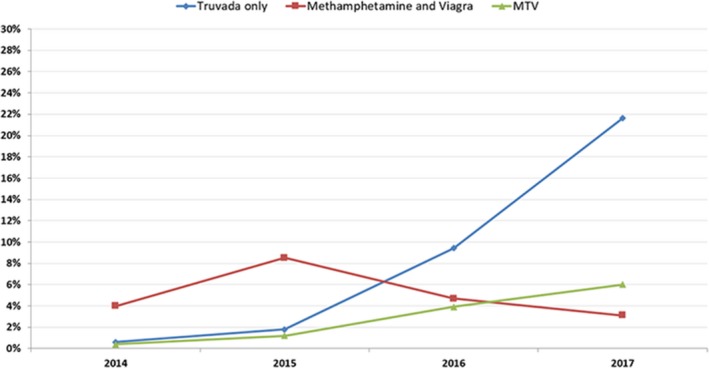

**Abstract TUPDX0103‐Figure 1. Hammoud et al AIDS 2018 MTV.**

## TUPDX0104

### High incidence of hepatitis C virus (re‐) infections among PrEP users in the Netherlands: implications for prevention, monitoring and treatment

**E. Hoornenborg^1^; L.N. Coyer^1^; R.C.A. Achterbergh^1^; M.F. Schim van derLoeff^1^; S. Bruisten^1^; H.J.C. deVries^1,2,3^; T.J.W. Van deLaar^4^; M. Prins^1,2^ and on behalf of the Amsterdam PrEP Project Team in the HIV Transmission Elimination AMsterdam Initiative (H‐TEAM)**

^1^Public Health Service of Amsterdam, Amsterdam, Netherlands, ^2^Academic Medical Center, Amsterdam, Netherlands, ^3^RIVM, Bilthoven, Netherlands, ^4^Sanquin Research, Amsterdam, Netherlands

**Background:** Hepatitis C virus (HCV) prevalence was 4.8% among HIV‐negative men who have sex with men (MSM) starting pre‐exposure prophylaxis (PrEP) in the Netherlands. We studied the HCV incidence rate (IR), characteristics of newly infected individuals, HCV genotype distribution and phylogenetic clustering among MSM and transgender persons (TGP) who use PrEP in the Netherlands.

**Methods:** HIV‐negative MSM (n = 374) and TGP (n = 2) participating in the Amsterdam PrEP project at the Public Health Service of Amsterdam were tested biannually for HCV antibodies, and subsequently for HCV RNA if antibodies were present. We analyzed data from study start (August 2015) through December 2017. We calculated the HCV IR, overall and separately for primary infection and re‐infection, and described baseline characteristics of participants with incident HCV infection. HCV genotyping was performed by sequencing part of the HCV NS5B gene (420 bp). Phylogenetic trees were constructed to compare HCV strains from HIV‐negative participants, HIV‐positive MSM with acute or chronic HCV in Amsterdam and Dutch risk groups other than MSM.

**Results: ** The median follow‐up was 1.76 person years (py) (IQR 1.57 to 1.98). We diagnosed 12 incident HCV infections, all in MSM: 6 primary infections and 6 re‐infections. The overall HCV IR was 1.9/100 py (95% CI 1.1 to 3.4). The IR of primary infection was 1.0/100 py (95% CI 0.5 to 2.2) and of re‐infection 25.5/100 py (95% CI 11.5 to 56.8).

Incident HCV infections were of genotype 1a (n = 9), 4d (n = 1), 2b (n = 1) and 3a (n = 1). Phylogenetic analysis revealed that 8/9 HCV‐1a infections were part of 4 large MSM‐specific HCV‐1a clusters containing MSM with and without HIV (Figure 1)

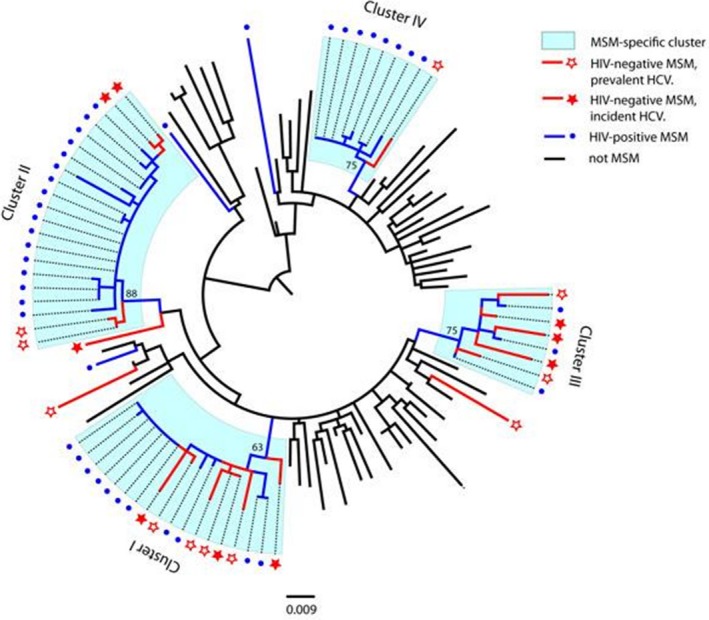

**Abstract TUPDX0104‐Figure 1. Hepatitis C virus (HCV) phylogenetic tree for subtype 1a, comparing HCV sequences from HIV negative MSM with HIV positive MSM and unrelated persons.**

Median age of those with incident infection was 35 years (IQR 26 to 41), most were white (83%), chose for daily PrEP (92%) and reported chemsex (75%) before initiating PrEP (Table 1).

**Abstract TUPDX0104‐Table 1. Characteristics of 12 MSM with incident HCV infection, the Netherlands, 2015 to 2017. All data were collected at the study inclusion visit**

**Table:** 

Age in years, median (IQR)	35 (26 to 41)
White ethnicity, no. (%)	10 (83%)
Living in Amsterdam, no. (%)	5 (42%)
Chose daily PrEP regimen, no. (%)	11 (92%)
Reported chemsex*, no. (%)	9 (75%)
Total number of anal sex partners in preceding three months, median (IQR)	19 (14 to 34)
Number of receptive condomless anal sex acts with unknown partners in preceding three months, median (IQR)	8 (3 to 22)

Chemsex is defined as use of gamma‐hydroxybutyric‐acid, mephedrone or crystal‐methamphetamine around sex

**Conclusions: **In the Netherlands, incidence of initial and re‐HCV infection among HIV‐negative MSM on PrEP was high and comparable to that observed in HIV‐positive MSM. The high degree of phylogenetic clustering between HCV strains acquired by MSM with and without HIV suggests a shared transmission network. Regular HCV testing to provide prompt treatment as well interventions to lower HCV‐related behavior should be offered to MSM on PrEP.

## TUPDX0105

### Expansion of HIV pre‐exposure prophylaxis (PrEP) among key populations in PEPFAR's global program data, fiscal year 2016 to 2017

**G. Djomand^1^; T. Bingham^1^; I. Benech^1^; T. Wheeler^2^; A. Sanicki^3^ and S. Mital^1^**

^1^Centers for Disease Control and Prevention, Division of Global HIV/AIDS and TB, Atlanta, United States, ^2^United States Agency for International Development, Office of HIV/AIDS, Bureau for Global Health, Washington, DC, United States, ^3^Office of the US Global AIDS Coordinator, Washington, DC, United States

**Background:** Key populations (KP), such as men who have sex with men (MSM), female sex workers (FSW) and transgender persons (TG), experience a disproportionate risk of HIV infection compared to the general population (GP). Pre‐exposure prophylaxis (PrEP) represents the preventive use of antiretroviral agents to reduce new HIV infections among HIV‐negative individuals with significant risk behavior. PEPFAR, the largest bilateral funder of HIV programs globally, issued guidance to implement PrEP programming to reduce HIV incidence among KP, developed KP‐specific monitoring indicators and began collecting KP disaggregated data in 2016 to 2017.

**Methods:** Program data on the number of people newly enrolled on oral PrEP are reported on a quarterly basis (Q1‐Q4) as site‐level totals by all partners receiving PEPFAR support. For each quarter, we summed the number across PEPFAR's 36 country or regional programs (CRP) that reported PrEP results among GP and KPs. We also calculated the proportion of all PrEP results contributed by KP and compared percentage change in PrEP enrollment for GP versus all KP groups between Q1 and Q4.

**Results: ** Of the five CRPs that enrolled patients on PrEP in Q1, only three reported enrolling KPs. This increased to 8 of 13 CRPs in Q3 but decreased to 6 of 9 CRPs in Q4. In Q1, 501 KPs newly initiated PrEP (56% of all results) and in Q4, 3717 KPs newly initiated PrEP (34% of all results) (Figure 1). All PrEP enrollment increased 1133% from Q1 to Q4; 1768% among GP and 642% among KPs. Among the KP groups, enrollment increased 445% among FSW, 1580% among MSM, and 341% among TG between Q1 and Q4.

**Conclusions: **Over the four reporting periods in 2016 to 2017, PrEP enrollment increased substantially among both KP and GP, albeit with more attenuated success in scaling this effective HIV prevention intervention among key populations compared to the general population. To address slower growth in enrollment among all KPs, particularly for FSW and TG compared to GP, specific efforts are needed to streamline PrEP scale‐up through advocacy with host country governments to address restrictive national policies and potential gender inequalities.

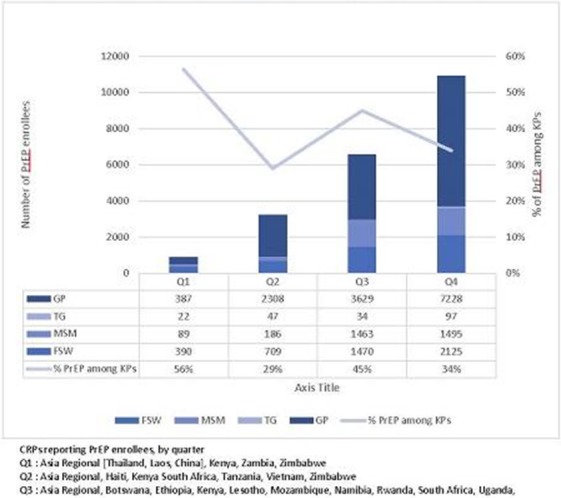

**Abstract TUPDX0105‐Figure 1. Number and percentage of PrEP enrolees among key population and general population clients supported by PEPFAR, October 2016‐September 2017.**

## TUPDX0106

### Altered TDF/FTC pharmacology in a transgender female cohort: implications for PrEP

**M.L. Cottrell^1^; H.M. Prince^2^; K. Maffuid^1^; A. Poliseno^1^; N. White^2^; C. Sykes^1^; J.A. Nelson^3^; A. Peery^2^; E. Dellon^2^; L. Hightow‐Weidman^2^; J.L. Adams^4^; C. Gay^2^ and A.D. Kashuba^1^**

^1^UNC Eshelman School of Pharmacy, Chapel Hill, United States, ^2^University of North Carolina School of Medicine, Chapel Hill, United States, ^3^UNC Center for AIDS Research, HIV/STD Laboratory Core, Chapel Hill, United States, ^4^University of the Sciences, Philadelphia, United States

**Background:** Transgender women (TGW) on feminizing hormone therapy (FHT) maintain estradiol (E2) concentrations 2 to 9 and 6 to 25‐fold higher than cisgender women in the mid‐follicular phase and cisgender men, respectively. E2 increases activity of 5′‐nucleotidase enzymes, which can decrease the active metabolite tenofovir diphosphate (TFVdp) or increase its competing nucleotide (dATP), depending on cellular location. To assess intracellular pharmacology at HIV transmission sites, we measured TFVdp, emtricitabine triphosphate (FTCtp), and their competing nucleotides (dATP and dCTP, respectively) in rectal tissue (RT) of TGW versus postmenopausal cisgender women (CGW; a low E2 control group).

**Methods:** HIV‐infected women on a Truvada^®^ containing regimen with HIV<50 copies/mL and CrCl>60 mL/min enrolled (N = 4 TGW; N = 4 CGW) between 01/2017 and 01/2018. Serum and RT biopsies were collected at a single visit. FHT included oral or injectable E2, medroxyprogesterone, and spironolactone. Serum E2 was measured with validated immunoassay (lower limits of quantification; LLOQ = 20 pg/mL). RT was homogenized and TFVdp/FTCtp and dATP/dCTP were measured by LC‐MS/MS (LLOQ = 0.1 and 0.05 ng/mL, respectively). Values below limits of quantification (BLQ) were imputed at sample specific LLOQ (depending on biopsy size; 4/28 measures). All measures were BLQ for 1 TGW, and excluded from statistical analyses (Student's t‐test and Pearson correlation using SASv9.4). Median (range) summary data are reported.

**Results: ** Age, BMI and CrCl were 42 (34, 46) versus 57 (55, 59) years; 30 (24, 42) versus 36 (27, 38) kg/m^2^; and 114 (100, 192) versus 108 (61, 138) mL/min for TGW versus CGW, respectively. E2 concentrations in TGW (252 (73, 490) pg/mL) were consistent with peak E2 in a typical periovulatory phase. TFVdp:dATP was 7‐fold lower in TGW versus CGW (*p* = 0.006; Table 1). One TGW exhibited TFVdp:dATP below an EC90 target ratio of 0.29. FTCtp:dCTP did not differ between groups and was above an EC90 target ratio of 0.07 in all participants. A significant inverse association was observed for log10E2 and TFVdp:dATP (r = −0.77; *p* = 0.04).

**Conclusions: **This is the first description of TFVdp/FTCtp rectal concentrations in TGW on FHT. TFVdp relative to dATP was significantly lower in TGW and decreased with increasing E2. A cisgender male comparator will also be studied to confirm these findings. These data confirm in vitro findings and suggest that feminizing E2 may impact PrEP efficacy.

**Abstract TUPDX0106‐Table 1. Median (min, max) rectal tissue concentrations**

**Table:** 

Analyte	CGW (N = 4)	TGW (N = 3)
TFVdp (fmol/g)	185158 (76265, 223542)	53674 (23240, 1170302)
FTCtp (fmol/g)	17247 (5959, 23181)	138293 (16303, 289495)
dATP (fmol/g)	7532 (5331, 11301)	547827 (6166, 656011)
dCTP (fmol/g)	6099 (2890, 8058)	80097 (5768, 251048)
TFVdp:dATP	20 (14, 30)	2.7 (0.08, 3.8)
FTCtp:dCTP	2.8 (2.0, 2.9)	2.3 (1.2, 2.9)

## WEPDA0101

### Antiretroviral treatment did not restore functionality of cervical mucosal cells for Th17‐related cytokines altered after HIV infection

**M.P. Caruso^1^; M.P. Holgado^1^; J. Falivene^1^; J. Salido^1^; D.H. Zurita^2^; A. Nico^2^; V. Fink^2,3^; N. Laufer^1,2^; H.M. Perez^2^; P. Cahn^2,3^; O. Sued^3^; G. Turk^1^ and M.M. Gherardi^1^**

^1^Universidad de Buenos Aires, Consejo Nacional de Investigaciones Científicas y Técnicas, Instituto de Investigaciones Biomédicas en Retrovirus y SIDA ^INBIRS^, Facultad de Medicina, Buenos Aires, Argentina, ^2^Hospital General de Agudos “Dr. JA Fernández,” Buenos Aires, Argentina, ^3^Fundación Huésped, Buenos Aires, Argentina

**Background:** Th17 and Treg cells play a key role in HIV infection and mucosal defenses. A reduction of Th17 cells in the female genital mucosa (FGM) of HIV+ women has been previously described.

**Aim**

To analyze the effects of antiretroviral treatment (ART) in these T‐cell subsets in FGM in different groups of individuals.

**Methods:** Cervical mononuclear cells (CMCs) and exocervical swabs from FGM were obtained from the following groups: HIV‐ (n = 21), HIV+ART+(n = 32) and HIV+ART‐ (n = 12). Cytokines (CKs) secreted by CMCs after stimulation with PMA+Ionomycin and chemokines in exocervical swabs were quantified by Cytometric Bead Array.

**Results: ** CMCs production of Th17‐related‐CKs (IL17A, IL17F, IL21 and IL22) and Treg‐related‐CKs (IL10 and TGF‐β1) were evaluated. HIV+ART‐ group showed diminished proportions of positive responses for Th17‐related‐CKs compared to HIV‐ (IL17A, IL17F and IL21: *p* < 0.01). Minor proportions were still found in HIV+ART+ group (IL17A and IL17F: *p* < 0.05). In Treg‐related‐CKs, reduction was only found for IL10 in both HIV+ groups (*p* < 0.05).

Analysis of global pattern of secretion of Th17‐related‐cks, indicated that in HIV+ART‐ the secretion pattern was severely modified (*p* = 0.0297). In HIV+ART+ the Th17‐related‐cks pattern tend to be restored, but a significant lower percentage of samples secreted 4CKs (HIV‐:62.5% vs. HIV+ART+:16.67%; *p* = 0.0281). By contrast, when Treg‐related‐CKs pattern was compared none significant differences were found.

Thirteen different chemokines were evaluated in exocervical swabs. Differences in both HIV+ groups vs. HIV‐ were detected: minor levels of CXCL5 and CXCL1 (neutrophil recruitment); major levels of CCL17 (homing of Treg‐cells).

To inquire if CMCs function deterioration could be related with minor chemokine levels observed, HIV+ART+ group was divided in two according to the number of Th17‐related‐CKs secreted: “at least 3CKs” and “1 or less CKs”.

Interestingly, for CXCL5 and CXCL1, significant lower levels were found in the “1 or less” group (compared to HIV‐ and to “at least 3”, all *p* < 0.05). Also in HIV+ART+, positive correlations were found between logCXCL5 vs. logIL17A (r = 0.5666, *p* = 0.0241), logCXCL1 and logIL17A (r = 0.7273, *p* = 0.0006) and logCCL17 vs. logIL10 (r = 0.5700, *p* = 0.0186).

**Conclusions: **These results suggest that HIV infection severely affects the functionality of cervical mucosal cells, and ART was not able to totally restore it. Earlier ART start might mitigate this HIV infection effect.

## WEPDA0102

### Effect of ART on reducing fungal translocation in HIV‐infected patients

**J.P. Routy^1,2^; V. Mehraj^1,2^; R. Ramendra^3^; C. Costiniuk^1^; B. Lebouché^1^; J. Chen^1,4^; R. Ponte^2^; R. Thomas^5^; B. Trottier^6^; J. Szabo^5^; P. Coté^6^; R. LeBlanc^7^; J.‐G. Baril^6^; N. Bernard^2^; D. Sheppard^2,3^; C. Chartrand‐Lefebvre^8^; M. El‐Far^8^; M. Durand^8^; H. Lu^4^; C. Tremblay^8,9^; Montreal Primary HIV‐infection Study; Canadian HIV and Aging Cohort Study Groups**

^1^Chronic Viral Illness Service, McGill University Health Centre, Montréal, Canada, ^2^Research Institute of McGill University Health Centre, Montréal, Canada, ^3^McGill University, Department of Microbiology and Immunology, Montréal, Canada, ^4^Shanghai Public Health Clinical Center, Infectious Diseases, Shanghai, China, ^5^Clinique Médicale l'Actuel, Montréal, Canada, ^6^Clinique Médicale Quartier Latin, Montréal, Canada, ^7^Clinique Médicale OPUS, Montréal, Canada, ^8^Centre de Recherche du Centre Hospitalier de l'Université de Montréal, Montréal, Canada, ^9^Université de Montréal, Département de Microbiologie, Infectiologie et Immunologie, Montréal, Canada

**Background:** LPS, LBP, and sCD14 are validated markers of microbial translocation in HIV‐infected persons. (1‐3)‐β‐d‐glucan (βDG) is a major component of most fungal cell walls that binds to the extracellular domain of myeloid cells, Dectin‐1. We and others have shown that βDG plasma levels may be considered as a marker of gut fungal translocation in HIV‐infected persons. However, the contribution of ART in improving gut fungal translocation remains to be defined. Herein, we look to assess the role of ART on normalization of βDG levels when initiated during acute (AHI) and chronic HIV infection (CHI).

**Methods:** 177 participants (42 AHI, 93 CHI, and 42 controls) without suspicion of fungal/bacterial infection nor colitis were assessed in a cross‐sectional analysis. 32 AHI patients were longitudinally assessed. Plasma levels of βDG were quantified using Fungitell^®^ assay and were compared with age, sex, viral load, CD4, CD4/CD8 ratio, marker of gut damage (I‐FABP), microbial translocation (LPS, LBP and sCD14), and inflammation (IL1‐β, IL‐6, IL‐8 and TNF‐α).

**Results: ** The mean age of participants was 47.9 ± 12.6 years with 86.4% being male. Plasma βDG levels were elevated during AHI (59.4 ± 33.6 pg/mL, *p* = 0.002) and further increased in CHI (135.6 ± 48.6 pg/mL, *p* < 0.001) vs. seronegative controls (26.7 ± 9.7 pg/mL). We observed a positive correlation of βDG with age (r = 0.351; *p* < 0.001) and with viral load (r = 0.429; *p* < 0.001) as well as a negative trend for CD4 counts (r = −0.135; *p* = 0.125). βDG levels correlated with sCD14 (r = 0.404; *p* = 0.002), IL‐6 (r = 0.404; *p* = 0.001), and IL‐8 (r = 0.640; *p* < 0.001) among HIV‐infected persons. βDG levels increased over 2‐years in the untreated AHI (111.2 ± 96.4 pg/mL *p* < 0.001) and remained stable in the early treated group. Similarly, CHI persons on 13.5 ± 7.0 years of ART did not show a decrease in their βDG levels (135.2 ± 47.3 pg/mL, *p* < 0.001). Multivariate analysis showed βDG elevation was independent of age, sex, CD4 and CD8 counts.

**Conclusions: **The elevation of plasma βDG levels observed during acute and chronic infection did not decrease with early ART initiation nor long‐term ART usage. Elevated βDG levels, which correlated with markers of immune activation, may be directly involved in HIV pathogenesis via Dectin‐1 mediated protein kinase C pathway in myeloid cells.

## WEPDA0103

### Persistence of myeloid cell‐associated inflammation in HIV‐infected children after eight years on early initiated therapy – the key role players in HIV persistence?

**S. Naidoo^1^; K. Veldsman^1^; M.F. Cotton^2^ and R.H. Glashoff^3^**

^1^Stellenbosch University, Medical Virology, Cape Town, South Africa, ^2^Stellenbosch University, Tygerberg Children's Hospital, Cape Town, South Africa, ^3^Stellenbosch University, Division of Medical Microbiology, Cape Town, South Africa

**Background:** Combination antiretroviral therapy (ART) does not completely eradicate HIV latently infected cells. Resting CD4+ T cells remain the most studied source of residual viremia. Research evaluating the role of myeloid lineage cells, such as monocytes and macrophages, in HIV persistence is limited. These long‐lived cell types provide optimal hideouts for the virus and are less susceptible to HIV‐induced cytopathic effects and death. Evaluating the interplay of the immune mechanisms of myeloid cells and HIV persistence within a pediatric population may provide valuable insight into therapeutic targets for eradicating latent reservoirs.

**Methods:** Plasma samples originating from the Children with HIV‐Early Antiretroviral Therapy (CHER) trial were evaluated. ART was initiated at <1 year of age and children sustained viral suppression at seven to eight years. Cytokines (IL‐1β, IL‐6, IL‐8, IL‐10, INF‐γ, TNF‐α, TGFβ1,2,3, sCD14, sCD163, GCSF, CMCSF, VEGF) and chemokines (MCP‐1, MIP‐1α, MIP‐1β, LBP) involved in monocyte/macrophage activation and trafficking were measured using Luminex^®^ Multiplex assays. Age‐matched controls were measured for the same biomarkers. A subset of HIV‐infected participants was tested for total HIV‐1 DNA using qPCR targeting a conserved region in HIV integrase. Statistical analysis employed a Wilcoxon matched paired test for nonparametric data.

**Results: ** 163 samples (88 HIV‐infected and 75 HIV‐uninfected controls) were evaluated. The median baseline viral load at ART initiation was 738,500.5 copies/mL. Median CD4‐percentage at baseline was 36.9% (range: 23.1% to 57.1%). At seven to eight years of age, there were no significant differences between the CD4‐percentage of the HIV‐infected (38.3%) and control groups (40.0%) (*p* = 0.261). HIV‐infected children showed highly significant (*p* < 0.001) levels of IL‐1β, IL‐6, TGFβ3, sCD14, sCD163, MCP‐1, MIP‐1a, MIP‐1b, GCSF, CMCSF, LBP, and VEGF when compared to controls. Significant increase in IL‐8 (*p* = 0.0450), TNFa (*p* = 0.0033), TGFb1 (*p* = 0.0140) and TGFβ2 (*p* = 0.0042) were also observed. Among 32 children assessed for HIV‐1 DNA at follow‐up, a median of 32.5 copies/million cells (range: 0 to 562.6) was observed at seven to eight years of age.

**Conclusions: **Despite early therapy initiation, long‐term viral suppression, low cell‐associated HIV‐1 DNA detection and normalized CD4 counts, HIV‐infected children display persistent myeloid‐cell associated inflammation which may drive ongoing low‐level replication. The increase in sCD14 and LBP levels implicate bacterial gut translocation.

## WEPDA0104

### Immune activation parameters are differentially expressed across four countries in sub‐Saharan Africa and are associated with comorbidities in HIV+ and HIV‐ individuals

**H. Streeck^1^; G. Son^1^; D. Habermann^2^; T.A. Crowell^3,4^; A. Esber^3,4^; L.A. Eller^3,4^; M.A. Eller^3,4^; A.P. Parikh^3,4^; P.A. Horn^5^; L. Maganga^6^; Y. Adamu^7^; F. Kiweewa^8^; J. Maswai^3,9,10^; J. Owuoth^3,10,11^; M.L. Robb^3,4^; N.L. Michael^3^; D. Hoffmann^2^; C.S. Polyak^3,4^ and J.A. Ake^3^**

^1^Institute for HIV Research, University Hospital, University Duisburg‐Essen, Essen, Germany, ^2^Bioinformatics and Computational Biophysics, University Duisburg‐Essen, Essen, Germany, ^3^U.S. Military HIV Research Program, Walter Reed Army Institute of Research, Silver Spring, United States, ^4^Henry M. Jackson Foundation for the Advancement of Military Medicine, Bethesda, United States, ^5^Institute for Transfusion Medicine, University Hospital, University Duisburg‐Essen, Essen, Germany, ^6^Mbeya Medical Research Centre, Mbeya, Tanzania, United Republic of, ^7^U.S. Military HIV Research Program, Walter Reed Army Institute of Research, Abuja, Nigeria, ^8^Makerere University‐Walter Reed Project, Kampala, Uganda, ^9^Henry M. Jackson Foundation Medical Research International, Kericho, Kenya, ^10^Kenya Medical Research Institute, Nairobi, Kenya, ^11^Henry M. Jackson Foundation Medical Research International, Kisumu, Kenya

**Background:** Immune activation is a significant contributor to HIV pathogenesis and disease progression. In ART virally‐suppressed individuals, low‐level immune activation has been linked to several non‐infectious comorbid diseases (NCDs). However, such studies have not been systematically performed in sub‐Saharan Africa and thus the impact of demographics, cART and regional endemic co‐infections on immune activation is not known. We therefore comprehensively evaluated in a large multinational African cohort markers for immune activation and its distribution in various settings.

**Methods:** In total, 2747 specimens from 2240 HIV‐positive (1492 on cART, 748 without cART) and 477 HIV‐negative individuals from the observational African Cohort Study (AFRICOS) were analyzed for 13 immune parameters. Samples were collected together with medical history, sociodemographic and comorbidity data at 11 HIV clinics across five programs in Uganda, Kenya, Tanzania and Nigeria. Data were analyzed with univariate and multivariate methods such as random forests and principal component analysis.

**Results: ** Immune activation was markedly different between HIV‐positive with detectable viral load and HIV‐ individuals across sites (*p* < 0.001), but only differed in some markers between HIV+ cART+ (<50 copies/mL) and HIV‐ individuals. Random Forest analysis revealed that immune activation parameters could successfully predict the country origin of the specimens (*p* < 0.001) as well as to lesser degree the level of education. In particular, CCL2 and IL2RA were significantly diametrically expressed in the five regions (*p* < 0.001). This was the case across HIV‐positive and HIV‐negative individuals and was not due to other infections such as hepatitis B, C, tuberculosis or co‐trimoxazole prophylaxis. Within the 2240 HIV‐positive individuals, our study revealed significant gender specific immune activation expression patterns that were not present in HIV‐negative individuals. Moreover, when we compare HIV‐negative individuals and HIV‐positive individuals with fully suppressed viremia, the latter group has a significantly larger number of NCDs, an effect that increases with age (*p* = 0.0069). These two groups can be distinguished based on diverging immune parameters such as CXCR10 and IL2RA.

**Conclusions: **We demonstrate region‐specific, gender‐specific and education‐specific differences in immune activation expression. Furthermore, several immune activation markers are differentially expressed in individuals with NCDs an effect that significantly increases with age.

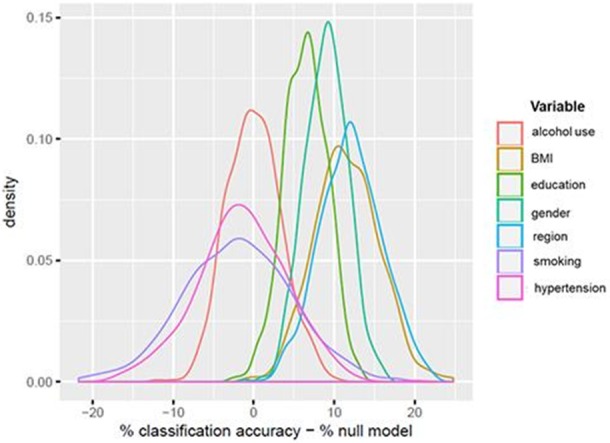

**Abstract WEPDA0104‐Table 1. Random Forrest Analysis of Immune Parameters Predicting Region, Gender and Education in HIV+ individuals**

## WEPDA0105

### Anticoagulant therapies alleviate SIV‐associated hypercoagulation as well as immune activation and inflammation

**T. He^1,2^; G. Haret‐Richter^2^; B. Andrade^3^; C. Xu^2^; B. Policicchio^2^; P. Sette^2^; K. Raehtz^2,4^; E. Falwell^2^; T. Dunsmore^2^; K. Martin^2^; G. Padovani^2^; I. Francischetti^5^; C. Apetrei^2,4^; I. Sereti^6^ and I. Pandrea^1,2^**

^1^University of Pittsburgh School of Medicine, Pathology, PITTSBURGH, United States, ^2^University of Pittsburgh, Center for Vaccine Research, Pittsburgh, United States, ^3^Instituto Brasileiro para a Investigação da Tuberculose, Multinational Organization Network Sponsoring Translational and Epidemiological Research (MONSTER) Initiative, Salvador, Brazil, ^4^University of Pittsburgh School of Medicine, Department of Microbiology and Molecular Genetics, Pittsburgh, United States, ^5^National Institute of Allergy and Infectious Diseases, Laboratory of Malaria and Vector Research, Bethesda, United States, ^6^National Institute of Allergy and Infectious Diseases, HIV Pathogenesis Section, Laboratory of Immunoregulation, Bethesda, United States

**Background:** Cardiovascular disease remains one of the leading non‐AIDS causes of death among chronically HIV‐infected subjects, fueled by a hypercoagulable state along with persistent immune activation and inflammation (IA/INFL). Tissue factor (TF) and its downstream thrombin production may bridge IA/INFL and hypercoagulation via protease‐activated receptor (PAR) signaling. We hypothesize that anticoagulant therapies targeting these coagulation pathways can break the vicious cycle of hypercoagulation and IA/INFL and reduce the risk of cardiovascular comorbidity in HIV infection.

**Methods:** We experimentally administered a thrombin inhibitor (Dabigatran) and a PAR‐1 inhibitor (Vorapaxar) in five SIV‐infected pigtail macaques (PTMs) each, and compared with a TF inhibitor (Ixolaris) treatment in PTMs reported before. Five untreated SIV‐infected PTMs were used as controls. Treatments were initiated at the time of infection for at least 80 days. Coagulation markers (D‐dimer, sTF), endothelial and platelet activation markers (sICAM‐1, sP‐selectin), as well as immune activation (CD38/HLA‐DR, Ki‐67, CD80, CD86) and inflammation markers (C reactive protein (CRP), proinflammatory and anti‐inflammatory cytokines) were measured throughout and after treatment.

**Results: ** All three anticoagulant therapies resulted in a different extent of reduction in hypercoagulation, marked by lower levels of D‐dimer, sTF, sICAM‐1 and sP‐selectin compared with controls after SIV infection. Treated PTMs also showed lower CRP, lower proinflammatory cytokines and chemokines (IL‐1β, IL‐17, Eotaxin, MIP‐1α, VEGF) and higher protective cytokine IL‐12. Meanwhile, treated PTMs had lower activated monocytes marked by CD80 and CD86. Among the anticoagulant therapies, Ixolaris had the best effect in reducing D‐dimer levels and IA/INFL markers. Additionally, besides reduced monocyte activation, Ixolaris also lowered T cell activation (CD38^+^ HLA‐DR^+^ coexpression). Ixolaris‐treated PTMs also had a better survival, with no progression to AIDS within 100 days post infection.

**Conclusions: **Our results reinforced the close causative relationship between hypercoagulation and IA/INFL in SIV pathogenesis. Anticoagulant therapies may thus represent effective strategies not only for reducing hypercoagulation but also to alleviate IA/INFL. Targeting TF directly, exerted a better effect compared with anticoagulant therapies targeting downstream players of the coagulation pathway, indicating that interventions prior to the establishment of coagulation‐inflammation vicious cycle may be more successful. Future studies to the effect of anticoagulants in viral‐suppressed setting are warranted.

## WEPDB0101

### Validation of Alere ™ q HIV‐1/2 detect for detection of acute HIV infection at Anonymous Clinic, The Thai Red Cross AIDS Research Centre

**I. Srila‐Or^1^; T. Pankam^1^; S. Pattanachaiwit^1^; S. Barisri^1^; P. Raprasert^1^; J. Jantarapakde^1^; S. Areeyowattana^1^; S. Sirivichayakul^2^; P. Phiayura^1^; R. Ramautarsing^1^; N. Phanuphak^1,3^ and P. Phanuphak^1,2^**

^1^The Thai Red Cross AIDS Research Centre, Laboratory, Bangkok, Thailand, ^2^Chulalongkorn University, Faculty of Medicine, Bangkok, Thailand, ^3^SEARCH, Bangkok, Thailand

**Background:** Diagnosis of acute HIV (AHI) is important to facilitate early linkage to treatment, to reduce onward HIV transmission and to reduce the risk of developing drug resistance in people taking pre‐exposure prophylaxis (PrEP). Qualitative detection of HIV type‐1 and type‐2 by nucleic acid amplification test (NAAT) for the diagnosis of AHI is labor intensive and is limited to facility‐based testing. The Alere™q HIV‐1/2 Detect (Alere™q) is a point‐of‐care, qualitative, cartridge‐based NAAT which allows its use in community‐based settings. We assessed the performance of Alere™q in AHI detection.

**Methods:** Ten HIV‐uninfected and 90 AHI (30 fourth generation immunoassay (G)+/third G+/second G‐, 30 fourth G+/third G‐/second G‐, and 30 fourth G‐/NAAT+) stored plasma samples (at −80°C for up to 5 years prior to use) were selected. All samples were tested by Alere™q. Sensitivity, specificity, NPV, and PPV were calculated. NAAT was performed using APTIMA HIV‐1 Qualitative Assay. Quantitative HIV‐RNA (Roche Molecular Systems, Inc., Mannheim, Germany) measured from all 90 AHI samples on the day of diagnosis were retrieved from database.

**Results: ** Alere™q was detectable in 80/90 (88.9%) AHI samples: 29/30 (96.7%) 4^th^G+/3^rd^G+/2^nd^G‐, 30/30 (100%) 4^th^G+/3^rd^G‐/2^nd^G‐, and 21/30 (70%) 4^th^G‐/NAAT+ samples. Sensitivity, specificity, PPV, and NPV for AHI detection were 88.9%, 100%, 100% and 50%, respectively. Median (IQR) HIV RNA level of 10 AHI samples with undetectable Alere™q was 282 (29 to 40,311 copies/mL) which mainly (9/10) had HIV‐RNA <log10 3.40 and the other one had HIV RNA 40,311 copies/mL (log10 4.61). Median (IQR) HIV RNA of 80 AHI samples with detectable Alere™q was 630,276 (165 to 17,515,700 copies/mL which mainly (75/80) had HIV‐RNA >log10 3.40).

**Conclusions: **Alere™q demonstrated high specificity and PPV to detect AHI from stored samples. The low sensitivity was 88.9% in our study, although could not completely exclude potential HIV RNA degradation due to storage, could be a concern if Alere™q will be used to screen for AHI. Alere™q missed one AHI cases who had HIV RNA above 2500 copies/mL (log10 3.40). More data on the sensitivity of Alere™q on real‐time samples from HIV testing or PrEP facilities is needed to inform its potential role in community‐based testing.

## WEPDB0102

### Favorable clinical phenotype reached in less than half of people treated in acute HIV infection

**J. Ananworanich^1,2,3^; S. Pinyakorn^1,2^; A. Avihingsanon^4^; J. Sophonphan^4^; C. Sacdalan^5^; E. Kroon^5^; D. Colby^5^; D. Suttichom^5^; P. Prueksakaew^5^; S. Ubolyam^4^; R. Trichavaroj^6^; M. de Souza^1,2,5^; N. Michael^2^; M.L. Robb^1,2^; P. Phanuphak^4,5^; N. Phanuphak^5^; on behalf of the RV254/SEARCH 010 and HIV‐NAT 006 Study Groups**

^1^The Henry M. Jackson Foundation for the Advancement of Military Medicine, Bethesda, United States, ^2^United States Military HIV Research Program; Walter Reed Army Institute of Research, Silver Spring, United States, ^3^Department of Global Health, University of Amsterdam, Amsterdam, Netherlands, ^4^HIV‐NAT, The Thai Red Cross AIDS Research Center, Bangkok, Thailand, ^5^SEARCH, The Thai Red Cross AIDS Research Centre, Bangkok, Thailand, ^6^Armed Forces Research Institute of Medical Sciences, Bangkok, Thailand

**Background:** Antiretroviral therapy (ART) in acute HIV infection (AHI) improves immune recovery. Viral load (VL) suppression, CD4 T cell count (CD4) and CD4/CD8 ratio are clinical markers for HIV‐associated morbidity and mortality. There are limited data on the proportion of treated AHI individuals who attain a favorable clinical phenotype (FCP).

**Methods:** Analysis included data from participants in the RV254/SEARCH 010 AHI cohort in Thailand who were enrolled between April 2009 and June 2017 and were ART naïve at enrollment and on ART for ≥24 weeks. FCP was defined as fulfilling all 3 criteria (1) VL <20 copies/mL at all visits from week 24 onwards; (2) Last CD4 >500 cells/mm^3^; (3) Last CD4/CD8 ratio >1. CD4, CD8 and VL were performed every three months. Mann‐Whitney U and Fisher's exact tests were used to compare continuous and binary outcomes, respectively, between groups. Logistic regression determined factors associated with favorable outcomes.

**Results: ** 246 AHI participants in Fiebig stages I to V were included of whom 96% were male. Baseline median (IQR) values were age of 27 (23 to 32) years, CD4 of 376 (267 to 505) cells/mm^3^, CD4/CD8 ratio of 0.8 (0.4 to 1.1), and VL of 5.8 (5.3 to 6.8) log10 copies/mL. The majority (99%) initiated efavirenz‐based regimens. The median (IQR) ART duration was 3.2 (2.6 to 4.4) years. FCP was achieved in 40% with differences between Fiebig I and Fiebig V (*p* = 0.046) (Figure). Proportions achieving each outcome were 76% for VL suppression, 81% for CD4 >500 cells/mm^3^and 59% for CD4/CD8 ratio >1. FCP was associated with pre‐ART CD4 (OR 1.27, 95% CI 1.11 to 1.46, *p* = 0.001) and CD4/CD8 ratio (OR 4.31, 95% CI 2.42 to 7.67, *p* < 0.001) but not VL, age or sex. In comparison, FCP was less likely in a chronic HIV Thai cohort (25%, *p* = 0.002) of 271 males who were older (31 years (25 to 37)) but had longer duration of ART (5.3 years (4.0 to 6.3)).

**Conclusions: **Despite initiating ART as early as Fiebig I/II AHI, less than half achieved favorable clinical markers of persistent viral suppression, normalized CD4 and CD4/CD8 ratio. Understanding the pathogenesis that distinguishes these clinical phenotypes may be important in improving therapy and developing remission strategies.

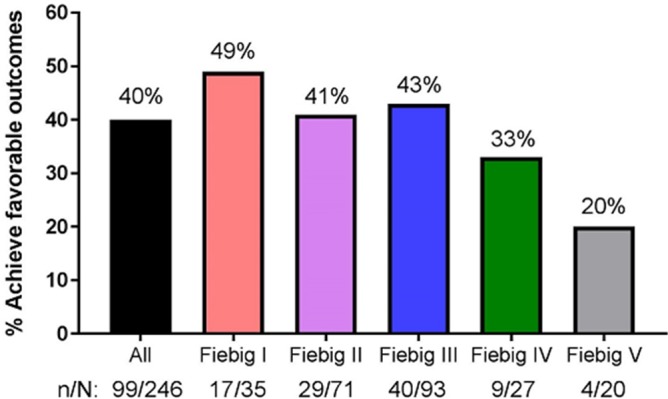

**Abstract WEPDB0102‐Figure 1. Proportions with favorable clinical phenotype after treatment.**

## WEPDB0103

### Increasing contribution of integrated forms to total HIV1‐DNA in blood, in primary infection during natural history – ANRS PRIMO and SEROCO cohorts

**P. Tremeaux^1^; T. Lenfant^1^; F. Boufassa^2^; A. Essat^3^; A. Melard^4^; M. Gousset^4^; O. Delelis^5^; J.‐P. Viard^1^; M. Bary^6^; C. Goujard^7^; C. Rouzioux^4^; L. Meyer^8^; V. Avettand‐Fenoel^4,9^; SEROCO and PRIMO ANRS Cohorts**

^1^Paris Descartes University, Paris, France, ^2^Université Paris Sud, Bicetre, France, ^3^Inserm CESP U1018, Bicetre, France, ^4^Université Paris Descartes, Paris, France, ^5^Centre National de la Recherche Scientifique UMR8113, Cachan, France, ^6^AP‐HP, Hôpital Bicêtre, Kremlin Bicetre, France, ^7^AP‐HP, Hôpital Bicêtre, Bicetre, France, ^8^INSERM CESP U1018, Bicetre, France, ^9^Necker Hospital, APHP, Paris, France

**Background:** HIV1‐DNA persistence in infected cells is the main hurdle preventing eradication. Markers estimating HIV reservoir size have been mostly studied in treated patients but less in natural history. Total cell‐associated HIV‐DNA includes integrated forms and more labile unintegrated forms, while integrated HIV‐DNA represents the most stable and productive form in infected cells. This study aimed to describe the blood dynamics over years of total HIV‐DNA and integrated HIV‐DNA during primary infection (PHI) and in recent to chronic infections and to AIDS, in untreated patients.

**Methods:** Both markers were quantified from frozen PBMC of 74 PHI patients from the ANRS‐PRIMO cohort and 97 recent seroconverters (<12 months since contamination) from the ANRS‐SEROCO cohort for whom at least two cell samples were available. Total HIV‐DNA and integrated HIV‐DNA evolutions were modeled (mixed‐effect linear models) and their predictive values were studied (Cox models).

**Results: ** High levels of total HIV‐DNA (median 3.59 log10 copies/10^6^ PBMCs (IQR: 3.29 to 4.03)) were observed at PHI with low levels of integrated forms among total HIV‐DNA for most patients (2.15 log10 copies/10^6^ PBMCs (IQR: 0.95 to 3.16)), suggesting a major proportion of unintegrated forms in PHI. Among recent seroconverters, those who progressed towards AIDS during the study (Rapid Progressors, n = 34) had higher total HIV‐DNA and integrated HIV‐DNA levels at inclusion compared to others (Progressors, n = 63), and higher integrated/total HIV‐DNA ratio (100% vs. 44%, respectively). In multivariate analysis, integrated HIV‐DNA load was strongly associated with the risk of developing AIDS (aRR = 2.6, *p* = 0.002). A total of 340 sequential samples were available. Parallel rates of increase were observed for both markers over six years follow up in Rapid Progressors and Progressors, with the highest levels observed in patients with AIDS (ratio at 100%).

**Conclusions: **The low contribution of integrated forms at PHI indicates that the stable reservoir is not completely established. These results may partly explain the high benefice of early treatment on total HIV‐DNA, preventing its progressive increase and controlling formation of unintegrated HIV‐DNA. The risk of “rapid” or “slow” progression seemed to be determined early in the course of infection, enhancing the crucial need for early diagnosis and treatment implementation.

## WEPDB0104

### Intermittent viremia after treatment interruption increased risk of ART resumption in post‐treatment HIV‐1 controllers. ANRS VISCONTI study

**L. Hocqueloux^1^; V. Monceaux^2^; V. Avettand‐Fènoël^3,4^; S. Orr^5^; F. Boufassa^5^; O. Lambotte^6^; T. Prazuck^1^; P. Miailhes^7^; D. Salmon^8^; C. Lascoux‐Combes^9^; A. Lafeuillade^10^; L. Meyer^5^; M. Müller‐Trutwin^2^; C. Rouzioux^3,4^; A. Saez‐Cirion^2^ and ANRS VISCONTI Study**

^1^CHR d'Orléans – La Source, Infectious and Tropical Diseases, Orléans, France, ^2^Institut Pasteur, HIV Inflammation and Persistance, Paris, France, ^3^CHU Necker – Enfants Malades, APHP, Virology, Paris, France, ^4^Université Paris Descartes, Paris, France, ^5^INSERM CESP U 1018, University Paris Sud, APHP Bicetre Hospital, Le Kremlin‐Bicetre, France, ^6^ImVA UMR 1184, Le Kremlin‐Bicetre, France, ^7^CHU de la Croix‐Rousse, Service des Maladies Infectieuses, Lyon, France, ^8^CHU Cochin, APHP, Service des Maladies Infectieuses, Paris, France, ^9^CHU Saint‐Louis, APHP, Service des Maladies Infectieuses, Paris, France, ^10^Hôpital Général, Service des Maladies Infectieuses, Toulon, France

**Background:** Some HIV‐1‐infected individuals achieve durable virological remission after discontinuation of antiretroviral therapy (ART). Because remission is a major objective in the global strategy towards an HIV cure, a better characterization of this phenomenon is necessary. We report here the long‐term outcome of 23 post‐treatment controllers (PTC) since their enrollment in a French study.

**Methods:** PTC were defined as HIV‐1‐infected individuals who achieved viral suppression (plasma viral load (pVL) <400 copies/mL) for at least 12 months after treatment interruption. These cases have been prospectively followed in the ANRS VISCONTI study since 2008. Intermittent viremia was defined after treatment interruption as a transient pVL >400 copies/mL, and virologic failure (VF) as 2 consecutive pVL >400 copies/mL. Inflammation markers (IL‐18, sCD14, CRP, IP‐10, I‐FABP and sCD163) were analyzed in plasma from 11 PTC at enrollment in the VISCONTI study.

**Results: ** Twenty‐three PTC (one case of mother‐to‐child transmission) were included, all of whom had started ART at the time of primary‐infection (PHI). Main characteristics (median or %) at PHI were: age = 34 years, sex male = 65%, Caucasian = 70%, MSM = 48%, symptomatic PHI = 87%, pVL = 5.2 log copies/mL. ART was stopped after a median 3.7 years. Thereafter, PTC were followed‐up for a median 11.9 years (until last visit or ART resumption). Overall, CD4 count and CD4/CD8 ratio were stable over time. Median total HIV‐DNA was 1.85 log copies/10^6^ PBMC at enrollment. Intermittent viremia was noted in 7 PTC (30%). Five patients (22%) resumed ART after a median 8.6 years off‐ART: four because of VF, one for a non‐AIDS defining cancer. Patients who had intermittent viremia were more likely to resume ART than those without: 5/7 versus 0/16, respectively (*p* = 0.0006). PTC without intermittent viremia presented low levels of inflammation when compared to HIV controllers or HIV‐infected individuals on ART (*p* < 0.05 for IL‐18, sCD163 and sCD14).

**Conclusions: **Among PTC, one third presented an intermittent viremia after treatment interruption and were at increased risk of ART resumption. By contrast, the other PTC who had tighter control of viral replication maintained their status during follow up and had close to normal inflammation levels.

## WEPDB0105

### Auranofin plus nicotinamide impact HIV reservoir among ART suppressed HIV individuals

**L.B. Giron^1,2^; J. Hunter^2^; J. Galinskas^2^; D. Dias^2^; P.R.A. Ferreira^2^; S. Tenore^2^; G. Gosuen^2^; S. Samer^2,3^; M.S. Arif^2,4^; E.D. Libera^2^; A. Savarino^5^; L.M.R. Janini^2^; M.C.A. Sucupira^2^ and R.S. Diaz^2^**

^1^The Wistar Institute, Philadelphia, United States, ^2^Federal University of Sao Paulo, Sao Paulo, Brazil, ^3^Emory University, Atlanta, United States, ^4^North Western University, Chicago, United States, ^5^Istituto Superiore di Sanità, Rome, Italy

**Background:** Multiple interventional strategies may be fundamental to decrease the size of HIV‐1 reservoir along with antiretroviral therapy (ART). To measure the impact of isolated and combined strategies in decreasing total HIV‐1 DNA, we investigated the effect of treatment intensification with Dolutegravir (DTG) with and without Maraviroc (MVC), Nicotinamide (NA), and Auranofin. NA is a class III HDAC inhibitor with anti‐lymphoproliferative effect, and Auranofin induced decay in viral DNA of ART treated SIVmac251‐infected macaques.

**Methods:** Data from six arms of NCT02961829 with five patients each followed every four weeks for a total of 48 weeks were analyzed. Selected patients were ART suppressed for >2 years, with CD4 nadir >350. Groups were: 1) continuation of ART, 2) intensified ART (ART+DTG and MVC), 3) intensified ART and HDACi (ART+DTG+MVC+NA), 4) intensified ART and Auranofin (ART+DTG+MVC+Auranofin), 5) partially intensified ART (ART+DTG), 6) partially intensified ART (DTG)+NA+Auranofin. Auranofin was used for the first 24 weeks of the study in G4 and G6. After week 48, Total viral DNA was measured by qPCR in PBMCs and rectal biopsy tissues, this latter performed at baseline.

**Results: ** Treatment intervention was well tolerated, and main adverse events (AE) were anxiety and sleep disorders, attributable to efavirenz/dolutegravir interaction. There were transient non‐statistic significant decreases in CD4 counts at weeks 8 and 12 from baseline in auranofin groups, (week 8: −119.3 ± 194.7; week 12: −187 ± 210.7 cells/mL). A decrease in viral DNA was observed in G6 as compared to all other groups. (*p* = 0.022; Odds ratio: 9.75, 95% CI: 1.1 to 72.39). Intensified ART with DTG+MVC presented higher decrease in the total DNA as compared to intensified ART with DTG only (G2 vs. G5, *p* = 0014). All individuals presented undetectable viral loads throughout the study, but G1 showed a significant linear trend towards an increase of the viral reservoir (*p* < 0.05). There was no correlation between proviral DNA from PBMCs and rectal biopsy tissues at baseline.

**Conclusions: **The interim analysis of this phase II trial suggests that NA+auranofin administration in combination with intensified ART is well tolerated, and an impact on the proviral reservoir size is possible.

## WEPDB0201

### TB/HIV co‐treatment with super‐boosted lopinavir and anti‐tuberculosis treatment lowers abacavir concentration in children

**H. Rabie^1^; T. Tikiso^2^; H. McIlleron^2^; J. Lee^3^; I. Andrieux‐Meyer^3^; M. Cotton^1^; M. Lallemant^3^ and P. Denti^2^**

^1^University of Stellenbosch, Tygerberg Hospital and Family Clinical Research Unit, Department of Pediatrics and Child Health, Cape Town, South Africa, ^2^University of Cape Town, Division of Clinical Pharmacology, Cape Town, South Africa, ^3^Drugs for Neglected Diseases Initiative (DNDi), Geneva, Switzerland

**Background:** The available data describing the pharmacokinetics and efficacy of abacavir given with super‐boosted lopinavir (LPV/r plus additional ritonavir), rifampicin, and other anti‐TB drugs is limited. Our objective was to compare pharmacokinetics of abacavir during treatment with standard doses of LPV/r vs. anti‐TB treatment and super‐boosted lopinavir.

**Methods:** 87 TB/HIV‐infected South African children (median, range age: 2.8, 0.25 to 6 years; weight: 9.4, 4 to 16 kg) were sampled on three separate visits: after at least two weeks on TB treatment and super‐boosted lopinavir during the intensive phase and end of TB treatment; and one month after TB treatment completion on standard doses of LPV/r dose without additional ritonavir. Abacavir twice‐daily was co‐administered throughout. All drugs were dosed according to the South African weight‐band dosing recommendations. At each visit, blood samples were collected immediately before dosing and one, two, four, five, eight, and ten hours after. NONMEM 7.3 was used to develop a population pharmacokinetic model.

**Results: ** Abacavir pharmacokinetics was best described by a two‐compartment model with first‐order elimination and transit compartment absorption. Allometric scaling was used to adjust for the effect of body size, after which maturation could be identified: clearance was predicted to reach half its mature value at around two months after birth and to be fully mature by around two years of age. The typical clearance in a 9 kg child co‐treated with normal dose LPV/r is estimated at 8.8 L/h. During co‐administration of TB treatment with lopinavir super‐boosting, a 38% decrease in bioavailability (and AUC) was found. Finally, the trough concentrations observed just before the morning dose were higher than the extrapolated values predicted 12 hours after a morning dose, best explained by a 24% reduction in clearance overnight.

**Conclusions: **The proposed model successfully characterised the PK of abacavir, including the effect of body weight and age. Abacavir exposure was decreased by concomitant administration of rifampicin and super‐boosted lopinavir. Larger trough concentrations were observed in the morning, possibly indicating circadian variation in the pharmacokinetics. Although 67 (82%) children were virologically suppressed at the end of TB treatment compared to 6 (6%) at study entry, further investigation should address whether dosing adjustments are necessary.

## WEPDB0202

### Efavirenz plasma exposure and immunologic outcome during anti‐tuberculosis co‐therapy: role of ethnicity and pharmacogenetic variations

**S. Mugusi^1^; W. Amogne^2^; A. Habtewold^3^; E. Ngaimisi^4^; G. Yimer^2^; O. Minzi^4^; E. Makonnene^3^; F. Mugusi^5^; C. Sudfeld^6^; M. Janabi^7^; J. Burhenne^8^ and E. Aklillu^9^**

^1^Muhimbili University of Health and Allied Sciences, Clinical Pharmacology, Dar es Salaam, Tanzania, United Republic of, ^2^Addis Ababa University, Internal Medicine, Addis Ababa, Ethiopia, ^3^Addis Ababa University, Pharmacology, Addis Ababa, Ethiopia, ^4^Muhimbili University of Health and Allied Sciences, Clinical Pharmacy and Pharmacology, Dar es Salaam, Tanzania, United Republic of, ^5^Muhimbili University of Health and Allied Sciences ^MUHAS^, Internal Medicine, Dar es Salaam, Tanzania, United Republic of, ^6^Harvard T.H. Chan School of Public Health, Global Health and Population, Boston, United States, ^7^Muhimbili University of Health and Allied Sciences, Internal Medicine, Dar es Salaam, Tanzania, United Republic of, ^8^University of Heidelberg, Clinical Pharmacology and Pharmacoepidemiology, Heidelberg, Germany, ^9^Karolinska Institutet, Division of Clinical Pharmacology, Department of Laboratory Medicine, Stockholm, Sweden

**Background:** Efavirenz (EFV) containing antiretroviral therapy (ART) is the regimen of choice for tuberculosis (TB)‐HIV co‐infected patients on anti‐TB therapy. Efavirenz is primarily metabolised via hepatic cytochrome P450 and has considerable interpatient variability. We investigated the role of ethnicity and pharmacogenetic variations in EFV plasma exposure.

**Methods:** We conducted a multi‐centred prospective cohort study of TB‐HIV co‐infected adults with a CD4 count of <200 cells/mL in Ethiopia and Tanzania. Patients were initiated on rifampicin‐based anti‐TB therapy, and four weeks later EFV‐based ART was also initiated and there patients were followed for one year. Efavirenz plasma concentrations were measured at four and sixteen weeks post ART initiation. Genotyping for functional *CYP2B6, CYP3A5, ABCB1, UGT2B7* and *SLCO1B1* variant alleles was conducted.

**Results: ** A total of 427 TB‐HIV co‐infected patients were included in the analysis (231 Tanzanians and 196 Ethiopians). In the sample 44% of the patients had smear positive TB and 54% of patients had a CD4 count of <100cells/mL. Genotypic distribution of *CYP2B6* was similar between the two countries with 50% having the CYP2B6*1/*1 genotype; however, there was a significantly larger proportion of CYP3A5*0/*0 among the Ethiopians compared to Tanzanians (61% vs. 28%).

In the sample 28% and 35% of patients had EFV levels <1000 ng/mL at weeks 4 and 16 respectively. In multivariate regression analysis, participants with CYP2B6*1/*6 and *6*6 allele had 0.50 (95% CI: 0.29 to 0.87) and 0.23 (95% CI: 0.05 to 1.03) times the risk of EFV concentrations <1000 ng/mL at 4 or 16 weeks as compared to those with *1/*1 respectively. In addition, 44% of patients with both CYP2B6*1/*1 and CYP3A5*0/*0 had EFV <1000 ng/mL which is a 2.2 higher risk compared to those who did not have both alleles.

**Conclusions:** *CYP2B6* and *CYP3A5* appear to be the primary determinants of plasma EFV concentrations among TB‐HIV patients. After adjustment for these alleles there was no difference in EFV concentration by country, suggesting other genetic differences may not significantly contribute to EFV metabolism. Individual‐level genotyping of *CYP* alleles is cost prohibitive in most treatment programs in Africa; however, provider knowledge of the population distribution of *CYP* alleles can help guide TB‐HIV treatment in low‐resourced African settings.

## WEPDB0203

### A randomized, open‐label, balanced, two‐treatment, single‐dose, crossover oral bioequivalence study of Lopinavir/Ritonavir Granules 40 mg/10 mg with KALETRA^®^ (Lopinavir/Ritonavir) Oral Solution 80 mg/20 mg per mL in normal healthy adults under fed conditions

**D. Abhijit^1^; C. Santanu^1^; D. Akhilesh^1^; K. Umakant^1^; R. Bangaru^2^; A.K. Datla^2^; P. Deshpande^3^; A. Kanda^3^ and E. Patras^3^**

^1^Mylan Laboratories Limited, R&D, Hyderabad, India, ^2^Mylan Laboratories Limited, Clinical Research Center, Hyderabad, India, ^3^Mylan Laboratiories Limited, Business Development, Hyderabad, India

**Background:** The development of pediatric fixed‐dose combinations (FDCs) for all lines of therapy has become a priority to simplify dosing, increase adherence and thus improve pediatric care. The use of LPV/r oral solution is limited by taste aversion and requires special storage conditions ‐refrigeration. Alternative palatable oral solid formulations as granules/pellets for small children are wanted. The objective of this study was to evaluate the relative oral bioavailability and safety profiles of Lopinavir/Ritonavir Granules 40 mg/10 mg (2 Sachets with 40/10 mg, Mylan Laboratories Limited, India) with KALETRA^®^ (Lopinavir/Ritonavir, AbbVie Inc.) Oral Solution 80 mg/20 mg/mL.

**Methods:** In this open label, 1:1 randomized, two‐period, two‐treatment, cross‐over, single dose evaluation, the relative oral bioequivalence was tested in 68 healthy adult subjects under fed conditions. In each study period, a single oral dose of either test product (T) or reference product (R) was administered orally under fed conditions. Subjects were monitored for safety and tolerability until completion of the study. Serial blood samples from pre‐dose 0.00 hour up to post‐dose 36.00 hours were collected in each period.

**Abstract WEPDB0203‐Table 1. Mean and SD of pharmacokinetic parameters estimated for test product and reference product**

**Table:** 

Parameters (units)	Geometric Least Squares Means Test product (T)	N	Geometric Least Squares Means Reference product (R)	N	T vs. R (90% CI)	T/R (%)	Intra Subject CV %	Power (%)
	Lopinavir							
Cmax (ng/mL)	389.809	68	369.967	68	94.15 to 117.91	105.36	40.9	94.7
AUC0‐t (ng·hour/mL)	3144.695	68	2955.001	68	94.48 to 119.86	106.42	43.4	92.6
AUC0‐inf (ng·hour/mL)	3215.917	67	3076.989	63	92.58 to 117.99	104.52	43.2	91.7
	Ritonavir							
Cmax (ng/mL)	27.859	68	25.242	68	100.91 to 120.72	110.37	32.1	99.2
AUC0‐t (ng·hour/mL)	219.562	68	201.972	68	98.53 to 119.94	108.71	35.4	98.1
AUC0‐inf (ng·hour/mL)	226.160	68	208.668	68	98.36 to 119.42	108.38	34.9	98.3

Geometric least squares mean, ratio of test product (T) and reference product (R), (T /R), 90% confidence intervals, Intra Subject Variability (CV in %) and power (in %) for the lntransformed pharmacokinetic parameters Cmax, AUC0‐t and AUC0‐inf for Lopinavir and Ritonavir

Drug concentrations in plasma were quantified by using a validated method for test product (T) and reference product (R). Pharmacokinetic parameters (Cmax, AUC0‐t, AUC0‐inf, Tmax, Kel, t½ and AUC_%Extrap_obs) were computed using the non‐compartmental model of Phoenix^®^ WinNonlin^®^ software version 6.3 (Pharsight Corporation, USA) for T and R. Statistical comparison of the pharmacokinetic parameters of both formulations were carried out by using PROC GLM from SAS^®^ statistical software version 9.2 to assess the bioequivalence of T and R.

**Results: ** The 90% confidence interval for the ratio of the test and reference product averages pharmacokinetic parameters Cmax, AUC0‐t and AUC0‐inf were between 80% and 125% for the ln‐transformed data with respect to Lopinavir and Ritonavir.

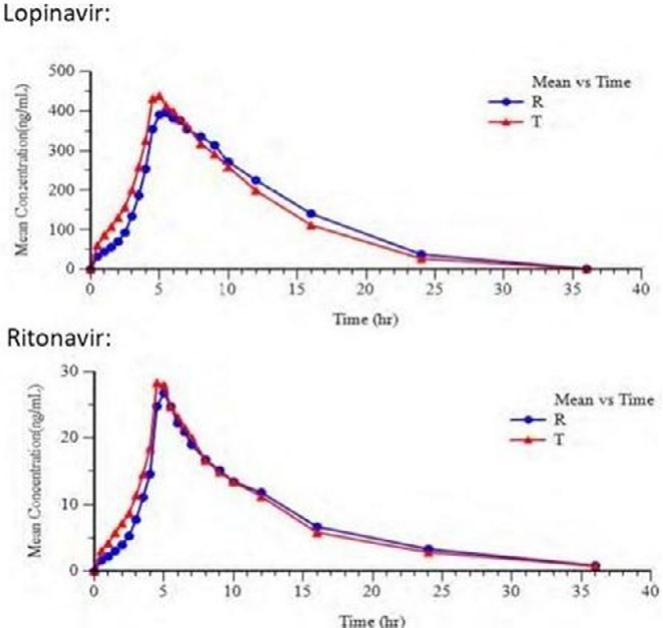

**Abstract WEPDB0203‐Figure 1. Linear plots of mean plasma concentration versus time curves of Lopinavir – Ritonavir after administration of test product (T) and reference product.**

Both the test and reference products were well tolerated, when administered as single dose under fed conditions.

**Conclusions: **Under fed conditions, the test product Lopinavir/Ritonavir Granules 40 mg/10 mg of Mylan Laboratories Limited, India was bioequivalent to the Reference product KALETRA^®^ (Lopinavir/Ritonavir) Oral Solution 80 mg/20 mg/mL of AbbVie Inc., USA, with regard to rate and extent of absorption. This new pediatric FDC could provide an easy‐to‐use treatment for small children.

## WEPDB0204

### Single‐dose fed bioequivalence study of Lamivudine, TenofovirDisoproxilFumarate and Dolutegravir tablets (300 mg/300 mg/50 mg) versus EPIVIR^®^ tablets (300 mg; ViiV‐Healthcare), VIREAD^®^ tablets (300 mg; Gilead Sciences) and TIVICAY^®^ tablets (50 mg; ViiV‐Healthcare) in healthy adult volunteers

**D. Abhijit^1^; C. Santanu^1^; R. Chetan^1^; G. Sachin^1^; R. Bangaru^2^; A.K. Datla^2^; P. Deshpande^3^; A. Kanda^3^ and E. Patras^3^**

^1^Mylan Laboratories Limited, R&D, Hyderabad, India, ^2^Mylan Laboratories Limited, Clinical Research Center, Hyderabad, India, ^3^Mylan Laboratiories Limited, Business Development, Hyderabad, India

**Background:** Dolutegravir (DTG) is one of the most frequently used recommended antiretrovirals for the treatment of HIV in high‐income countries. WHO and several national guidelines recommend DTG/3TC/TDF as an alternative first‐ and third‐line in low‐and middle‐income countries. The objective of this study was to compare the relative bioequivalence and safety profile of Mylan's lamivudine, tenofovirdisoproxilfumarate and dolutegravir tablets, 300 mg/300 mg/50 mg FDC tablets (T) with the reference combination (R) of EPIVIR^®^ (300 mg), VIREAD^®^ (300 mg) and TIVICAY^®^ (50 mg).

**Methods:** In this open label, randomized, two‐period, two‐treatment, cross‐over, single dose evaluation, the relative oral bioequivalence was tested in 33 healthy adult human subjects under fed conditions. In each period, each subject received a single, oral dose of T (Mylan's 3TC/TDF/DTG tablets) or R. Serial blood samples were collected pre‐dose and at 21 timepoints until 72 hours post dose. Subjects were monitored for safety and tolerability. Single‐dose pharmacokinetic parameters for FTC/TAF/DTG were calculated using non‐compartmental techniques.

**Results: ** All statistical analyses of these data reveal that the 90% confidence intervals are within the acceptable bioequivalent range of 80.00% and 125.00% for the natural log transformed parameters LNAUCL, LNAUCINF, and LNCPEAK for lamivudine, tenofovir and dolutegravir. (Table 1).

**Abstract WEPDB0204‐Table 1. Pharmacokinetic Results 3TC, TNF and DTG**

**Table:** 

Product	Parameter	Arithmetic Mean (%CV) T = Mylan	Arithmetic Mean (%CV) R = Reference	LSMEANS Ratio (T/R)	90% Confidence Interval
3TC	AUCL (ng·hour/mL)	11648 (18.64)	11911 (18.84)	0.98	96.41% to 100.29%
3TC	AUCINF (ng·hour/mL)	12062 (19.04)	12206 (18.26)	0.99	96.94% to 101.15%
3TC	CPEAK (ng/mL)	1960 (27.94)	2064 (24.37)	0.94	88.17% to 100.59%
TNF	AUCL (ng·hour/mL)	3124 (19.55)	3107 (19.45)	1.01	98.10% to 103.80%
TNF	AUCINF (ng·hour/mL)	3311 (19.74)	3269 (19.85)	1.01	98.69% to 104.32%
TNF	CPEAK (ng/mL)	318.7 (24.99)	278.9 (20.60)	1.11	104.08% to 119.12%
DTG	AUCL (ng·hour/mL)	71099 (24.79)	70780 (26.25)	1.00	95.64% to 104.78%
DTG	AUCINF (ng·hour/mL)	74636 (25.55)	73486 (26.72)	1.01	96.51% to 105.45%
DTG	CPEAK (ng/mL)	4025 (18.75	3840 (18.14)	1.03	99.05% to 107.71%

The AEs were mild in severity. Overall both R and T were well tolerated administered as a single, oral dose under fed conditions.

**Conclusions: **This study demonstrates that Mylan's 3TC/TDF/DTG 300 mg/300 mg/50 mg tablets are bioequivalent to a combination of EPIVIR^®^ (300 mg), VIREAD^®^ (300 mg) and TIVICAY^®^ (50 mg) as separate tablets following administration of a single, oral dose administered under fed conditions.

## WEPDB0205

### Population pharmacokinetics of cabotegravir in adult healthy subjects and HIV‐1 infected patients following administration of oral tablet and long acting intramuscular injection

**K. Han^1^; P. Patel^2^; M. Baker^3^; D. Margolis^2^; W. Spreen^2^ and S. Ford^4^**

^1^ViiV Healthcare, Upper Merion, United States, ^2^ViiV Healthcare, Research Triangle Park, United States, ^3^ViiV Healthcare, Münchenbuchsee, Switzerland, ^4^GlaxoSmithKline, Research Triangle Park, United States

**Background:** Cabotegravir is an integrase inhibitor currently in Phase 3 development as an oral tablet and an intramuscular long‐acting injection (LAIIM) for HIV treatment and prevention. The aim was to characterize cabotegravir population pharmacokinetics (PopPK) using data from Phase 1 and 2 studies, evaluate the association of intrinsic and extrinsic factors with the variability of cabotegravir PopPK, and perform simulations to inform dosing strategies.

**Methods:** All analyses were implemented in NONMEM 7.3 and R. Covariate relationships were evaluated using a forward addition (*p* < 0.01) and backward elimination (*p* < 0.001) approach. Model adequacy and predictive performance was assessed using bootstrapping and visual predictive check. Clinical relevance of covariates was assessed using tornado plots. Simulations were performed using parameter estimates from the final model.

**Results: ** A total of 12,294 cabotegravir plasma concentrations were collected from 881 healthy (44%) and HIV infected (56%) adults in 11 studies at 9 dose levels (5 mg to 60 mg for oral tablet; 100 mg to 800 mg for LAIIM). LAIIM was administered in 64% of the subjects. A two‐compartment model with first‐order oral and intramuscular absorption and elimination including inter‐occasion variability adequately described the data. Clearances and volumes were scaled to body size. Relative bioavailability of the oral to LAIIM formulation was >70%. Residual variability was higher following LAIIM administration. Race and age were not significant covariates. LAIIM absorption rate constant was lower in females, in subjects with higher BMI and if the LAIIM dose was given as one single injection instead of two “split” injections. However, gender, BMI or split dose alone was associated with less than 15% of change in exposure at steady‐state following oral or LAIIM administration. Simulations supported dosing strategies for ongoing Phase 3 studies.

**Conclusions: **A robust PopPK model for cabotegravir was developed and used for simulations to support dosing recommendations for HIV treatment. Gender, BMI, and split LAIIM dosing were statistically significant covariates on cabotegravir LAIIM absorption, but the magnitude of their impact on cabotegravir exposures was not clinically relevant. No dose adjustment of cabotegravir is recommended based on gender, BMI or body size, split LAIIM dosing, race, or adult age group.

## WEPDC0102

### Implementation and impact of a technology‐based HIV risk‐reduction intervention among Thai men who have sex with men using “Vialogues:” A randomized controlled trial

**T. Anand^1^; C. Nitpolprasert^1^; J. Jantarapakde^2^; R. Meksena^2^; S. Phomthong^2^; P. Phoseeta^2^; P. Phanuphak^2^ and N. Phanuphak^2^**

^1^Adam's Love, Bangkok, Thailand, ^2^PREVENTION, The Thai Red Cross AIDS Research Centre, Bangkok, Thailand

**Background:** To conduct a randomized control trial to evaluate the impact of a novel technology‐based intervention on HIV/AIDS knowledge, self‐perceived risk, condom use attitudes, self‐efficacy intentions, sexual risks and condom use behaviors among Thai MSM.

**Methods:** The technology‐based HIV risk reduction intervention was piloted by Adam's Love (www.adamslove.org). Participants aged 18 years or above, and having engaged in unprotected sex in the past six months were enrolled and randomly assigned to control or intervention arm, and received private clinic‐based HIV counseling and testing at baseline, month 6 and month 12. Intervention arm participants engaged in 12 monthly HIV/STI prevention and educational sessions delivered via Vialogues.com (using online time‐based videos followed by time‐stamped discussions around the video content with a health educator, focusing on applying knowledge and skills learned during the session in setting long‐term goals to reduce sexual risks). The differences in behavioral outcomes between the arms over the 12‐month period were assessed.

**Results: ** Of 76 MSM enrolled, 37 were randomized to intervention and 39 to control arm. Median age was 28 (IQR 24 to 32) years. Of 37 intervention arm participants, 33 (89.2%) completed all 12 monthly Vialogues sessions. Mean session duration was 37.45 minutes. Retention rates at month 12 among intervention and control arms were 97.3% and 79.5%, respectively. At month 12, median percentage of condom use for anal intercourse was higher in the intervention versus control arm (100% vs. 93.3%, *p* = 0.023). Over the 12‐month period, intervention arm reported significant reduction in self‐perceived risk for HIV (3.08 on 5‐point LIKERT scale to 2.6, *p* = 0.001), popper usage (29.7% to 13.9%, *p* = 0.002), seeking sex online (59.5% to 44.4%, *p* = 0.01), median number of sexual partners in the past three months (2 to 1, *p* = 0.003), and increased median percentage of condom use (88.9% to 100%, *p* = 0.006). Process measures yielded high participant satisfaction of Vialogues (mean 4.67 on a 5‐point scale, SD 0.48).

**Conclusions: **Our study provides clear evidence that “Vialogues” intervention significantly reduced number of sexual partners and increased condom use rates among MSM. HIV program implementers are encouraged to harness the potential of free, online learning technologies used in this study.

## WEPDC0103

### P3 (Prepared, Protected, emPowered): feasibility and acceptability of a PrEP adherence app featuring peer‐to‐peer sharing, game‐based elements and in‐app adherence counseling

**S. Legrand^1^; K. Knudtson^2^; K. Muessig^3^; M. Dixon^4^; A. Mcgee^2^; J. Dormitzer^5^; R. Jackson^6^; E. Adam^7^ and L. Hightow‐Weidman^2^**

^1^Duke University, Duke Global Health Institute, Durham, United States, ^2^University of North Carolina‐Chapel Hill, Medicine/Infectious Diseases, Chapel Hill, United States, ^3^University of North Carolina‐Chapel Hill, Health Behavior, Chapel Hill, United States, ^4^Ayogo, Vancouver, Canada, ^5^Fenway Health, Boston, United States, ^6^Cook County Hospital, Chicago, United States, ^7^Emory University, Atlanta, United States

**Background:** To date, efficacious interventions to promote and sustain pre‐exposure prophylaxis (PrEP) adherence among youth are limited. Smartphone apps provide a platform to address adherence barriers by delivering tailored strategies and culturally‐relevant content in an engaging format. App‐based interventions that also allow for intensified interactions through direct communication with adherence counselors may improve outcomes among nonresponsive youth. We describe the development and initial testing of a PrEP adherence app for young men who have sex with men (YMSM) and young transgender women who have sex with men (YTWSM) that includes provision of adherence counseling delivered within the app through a provider texting interface.

**Methods:** P3 (Prepared, Protected, emPowered) is an app built upon an established theory‐based, health platform, optimized through our teams’ prior work with HIV+ YMSM and with youth advisory board (YAB) members at 7 Adolescent Trials Network for HIV Interventions (ATN) sites. P3 includes interactive components to encourage peer‐to‐peer sharing and the development of daily medication self‐monitoring behaviors. Game‐based elements, including virtual and real‐world rewards, based on established behavioral economics principles, incentivize engagement. Strengths‐based adherence counseling delivered by a centrally‐located adherence counselor via two‐way text messaging through the app addresses individuals’ unique barriers to PrEP adherence.

**Results: ** Usability testing was conducted with 12 YMSM/YTWSM (mean age 20.9, 10 male), either PrEP experienced or considering, at 2 ATN sites using a clickable app prototype (screenshots presented in Figure 1). Most (92%) found the app highly acceptable, particularly the tailored medication reminders, the daily medication tracking with corresponding medication history calendar, and the accessibility, comprehensiveness and tone of the information provided. Youth were enthusiastic about connecting directly with an adherence counselor and provided concrete recommendations for rapport building, session duration and explicitly defining the counselors’ role.

**Conclusions: **P3 represents, to our knowledge, the first theoretically‐based PrEP adherence app for YMSM/YTWSM that includes features to increase intervention engagement (e.g. gamification, financial incentives, social connectivity) and provides in‐app adherence counseling. Interventions that capitalize on technology‐based platforms have great potential to encourage health promotion behaviors. A technical pilot of P3 will be conducted in Summer 2018 followed by an efficacy trial if feasibility/acceptability is achieved.

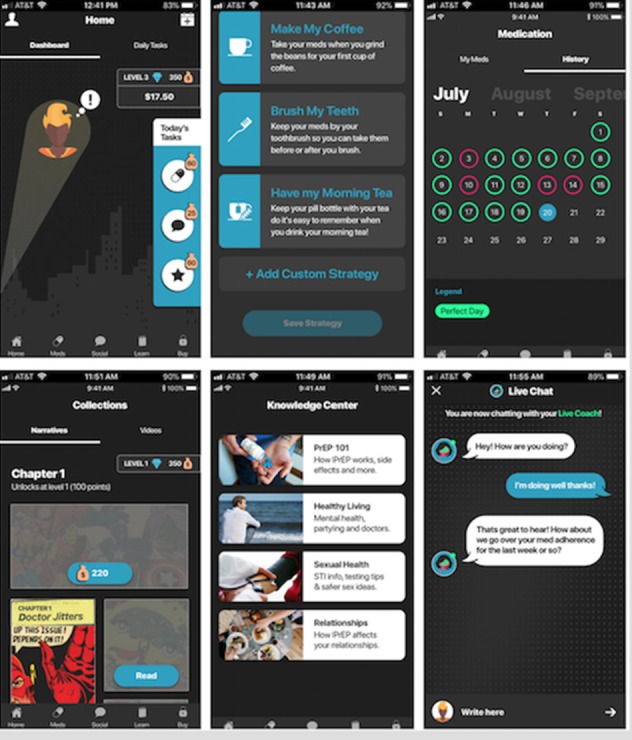

**Abstract WEPDC0103 Figure 1. Screen Shots of the P3 app.**

## WEPDC0104

### Effectiveness of community‐led sex‐positive campaign on HIV testing for young men who have sex with men (YMSM) in Metro Manila (MM), Philippines: TestMNL

**L.‐A.F.M. Lomarda^1,2^; R.G. Pagtakhan^1^ and P.J. Tanpoco^1^**

^1^LoveYourself Inc., Mandaluyong City, Philippines, ^2^APCOM, Bangkok City, Thailand

**Background:** In a predominantly Catholic country like the Philippines, HIV prevention campaigns use stigma and fear to educate the public about HIV. There is an absence of sex‐positive HIV prevention efforts in the Philippines that use internet and social media to engage YMSM. This campaign is an attempt to leverage on online platforms to drive sex‐positive HIV prevention efforts while engaging YMSM. Due to the concentration of HIV infections in MM, this campaign is focused on young gay men (18 to 24 years old) who regularly seek out male sexual partners online.

**Description:** In 2015, TestBKK has been a successful campaign in Thailand to encourage YMSM to get tested for HIV. LoveYourself, a community‐based LGBT organization, partnered with APCOM to test the effectiveness of this sex‐positive HIV prevention model in the Philippines. After a series of focus group discussions, LoveYourself and APCOM adapted TestBKK's simple, clear, and easy‐to‐remember slogan to encourage YMSM to get tested for HIV: “SUCK. F*#K. TEST. REPEAT”. The campaign is promoted in campaign websites, social media, gay networking apps, and offline events.

The campaign reached 46,910 individuals to learn about HIV testing in MM via http://www.TestMNL.org;; 18,369 individuals were tested for HIV (38.25% are YMSM); and 1343 turned positive (7.31%). On social media, TestMNL created 79,934 +  views on campaign videos on Facebook and YouTube, 7642 Facebook likes, and 3155 Twitter followers.

**Lessons learned:** Partner clinics of TestMNL reported an average increase in HIV testing of 62.05% compared to previous months the year before. Meetings with community groups, clinical partners, private partners and YMSM also reported the effectiveness of the campaign in their engagements on the importance of sexual health promotion, creating a shift to a more sex‐positive environment in the gay community in MM. Formative assessment is essential to determine the type, tone, theme, and approach of campaign that will work.

**Conclusions/Next steps:** The campaign concludes the need for more community‐led sex‐positive campaigns that will educate YMSM about their sexual health, and encourage them to get tested for HIV. Scale up of the program is needed to provide more opportunity to develop new campaign messages that address key barriers to HIV continuum of care.

## WEPDC0105

### Internet‐based self‐testing model – “Easy Test”: A cross‐sectional survey targeting MSM who never tested before in 14 provinces of China

**X. Jin^1^; D. Xiao^2^; X. Xiu^1^; Z. Ding^1^; Y. Zhang^1^; Y. Jie^1^; Y. Liao^1^; H. Wu^3^; N. Cao^4^; B. Shepherd^5^; J. Vandenhombergh^5^ and Y. Bao^1^**

^1^AIDS Healthcare Foundation (AHF), AHF China Office, Beijing, China, ^2^Tongzhi Welfare, Beijing, China, ^3^Beijing Youan Hospital, Beijing, China, ^4^Hospital of Dermatology, Chinese Academy of Medical Sciences & Peking Union Medical College, Nanjing, China, ^5^AIDS Healthcare Foundation (AHF), Los Angeles, United States

**Background:** HIV prevalence among men who have sex with men (MSM)increased rapidly from 2006 to 2016 in China. While about half of MSM have never been tested HIV, even though facility‐based HIV testing sites had been greatly scaled up by public health services. With the country's explosive internet growth, MSM often socialize and search for casual sexual partners through online activity, cellular phone applications and social media. To reach an MSM community whose members rarely access traditional, offline testing facilities, innovative and convenient HIV testing models are urgently required

**Methods:** An Internet‐based Self‐testing Model (“Easy Test”) was developed by AIDS Healthcare Foundation (AHF) to provide free online applications with instructed testing from October to December 2017 in 14 Chinese Provinces. Clients were required to complete a questionnaire and pay a $5 deposit when applying for a blood‐based HIV test. Upon uploading an image of the client's test result to the online applications, the deposit would be refunded. For clients with an HIV‐positive result, a one‐on‐one accompanied referral was provided for further status confirmation and medical services.

**Results: ** A total of 879 MSM applied a self‐testing test kit and completed the questionnaire. Of the total tested, 78% (683/879) of clients provided feedback of their test results, of which 52% (352/683) had never before been tested for HIV. HIV prevalence was 14% (98/683) with 72% (71/98) of those found positive linked and enrolled in treatment. For all testers, the median age was 28 years (IQR, 24 to 34 years); Han ethnicity (92%); single (74%); college‐educated and above (69%); monthly income between $450‐$750 US dollars (51%). Of those never tested before, 37% (108/295) reported seldom or never using condoms during anal sex in the past three months. Reported condom use during most recent anal intercourse was 76% (266/352), and of those, 48% (128/266) failed to consistently use condoms during the entire process of anal intercourse.

**Conclusions: **The online “Easy Test” model is an innovative, effective remedial measure for high‐risk MSM who are reluctant to be HIV tested at stationary facilities. It effectively increases access to HIV treatment services for HIV‐positive MSM in China.

## WEPDC0106

### From online reach to offline services: using social media strategies to increase uptake of and access to HIV testing among MSM in Vietnam

**T.T. Tran^1^; K. Green^1^; V.D. Nguyen^2^; N.B. Vu^1^; T.T. Nguyen^3^; T.T. Doan^4^; M.T. Le^5^ and M.T. Ngo^6^**

^1^PATH, Ha Noi, Vietnam, ^2^PATH, Ho Chi Minh City, Vietnam, ^3^KOL, Ho Chi Minh City, Vietnam, ^4^Lighthouse, Ha Noi, Vietnam, ^5^G‐Link, Ho Chi Minh City, Vietnam, ^6^USAID, Ha Noi, Vietnam

**Background:** Rates of HIV are increasing among the estimated 330,000 men who have sex with men (MSM) in Vietnam. Face‐to‐face outreach only reaches a fraction of at‐risk MSM. A 2015 USAID/PATH Healthy Markets (HM) study found that 98% of MSM surveyed across four provinces regularly used Facebook, and preferred social media as a source of HIV and health information. HM and MSM leaders co‐created a fun, sex‐positive HIV prevention and service awareness, trust, and uptake campaign—”My Future, My Choice”—that utilized a Facebook community (Rainbow Village), online influencers trained as HIV lay testers, and an HIV service booking application (I Reserve), to allow for a measurable online‐to‐offline HIV testing‐treatment cascade.

**Methods:** Three techniques were applied to characterize the online‐to‐offline cascade in Ho Chi Minh City (HCMC) and Hanoi from 2016 to 2017 (18 months):

(1) an online Facebook user survey;

(2) a rapid survey assessing MSM self‐reported motivation for HIV testing when they presented for an HIV test;

(3) results from online peer influencers and I Reserve app, applying an HIV testing‐treatment cascade analysis.

**Results: ** By December 2017, Rainbow Village had over 232,000 members; 88% were aged 25 or over and 70% were HCMC/Hanoi residents. The Facebook user study (n = 424) found 50% of respondents visited Rainbow Village at least once a week, 75% were not in contact with HIV outreach workers/peers, and 38% self‐assessed at substantial HIV risk. The rapid survey on primary HIV testing motivators (n = 3989) found that 35% stated online content as the reason for testing, 8% of whom tested HIV‐positive—compared to 5% of those motivated by face‐to‐face peer interactions/referrals. Among 2454 people reached by online peer influencers or the I Reserve app, 73% tested, 11% of whom were HIV‐diagnosed and enrolled in treatment (100%). This compares to an overall 6% HIV positivity yield among MSM seeking HIV lay or self‐testing.

**Conclusions: **Online interventions effectively reach MSM who may never be contacted through conventional face‐to‐face peer outreach, and are more effective in reaching higher risk MSM. Online strategies should be further scaled and adapted alongside face‐to‐face interventions to reach more diverse segments of at‐risk MSM.

## WEPDC0107

### Online supervised HIV self‐testing identified high HIV yield among Thai men who have sex with men and transgender women

**J. Jantarapakde^1^; K. Himmad^1^; T. Sungsing^1^; T. Anand^1^; C. Nitpolprasert^1^; S. Promthong^1^; W. Waiwinya^1^; P. Meekrua^2^; S. Sukthongsa^2^; S. Hongwiangchan^3^; N. Upanun^4^; D. Trachunthong^1^; S. Barisi^1^; S. Jirajariyavej^5^; S. Charoenying^6^; S. Mills^6^; R. Vannakit^7^; M. Cowing^8^; S. Buchbinder^8^ and N. Phanuphak^1^**

^1^The Thai Red Cross AIDS Research Centre, Prevention, Bangkok, Thailand, ^2^Service Workers in Group Foundation, Bangkok, Thailand, ^3^Rainbow Sky Association of Thailand, Bangkok, Thailand, ^4^Sisters Foundation, Chonburi, Thailand, ^5^Taksin Hospital, Bangkok, Thailand, ^6^FHI 360 and USAID LINKAGES Project, Bangkok, Thailand, ^7^Office of Public Health, U.S. Agency for International Development Regional Development Mission Asia, Bangkok, Thailand, ^8^The Foundation for AIDS Research (amfAR), New York, United States

**Background:** Online technology has high potential to enhance access to HIV counseling and testing among members of key populations whose HIV status is undiagnosed. We evaluated HIV‐positive/reactive rates and confirmed successful linkage to antiretroviral treatment (ART) among MSM and TGW in an implementation research study (“Online Supervised HIV Self‐Testing”) in Thailand.

**Methods:** During December 2015‐June 2017, MSM and TGW self‐selected to enroll into one of three groups:

(1) clinic‐based HIV testing and counseling (Offline group);

(2) online pre‐test counseling and clinic‐based HIV testing (Mixed group); and

(3) online pre‐test counseling and supervised HIV self‐testing (Online group).

Linkage to ART was provided immediately after HIV‐positive/reactive test results were known. Online retention and support were provided to the Mixed and Online groups only. Sociodemographic data, beliefs and experiences around stigma and discrimination related to HIV and/or being MSM or TGW, sexual and drug use behaviors, perceived barriers and facilitators for access to HIV testing were collected by self‐administered questionnaires. Factors associated with unsuccessful linkage to ART were identified using binary logistic regression method.

**Results: ** Of 564 participants (465 MSM and 99 TGW), 200 selected the Offline group, 156 selected the Mixed group, and 208 chose the Online group. Mean (±SD) age was 27.9 (7.2) years. Baseline HIV‐positive/reactive result was higher in the Offline (13.0%) and Online (14.4%) groups, compared to the Mixed group (3.2%), *p* = 0.001. Linkage to ART, however, was least successful in the Online group (52.8%), compared to the Offline (83.9%) and Mixed (75.0%) groups, *p* = 0.02. Being in the Online group (aOR = 8.54, 95% CI 1.08 to 67.59, *p* = 0.04), aged <17 years at first sex (aOR = 13.16, 95% CI 1.62 to 107.08, *p* = 0.02), and having single partner (aOR 12.61, 95% CI 1.52 to 104.9, *p* = 0.02) increased risk for unsuccessful linkage to ART. Stigma and discrimination experiences did not reduce the chance of successful ART linkage.

**Conclusions: **Offering online supervised HIV self‐testing successfully engaged MSM and TGW with high HIV‐reactive yield into HIV testing service. Linking clients tested HIV‐reactive online to come out for offline HIV confirmation and ART initiation proved to be a real challenge. Innovative methods to support transition of these clients from online to offline services are urgently needed.

## WEPDC0201

### Syndemics predict bio‐behavioral HIV sexual transmission risk (TRB) longitudinally in US HIV clinics

**S. Satyanarayana^1^; S.A. Safren^1^; B.G. Rogers^1^; S.A. Bainter^1^; K.A. Christopoulos^2^; R.J. Fredericksen^3^; W.C. Mathews^4^; R.D. Moore^5^; M.J. Mugavero^6^; S. Napravnik^7^; M.J. Mimiaga^8^; K.H. Mayer^9^ and H.M. Crane^3^**

^1^University of Miami, Psychology, Coral Gables, United States, ^2^University of California, San Francisco, United States, ^3^University of Washington, Seattle, United States, ^4^University of California, San Diego, United States, ^5^Johns Hopkins University, Baltimore, United States, ^6^University of Alabama at Birmingham, Birmingham, United States, ^7^University of North Carolina at Chapel Hill, Chapel Hill, United States, ^8^Brown University, Providence, United States, ^9^Harvard University, Boston, United States

**Background:** Syndemic conditions are co‐occurring psychosocial conditions that interact synergistically to exacerbate the risk for HIV transmission. Little is known about the role syndemics play in HIV transmission risk in resource‐rich clinic settings. While antiretroviral therapy (ART) is key to treatment‐as‐prevention, to attain the 90‐90‐90 goals for HIV control, HIV care clinics will likely need to identify individuals most in need of psychosocial interventions. Syndemics could potentially aid providers in identifying these individuals.

**Methods:** Data were obtained from 15,739 HIV‐positive individuals receiving care through the Centers for AIDS Research Network of Integrated Clinical Systems (CNICS) at seven US sites between July 2000 and April 2017. Syndemic conditions (substance abuse, at‐risk drinking, depression, anxiety) and sexual risk behaviors were collected using validated instruments through patient‐reported outcomes completed at clinical visits at least six months apart. Because sexual orientation data were only available for some of the sample, sexual risk groups were classified iteratively by examining self‐identified sexual orientation and then sexual behaviors depending on sex of partner(s) and sex acts. Using multilevel modeling, we modeled time, number of syndemic conditions, and HIV risk group as predictors of bio‐behavioral transmission‐risk behavior (TRB; condomless anal or vaginal sex while virally detectable (HIV RNA>400)) with a partner of negative or unknown status.

**Results: ** Between‐person and within‐person effects of syndemics on TRB emerged. Patients who, on average, had more syndemics had greater odds of engaging in TRB, such that each syndemic condition endorsed increased the odds by 1.61 (OR = 1.61; 95% CI = 1.51, 1.72). Additionally, within individuals, each additional syndemic condition endorsed at any given time point resulted in 1.75 odds greater of engaging in TRB (OR = 1.75; 95% CI = 1.52, 2.02). Compared to females (referent), heterosexual males had 41% lower odds of engaging in TRB (OR = 0.59; 95% CI = 0.43,0.80). There were no other risk‐group differences.

**Conclusions: **Across risk groups, identifying syndemics via patient‐report‐outcome measures in HIV clinics is feasible. Integrating the treatment of syndemic conditions with approaches to promote sexual and behavioral health in HIV clinic settings carries potential for reducing the number of new infections via TRB.

**Abstract WEPDC0201‐Table 1. Summary of Demographics**

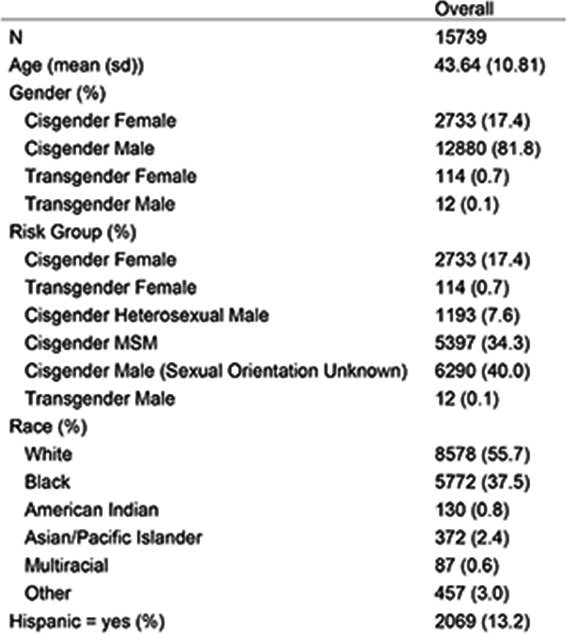

## WEPDC0202

### Correlates of benzodiazepine and opioid co‐prescription among people living with HIV in British Columbia, Canada

**S. Parent^1^; S. Nolan^2,3^; N. Fairbairn^2,3^; M. Ye^1^; A. Wu^4^; J. Montaner^1,3^; R. Barrios^1,5^; L. Ti^1,3^ and STOP HIV/AIDS Study Group**

^1^British Columbia Centre for Excellence in HIV/AIDS, Vancouver, Canada, ^2^British Columbia Centre for Substance Use, Vancouver, Canada, ^3^University of British Columbia, Department of Medicine, Vancouver, Canada, ^4^British Columbia Centre for Excellence in HIV/AIDS, Canada, Canada, ^5^Vancouver Coastal Health, Vancouver, Canada

**Background:** The co‐prescription of opioids and benzodiazepines is relatively contraindicated due to possible overdose risk. However, people living with HIV (PLWH) may have concurrent psychiatric or chronic pain diagnoses that require the use of either opioids or benzodiazepines for symptomatic treatment. Consequently, some PLWH may be at‐risk for the health harms associated with the co‐prescribing of these medications. Given this, the objective of this study was to characterize patient factors associated with the co‐prescribing of opioids and benzodiazepines among PLWH in British Columbia (BC), Canada.

**Methods:** Using data derived from the Seek and Treat for Optimal Prevention HIV/AIDS in BC cohort, we used bivariable and multivariable generalized estimating equation models to establish the prevalence of a concurrent opioid and benzodiazepine co‐prescription and determine factors associated with this practice.

**Results: ** Between April 1996 and February 2015, a total of 14,484 PLWH were included in the study. A total of 3835 (26.5%) participants were prescribed both medications at least once during the study period. At baseline, 45.5% were prescribed opioids only, 19.9% were prescribed benzodiazepines only, and 30.8% were prescribed neither medication. A concurrent opioid and benzodiazepine prescription was independently and positively associated with depression/mood disorder (adjusted odds ratio (AOR) = 1.32, 95% confidence interval (CI): 1.22 to 1.43) and anxiety disorder (AOR = 1.45, 95% CI: 1.27 to 1.66), whereas female sex (AOR = 0.76, 95% CI: 0.64 to 0.91) and substance use disorder (SUD) (AOR = 0.82, 95% CI: 0.74 to 0.90) were negatively associated with the outcome.

**Abstract WEPDC0202‐Table 1. Bivariable and multivariable generalized estimating equation analyses of factors associated with opioids and benzodiazepiness co‐prescription**

**Table:** 

Characteristic	Unadjusted odds ratio (95% confidence interval)	Adjusted odds ratio (95% confidence interval)
Sex (male vs. female)	0.78 (0.67 to 0.90)	0.76 (0.64 to 0.91)
Age at baseline (10 years)	1.16 (1.11 to 1.22)	1.11 (1.04 to 1.18)
Calendar year (10 years)	0.73 (0.68 to 0.79)	0.65 (0.59 to 0.72)
Depression/mood disorder (no vs. yes)	1.47 (1.37 to 1.57)	1.32 (1.22 to 1.43)
Anxiety (no vs. yes)	1.48 (1.33 to 1.66)	1.45 (1.27 to 1.66)
Substance use disorder (no vs. yes)	0.95 (0.88 to 1.03)	0.82 (0.74 to 0.90)
Charlson comorbidity index	1.08 (1.06 to 1.10)	1.09 (1.07 to 1.11)
CD4 cell count (100 cells/mm^3^)	1.00 (0.98 to 1.03)	1.02 (1.00 to 1.05)
Viral load (log10 copies/mL)	1.04 (1.02 to 1.07)	1.03 (1.00 to 1.07)

**Conclusions: **Our findings indicate that co‐prescription of opioids and benzodiazepines was seen in a high proportion of patients. Concurrent prescription was positively associated with anxiety and depression/mood disorder, but negatively associated with being female and presence of a SUD. These findings hint towards groups to target for prevention of harms stemming from co‐prescription. However, given the risks associated with co‐prescribing and the common comorbidities among PLHIV where these medications may be indicated, careful consideration should be taken prior to co‐prescribing. Future research should seek to further explore co‐prescription practices in order to determine their appropriateness in these circumstances.

## WEPDC0203

### When sex, drugs and violence overlap: assessing the syndemic and synergistic effects of intimate partner violence, crystal methamphetamine, and depression on HIV sexual risk among women who inject drugs

**C. Stoicescu^1^; L.D. Cluver^1^ and R. Ameilia^2^**

^1^University of Oxford, Social Policy & Intervention, Oxford, United Kingdom, ^2^University of Indonesia, Department of Criminology, Depok, Indonesia

**Background:** Women who inject drugs are disproportionately affected by concomitant intimate partner violence (IPV), depression and substance use. Little is known about the synergistic effects of these conditions on women's HIV risk in low‐ and middle‐income countries. The Perempuan Bersuara study assessed additive and interactive effects of syndemic health conditions on HIV risk in Indonesia's largest sample of drug‐using women.

**Methods:** 731 women aged ≥18 years and injecting drugs in the preceding year were recruited from Jakarta and Bandung using respondent‐driven sampling. Logistic regressions and marginal effects models tested associations and predicted probabilities of exposure to depression, IPV and crystal meth on three sexual risk outcomes. Additive interaction was assessed using relative excess risk due to interaction (RERI), attributable proportion due to interaction (AP) and synergy index (S).

**Results: ** Prevalence of concurrent exposure to IPV, crystal meth and depression was 26%. Relative to the absence of these conditions, simultaneous exposure to all 3 increased rates of HIV risk: STI symptomatology (from 12% to 60%), inconsistent condom use (from 3% to 22%), and survival sex (from 6% to 25%). Additive interaction was detected between: (a) IPV x crystal meth on inconsistent condom use (AP = 0.38, *p* < 0.05), such that 38% of inconsistent condom use among women reporting IPV and crystal meth was attributable to the interaction between these exposures; (b) depression x crystal meth on STI symptomatology (RERI = 2.04, *p* < 0.001; AP = 0.61, *p* < 0.001) and survival sex (RERI = 1.20, *p* < 0.01; AP = 0.53, *p* < 0.01), meaning that 61% of STI symptoms and 53% of survival sex participation among women reporting depression and crystal meth use was attributable to interaction between those exposures; and IPV x depression on STI symptomatology (RERI = 3.01, *p* < 0.01; AP = 0.52, *p* < 0.001; S = 2.70, *p* < 0.01) and survival sex (RERI = 1.21, *p* < 0.05; AP = 0.40, *p* < 0.05), suggesting the joint effect of IPV and depression resulted in a three‐fold increase in STI symptoms and 1.2x increase in survival sex compared to each exposure's main effect.

**Conclusions: **This study provides new empirical evidence showing the syndemics of IPV, depression and crystal meth interact synergistically to heighten HIV risk among women who inject drugs. Interventions that consider the full scope of syndemic vulnerabilities, rather than addressing individual afflictions separately, are essential.

## WEPDC0204

### Combination prevention for women who use alcohol in South Africa: outcomes from the Women's Health CoOp Plus Study in Pretoria, South Africa

**W. Wechsberg^1^; W. Zule^1^; C. van derHorst^2^; C. Peasant Bonner^1^; J. Ndirangu^3^; F. Browne^1^; T. Kline^4^; B. Howard^1^ and N. Rodman^5^**

^1^RTI International, Substance Use, Gender & Applied Research, Durham, United States, ^2^UNC School of Medicine, Chapel Hill, United States, ^3^RTI International, Substance Use, Gender & Applied Research, Washington, United States, ^4^RTI International, Statistics and Epidemiology, Durham, United States, ^5^RTI International, Research Computing Division, Durham, United States

**Background:** Women who use substances, are at heightened risk for HIV but have been largely ignored by HIV primary and secondary prevention efforts. They are also more likely to experience gender‐based violence (GBV) and sexual risk, with many reporting sex work – increasing their HIV risk and decreasing their access to HIV care and antiretroviral (ARV) adherence. The purpose of this study was to determine the efficacy of voluntary HIV counseling and testing (HCT) compared to the Women's Health CoOp (WHC+; 2 sessions) a gender focused prevention package to reduce substance use, GBV, sexual risk, and increase linkage to HIV care and adherence for women; hypothesizing that the WHC+ will report less substance use, GBV, sexual risk and more ARV adherence.

**Methods:** This NIH cluster randomized trial recruited 641 Black African women (Mean age = 29.9 SD = 0.31 from 2013 to 2016) across the 14 geographic zones in Pretoria. Women completed interviews, drug, alcohol, pregnancy and HIV screening at baseline, 6‐ and 12‐months in both arms with over 90% follow‐up. CD4 tests were added to assist clinical staging in linkage to local clinics. Dried blood spots were obtained for viral load (VL) testing from a subset of women who were HIV positive.

**Results: ** Over 90% of women completed the study. Multiple and logistic regression using robust standard errors to account for clustering at the zone level and controlling for sex work and HIV status revealed that women in the WHC+ arm were less likely to report frequent heavy drinking (*p* < 0.001); physical beating by boyfriend (*p* < 0.001); and reported more protected condom use (*p* < 0.03) with main partner at 6‐month follow‐up compared to those in the HCT arm. Of those linked to HIV treatment, 81% of participants in the WHC+ arm reported adhering to their ARVs compared to 65% of participants in the HCT arm (*p* = 0.07). There was a relationship between reduced heaving drinking and ARV adherence.

**Conclusions: **The WHC+ combination prevention was found efficacious reducing intersecting risks, linking women who tested HIV+ to care and helping with adherence. It could have further impact with booster sessions after six months with larger implementation.

## WEPDC0205

### Behavioral activation integrated with sexual risk reduction counseling for high‐risk MSM with crystal methamphetamine dependence: an initial randomized controlled trial

**M. Mimiaga^1,2,3^; D. Pantalone^3,4^; K. Biello^3,5^; J. White Hughto^3,6^; J. Frank^2,5,7^; C. O'Cleirigh^3,8^; S. Reisner^9,10,11^; A. Restar^5,7^; C. Santostefano^7^; K. Mayer^3,12^ and S. Safren^3,13^**

^1^Brown University, Providence, United States, ^2^Brown University School of Public Health, Department of Psychiatry and Human Behavior, Providence, United States, ^3^The Fenway Institute, Boston, United States, ^4^University of Massachusetts, Department of Psychology, Boston, United States, ^5^Brown University School of Public Health, Department of Behavioral & Social Health Sciences and Epidemiology, Providence, United States, ^6^Yale University School of Public Health, Department of Epidemiology, New Haven, United States, ^7^Brown University, Center for Health Equity Research, Providence, United States, ^8^Harvard Medical School/Massachusetts General Hospital, Department of Psychiatry, Behavioral Medicine Service, Boston, United States, ^9^Boston Children's Hospital, Division of General Pediatrics, Boston, United States, ^10^Harvard Medical School, Department of Pediatrics, Boston, United States, ^11^Harvard T.H. Chan School of Public Health, Department of Epidemiology, Boston, United States, ^12^Harvard Medical School/Beth Israel Deaconess Medical Center, Department of Infectious Diseases, Boston, United States, ^13^University of Miami, Department of Psychology, Coral Gables, United States

**Background:** Men who have sex with men (MSM) continue to be the largest risk group for HIV infections in the U.S., where crystal methamphetamine abuse heightens risk for HIV infection through greater engagement in condomless anal sex (CAS). One potential contributor to the intractability of existing crystal methamphetamine treatments may be a lack of attention to replacement activities or the role of depressed mood. Behavioral activation (BA) is an evidence‐based approach for depression that involves identifying and participating in pleasurable, goal‐directed activities. The hypothesis was that, for MSM abusing crystal methamphetamine, re‐learning how to engage in non‐drug‐using aspects of life would facilitate their ability to benefit from sexual risk reduction (SRR) counseling.

**Methods:** Project IMPACT was a pilot randomized controlled trial. Forty‐six MSM at sexual risk of acquiring HIV who met DSM‐IV criteria for crystal methamphetamine dependence were enrolled. Of those MSM, 41 were randomized: 21 were assigned to the intervention, consisting of two sessions of SRR, ten sessions of BA with SRR, and one session of relapse prevention (13 sessions total); 20 participants were assigned to a control condition (two sessions of SRR).

**Results: ** At the acute post‐intervention visit, intervention participants reported an average of 3.2 CAS acts with men who were HIV‐infected or whose status they did not know, compared to 4.5 among control participants (β = −0.36;95% CI: −0.69, −0.02; *p* = 0.035). At the 6‐month post‐intervention visit, intervention participants reported 1.1 CAS acts with men who were HIV‐infected or whose status they did not know compared to 2.8 among control participants (β = −0.95;95% CI: −1.44, −0.46; *p* < 0.0001). Similarly, intervention participants reported 1.0 CAS acts under the influence of crystal methamphetamine with men who were HIV‐infected or whose status they did not know compared to 2.5 among control participants (β = −0.87;95% CI: −1.38, −0.36; *p* = 0.0005). Lastly, intervention participants reported more continuous days abstaining from crystal methamphetamine compared to control (50.1 vs. 39.0, respectively) (β = 0.25;95% CI: 0.16,0.34; *p* < 0.0001).

**Conclusions: **Findings are encouraging, provide evidence of feasibility (98% retention at six months) and acceptability (93% of counseling sessions attended; all participants rated the intervention as “acceptable” or “very acceptable”), and demonstrate initial efficacy for reducing sexual risk for HIV and crystal methamphetamine use. Future testing in a fully‐powered efficacy trial is warranted.

## WEPDC0206

### Association between syndemics (of alcohol and drug use, and violence) and HIV‐related sexual risk among men who have sex with men in India: findings from a large‐scale national bio‐behavioural survey

**V. Chakrapani^1^; P.V.M. Lakshmi^1^; P. Kumar^2^ and V. Srinivas^2^**

^1^Postgraduate Institute of Medical Education and Research (PGIMER), Chandigarh, India, ^2^National AIDS Control Organisation (NACO), New Delhi, India

**Background:** Despite more than a decade of HIV prevention interventions among men who have sex with men (MSM) in India, consistent condom use is suboptimal (about 50%). Syndemics theory states that multiple co‐occurring psychosocial health problems could synergistically increase negative outcomes (e.g. HIV risk). We examined whether syndemic conditions such as alcohol use, drug use (including injecting drug use), and violence victimisation contribute to HIV‐related sexual risk among MSM.

**Methods:** We used data from a national Integrated Bio‐Behavioural Surveillance (IBBS), a cross‐sectional survey among 23,081 MSM recruited through probability‐based sampling from 24 states in India. We used three syndemic indicators:

(1) alcohol use (at least once in the past week);

(2) drug use (including injectable drugs in the past year); and

(3) violence victimisation (physical and/or sexual violence in the past year).

Inconsistent condom use in anal sex (“sexual risk”) was assessed by how frequently condoms were used in the past month (“every time” coded as consistent; the rest as inconsistent). Logistic regression was used predict to predict inconsistent condom use with three syndemic indicators and their interaction terms (alcohol use x drug use x violence victimisation).

**Results: ** Participants’ mean age was 27.6 years (SD 7.4). Most had completed secondary school, and 37% had paying partners. Prevalences of alcohol use, drug use, and violence victimisation were 48.8%, 11.1% and 24.7%, respectively. With the interaction terms of 3 syndemic conditions in the model (Table 1), alcohol use (aOR = 1.09, *p* < 0.01), drug use (aOR = 1.26, *p* < 0.001), and violence victimisation (aOR = 1.46, *p* < 0.001) were found to significantly predict inconsistent condom use. Further, the interaction terms of the three syndemic indicators were also significant and positive in sign, indicating the synergistic effect of co‐occurring syndemic conditions in heightening the sexual risk.

**Conclusions: **Syndemic conditions are highly prevalent and synergistically increase sexual risk among MSM. While HIV prevention interventions that address one or two of these syndemic conditions (alcohol and drug use, and violence victimisation) could reduce sexual risk, integrating interventions that jointly screen for and address syndemics, in addition to routine condom promotion interventions, could substantially reduce sexual risk among MSM.

**Abstact WEPDC0206‐Table 1.**

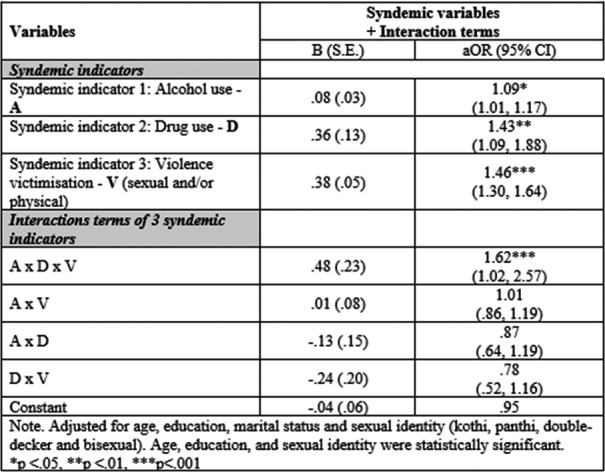

## WEPDD0101

### Examining the socioeconomic gradient in viral suppression in Malawi

**L. Sithole^1^; A. Palma^2^; D. Payne^3^; E. Kim^3^; C. West^3^; M. Nzima^4^; G. Bello^5^ and MPHIA Study Team**

^1^National AIDS Commission, Planning, M&E and Research, Lilongwe, Malawi, ^2^Columbia University, ICAP, New York City, United States, ^3^Center for Diseases Control, Lilongwe, Malawi, ^4^UNAIDS, Lilongwe, Malawi, ^5^Ministry of Health, Lilongwe, Malawi

**Background:** Achieving viral suppression is associated with better health outcomes for people living with HIV. Studies in some countries have revealed socioeconomic disparities in viral suppression. Due to lack of valid measures of socioeconomic status, most studies construct wealth indices based on household asset variables often collected in population‐based surveys. There are, however, multiple techniques for constructing a wealth index and there is lack of consensus regarding which technique is superior. This study examined the socioeconomic gradient in viral suppression in Malawi, checking robustness of results to choice of method for constructing the wealth index.

**Methods:** The Malawi Population‐based HIV Impact Assessment (MPHIA) was a nationally representative survey conducted in Malawi in 2015 to 2016. Among 17,187 adults aged 15 to 64 who were interviewed and tested for HIV using the national algorithm, 2227 (13%) tested HIV positive, and 2220 (99.7%) of these had a viral load test performed. The Erreygers Index (EI) for income‐related health inequality was computed for viral suppression under three scenarios based on statistical procedures for deriving the wealth index: Standard Principal Components Analysis (PCA), Multiple Correspondence Analysis (MCA) and Uncentered Principal Components Analysis (UPCA). All analyses were adjusted for the survey's complex design using jackknife replicated weights. These weights were also applied to calculation of viral suppression rates within the quintiles.

**Results: ** Across the PCA and MCA scenarios, viral suppression rates appear to slightly increase monotonically from lowest to the highest wealth quintile while in the UPCA scenario, viral suppression rates vary only marginally and non‐monotonically across the quintiles. On the other hand, the EI for viral suppression using the standard PCA wealth index was statistically insignificant (EI value = 0.0120; *p*‐value = 0.2860). The EI for the MCA and UPCA indices were also not statistically significant (EI values = 0.00743 and −0.0204; *p*‐values of 0.5128 and 0.0860 respectively).

**Conclusions: **Based on the inequality index used in this study, there were no substantial differences with respect to the socioeconomic gradient in viral suppression across the three scenarios, which implies there are no socioeconomic disparities in viral suppression among PLHIV in Malawi. This result is robust to choice of method for constructing the asset index.

**Abstract WEPDD0101‐Table 1. Socioeconomic Gradient in Viral Suppression in Malawi**

**Table:** 

	PCA	MCA	UPCA
Viral suppression rates across wealth quintiles			
Lowest	15.44%	15.85%	21.91%
Second	16.64%	16.81%	18.42%
Middle	18.8%	20.02%	20.67%
Fourth	22.23%	21.83%	18.84%
Highest	26.89%	25.49%	20.16%
Socioeconomic disparities			
EI	0.012	0.00743	−0.0204
*p*‐value	0.286	0.5128	0.086

## WEPDD0102

### Food insecurity is common and associated with unsuppressed viral load in HIV‐infected pregnant women in Kenya

**K. Ronen^1^; L. Osborn^2^; B. Khasimwa^2^; B. Chohan^2,3^; D. Matemo^2^; J. Unger^1^; A. Drake^1^; J. Kinuthia^2,4^ and G. John‐Stewart^1^**

^1^University of Washington, Seattle, United States, ^2^Kenyatta National Hospital, Nairobi, Kenya, ^3^Kenya Medical Research Institute, Nairobi, Kenya, ^4^University of Nairobi, Nairobi, Kenya

**Background:** Viral suppression in HIV‐infected pregnant women is essential to elimination of mother‐to‐child transmission. Limited data exist on the impact of food insecurity on viral suppression in pregnant women. We evaluated prevalence of food insecurity and its association with viral suppression among pregnant women in Kenya.

**Methods:** We conducted a cross‐sectional analysis of enrollment data from a trial evaluating mHealth strategies to improve ART adherence (Mobile WAChX, NCT02400671). Participants were age ≥14, HIV‐infected, pregnant and had daily access to a mobile phone. Participants were recruited from 6 public MCH clinics in Nairobi and Nyanza region. Self‐report questionnaires and plasma viral load (VL) were collected. Viral suppression was defined as VL <1000 copies/ml among women on ART ≥6 months. Food insecurity, depression and social support were assessed using the Household Food Insecurity Access Scale (HFIAS), PHQ9 and MOS respectively. HFIAS scores were categorized per instrument guidelines and dichotomized into secure/mildly‐insecure versus moderately/severely‐insecure. Correlates of food insecurity were assessed by univariable Poisson regression with robust standard errors.

**Results: ** Eight‐hundred twenty‐five women were enrolled, of whom 820 had complete data for this analysis. Median age was 27 (IQR 23 to 31), gestational age was 24.3 weeks (18.3 to 29.6), monthly income was 8000 KSh (˜80 $US). Women had a median of 2 (1 to 2) living children and 695 (84.3%) were married/cohabiting. Overall, 336 (41.0%) were food secure, 72 (8.8%) mildly insecure, 179 (21.8%) moderately insecure, and 233 (28.4%) severely insecure. Prevalence of moderate/severe insecurity was associated with older age (RR 1.02, 95% CI 1.01 to 1.03, per year), lower income (RR 0.95, 0.94 to 0.97, per $10 increase), more children (RR 1.17, 1.12 to 1.22, per child), ≥mild depression (RR 1.64, 1.44 to 1.86), and lower social support (RR 0.81, 0.76 to 0.86, per 1‐point increase on 4‐point scale). Of 442 women on ART ≥6 months, 385 (87.1%) were virally suppressed. Moderate/severe food insecurity was associated with unsuppressed VL (RR 1.88, 1.10 to 3.22).

**Conclusions: **Our findings suggest that approximately half of pregnant HIV‐infected women in Kenya experienced moderate or severe food insecurity. Food insecurity was significantly associated with older age, lower income, more children, lower social support, depression and unsuppressed VL. Nutrition assistance may be useful to support viral suppression in pregnant women.

## WEPDD0103

### The impact of homelessness in achieving viral suppression among extremely low‐income HIV‐infected women living in a well‐resourced US city: a longitudinal perspective of overlapping risks

**E.D. Riley^1^; E. Vittinghoff^2^; K.A. Christopoulos^1^; A. Clemenzi‐Allen^1^; S.E. Dilworth‐Johnson^1^; C.A. Koss^1^ and A.W. Carrico^3^**

^1^University of California, Medicine, San Francisco, United States, ^2^University of California, Epidemiology and Biostatistics, San Francisco, United States, ^3^University of Miami, Department of Public Health Sciences, Miami, United States

**Background:** Current best practices and high‐impact interventions focus on retention in care and ART adherence to improve HIV outcomes. Simultaneously, research consistently indicates non‐clinical conditions due to poverty as predictors of clinical outcomes. We considered social, structural and clinical factors as predictors of unsuppressed viral load (VL) in a community‐recruited cohort of homeless and unstably housed women living with HIV/AIDS (HUH‐WLWHA). The study was conducted in San Francisco, a well‐resourced US city where unsuppressed VL among HIV‐infected persons is estimated to be 12% to 28%.

**Methods:** Using three years of biannual data, paired with electronic VL data from routine clinical care, we estimated the odds of unsuppressed VL (HIV RNA >200 copies/mL) between 2008 and 2012. Time periods before/after the introduction of universal ART (1/1/10) were considered. Time‐dependent covariates included homelessness, food insecurity, incarceration, insurance, outpatient visits, case management, polysubstance use and violence (emotional, physical, sexual), all evaluated 0 to 3 months before VL assessments. Logistic models with robust standard errors were used to assess the associations of covariates with detectable VL.

**Results: ** Among 120 HUH‐WLWHA, 508 VL assessments were analyzed (median = 3/patient; IQR = 2 to 6). Median baseline age was 47 and 72% of participants reported non‐Caucasian race/ethnicity. Unsuppressed VL was observed among 60% of participants during follow‐up and 19% were unsuppressed at all visits. In adjusted longitudinal analysis, the odds of unsuppressed VL increased 11% for every 10 nights homeless (OR = 1.11, *p* < 0.001) and were over four‐fold higher among recently incarcerated women (OR = 4.46, *p* < 0.001). Adjusting for substance use, odds were also three‐fold higher among participants experiencing recent sexual violence by someone who was not a primary intimate partner (OR = 3.10, *p* = 0.009).

**Conclusions: **In a city where 72% to 88% of HIV‐infected persons have undetectable viremia, only 40% of HUH‐WLWHA remain undetectable for three years. Homelessness, incarceration, sexual violence and drug use increase the odds of unsuppressed VL over time; other social/structural factors and healthcare do not. Current best practices and high‐impact interventions do not address the root of the problem for impoverished WLWHA in a resource‐rich setting. Considered alongside prior research, results suggest multi‐component interventions that prioritize housing as being critical to optimizing health outcomes in this high‐risk population.

## WEPDD0104

### Unstable housing associated with injection risk behaviors among PWID in Ukraine

**A. Mazhnaya^1,2^ and J. Owczarzak^1^**

^1^Johns Hopkins School of Public Health, Department of Health, Behavior and Society, Baltimore, United States, ^2^ICF Alliacne for Public Healtj, Treatment, Kyiv, Ukraine

**Background:** In Eastern Europe and Central Asia new HIV infections occur at a high rate among people who inject drugs (PWID). Moreover, PWID account for most hepatitis C (HCV) cases in EECA. Injection risk behaviors are considered to be a product of “risk environment” which includes socioeconomic factors, such as housing. There has been, however, limited attention towards exploring association between PWID's housing situation and injection risk behaviors in Ukraine which has HIV prevalence 21% to 42%, and 60% HCV prevalence among PWID.

**Methods:** This study is based on a data from a baseline survey of PWID (n = 684) recruited to participate in a behavioral HIV prevention intervention in 4 urban settings across Ukraine. Using poisson regression model we estimated independent association between housing situation (stable vs. unstable) and injection risk behaviors within last 30 days controlling for age, sex, city, drug of choice and education.

**Results: ** Participants who reported unstable housing also reported more injection risk behaviors within last 30 days, including using old syringe (33% vs. 10%, *p*‐value <0.001), sharing cooker/cotton/water (25% vs. 15%, *p*‐value 0.1), front/backloading (73% vs. 62%, *p*‐value 0.1), using preloaded syringe (64% vs. 48%, *p*‐value 0.06). In multivariable analysis having unstable housing was associated with almost two‐fold increase in using needles/syringes after someone or sharing injecting equipment (cooker, cotton, water) within last 30 days (prevalence ratio (PR) 1.91, 95% CI 1.14 to 3.20).

**Conclusions: **Understanding and addressing structural context associated with injection risk behaviors should be part of research and intervention agenda to fight HIV and HCV in Ukraine. National programs would benefit from expanding models to include structural determinants of health.

## WEPDD0105

### Displacement, urban gentrification and declining access to HIV/STI, sexual health and outreach services amongst women sex workers between 2010 and 2014: results of a community‐based longitudinal cohort

**S. Goldenberg^1,2^; O. Amram^3^; M. Braschel^1^; S. Machat^1^; K. Shannon^1,4^ and on behalf of the AESHA Study Team**

^1^Gender and Sexual Health Initiative, BC Centre for Excellence in HIV/AIDS, St. Paul's Hospital, Vancouver, Canada, ^2^Simon Fraser University, Faculty of Health Sciences, Burnaby, Canada, ^3^Washington State University, Elson S. Floyd College of Medicine, Spokane, United States, ^4^University of British Columbia, Faculty of Medicine, Vancouver, Canada

**Background:** Despite increasing gentrification across North American cities, little is known about its impacts on work and living environments and health access amongst sex workers (SWs). Using a spatial epidemiological approach, we

(1) explored changes in land use and work/living environments in relation to gentrification exposure, and

(2) modeled the independent effect of gentrification exposure on utilization of HIV/STI testing and sexual/reproductive health (SRH) and SW‐tailored services.

**Methods:** Data were drawn from a community‐based longitudinal cohort of SWs (AESHA) and publically available land use data. Analysis was restricted to 2010 to 2014, given legal changes in December 2014. Changes in land use and SWs’ residential and working environments were mapped for the pre‐gentrification (2010) vs. gentrification (2014) periods. Using a before‐and‐after design, confounder modeling with multivariable logistic regression using generalized estimating equations (GEE) assessed the independent effect of gentrification exposure on utilization of HIV/STI testing and SRH and SW‐led/tailored services. Analyses were restricted to 203 SWs who participated in both study periods; spatial analyses were further restricted to those who provided data on work/residential locations.

**Results: ** In the Downtown Eastside (DTES) and Strathcona neighborhoods, major shifts away from industrial/commercial land use towards residential, newly built housing were observed. Increases in the distance between the DTES core and SWs’ place of residence (547 vs. 764 meters, *p* = 0.03) and servicing clients (528 vs. 1894 meters, *p* = 0.06) were found; shifts away from street/public (72.9% vs. 30.5%) towards online/off‐street solicitation (15.8% vs. 28.6%, *p* < 0.001) were also documented. In separate multivariable GEE models adjusted for key confounders, gentrification exposure was correlated with reduced utilization of HIV (AOR: 0.33, 95% CI: 0.21 to 0.51) and STI testing (AOR: 0.39, 95% CI: 0.26 to 0.61), and use of SRH (AOR: 0.36, 95% CI: 0.23 to 0.56) and SW‐led/tailored services (AOR: 0.43, 95% CI: 0.25 to 0.75).

**Conclusions: **Observed decreases in use of HIV, STI, SRH and SW‐tailored services occurred despite ongoing efforts to scale‐up HIV cascade interventions for key populations in BC, Canada. Results suggest these declines may be linked to gentrification‐related displacement of SWs from areas where services for key populations are concentrated. Health and housing policies that promote marginalized women's access to safe working/living environments and investment in SW‐led and SW‐friendly health and social services are critically needed.

## WEPDE0101

### Lessons learned from sustained global health investments

**A. Downer^1^; J. Johnstone^1^; M. McDowell^1^ and E.M. Reyes^2^**

^1^University of Washington, Global Health, Seattle, United States, ^2^University of California, Global Health Institute, San Francisco, United States

**Background:** There is emerging evidence to support those factors in global development that are related to successful transition and sustainability of aid investments. At the 15 year mark, the scale and scope of the the US President's Emergency Plan for AIDS Relief (PEPFAR) program provides ample opportunity to explore these factors and to confirm or identify those that can be incorporated early in project design to increase the sustainability of investments.

**Description:** The International Training and Education Center for Health (I‐TECH) at the University of Washington in Seattle, USA, has been a PEPFAR partner since 2002. I‐TECH has transitioned more than 300 programs, products, and tools to local ownership in that time. In 2017, I‐TECH undertook to explore the degree to which a sub‐set of these improvements have been sustained by local partners over time. Case examples were selected on the basis of geographic diversity, type of intervention, and sufficient time from transition to make an assessment. Key informants were interviewed, and the four domains and 15 elements of the PEPFAR Sustainability Index Dashboard (SID) were used to provide a framework and starting point for better understanding each example.

**Lessons learned:** Case studies were drafted on transitioned interventions in six resource limited countries to illustrate lessons learned on the long‐term sustainability of health systems improvements, including both successes and failures. The cases reinforce the relevance of the SID in planning for sustainability, in particular the elements of advanced planning and coordination (1); human resources for health (7); quality management (9); domestic resource mobilization (11); and performance data (15). In addition, we found the actions of the implementing partner during transition to be a critical component.

**Conclusions/Next steps:** While aid investments in low and middle income countries can clearly be transitioned successfully to local ownership, they may not remain beneficial over time unless key elements of sustainability planning are intentionally addressed at the outset. Such lessons learned are instructive to a wide set of global audiences, from health and development specialists, government officials, economists, and social scientists to diplomats and security professionals worldwide. As such, lessons should be regularly disseminated.

## WEPDE0102

### How it was possible to offer Integrase Inhibitor as first line ART while maintaining the sustainability of the Brazilian policy of universal access to drugs

**C.J.B. Batista^1^; R.G. Corrêa^2^; E. Malheiros^2^; M.D.A. Freitas^2^ and A.S. Benzaken^2^**

^1^Ministry of Health, STI/AIDS and Viral Hepatitis, Brasília, Brazil, ^2^Ministry of Health, Brasília, Brazil

**Background:** Brazilian antiretroviral therapy is free and offered to 100% by Federal Government. In December 2016, about 500,000 people were on antiretroviral therapy (ART) and it was estimated that 100,000 new patients would be included in 2017. The Brazilian protocol recommended EFZ/TDF/3TC regimen as first line ART, and Integrase Inhibitors (II) as rescue therapy (Raltegravir – RAL 400 mg). As a result of the advances in international studies about the benefits of incorporating II in first line ART,

the Brazilian government initiated the negotiations to offer II, even considering the impossibility of generic drugs acquisition due to patent laws.

**Methods:** With an annual budget of approximately 350 million dollars to purchase antiretroviral drugs, the objective of the government was to offer II without significantly increasing the budget. Therefore, two strategies were used: a) price negotiation through bidding processes for the two II options available in Brazil (DTG 50 mg and RAL 400 mg) – only one of these would be included in the guidelines as a preferential first line drug and the same medication would be indicated for rescue regimens; b) reorganization of the guideline drug portfolio, including the removal of obsolete drugs and recommendations on switching patients to the new regimens.

**Results: ** In 2016, treatment cost with available II in Brazil was US 8.8 dollars/day; the negotiation allowed a reduction to US 1.50/day – purchase of DTG. DTG was then included in the guidelines as the preferential drug for first line ART; switching RAL to DTG as rescue regimens was also recommended. In relation to the portfolio reorganization, the following were excluded: Fosamprenavir, Didanosine, Stavudine, and Saquinavir as well as the change in the recommendation of Atazanavir as the preferential drug for second line ART. These actions permitted ART procurement for 2017, including DTG, without significantly increasing the budget, as shown in Figure 1.

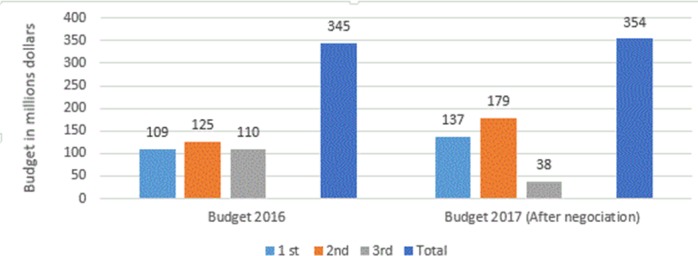

**Abstract WEPDE0102‐Figure 1. Comparison of budget used to purchase ARV per line of ART in the years of 2016 and 2017, Brazil.**

**Conclusions: **The strategies used by Brazil proved advantageous and made it possible to offer a better antiretroviral treatment without significant budget changes.

Even with the increase in cost with the first and second lines, the economy generated in the third line was decisive for the expansion of the use of II.

## WEPDE0103

### Cost of HIV care and treatment in Mozambique

**A. Berruti^1^; A. Krivelyova^2^; F. Mbofana^3^; D. Single^4^ and A. Vergara^4^**

^1^U.S. Centers for Disease Control and Prevention, Division of STD Prevention/NCHHSTP, Atlanta, United States, ^2^ICF, Atlanta, United States, ^3^Mozambique Ministry of Health, Maputo, Mozambique, ^4^U.S. Centers for Disease Control and Prevention, DGHT, Atlanta, United States

**Background:** In 2016, approximately 990,000 adults and 76,000 children in Mozambique were receiving antiretroviral therapy. This study quantified costs of HIV treatment and care of different patient groups incurred in publicly funded HIV treatment facilities.

**Methods:** We collected data on the costs of the HIV treatment program, volume, and types of patients being treated. Data were collected retrospectively for two 6‐month periods during 2016. Costs were categorized by source of support, type of expenditure, and program activity. Analyses focus on: total per‐patient costs, cost across categories, and costs over time. Per‐patient cost calculation was based on resource use intensity and allocation of costs across different patient categories.

**Results: ** The median economic cost per patient‐year was $96.73 (2017 USD) for ART patients and $22.09 for pre‐ART patients. Costs were higher for newly initiated adult and pediatric patients compared to established patients. ARVs cost a median of $67.07 for ART patients, the largest single cost component for these patients. With ARVs excluded, laboratory services constituted the most substantial cost category for ART and pre‐ART patients, at a median of $11.10 and $10.82, respectively. No substantial investments were made over the study period in equipment, training, or infrastructure. Per‐patient cost comparisons across different types of facilities revealed lower costs in rural facilities ($88.13 vs. $115.49 and $109.33 in peri‐urban and urban facilities, respectively). Costs were higher in secondary level facilities ($121.07 vs. $85.65 and $99.33 in primary and tertiary facilities, respectively). Analysis of costs by patient volume indicated that some economics of scale were present with larger facilities having smaller per patient costs.

**Conclusions: **Treatment costs varied widely between facilities, reflecting differences in the clinical models used, intensity of services, and types of drugs provided. While the costs of antiretroviral drugs may still dominate the cost per‐patient, personnel and laboratory supplies costs are not insignificant. The study results indicate that HIV services can be optimized as the number of patients grows without substantial increases in investment. Under the test and start model, this study demonstrates that efficiency can be achieved if more patients start treatment, and this will in turn improve allocative efficiency.

## WEPDE0104

### Building sustainable HIV service delivery model at a local level in Ukraine

**O. Iaremenko; A. Vasylkova; L. Khomych; N. Avaliani; N. Davydenko and M. Makovetska**

USAID Project HIV Reform in Action, Deloitte, Kyiv, Ukraine

**Background:** In 2018, Ukraine remains the second highest‐burden HIV epidemic country in Europe, with an estimated 241,000 people living with HIV/AIDS (PLWH). The significant financial dependence on external donor resources (67.41% of total HIV funding) threatens the sustainability of critical HIV services provision in the near future. As donor funding for HIV programming continues to decline in middle‐income countries, Ukraine needs to revise its national HIV/AIDS response strategy and funding allocations, and optimize current service delivery models.

**Description:** Between February 2016 and December 2017 the USAID‐funded HIV Reform in Action Project supported 14 local state administrations in 7 high‐burden regions of Ukraine in developing and piloting a sustainable model for provision and scale‐up of critical HIV services for PLWH and key populations.

The key components of the sustainable model include:

(1) advocacy for increased local funding for HIV rapid testing and social services;

(2) removal of policy and legal barriers to service provision by local government and NGOs;

(3) introducing new financing models for local state administrations;

(4) decentralization and integration of HIV services, and

(5) optimizing human resources for community‐level service delivery.

To facilitate the pilot, we established mechanisms to coordinate efforts across regional and local authorities, primary and secondary level health care providers and civil society service providers.

**Lessons learned:** Local level advocacy efforts resulted in strengthening partnerships between government and civil society in HIV service delivery and increased 14 local budgets for HIV response from $225K in 2015 to $1.96M in 2017. Bridging local policy gaps in provision of HIV services at primary healthcare centers (PHCs), education and the local resource allocation allow to ten‐fold increase in the number of HIV rapid testing sites at PHCs, with a six‐fold increase in the quantity of HIV rapid tests procured with local budgets. Thirty five new opioid substitution therapy (OST) sites were opened, with an increase of 536 people who inject drugs receiving OST services (compared to 2015).

**Conclusions/Next steps:** Pilot results and a replication plan are being shared with local state administrations beyond the pilot regions to fast‐track comprehensive, integrated and sustainable HIV response at a national level.

## WEPDE0105

### Sustainable financing of the HIV response in Vietnam: Integration of donor funded treatment facilities into public health system and the social health insurance scheme

**H.T. Nguyen^1^; H.T. Nguyen^1^; A.T.C. Nguyen^2^; H. Pham^1^; A.N. Le^1^; L. Dam^1^; D. Nguyen^1^; N. Todini^1^; N.T. Do^3^; H.T.T. Phan^3^ and T.M. Hammett^1^**

^1^USAID/Health Finance and Governance Project, Abt Associates, Hanoi, Vietnam, ^2^USAID Hanoi, Vietnam, Hanoi, Vietnam, ^3^Vietnam Administration of HIV/AIDS Control, Ministry of Health, Hanoi, Vietnam

**Background:** As international donors reduce support for the HIV response in Vietnam (currently ˜70%) and many other countries, the Government of Vietnam (GVN) is implementing a unique and innovative transition strategy: strengthen domestic governance and financing by integrating donor‐funded HIV treatment facilities into the public health system with coverage of services through an expanded Social Health Insurance (SHI) scheme. Implementing partners of PEPFAR, including the Health Finance and Governance (HFG) project, have been providing technical assistance in support of this transition since 2015.

**Description:** HFG provides technical assistance to the Vietnam Administration of HIV/AIDS Control (VAAC) and other departments of the Ministry of Health (MoH), Vietnam Social Security (VSS), and 9 provinces including Hanoi and Ho Chi Minh City, the two provinces with the highest HIV burden. Beneficiaries include >51,000 ART patients (˜46% of the total in Vietnam) in 115 treatment facilities. Technical assistance at national level includes: development of an integration monitoring tool and ongoing assessment of transition progress. HFG assists provinces and treatment facilities to achieve key integration steps of SHI contracting and reimbursement, and expansion of SHI coverage among PLHIV. The table and graph show the dramatic integration progress in the 9 HFG‐supported provinces and the predominant contribution of PEPFAR TA to national progress on SHI reimbursement for HIV services. SHI coverage among ART patients in the 9 HFG supported provinces increased from 36% in 2016 to73% in 2017.

**Lessons learned:** Multi‐sectoral and multi‐level technical assistance on integration of parallel systems of HIV treatment and expansion of population and service coverage of SHI can achieve remarkable progress in the transition from predominantly donor‐funded to locally‐funded and operated HIV responses.

**Conclusions/Next steps:** In Vietnam, full integration will only be completed when all HIV treatment services including viral load testing and ART are covered and reimbursed by SHI (expected to start in 2018 for VL testing and in 2019 for ART) and all PLHIV are enabled to obtain SHI through appropriate subsidies. PEPFAR and HFG will continue to provide TA to GVN at all levels to achieve these objectives, which will in turn help Vietnam achieve the 90‐90‐90 goals.

**Abstract WEPDE0105‐Table 1. Integration progress in HFG‐supported provinces**

**Table:** 

Indicators	Baseline (December 2015) (n/%)	December 2016 (n/%)	December 2017 (n/%)	Expected June 2018 (n/%)
HIV treatment facilities with SHI contracts	0	47/109 (43%)	83/115 (72%)	106/115 (90%)
HIV treatment facilities with SHI reimbursement	0	24/109 (22%)	70/115 (61%)	93/115 (81%)

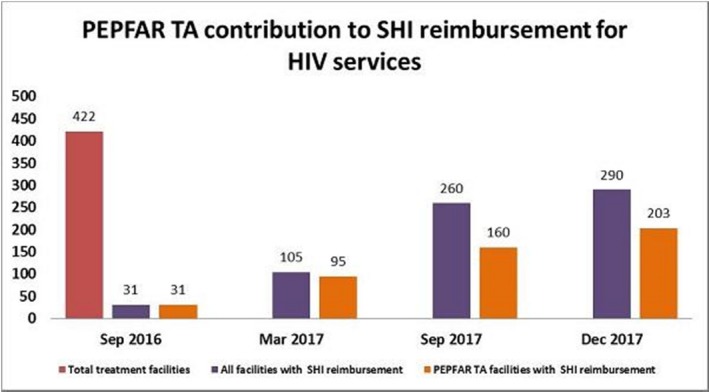

**Abstract WEPDE0105‐Figure 1. PEPFAR TA contribution to SHI reimbursement for HIV services.**

## WEPDE0106

### PEPFAR's Sustainability Index and Dashboard: results from SID 3.0

**C. Hart; M. Ruffner; J. Timberlake and M. Wollmers**

US Department of State/PEPFAR, Washington, United States

**Background:** The achievement of sustained control of the HIV epidemic is a critical goal for individuals, communities, and governments. However, defining sustainability and measuring progress over time has proved challenging. PEPFAR's Sustainability Index and Dashboard (SID) defines critical elements that contribute to sustainability, and enables its users to assess the current state of national HIV/AIDS responses while identifying strengths and vulnerabilities over time. After its third iteration, SID 3.0 provides results that reveal important insights into common challenges and advances across countries.

**Description:** SID 3.0 was implemented in 40 countries in 2017 through a participatory process. PEPFAR country teams worked with UNAIDS country directors to convene partner governments, multilateral and bilateral donor organizations, civil society organizations, private sector partners, and PEPFAR country staff. These stakeholders completed the 90‐question SID tool to assess sustainability across 15 elements organized into four domains.

**Lessons learned:** The results of SID 3.0 demonstrate that countries are making progress towards a sustainable response to the epidemic. When analyzed by income level, low income countries generally scored lower than middle income countries on SID elements related to national health systems. Additionally, differences were observed by type of PEPFAR support, with technical assistance and technical collaboration countries generally performing better than long‐term strategy countries. Among thirteen PEPFAR partner countries that are poised to reach epidemic control, scores from SID 2.0 (2015) to SID 3.0 (2017) increased significantly, by an overall average of 0.40 points (from 5.97 to 6.37, on a 0.00 to 10.00 scale) across all elements.

**Conclusions/Next steps:** PEPFAR has designed an original index that enables important cross‐country comparisons that highlight which critical variables support advancement of sustainable systems to control the HIV epidemic. The SID provides essential data used to determine health systems investments and metrics to track the impact of those investments over time. Its regular implementation leads to results that in combination with epidemiologic data yield valuable insights about the most efficient path to sustained epidemic control. At the country level, the SID framework orients national stakeholders to their sustainability strengths and challenges, and facilitates informed decisions about where to target effort and resources to respond to the HIV epidemic more efficiently.

## THPDA0102

### Shorter cell subset telomeres in HIV slow progressors than in HIV non‐slow progressor women

**A. Hsieh^1^; B. Sattha^1^; N. Bernard^2^; C. Tremblay^3^; H. Côté^1,4^; for the CIHR Team on Cellular Aging; HIV Comorbidities in Women and Children (CARMA)**

^1^University of British Columbia, Pathology and Laboratory Medicine, Vancouver, Canada, ^2^McGill University Health Centre Research Institute, Montreal, Canada, ^3^Centre de Recherche du Centre Hospitalier de l'Université de Montréal, Montreal, Canada, ^4^Centre for Blood Research, Vancouver, Canada

**Background:** Although shorter leukocyte telomere lengths (TL) of people living with HIV have been reported, TL investigations in HIV‐relevant immune subsets remain scarce. Immune aging, including subset‐specific TL attrition and imbalance in senescent/proliferative CD8 T cell distributions, likely link HIV with premature age‐related comorbidities among people on stable cART. It is unknown whether this link exists for HIV slow progressors (SP). Our objective was to characterize immune subset TL in SP to determine whether their ability to control HIV protects against HIV‐modulated immune aging. We hypothesized that TL shortening and senescent CD8 T cell subset expansion would be mitigated in SP compared to cART‐controlled HIV+ non‐slow progressors (NSP).

**Methods:** Live PBMCs were obtained from cART‐controlled NSP and HIV‐ women enrolled in the CARMA cohort, and SP from the Canadian Cohort of HIV+ Slow Progressors. Groups were matched 1:1:1 for age. CD4 T cells, proliferative (CD28 + ), senescent (CD28‐) CD8 T cells, and (CD19 + ) B cells were sorted by FACS, and their relative TL measured by multiplex qPCR. Groups were compared using Mann‐Whitney or χ^2^ tests.

**Results: ** Women (n = 35/group) 27 to 60 years were well‐balanced for age and ethnicity, and CD4 counts were similar between SP/NSP. All NSP were undetectable, while 15/35 SP were ART‐experienced, including two on cART at visit. Cells were sorted from all matched groups and TL data were available for n = 20 to 31, as matched trios with insufficient sorted cells were excluded. Shorter TL was observed in SP proliferative CD8, senescent CD8, and B, but not CD4 T cell subsets, compared to both NSP (*p* < 0.047, n = 20 to 31) and HIV‐ (*p* = 0.002) participants (Figure 1). TL was shorter in senescent compared to proliferative CD8 T cells among SP (n = 34, *p* < 0.001) and NSP (n = 27, *p* = 0.027) but not HIV‐ controls (n = 22, *p* = 0.13). The senescent CD8 compartment was expanded in SP (median CD28‐:CD28+ ratio = 1.67) compared to NSP (0.66, *p* < 0.001) and HIV‐ (0.45, *p* < 0.001) participants.

**Conclusions: **Contrary to our hypothesis, these data strongly suggest that cellular aging, at least within CD8 T and B subsets, may be accelerated among SP compared to HIV+ NSP and HIV‐ women. These results stress the importance of cART treatment and viral suppression in SP.

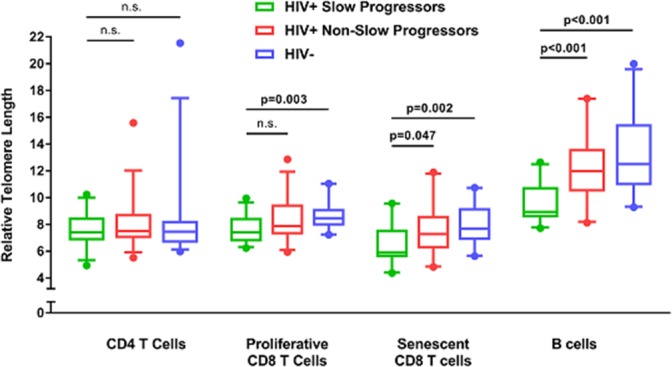

**Abstract THPDA0102‐Figure 1. Relative telomere lengths of immune subsets between HIV+ Slow Progressor, HIV+ Non‐Slow Progressor, and HIV‐ groups.**

## THPDA0103

### Does HIV‐seroconversion affect the serum N‐glycans profile, one possible biomarker of ageing? Insights from a longitudinal study

**C. Pirazzini^1^; P. Garagnani^2^; E. Giampieri^3^; C. Sala^3^; V. Borelli^2^; M.G. Bacalini^1^; D. De Francesco^4^; C.A. Sabin^4^; M. Prins^5,6^; F.N.M.W. Wit^7,8^; N.A. Kootstra^9^; C. Libert^10,11^; C. Franceschi^1^; P. Reiss^7,8^; The Co‐morBidity in Relation to AIDS (COBRA) Collaboration and the Amsterdam Cohort Studies on HIV**

^1^IRCCS Institute of Neurological Sciences, Bologna, Italy, ^2^Alma Mater Studiorum Università di Bologna, Department of Experimental, Diagnostic and Specialty Medicine, Bologna, Italy, ^3^Alma Mater Studiorum University of Bologna, Department of Physics and Astronomy, Bologna, Italy, ^4^Institute for Global Health, University College London, London, United Kingdom, ^5^Public Health Service of Amsterdam, Department of Infectious Diseases, Amsterdam, Netherlands, ^6^Academic Medical Center, Department of Internal Medicine, Amsterdam, Netherlands, ^7^Academic Medical Center and Amsterdam Institute for Global Health and Development, Department of Global Health and Amsterdam Infection and Immunity Institute, Amsterdam, Netherlands, ^8^Stichting HIV Monitoring, Amsterdam, Netherlands, ^9^Department of Experimental Immunology, Amsterdam, Netherlands, ^10^Ghent University, Department of Biomedical Molecular Biology, Ghent, Belgium, ^11^Center for Inflammation Research, Flanders Institute for Biotechnology, Ghent, Belgium

**Background:** Persons living with HIV (PLWH) who are successfully treated with combination antiretroviral therapy can survive to older ages but have an increased risk of age‐related conditions compared to HIV‐negative individuals. Whether HIV infection per sé affects the ageing process and contributes to PLWH's susceptibility to age‐related conditions remains unclear. To address this question, we determined the serum profile of N‐glycans, which are powerful and reliable biomarkers of ageing, in cryopreserved longitudinal serum samples from Amsterdam Cohort Studies on HIV/AIDS (ACS) participants, from before and after HIV‐seroconversion (SC).

**Methods:** The N‐glycans profile was analyzed using longitudinal serum samples from 73 ACS participants (all males, with a mean (SD) age at time of first pre‐seroconversion sampling of 34.8 (7.5) years). For each subject, three samples were obtained between 12 and 6 months *before* SC and three samples were obtained between 6 and 60 months *after* SC. None of the participants were receiving antiretroviral treatment during the time of sampling. Serum glycoproteins were analyzed using DSA‐FACE technology. Ten N‐glycan peaks were detected; a combination of the ten peaks was derived using measurements before SC. This combination was then applied to measurements after SC to estimate the age advancement (difference between chronological and estimated age). The effect of SC on each N‐glycan peak was evaluated using a multivariate linear model including age, SC event and length of time after SC.

**Results: ** We found a significant mean age advancement (sem) after SC of 2.61 (0.16) years. SC was significantly associated with a significant increase in peaks 1 (*p* < 0.001), 2 (*p* < 0.001) and 3 (*p* < 0.001) and a significant decrease in peaks 5 (*p* < 0.001), 7 (*p* < 0.001) (Table 1). The changes in peaks two, five and seven were influenced also by the length of time after SC.

**Abstract THPDA0103‐Table 1. Regression coefficient and p‐value for SC event (after vs. before) adjusted for age and time after SC**

**Table:** 

	Regression coefficient for SC event	*p*‐value for SC event
logP1	0.80	*p* < 0.001
logP2	0.66	*p* < 0.001
logP3	0.73	*p* < 0.001
logP5	−0.55	*p* < 0.001
logP7	−0.40	*p* < 0.001

**Conclusions: **We confirmed the well‐known dependency with age for N‐glycan peaks 1 and 6. Our results suggest that both SC and the length of time after SC may affect the aging process by acting on the glycan profile, including on peaks that thus far have not been routinely used for age prediction.

## THPDA0104

### Association between gut microbiota and CD4 recovery in HIV‐1 infected patients

**W. Lu^1,2^; Y. Feng^3,4,5^; F. Jing^1,2^; Y. Han^1,2^; Z. Qiu^1,2^; T. Li^1,2^ and B. Zhu^3,4,5^**

^1^Peking Union Medical College Hospital, Division of Infectious Diseases, Beijing, China, ^2^Chinese Academy of Medical Sciences and Peking Union Medical College, Center for AIDS Research, Beijing, China, ^3^Chinese Academy of Science, Institute of Microbiology, Beijing, China, ^4^University of Chinese Academy of Science, Beijing, China, ^5^Beijing Key Laboratory of Microbial Drug Resistance and Resistome, Beijing, China

**Background:** Changes in gut microbial compositions have been described in human immuno‐deficiency virus (HIV)‐infected patients on antiretroviral therapy (ART). Evidences suggest that ART‐treated patients with poor CD4^+^ T‐cell recovery have higher levels of immune activation and microbial translocation. However, the association between gut microbiota and immune recovery remains unclear.

**Methods:** We performed a cross‐sectional study on 30 healthy controls (HC) and 61 HIV‐infected individuals, including 15 immunological ART responders (IRs), 20 immunological ART non‐responders (INRs) (IRs and INRs, CD4^+^ T‐cell counts ≥350 cells/mm^3^ and <350 cells/mm^3^ after two years of ART, respectively), and 26 untreated individuals (VU). Each subject's microbiota composition was analyzed by metagenomics sequencing. Levels of CD4^+^ T cells, CD8^+^HLA‐DR^+^ T cells and CD8^+^CD38^+^ T cells were measured by flow cytometry.

**Results: ** We identified more *Prevotella* and fewer *Bacteroides* in HIV‐infected individuals than in healthy controls. Patients in INR group were enriched in *Faecalibacterium prausnitzii*, unclassified *Subdoligranulum sp*. and *Coprococcus comes* when compared with those in IR group. *F. prausnitzii* and unclassified *Subdoligranulum sp*. were overrepresented in individuals in VU group with CD4^+^ T‐cell counts <350 cells/mm^3^. Moreover, we found that the relative abundances of unclassified *Subdoligranulum sp*. and *C. comes* were positively correlated with CD8^+^HLA‐DR^+^ T‐cell count and CD38^+^HLA‐DR^+^/CD8^+^ percentage.

**Conclusions: **Our study has shown that gut microbiota changes were associated with CD4 T cells and immune activation in HIV‐infected subjects. Interventions to reverse gut dysbiosis and inhibit immune activation could be new strategies for immune reconstitution in HIV infected individuals.

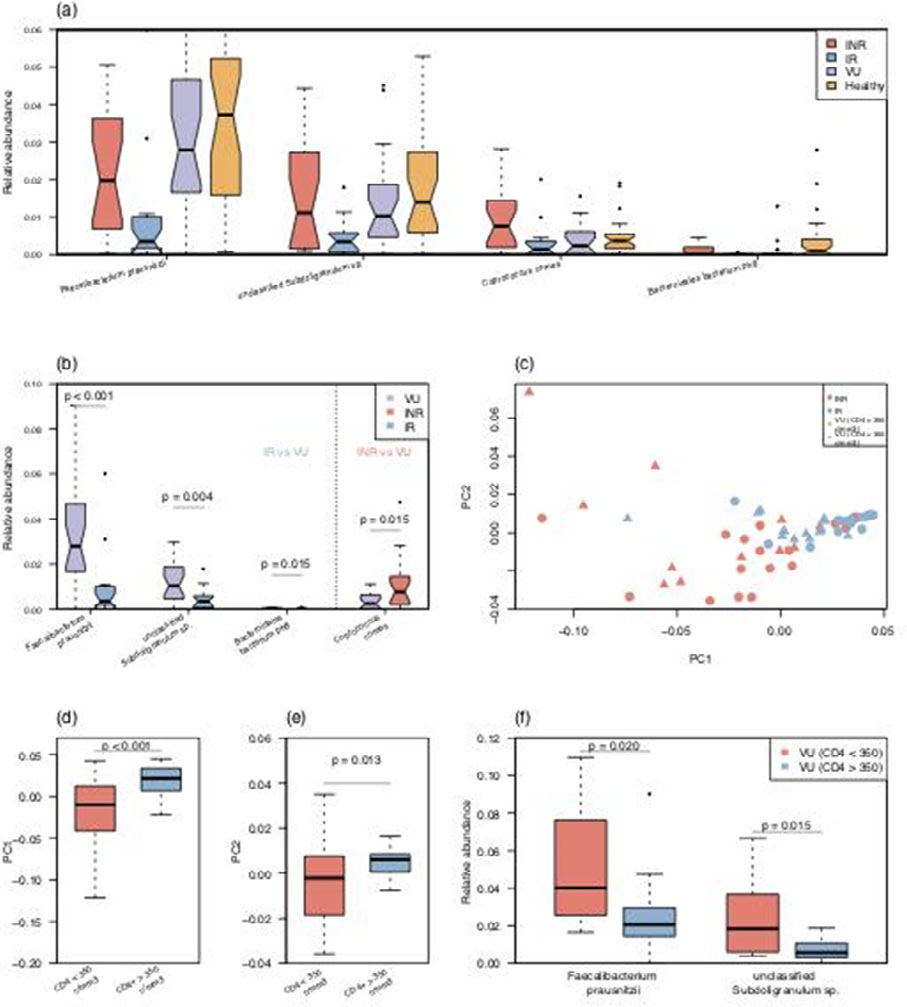

**Abstract THPDA0104‐Figure 1. Differentially abundant species in the IR and INR groups and correlation with CD4 T cells.**

## THPDA0105

### Polymorphism rs1385129 within Glut1 gene SLC2A1 is linked to poor CD4 +  T cell recovery In antiretroviral‐treated HIV+ individuals

**J. Masson^1^; C. Cherry^1,2^; N. Murphy^3,4^; I. Sada‐Ovalle^5^; T. Hussain^3^; R. Palchaudhuri^1^; J. Martinson^6^; A. Landay^6^; B. Billah^3^; S. Crowe^1,3^ and C. Palmer^1,2^**

^1^Burnet Institute, Life Sciences, Melbourne, Australia, ^2^Monash University, Department of Infectious Diseases, Melbiurne, Australia, ^3^Monash University, Melbourne, Australia, ^4^Monash IVF, Melbourne, Australia, ^5^Unidad de Investigación Instituto Nacional de Enfermedades Respiratorias, Mexico City, Mexico, ^6^Rush University Medical Centre, Department of Immunology‐Microbiology, Chicago, United States

**Background:** HIV infection is associated with progressive CD4 +  T cell depletion, which is generally recovered with combination antiretroviral therapy (cART). However, a signification proportion of cART‐treated individuals have poor CD4 +  T cell reconstitution. Abnormal glycolytic activation marked by increased CD4 +  T cell Glucose transporter‐1 (Glut1) expression is associated with low CD4 T cell count in treated HIV infection. We investigated the association between the frequency of circulating CD4 + Glut1 +  T cell, specific single nucleotide polymorphisms (SNPs) within the Glut1 gene, and those regulating Glut, and HIV disease progression in treatment naïve patients and immunological response to cART.

**Methods:** Glut1 levels on CD4 +  T cells was determined by flow cytometry. Study groups comprise 17 HIV‐positive treatment naïve individuals with favourable disease progression (>200 CD4 +  cells/μL within three to seven years, or the loss of <80 CD4 +  cells/μL/year) and 11 with non‐favourable progression (<200 CD4 +  cells/μL within three years of diagnosis, or a loss of >80 CD4 +  cells/μL/year). Treated groups comprise 25 treatment responders (>500 CD4+ cells/μL; >3 years on cART), and 14 non‐responders (<500 CD4+ cells/μL; >3 years on cART). SNP was evaluated in the Glut1 gene SLC2A1 (rs1385129, and rs841853), Glut1 regulatory AKT (rs1130214, rs2494732, rs1130233 and rs3730358), and the antisense RNA 1 region SLC2A1‐AS1 (rs710218).

**Results: ** High CD4 + Glut1 +  T cell percentage is associated with rapid CD4 +  T cell decline in HIV‐positive treatment‐naïve individuals and non‐favourable CD4 +  T cell recovery in HIV‐positive individuals on cART. Poor CD4 +  T cell recovery in HIV+/cART individuals is linked to the homozygous genotype (GG) of SLC2A1 SNP rs1385129 when compared to those with a recessive allele (GA/AA) (OR: 4.67; *p*: 0.04; Univariable logistic regression). CD4 + Glut1 +  T cell percentage is elevated among those with a homozygous dominant genotype for SNPs rs1385129 (GG) and rs710218 (AA) when compared to those with a recessive allele (GA/AA and AT/TT respectively) (*p*: 0.04; Mann‐Whitney). The heterozygous genotype of AKT SNP 1130214 (GT) has a higher CD4 + Glut1 +  T cell percentage when compared to the dominant homozygous genotype (GG) (*p*: 0.0068; Mann‐Whitney).

**Conclusions: **SNPs within genes that regulate glycolysis offer new insights into HIV pathogenesis and factors controlling HIV disease outcomes.

## THPDB0101

### Characterization of doravirine‐selected resistance patterns from participants in treatment‐naïve Phase 3 clinical trials

**M.‐T. Lai^1^; M. Xu^2^; W. Ngo^3^; M. Feng^3^; D. Hazuda^3^; G. Hanna^4^; S. Kumar^5^; X. Xu^6^; E. Martin^4^ and C. Hwang^4^**

^1^Merck & Co., Inc., Kenilworth, United States, ^2^Merck & Co., Inc., Department of Pharmacology, West Point, United States, ^3^Merck & Co., Inc., Department of Antiviral Research, West Point, United States, ^4^Merck & Co., Inc., Department of Clinical Research, West Point, United States, ^5^Merck & Co., Inc., Department of Clinical Sciences and Study Management, West Point, United States, ^6^Merck & Co., Inc., Department of Biostatistics, West Point, United States

**Background:** Doravirine (DOR) is a novel human immunodeficiency type 1 virus (HIV‐1) non‐nucleoside reverse transcriptase inhibitor (NNRTI), with improved potency against prevalent NNRTI resistance‐associated mutations, including RT K103N, Y181C, G190A, and E138K, at clinically relevant concentrations. This study aimed to characterize the mutant viruses selected in treatment‐naïve participants through Week 48 from DRIVE‐FORWARD and DRIVE‐AHEAD, and to assess the impact of selected mutations on NNRTI susceptibility and viral fitness.

**Methods:** Plasma samples from the trials were tested for genotypic and phenotypic NNRTI susceptibility using a Monogram Biosciences resistance assay. Additionally, laboratory mutant isolates were generated via a site‐directed mutagenesis (SDM) method with gene synthesis and subcloning into plasmid RT112. The resulting mutants were tested for their susceptibility to DOR and other NNRTIs in MT4‐GFP cells to assess potential cross‐resistance. The relative replication capacity of the mutants was measured by mixing various ratios of wild‐type (WT) and mutant infected cells. The resulting cultures were incubated for four weeks with medium change every three to four days. At each passage, supernatant was harvested for clonal sequencing analysis to quantitate the relative abundance of WT and mutant viruses.

**Results: ** Seven of 747 (0.9%) participants developed NNRTI resistance‐associated mutations from 2 DOR phase 3 clinical trials (Table 1). SDMs were generated for the substitutions Y188L, V106I/F227C, A98G/F227C, V106I/H221Y/F227C, A98G/V106I/H221Y/F227C, V106A/P225H/Y318F, and V106M/F227C and their susceptibility to NNRTIs was evaluated. Most of the mutants conferred high level of resistance to DOR with a fold change (FC) >100 (FC: mutantEC50 vs. WTEC50). Among the seven mutants, V106I/F227C, V106I/H221Y/F227C, V106M/F227C, and Y188L mutants displayed FC <10 against etravirine and rilpivirine, which is consistent with the phenotypic data from Monogram Biosciences. In addition, mutants containing F227C substitution were shown to be hypersensitive to some NRTIs such as AZT, TDF/TAF, and d4T. The replication capacity (RC) of Y188L, V106I/F227C, and A98G/V106I/H221Y/F227C was <10% of WT virus and the RC of A98G/F227C and V106M/F227C was approximately 20% of WT virus.

**Conclusions: **The majority of DOR‐selected viruses identified in the treatment‐naive participants in clinical trials to date may retain susceptibility to etravirine and hypersensitivity to some NRTIs with low replication capacity.

**Abstract THPDB0101‐Table 1. Doravirine‐selected NNRTI resistance in treatment‐naïve participants in clinical trials)**

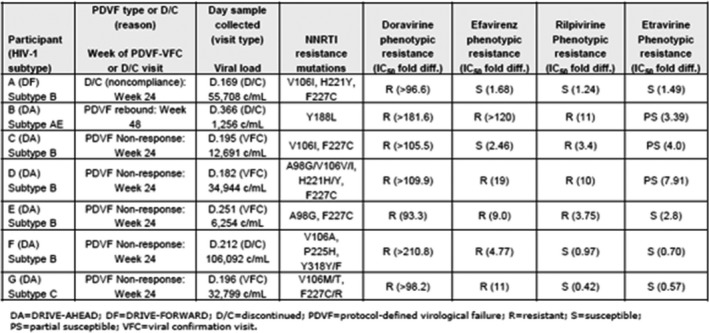

## THPDB0102

### Every site counts: Detecting low frequency variants in non‐subtype B HIV‐1 integrase associated with drug resistance in Uganda

**M. Avino^1^; E. Ndashimye^1,2^; D. Lizotte^1^; F. Kyeyune^2^; I. Nankya^2^; R. Gibson^1^; E. Nabulime^2^; C. Kityo^2^; P. Mugyenyi^2^; M. Quiñones‐Mateu^3^; E. Arts^1^ and A. Poon^1^**

^1^Western University, London, Canada, ^2^Joint Clinical Research Centre/Case Western Reserve University Center for AIDS Research, Kampala, Uganda, ^3^Case Western Reserve University, Cleveland, United States

**Background:** Next‐generation (deep) sequencing provides a sensitive and cost‐effective assay for low‐frequency variants in diverse HIV‐1 infections, but historically has been underutilized for non‐subtype B HIV‐1 infections in resource‐limited settings. Here, we use deep sequencing to analyze samples from treatment‐naïve individuals and individuals experiencing virological failure on combination antiretroviral treatment in Uganda. Our objective was to detect associations between low‐frequency mutations in HIV‐1 integrase and treatment outcomes in Uganda.

**Methods:** We retrieved a total of 362 archived plasma samples from patients at the Joint Clinical Research Centre (Kampala) with non‐B infections, of which 85 were treatment‐naive and 277 had experienced virological failure (VF) on first‐ (N = 129), second‐line (N = 116) or raltegravir (RAL)‐based (N = 32) regimens. For each sample, we extracted HIV‐1 plasma RNA and generated amplicon libraries for two overlapping regions spanning HIV‐1 integrase for sequencing on an Illumina MiSeq. Sequencing reads were iteratively aligned with bowtie2 and subtypes were classified with SCUEAL. Amino acid presence/absence matrices were generated at a 1% frequency cutoff and multiple imputations (n = 50) were analyzed by L1‐norm support vector machine (SVM) classification with five‐fold cross‐validation.

**Results: ** Overall, HIV‐1 subtype A (47%) was the most frequent, followed by subtype D (21%). More importantly, we detected several polymorphisms associated with integrase inhibitor resistance (e.g. E138K, G140A, Y143R, S147G, Q148K) in a small number of VF samples, although none of these polymorphisms were significantly associated with treatment outcomes. Our SVM analysis determined that the mutations T93A and V126M were the most strongly associated with first‐line VF; T174A and K211T with second‐line VF; and V165I and V151I with RAL‐based VF.

**Conclusions: **Detecting minority HIV‐1 variants with deep sequencing is important in settings where patients frequently discontinue treatment following VF, often leading to reversion to wild‐type genotype by the follow‐up visit. Our method describes a general strategy for detecting potential associations between the residual polymorphisms and treatment outcomes.

## THPDB0103

### Phenotypic assays of 5 integrase inhibitors on HIV‐2 clinical isolates reveal a new resistance pathway

**Q. Le Hingrat^1^; G. Collin^1^; G. Peytavin^2^; B. Visseaux^1^; M. Bertine^1^; R. Tubiana^3^; M. Karmochkine^4^; N. Valin^5^; A. Lemaignen^6^; L. Bernard^6^; F. Damond^1^; S. Matheron^7^; D. Descamps^1^; C. Charpentier^1^ and ANRS CO5 HIV‐2 Cohort**

^1^IAME, UMR 1137, INSERM, Université Paris Diderot, Sorbonne Paris Cité, AP‐HP, Laboratoire de Virologie, Hôpital Bichat, AP‐HP, Paris, France, ^2^IAME, UMR 1137, INSERM, Université Paris Diderot, Sorbonne Paris Cité, AP‐HP, Laboratoire de Pharmacologie, Hôpital Bichat, AP‐HP, Paris, France, ^3^Department of Infectious Diseases, AP‐HP Hôpital Pitié‐Salpêtrière, Paris. Sorbonne Universités, UPMC Univ Paris 06, INSERM, Institut Pierre Louis d'Epidémiologie et de Santé Publique (IPLESP UMRS 1136), Paris, France, ^4^Hôpital Européen Georges Pompidou, Service d'Immunologie Clinique, Paris, France, ^5^Hôpital St‐Antoine, Service de Maladies Infectieuses et Tropicales, Paris, France, ^6^CHU Tours, Service de Maladies Infectieuses et Tropicales, Tours, France, ^7^IAME, UMR 1137, INSERM, Université Paris Diderot, Sorbonne Paris Cité, AP‐HP, Service de Maladies Infectieuses et Tropicales, Hôpital Bichat, AP‐HP, Paris, France

**Background:** Integrase strand‐transfer inhibitors (INSTI) represent an important therapeutic option in HIV‐2‐infected patients for whom the number of active ARV is limited. Efficiency of INSTI has rarely been determined on HIV‐2 clinical isolates. We aimed to assess phenotypic susceptibility to five INSTI on integrase clinical isolates.

**Methods:** Phenotypic susceptibility assays were performed on 10 HIV‐2 isolates (3 group A and 7 group B) and the ROD reference strain using peripheral blood mononuclear cells method for bictegravir (BIC), cabotegravir (CAB), dolutegravir (DTG), elvitegravir (EVG) and raltegravir (RAL). Viruses were cultured without antiretroviral and with five ten‐fold dilutions of drug, ranging from 1000 to 0.1 nmol/L. At days 3 or 4, supernatant was withdrawn to assess viral replication (Biocentric HIV‐2 RNA^®^). Phenotypic susceptibility was expressed in fold‐changes of the IC50 between the isolate and the HIV‐2 ROD reference strain.

**Results: ** Ten clinical isolates were obtained from patients included in the ANRS CO5 HIV‐2 cohort, exhibiting virological failure (VF) under an INSTI‐based regimen (RAL = 6, EVG = 1 and DTG = 3). Five isolates displayed integrase resistance‐associated mutations (RAM) (97A+143G, 143R, 92Q+97A, 92Q+97A+155H and 140S+148). Five had no RAM but four of them presented a similar 5 amino‐acids insertion in the integrase N‐terminal region (S/Y‐R‐E‐G‐R/K), selected under RAL‐based regimen.

On the 5 isolates with RAM, BIC and DTG exhibited lower fold‐changes than RAL and EVG. On the 92Q+97A and 140S+148H combinations, BIC had lower fold‐changes than DTG (1.4 and 35‐fold for BIC vs. 10 and >300‐fold for DTG, respectively).

Regarding the four isolates with integrase insertion, they all presented high fold‐changes to RAL and EVG ranging from 20 to >300‐fold and intermediate fold‐changes to DTG and CAB ranging from 3 to 17‐fold and 11 to 58‐fold, respectively. Fold‐changes of BIC on those isolates were unmodified (0.4 and 1.1‐fold) or moderate (three and five‐fold).

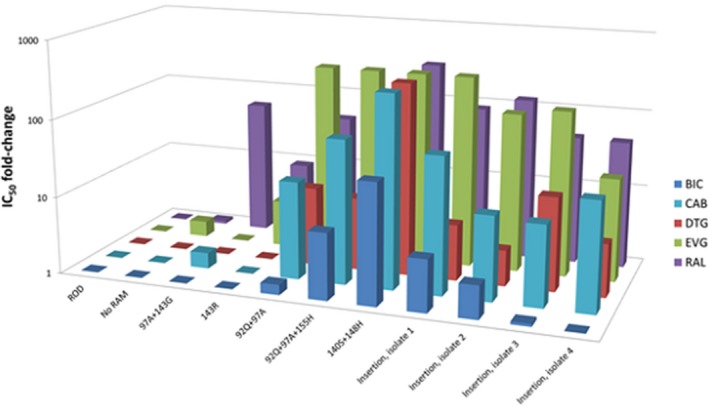

**Abstract THPDB0103‐Figure 1. Phenotypic susceptibility of HIV‐2 isolates to integrase inhibitors For each isolate, RAM in the integrase region are indicated.**

**Conclusions: **We describe for the first‐time, a new INSTI‐resistance pathway in HIV‐2‐infected patients with an insertion of 5 amino‐acids in the integrase. This insertion, selected under RAL‐based regimen, severely impacts RAL and EVG but might also compromises CAB, DTG and BIC susceptibility. Phenotypic susceptibility to BIC was less impacted than other INSTI by the presence of INSTI‐RAM.

## THPDB0104

### Accumulation of mutations in vivo confer cross‐resistance to new integrase inhibitors

**W.W. Zhang^1^; P.K. Cheung^2^; N.S. Oliveira^2^; M. Robbins^2^; P.R. Harrigan^3^ and A. Shahid^2^**

^1^University of British Columbia, Medicine, Vancouver, Canada, ^2^British Columbia Centre for Excellence in HIV/AIDS, Vancouver, Canada, ^3^University of British Columbia, Vancouver, Canada

**Background:** Bictegravir (BIC) and cabotegravir (CAB) are novel HIV integrase inhibitors currently in clinical trials. Descriptions of clinically relevant resistance to these newest inhibitors are still relatively limited. Dolutegravir (DTG) response in vivo in the VIKING study was reduced in patient viruses with Q148H±G140S and/or additional mutations, with two‐fold increases in IC50 considered clinically relevant. Reduced in vitro susceptibility to viruses with these mutations has also been observed with BIC and CAB. Here, we compare the phenotypic susceptibility to all five available HIV integrase inhibitors of a panel of fourteen viruses derived from patients having integrase inhibitor resistance.

**Methods:** Initially clonal recombinant viruses were produced by PCR amplification under conditions where single copies of integrase were amplified. This was followed by co‐transfection of integrase amplicons and linearized integrase‐deleted pNL4.3 plasmid into CEM‐GXR cells. Subsequent titering and phenotyping were performed in MT4‐LTR‐EGFP cells, where infectivity data was collected using a Spectra‐max i3 Minimax 300 microplate reader. Recombinant viruses were grown under a range of concentrations of raltegravir, elvitegravir, DTG, BIC and CAB. EC50 fold‐changes (FC) relative to a NL4.3 control were determined on day 3 post‐infection.

**Results: ** Viruses with the combination of G140S and Q148H substitutions alone had >100‐fold increases in EC50 to raltegravir and elvitegravir, but relatively small changes (two to four‐fold) in DTG, BIC or CAB susceptibility (Table 1). Viruses with progressively more substitutions showed extensive high level cross resistance to all five drugs (increases >50‐fold). Viruses with T97A and L74M substitutions exhibited six‐fold greater increases in IC50 (67 to 456‐fold change to DTG, BIC or CAB) compared to viruses which had only a T97A substitution (11 to 80‐fold change). Phenotypic resistance values were strongly correlated between DTG, BIC, and CAB, with correlation coefficients ranging from 0.96 to 0.98.

**Conclusions: **Accumulation of multiple mutations in HIV integrase led to high level phenotypic resistance to all five HIV integrase inhibitors in patient‐derived samples. Increases in phenotypic resistance values for DTG, BIC and CAB were almost co‐linear.

**Abstract THPDB0104‐Table 1. Median Fold Change in EC50 (IQR) of recombinant viruses with G140S and Q148H mutations and additional mutations for RAL, EVG, DTG, BIC and CAB**

**Table:** 

Key Mutations (Stanford HIV DB)	G140S + Q148H	G140S + Q148H	G140S + Q148H
Additional mutations	‐	+ T97A	+ T97A + L74M
N(patients)	6	3	3
n(viruses)	7	3	4
RAL	>100 (47‐>100)	>50 (>50‐>50)	>50 (>50‐>50)
EVG	>100 (>100 to >100)	>100 (>100 to >100)	>100 (>100 to >100)
DTG	3.5 (2.7 to 8.5)	33 (16 to 54)	417 (345 to 563)
BIC	2. 7 (2.1 to 3.1)	11 (7.0 to 15)	67 (65 to 81)
CAB	3.7 (3.2 to 4.5)	80 (55 to 111)	456 (279 to 522)

## THPDB0105

### Baseline resistance testing in the current treatment era – no longer cost‐effective?

**E. Hyle^1^; J. Scott^1^; P. Sax^2^; L. Millham^1^; T. Hou^1^; M. Weinstein^3^; K. Freedberg^1^ and R. Walensky^1^**

^1^Massachusetts General Hospital, Boston, United States, ^2^Brigham and Women's Hospital, Boston, United States, ^3^Harvard T.H. Chan School of Public Health, Boston, United States

**Background:** For people newly diagnosed with HIV, US guidelines recommend standard genotype testing to detect transmitted resistance to NNRTI, NRTI, and PIs, but not INSTIs. With INSTI‐based regimens as preferred first‐line therapy, results of a standard genotype at HIV diagnosis will influence only second‐line ART selection for people who experience an adverse event (AE) on INSTIs and require an ART switch.

**Methods:** We used the Cost‐effectiveness of Preventing AIDS Complications (CEPAC) model to examine the value of standard genotype at HIV diagnosis for people starting dolutegravir, comparing: (1) *No Genotype* and (2) *Genotype*. In both strategies (Table), patients with an AE to dolutegravir switch to rilpivirine, except those with known NNRTI‐resistance in *Genotype* who instead transition to darunavir. In *No Genotype*, patients with undiagnosed NNRTI‐resistance have lower viral suppression with second‐line rilpivirine. In both strategies, patients with virologic failure then have genotypes to guide subsequent ART. Standard genotype costs $350; ART costs $3000 to 3700/month. In sensitivity analysis, we varied the prevalence of transmitted NNRTI‐resistance (base case, 8%), prevalence of AEs on dolutegravir (base case, 14%), and suppression of NNRTI‐resistant virus with rilpivirine. Model outcomes (discounted 3%/year) included quality‐adjusted life years (QALYs), costs, and incremental cost‐effectiveness ratios (ICERs). We considered ICERs< $100,000/QALY cost‐effective.

**Abstract THPDB0105‐Table 1. Strategy‐specific response to adverse events on first‐line dolutegravir**

**Table:** 

	No Genotype		Genotype	
	NNRTI‐susceptible	NNRTI‐resistant	NNRTI‐susceptible	NNRTI‐resistant
First‐line ART	Dolutegravir		Dolutegravir	
48‐week suppression	93%		93%	
Monthly cost	$3700		$3700	
Second‐line ART	Rilpivirine	Rilpivirine	Rilpivirine	Darunavir
48‐week suppression	83%	37%	83%	83%
Monthly cost	$3000	$3000	$3000	$3700

**Results: ** Among all newly‐diagnosed patients, *No Genotype* resulted in 15.3043 QALYs and cost $730,240/person; G*enotype* gained 0.0003 QALYs and cost $850/person more (ICER, $2.8 million/QALY gained). Among patients with transmitted NNRTI‐resistance, *Genotype* resulted in 0.0039 additional QALYs compared to *No Genotype* and cost $6,590/person more (ICER, $1.6 million/QALY gained). At base case assumptions, 1.1% of newly diagnosed people with HIV would benefit clinically from *Genotype*, but it would cost $114,510 to test 100 patients for a maximum gain of 2.6 quality‐adjusted days for one person. *Genotype* was not cost‐effective compared to *No Genotype* unless prevalence of transmitted NNRTI‐resistance >40% and AEs on dolutegravir >40%, with no suppression of NNRTI‐resistant virus on rilpivirine‐based ART (Figure 1).

**Conclusions: **With INSTI‐based regimens as first‐line treatment in the US, the standard genotype test at HIV diagnosis offers minimal clinical benefit, is more expensive, and is not cost‐effective. Practice guidelines should consider removing genotypes from the recommended baseline evaluation.

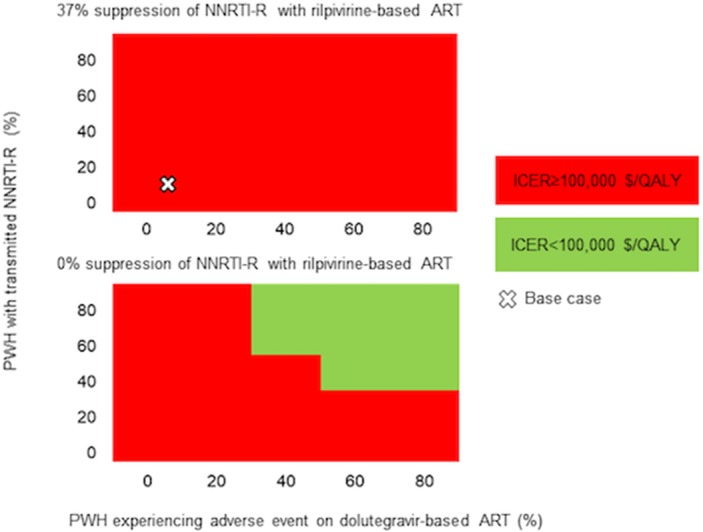

**Abstract THPDB0105‐Figure 1.**

## THPDC0101

### Dispensing HIV self‐tests in pharmacies in France: the pharmacists’ point of view

**T. Greacen; A. Simon; A. Troisoeufs and K. Champenois**

EPS Maison Blanche, Laboratoire de Recherche, Paris, France

**Background:** HIV self‐test kits have been available in pharmacies and on pharmacy websites in France since September 2015. What are the principle obstacles and facilitators that pharmacists themselves have encountered providing self‐tests and information and support for self‐test clients?

**Methods:** From February to December 2016, 22 interviews were conducted with pharmacists in three high HIV prevalence areas (a gay neighbourhood in central Paris; Guadeloupe/French Guyana; Paris suburbs with large sub‐Saharan migrant communities) and one low HIV prevalence area in Northern France.

**Results: ** Although pharmacists saw HIV self‐test provision in pharmacies as a step forward with regard to engaging their profession in a key public health issue in France, most professionals interviewed had sold relatively few self‐tests, and significantly less than expected. The major concern of pharmacists was the risk associated with individuals discovering positive test results alone at home and the issue of linkage to care. The current price of self‐test kits was generally considered to be too high, even for pharmacists making minimal profit. Discretion and anonymity were clearly major issues. Few clients actually asked any direct questions. Clients interested in HIV self‐tests were generally not their habitual clientele. A number of pharmacists reported clients purchasing two tests at a time, and hypothesized that this might be for their partners. The positioning of the HIV self‐test kits in the pharmacy was a key indicator of pharmacists’ attitudes with regard to self‐testing. Although the law specifically states that the HIV self‐test should be kept behind the counter, some pharmacists provided off‐the‐shelf access to ensure convenience and privacy to clients. In other pharmacies, typically in gay areas in central Paris, the self‐test kits were stored behind the counter but visible to the public, with the aim of facilitating discussion with clients. Finally, in a not insignificant number of cases, no self‐test kits or information on HIV self‐testing were visible at all.

**Conclusions: **Although pharmacists see HIV self‐testing as part of a significant trend in France towards facilitating access to screening for health conditions in general, price, anonymity, discretion and linkage to care remain crucial issues.

## THPDC0102

### Linkage to HIV care following HIV self‐testing: a cluster randomised trial of community‐based distribution of oral HIV self‐test kits nested in four HPTN 071 communities in Zambia

**S. Floyd^1^; K. Shanaube^2^; A. Schaap^1,2^; M. Phiri^2^; B. Hensen^1^; C. Mulubwa^2^; V. Bond^1,2^; B. Chiti^2^; M. Simwinga^2^; R. Hayes^1^; S. Fidler^3^; A. Mwinga^2^ and H. Ayles^1,2^**

^1^London School of Hygiene & Tropical Medicine, London, United Kingdom, ^2^ZAMBART, Lusaka, Zambia, ^3^Imperial College, London, United Kingdom

**Background:** HIV self‐testing (HIVST) has the potential to help achieve the UNAIDS 90‐90‐90 targets. However, evidence is limited about how to ensure linkage to care (LTC) among individuals with HIV‐positive results. We report LTC findings from a HIVST cluster‐randomised trial nested within the HPTN071 (PopART) trial in Zambia. The PopART intervention was delivered in “annual rounds” from 1 December 2013 to 31 December 2017, during which community‐HIV‐care‐providers (CHiPs) visited all households, and offered home‐based HIV testing with a rapid diagnostic test using fingerprick blood (RDT), referral to routine clinic services, and support (including follow‐up visits) for LTC.

**Methods:** In 4 December 2016 of the PopART intervention communities in Zambia, comprising 66 zones, were included in a cluster‐randomised trial of adding oral HIVST to the standard intervention. Self‐testing was offered in‐person, supervised or unsupervised, and to absent partners via secondary distribution. We estimated the time from CHiP referral to LTC among individuals who were (re‐)enumerated as a household member during 1 February 2017 to 30 April 2017, aged ≥16 years, and diagnosed HIV‐positive based on initial or confirmatory (following HIVST) RDT. We used the Kaplan‐Meier method for “time‐to‐event” analysis, and follow‐up information to 30/9/2017.

**Results: ** Among 13,267 individuals in 33 HIVST zones, 195 were diagnosed HIV‐positive; additionally 20 tested HIV‐positive with supervised/unsupervised self‐testing but did not have confirmatory RDT, and 13 tested HIV‐positive following secondary distribution but were not contacted in‐person by CHiPs. Among 13,706 individuals in 33 non‐HIVST zones, 204 were diagnosed HIV‐positive. Among those diagnosed, 94% (184/195) in HIVST and 98% (199/204) in non‐HIVST zones were referred to care. We estimated that 65% in HIVST, and 64% in non‐HIVST, zones were LTC by three months after referral (hazard ratio 1.11, 95% CI 0.78 to 1.58; Figure 1, Table 1). In HIVST zones, there was a suggestion that LTC was slower for individuals who tested with unsupervised self‐testing or via secondary distribution, compared with those who tested with RDT (Table 1).

**Conclusions: **LTC following an HIV‐positive diagnosis and CHiP referral was not undermined by offering HIVST as a testing option, in the context of LTC support. Strategies to facilitate confirmatory RDT following an initial HIVST, and LTC following unsupervised self‐testing and secondary distribution, may be important.

**Abstract THPDC0102‐Table 1. Time to link to HIV care after CHiP referral, by whether individual was referred from a HIVST or non‐HIVST zone**

**Table:** 

	Referred to HIV care (n/N, %)	Linked to HIV care, by months after referral (%)			
1	1	1	3	6	Hazard ratio, 95% confidence interval
Overall					
Non‐HIVST	199/204, 98%	50.3	63.8	76.6	1 (ref)
HIVST	184/195, 94%	52.6	64.8	78.2	1.11 (0.78 to 1.58)
Overall, HIVST zones					
RDT	85/88, 97%	51.2	71.4	78.9	1 (ref)
Supervised	80/86, 93%	57.5	68.1	80.8	1.04 (0.66 to 1.63)
Unsupervised	14/16, 87%	45.6	45.6	45.6	0.47 (0.19 to 1.14)
Secondary distribution	5/5, 100%	25.0	25.0	25.0	0.24 (0.03 to 1.76)
“Minimum estimate” of linkage to HIV care					
Non‐HIVST	199/204, 98%		82/199 41.2%		
HIVST	184/195, 94%		82/184 44.6%

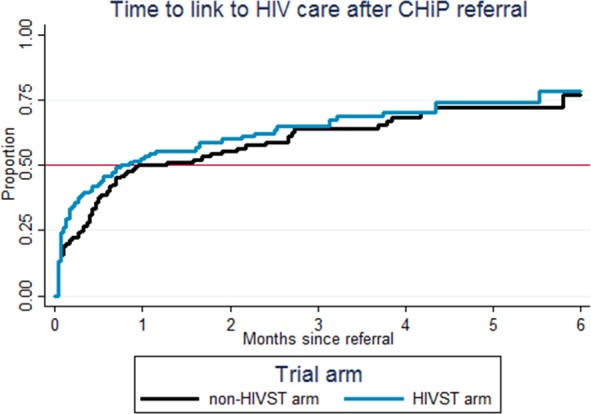

**Abstract THPDC0102‐Figure 1. Time to link to HIV care after CHiP referral, among individuals who were diagnosed HIV‐positive by CHiPs).**

## THPDC0103

### Increasing knowledge of HIV status and demand for antiretroviral therapy using community‐based HIV self‐testing in rural communities: a cluster randomised trial in Malawi

**P. Indravudh^1,2^; K. Fielding^1^; M. Neuman^1^; R. Chilongosi^3^; P. Mkandawire^3^; E. Nyondo^3^; R. Nzawa^2^; L. Magombo^3^; D. Chalira^2^; M. Kumwenda^2^; R. Nyirenda^4^; C. Johnson^5^; C. Nkhoma^3^; N. Desmond^2,6^; K. Hatzold^7^ and E.L. Corbett^1,2^**

^1^London School of Hygiene and Tropical Medicine, London, United Kingdom, ^2^Malawi‐Liverpool‐Wellcome Trust Clinical Research Programme, Blantyre, Malawi, ^3^Population Services International, Blantyre, Malawi, ^4^Ministry of Health, Lilongwe, Malawi, ^5^World Health Organisation, Geneva, Switzerland, ^6^Liverpool School of Tropical Medicine, Liverpool, United Kingdom, ^7^Population Services International, Washington, United States

**Background:** HIV self‐testing (HIVST) has potential to reach populations poorly served by facility‐based HIV testing services. We used a cluster‐randomised trial design to investigate the impact of community‐based HIVST distribution on recent HIV testing and antiretroviral therapy (ART) uptake in rural Malawi.

**Methods:** Government clinics (n = 22) and their defined rural catchment areas were allocated using restricted 1:1 randomisation to either (i) door‐to‐door distribution of HIVST kits by resident community‐based distributors (CBD) or (ii) the standard of care (SOC). Distributors provided continuous HIVST access and option of post‐test support and assisted referral to routine confirmatory testing and ART services. Social harm monitoring was also established.

The primary outcome compared recent HIV testing (previous 12 months) across arms, ascertained through population‐based surveys conducted 12 months after the cluster start date in pre‐defined evaluation villages. Analysis used logistic regression with adjustment for imbalance between arms. For the secondary outcome, we used generalized estimating equations to analyse cluster‐level ART initiations that were recorded in clinic registers in the 12 months after cluster enrolment, adjusting for ART initiations in the preceding 12 months.

**Results: ** A total of 83 CBDs delivered 79,349 HIVST kits over a 12 to 15‐month period, with three reported social harms. Of 5504 adults in the post‐intervention survey, 42.6% were men and 15.4% were adolescents aged 16 to 19 years. Coverage was significantly higher in the HIVST than SOC clusters for both recent testing (64.1% vs. 45.6%, adjusted risk ratio (aRR) 1.38, 95% CI 1.14 to 1.68) and lifetime testing (87.3% vs. 78.7%, aRR 1.12, 95% CI 1.05 to 1.16). Differences between arms were more pronounced for adolescents (aRR 1.99, 95% CI 1.35 to 2.92) and men (aRR 1.55, 95% CI 1.19 to 2.01).

Among 93,640 adults living in the defined study area, the proportion of ART initiations per 1000 adult clinic population increased in the HIVST versus SOC arm in the intervention period, adjusting for pre‐intervention ART uptake (adjusted initiation risk ratio 1.36, 95% CI 0.95 to 1.94, *p* = 0.09).

**Conclusions: **CBD‐delivered HIVST increased HIV testing coverage in rural populations, especially among men and adolescents, and population‐level demand for ART. This approach can rapidly improve knowledge of HIV status in underserved populations and have a measurable impact on ART uptake.

**Abstract THPDC0103‐Table 1. (Effect of community‐based HIVST self‐testing on testing coverage and ART demand) (inlinegraphic)**

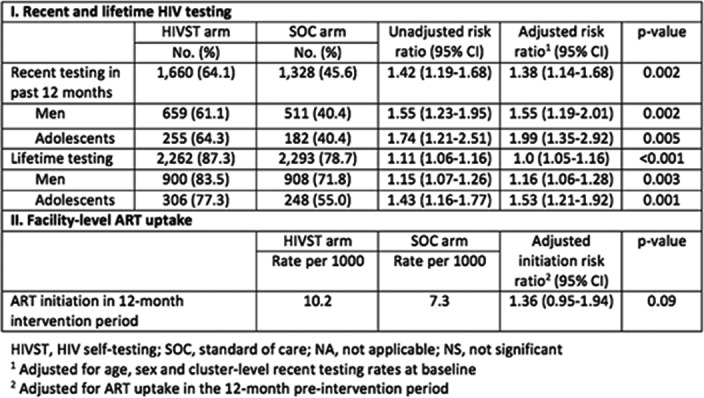

## THPDC0104

### Secondary distribution of HIV self‐tests as a way to promote HIV testing among male partners of young women: subgroup analysis from a randomized trial

**K. Agot^1^; B. Obonyo^1^; J. Ambia^1^; G.‐N. Wango^2^ and H. Thirumurthy^3^**

^1^Impact Research and Development Organization, Kisumu, Kenya, ^2^Nyanza Initiative for Girls’ Education and Empowerment, Kisumu, Kenya, ^3^University of Pennsylvania, Department of Medical Ethics and Health Policy, Philadelphia, United States

**Background:** HIV risk among young women in eastern and southern Africa remains extremely high and age‐disparate sexual relationships are widely believed to be a contributing factor. Interventions that promote HIV testing among male partners of young women are essential for reducing HIV risk. Given compelling evidence on the acceptability of HIV self‐testing (HIVST), we assessed whether provision of multiple self‐tests to young women can result in higher male partner testing.

**Methods:** This sub‐study analyzed data among a subgroup of young women aged 18 to 24 years who participated in a larger randomized trial conducted at clinics in Kisumu, Kenya (NCT02386215). The trial enrolled women seeking antenatal and postpartum care and randomized them to receive two HIV self‐tests (HIVST group) or a comparison group in which invitation cards were given to encourage clinic‐based HIV testing. Women in the HIVST group also received a brief demonstration of how to use self‐tests along with pictorial use instructions. Follow‐up interviews were conducted with women at three months to assess whether partner and couples testing occurred. The primary outcome was partner testing and the secondary outcome was couples testing. Logistic regression analyses were used to compare outcomes in the two study groups.

**Results: ** Of 599 women enrolled in the trial in 2015, 367 (61.2%) were aged 18 to 24 years. Eighty‐eight percent of the young women were married. A total of 179 and 188 women were randomized to the HIVST and comparison groups, respectively. Follow‐up interviews were completed by 347 women (94.5%). Male partner testing uptake was 92.4% in the HIVST group and 55.7% in the comparison group (odds ratio 9.7, 95% CI 5.1 to 18.3). Couples testing was also significantly more likely in the HIVST group than the comparison group (77.8% vs. 38.1%, odds ratio 5.7, 95% CI 3.6 to 9.1).

**Conclusions: **Provision of multiple HIV self‐tests to young women seeking pregnant and postpartum care was very effective in increasing male partner and couples testing. Although not generalizable to unmarried young women, the findings suggest that HIVST can play a prominent role in facilitating testing among their male partners. As countries begin to scale‐up HIVST, further investigation of secondary distribution interventions among young women is warranted.

## THPDC0105

### Effective promotion of HIV Self‐testing among MSM in Russia in the context of growing stigma and discrimination

**E. Pisemskiy**

Orlovskaya Regional Non‐Profit Organization against AIDS Phoenix PLUS ^NGO Phoenix PLUS^; Orel, Russian Federation

**Background:** This project provided for free and easy access for gays and other MSM and TG to self‐testing for HIV. They could get a free self‐testing kit in various places: community organizations, friendly clinics, friendly commercial pharmacies, clubs and saunas, as well as by limited courier delivery and from volunteers. The project was implemented in five cities

The project answers three major questions:

(1) How to increase testing coverage?

(2) What methods of self‐testing kits distribution are the most convenient for MSM and TG and are cost effective for an NGO?

(3)How can we make MSM and TG come to AIDS centers for confirmation testing and linkage to care?

**Methods:** Throughout the project, users could contact the organizers via the free 8 to 800 hotline or a friendly doctor at the AIDS center (whose business card was included into the self‐test kit) or a peer consultant by the phone and any social media. When receiving a self‐testing kit, the users were also encouraged to give feedback on the gaytest.info web‐site using an individual code.

**Results: ** The project supported by EJAF allowed to perform roughly the same number of HIV tests among MSM as five large projects supported by the Global Fund in 5 Russian cities.

Over 10,000 tests were conducted within a year. Self‐reports show that 28% of users leave feedback on the result of the test, 15% of them reporting a reactive result. As a result of case management, 230 confirmation tests were performed in AIDS centers (registered) and 186 people were enrolled in care at AIDS centers.

**Conclusions: **The HIV self‐testing project significantly increases testing coverage of MSM and TG. Since most MSM do not turn to community organizations for services, self‐testing allows to reach the groups of MSM and TG people that were not covered by testing programs earlier. We recommend self‐testing as a method that can be effectively used for such key populations as MSM, SW and DU even under high levels of stigma and discrimination.

## THPDC0106

### An intervention to teach young MSM and transgender women of color how to HIV self‐test with a friend: Lessons learned in project TRUST

**M.Q. Paige^1^; L. Wilton^2^; D. Lucy^1^; G. Ortiz^1^; V. Nandi^1^; B. Koblin^1^ and V. Frye^1,3^**

^1^New York Blood Center, Project ACHIEVE, New York, United States, ^2^Binghamton University, Binghamton, United States, ^3^City University of New York, New York, United States

**Background:** Increasing consistent HIV testing among high‐risk populations such as men who have sex with men (MSM) and transgender women (TW) can lead to earlier treatment and uptake of PEP/PrEP, reducing HIV transmission. HIV self‐testing is a relatively novel method that may increase consistent testing by reducing the travel and time burdens, HIV stigma, and/or increasing client control.

**Description:** TRUST tested a behavioral intervention to increase consistent HIV self‐testing among Black/African‐American and Latino MSM and TW aged 18 to 34. The experimental arm randomized friend pairs to taking 4^th^ generation HIV rapid tests together and a 30‐minute, facilitated session designed to: increase motivation to test consistently, master HIV self‐testing skills, and commit to consistent HIV testing. Here we describe participant experiences of the experimental arm.

**Lessons learned:** Correct knowledge of HIV transmission is fundamental to increasing testing motivation, yet many participants had low knowledge (e.g. the “window period,” status certainty, PEP/PrEP, etc.), indicating that HIV/sex education is lacking in primary/secondary US education. HIV testing is an anxiety‐producing experience, with stigma an important driver of testing fear. “TRUST” was appropriately named, as participants chose the study friend based primarily on whether they “trusted” them. Increasing trust within the friend pair was facilitated by concrete discussion of testing plans, specifying and practicing support acts, and integrating familiarity and levity into testing. Participants reported high levels of satisfaction with the session, particularly learning how to self‐test and with a friend. Increased self‐efficacy and control were reported and attributed to mastering test mechanics and the realization that participants could control the setting, timing of and people present via self‐testing. This last finding was consistent with the theoretical bases for the intervention, which emphasized self‐determination and agentic control.

**Conclusions/Next steps:** Teaching friend pairs of MSM and TW of color to HIV self‐test may increase consistent HIV testing among this higher risk group. Our experimental arm implementation assessment suggests that the intervention hit many of the theoretical targets and was highly acceptable to participants. Results of the trial will determine if the experimental arm outperformed the control arm on study outcomes.

## THPDD0101

### Social‐structural correlates of HIV stigma among women living with HIV in Metro Vancouver

**K. Deering^1,2^; C. Logie^3^; A. Krusi^4^; F. Ranville^2^; M. Braschel^2^; P. Duff^4^; K. Shannon^2,4^ and on Behalf of the SHAWNA Project (Sexual Health, HIV/AIDS: Women's Longitudinal Needs Assessment)**

^1^University of British Columbia, Vancouver, Canada, ^2^Gender and Sexual Health Initiative, Vancouver, Canada, ^3^University of Toronto, Actor‐Inwentash Faculty of Social Work, Toronto, Canada, ^4^University of British Columbia, Medicine, Vancouver, Canada

**Background:** HIV stigma is widely known as a substantial barrier to access to and use of HIV treatment and care services. Less is known about the social and structural factors that produce HIV stigma. The objective of this study was therefore to understand the social and structural correlates of HIV stigma among women living with HIV (WLWH) in Vancouver, Canada.

**Methods:** Data were drawn from two years of follow‐up from a longitudinal community‐based participatory open cohort of 318 cis or trans WLWH who lived and/or accessed care in Metro Vancouver, Canada (2014‐present)(Sexual Health and HIV/AIDS: Women′s Longitudinal Needs Assessment “*SHAWNA*”). Participants completed semi‐annual interviewer‐administered questionnaires by trained peer researchers/community interviewers and clinical questions by a sexual health research nurse. Stigma outcomes included three dimensions:

(1) Disclosure Concerns;

(2) Personalized Stigma;

(3) Internalized Stigma; and an additional question measuring

(4) Social Attitudes Stigma.

Sexual orientation, gender identity, immigrant status and ethnic/racial identity were all measured at the initial interview; remaining social‐structural variables were time‐updated and measured in the last six months, including stigma outcomes. Bivariate and multivariable linear regression using generalized estimating equations for repeated measures were used to examine correlates of the four stigma measures.

**Results: ** Overall, 215 women responded to ≥1 follow‐up survey with 509 total observations. In multivariable analysis, HIV disclosure without consent was significantly associated with Disclosure Concerns (estimate: 0.84, 95% CIs: 0.30 to 1.37; *p* = 0.002); Personalized Stigma (estimate: 2.17, 95% CIs: 1.35 to 2.98; *p* < 0.001); Internalized Stigma (estimate: 1.04, 95% CIs: 0.31 to 1.76; *p* = 0.005); and Social Attitudes Stigma (estimate: 0.44, 95% CIs: 0.11 to 0.76; *p* = 0.008). Time since first diagnosed with HIV (estimate: −0.04 *p*/year, 95% CIs: −0.08 to −0.01; *p* = 0.016) was negatively associated with Disclosure Concerns. Physical violence by any perpetrator (estimate: 0.90, 95% CIs: 0.13 to 1.67; *p* = 0.022); poor treatment by health professionals (estimate: 0.98, 95% CIs: 0.10 to 1.86; *p* = 0.030); and physical/verbal violence associated with the participant's HIV‐positive status (estimate: 1.43, 95% CIs: 0.49 to 2.38; *p* = 0.003) were associated with Personalized Stigma. Physical violence from any perpetrator (estimate: 0.41, 95% CIs: 0.15 to 0.68; *p* = 0.002); and physical/verbal violence associated with participant's HIV‐positive status (estimate: 0.38, 95% CIs: 0.11 to 0.66; *p* = 0.006) were significantly associated with Social Attitudes Stigma.

**Conclusions: **Study results strongly suggest a critical need to develop strategies to address social and structural violence against WLWH, including amending Canada's restrictive HIV disclosure laws as a structural intervention to reduce HIV stigma and promote safe disclosure for WLWH.

## THPDD0102

### HIV‐related stigma and discrimination among health care personnel in Thailand: results of the 2017 national surveillance survey

**K. Srithanaviboonchai^1,2^; P. Khemngern^3^; N. Pudpong^4^; J. Chueayen^1^ and T. Siraprapasiri^3^**

^1^Chiang Mai University, Research Institute for Health Sciences, Chiang Mai, Thailand, ^2^Chiang Mai University, Faculty of Medicine, Chiang Mai, Thailand, ^3^Ministry of Public Health, Department of Disease Control, Nonthaburi, Thailand, ^4^International Health Policy Program (IHPP), Nonthaburi, Thailand

**Background:** HIV‐related stigma and discrimination (S&D) is a major obstacle in the attempt to end the AIDS epidemic. Stigma and discrimination that occur within health facilities are of particular concern. To monitor this issue, Thailand developed a national surveillance system to monitor S&D in government health facilities.

**Methods:** Thirteen provinces, 12 provinces that serve as the centers for Thailand′s 12 health regions as well as the capital city Bangkok, were selected as national surveillance sites. All government hospitals with antiretroviral treatment clinics in each province served as survey venues. Both health staff and supportive staff providing services directly to the patients (regardless of patient's HIV status) were eligible to participate in the survey. The sample size at each health facility was determined proportional to the size of health staff of the whole province. Simple random sampling was used to identify potential participants. The participants completed a standardized questionnaire capturing actionable drivers and manifestations of HIV‐related S&D in health care facilities online using a smartphone or tablet. The composite indicators, defined as the percentage of positive responses with at least one question within a particular domain, were computed and reported as the main outcomes.

**Results: ** Of the 2615 participants, 78.2% were females and 51.2% were professional health staff. The average age was 38.6 years old. The most frequently reported composite indicators of the drivers of HIV‐related S&D were negative attitudes toward people living with HIV (PLHIV) (82.1%), followed by fear of acquiring HIV while caring for PLHIV (50.5%). The most frequently reported composite indicators of the manifestations of stigma were over‐protecting oneself while caring for PLHIV (56.7%), followed by observed discrimination towards PLHIV during the last 12 months (25.8%). Compared to non‐professional staff, professional staff were more fearful acquiring HIV while caring for PLHIV (OR = 1.53; 95% CI 1.24 to 1.88), but had fewer negative attitudes toward PLHIV (OR = 0.50; 95% CI 0.40 to 0.63).

**Conclusions: **The survey provided evidence of HIV‐related S&D in Thai health facilities. The information could be used as an advocacy tool for policy change and to tailor stigma reducing interventions at the local level.

## THPDD0103

### Socio‐structural protection from internalized HIV stigma among South African adolescents living with HIV: the potential of clinic‐community collaborations for stigma reduction

**M. Pantelic^1,2^; M. Moshabela^3^; E. Toska^2,4^; L. Cluver^2,4^; G. Caswell^5^ and A. Ronan^6^**

^1^International HIV/AIDS Alliance, Secretariat, Brighton, United Kingdom, ^2^University of Oxford, Department of Social Policy and Intervention, Oxford, United Kingdom, ^3^University of KwaZulu‐Natal, Durban, South Africa, ^4^Cape Town University, Cape Town, South Africa, ^5^International HIV/AIDS Alliance, Cape Town, South Africa, ^6^Pediatric‐Adolescent Treatment Africa, Cape Town, South Africa

**Background:** Southern Africa is home to 2 million adolescents living with HIV (ALHIV), who struggle to adhere to HIV treatment and care partly due to exceptionally high levels of self‐stigma. South Africa's new National Strategic Plan HIV, TB and STIs includes a key objective to halve HIV‐related self‐stigma by 2022 but there is no evidence of scalable interventions to achieve this. This study examined protective factors in both clinics and communities that could reduce self‐stigma among ALHIV.

**Methods:** Total population sampling of ALHIV (aged 10 to 19) from 53 public health facilities in the Eastern Cape, South Africa was used. Self‐stigma was measured via the adolescents living with HIV stigma scale (ALHIV‐SS). Community protection was measured via adolescent report of

(1) no experiences of discrimination and

(2) no perceived stigma in the community.

Clinic protection was measured via five key adolescent‐reported indicators:

(1) No past‐year ART stockouts,

(2) Flexible clinic appointments that prevented excessive school truancy,

(3) Adolescent‐sensitive healthcare providers,

(4) Perceived data confidentiality and

(5) Access to a regular HIV support group.

A multivariate logistic regression tested associations between clinic and community protection and self‐stigma controlling for age, gender and knowledge of HIV status. A marginal effects model tested potential additive effects of combining clinic and community protection.

**Results: ** 90.1% of eligible ALHIV were interviewed (n = 1060, 55% female, mean age  = 13.8, 21% living in rural locations and 67% vertically infected). Prevalence of self‐stigma was 26.5%. At the community level, protection from discrimination (OR:.38; CI:.22‐.63) and non‐stigmatizing perceptions (OR:.40; CI:.29‐.64) decreased odds of self‐stigma. At the clinic level, reliable ART stocks (OR:.40; CI:.23‐.72), flexible appointment times (OR:.78; CI:.50‐.93) and kind healthcare providers (OR:.58; CI:.41‐.93) decreased odds of self‐stigma among ALHIV. Age, gender, HIV status awareness, clinic confidentiality and support group access were not associated with self‐stigma. Prevalence of self‐stigma dropped from 85.8% among ALHIV without clinic or community protection to 12.9% among ALHIV with both clinic and community protection (Figure 1).

**Conclusions: **Findings suggest that a combination of clinic and community interventions hold promise for adolescent‐centred HIV care. Self‐stigma among ALHIV can be substantially reduced by addressing stigma in communities and strengthening health systems.

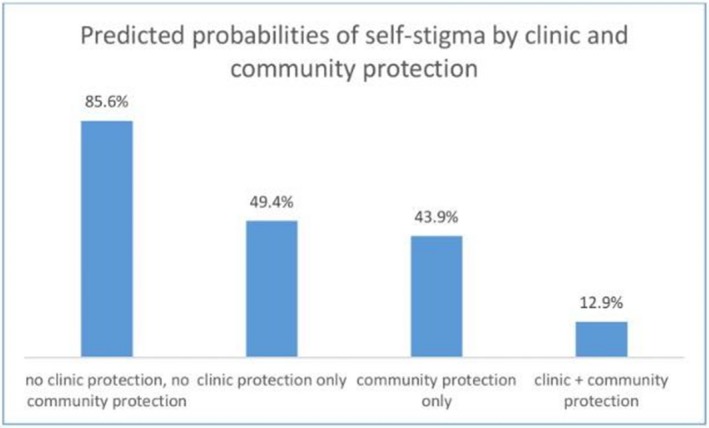

**Abstract THPDD0103‐Figure 1.**

## THPDD0104

### Pathways from sexual stigma to incident HIV and sexually transmitted infections among Nigerian MSM

**E. Okporo^1^; C. Rodrguez‐Hart^2^ and TRUST Study Group**

^1^Hyacinth AIDS Foundation, Prevention, Jersey, United States, ^2^New York State Department of Health and Mental Hygiene, New York, United States

**Background:** Sexual stigma is the co‐occurrence of the following four components within a power imbalance: labeling, stereotyping, separation, and status loss and discrimination specific to sexual minorities because of same‐sex practices. Although sexual stigma has been found to be associated with HIV prevalence and with avoidance of seeking health care, it remains unknown whether sexual stigma drives onward transmission of HIV and sexually transmitted infections (STIs) among Nigerian MSM. Sexual stigma has also been found to be associated with condom less sex among MSM across a variety of settings outside of Nigeria.

**Methods:** The Network‐Based Recruitment of MSM into HCT, Care, Treatment and Prevention Services at Trusted Community‐based Venues (TRUST/RV368) study utilizes respondent‐driven sampling to recruit MSM into a prospective cohort at ICARH site in Abuja Nigeria, Eligibility criteria included male sex assigned at birth and at least 16 years of age in Abuja. total of 1480 participants.

**Results: ** The sample consisted of participants who were primarily under 25 years of age (60%), had completed high school or less education (70%), identified their sex as male (82%), had never disclosed their same‐sex practices to a family member (83%), engaged in both insertive and receptive anal sex with male partners in the past 12 months (53%), and approximately half had a female sex partner in the past 12 months. Bivariate analysis revealed that in creasing sexual stigma was associated with increasing incident HIV and/or STI infections in a dose‐response association (low: 10.6%, medium: 14.2%, high 19.0%, P¼0.008.

**Conclusions: **The path analysis model revealed that MSM who were in higher stigma classes at baseline were more likely to contract HIV/STIs over the course of the study, and this was partially explained by stigma's association with suicidal ideation, suicidal ideation's association with condomless sex with casual sex partners, and condomless sex's association with HIV/STI acquisition. Therefore, our findings highlight the need to incorporate mental health issues of MSM in HIV and STI programming in Nigeria. One option would be to adopt WHO's Mental Health Gap Action Programme intervention.

## THPDD0105

### Project on HIV destigmatization in the marriage equality movement in Taiwan

**S.‐C. Du; C.‐L. Peng and M.‐Y. Juan**

Taiwan Tongzhi ^LGBTQ+^ Hotline Association, Taipei City, Taiwan, Province of China

**Background:** In 2016 to 2017, the marriage equality debate was heated in Taiwan, and certain anti‐LGBTQ+ groups have used HIV‐related issues to attack the LGBTQ+ community, spreading false information about LGBTQ+ and HIV/AIDS via mass and social media. Gay and bisexual men, especially those living with HIV/AIDS (PLWHA), have become a major target of rumors and slanders. This presentation aims to introduce how Taiwan Tongzhi (LGBTQ+) Hotline Association tackled the HIV stigma incurred in the marriage equality movement, and how we destigmatize the disease and reflect on the issue.

**Description:** This project adopts two strategies of destigmatizing HIV while promoting marriage equality:

1 Analyzing and responding to false information regarding LGBTQ+ and HIV/AIDS: We designed an easy‐to‐understand version of “HIV/AIDS for Dummies,” and have distributed the correct information to the general public via social media.

2 HIV+OK Campaign: We have invited people to express their support for PLWHA with actions through organizing public events, campaigning on social media, and taking actions on Taiwan LGBT Pride Parade.

**Lessons learned:** The “HIV/AIDS for Dummies” has attracted 107,353 views and public discussions. Our targeted audience is not limited to the LGBTQ+ community but also their parents and LGBTQ+‐friendly heterosexuals. Meanwhile, the HIV+OK Campaign has encouraged people to express their support for PLWHA. More than 30 people, including PLWHA, their family members and friends shared their stories. More than 100 people joined Hotline's procession in Taiwan LGBT Pride Parade that promoted the concept of “HIV+OK,” and more than 2000 social media users changed their profile picture to openly show their support.

**Conclusions/Next steps:** Examining the implementation and outcomes of this project, we found that:

(1) This project is the first time for LGBTQ+ group to initiate large‐scale, broad‐scope public dialogues about HIV related issues, not just focusing on LGBTQ+ community.

(1) There is an obvious lack of anti‐discrimination HIV/AIDS education that targets adults in Taiwan, enabling the false information about HIV/AIDS to be spread rapidly. We urge that the government devote more state resources to this issue.

(3) The fight for equal rights for PLWHA cannot be independent from LGBTQ+ movements and the marriage equality movement. They have to ally with one another to move forward.

## THPDD0106

### Stigma as a barrier to obtaining quality VCT services: monitoring outcomes from the Zaporizhzhia region of Ukraine

**R. Milevskyi^1^; O. Vytvitskyi^1^; D. Kalinin^1^; S. Maltabar^1^; Y. Radchenko^2^ and O. German^2^**

^1^Gender Z, Zaporizhzhia, Ukraine, ^2^ECOM, Tallinn, Estonia

**Background:** Due to the pervasiveness of homophobia and transphobia in Ukrainian society, we decided to research the prevalence of stigma towards MSM and LGBT people by health professionals and NGO social workers providing VCT services in the Zaporizhzhia region. Additionally, we examined providers’ awareness levels in regards to the specifics of MSM/LGBT counselling.

**Description:** With support from ECOM, we piloted an advocacy project, which included the monitoring of VCT providers in both NGO and public health care settings. The program also included training elements, as well as the development and regional‐level approval of an Algorithm and practical recommendations for the consultation of MSM/LGBT people. During field research our “test clients” visited 95 service centers to assess their comprehensiveness and suitability, as well as providers’ tolerance and professionalism upon disclosures of non‐heterosexual sexual orientations and practices.

Based on our findings, we organized a training on SOGI and MSM/LGBT counselling for 100 representatives from every VCT point in the region.

After having jointly analysed the problems that we uncovered with local authorities, the Zaporizhzhia Department of Health adopted an Algorithm for counselling MSM and LGBT people and approved our recommendations on the prevention of stigma and discrimination in the provision of health care services.

**Lessons learned:** The monitoring visits revealed a range of shortcomings and gaps in the health care sector, including: inadequate accessibility to HIV testing, violations of VCT principles, homophobia and transphobia, and a widespread lack of knowledge of key populations’ issues.

Our follow‐up advocacy activities were designed to decrease the number of rights violations experienced by MSM/LGBT people accessing health care in our region and to reduce the overall level of stigma and discrimination.

The project confirmed that:

(1) Provider‐based stigma and inadequate knowledge of LGBT issues remain salient barriers to obtaining quality services;

(2) The initiative and meaningful involvement of affected communities is essential in order to promote any positive changes.

**Conclusions/Next steps:** Raising tolerance levels towards MSM/LGBT significantly decreases stigmatization within professional circles and, subsequently, helps fight the HIV epidemic. Our positive experience using the methodology of “test clients” suggests that this research method can be applied to future projects aimed at other key populations.

## THPDD0201

### Multi‐level barriers to antiretroviral therapy initiation, retention, and adherence for female sex workers living with HIV in South Africa

**C. Comins^1^; L. Parmley^1^; S. Schwartz^1^; H.T. Mkhize^2^; V. Guddera^2^; D.R. Phetlhu^3^; K. Young^4^; H. Hausler^4^ and S. Baral^1^**

^1^Johns Hopkins Bloomberg School of Public Health, Key Populations Program, Center for Public Health and Human Rights, Department of Epidemiology, Baltimore, United States, ^2^TB/HIV Care, Durban, South Africa, ^3^University of Western Cape, School of Nursing, Cape Town, South Africa, ^4^TB/HIV Care, Cape Town, South Africa

**Background:** South Africa has a generalized HIV epidemic that disproportionately affects female sex workers (FSW). FSW experience individual, network, and structural barriers to initiation and sustained engagement in antiretroviral therapy (ART). Here, we use a modified socio‐ecological model (SEM) to map ART initiation, retention and adherence barriers among FSW in South Africa.

**Methods:** FSW living with HIV (n = 24) and key informants (n = 15) participated in semi‐structured, in‐depth interviews from September‐November 2017 in Durban, South Africa. FSW participants were sampled using maximum‐variation sampling, ensuring variations in sex work and ART experiences. Key informants included providers at various levels, brothel managers, and police. Qualitative data were coded using a grounded theory approach in Atlas.ti 8. Matrices were used as a post‐coding technique to map emergent themes at each level of the SEM including individual, network, community, and public policy levels.

**Results: ** Each level of the SEM presented specific barriers to initiation, retention, and adherence for FSW living with HIV (Figure 1). Diagnosis prior to universal test and treat, lack of clinic card, and incarceration were policy‐level challenges among FSW and related to ART initiation, retention and adherence, respectively. Initiation and retention in care was affected at all levels, with emphasis on individual‐level barriers, while network‐ and individual‐ level barriers predominately affected ART adherence. At the network‐ and community‐ levels, FSW experienced unique barriers generally and based on their sex work venue (indoor vs. outdoor) and primary work time (nighttime vs. daytime) respectively. FSW identified no community‐level barriers related to ART adherence.

**Conclusions: **While ART is well known to improve clinical outcomes, and decrease onward HIV transmission, these data highlight the sustained barriers to ART initiation and retention among FSW living with HIV in South Africa. Moving forward necessitates tailored yet scalable interventions to address level‐specific barriers among FSW and ultimately optimize sustained treatment outcomes.

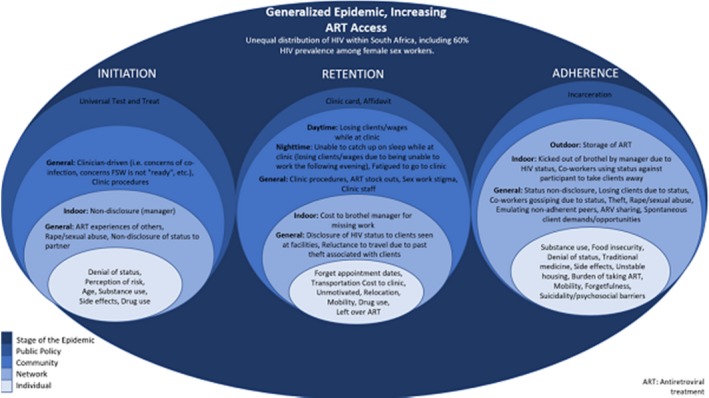

**Abstract THPDD0201‐Figure 1. Challenges engaging in HIV Treatment among female sex workers living with HIV in Durban, South Africa).**

## THPDD0202

### *“I wish I had more help to be able to control myself with drugs”*: Experiences with substance use among female sex workers living with HIV in the Dominican Republic

**J. Knight; H. Gomez; M. Pérez; Y. Donastorg; D. Kerrigan and C. Barrington**

Instituto Dermatológico de Cirugía y Piel, Unidad de Vacunas e Investigación, Santo Domingo, Dominican Republic

**Background:** Substance use, including alcohol and illicit drugs, is prevalent among female sex workers (FSW) and the social environments in which they work. In Santo Domingo, nearly half report consuming alcohol before sex and one‐quarter have ever used drugs. Substance use is negatively associated with adherence to anti‐retroviral therapy (ART), protected sex, and viral suppression. To inform tailored strategies related to substance use, we explored in‐depth substance use experiences among FSW living with HIV and assessed how they impact HIV care and treatment.

**Methods:** We recruited 40 FSW living with HIV among participants of a multi‐level intervention called *Abriendo Puertas* (Opening Doors) for qualitative in‐depth interviews in 2016. Interviews were audio‐recorded and transcribed in Spanish. We used a combination of narrative and inductive thematic analysis in Nvivo11.

**Results: ** We identified three main analytical domains: (1) contexts in which substance use occurred, (2) attitudes and perceptions of use, and (3) impact on HIV care. Women reported consuming substances socially with peers, to better entertain clients, and to help cope with the stresses and stigma of both sex work and HIV. Most women who reported high levels of consumption did not view these behaviors as problematic or as negatively impacting their health, including HIV. Yet, some did report forgetting or discontinuing ARTs and HIV appointments after using. Awareness of support services for alcohol and drug dependence was low, but interest in such services was high, as reflected in the quote in the title.

**Conclusions: **Substance use is a central part of women's experiences with sex work and the social environments in which they work and is a major barrier to ART adherence and retention in care. Greater capacity of HIV care providers to detect substance use among FSW and integration of substance use counseling and support services into HIV care models is urgently needed to improve HIV outcomes in this population. We propose a potential model after successfully piloting a substance use module targeting FSW living with HIV in 9 HIV care facilities, which involved provider training to detect drug dependence, a brief intervention, and referrals to support facilities.

## THPDD0203

### “They must understand us as sex workers”: health service perspectives among female sex workers in the context of PrEP and early ART introduction in the TAPS Demonstration Project, South Africa

**R. Eakle^1^; A. Bourne^2^; R. Bothma^1^; G. Gomez^3^; F. Venter^1^ and H. Rees^1^**

^1^Wits Reproductive Health and HIV Institute, Johannesburg, South Africa, ^2^LaTrobe University, Melbourne, Australia, ^3^London School of Hygiene and Tropical Medicine, London, United Kingdom

**Background:** Female sex workers (FSWs) are a key population and could greatly benefit from HIV prevention methods such as pre‐exposure prophylaxis (PrEP). However, FSWs often experience difficulties accessing health services which could pose a barrier to PrEP uptake and retention. We explored previous health service experiences and suggestions for best practices with FSWs in focus group discussions (FGDs) as part of designing the TAPS Demonstration Project.

**Methods:** FGDs were conducted in Johannesburg and Pretoria, and examined opportunities and barriers for safe and efficient PrEP delivery within the context of TAPS. Sex worker peer educators recruited participants through social networks using snowball sampling. Facilitation was in English with adaptation by facilitators into local languages as needed. Transcripts were translated and transcribed into English. Data were subject to a thematic analysis.

**Results: ** Four FGDs were conducted in each of the two sites engaging 69 participants, ages 20 to 60. Overwhelmingly, participants voiced concerns about stigma and negative treatment of FSW in public health facilities. This was seen as a major potential barrier to successful provision of PrEP and early ART as FSWs would not attend clinics where they had been negatively treated. “Feeling free” to openly discuss health issues, such as burst condoms, STIs, or even sexual assault, was critically important, as was accessibility. Some FSWs felt that getting to the clinic when they lived some distance away would be challenging for monthly clinic visits. Mobile delivery was highly recommended. Consistency and flexibility in service provision (e.g. aligning clinic times, regular mobile clinics) were recurring themes as part of effective, quality care. Peer‐driven education, navigation, and service were highlighted as an important best practice.

**Conclusions: **Service provision sensitised and tailored to sex workers needs will be critical to successful delivery of PrEP and early ART. Stigmatization of sex workers in clinic environments is well‐documented, yet little has been published from FSW perspectives about how to address these issues. Involving FSWs will help to build relevant services especially when implementing new interventions.

## THPDD0204

### Occupational barriers to antiretroviral therapy adherence, sources of support, and coping strategies for female sex workers living with HIV in South Africa

**L. Parmley^1^; C. Comins^1^; S. Baral^1^; H.T. Mkhize^2^; V. Guddera^2^; D.R. Phetlhu^3^; K. Young^4^; H. Hausler^4^ and S. Schwartz^1^**

^1^Johns Hopkins Bloomberg School of Public Health, Center for Public Health and Human Rights, Baltimore, United States, ^2^TB/HIV Care, Durban, South Africa, ^3^University of Western Cape, School of Nursing, Cape Town, South Africa, ^4^TB/HIV Care, Cape Town, South Africa

**Background:** Despite advances in antiretroviral therapy (ART) globally, achieving sustained viral suppression among persons living with HIV continues to be a major challenge in the HIV response. Social and structural barriers impede ART adherence and are heightened among marginalized and key populations including female sex workers (FSW) in South Africa. The objective of these analyses is to characterize FSW‐specific barriers and facilitators to ART adherence affecting clinical outcomes and HIV prevention goals.

**Methods:** Semi‐structured, in‐depth and key informant interviews were conducted with 24 FSW living with HIV and 15 key informants in Durban, South Africa from September‐November 2017. FSW and key informants were recruited using maximum‐variation and snowball sampling respectively. FSW were recruited on key variants including: type of sex work venue, primary work time, and ART‐naivety and use. Data collection and analysis were iterative; transcripts were coded and analyzed using grounded theory in Atlas.ti 8.

**Results: ** Multiple relevant themes were reported that included sex work venues dismissing FSW known to be living with HIV based on ART use, theft of clinic cards and ART by clients, and concerns of wage loss if HIV status were disclosed to clients, colleagues, and pimps/managers either by being seen at an HIV clinic or by carrying ART. Occupational pressures including short term or seasonal migration for work further challenged engagement in treatment programs while pressures to use drugs and alcohol from clients or the use of similar substances to de‐sensitize themselves challenged adherence to ART. Support systems to optimize ART included FSW receiving HIV treatment support from peers. In addition, many FSW reported individual coping strategies to overcome adherence barriers including sharing and purchasing ART from peers, planning for spontaneous client demands and approaches to facilitate taking medications at work while limiting unwanted disclosure of status.

**Conclusions: **Taken together, these data highlight the occupational barriers to accessing and taking ART among FSW living with HIV. While coping strategies included social cohesion among FSW, ART sharing and purchasing may be associated with suboptimal treatment outcomes. Considering these occupational pressures on FSW is important when designing and implementing HIV treatment programs to support sustained ART engagement.

## THPDD0205

### The sociability of risk: sex work, criminalization and HIV/AIDS in Kampala, Uganda

**S. Cruz**

University of Amsterdam, Research Center for Gender & Sexuality, Amsterdam, Netherlands

**Background:** In Kampala, Uganda women engaging in “high risk” sexual practices, like commercial sex work, have an estimated 33% to 37% HIV seroprevalence (Vandepitte et al. 2011; Hladik et al. 2017). In response to this statistic I set out to investigate how women manage daily risks associated with sex work, criminalization, and HIV/AIDS.

**Methods:** Primary ethnographic data collection occurred over fifteen months within two Kampala Divisions (Rubaga and Makindye), involving participant observation, group discussions, and informal, unstructured interviews with female commercial sex workers. Research insights then informed policy analysis.

**Results: ** The study reveals that women's social networks have the greatest impact on their ability to manage daily risks. However, three important variables intervene with women's risk management:1)the sex work environment,2)divergence in women's social relationships, and3)the current policy approach guiding HIV healthcare.

The study reveals that a woman's physical sex work environment, such as the room she rents in a brothel, impacts her ability to safeguard private property (e.g. condoms, cash, medications, clothing, shoes, etc.). In turn, a woman with fewer personal belongings is perceived by other women in the brothel as less capable of minimizing risks, such as police abuse, arrest, client violence, and/or contracting HIV, and therefore less deserving of social relationships in the brothel. Alternatively, women deemed more capable of maintaining their social ties are far more likely to develop cash reserves in order to purchase medications, improve their brothel working and living conditions, receive and retain condom supplies, and/or frequently travel outside the brothel to receive healthcare and/or improve their commercial sex work prospects. Data furthermore reveals how current HIV interventions, prioritizing individualized behavior, undermine women's social resources (i.e. their social networks and social capital). The study documents women's struggles to adhere to recommended HIV/AIDS treatment protocols when they believe they must choose between their individualized HIV healthcare plan and demands from their social network (i.e. the social practices minimizing daily risks associated with sex work and criminalization).

**Conclusions: **In conclusion, this research underscores the sociability of women's HIV risk, risk management, and the implications of these social processes on current HIV/AIDS prevention and treatment protocols.

## THPDE0101

### Integrated youth‐friendly health services lead to substantial improvements in uptake of HIV testing, condoms, and hormonal contraception among adolescent girls and young women in Malawi

**N. Rosenberg^1^; N. Bhushan^1^; D. Vansia^2^; T. Phanga^2^; B. Maseko^2^; A. Kachigamba^3^; J. Tang^1^; M. Hosseinipour^1,2^; A. Pettifor^1^ and L.‐G. Bekker^4^**

^1^University of North Carolina at Chapel Hill, Chapel Hill, United States, ^2^University of North Carolina Project, Lilongwe, Malawi, ^3^District Health Office, Lilongwe, Malawi, ^4^Desmond Tutu HIV Foundation, Cape Town, South Africa

**Background:** Adolescent girls and young women (AGYW) in sub‐Saharan Africa (SSA) experience high incidence of HIV, pregnancy, and sexually transmitted infections (STIs), but face numerous barriers to HIV and sexual and reproductive health (SRH) care‐seeking. In this analysis, we assessed whether a model of integrated youth‐friendly health services (YFHS) for AGYW led to increased uptake of condoms, HIV testing, and hormonal contraception, compared to standard of care (SOC).

**Methods:** Through the Girl Power study, four comparable public sector health centers were selected in Lilongwe, Malawi and randomly assigned to either the SOC or YFHS. The SOC offered vertical HIV testing, STI management, and family planning in three separate areas with providers who received no additional training. The other three health centers offered YFHS, which consisted of these same services in an integrated fashion, in youth‐dedicated spaces, with peers and providers trained in youth‐friendly approaches. In each health center, AGYW 15 to 24 years old were enrolled and followed for one year for uptake and frequency of HIV testing, condoms, and hormonal contraception. The SOC and YFHS models were compared using adjusted risk differences and incidence rate ratios and ninety‐five percent confidence intervals.

**Results: ** One thousand AGYW enrolled (N = 250/health center). Median age was 19 years (inter‐quartile range 17 to 21 years). Compared to AGYW in the SOC health center, those in the YFHS health centers were 23% (CI: 17% to 29%) more likely to ever receive HIV testing, 57% (CI: 51% to 63%) more likely to ever receive condoms, and 39% (CI: 34% to 45%) more likely to ever receive hormonal contraception. Compared to AGYW in the SOC, AGYW in the YFHS models accessed HIV testing 2.4 (CI: 1.9 to 2.9) times more often, condoms 7.9 (CI: 6.0 to 10.5) times more often, and hormonal contraception 6.0 (CI: 4.2 to 8.7) times more often. Each of the three YFHS health centers performed better than the SOC on each indicator.

**Conclusions: **In public sector health centers an integrated model of YFHS that included brief provider training and modest clinical modifications lead to considerably higher SRH and HIV service utilization for AGYW. Implementation science is needed to guide scale‐up of this highly promising service delivery model.

**Abstract THPDE0101‐Table 1. Comparison of the SOC Clinic (Model 1) to the YFHS Clinics (Models 2 to 4)**

**Table:** 

	Proportion of Participants Who Ever Received the Service Over One Year				Mean Number of Times Service Received Per Participant Per Year			
	SOC (N = 250)	YFHS (N = 750)	Adjusted Risk Difference*	*p*‐value	SOC (N = 250	YFHS (N = 750)	Adjusted Incidence Rate* Ratio	*p*‐value
Condoms‡	26%	83%	57%	<0.001	0.3	2.3	7.9	<0.001
HIV Testing†	72%	97%	23%	<0.001	1.1	2.7	2.4	<0.001
Hormonal Contraception§	10%	54%	39%	<0.001	0.2	1.0	6.0	<0.001

*All models control for age, number of children, and marital status measured at baseline. Some models also controlled for baseline HIV testing†, condom use‡, and hormonal contraceptive use§

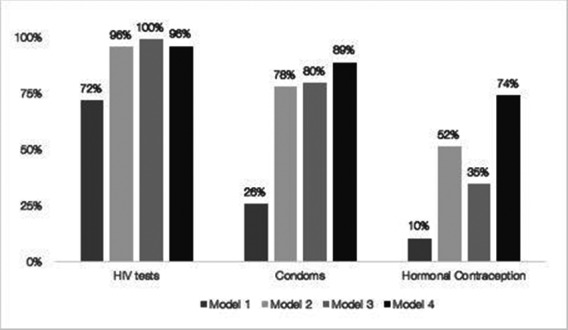

**Abstract THPDE0101‐Figure 1. Proportion of AGYW who Received Each Service by Arm)**

## THPDE0102

### Meaningful engagement of schools and school‐based advocates in promoting education on HIV and supporting adolescents living with HIV in Kenya

**E. Ruria^1^; M. Sayo Muandale^2^; J. Odionyi^3^; J. Kose^3^; L. Matu^3^; E. Mwangi^3^; R. Otieno‐Masaba^3^; G. Woelk^4^ and N. Rakhmanina^4,5,6^**

^1^Elizabeth Glaser Pediatric AIDS Foundation, Homa Bay, Kenya, ^2^Ministry of Education, Homa Bay County, Homa Bay, Kenya, ^3^Elizabeth Glaser Pediatric AIDS Foundation, Nairobi, Kenya, ^4^Elizabeth Glaser Pediatric AIDS Foundation, Washington, United States, ^5^Children's National Health System, Washington, United States, ^6^George Washington University, Washington, United States

**Background:** Adolescents living with HIV (ALHIV) spend a significant proportion of their daily lives in schools. In 2016, with support from ViiV Healthcare, the Elizabeth Glaser Pediatric AIDS Foundation implemented the innovative peer‐designed fast‐track linkage‐to‐care and early retention Red Carpet Program (RCP) throughout public health care facilities (HCFs) and schools in Kenya. RCP worked closely with the Ministry of Education (MOE) and Ministry of Health (MOH) to design and implement school‐based interventions to promote HIV education and support for ALHIV. The aim of this analysis was to evaluate the implementation of RCP school‐based interventions conducted in 2017.

**Description:** RCP conducted two main interventions in the Homa Bay County boarding schools:(1)enhanced partnership between schools and 50 RCP HCFs;(2)built capacity of adolescent health advocates (counselling teachers, school matrons, nurses and boarding in‐charge managers).

A sensitization meeting was held with 70 secondary boarding school in‐charge managers, principals and MOE officials in August 2017. During August‐September 2017, RCP conducted a two‐day capacity building workshop for 90 school‐based adolescent health advocates from 44 high volume local secondary boarding schools.

**Lessons learned:** By November 2017, 50 boarding schools in Homa Bay County developed key HIV strategies including: a) fostering direct linkages with HCFs; b) formation of School Health Committees (SHC); c) training of adolescent health advocates; and d) support of ALHIV. By December 2017, 50 SHC were formed and >3000 students (both HIV+ and HIV‐) were reached with education on HIV, HIV stigma, antiretroviral treatment, pre‐exposure prophylaxis, and sexual and reproductive health. Fifty schools implemented activities targeting ALHIV, parent representatives and linked RCP HCFs teams. All 50 schools implemented adherence counselling, confidential storage and access to HIV medications for ALHIV.

**Conclusions/Next steps:** Schools provided daily support to ALHIV from accessing care, medications to retention in care. The uptake of the general HIV education and support services for ALHIV was high in schools of Homa Bay County, Kenya. Going forward, RCP works to expand collaboration between HCFs and schools with the goal to increase long‐term retention in care and on treatment, and achieve high levels of virologic suppression among ALHIV.

## THPDE0103

### Cash+Care: parenting support and violence reduction programme associated with reductions in adolescent HIV‐risks in South Africa: a cluster randomized trial of a DREAMS and 4Children‐implemented programme ‘Parenting for Lifelong Health’

**L. Cluver^1,2^; F. Meinck^1^; J. Doubt^1,3^; C. Ward^2^; C. Lombard^4,5^; Y. Shenderovich^1,6^; J. Steinert^1^; R. Herrero Romero^1^; S. Medley^1^; A. Redfern^1^; N. Salah^7^; S. De Stone^8^; L. Ncobo^9^; J. McLaren Lachman^1,9^; S. Tsoanyane^9^; H. Loening^3^; J. Byrne^3^; L. Sherr^10^; M. Casale^1,11^; F. Gardner^1^; C. Wittesaele^1^; R. Catanho^1^; S. Hoeksma^1^; C. Mikton^12^; J. van derWal^1^; M. Nocuza^1^; M. Pancoast^1,13^; M. Danisa^9^; I. Wessels^2,9^; N. Masuku‐Mukadah^9^; M. Boyes^14^; D. Nzima^15,16^ and N. Sibanda^17^**

^1^Oxford University, Department of Social Policy & Intervention, Oxford, United Kingdom, ^2^University of Cape Town, Department of Psychiatry and Mental Health, Cape Town, South Africa, ^3^UNICEF Innocenti Office of Research, Florence, Italy, ^4^South African Medical Research Council, Biostatistics Unit, Cape Town, South Africa, ^5^University of Cape Town, School of Public Health and Family Medicine, Cape Town, South Africa, ^6^University of Cambridge, Institute of Criminology, Cambridge, United Kingdom, ^7^London School of Hygiene & Tropical Medicine, London, United Kingdom, ^8^University of Warwick, Warwick Medical School, Warwick, United Kingdom, ^9^Clowns Without Borders South Africa, Durban, South Africa, ^10^University College London, London, United Kingdom, ^11^University of the Western Cape, School of Public Health and Family Medicine, Cape Town, South Africa, ^12^WHO, Department of Violence and Injury Prevention and Disability, Geneva, Switzerland, ^13^The Beans, San Francisco, United States, ^14^Curtin University, Faculty of Health Sciences, School of Psychology and Speech Pathology, Perth, Australia, ^15^University of Fort Hare, Department of Sociology & Anthropology, Alice, South Africa, ^16^Ali Douglas Research Network, Bulawayo, Zimbabwe, ^17^London School of Economics and Political Science, Department of International Development, London, United Kingdom

**Background:** Adolescent HIV‐risk behaviors are increased by family violence, low parental supervision, substance use and poverty. ‘Cash + care’ structural approaches can reduce adolescent HIV‐risks, but parenting a teenager is complex and challenging. WHO, UNICEF, USAID‐PEPFAR and academics developed and tested a parenting support and violence reduction program for low‐resource settings, to be used as part of structural prevention programs.

**Methods:** Pragmatic cluster randomized trial (n = 1100 participants, 40 clusters) in South Africa's rural and urban Eastern Cape. Adolescents and caregivers participated in 14 evidence‐based sessions

(i.e. conflict reduction, protection from sexual abuse in the community, family budgeting), implemented by local community members with NGOs Clowns Without Borders, REPSSI, UNICEF South Africa and the Department of Social Development. Analyses used intention‐to‐treat with hierarchical negative binomial or Poisson regression for counts and hierarchical linear mixed effects regression for continuous outcomes.

**Results: ** Retention was 97% at five to nine months post‐intervention. The intervention did not impact all parenting outcomes (i.e. neglect), but had significant impact on six key HIV risk reduction factors: lower family violence (caregiver report IRR 0.55 (95% CI 0.40 to 0.75, *p* < 0.001); improved involved parenting (caregiver report d = 0.86 (95% CI 0.64 to 1.08, *p* < 0.001; adolescent report d = 0.28 (95% CI 0.08 to 0.48, *p* = 0.006) and less poor supervision (caregiver report d = −0.50 (95% CI −0.70 to −0.29, *p* < 0.001; adolescent report d = −0.34 (95% CI −0.55 to −0.12, *p* = 0.002), and improved family economic welfare, including sustained food availability (caregiver report d = −0.62, 95% CI −0.84 to −0.40, *p* < 0.001; adolescent report d = −0.28, 95% CI −0.52 to −0.05, *p* = 0.017). It was also associated with lower alcohol and drug use amongst adolescents (IRR = 0.55, 95% CI 0.33 to 0.93, *p* = 0.026) and amongst caregivers (IRR = 0.67, 95% CI 0.49 to 0.99, *p* = 0.041), and with improved planning for protection against sexual predators (caregiver report d = 0.48, 95% CI 0.24 to 0.72, *p* < 0.001; adolescent report d = 0.33, 95% CI 0.06 to 0.59, *p* = 0.017).

**Conclusions: **This cluster RCT in South Africa shows that an intervention to support families can reduce direct risks for adolescent HIV‐acquisition: violence, low supervision, food insecurity and substance use. The programme is being adapted and scaled in eight countries in the region through DREAMS, 4Children, USAID, UNICEF and by national governments. Strengthening families may be an essential component of adolescent HIV prevention in Africa.

## THPDE0104

### Improving timely linkage to care among newly diagnosed HIV‐infected adolescents: Results of SMILE

**R. Miller^1^; D. Chiaramonte^1^; C. Banerjee^2^; D. Sharma^2^; J.D. Fortenberry^3^ and Adolescent Medicine Trials Network for HIV/AIDS Interventions**

^1^Michigan State University, Psychology, East Lansing, United States, ^2^Michigan State University, CSTAT, East Lansing, United States, ^3^University of Indiana, Adolescent Medicine, Indianapolis, United States

**Background:** In the U.S., youth are the least likely among people of all age groups to link to HIV medical care quickly following a positive HIV‐test result. Delayed linkage to care deprives youth of the benefits of HIV treatment and risks increased HIV transmission. Interventions to improve the rates of timely linkage to care for youth represent an urgent national priority. In 2009, we initiated the Strategic Multisite Initiative for Identification, Linkage and Engagement (SMILE) program to improve timely linkage to care among newly diagnosed HIV‐infected youth in eight urban U. S. adolescent medicine clinical trials units (AMTU). We deployed a dedicated linkage‐to‐care specialist to link youth to an infectious disease physician within 42 days of a positive test result. In 2013, we additionally pursued local organizational, institutional, community, and policy changes to address structural barriers to youth's timely access to care through coalitions convened by each AMTU.

**Methods:** We collected anonymized clinical patient records from 2008 to 2015. Data included demographics and dates of HIV testing and medical care linkage events for 1695 newly diagnosed HIV‐positive youth ages of 12 to 24. We plotted the linkage‐to‐care interval in days for each quarter from the start of 2008, prior to the start of SMILE, through 2015.

**Results: ** At the start of SMILE, the average number of days between HIV test result and linkage was 951. This reflects, in part, that youth who had HIV tests long before the program's initiation ultimately linked to care during its start‐up phase. By end of 2012, the average number of days to linkage had decreased to 55 (Figure 1). By the end of the initiative (2015), the average number of days to linkage had fallen to 16.

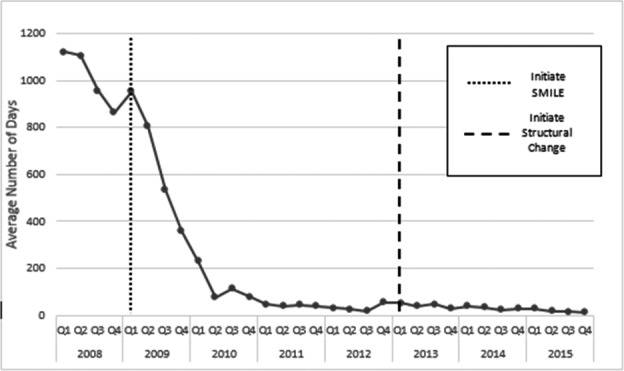

**Abstract THPDE0104‐Figure 1. Average number of days between HIV test and linkage‐to‐care.**

**Conclusions: **Integrated, multi‐level interventions that draw on existing community resources can dramatically improve timely linkage to care among high‐risk youth.

## THPDE0105

### 90‐90‐48: The reality of viral suppression among ART‐initiated adolescents in South Africa

**R. Haghighat^1^; L. Cluver^1^; N. Bungane^2^ and E. Toska^3^**

^1^University of Oxford, Social Policy and Intervention, Oxford, United Kingdom, ^2^Mzantsi Wakho, East London, South Africa, ^3^University of Cape Town, AIDS and Society Research Unit, Cape Town, South Africa

**Background:** Global fast‐track targets include 90% viral suppression among all ART‐initiated persons. Data suggests adolescents have worse viral suppression rates than children and adults, but little is known on adolescent progression through the HIV treatment cascade. This study examines the HIV treatment cascade for a large sample of HIV+ adolescents in South Africa.

**Methods:** 1058 ART‐initiated adolescents (10 to 19 years) from 52 urban and rural healthcare facilities in the Eastern Cape were interviewed (March 2014‐September 2015). Data were extracted from paper‐based medical records from all facilities (including records in multiple facilities) through January 2018. Predictors of progression through cascade were identified using sequential multivariate logistic regressions, with age (10 to 14/15 to 19 years), sex, urban/rural residence, mode of infection, decentralised/centralised ART care, and time on ART entered simultaneously. Interactive effects and moderating effects of gender and mode of infection were tested with regressions, corrected with the Benjamini‐Hochberg procedure.

**Results: ** 92.5% of adolescents had viral loads available in clinic files. 63.1% had viral loads recorded in the past two years. At most recent viral load, 78.5% of measurements were ≤1000 copies/mL, but only 58.8% were undetectable (≤50 copies/mL). Participants were female (54.0%), median age 13 years (IQR 11 to 16), urban‐living (76.8%); and 30.0% attended ≥2 healthcare facilities. Adolescents on ART for <2 years were more likely to lack viral loads from the past two years (OR 5.73 (95% CI 1.82 to 18.08), *p < *0.003), and decentralised care was protective only for females (OR 2.56 (95% CI 1.40 to 4.64), *p* = 0.002). Viral load >1000 copies/mL was associated with older age and rural‐living (OR 2.18 (95% CI 1.58 to 3.00), *p < *0.001; OR 1.48 (95% CI 1.06 to 2.12), *p* = 0.031). Older adolescents, those on ART for <2 years, and decentralised adolescents were less likely to have undetectable viral loads (OR 0.65 (95% CI 0.49 to 0.87), *p* = 0.003; OR 0.36 (95% CI 0.13 to 0.91), *p* = 0.047; OR 0.74 (95% CI 0.57 to 0.98), *p* = 0.035).

**Conclusions: **Viral suppression rates remain low among adolescents in South Africa. Older, recently initiated, and decentralised adolescents were least likely to be virally suppressed—potentially due to down‐referrals from tertiary paediatric facilities to generalised primary clinics. With 30% of adolescents receiving care in multiple facilities, interventions supporting patient linkages to care may be essential for adolescents transitioning across multiple forms of care.

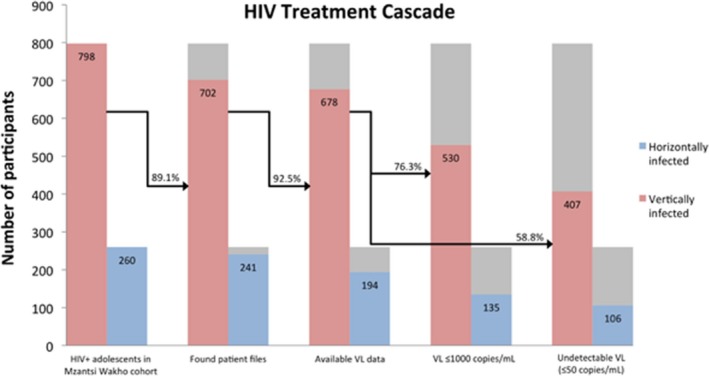

**Abstract THPDE0105‐Figure 1. HIV treatment cascade for ART‐initiated adolescents in the Eastern Cape, South Africa**

## THPDE0106

### A randomized, controlled trial of a patient‐centered disclosure counseling intervention for Kenyan children living with HIV

**R. Vreeman^1,2,3^; W. Nyandiko^2,3^; I. Marete^2,3^; A. Mwangi^3,4^; C. McAteer^1,3^; A. Keter^3,4^; M. Scanlon^3,5^; S. Ayaya^2,3^; J. Aluoch^3^ and J. Hogan^3,6^**

^1^Indiana University School of Medicine, Ryan White Center for Pediatric Infectious Disease and Global Health, Department of Pediatrics, Indianapolis, United States, ^2^College of Health Science, Moi University, Department of Child Health and Paediatrics, Eldoret, Kenya, ^3^Academic Model Providing Access to Healthcare, Eldoret, Kenya, ^4^College of Health Science, Moi University, Department of Behavioral Sciences, Eldoret, Kenya, ^5^University of Massachusetts Boston, John W McCormack Graduate School of Policy and Global Studies, Boston, United States, ^6^Brown University, Department of Biostatistics, Providence, United States

**Background:** For children living with HIV, learning about their HIV status (“disclosure”) is a critical process within their transition to adulthood. Caregivers of perinatally HIV‐infected children frequently worry about the impact of disclosure, while also reporting delayed disclosure can hurt medication adherence. We evaluated the impact of a patient‐centered, culturally‐ and age‐appropriate disclosure counseling intervention among Kenyan children and their caregivers.

**Methods:** We conducted a prospective, clinic‐cluster randomized trial in which we followed child‐caregiver dyads (children ages 10 to 14) attending eight clinics (randomized to intervention or control) at a large HIV treatment program in Kenya. All patients at the intervention clinics had access to intensive counseling (family, one‐on‐one, and peer group sessions) with trained disclosure counselors and culturally‐tailored materials, compared to control clinics with standard care. Disclosure was treated as a time‐to‐event outcome, measured on a discrete time scale, with assessments at 0, 6, 12, 18, and 24 months. Mental health and psychosocial outcomes were assessed using standardized questionnaires.

**Results: ** The 285 children were mean age 12.3 years, 52% female, with average time‐on‐treatment of 4.4 years. At baseline, 32% of the children reported that they knew their HIV status already (no difference between control and intervention groups). Disclosures in both control and intervention arms increased over follow‐up, but the intervention arm had significantly more disclosures. Using child‐reported disclosure, the prevalence of disclosure increased significantly between the baseline and 24 months of follow‐up from 29.2% to 58.5% in the control arm and from 33.2% to 74.0% in the intervention arm (difference of 15.5%, 95% confidence interval: 3.7, 27.3). Overall, there were not significant differences in mental and behavioral health outcomes, although trends suggested mental and behavioral distress increased at month 6 in the intervention group as disclosures increased, and then decreased compared to controls thereafter.

**Conclusions: **This study provides evidence for an effective, clinic‐based intervention to increase disclosure of HIV status to children living with HIV. Making counseling support available throughout the disclosure process may be particularly important to navigate increased psychological distress immediately after disclosure and move towards resilience.

## THPDE0201

### Empirical cost of community mobilization activities to support the scale‐up of Universal Test and Treat in Swaziland

**S. Khan^1^; C. Wong^1^; T. Maziya^1^; F. Walsh^2^; C. Lejeune^1^; Y. Fleming^3^; G. Khumalo^4^; M. Zwane^5^; V. Okello^6^ and T. Bärnighausen^7,8^**

^1^Clinton Health Access Initiative ^CHAI^, Mbabane, Swaziland, ^2^Clinton Health Access Initiative ^CHAI^, Boston, United States, ^3^aidsfonds, Amsterdam, Netherlands, ^4^Swaziland National Network of People Living with HIV/AIDS ^SWANNEPHA^, Mbabane, Swaziland, ^5^SAfAIDS, Mbabane, Swaziland, ^6^Ministry of Health, Mbabane, Swaziland, ^7^Harvard T.H. Chan School of Public Health, Boston, United States, ^8^Heidelberg Institute of Public Health, University of Heidelberg, Heidelberg, Germany

**Background:** Swaziland and many other countries in sub‐Saharan Africa are adopting the 2015 World Health Organization (WHO) antiretroviral therapy (ART) guidelines and implementing universal test and treat (UTT). However, it has become increasingly clear that without successful community mobilization activities and demand generation, UTT will not lead to substantial increases in ART coverage. Here we present for the first time empirical cost estimates of community mobilization implemented as part of UTT in Swaziland.

**Methods:** From September 2014‐August 2017, we collected comprehensive data from community partners supporting the transition of fourteen public health facilities from the previous standard‐of‐care (SOC) to UTT as part of a stepped‐wedge implementation trial. The activities included community dialogues and mobilization events to educate both targeted and broader community as facilities introduced UTT. We carried out bottom‐up costing of sub‐activities for total unit cost estimation. The mean costs of each activity type are presented in USD ($).

**Results: ** During the observation period, 311 events were carried out at a total combined cost of $90,448. Of this total, 54% were associated with community mobilization activities with mean cost of $348 (95% CI: 137,559) for activities ranging from dialogue with people living with HIV (PLHIV) to broader community. The cost of Information, Education and Communication (IEC) production and printing was $4,404 (95% CI: −7217, 16024) or 15% of total, while the cost of running a community advisory board (CAB) to ensure rights of clients was $348 (95% CI: 137,559) or 23% of total. Remaining costs were associated with orientation of the media and community leaders as well as monitoring and evaluation activities.

**Conclusions: **We present one of the first comprehensive set of estimates of the costs of community related activities carried outside the healthcare facilities to support UTT. The costs vary widely across broad categories due to mix of fixed (equipment rental) and variable costs (distance traveled, food), while CAB costs were high due to initial capital investment needed in training support. Future funding and planning should use these cost estimates to ensure that scarce resources for community mobilization are used to largest possible health and social benefit.

**Abstract THPDE0201‐Table 1. Average cost of community events in support of UTT policy in Swaziland**

**Table:** 

Events	Mean cost per event (USD) (95% CI)
General Orientation	$1267
Media Orientation Workshop	$933
Information, Education and Communication (IEC) material	$4404 (−7217, 16025))
Community Mobilization Events	$231 (198 to 265)
M&E	$145 (98 to 192)
Community Advisory Board (CAB)	$348 (137 to 559)

## THPDE0202

### Increased domestic financing of key population‐led health services (KP‐LHS): Lessons from Thailand's transition planning and response

**K. Rungtanatada^1^; S. Janyam^2^; D. Linjongrat^3^; S. Charoenying^4^; K. Benjamaneepairjoj^4^; M. Kim^5^; M. Sanguankwamdee^5^ and S. Mills^4^**

^1^National Health Security Office (NHSO), Bangkok, Thailand, ^2^Ministry of Public Health (MoPH), Bangkok, Thailand, ^3^Rainbow Sky Association of Thailand, Bangkok, Thailand, ^4^LINKAGES/Thailand, FHI 360, Bangkok, Thailand, ^5^United States Agency for International Development Regional Development Asia, Bangkok, Thailand

**Background:** As international donors such as The Global Fund (GFATM) and PEPFAR transition out of middle‐income countries, there is an urgent need for domestic budgets of countries to increase their financial commitments to the HIV response. In Thailand, nowhere is this more urgent than with HIV cascade interventions for key populations (KPs) most successfully delivered by civil society organizations (CSOs) uniquely able to reach these often‐marginalized persons affected and infected by HIV. Yet these critical KP‐LHS have thus far failed to attract increased domestic financing because of poor targeting of scarce resources, pervasive stigma and discrimination towards KPs, and a lack of contracting mechanisms to fund CSOs providing KP‐LHS.

**Description:** The Thailand National Health Security Office (NHSO) committed approximately $6 million starting in 2016 for initiatives to increase access to HIV testing and antiretroviral treatment among KPs. The initiative was designed to tap the expertise of KP‐led organizations, many of whom have been traditionally financed by GFATM and PEPFAR. NHSO developed reimbursement schemes based on cost estimates of “reach‐recruit‐test‐treat‐prevention‐retain” activities which vary for each KP group. These amounts were made available to organizations such as CSOs, hospitals, and provincial health offices providing services for KPs through contracts with NHSO.

**Lessons learned:** Despite the increases in government budgetary support for KP‐LHS, certain gaps remain. An estimated 60% of the total were funds provided to CSOs and 40% were provided to hospitals. NHSO identified the following gaps and needs: strengthening of systematic management of funding and service monitoring; accreditation system to ensure organizational and service capacity of CSOs; ensuring that unit cost reflects appropriate operational costs of CSOs; funds integration with other funding sources for greater coverage.

**Conclusions/Next steps:** NHSO will work with stakeholders to address these challenges through establishment of an accreditation process to ensure organizational capacity to manage funds and provide services, conducting of costing studies to ensure alignment of service reimbursement for KP‐LHS under models applicable to each KP group and local epidemic contexts, and strengthening local fund management entities to manage and monitor NHSO funding.

## THPDE0203

### Last to be funded, first to be cut: an analysis of current funding trends to local community organisations in the Positive Action Network

**N. Spicer^1^; D. Kemps^2^ and S. Demellweek^3^**

^1^ViiV Healthcare, Positive Action, London, United Kingdom, ^2^ViiV Healthcare, Brentford, United Kingdom, ^3^ViiV Healthcare, Positive Action, Brentford, United Kingdom

**Background:** To enable the global community to meet its own Fast Track targets, UNAIDS estimates that approximately US$ 26.2 billion will need to be spent on HIV responses in 2020. Despite this, there was a $51 million reduction in donor Government funding for HIV between 2015 and 2016. The critical role of community‐based interventions in the fight against HIV has never been in question, but if funding squeezes are affecting these small groups in equivalent proportions, we could see transmission rates and HIV‐related deaths rise as a result.

**Description:** ViiV Healthcare is conducting a comprehensive survey of organisations engaged in our Positive Action for Children Fund (PACF) and Positive Action for Girls and Women (PAGW) online communities. The survey of 350 current and former grantees and over 2000 community members seeks to understand funding trends since 2015, how grant durations are spread, and in what proportions different funder types are contributing to community HIV interventions in low resource settings.

**Lessons learned:** Preliminary findings from community based organisations (CBOs) indicate that funding opportunities are unchanged (less than 20% change) in the past two years, with commentary that the number of international funders funding local CBOs has also remained unchanged. This is despite the High Level Meeting in 2016 proposing increased funding to local communities to optimise investments in health services.

Offering a rare insight into funding levels in communities, these results are valuable in allowing us to quantify the recent funding trends for grassroots CBOs for the first time, and determine whether the globally reported percentages for funding to CBOs are reflected in the experiences of respondents on the ground.

**Conclusions/Next steps:** The results point to the urgency in prioritising funding to local communities if we are to meet funding commitments and ensure that investments in health systems are optimised. Further research will be needed to assess the intensity of fundraising required to achieve the current level of funding versus previous years.

## THPDE0204

### Post‐global fund HIV financing: a promising transitioning model and the implementation of plan from the SHIFT (Sustainable HIV Financing in Transition) program in Thailand

**R. Jommaroeng^1,2^; Y. Somphoh^1^; K. Uppakaew^1^; A. Karakit^1^; D. Linjonrut^2^; C. Thongbai^3^; P. Panitchpakdi^4^ and CSO Resource Mobilization (CRM) Coalition**

^1^Thai National AIDS Foundation, Bangkok, Thailand, ^2^Rainbow Sky Association of Thailand, Bangkok, Thailand, ^3^Pink Monkey Organization, Lopburi, Thailand, ^4^Raks Thai Foundation, Bangkok, Thailand

**Background:** Thailand has been a recipient country of the Global Fund to Fight against AIDS, Tuberculosis and Malaria(GFATM) since 2004 with the accumulative amount of US$287.4 million which sustains 95.0% of the programs implemented by civil society organisations(CSO) 5.0% is supported by the Subsidiary Fund of the Thai government. The country has been categorised as an upper‐middle income country in 2011, partly resulting in the out‐transitioning of GFATM, negatively affecting sustainability of CSO‐implemented HIV programs.

**Description:** The initial of 30 CSOs, including MSM, transgender women, sex workers, PWID organizations, gathered in late 2015 to form CSO Resource Mobilization(CRM) under “AIDS‐Almost Zero” program to address HIV financing sustainability and currently supported by the SHIFT program to ensure increased fiscal space, allocative efficiency, effective transitioning plan and funding mechanisms for CSOs. We have been advocating the National Health Security Office(NHSO) for a regular annual scheme for CSOs working with key populations. The goal is to reach US$14.7 million/year and US$5.9 million has been successful allocated in 2018 and with the Disease Control Department to increase the Subsidiary Fund from US$1.5 million to US$ 3 million. To minimize the risks of sole government funding, the CRM have initiated a public‐private partnership program to raise funds for US$1.5 million with an exponentially annual increase for five consecutive years.

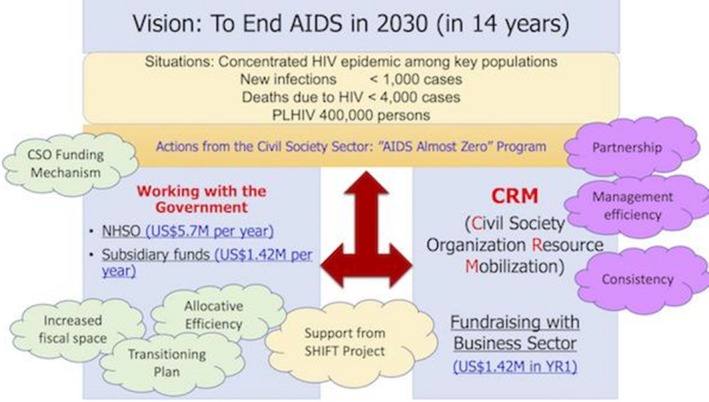

**Abstract THPDE0204‐Figure 1. Thailand's Model of CSO Resource Mobilization on HIV Financing for Post‐GFATM.**

**Lessons learned:** Collaboration and partnership across all key stakeholders is essential for an effective HIV response. Formal dialogues help convey strategic information to the government but informal dialogues help change their perception towards CSOs, influencing favorable policy outcomes. Consistency in engaging with the government is crucial since government staff rotation is to be expected and trust cannot be earned over a few meetings. Instant messaging applications such as Facebook and LINE™ are used as informal but effective communication means, eventually leading to improved trust.

**Conclusions/Next steps:** We are fostering strengthened partnership with both central and local governments with a key message “Get yourselves invited in all policy dialogues, seen in all festive occasions, and perceived as highly reliable partners. In addition, a development of national CSO accreditation is in progress to ensure our effective management, accountability and that we deliver results.

**Abstract THPDE0204‐Table 1. HIV financing policy progress monitoring**

**Table:** 

Policies/Mechanisms	Policy target	Themes	Achievements	Challenges
The US$14.7 million Scheme for Key Populations	The National Health Security Office	Increased fiscal space, Allocative efficiency, CSO funding mechanism	US$5.9 million is allocated annually in 2018. 15 CSOs working with MSM and SW receive direct funding and 9 CSOs through local government agencies	This challenges the Public Administration Reform in Thailand, hence this is innovative. More standardization on allocative efficiency is yet to be strengthened.
The US$3 million Subsidiary Fund	The Ministry of Public Health Department of Disease Control	Increased fiscal space, Allocative efficiency	US$1.5 million is allocated to CSOs in 2018. 500 projects are financed.	More strategic allocation is needed to increase fiscal space as the MoPH promises 500 projects to be funded as the only indicator.
The US$3 million Public‐Private Partnership Fund	Public Sector (Corporate and Individual)	CSO funding mechanism	US$0.79 million has been raised so far.	HIV is no longer an appealing to the public.

## THPDE0205

### A sustainable approach to providing HIV services and information at the community level: a longitudinal exploration of female community health entrepreneurs’ performances

**R.A.J. Borst^1^; M.O. Kok^1,2^; F. Bucker^3^ and D. Muhangi^4^**

^1^Erasmus University Rotterdam, Erasmus School of Health Policy & Management, Rotterdam, Netherlands, ^2^Amsterdam Public Health, Vrije Universiteit Amsterdam, Health Sciences, Amsterdam, Netherlands, ^3^Vrije Universiteit Amsterdam, Health Sciences ^Student^, Amsterdam, Netherlands, ^4^Makerere University, Social Work and Social Administration, Kampala, Uganda

**Background:** Community health entrepreneurship is an innovative way of providing rural communities access to primary healthcare by harnessing the entrepreneurial skills of lay health workers. Previous research shows that community health entrepreneurs have a highly beneficial impact on communities’ knowledge of HIV prevention. However, it remains unclear how the performance of these entrepreneurs evolve over time. Hence, this study aimed to longitudinally compare the performances of lay health workers and community health entrepreneurs. The findings of this study aim to contribute to the construction of a sustainable primary sexual and reproductive healthcare model.

**Methods:** Data were collected using a tablet‐based performance survey in a six‐month quasi‐experimental study. The study aimed to sample 500 female lay health workers from two rural districts in East and Central Uganda who were recruited to become a community health entrepreneur. A random sample of 150 participants would receive their training directly, whereas the other 350 would start six months later. Mixed models were used to longitudinally asses the difference in performance indicators between the two groups. These indicators included their income, self‐esteem, and availability of essential medicines and equipment.

**Results: ** A group of 56 female community health entrepreneurs successfully completed their training directly, followed by a second group 77 six months later. After six months, the entrepreneurs showed sustained performance over the lay health workers. The community health entrepreneurs proved to have a key role in providing rural populations with basic services and products for sexual and reproductive health. The entrepreneurs had a higher availability of essential medicines (OR: 3.39, 95%‐CI: 2.03; 5.65) and key equipment (OR: 1.87, 95%‐CI: 1.03; 3.37). In addition, their Rosenberg self‐esteem score increased with 1.24 points (95%‐CI: 0.09; 2.39) more than that of lay health workers, whereas their weekly overall income increased with $8.96 (95%‐CI: $3.59; $14.32) more than that of lay health workers.

**Conclusions: **Female lay health workers who were trained to become a community health entrepreneur showed an increased and sustained performance in the medium‐term. This study provides the first evidence that community health entrepreneurship may be a sustainable and lasting model through which to organise sexual and reproductive healthcare.

## THPDE0206

### Building a constituency of advocates for sustainability: identifying the capacity needs of HIV CSOs and key populations to advocate for HIV financing and sustainability in Indonesia, Malaysia, Philippines and Thailand

**J. Bagas**

APCASO, Bangkok, Thailand

**Background:** In response to declining donor funding for HIV, the Sustainable HIV Financing in Transition (SHIFT) Programme is supporting HIV financing advocacy for CSOs in four middle‐income countries in Southeast Asia through capacity building and strategic information.

APCASO, a regional sub‐recipient of SHIFT, conducted a needs assessment for CSOs and key population (KP) groups in Indonesia, Malaysia, Philippines and Thailand in 2017 to identify their capacity needs in relation to HIV financing advocacy.

**Description:** APCASO developed a tool for a two‐day focus group discussion where participants representing SHIFT SRs, HIV CSOs, and KP groups scored or assessed their capacities in five areas: programmatic capacity; organisational capacity; individual capacity; linkages to HIV financing stakeholders; and enabling environment.

48 participants from 28 CSOs and KPs took part of the FGDs: Philippines = 5 groups; Malaysia = 8; Indonesia = 8; and Thailand = 7. They represented the following communities and KPs: PLHIV (five groups); MSM (10); TGs (8); SWs (3); PWUDs (5); women PLHIV (1), and YKPs (1). Seven organisations reported representing broad constituencies or all KPs.

**Lessons learned:** Here are some of the major findings of the needs assessment:

(1) Almost all groups reported moderate to weak capacity on key HIV financing topics, such as transition policies, UHC, and budget cycles.

(2) Advocacy is perceived as an add‐on to service delivery despite prevailing notion that advocacy is their core mandate.

(3) There is limited or no engagement with critical government decision‐makers, such as the Finance ministry, treasury, or major political leaders.

(4) Spaces for HIV financing advocacy presented by decentralisation are not maximised. Existing government funding mechanisms for CSOs are not fully understood.

(5) HIV networks present a means to mitigate KP‐specific barriers (such as criminalisation). However, capacity to advocate for KP‐specific programmes varies across groups.

**Conclusions/Next steps:** To address these gaps, APCASO worked with the SRs and SHIFT regional partners to design in‐country advocacy trainings that covered national HIV funding landscapes, UHC, budget cycles, CSO funding mechanisms, as well as advocacy skills on HIV financing. APCASO also developed a set of tailored technical assistance activities for the four countries to continue enhancing capacities on HIV financing.

## THPDD0206

### Differentiated ART delivery model for female sex workers in Uganda: Community client led ART delivery to improve outcomes

**L. Oucul^1^; J. Birungi^2^ and K. David^3^**

^1^The AIDS Support Organization (TASO), Programs and Capacity Development, Kampala, Uganda, ^2^Medical Research Council, Kampala, Uganda, ^3^The AIDS Support Organization (TASO), Kampala, Uganda

**Background:** In 2013, Uganda National ART guidelines recommended test and start for all key populations. The AIDS Support Organization (TASO) started implementing these guidelines in 2014. In 2016, TASO ART data showed16% ART retention of FSW. To support improved outcomes, TASO expanded differentiated ART delivery model and adapted it for FSWs.

**Description:** TASO has been implementing a client‐managed group model of differentiated ART delivery called Community Client Led ART Delivery (CCLAD) since 2012. In 2017, this was expanded and adapted for FSWs. Eligible FSWs who consent to be on group model, were grouped by geographical area into a peer support group of five to ten members. The group selected a peer leader who is responsible for collecting 3 monthly ART refills. All group members attend 6‐ monthly clinical reviews and annual viral load monitoring at TASO clinic.

**Lessons learned:** Between October 2016 and June 2017, more than 7,000 FSWs were reached with a behavior change campaign. A total of 5,775 HIV tests were completed and 525 people living with HIV were identified (positivity rate = 9.1%). Of those identified as HIV%, 81.5% were linked to care and 23.6% of those linked to care were initiated on ART. Over the same time period, a cumulative number of 215 FWs were in care with 89.8% being on ART of which 90.9% were virally suppressed. All 24 FSW in CCLAD group had viral suppression above 95%.

**Conclusions/Next steps:** Key populations can benefit from having access to differentiated ART delivery models. In Uganda, FSWs with access to a differentiated ART delivery model with community ART distribution and less frequency clinical consultations had good client outcomes. This model supported retention in care by tailoring services for a highly mobile population.

## Late Breakers

## TUAA0202LB

### A randomised controlled trial comparing the impact of antiretroviral therapy (ART) with a “Kick‐and‐Kill” approach to ART alone on HIV reservoirs in individuals with primary HIV infection (PHI); RIVER trial

**S. Fidler^1^; W. Stohr^2^; M. Pace^3^; L. Dorrell^3^; A. Lever^4^; S. Pett^2^; S. Kinloch^5^; J. Fox^6^; A. Clarke^7^; M. Nelson^8^; M. Khan^9^; A. Fun^4^; D. Kelly^10^; J. Kopycinski^3^; M. Johnson^5^; T. Hanke^3^; H. Yang^3^; B. Howell^11^; S. Kaye^9^; M. Wills^4^; R. Barnard^11^; A. Babiker^2^; J. Frater^12^ and On Behalf of the RIVER trial investigators**

^1^Imperial College London, Medicine, London, United Kingdom, ^2^University College London, MRC CTU, London, United Kingdom, ^3^University of Oxford, NDM, Oxford, United Kingdom, ^4^University of Cambridge, Cambridge, United Kingdom, ^5^University College London, Royal Free Hospital, London, United Kingdom, ^6^Kings College London, Guys & St Thomas Kings, London, United Kingdom, ^7^Univeristy of Brighton, Medicine, Brighton, United Kingdom, ^8^Chelsea & Westminster Hospital, Medicine, London, United Kingdom, ^9^Imperial College London, London, United Kingdom, ^10^Patient advocacy Alliance CIC, Manchester, United Kingdom, ^11^Merck MSD, Pennsylvania, United States, ^12^University of Oxford, Peter Medwar, Oxford, United Kingdom

**Background:** ART alone is unable to cure HIV because of an inaccessible pool of latently infected cells, the HIV reservoir. In the first randomised controlled trial (RCT) of a “kick‐and‐kill” strategy, amongst participants with PHI on ART, we investigated the impact of HIV‐specific T‐cell vaccines and a latency reversing agent (vorinostat) on the HIV reservoir.

**Methods:** Individuals who started ART within four weeks of confirmed PHI diagnosis with suppressed plasma HIV‐RNA were randomised to either ART plus vaccination with ChAdV63.HIVconsv prime <1 week post‐randomisation (PR) and MVA.HIVconsv boost (eight weeks later) followed by 10 doses of 400 mg oral vorinostat taken every three days (intervention) or ART alone (control).

The two arms were compared for the primary outcome (log10 total HIV‐DNA copies/million from CD4 +  T‐cells at weeks PR‐16&18) using analysis of covariance adjusted for baseline level. Secondary endpoints included quantitative viral outgrowth measuring replication‐competent HIV‐1 reservoir at week PR‐16, HIV‐specific CD4 +  and CD8 +  T‐cell responses by intracellular cytokine staining at weeks PR‐9&12, histone acetylation pre&post vorinostat and adverse events.

**Results: ** 60 male participants were randomised at 6 UK sites (30 intervention, 30 control), with median: age 32 years, 26 weeks since ART start and CD4 +  count 708 cells/mm^3^. All participants completed follow‐up. There was no difference between the arms in the primary outcome (intervention vs. control: 0.041 (95% CI −0.031, 0.113) log10 HIV‐DNA copies/million CD4 +  T‐cells *p *=* *0.256), or in the proportion with undetectable viral outgrowth (0.42 (95% CI 0.13, 1.37) *p *=* *0.151) Participants with undetectable viral outgrowth had significantly lower total HIV‐DNA. Participants in the intervention arm showed significantly higher HIV‐specific CD4 +  (IFNg/IL2/TNFa/CD154) and CD8 +  (IFNg/TNFa) T‐cell responses post vaccination than control. Histone acetylation increased 3.2‐fold two hours post‐vorinostat (*p *<* *0.001). There was no virological failure or intervention‐related SAE. More clinical adverse events were reported in the intervention arm, all were mild/moderate.

**Conclusions: **In the first “kick‐and‐kill” RCT in PHI, despite evidence of robust vaccine‐induced HIV‐specific T‐cell immunity and vorinostat activity, there was no impact on measures of HIV reservoir compared to ART alone. Vaccination and vorinostat did not raise any safety concerns. Further analyses are ongoing to explore mechanisms to explain these findings.

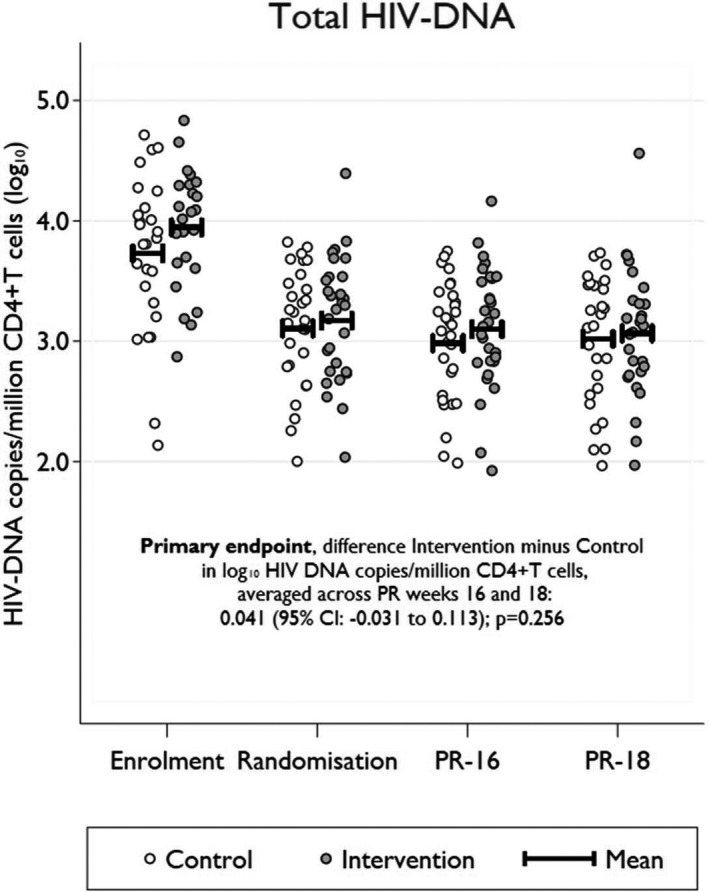

**Abstract TUAA0202LB‐Figure 1. of total HIV DNA by arm.**

## TUAA0206LB

### Evaluation of an antibody to Alpha4Beta7 in the control of SIV infection

**M. Di Mascio^1^; J.J. Lifson^2^; S. Srinivasula^3^; P. Degrange^4^; B. Keele^2^; Y. Wang^1^; P. Lusso^1^; M. Proschan^1^; H.C. Lane^1^ and A.S. Fauci^1^**

^1^National Institute of Allergy and Infectious Diseases, Bethesda, United States, ^2^AIDS and Cancer Virus Program, Frederick National Laboratory for Cancer Research, Frederick, United States, ^3^Leidos Biomedical Research, Inc, Frederick, United States, ^4^Battelle/Charles River‐Integrated Research Facility, NIAID Frederick, Frederick, United States

**Background:** It was recently reported that treatment with an antibody to the a4b7 integrin in rhesus macaques infected with SIVmac 239 having a stop codon in nef (SIVmac239nefstop) was associated with prolonged post‐treatment suppression of viremia. The present study was undertaken to try to confirm and extend those observations.

**Methods:** Twenty‐two Mamu‐A001, Mamu‐B008 and Mamu‐B017 negative juvenile to adult Indian rhesus macaques (>4 kg or three years; mixed sex) were infected intravenously with 200 TCID50 SIVmac239nefstop (courtesy F. Villinger). At five weeks post‐infection (wpi), combination anti‐retroviral therapy (cART) was started with tenofovir, emtricitabine and L‐870812. After four weeks of cART, animals received a total of 8 infusions every three weeks of primatized anti a4b7 antibody (n = 12) or control antibody (n = 10); cART was stopped at 18 wpi (after the fourth antibody infusion). Plasma SIV RNA levels had been monitored through at least 33 wpi at the time of abstract submission.

**Results: ** Peak plasma SIV RNA levels averaged ˜10^6 copies/mL in both groups. Sequencing confirmed the presence of the expected stop codon in nef in the challenge virus, with restoration to nef open by 5 wpi in all animals. Four weeks after cART initiation plasma SIV RNA levels were <100 copies/mL in 10/12 experimental group animals and 10/10 controls. Three weeks following discontinuation of cART, plasma SIV RNA levels rose to above 100 copies/mL in 10/12 a4b7‐treated animals and 8/10 controls. Fifteen weeks following cART interruption (33 wpi) mean plasma SIV RNA levels were ˜ 10^4 copies/mL in both groups and not significantly different

**Conclusions: **In the present study, administration of an antibody to the a4b7 integrin in conjunction with short term cART was not associated with control of SIV replication following treatment cessation. Follow up studies to explore potential reasons for differences between the present findings and published results will focus on potential antidrug antibody responses and other possibilities.

## TUAB0106LB

### Non‐inferior efficacy of dolutegravir (DTG) plus lamivudine (3TC) versus DTG plus tenofovir/emtricitabine (TDF/FTC) fixed‐dose combination in antiretroviral treatment‐naïve adults with HIV‐1 infection ‐ 48‐week results from the GEMINI studies

**P. Cahn^1^; J. Sierra Madero^2^; J. Arribas^3^; A. Antinori^4^; R. Ortiz^5^; A. Clarke^6^; C.‐C. Hung^7^; J. Rockstroh^8^; P.‐M. Girard^9^; C. Man^10^; J. Sievers^11^; A. Currie^12^; M. Underwood^10^; A. Tenorio^10^; K. Pappa^10^; B. Wynne^10^; M. Gartland^10^; M. Aboud^11^ and K. Smith^10^**

^1^Fundación Huésped, Buenos Aires, Argentina, ^2^Instituto Nacional de Ciencias Médicas y Nutrición Salvador Zubirán, Mexico City, Mexico, ^3^Hospital La Paz, Madrid, Spain, ^4^Istituto Nazionale per le Malattie Infettive Lazzaro Spallanzani, Rome, Italy, ^5^Bliss Healthcare Services, Orlando, United States, ^6^Royal Sussex County Hospital, Brighton, United Kingdom, ^7^National Taiwan University Hospital, Taipei, Taiwan, Province of China, ^8^Rheinische Friedrich‐Wilhelms Universität, Bonn, Germany, ^9^Hôpital Saint Antoine, Paris, France, ^10^ViiV Healthcare, Research Triangle Park, United States, ^11^ViiV Healthcare, Brentford, United Kingdom, ^12^GlaxoSmithKline, Stockley Park, United Kingdom

**Background:** The requirement for life‐long antiretroviral therapy of HIV infection has highlighted interest in 2‐drug regimens (2DRs) to minimise cumulative drug exposure. DTG´s potency, safety and resistance barrier make it an optimal core agent for 2DRs while 3TC´s safety, tolerability and efficacy make it an attractive partner for initial HIV‐1 treatment.

**Methods:** GEMINI‐1 and GEMINI‐2 are two identical global double‐blind, multicentre Phase III studies evaluating efficacy and safety of DTG+3TC once daily in treatment‐naïve HIV‐1‐infected adults with Screening HIV‐1 RNA ≤500,000 copies/mL (ClinicalTrials.gov: NCT02831673/NCT02831764). Participants were randomised 1:1 (stratified by Screening plasma HIV‐1 RNA and CD4 +  cell count) to treatment with DTG+3TC or DTG+TDF/FTC. The primary endpoint is the proportion of participants with plasma HIV‐1 RNA <50 copies/mL at Week 48 (Snapshot algorithm).

**Results: ** 714 and 719 adults were randomised and treated in GEMINI‐1&2, respectively. Participants were well matched for demographic/baseline characteristics. Overall, 20% of participants had baseline HIV‐1 RNA >100,000 copies/mL; median CD4 +  was 432 cells/mm^3^. Based on a 10% non‐inferiority margin, DTG+3TC was non‐inferior to DTG+TDF/FTC at Week 48 in both GEMINI‐1&2 and in the pooled analysis (Table 1). Response rates in subjects with baseline HIV‐1 RNA >100,000 copies/mL were high and similar between arms. Across both studies, 6 participants on DTG+3TC and 4 on DTG+TDF/FTC met protocol‐defined virologic withdrawal criteria through Week 48; none had treatment‐emergent primary integrase‐strand transfer inhibitor or NRTI resistance mutations. Overall rates of AEs were similar between arms, with low rates of withdrawals due to AEs for both DTG+3TC and DTG+TDF/FTC. More drug related AEs were reported with DTG+TDF/FTC. Post baseline changes in markers of bone and renal function favoured DTG+3TC through week 24.

**Abstract TUAB0106LB‐Table 1. Proportion of Participants with Plasma HIV‐1 RNA <50 copies/mL at Week 48: Snapshot Analysis ‐ ITT‐E population**

**Table:** 

		GEMINI‐1	GEMINI‐2	Pooled
Snapshot responders	DTG+3TC	320/356 (90%)	335/360 (93%)	655/716 (91%)
	DTG+TDF/FTC	332/358 (93%)	337/359 (94%)	669/717 (93%)
Adjusted Difference (95% CI)		−2.6 (−6.7, 1.5)	−0.7 (−4.3, 2.9)	−1.7 (−4.4, 1.1)

**Conclusions: **In GEMINI‐1&2, DTG+3TC demonstrated non‐inferior efficacy to DTG+TDF/FTC in treatment‐naïve adults with Screening HIV‐1 RNA ≤500,000 copies/mL at Week 48. Both regimens were well tolerated. Biomarkers of bone turnover and renal function favoured DTG+3TC. The results suggest DTG+3TC is an option for initial treatment of HIV‐infected patients.

## TUAB0107LB

### Non‐inferior efficacy for darunavir/ritonavir 400/100 mg once daily versus lopinavir/ritonavir, for patients with HIV RNA below 50 copies/mL in South Africa: The 48‐week WRHI 052 study

**F. Venter^1^; M. Moorhouse^1^; S. Sokhela^1^; E. Maharaj^1^; G. Akpomiemie^1^; B. Simmons^2^; C. Serenata^1^ and A. Hill^3^**

^1^University of Witwatersrand, WITS Reproductive Health and HIV Institute, Johannesburg, South Africa, ^2^Imperial College, Faculty of Medicine, London, United Kingdom, ^3^Liverpool University, Pharmacology, Liverpool, United Kingdom

**Background:** Darunavir/ritonavir (DRV/r) is the most widely recommended protease inhibitor in treatment guidelines. The approved dose of DRV/r is 800/100 mg once daily (QD) for patients with no PI resistance. In the POWER studies, patients treated with a lower dose ‐ 400/100 mg QD ‐ showed similar reductions in HIV RNA to the standard dose, with consistent results shown in other pilot studies (DARULIGHT, DRV600). Reductions in the dose of DRV/r could improve safety and lower costs of mass treatment in low‐ and middle‐income countries. Low cost generic DRV/r is becoming available in many countries as patents expire.

**Methods:** In this study in Johannesburg, South Africa, 300 patients previously stable on 2NRTI+LPV/r with HIV RNA <50 copies/mL were randomised to 2NRTI+DRV/r 400/100 mg QD (n = 148) or continued 2NRTI+LPV/r (n = 152). Treatment success was defined as HIV RNA <50 copies/mL at Week 48 (FDA snapshot). Treatment arms were compared using the new FDA non‐inferiority (NI) margin of −4% for switch studies, using the Intent to Treat (ITT) population.

**Abstract TUAB0107LB‐Table 1. WRHI 052 trial – 48 week results (n = 300)**

**Table:** 

Treatment arm	2NRTI + DRV/r	2NRTI + LPV/r
Sample size	148	152
HIV RNA <50 copies/mL	143 (96.7%)	145 (95.4%)
Grade 1 to 4 adverse events	101 (68.2%)	108 (71.1%)
Grade 3 to 4 adverse events	7 (4.7%)	5 (3.3%)
Serious adverse events	5 (3.4%)	4 (2.6%)
Discontinuation for adverse events	3 (2.0%)	0 (0.0%)

**Results: ** Patients were 68% female and 99.7% Black, with mean age 42 years, CD4 count 621 cells/µL, body weight 72 kg. In the primary efficacy analysis, HIV RNA <50 copies/mL by Week 48 (ITT) was 143/148 (96.7%) in the DRV/r arm versus 145/152 (95.4%) in the LPV/r arm (Difference = +1.2% (95% CI = −3.7% to +6.2%). Of the 12 patients with failure, 7 had low‐level viraemia (50 to 199 copies/mL), 2 had transient high‐level viraemia at Week 48 which resolved after adherence counselling; 3 had missing data. Summary safety data is shown in the table below.

**Conclusions: **In this study for patients with HIV RNA <50 copies/mL at baseline, switching to 2NRTI+DRV/r 400/100 mg once daily showed non‐inferior efficacy versus 2NRTI+LPV/r in the primary efficacy analysis (96% vs. 95%), within the −4% FDA non‐inferiority margin for switch studies. For stable patients, switching from LPV/r to DRV/r 400/100 QD would improve convenience and could lower long‐term costs. New clinical trials are required to evaluate DRV/r 400/100 mg after first‐line treatment failure, where this PI is most widely used.

## TUAC0207LB

### Integrated biological and behavioural surveillance (IBBS) survey among men who have sex with men in South Sudan

**M. Solangon^1^; K. Kriitmaa^2^; S. Taher^1^; V. Achut^3^; A.J. Nyniyal^3^; H.D. Awongo^4^; G. Atillio^4^; P. Moyo^5^ and P. Jasi^6^**

^1^International Organization for Migration (IOM), Juba, South Sudan, ^2^International Organization for Migration (IOM), Geneva, Switzerland, ^3^Republic of South Sudan, Ministry of Health (MoH), Juba, South Sudan, ^4^Republic of South Sudan, South Sudan HIV/AIDS Commission (SSAC), Juba, South Sudan, ^5^Consultant, East London, South Africa, ^6^International Organization for Migration (IOM), Dhaka, Bangladesh

**Background:** South Sudan has a 2.6% HIV prevalence rate and is regarded as low and generalized with pockets of high concentration among key populations (KPs). Female sex workers (FSW), their clients and men who have sex with men (MSM) account for 63% of new adult HIV infections. No research has been done to date on MSM risk behaviours, limiting development of evidence‐based interventions. This first ever IBBS survey among MSM aimed at establishing HIV and syphilis prevalence and risk behaviours to better inform policy and programming.

**Methods:** The IBBS study was conducted in six sites in South Sudan. MSM is a criminalised behaviour in South Sudan, thus the study utilized non‐probability sampling techniques including purposive and snowball sampling. Data collection methods used included electronic structured questionnaires, key informant interviews and focus group discussions. Respondents were tested for HIV and syphilis, using national testing algorithms.

**Results: ** A total of 165 MSM participated in this survey. About 92% (n = 152) were South Sudanese, and 87% (n = 143) were under 28 years old. The HIV prevalence among MSM was 3.3% (N = 152), higher than the 2.6% national average. The syphilis prevalence was also 3.3% (N = 152). About 36% (n = 59) of the MSM had a relationship with a female, which shows that sexual partners of MSM are not only same‐sex but also heterosexual. About 26% (n = 43) used condoms with non‐regular partners every time and 12% (n = 20) had a sexually transmitted infection (STI) during the past 12 months preceding the survey. Only 5% (n = 8) had ever disclosed that they are MSM to health care workers, 22% (n = 36) had avoided health services due to stigma and discrimination, while 26% (n = 43) had access to HIV and AIDS programmes.

**Conclusions: **The preliminary results provide the first ever baseline data on the sexual behaviours, HIV and syphilis prevalence for MSM in South Sudan. Results show higher HIV prevalence than the national average, presence of high‐risk sexual behaviours, limited access to tailored HIV and AIDS programmes and high levels of stigma and discrimination towards MSM. These findings illustrate the need to strengthen the country's policies and KP‐focused interventions.

## TUAC0307LB

### Integrating oral HIV pre‐exposure prophylaxis (PrEP) in a public family planning facility and youth center to inform national roll out in Zimbabwe

**M.M. Gombe^1^; Y. Mangwendeza^2^; G. Ncube^3^; N. Zwangobani^4^; M. Prust^5^; B. Cakouros^5^; A. Svisva^2^; A. Mangwiro^2^; M. Murwira^4^; A. Mkwamba^1^; A. Erlwanger^2^ and 3DE CHAI**

^1^Clinton Health Access Initiative, Demand Driven Evaluations for Decisions, Harare, Zimbabwe, ^2^Clinton Health Access Initiative, Harare, Zimbabwe, ^3^Ministry of Health and Child Care, HIV prevention, Harare, Zimbabwe, ^4^Zimbabwe National Family Planning Council, Harare, Zimbabwe, ^5^Clinton Health Access Initiative, Boston, United States

**Background:** There is limited information on the process of offering oral PrEP to the general at‐risk population in resource‐limited public sector settings globally. Programming has been limited to private sector demonstration projects targeting key populations. This study aims to provide operational guidance to the Zimbabwe Ministry of Health on national public sector integration of oral PrEP as part of combination HIV prevention.

**Description:** PrEP was piloted in an urban family planning clinic and a rural youth center, integrating it into existing HIV prevention and reproductive health services and activities. 150 HIV negative clients screened as being at high risk of HIV infection were offered PrEP between January and April 2018, primarily through provider initiative. Demand generation included group client education and in‐facility posters and pamphlets; community leader sensitization was also conducted at the rural center. Semi‐structured interviews on PrEP experiences were conducted with five healthcare workers (HCWs) and 37 clients who agreed to be followed up (7 decliners and 30 accepters).

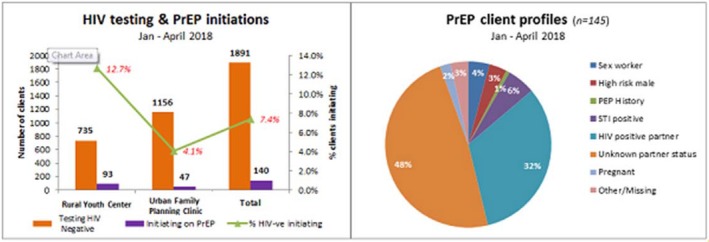

**Abstract TUAC0307LB‐Figure 1. Graph showing percentage uptake of PrEP and risk status of clients.**

**Lessons learned:** PrEP risk assessment can be integrated into existing routine family planning and HIV testing and counselling services. Differences in facility enrollment data and interviews with study participants indicate that HCW knowledge and attitudes, and client awareness, affect whether PrEP is offered and/or accepted. PrEP uptake is driven by partner's risky behaviour or positive HIV status; this did not vary by population group. Female uptake, retention and adherence is largely determined by family or partner support. Male PrEP clients were primarily in polygamous marriages where one wife is HIV positive. Male decliners prefer condoms. Females in sero‐discordant marriages report decreased condom use after PrEP initiation. This is driven by their male HIV positive partners.

**Conclusions/Next steps:** PrEP demand generation strategies should address HCW knowledge and client awareness. National clinical training tools for HCWs should emphasize client education and counselling on PrEP as part of combination of HIV prevention options. The national HIV Communication Strategy should focus on increasing general awareness of how PrEP works in order to increase uptake and reduce stigma associated with use. Adjustments to national PrEP target‐setting will be informed by study findings on PrEP uptake among those testing HIV negative.

## TUAD0308LB

### To give birth and die: The needs of HIV‐positive mothers for retention in longer‐term HIV care and treatment in Russia

**E. Shastina^1,2^; J. Godunova^3^; A. Yakovleva^4^; E. King^5^; I. Evdokimova^3^; N. Sukhova^3^; E. Titina^6^ and D. Legchilova^7^**

^1^The Association “E.V.A.,” Orenburg, Russian Federation, ^2^Autonomous Nonprofit Organization for Prevention of Socially Significant Diseases “New life,” Orenburg, Russian Federation, ^3^The Association “E.V.A.,” St. Petersburg, Russian Federation, ^4^Sociological Institute of Russian Academy of Sciences, St. Petersburg, Russian Federation, ^5^University of Michigan School of Public Health, Ann Arbor, United States, ^6^The Association “E.V.A.,” Samara, Russian Federation, ^7^The Association “E.V.A.,” Khanty‐Mansi Autonomous Ddistrict, Russian Federation

**Background:** The feminization of the HIV epidemic continues in Russia. PMTCT offers the opportunity to engage women in treatment but Option B+ is not commonly accepted. Little is known about HIV care and treatment retention among women after pregnancy. Our research objective was to identify factors influencing postpartum retention in treatment in Russia.

**Methods:** We conducted mixed‐methods, community‐based participatory research in 7 regions across Russia. In phase one, we conducted in‐depth interviews with WLHIV and service providers in order to explore and identify factors related to HIV care and treatment among HIV‐positive mothers. In phase two, we administered a survey to 200 HIV‐positive mothers in order to identify the most salient factors and measure the strength of their association with postpartum treatment. We triangulated study results across sites and sources to draw conclusions. WLHIV played an active role in study design, data collection and interpretation of findings.

**Results: ** 1. The majority of women don't connect ARV with their own health but rather with the goal of PMTCT. Health care providers frequently hold the idea that ARV use is solely to protect the future child and don't encourage women's longer‐term use.

2. Psychosocial support is devastatingly low. Lack of counseling has adverse effects on HIV treatment retention. Only 27% received post‐test counseling. 80% of women not receiving pre‐test counseling during pregnancy reported skipping doses or stopping ARVs postpartum.

3. Every third woman experienced a change in dosage or treatment plan. Taking pills twice daily and side effects were especially difficult postpartum. 25% lacked provider support when experiencing harsh side effects.

4. Single mothers, mothers afraid to disclose their serostatus, and mothers married to AIDS denialists are especially vulnerable to stopping treatment.

5. The majority of women expressed grave financial need ‐lacking money for food or housing. Financial hardship was associated with poor ARV adherence.

**Conclusions: **Programs to support HIV‐positive mothers are needed across Russia that incorporate education on the benefits of ARV, emphasize valuing one's own health, promote skills (nutrition, lifestyle, sobriety), strengthen social support, and focus on families. Structural issues need to be addressed: including, economic opportunities and availability of quality ARVs.

## WEAA0108LB

### The majority of the replication‐competent virus in the latent reservoir originates from viruses circulating near the time of ART initiation

**S.B. Joseph^1^; M.‐R. Abrahams^2^; N. Garrett^3^; S. Zhou^4^; M. Moeser^4^; W. Burgers^2^; L. Tyers^2^; D. Matten^5^; C. Anthony^2^; O. Council^1^; N. Archin^6,7^; D.M. Margolis^6,7^; S. Abdool Karim^3,8^; R. Swanstrom^4,9^ and C. Williamson^2,3^**

^1^University of North Carolina at Chapel Hill, Department of Microbiology and Immunology, Chapel Hill, United States, ^2^University of Cape Town Faculty of Health Sciences, Division of Medical Virology, Institute of Infectious Disease and Molecular Medicine, Cape Town, South Africa, ^3^Centre for the AIDS Programme of Research in South Africa (CAPRISA), Durban, South Africa, ^4^University of North Carolina at Chapel Hill, UNC Center for AIDS Research, Chapel Hill, United States, ^5^University of Cape Town Faculty of Health Sciences, Division of Medical Virology, institute of Infectious Disease and Molecular Medicine, Cape Town, South Africa, ^6^University of North Carolina at Chapel Hill, Division of Infectious Diseases, School of Medicine, Chapel Hill, United States, ^7^University of North Carolina at Chapel Hill, UNC HIV Cure Center, Chapel Hill, United States, ^8^Columbia University, Department of Epidemiology, New York City, United States, ^9^University of North Carolina at Chapel Hill, Department of Biochemistry and Biophysics, Chapel Hill, United States

**Background:** HIV‐1 persists during suppressive therapy in latently infected cells. These cells have been observed in all HIV‐infected people even if treated very early, but little is known about when viruses enter the latent reservoir. Such knowledge would inform our understanding of the dynamics of this reservoir.

**Methods:** Plasma samples were collected longitudinally from 9 HIV‐infected, ART‐naive women enrolled in the CAPRISA 002 acute infection cohort who initiated therapy when CD4 +  T cell count dropped below 500 cells/µL (mean = 4.5 year p.i.). After prolonged ART (mean = 5.1 years), resting CD4 +  T cells were isolated from blood and cultured in a quantitative viral outgrowth assay (QVOA) to sample virus in the latent reservoir. vRNA from plasma collected longitudinally pre‐ART was analyzed by MiSeq deep sequencing across the genome, and PacBio was used to analyze vRNA from p24 +  QVOA outgrowth wells to generate near full‐length sequences. Phylogenetic analyses were performed to compare these two types of sequences.

**Results: ** Of the genetically unique outgrowth viruses sampled from each participant, 60% to 100% were related most closely to virus circulating in the year prior to ART initiation, and 0% to 22% to virus circulating during the first two years of infection. This suggests that the start of therapy is associated with the formation of the majority of the persistent reservoir. In sampling 6 to 44 outgrowth viruses per person, between 0% and 50% of these viruses were genetically identical indicating they were derived from clonally expanded cells, with half arising well before ART initiation and the other half proximal to the start of ART.

**Conclusions: **We used virus evolution off therapy as a molecular clock to date formation of the latent reservoir. Unexpectedly, the majority of the viruses that we detected entered the reservoir near the time of therapy. Our results support several models including ones in which ART‐induced changes to the host immune environment promote latency in HIV‐infected cells and/or increase the half‐life of latently infected cells. These findings provide unique insights into the formation of the latent reservoir and suggest that interventions at the time of ART initiation may significantly reduce the size of the latent reservoir.

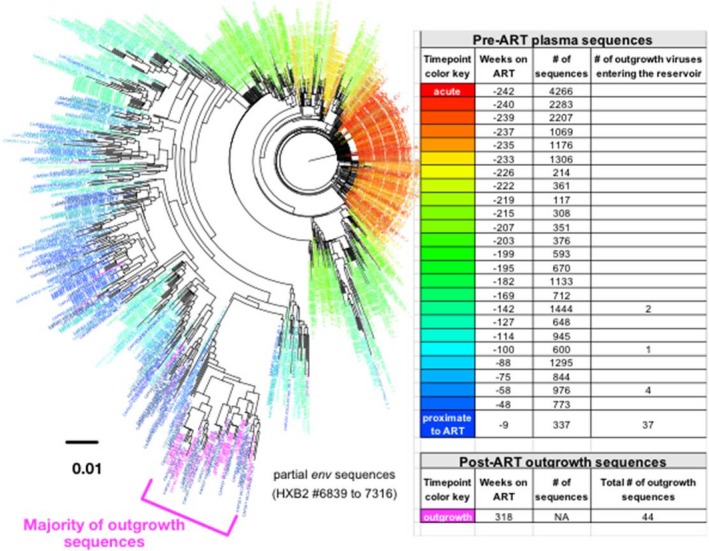

**Abstract WEAA0108LB‐Figure 1. Representative example of the phylogenetic relationship between pre‐ART plasma sequences and sequences from the replication‐competent reservoir.**

## WEAB0208LB

### Impact of late versus early antiretroviral therapy on PBMC‐associated HIV‐1‐DNA levels and the percentage of naive T lymphocytes in HIV‐1 infected children and adolescents ‐ The ANRS‐EP59‐CLEAC study

**P. Frange^1,2^; V. Avettand‐Fenoel^1,2^; J. Le Chenadec^3^; C. Dollfus^1^; L. Nailler^3^; O. Dialla^3^; T. Montange^4^; D. Batalie^4^; M. Fillion^2^; I. Leymarie^3^; L. Ait Si Selmi^3^; T. Wack^3^; J. Warszawski^3^; F. Buseyne^4^ and ANRS‐EP59‐CLEAC Study Group**

^1^AP‐HP, Paris, France, ^2^Université Paris Descartes, EA7327, Paris, France, ^3^INSERM, Le Kremlin‐Bicêtre, France, ^4^Institut Pasteur, Unité d'Epidémiologie et Physiopathologie des Virus Oncogènes, Paris, France

**Background:** Early combined antiretroviral therapy (cART) initiation reduces progression to AIDS and death and decreases the cell‐associated viral reservoir in children infected with HIV‐1 during the perinatal period. Little data are available on the benefit of early cART initiation for children over five years of age and adolescents. The ANRS‐EP59‐CLEAC study aimed to assess the immunological and virological characteristics of HIV‐1‐infected children and adolescents who achieved initial virological suppression, according to age at cART initiation (<6 months vs. ≥24 months of age).

**Methods:** Patient recruitment was conducted in the Paris area between 2016 and 2018. Total cell‐associated HIV‐1 DNA was quantified in the blood using ultrasensitive real‐time PCR (adapted from Biocentric, Bandol, France). CD4 and CD8 CD45RA+CCR7 +  naive T lymphocytes were quantified in fresh blood by flow cytometry. The Kruskal‐Wallis test was used to compare the parameters of early/late treated children (five to twelve years) and adolescents (13 to 17 years).

**Results: ** We prospectively enrolled 27 children (E‐Ch) and nine adolescents (E‐Ado) in the early‐cART group, and 19 children (L‐Ch) and 21 adolescents (L‐Ado) in the late‐cART group. The patients were mainly girls (54%), born in mainland France (60%) to mothers originating from Sub‐Saharan African countries (74%). At the time of the study, all patients were receiving ART, 76% had undetectable plasma HIV‐1 RNA, and the median (interquartile range) CD4 T‐cell count was 824 (660; 1167) cells/µL. HIV‐1 DNA levels were lower in the early‐cART than late‐cART groups for both children and adolescents (medians were 2.2 (E‐Ch), 2.9 (L‐Ch), 2.3 (E‐Ado), and 3.0 (L‐Ado) log10 copies/10^6^ PBMCs, *p *<* *0.0001). Data on the percentage of naive CD4 and CD8 T lymphocytes were available for 58 subjects. We observed the highest percentages in E‐Ch (medians in the E‐Ch, L‐Ch, E‐Ado, and L‐Ado groups were respectively 61, 54, 36, and 55% for CD4, *p *=* *0.02; and 50, 33, 29, and 29%, for CD8, *p *=* *0.004).

**Conclusions: **Early cART initiation during infancy is associated with lower short‐ and long‐term PBMC‐associated HIV‐1 DNA levels, as targeted in HIV‐1 remission strategies. An immunological benefit of early cART initiation on naive T lymphocytes was suggested in children from this study.

## WEAD0208LB

### The third generation of HIV: World first longitudinal study of pregnancy in adolescents living with HIV

**E. Toska^1,2^; L. Sherr^3^; L. Cluver^2,4^; S. Zhou^1^ and Mzantsi Wakho Cohort Study**

^1^University of Cape Town, AIDS and Society Research Unit, Cape Town, South Africa, ^2^University of Oxford, Department of Social Policy and Intervention, Oxford, United Kingdom, ^3^University College London, London, United Kingdom, ^4^University of Cape Town, Dept of Psychiatry and Mental Health, Cape Town, South Africa

**Background:** Nearly 1.7 million HIV‐positive adolescent girls in Southern Africa experience high rates of pregnancy, poor birth outcomes, and AIDS‐related mortality. Option B+ has dramatically reduced the overall rates of paediatric HIV‐infections, but the legacy of adolescents living with HIV persists. HIV‐positive adolescent mothers have exceptionally high rates of mortality, morbidity, and onwards vertical HIV transmission. Their HIV‐exposed children face increased risk of delayed HIV‐testing and ART initiation, worse retention in care, poorer health and higher mortality. The study of the syndemic of adolescent pregnancy and HIV is urgently needed. This study presents the first data on rates of incident and repeated pregnancies among adolescent girls living with HIV and factors shaping early motherhood.

**Methods:** All HIV‐positive adolescent from 52 clinics in a health district in South Africa were approached (90.1%), resulting in n = 563 HIV‐positive adolescent girls. Two interviews were conducted 18‐months apart, following voluntary consent from adolescents and caregivers. Data collection tools were piloted with n = 25 HIV‐positive adolescents. Analyses investigated factors longitudinally associated with incident and repeated pregnancies: socio‐demographic, family, mental health, mode of infection and HIV‐related factors using STATA25.

**Results: ** N = 98/563 (17%) reported lifetime pregnancy, with N = 54 (9.6%) becoming pregnant between the interviews (incident pregnancy) and 37 (6.6%) reporting multiple pregnancies. The majority (n = 76, 82.6%) reported knowing their children's HIV‐status, with n = 4 (4.35%) reporting having at least one HIV‐positive child. HIV‐positive adolescents who were pregnant were more likely to be older, from poor households, not enrolled in school, suicidal, and sexually infected (Figure 1). Three factors were longitudinally associated with both incident and repeated pregnancy: age >15 years‐old (OR 10.7, CI 3.6 to 31.9, *p *<* *0.001), not being enrolled in school at baseline (OR 4.2, CI 2.0 to 9.6, *p *<* *0.001), and being sexually/ behaviourally infected (OR 10.9, CI 4.9 to 24.0, *p *<* *0.001).

**Conclusions: **The next generation of HIV infection dawns. Factors which result in HIV infection in young people may have long‐term ramifications for HIV‐positive young mothers and the next generation ‐ their HIV‐exposed/ infected children. Adolescent pregnancy (and multiple pregnancy) is an issue, with cycles of disadvantage clustering in terms of poverty, school dropout and mental health. Breaking the cycle of onward transmission to their children is an urgent intervention gap.

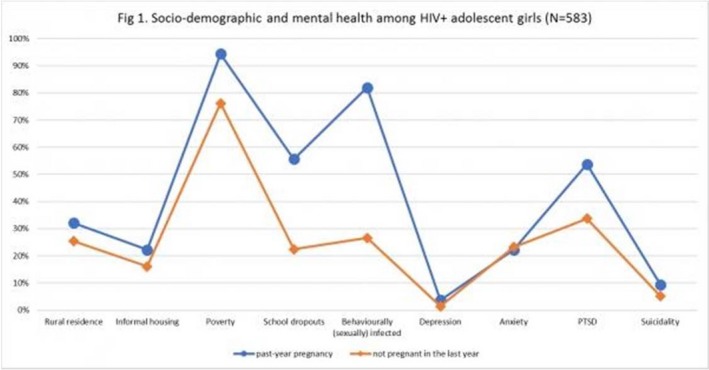

**Abstract WEAD0208LB‐Figure 1. Socio‐demographic and mental health among HIV+ adolescent girls (N = 583).**

## WEAE0406LB

### Incidence of HIV‐infection in the ANRS Prevenir study in Paris region with daily or on‐demand PrEP with TDF/FTC

**J.‐M. Molina^1^; J. Ghosn^2^; L. Béniguel^3^; D. Rojas‐Castro^4^; M. Algarte‐Genin^3^; G. Pialoux^5^; C. Delaugerre^1^; Y. Yazdanpanah^6^; C. Katlama^7^; C. Ségouin^8^; S. Morel^9^; C. Pintado^10^; B. Loze^10^; S. Le Mestre^11^; S. Gibowski^11^; V. Doré^11^; L. Assoumou^3^; B. Spire^12^; D. Costagliola^3^ and Prevenir ANRS study group**

^1^University of Paris Diderot, Sorbonne Paris Cité, Saint‐Louis Hospital, Assistance Publique Hôpitaux de Paris, Paris, France, ^2^Paris Descartes University, Sorbonne‐Paris‐Cité, Hotel‐Dieu Hospital, Assistance Publique Hôpitaux de Paris, Paris, France, ^3^INSERM, Sorbonne Université, Institut Pierre Louis d’Épidémiologie et de Santé Publique (IPLESP), Paris, France, ^4^Coalition PLUS, Pantin, France, ^5^Sorbonne Université, Tenon Hospital, Assistance Publique Hôpitaux de Paris, Paris, France, ^6^INSERM, University of Paris Diderot, Sorbonne Paris Cité, IAME (Infection, Antimicrobials, Modelling, Evolution), Bichat Hospital, Assistance Publique Hôpitaux de Paris, Paris, France, ^7^INSERM, Sorbonne Université, Institut Pierre Louis d’Épidémiologie et de Santé Publique (IPLESP), Pitié‐Salpétrière Hospital, Assistance Publique Hôpitaux de Paris, Paris, France, ^8^Lariboisière Hospital, Assistance Publique Hôpitaux de Paris, Paris, France, ^9^AIDES, Pantin, France, ^10^Saint‐Louis Hospital, Assistance Publique Hôpitaux de Paris, Paris, France, ^11^ANRS, France Recherche Nord & Sud Sida‐hiv Hépatites, Agence autonome de l'INSERM, Paris, France, ^12^Aix Marseille Univ, INSERM, IRD, SESSTIM, Sciences Economiques and Sociales de la Santé and Traitement de l'Information Médicale, ORS PACA, Observatoire Régional de la Santé Provence‐Alpes‐Côte d'Azur, Marseille, France

**Background:** On‐demand PrEP with TDF/FTC has been recommended as an alternative to Daily PrEP for MSM by the European AIDS Clinical Society following the results of clinical studies but data are limited on real‐world experience.

**Methods:** The ANRS Prevenir study is an ongoing prospective cohort study in the Paris region enrolling high risk individuals willing to/or using PrEP. Both daily and On‐demand PrEP were offered to eligible individuals. At baseline, month 1 and every three months thereafter subjects were tested for HIV using a fourth generation combined ELISA test and other STIs and creatinine plasma levels were monitored. At each visit participants provided information regarding sexual behaviour and adherence using computer assisted self‐interviews. Analysis of HIV incidence was assessed to provide additional data on the efficacy and safety of the two PrEP dosing regimens.

**Results: ** From 3 May 2017 to 1 May 2018, 1435 subjects were enrolled across 22 sites, 59% being PrEP experienced for a median of 10 months. Median age was 37 years (IQR: 30 to 44), 98.7% were MSM. At enrolment, PrEP was used Daily in 44% and On‐demand in 53% of participants. Median number of partners in the three months before enrolment was 15 (IQR: 7 to 25) in the Daily group and 10 (5 to 15) in the On‐demand group (*p* < 0.001). Median number of condomless sex in the prior four weeks was 3 (1 to 8) and 2 (0 to 4), respectively, *p* < 0.001. The current follow‐up lasted 302 and 361 person‐years (PY) in the Daily and On‐demand groups, respectively. The incidence of HIV‐1 infection was 0 (95% CI: 0 to 1.2) per 100 PY and 0 (95% CI: 0 to 1.0) in the Daily and On‐demand groups respectively (*p* = 1.00) and the incidence of study discontinuation was 3.0 and 3.6 per 100 PY (*p* = 0.674) respectively, including 1.3 and 1.1 per 100 PY drop out of PrEP because participants no longer feel at risk. No participant discontinued PrEP for drug‐related adverse events.

**Conclusions: **In this ongoing PrEP cohort in Paris region, enrolling mainly MSM at high risk of HIV‐acquisition, no breakthrough HIV‐infection was reported so far with either daily or on‐demand PrEP, supporting continuing use of both dosing regimens in this population.

## WEAE0508LB

### Taking biometric coding and the patient‐linkage and retention system to scale: Game changers for improving patient tracking and reducing loss to follow‐up (LTFU) among PLHIV in Haiti

**L. Hall^1^; M.P.J.S. Valles^2^; R. Jean‐Francois^3^; P. Joseph^2^; M. Antoine^2^; N. Celestin^2^; G. Perrin^4^; S. Morisseau^3^ and A. Leggoe^3^**

^1^Centers for Disease Control and Prevention, Atlanta, United States, ^2^Centers for Disease Control and Prevention, Port‐au‐Prince, Haiti, ^3^United States Agency for International Development, Port‐au‐Prince, Haiti, ^4^Centers for Disease Control and Prevention, Port‐au‐Prince, Heard Islands and McDonald Islands

**Background:** In 2015, after identifying an alarming rate of LTFU (15% for FY14) among PLHIV enrolled on treatment, the PEPFAR Haiti program implemented a multi‐faceted intervention to improve patient tracking, LTFU retrieval, adherence, and retention: fingerprint‐based Biometric Coding (BC) and the Patient Linkage and Retention (PLR) System.

**Methods:** PLR encompasses tracking of patients at the community level, including HIV+ patients never linked to care, LTFU, and active patients at risk of being LTFU. PLR is optimized with GPS and BC, allowing for a unique identifier for HIV+ patients, improved estimates of the never‐diagnosed PLHIV, and identification of silent transfers and medical shopping. Details of eligible patients are provided to community health workers (CHWs) who conduct home visits.

**Results: ** To date, PLR and BC have been rolled‐out in 90% of clinical facilities, covering 72% of PLHIV enrolled on ART. 55% of LTFU have been brought back to care with 57% of retention. The overall annual LFTU rate has decreased from 15% in FY14 to 9% in FY17. Barriers to retention, documented through the PLR tool, have been incorporated to address programmatic gaps in patient linkage and retention. Moreover, GPS and BC facilitate data quality assurance by identifying duplicate patients and medical shopping across Haiti. BC, PLR and the CHW network also facilitate data‐driven partne

**Conclusions: **While the validity of BC connected to patient tracking has been shown effective at improving HIV treatment outcomes in other geographically limited settings, PEPFAR‐Haiti is demonstrating that BC taken to scale in a resource‐constrained setting and applied at the national level, improves clinical outcomes, de‐duplicating national figures of PLHIV on ART and tracking patterns of LTFU and re‐entry across Haiti. BC and PLR are an important factor in decreasing LTFU. PEPFAR‐Haiti will continue to take this innovation to scale to all supported facilities for improved program management to achieve epidemic control in Haiti.

## WEAX0101LB

### Engagement in methadone maintenance therapy associated with less time with plasma HIV‐1 RNA viral load above 1500 copies/mL among a cohort of HIV‐positive people who use drugs in Vancouver, Canada

**B. Barker^1,2^; C. Fairgrieve^1^; K. Ahamad^1,3,4^; T. Kerr^1,5^; J. Shoveller^6^; J. Montaner^5,7^; E. Wood^1,5^ and M.‐J. Milloy^1,5^**

^1^BC Centre on Substance Use, Vancouver, Canada, ^2^University of British Columbia, Interdisciplinary Studies Graduate Program, Vancouver, Canada, ^3^Providence Health Care, Department of Family and Community Medicine, Vancouver, Canada, ^4^University of British Columbia, Department of Family Medicine, Vancouver, Canada, ^5^University of British Columbia, Department of Medicine, Division of AIDS, Vancouver, Canada, ^6^University of British Columbia, School of Population and Public Health, Vancouver, Canada, ^7^BC Centre for Excellence in HIV/AIDS, Vancouver, Canada

**Background:** It is well established that elevated plasma HIV‐1 RNA viral load (VL) drives the risk of onward viral transmission. Despite being a key population living with HIV, people who inject drugs continue to experience individual, social and structural barriers in accessing and being retained in HIV treatment and care. In the present study, we sought to longitudinally examine the relationship between engagement in a low‐threshold methadone maintenance therapy (MMT) program and amount of person‐time with heightened HIV transmission risk (i.e. VL >1500 copies/mL plasma) among HIV‐positive people who use drugs (PWUD).

**Methods:** Data were derived from the AIDS Care Cohort to evaluate Exposure to Survival Services (ACCESS), a community‐recruited prospective cohort of HIV‐positive PWUD in Vancouver, Canada. Longitudinal cohort data was confidentially linked to comprehensive HIV clinical monitoring records in a setting of universal no‐cost HIV treatment and care. We used generalized estimating equation analyses to assess the impact of engagement in low‐barrier MMT on the number of days with an HIV‐1 RNA VL above 1500 copies/mL in the previous 180 days.

**Results: ** Between 5 December 2005 and 29 November 2017, 867 HIV‐seropositive antiretroviral therapy‐exposed PWUD were recruited and contributed 4531 person‐years of observation time. Among these, 522 (60.2%) were engaged in MMT at least once during follow‐up. In a multivariable model, periods of MMT were independently associated with fewer days with a VL above 1500 copies/mL (Adjusted Rate Ratio=0.70, 95% Confidence Interval: 0.60 to 0.81), after controlling for demographics, drug use patterns, and CD4 count.

**Conclusions: **We observed that engagement in MMT was associated with significantly less person‐time with a VL above 1500 copies/mL among a large and long running cohort of PWUD. These findings suggest that low‐threshold MMT is an effective intervention in lowering the risk of onward viral transmission among this key population. Further, these findings demonstrate the important role of evidence‐based addiction treatment in optimizing individual and community‐level impacts of antiretroviral therapy among HIV positive patients with comorbid opioid dependence. Efforts to address barriers to the use and availability of MMT will likely improve HIV outcomes and reduce new infections among this population and should therefore be prioritized.

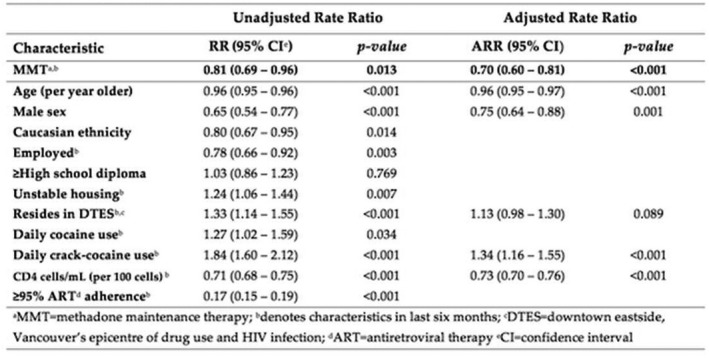

**Abstract WEAX0101LB‐Table 1. Generalized estimating equation analyses of factors associated with person‐time exceeding an HIV‐1 RNA viral load above 1500 copies/mL plasma.**

## WEAX0102LB

### Universal test and treat (UTT) versus standard of care for access to antiretroviral therapy in HIV clients: The MaxART stepped‐wedge randomized controlled health systems trial in Swaziland

**S. Khan^1^; D. Spiegelman^2^; F. Walsh^3^; S. Mazibuko^4^; M. Pasi^4^; B. Chai^2^; R. Reis^5,6,7^; K. Mlambo^1^; W. Delva^8,9,10^; G. Khumalo^11^; M. Zwane^12^; Y. Fleming^13^; E. Mafara^1^; A. Hettema^1^; C. Lejeune^1^; T. Bärnighausen^2,14^ and V. Okello^4^**

^1^Clinton Health Access Initiative (CHAI), Mbabane, Swaziland, ^2^Harvard T.H. Chan School of Public Health, Boston, United States, ^3^Clinton Health Access Initiative (CHAI), Boston, United States, ^4^Ministry of Health, Mbabane, Swaziland, ^5^Leiden University, Leiden University Medical Center, Leiden, Netherlands, ^6^University of Amsterdam, Amsterdam Institute for Social Science, Amsterdam, Netherlands, ^7^University of Cape Town, Children's Institute, Cape Town, South Africa, ^8^Stellenbosch University, The South African Department of Science and Technology ‐ National Research Foundation (DST‐NRF) Centre of Excellence in Epidemiological Modelling and Analysis (SACEMA), Stellenbosch, South Africa, ^9^Hasselt University, Center for Statistics, Diepenbeek, Belgium, ^10^Ghent University, Ghent, Belgium, ^11^Swaziland National Network of People Living with HIV/AIDS (SWANNEPHA), Mbabane, Swaziland, ^12^SAfAIDS, Mbabane, Swaziland, ^13^aidsfonds, Amsterdam, Netherlands, ^14^Heidelberg Institute of Public Health, University of Heidelberg, Heidelberg, Germany

**Background:** The World Health Organization recommends offering antiretroviral treatment (ART) to all HIV‐positive individuals regardless of CD4 count or disease stage, known as “universal test and treat” (UTT). However, the health systems effects of UTT implementation are unknown. We investigated the effect of UTT on retention and viral suppression in the world's first UTT implementation trial in a government‐managed health system.

**Methods:** In this stepped‐wedge randomized controlled trial, fourteen public sector health facilities in Swaziland were paired and randomly assigned to transition in four‐month steps from implementing the current national standard of care (SoC) to providing ART under UTT. ART‐naïve clients ≥18 years who were not pregnant or breastfeeding were eligible for enrollment. We used Cox proportional hazard models with censoring of follow‐up at clinic transition to measure the effects of UTT on our two primary endpoints: retention and viral suppression after ART initiation. The trial is registered with clinicaltrials.gov (NCT02909218).

**Results: ** Between September 2014 and August 2017, 3405 clients (62% women, median age 33 years (IQR: 28 to 42)) were enrolled. Under SoC, 12‐month retention and post ART initiation viral load suppression rates were 80% (95% CI): 77 to 83) and 4% (95% CI: 2 to 7), respectively, compared to 86% (95% CI: 83 to 88) and 79% (95% CI: 75 to 83) under UTT. 75% of clients were missing viral load at the six‐month time window following ART initiation; they were considered unsuppressed. Compared to SoC, UTT had a modest effect on retention (hazard ratio (HR) 1.60, 95% CI 1.15 to 2.21) and a large effect on viral suppression among those retained six months after ART initiation (HR: 14.51, 95% CI: 7.31 to 28.79) (Table 1). The UTT effect on the combined endpoint of retention and viral suppression was also substantial (HR 4.88, 95% CI 2.96 to 8.05).

**Conclusions: **Adopting UTT improves the performance of the health system in providing ART to people living with HIV. The observed improvement in retention and viral suppression, key indicators of ART success, provides an important co‐benefit of UTT. Our results from this “real world” health systems trial strongly support the scale‐up of UTT in Swaziland and countries with similar HIV epidemics and health systems.

**Abstract WEAX0102LB‐Table 1. Primary endpoints for MaxART universal test and treat health systems trial**

**Table:** 

Endpoint	Crude HR (95% CI)	*p*‐value	Adjusted HR (95% CI)	*p*‐value
Retention	1.60 (1.15 to 2.21)	0.005	1.94 (1.33 to 2.82)	0.0006
Viral suppression	14.51 (7.31 to 28.79)	<0.0001	22.08 (7.91 to 61.59)	<0.0001
Combined endpoint (retention and viral suppression)	4.88 (2.96 to 8.05)	<0.0001	6.90 (3.11 to 15.31)	<0.0001

## WEAX0103LB

### Men's HIV risk profiles in South African DREAMS sites: Using latent class analysis for more strategic, context‐specific programming and evaluation

**A. Gottert^1^; C.J. Heck^2^; S. Mathur^1^ and J. Pulerwitz^3^**

^1^Population Council, HIV and AIDS Program, DREAMS IS, Washington, DC, United States, ^2^Population Council, Poverty, Gender, and Youth Program, New York, United States, ^3^Population Council, HIV and AIDS Program, Washington, DC, United States

**Background:** There is a critical need to reach high‐risk men with HIV prevention, care, and treatment services. Latent class analysis (LCA) can generate context‐specific profiles based on demographic, attitudinal, and behavioral indicators – which can then be used to refine program targets and assess service uptake.

**Methods:** From May to September 2017, we conducted surveys with 962 men ages 20 to 40 in informal settlements in Durban, South Africa where the DREAMS Partnership is being implemented. Using LCA, we identified classes based on sociodemographics, normative gender attitudes, and HIV risk behaviors. We then assessed associations between class membership and HIV service use.

**Results: ** We identified four latent classes (Table 1), with good model fit statistics. The younger moderate‐risk class (36% of the sample; mean age 23) were largely unmarried, recent technical college/university graduates, unemployed, and moderately gender‐inequitable. They had high numbers of same‐age partners, low‐end transactional relationships, and some hazardous drinking. The younger high‐risk class (25%; mean age 27) were unmarried, employed secondary graduates with the highest number of partners and the most inequitable gender views, hazardous drinking, and low‐end transactional relationships. The older low‐risk class (20%; mean age 30) were largely married/cohabiting, employed secondary graduates and were the most gender‐equitable, with few partners, limited transactional sex and relatively low hazardous drinking. Finally, the older high‐risk class (20%; mean age 36) were largely married/cohabiting, employed secondary graduates, and moderately gender‐inequitable. They had highly age‐disparate partners and substantial high‐end transactional relationships and hazardous drinking. High‐risk men tended to be taxi drivers, factory/construction workers, or small business owners/entrepreneurs. Uptake of voluntary medical male circumcision was much higher among the younger moderate‐risk class than the others (all *p *<* *0.001). The older low‐risk class had higher HIV treatment literacy than the younger high‐risk class (*p *<* *0.01). HIV testing and current use of antiretroviral therapy did not differ across the classes.

**Conclusions: **We identified distinct HIV risk profiles among men in Durban. Interventions should focus on reaching the highest‐risk profiles who, despite their elevated risk, were less likely or no more likely than the lower‐risk to use HIV services. LCA has the potential to enable more strategic, data‐driven programming and evaluation.

**Abstract WEAX0103LB‐Table 1.HIV risk profiles among men (n = 962)**

**Table:** 

Indicators	Younger moderate risk (35.8% of sample)	Younger high risk (24.7% of sample)	Older low risk (19.5% of sample)	Older high risk (20.1% of sample)	Full sample
**Sociodemographic**					
Age (mean)	22.5 years	27.2 years	29.5 years	35.9 years	27.7 years
Married/cohabiting	3.9%	7.5%	26.0%	35.7%	15.5%
Educ. (last compl.)					
Some second or less	16.5%	20.0%	24.9%	35.3%	22.8%
Secondary	51.3%	66.3%	60.1%	47.6%	56.0%
Tech college/Univ	32.2%	13.6%	15.0%	17.1%	21.2%
**Occupation**					
Unemployed	73.6%	21.4%	21.0%	15.5%	38.9%
Taxi/bus driver	11.4%	35.6%	31.3%	30.4%	25.0%
Fact./constr. worker	2.4%	12.3%	7.3%	11.2%	7.5%
Informal labor	1.3%	4.9%	10.9%	7.7%	5.3%
Service industry	3.5%	5.7%	10.4%	11.1%	6.9%
Sm. bus./entrep.	1.8%	9.2%	2.4%	8.2%	5.0%
Other occupation	6.1%	11.0%	16.7%	15.8%	11.3%
**Normative gender attitudes**					
Inequitable views toward gender norms^a^	25.3%	38.4%	6.9%	26.0%	51.8%
**HIV risk behaviors**					
# sexual partners in last year					
0 to 1	27.8%	4.8%	57.7%	33.1%	29.0%
2 to 4	49.3%	53.3%	35.6%	47.2%	47.2%
5+	22.9%	41.9%	6.6%	19.7%	23.8%
Age diff w/ last 3 partners(mean)^b^	1.0 years	3.6 years	3.2 years	7.4 years	3.6 years
**Transactional relationships** ^c^					
None	49.4%	6.8%	78.3%	48.6%	44.4%
Low‐end	44.6%	75.9%	11.9%	31.7%	43.4%
High‐end	6.0%	17.3%	9.8%	19.6%	12.3%
Hazardous drinking^d^	41.0%	72.0%	39.9%	58.5%	51.8%
***HIV SERVICE UPTAKE, by latent class membership***	Younger moderate risk	Younger high risk	Older low risk	Older high risk	Overall *p*‐value^f^
Tested for HIV in last 12 months	74.7%	69.6%	67.4%	70.2%	>0.05
Received VMMC in last 5 years	42.6% (ref)	18.8%***	13.1%***	8.6%***	<0.001
HIV treatment literacy score (range 0 to 5)^e^	3.57	3.36**	3.70 (ref)	3.57	<0.01
Currently taking antiretroviral therapy (n = 84)	90.1%	89.5%	94.7%	90.6%	>0.05

Fit statistics for this LCA model: AIC=22,122; BIC=22,478 ^a^Measured by a 19‐item Gender Equitable Men's (GEM) Scale (Cronbach's alpha=0.87), with binary cut‐point at midpoint of range. ^b^Mean number of years up to last three partners were younger than respondent. ^c^Low‐end transactional relationships include providing various low‐cost goods or services like cash, food, or transportation mainly to start or stay in a relationship; high‐end transactional relationships include paying for things like housing, debt or school fees mainly to start or stay in a relationship. ^d^Assessed by AUDIT‐C measure. ^e^Composite score of 5 items assessing correct knowledge about the attributes and benefits of HIV treatment. ^f^ *p*‐value represents overall statistical significance of difference between groups, based on Pearson's chi‐square test. *p <* *0.05, **p <* *0.01, ***p <* *0.001; significance of pairwise comparisons with the reference category (ref).

## WEAX0104LB

### Risk of HIV transmission through condomless sex in MSM couples with suppressive ART: The PARTNER2 Study extended results in gay men

**A. Rodger^1^; V. Cambiano^1^; T. Bruun^2^; P. Vernazza^3^; S. Collins^4^; G.M. Corbelli^5^; O. Degen^6^; V. Estrada^7^; A.M. Geretti^8^; A. Beloukas^8^; A.N. Phillips^1^; J. Lundgren^2^ and for the PARTNER Study Group**

^1^University College London, London, United Kingdom, ^2^University of Copenhagen, Copenhagen, Denmark, ^3^Cantonal Hospital, St. Gallen, Switzerland, ^4^HIV i‐Base, London, United Kingdom, ^5^European AIDS Treatment Group, Bruxelles, Belgium, ^6^University Medical Center, Hamburg, Germany, ^7^Hospital Clinico San Carlos & Universidad Complutense, Madrid, Spain, ^8^University of Liverpool, Liverpool, United Kingdom

**Background:** Although zero cases of HIV transmission in gay men have been reported in observational studies (PARTNER1 and Opposites Attract) of serodifferent couples where the positive person was on suppressive ART, the level of evidence for gay men remained less than for heterosexual couples. The aim of PARTNER2 was to provide more precise estimates of transmission risk through condomless‐sex in serodifferent gay male couples where the HIV‐positive partner was on suppressive ART.

**Methods:** The PARTNER Study was a prospective observational study in 14 European countries. Phase 1 (Sep 2010‐May 2014) recruited both heterosexual and gay male couples, and Phase 2 (to April 2018) recruited and followed gay couples only. Study data, collected at baseline and every six to twelve months, included sexual behaviour questionnaires with HIV testing (HIV‐negative partner) and HIV‐1 viral‐load (HIV‐positive partner). Eligibility criteria for CYFU inclusion were condomless sex, no reported PEP or PrEP use, and most recent plasma HIV‐1 RNA load <200 copies/mL in the last year. If a seroconversion occurred, anonymised phylogenetic analysis compared HIV‐1 polymerase and envelope sequences in both partners to identify linked transmissions.

**Results: ** Between Sep 2010 and July 2017, 972 gay couples were enrolled. Of these, 779 couples provided 1561 eligible CYFU over a median of 1.6 years (IQR 0.8 to 2.8). At baseline, mean age was 40 years (IQR 33 to 46) and couples reported condomless‐sex for a median of 1.0 years (IQR 0.4 to 2.9). During eligible CYFU, a total of 74,567 condomless‐sex acts were reported, a median of 42 times per couple year (IQR 19 to 74). Condomless‐sex with other than the main partner was reported by 285 HIV‐negative men (37%). There were 17 new HIV infections, but none were phylogenetically linked transmissions, giving a precise rate of within‐couple HIV transmission of zero, with a narrow upper 95% confidence limit of 0.24/100 CYFU. This upper‐limit for condomless anal sex with ejaculation was 0.59/100 CYFU.

**Conclusions: **Despite almost 75,000 condomless‐sex acts in gay serodifferent couples where the positive partner was on suppressive ART, we found zero cases of within couple HIV transmission. PARTNER2 provides a similar level of confidence for gay men as for heterosexual couples in PARTNER1.

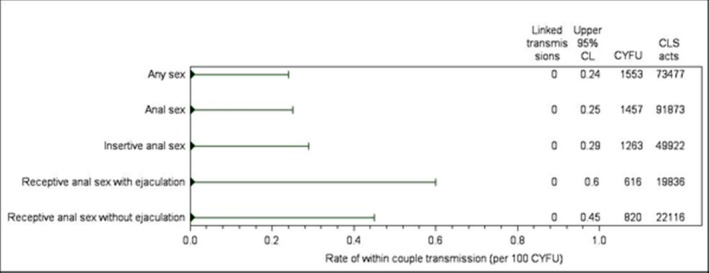

**Abstract WEAX0104LB‐Figure 1. Rate of HIV Transmission According to Sexual Behavior Reported by the HIV‐Negative Partner.**

## WEAX0105LB

### Impact of prevention and treatment interventions on population HIV incidence: Primary results of the community‐randomized Ya Tsie Botswana prevention project

**M.J. Makhema^1^; K. Wirth^2^; M. Pretorius Holme^2^; T. Gaolathe^1^; M. Mmalane^1^; E. Kadima^1^; U. Chakalisa^1^; K. Manyake^1^; A. Mbikiwa^1^; S. Simon^1^; R. Letlhogile^1^; K. Mukokomani^1^; E. van Widenfelt^1^; S. Moyo^1^; K. Bennett^3^; J. Leidner^4^; R. Lebelonyane^5^; M.G. Alwano^6^; K. Powis^1,2,7^; S. Dryden‐Peterson^1,2,8^; C. Kgathi^1^; V. Novitsky^2^; J. Moore^9^; P. Bachanas^9^; W. Abrams^6^; L. Block^9^; S. El‐Halabi^5^; T. Marukutira^6^; L.A. Mills^6^; H. Bussmann^1^; L. Okui^1^; O. John^1^; R. Shapiro^2^; V. DeGruttola^2^; Q. Lei^2^; R. Wang^2^; E. Tchetgen Tchetgen^2,10^; M. Essex^1,2^ and S. Lockman^1,2,8^**

^1^Botswana Harvard AIDS Institute Partnership, Gaborone, Botswana, ^2^Harvard T.H. Chan School of Public Health, Boston, United States, ^3^Bennett Statistical Consulting, Inc., Ballston Lake, United States, ^4^Goodtables Data Consulting, LLC, Norman, United States, ^5^Botswana Ministry of Health, Gaborone, Botswana, ^6^Centers for Disease Control and Prevention, Gaborone, Botswana, ^7^Massachusetts General Hospital, Boston, United States, ^8^Brigham and Women's Hospital, Boston, United States, ^9^U.S. Centers for Disease Control and Prevention, Atlanta, United States, ^10^The Wharton School, University of Pennsylvania, Philadelphia, United States

**Background:** Antiretroviral therapy(ART) markedly reduces incidence in known HIV‐discordant relationships. However, the impact of expanded access to HIV testing/counseling(HTC), ART, and male circumcision(MC) on community HIV incidence is unknown, particularly in settings with both high HIV prevalence and high baseline ART coverage such as Botswana.

**Methods:** The Ya Tsie Botswana Prevention Project was a pair‐matched community‐randomized trial that evaluated the impact of prevention interventions on HIV incidence in 30 rural/semi‐urban communities throughout Botswana, from 2013 to 2018. Fifteen communities were randomized to receive community‐wide HTC, linkage‐to‐care, earlier ART initiation, and enhanced MC services, and 15 communities received standard of care. Universal ART became standard of care in both arms mid‐2016. A random sample of ˜20% of households in each community was selected, and HIV‐uninfected 16 to 64 year‐old residents of these households enrolled in a longitudinal HIV incidence cohort (HIC) that underwent ˜annual HTC. We compared HIV incidence by randomized arm over ˜30 months. The pre‐specified primary analysis used a permutation test of inverse variance weighted average of log‐ transformed incidence ratios from pair‐specific, interval‐censored Cox proportional hazards models (PHM); 95% CIs were obtained using standard pair‐stratified Cox PHM for interval censored data. *p*‐values are two‐sided.

**Results: ** Among 12,610 participants, at baseline 29% were HIV‐infected, 72% of whom were already on ART (97% of individuals on ART had HIV‐1 RNA<400 copies/mL). We enrolled 8,974 HIV‐uninfected individuals in the HIC (4,487/arm), with median age 29 years (60% female). The median duration of follow‐up was 29 months, and 95% of participants in each arm re‐tested for HIV at ≥1 follow‐up visit. 57 HIC participants in the intervention arm (annualized HIV incidence: 0.59%) and 90 in the control arm (annualized HIV incidence: 0.92%) acquired HIV. The HIV incidence ratio was 0.69 (*p *=* *0.09) in intervention vs. standard‐of‐care communities in the primary weighted‐average Cox PHM. The pair‐stratified Cox PHM produced 95% CI of 0.46 to 0.90 (incidence ratio=0.65, *p *=* *0.01).

**Conclusions: **We observed a 30% reduction in community HIV incidence with expanded HTC, linkage, ART, and MC campaigns. Importantly, our findings demonstrate that it is possible to reduce HIV incidence in high‐HIV‐prevalence settings that have already approached the ambitious UNAIDS 90‐90‐90 targets, by further increasing coverage.

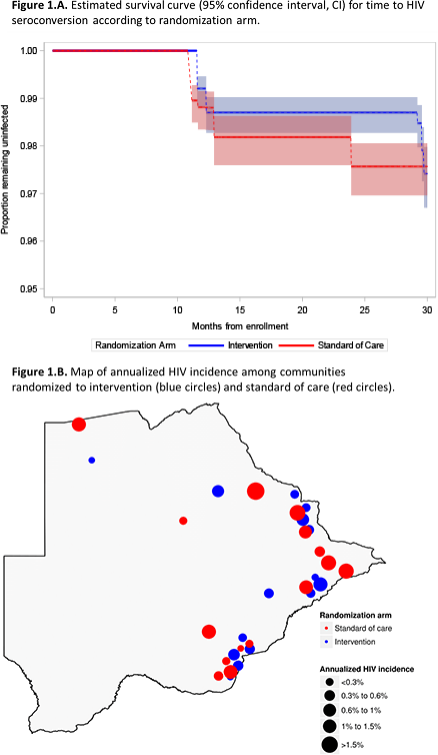

**Abstract WEAX0105LB‐Figure 1.**

## WEAX0106LB

### SEARCH community cluster randomized study of HIV “test and treat” using multi‐ disease approach and streamlined care in rural Uganda and Kenya

**D. Havlir^1^; E. Charlebois^1^; L. Balzer^2^; T. Clark^1^; D. Kwarisiima^3^; J. Ayieko^4^; J. Kabami^3^; N. Sang^4^; T. Liegler^1^; G. Chamie^1^; C. Camlin^1^; K. Kadede^4^; A. Mucunguzi^3^; V. Jain^1^; T. Ruel^1^; S. Shade^1^; E. Ssemondo^3^; D. Byonanebye^3^; F. Mwangwa^3^; A. Owaraganise^3^; W. Olilo^4^; D. Black^1^; K. Snyman^1^; R. Burger^1^; M. Getahun^1^; J. Achando^4^; B. Awuonda^4^; H. Nakato^3^; J. Kironde^3^; H. Thirumurthy^5^; C. Koss^1^; L. Brown^1^; C. Marquez^1^; J. Schwab^6^; G. Lavoy^3^; A. Plenty^1^; E. Mugoma Wafula^4^; P. Omanya^4^; J. Rooney^7^; M. Bacon^8^; M. van der Laan^6^; C. Cohen^1^; E. Bukusi^4^; M. Petersen^6^; M. Kamya^3^ and SEARCH Collaboration**

^1^University of California, San Francisco, Department of Medicine, San Francisco, United States, ^2^University of Massachusetts, Amherst, Amherst, United States, ^3^Infectious Diseases Research Collaboration, Kampala, Uganda, ^4^Kenya Medical Research Institute, Nairobi, Kenya, ^5^University of Pennsylvania, Philadelphia, United States, ^6^University of California, Berkeley, Berkeley, United States, ^7^Gilead Sciences, Foster City, United States, ^8^National Institutes of Health, Bethesda, United States

**Background:** SEARCH (NCT: 01864683) is a community‐cluster randomized study in rural Uganda and Kenya evaluating whether a multi‐disease, streamlined‐care approach to HIV “test and treat” reduces HIV incidence and improves community health, compared to a national guideline approach augmented with baseline HIV and NCD (hypertension and diabetes) testing.

**Methods:** We randomized (2013 to 2014) 32 pair‐matched communities in 3 regions (Uganda‐ West, Uganda‐East, Kenya) to an active control with baseline HIV/NCD testing and ART/NCD care by national guidelines or an intervention with additional annual population HIV/NCD testing, rapid‐start ART for all HIV+, and patient‐centered, streamlined ART/NCD care. In all communities, population‐level testing was delivered through multi‐disease health fairs; non‐ participants were tested at home/in community. In the control arm, ART eligibility expanded during the study from CD4 + < 350 to CD4 + < 500 to all HIV+. Three‐year HIV cumulative incidence, HIV viral suppression, mortality, HIV‐TB, and hypertension control were compared between arms using targeted maximum likelihood estimation.

**Results: ** At baseline, 335,005 persons (150,395 adults ≥15 years) were enrolled; 90.1% of adults were HIV‐tested. HIV prevalence was 6.6%, 3.5%, and 19.3%, and HIV viral suppression was 47.5%, 43.0%, and 52.8% in Uganda‐West, Uganda‐East, and Kenya, respectively. Population‐level viral suppression increased to 73.0% by year 1 in the intervention arm; at year 3, suppression was higher in intervention (79.7%) vs. control (68.4%) (RR: 1.17; 95% CI: 1.11,1.22; *p* < 0.001). At year 3, the intervention arm had 21% lower mortality among HIV+ (RR: 0.79; 95% CI: 0.65,0.96; *p* = 0.02), 59% lower annual TB incidence among HIV+ (RR: 0.41; 95% CI: 0.19,0.86; *p* = 0.02), and 16% less uncontrolled hypertension (RR: 0.84; 95% CI: 0.79,0.90; *p *<* *0.001) compared to control. Annual HIV incidence in the intervention arm decreased from year 1 to year 3 by 30% (RR: 0.70; 95% CI: 0.57,0.86; *p* < 0.001); incidence decreased by 45% in Kenya (RR: 0.55; 95% CI: 0.40,0.76; *p* < 0.001). Three‐year cumulative HIV incidence did not differ between intervention (0.77%) and control (0.81%) (RR: 0.95; 95% CI: 0.77,1.17; *p* = 0.60).

**Conclusions: **A multi‐disease disease approach using streamlined care rapidly achieved UNAIDS 90‐90‐90 targets, improving community health (HIV mortality, HIV‐TB, hypertension control). Annual HIV incidence decreased by 30% during the study; however, three‐year cumulative HIV incidence did not differ between arms.

Continued investment and innovation in HIV treatment and prevention are needed for HIV elimination.

## THAB0108LB

### Superiority of paclitaxel compared to either bleomycin/vincristine or etoposide as initial chemotherapy for advanced AIDS‐KS in resource‐limited settings: A multinational randomized trial of the ACTG and the AIDS Malignancy Consortium

**M. Borok^1^; C. Moser^2^; T. Campbell^3^; P. MacPhail^4^; R. Matining^5^; S. Caruso^6^; C. Godfrey^7^; A. Moses^8^; W. Samaneka^9^; M. Nyirenda^10^; N. Busakhala^11^; H. Burger^12^; N. Mwelase^4^; B. Hoagland^13^; J. Kosgei^14^; J. Orem^15^; M. Nokta^16^; V. Otieno^17^; R. Mngqibisa^18^; S. Krown^19^ and A5263/AMC‐066 team**

^1^University of Zimbabwe College of Health Sciences, Department of Medicine, Harare, Zimbabwe, ^2^Harvard TS Chan School of Public Health, Statistical & Data Analysis Center, Boston, United States, ^3^University of Colorado, Denver, Division of Infectious Diseases, Denver, United States, ^4^University of the Witwatersrand, Medicine, Johannesburg, South Africa, ^5^Harvard T.H. Chan School of Public Health, Statistical & Data Analysis Center, Boston, United States, ^6^Frontier Science & Technology Research Foundation, Inc., Amherst, United States, ^7^National Institutes of Health, Division of AIDS, Therapeutics Research Program, Washington DC, United States, ^8^Kamuzu Central Hospital, Lilongwe, Malawi, ^9^Parirenyatwa CRS, Harare, Zimbabwe, ^10^Blantyre CRS, John Hopkins Research Project, Blantyre, Malawi, ^11^Moi University Clinical Research Center, Oncology, Eldoret, Kenya, ^12^Tygerberg Hospital, Family Clinical Research Unit, Cape Town, South Africa, ^13^Instituto de Pesquisa Clinica Evandro Chagas, Rio de Janeiro, Brazil, ^14^Kenya Medical Research Institute, Kericho, Kenya, ^15^Uganda Cancer Institute, Kampala, Uganda, ^16^National Cancer Institute, Bethesda, United States, ^17^Kisumu Clinical Research Site, Kisumu, Kenya, ^18^Durban International CRS, Enhancing care foundation, Durban, South Africa, ^19^AIDS Malignancy Consortium, New York, United States

**Background:** Advanced Kaposi sarcoma (KS) is a potentially life‐threatening complication of HIV infection where access to chemotherapy is limited and HIV and KS herpesvirus coinfection rates are high. No evidence‐based standard of care (SOC) guideline for advanced KS exists in settings where resources for safe preparation and administration of intravenous (IV) chemotherapy are scarce.

**Methods:** Participants at 11 ACTG sites in 5 sub‐Saharan African countries and Brazil with measurable, previously‐untreated, biopsy‐proven, advanced (T1 stage) AIDS‐related KS, adequate organ function and performance status, and limited (≤42 days) or no ART exposure were prospectively randomized 1:1:1 to receive oral etoposide plus ART (ET+ART), IV bleomycin and vincristine+ART (BV+ART), or IV paclitaxel+ART (PTX+ART). Randomization was stratified by CD4 count (<100 or ≥100 cells/mm^3^) and country. PTX+ART, a standard regimen in resource‐rich settings, was considered the active control. The trial was designed to evaluate whether ET+ART (an oral regimen affording significant logistical advantages in resource‐limited settings (RLS)) and/or BV+ART (a commonly‐used regimen in RLS) were noninferior to PTX+ART. Noninferiority was defined as a week‐48 progression‐free survival (PFS) rate within 15% of the PFS rate of the PTX+ART arm, projected at 65% and based on Kaplan‐Meier methods.

**Results: ** Entry and on‐study characteristics are shown in Table 1. An interim Data and Safety Monitoring Board (DSMB) review in 3/2016 found the ET+ART arm inferior to the PTX+ART arm. The ET arm was closed; accrual continued for the two remaining arms. A subsequent interim DSMB review in 3/2018 found the BV+ART arm inferior to the PTX+ART arm; further accrual was halted. PTX was offered to all remaining eligible study participants. Week‐48 PFS rates (95% CI) at the time each study arm was closed were 19% (8, 35), 43% (34, 53) and 63% (54, 72), respectively for the ET+ART, BV+ART and PTX+ART arms (Fig.1). There were no safety concerns about any of the treatment regimens; ˜90% had HIV VL <400 copies/mL by week 12.

**Conclusions: **These findings establish PTX+ART as a SOC for initial treatment of advanced AIDS‐KS, and underscore both the urgent need to improve the cancer therapeutic infrastructure and the accessibility of essential chemotherapeutic agents in RLS.

**Abstract THAB0108LB‐Table 1. Selected entry and on study characteristics**

**Table:** 

	ET+ART N = 59	BV+ART N = 125	PTX+ART N‐132	ALL PARTICIPANTS N = 316
Women	13 (22%)	29 (23%)	31 (23%)	73 (23%)
Age	35 (31, 42)	35 (30, 42)	35 (31, 40)	35 (31, 41)
Visceral KS at Entry	20 (35%)	33 (27%)	31 (24%)	84 (27%)
KS‐associated Edema at Entry	54 (95%)	116 (94%)	117 (89%)	287 (92%)
CD4 count (cells/mm^3^) at entry	194 (99, 318)	230 (134, 369)	232 (125, 348)	228 (120, 362)
@ Week 24	316 (206, 429)	268 (178, 409)	337 (201, 481)	305 (194, 451)
@ Week 48	325 (174, 407)	261 (211, 455)	290 (210, 481)	285 (210,473)
HIV VL<400 copies/mL at entry	4 (7%)	26 (21%)	34 (26%)	64 (21%)
@ Week 12	31 (89%)	96 (90%)	103 (93%)	230 (91%)

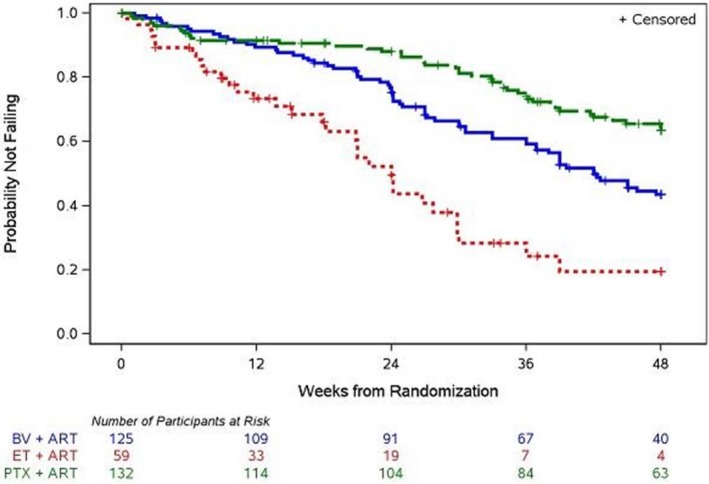

**Abstract THAB0108LB‐Figure 1. Progression‐free survival (PFS) by arm. PFS is lack of KS progression, death, entry into an additional study step, or LTFU before wk48.**

## THAB0307LB

### DolPHIN‐1: Randomised controlled trial of dolutegravir (DTG)‐ versus efavirenz (EFV)‐based therapy in mothers initiating antiretroviral treatment in late pregnancy

**C. Orrell^1^; K. Kintu^2^; J.A. Coombs^1^; A. Amara^3^; L. Myer^4^; J. Kaboggoza^2^; B. Simmons^5^; L. Else^3^; C. Heiberg^1^; C. Waitt^3^; S. Walimbwa^2^; E.M. Hodel^3^; A. Hill^3^; S. Khoo^3^; M. Lamorde^2^ and DolPHIN‐1 Study Group**

^1^Desmond Tutu HIV Foundation, Cape Town, South Africa, ^2^Infectious Diseases Institute, Kampala, Uganda, ^3^University of Liverpool, Liverpool, United Kingdom, ^4^University of Cape Town, Cape Town, South Africa, ^5^Imperial College, London, United Kingdom

**Background:** ART initiation in the third trimester of pregnancy is associated with failure to achieve viral suppression (VS) by delivery and increased transmission of HIV. We randomised 60 treatment naïve pregnant women at 28 to 36 w gestation in Uganda and South Africa 1:1 to receive EFV or DTG+2NRTIs. The primary endpoint was pharmacokinetics (PK) of DTG in women and breastfed infants; secondary endpoints included VS.

**Methods:** To comply with national guidelines, EFV+2NRTI was initiated on referral, with subjects randomized to DTG switched within seven days. Viral load (VL) was collected at every visit; intensive maternal PK sampling (0 to 24 hours) was performed at 14 days on DTG, and two weeks post‐partum, with paired sampling between maternal plasma and cord blood, breastmilk and infant plasma. All infants were exclusively breastfed.

**Results: ** There was no significant pre‐ART differences between DTG (n = 29) and EFV (n = 31) arms in maternal age, gestation at treatment initiation (30.8w), weight, obstetric history, VL (log 4 copies) and CD4 count (394 cells/mm^3^). Third trimester DTG exposures were low with Ctrough at or below target (MEC 324 ng/mL) in 9/28 (32%) mothers. DTG transfer across the placenta (122%) and in breast milk (3%) coupled with delayed elimination resulted in significant infant exposures potentially persisting during breast‐feeding. Both regimens were well‐tolerated. A total of 10 SAEs were reported in five mothers and three infants, with no significant differences between arms.

Superior VS was observed with DTG (Table 1) at the 2w post‐partum visit (*p* = 0.005). However, VL>1000 copies/mL near delivery was still observed with both DTG (3.7%) and EFV (7.4%). No HIV transmissions were observed

**Conclusions: **HIV RNA suppression <50 copies/mL was more rapid with DTG (despite low DTG exposures when started in the third trimester) which may translate to improved PMTCT for ART initiation in late pregnancy. The impact of significant infant DTG exposures related to intrauterine transfer, continued breastfeeding and delayed elimination is being evaluated in the DolPHIN‐2 study.

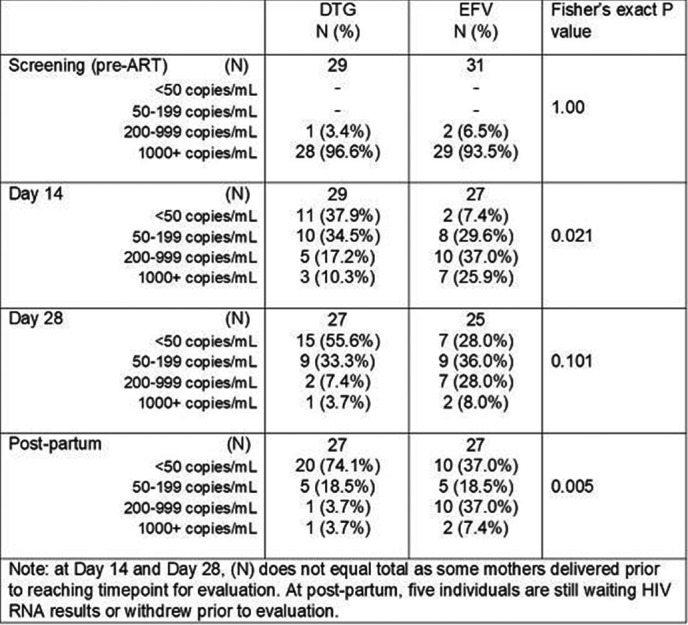

**Abstract THAB0307LB‐Table 1**

## THAC0108LB

### Using a social networking “app” to establish an open cohort for assessing HIV incidence among men who have sex with men in Beijing, China

**Z. Chen^1^; X. Chu^2^; F. Yu^1^; Y. Ma^1^; X. Xu^3^; J. McGoogan^3^; G. Mi^1,3^ and Z. Wu^3^**

^1^Blued, Beijing, China, ^2^Langfang CDC, Langfang, China, ^3^National Center for AIDS/STD Control and Prevention, Beijing, China

**Background:** HIV infection is spreading quickly among men who have sex with men (MSM) in China. Yet, MSM continue to be difficult to reach, and as a result, creating and maintaining a cohort by traditional methods is challenging. Blued is a gay social networking application (“app”) with over 40 million users worldwide and 480,000 monthly active users in Beijing. We aimed to develop an innovative new method using Blued to estimate HIV incidence among MSM.

**Methods:** All Blued users in Beijing who obtained rapid HIV screening tests via Blued (ie, online appointment booking through Blued and attendance at one of four Blued‐designated voluntary counseling and testing (VCT) clinics in Beijing)) from 21 June 2017 to 31 March 2018 were eligible for the study. Before testing, users provided inform consent electronically and completed an online survey, which collected demographic and sexual health and behavior information, through Blued. After testing, results (negative or reactive) were recorded. Users who tested more than once had their testing records linked automatically by Blued UID and the time intervals between tests were captured. Attendances with reactive results were referred to local CDC for confirmatory testing by Western blot, and all confirmed diagnoses were counted as seroconversions. All those recruited into the cohort and followed were censored at seroconversion or study end date, whichever came first, and observed time and HIV incidence rate were calculated.

**Results: ** A total of 4808 HIV screening tests were taken by 3766 Blued users, among whom 168 were HIV‐reactive and received confirmed diagnoses of HIV infection, for a point prevalence of 4.5%. The remaining 3598 were followed, among whom 673 (18.7%) accessed HIV screening again via Blued. Ten HIV seroconversions were observed during 827.7 person‐years (PY) of follow‐up time, for an incidence rate of 1.21 per 100 PY.

**Conclusions: **Our study demonstrates that use of social networking apps such as Blued provide a promising new method for evaluating and monitoring HIV incidence. Although further study is required, this method may have great potential for nationwide scale‐up to monitor HIV incidence in this important key population.

## THAC0408LB

### Progress toward HIV epidemic control: Results from the Namibia Population‐Based HIV Impact Assessment (PHIA)

**N. Hamunime^1^; A. Wolkon^2^; M. Grasso^3^; D.B. Williams^2^; A.D. Maher^3^; A. Low^4^; B. Pitt^4^; J.B. Palmier^2^; K. Banda^3^; T. Shuumbwa^1^; A. Mengistu^1^; S. Agolory^2^; G. Rutherford^3^; A.‐M. Nitschke^1^; N. Mutenda^1^; L.A. Miller^2^; S. Sawadogo^2^; O. Mwazi^5^; H. Simtaa^5^; B. Makumbi^6^; N. Pentinaiken^6^; S. Israel^6^; B. Parekh^2^; D. Prybylski^2^; K. Sachathep^4^; J. Justman^7^ and B. Haufiku^1^**

^1^Namibia Ministry of Health and Social Services, Windhoek, Namibia, ^2^U.S. Centers for Disease Control and Prevention, Division of Global HIV and TB, Atlanta, United States, ^3^University of California in San Francisco, Institute for Global Health Sciences, San Francisco, United States, ^4^ICAP at Columbia University, New York, United States, ^5^Namibia Statistics Agency, Windhoek, Namibia, ^6^Namibia Institute of Pathology, Windhoek, Namibia, ^7^Columbia University, Epidemiology and Medicine, Hastings‐on‐Hudson, United States

**Background:** In 2015, Namibia implemented an Acceleration Plan rapidly scaling up HIV testing and treatment services with the goal of reaching the UNAIDS 90‐90‐90 targets by 2020. In 2017, Namibia conducted the Namibia Population‐based HIV Impact Assessment (NAMPHIA) to estimate HIV viral load suppression (VLS) and progress toward the 90‐90‐90 targets.

**Methods:** NAMPHIA was a cross‐sectional household‐based survey conducted between June and December, 2017; analyses were weighted to account for complex survey design. Eligible adults aged 15 to 64 years who consented were interviewed and offered HIV rapid testing according to national guidelines. All HIV‐seropositive (HIV+) samples were tested for viral load at a central laboratory. The 90‐90‐90 targets were defined as: (first 90) the proportion of people living with HIV (PLHIV) who reported knowing their HIV+ status; (second 90) the proportion of PLHIV who knew their status and reported being on antiretroviral therapy (ART); and (third 90) the proportion of PLHIV who reported being on ART who had measured VLS (HIV RNA <1000 copies/mL).

**Results: ** Of 16,939 participants, 2446 tested HIV+ (Overall/Total HIV prevalence=12.6%; female=15.7%, male= 9.3%). Among all PLHIV, irrespective of knowledge of HIV+ status and reported ART status, 77.4% had VLS (female VLS=81.7%, male VLS=69.6%) (Table 1). Among PLHIV, 78.8% reported knowing that they were HIV+ (female=83.1%, male=71.1%). Among PLHIV who reported knowing their HIV+ status, 95.3% reported being on ART, (female ART=96.0%, male ART=93.8%). Among PLHIV who reported ART use, 91.5% had VLS (female VLS=92.2%, male VLS=89.9%). (Table 1)

**Conclusions: **Namibia is the first country in Africa to have reached and surpassed at a national level the UNAIDS 2020 goal of having at least 73% of all people living with HIV be virally suppressed. NAMPHIA data also show that once diagnosed, a high proportion (≥ 90%) of both male and female PLHIV initiated ART and achieved VLS. While this is a remarkable accomplishment, 21.2% of PLHIV are unaware of their HIV+ status, including almost 30% of men. Strategies to improve HIV testing, particularly for men, are urgently needed to ensure Namibia's continued progress towards HIV epidemic control.

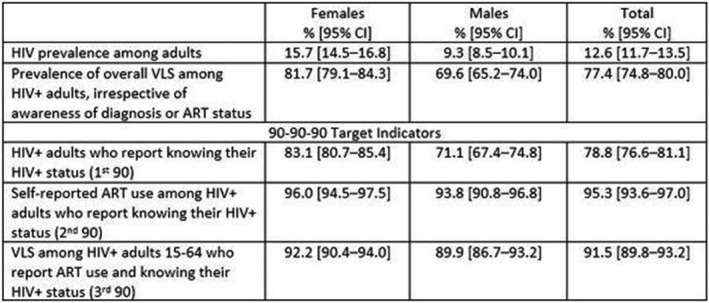

**Abstract THAC0408LB‐Table 1**

## THAD0308LB

### *Let's Stick Together*: community empowerment approach significantly impacts multiple HIV and sexual and reproductive health outcomes among female sex workers in Tanzania

**D. Kerrigan^1,2^; J. Mbwambo^3^; S. Likindikoki^3^; W. Davis^4^; A. Mantsios^2^; S. Beckham^2^; A. Leddy^5^; S. Aboud^3^ and N. Galai^2^**

^1^American University, Sociology, Washington, United States, ^2^Johns Hopkins Bloomberg School of Public Health, Baltimore, United States, ^3^MUHAS, Dar es Salaam, Tanzania, United Republic of, ^4^American University, Washington, United States, ^5^UCSF, San Francisco, United States

**Background:** Female sex workers (FSW) are 13.5 times more likely to be HIV‐infected than other women. They are also at risk for a number of other negative health outcomes and conditions, including gender‐based violence and unintended pregnancies.

**Methods:** We conducted a community‐randomized trial of a community empowerment model of combination prevention (Project *Shikamana/*Let's Stick Together) among FSW in Iringa, Tanzania. We used time‐location‐sampling to enroll a cohort of 496 women, collecting survey data and conducting HIV screening and viral load assessments at 0 and 18 months. The intervention, targeting both HIV‐infected and uninfected women, was anchored around the promotion of social cohesion and mobilization to address socio‐structural constraints such as stigma, discrimination, violence and financial insecurity. We conducted an intent‐to‐treat analysis with logistic and GEE Poisson regression.

**Results: ** Project *Shikamana* peer educators conducted 7677 outreach sessions and distributed 81,463 condoms during the 18‐month intervention period. Even without PrEP, participants in the intervention were significantly (62%) less likely to become infected with HIV at follow‐up (OR .38; *p *=* *0.05); with an HIV incidence of 5.0% in intervention vs. 10.4% in control. Consistent condom use increased significantly in the intervention (RR 1.50; *p *=* *0.01) vs. the control (RR .97; NS). The intervention also had a significant impact on reductions in gender‐based violence (RR: 0.80; *p *=* *0.05) and increases in modern family planning methods at follow‐up (RR=1.48; *p *=* *0.02). The *Shikamana* women's group has now been formally registered, established a community savings group, and started a local business to continue to support mobilization activities.

**Conclusions: **Project *Shikamana* is one of the first comprehensive models of community empowerment implemented and evaluated among FSW in Africa, which if taken to scale, holds significant promise to generate and sustain improvements in HIV‐related outcomes, sexual and reproductive health, and the human and labor rights of this key population.

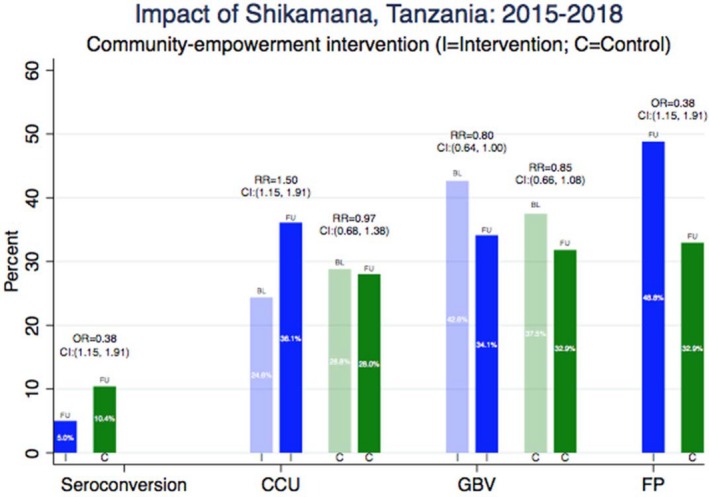

**Abstract THAD0308LB‐Figure 1. Impact of Project Shikamana on HIV, GBV and Reproductive Health Outcomes among FSW in Tanzania**

## THAE0106LB

### Integration of GBV into HIV/STI services increases uptake of post GBV care services among key populations, lessons learned from Papua New Guinea, 2015 to 2017

**I.U. Mogaba^1^; R. Nopa^2^; W. Yeka^3^; M. Dogimab^4^; D. Tesfaye^1^; E. Valaun^3^ and M. Ramsey^5^**

^1^FHI 360 (Family Health International), Papua New Guinea, Program Management (Country Office), Port Moresby, Papua New Guinea, ^2^FHI 360 (Family Health International), Papua New Guinea, Programs, Port Moresby, Papua New Guinea, ^3^FHI 360 (Family Health International), Papua New Guinea, M&E, Port Moresby, Papua New Guinea, ^4^Cardno, Papua New Guinea, Formerly with FHI 360 (Family Health International), Papua New Guinea, Port Moresby, Papua New Guinea, ^5^FHI 360 (Family Health International), Headquarters, Global Health Population and Nutriontion, Port Moresby, Papua New Guinea

**Background:** Overall adult HIV prevalence in Papua New Guinea (PNG) is 0.9% (UNAIDS, 2016), higher (1.1% to 1.68%) among key populations (KPs), including female sex workers (FSW) (14.9%), men who have sex with men (MSM)/transgender women (TG) (8.5%) (NDoH, 2017). Two‐thirds of women experienced gender‐based violence (GBV) (Darko et al., 2015), and 41% to 45% of FSW and MSM/TG reported sexual violence in the last 12 months (Kelly‐Hanku et al., 2017). GBV increases HIV risk yet decreases service utilization. Sociocultural factors inhibit GBV disclosure and service uptake for all survivors; laws criminalizing sex work and sodomy heighten this inhibition for KPs. In 2015, FHI 360 began integrating gender and GBV interventions into its USAID‐funded Strengthening HIV/AIDS for KPs in PNG project (2012 to 2018) to prevent GBV and to increase post‐GBV service uptake. Initial interventions prepared service providers to offer KP‐friendly, comprehensive post‐GBV clinical services and then moved to GBV prevention, awareness and referral among KP, communities and other service providers.

**Description:** Interventions implemented in five health facilities and the outcomes are listed below. Disaggregated descriptive and trends analyses were conducted using health facility program monitoring data (fiscal year (FY) 2015 to 2017) to understand results from routine GBV screening and post‐GBV care uptake.

**Abstract THAE0106LB‐Table 1. Interventions implemented to integrate GBV into HIV/STI services and outcomes**

**Table:** 

Interventions implemented	Outputs/outcomes
KP sensitization trainings for service providers (health care workers and GBV hotline counsellors).	Service providers: • Understand KPs’ vulnerability to violence, their rights to live lives free from violence and to access friendly and welcoming services should they experience GBV. • Become more receptive to KPs accessing GBV and other services.
Development of a GBV screening protocol; Implementation of routine GBV screening among clients accessing HIV/STI services; Introduction of a minimum package of post‐GBV care at health facilities with onward referral for non‐health services.	The 5 participating health facilities (1 in Madang Province and 4 in the National Capital District) have: • Simple one page GBV screening protocol available to all HIV service providers • The ability to provide the minimum 5 essential post‐GBV services (psychological first aid, emergency contraceptives, post‐exposure prophylaxis, prophylaxis for STIs and vaccination against tetanus and HepB). First sites offering GBV services specifically for KP. Number of all‐population GBV service sites in Port Moresby increased from 1 to 5, and from 1 to 2 in Madang Province, increasing access in a community where travel between and among neighborhoods can be dangerous.
Training of HIV/STI service providers on the GBV screening protocol and the post‐GBV care minimum package.	Health care workers screen all clients accessing STI, HIV testing and ART services and provide the minimum post‐GBV care package.
Sensitization of communities on GBV prevention/bystander interventions and post‐GBV services. Strengthening post‐GBV care referral linkages between communities and health facilities.	Community leaders aware of GBV and its relationship to HIV, and where and how to access post‐GBV care. Telephone counsellors working with GBV hotline counselling service became conversant with the referral pathways for post‐GBV services and make referrals. Increase in number of referrals (including self‐referrals) from the community.
Inclusion of GBV empowerment and safety planning in the community outreach minimum HIV prevention package. Training KP peer educators and GBV hotline counsellors towards engaging clients on GBV/post‐GBV care referral pathways.	Peer educators provide education to KPs on GBV, GBV prevention and identification and referral of survivors for post‐GBV care. A standard operating procedure on GBV prevention, recognition and referral, and GBV safety planning tool kit developed. More KPs reached with GBV prevention and post‐GBV care.
Revision of HIV data collection and reporting tools to capture relevant data on GBV screening and post‐GBV care.	Program monitoring data on GBV screening and uptake of post‐GBV care available (disaggregated by sex, KP type, KP/general population, year), including: • Number of individuals screened for GBV • Number and proportion of GBV‐survivors among those screened • Number of self‐referred GBV‐survivors • Number and proportion of GBV‐survivors receiving post‐GBV care.

**Lessons learned:** The number of GBV‐screened individuals among clients accessing HIV/STI services increased from 718 (2015) to 8426 (2017). The percentage of individuals screened and identified as GBV‐survivors remained low compared to survey results (Darko et al., 2015). The number of GBV cases expanded exponentially. The percentage of survivors seen (including walk‐ins/referrals) receiving post‐GBV care for both sexes increased sharply before a 2017 slight decline. The percentage of GBV survivors among KPs who received care increased sharply (2015 to 2017) (MSM/TG: 0% to 86.1%, FSW: 33.3% to 86.5%).

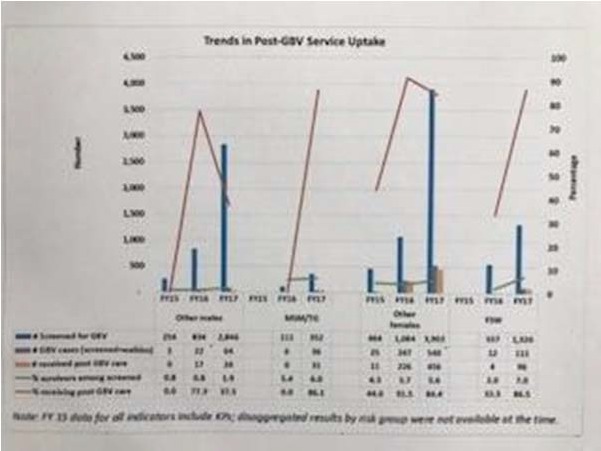

**Abstract THAE0106LB‐Figure 1. Trends in Post‐GBV Service Uptake.**

**Conclusions/Next steps:** In this context, increasing the number of service points, providing integrated KP‐friendly and comprehensive GBV services combined with community GBV awareness and referral had a dramatic impact on post‐GBV care uptake. However, proactive screening did not increase the proportion of GBV survivors identified by much, suggesting other factors exist affecting GBV survivors’ disclosure.

## FRAE0108LB

### Empowerment clubs did not increase PrEP continuation among adolescent girls and young women in South Africa and Tanzania ‐ Results from the EMPOWER randomised trial

**S. Delany‐Moretlwe^1^; M. Chersich^1^; S. Harvey^2^; A. Stangl^3^; D. Baron^1^; M. Columbini^2^; F. Scorgie^1^; N. Naicker^1^; S. Kapiga^2,4^ and EMPOWER study group**

^1^University of the Witwatersrand, Wits RHI, Johannesburg, South Africa, ^2^London School of Hygiene and Tropical Medicine, London, United Kingdom, ^3^ICRW, Washington DC, United States, ^4^Mwanza Intervention Trials Unit, Mwanza, Tanzania, United Republic of

**Background:** Adolescent girls and young women (AGYW) are likely to benefit from PrEP. Strategies to support PrEP uptake and address barriers to consistent use are urgently needed. We conducted a randomised controlled trial to evaluate whether empowerment clubs increase PrEP uptake and continuation among AGYW.

**Methods:** We enrolled sexually active, HIV‐negative women into an open‐label PrEP study. Participants were randomised to standard of care (SOC), which included comprehensive sexual and reproductive health care, with counselling and SMS reminders for PrEP users, or to empowerment clubs plus SOC. A standardised four‐session curriculum developed to support safe introduction of PrEP within relationships was delivered at monthly facilitator‐led, small group sessions. Clinic follow‐up visits were scheduled at months 1, 3 and quarterly thereafter, for up to 15 months. We used pharmacy records to measure PrEP continuation. We assessed differences in PrEP continuation using Kaplan‐Meier survival analysis, by log‐rank test.

**Results: ** From October 2016 to July 2017, 619 women were screened, 431 women enrolled (SA n = 379, Tz n = 52) and 213 randomised to clubs. Participants were mostly unmarried (90%), 27% had >1 partner in the past six months, 39% used a condom at last sex and 33% had a curable STI; most (84%) believed PrEP could prevent HIV. Of these, 408 initiated PrEP at, and 8 after, enrolment (97%) (SA n = 364, Tz n = 52). Participants completed a median of 3 follow up visits (range 0 to 6) and one club session (range 0 to 7); 48% did not attend any club sessions. In the ITT, PrEP continuation did not vary significantly by study arm (*p*‐value =0.31); PrEP continuation was 73% at M1, 61% at M3 and 34% at M6 (Figure 1). There was also no difference in PrEP continuation when comparing those that attend ≥1 club session compared to none (*p*‐value =0.12).

**Conclusions: **While PrEP uptake was high in this at‐risk population, use diminished with time. Empowerment club participation was low and did not enhance PrEP continuation, contrary to experiences in the HIV treatment field. Ongoing analyses will elucidate barriers to club participation and reasons for PrEP discontinuation and inform the development of future tailored PrEP support packages for adolescents.

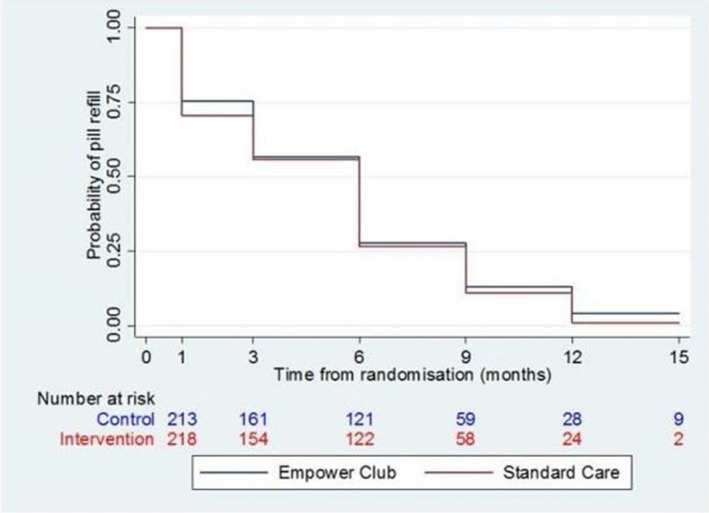

**Abstract FRAE0108LB‐Figure 1.**

## TUPDA0109LB

### Ixazomib reduces HIV‐1 reservoir size in a Casp8p41‐dependent manner

**N. Cummins^1^; S. Natesampillai^1^; Z. Nie^1^; R. Sampath^1^; J. Baker^2^; K. Henry^3^; M. Pinzone^4^; U. O'Doherty^4^; E. Polley^5^; G. Bren^1^; D. Katzmann^5^; S. Rizza^1^; A. Tande^1^; M. Mahmood^1^; J. Zeuli^5^; C. Rivera^5^; S. Kumar^5^ and A. Badley^1^**

^1^Mayo Clinic, Infectious Diseases, Rochester, United States, ^2^University of Minnesota, Minneapolis, United States, ^3^Hennepin County Medical Center, Minneapolis, United States, ^4^University of Pennsylvania, Philadelphia, United States, ^5^Mayo Clinic, Rochester, United States

**Background:** The main barrier to HIV‐1 cure is the latent reservoir that persists despite suppressive ART. Following reactivation from latency, HIV‐infected cells generate protease and the HIV‐specific cell death stimulus, Casp8p41, yet resist Casp8p41‐mediated apoptosis through neutralization by Bcl2. Since proteins that have been inactivated are often degraded by the proteasome, we investigated whether the Casp8p41‐Bcl2 complex was as well using *in vitro* and *ex vivo* models.

**Methods:** Polyubiquitination of Casp8p41 and the Casp8p41‐Bcl2 complex was assessed by immunoprecipitation and immunoblot. Expression of Casp8p41 was assessed in the presence of ixazomib by flow cytometry. Apoptosis in HIV‐1 infected cells in response to ixazomib was assessed in chronically infected J‐Lat 10.6 cells, acutely infected primary CD4 T cells, and *ex vivo* CD4 T cells from HIV‐1 infected patients. HIV reactivation was assessed in J‐Lat 10.6 cells and HIV LTR activation assessed using HIV‐LTR luciferase reporter. We have obtained an FDA IND for use of ixazomib in HIV infected patients and are enrolling a Phase 1b/2a clinical trial of ixazomib in ART‐suppressed HIV‐1 positive patients (NCT02946047).

**Results: ** Casp8p41 bound to Bcl2 is polyubiquitinated and degraded by the proteasome. Ixazomib directly (a) induces HIV reactivation in an NFkB dependant manner, (b) increases Casp8p41 and induces apoptosis of infected but not uninfected cells, and (c) decreases viral replication. *Ex vivo* treatment of CD4 T cells from ART suppressed, HIV‐1+ patients with a single dose of ixazomib reduces total cell associated HIV DNA by a median of 35% (IQR 7%, 42%) (*p* = 0.007) and integrated HIV DNA by a median of 69% (IQR 58%, 89%) (*p* < 0.001). Preliminary clinical trial results indicate that ixazomib doses of 1 to 2 mg weekly are safe and well tolerated.

**Conclusions: **The FDA‐approved proteasome inhibitor ixazomib reactivates HIV‐1, blocks degradation of HIV specific death stimulus Casp8p41, and reduces HIV reservoir size *ex vivo*. Enrollment of a Phase 1b/ 2a clinical trial of ixazomib is ongoing to evaluate the primary outcome of safety and tolerability, and secondary outcomes of HIV reservoir size and immunologic status.

## TUPDX0107LB

### Drug‐drug interactions between the use of feminizing hormone therapy and pre‐exposure prophylaxis among transgender women: The iFACT study

**A. Hiransuthikul^1^; K. Himmad^1^; S. Kerr^2,3^; N. Thammajaruk^2^; T. Pankam^1^; R. Janamnuaysook^1^; S. Mills^4^; R. Vannakit^5^; P. Phanuphak^1^; N. Phanuphak^1^ and iFACT study team**

^1^The Thai Red Cross AIDS Research Centre, Prevention, Bangkok, Thailand, ^2^HIV‐NAT, The Thai Red Cross AIDS Research Centre, Bangkok, Thailand, ^3^Kirby Institute, University of New South Wales, Sydney, Australia, ^4^LINKAGES Thailand FHI 360, Bangkok, Thailand, ^5^Office of Public Health, United States Agency for International Development, Bangkok, Thailand

**Background:** Concerns about potential drug‐drug interactions (DDI) between feminizing hormone therapy (FHT) and pre‐exposure prophylaxis (PrEP) have hampered uptake and adherence of PrEP among transgender women (TGW). To determine DDI between FHT and PrEP, we measured pharmacokinetic parameters of blood plasma tenofovir (TFV), estradiol (E2), and testosterone.

**Methods:** Twenty TGW who never underwent orchiectomy and had not received injectable FHT within 6 months were enrolled between January and March 2018. FHT (estradiol valerate 2 mg and cyproterone acetate 25 mg) were prescribed to participants at baseline until week 5, and week 8 until the end of study. PrEP (tenofovir disoproxil fumarate 300 mg/emtricitabine 200 mg) was initiated at week 3 and continued without interruption. Intensive E2 pharmacokinetic parameters and trough serum testosterone concentration (Ctrough) were measured at weeks 3 and 5 (assessing DDI between PrEP and FHT), and intensive TFV pharmacokinetic parameters were measured at weeks 5 and 8 (assessing DDI between FHT and PrEP).

Absract TUPDX0107LB‐Figure 1. iFACT study scheme.

**Results: ** Median (IQR) age, BMI, and CrCl were 21.5 (21 to 26) years, 20.6 (19.0 to 22.4) kg/m^2^, and 0.86 (0.75 to 0.94) mL/min, respectively. The geometric mean (%CV) of area under curve from time zero to 24 hr (AUC0‐24), maximum concentration (Cmax), and concentration at 24 hr (C24) of E2 at weeks 3 and 5 were 775.13 (26.2) pg*h/mL, 51.47 (26.9) pg/mL, and 15.15 (42.0) pg/mL; and 782.84 (39.6), 55.76 (32.9), and 14.32 (67.4), respectively. The geometric mean (%CV) of TFV AUC0‐24, Cmax, and C24 at weeks 5 and 8 were 2.28 (26.2) mg*h/L, 0.36 (34.8) mg/L, and 0.04 (28.8) mg/L; and 2.63 (26.9), 0.32 (25.3), and 0.05 (28.0), respectively. The geometric mean of AUC0‐24 and C24 of TFV at week 5 were significantly less than that at week 8 by 13% (*p* = 0.009) and 17% (*p* < 0.001), respectively. There were no significant changes in E2 pharmacokinetic parameters and median (IQR) Ctrough of bioavailable testosterone between week 3 and 5.

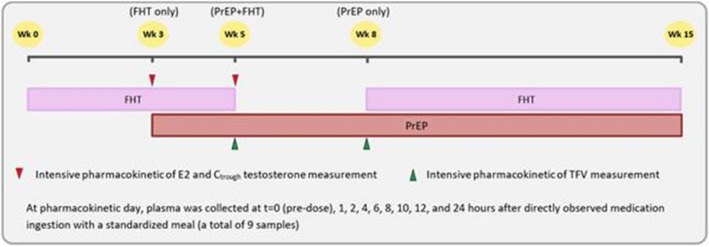

**Abstract TUPDX0107LB‐Figure 1.**

**Absract TUPDX0107LB‐Table 1. Summary of E2 and TFV pharmacokinetic parameters; data are presented in geometric mean (%CV)**

**Table:** 

E2 pharmacokinetic parameter	Week 3 (FHT only)	Week 5 (PrEP+FHT)	GMR (95%CI)	*p*‐value
AUC0‐24 (pg*h/mL)	775.13 (26.2)	782.84 (39.6)	1.01 (0.89 to 1.15)	0.88
Cmax (pg/mL)	51.47 (26.9)	55.76 (32.9)	1.08 (0.94 to 1.24)	0.25
C24 (pg/mL)	15.15 (42.0)	14.32 (67.4)	0.95 (0.75 to 1.19)	0.63
Half‐life (h)	11.25 (32.6)	11.83 (50.9)	1.05 (0.87 to 1.27)	0.60
TFV pharmacokinetic parameter	Week 5 (PrEP+FHT)	Week 8 (PrEP only)	GMR (95%CI)	*p*‐value
AUC0‐24 (mg*h/L)	2.28 (26.2)	2.63 (26.9)	0.87 (0.78 to 0.96)	0.009
Cmax (mg/L)	0.36 (34.8)	0.32 (25.3)	1.10 (0.95 to 1.28)	0.2
C24 (mg/L)	0.04 (28.8)	0.05 (28.0)	0.83 (0.76 to 0.90)	<0.001
Half‐life (h)	15.19 (15.4)	15.69 (23.0)	0.97 (0.88 to 1.07)	0.53

**Conclusions: **Our study demonstrated lower plasma TFV exposure in the presence of FHT, suggesting that FHT may potentially affect PrEP efficacy among TGW; but E2 exposure was not affected by PrEP. Further studies are warranted to determine whether these reductions in TFV are clinically significant.

## THPDC0107LB

### Diagnostic accuracy, feasibility and acceptability of HIV oral fluid rapid tests among hard‐to‐reach key populations in Latvia, the country with highest infection rates in Europe

**A. Kivite^1^; R. Kaupe^1,2^; I. Linina^1^; I. Upmace^1,3^ and HERMETIC study group**

^1^Riga Stradins University, Riga, Latvia, ^2^NGO “DIA+LOGS”, Riga, Latvia, ^3^NGO “Baltic HIV Association”, Riga, Latvia

**Background:** In Europe**,** Latvia has the highest rate of new HIV diagnoses and one of the lowest HIV testing rates. Half of the HIV cases are estimated to be undiagnosed and the proportion of late diagnoses is high (>50%). Thus, new strategies to promote HIV testing are of utmost priority.

**Methods:** For the first time oral fluid rapid tests (OraQuick®) were used to promote HIV testing outside clinical settings and stationary harm reduction sites. Between September 2017 and February 2018, 310 people who inject drugs (PWID) were tested via mobile van services and 205 men who have sex with men (MSM) via outreach work in night clubs. Participants were also tested with a capillary blood rapid test (CHIL®) to compare the two methods. Diagnostic accuracy of OraQuick® was assessed against the HIV positive serostatus, which was defined by either having a positive capillary blood rapid test or participants’ self‐reported positive HIV serostatus. Acceptability of the oral fluid tests was assessed through mixed methods, i.e. quantitative questionnaires (n = 515), 3 focus groups and 7 in‐depth interviews. Chi square or Fisher exact test were employed to analyze quantitative data, qualitative data were analyzed inductively adopting thematic analyses.

**Results:** OraQuick® tests had 84.4% sensitivity, 99.1% specificity, 94.2% positive and 97.3% negative predictive value. No significant differences were identified in accuracy measures between PWID and MSM. Contradictory test results occurred mostly among participants on anti‐retroviral therapy. MSM's awareness of the oral fluid test before the study was greater compared to PWID (32.0% vs 21.8%, *p* < 0.001). Acceptability was higher among MSM. They had greater trust in the validity of the test (*p* < 0.001) and recommended the test more often to others (*p* = 0.08). PWID trusted the capillary blood test more than the oral fluid test (*p* < 0.001). Qualitative results showed that mistrusting oral fluid test results was due to scepticism about new interventions in general and by confusion about the fact that HIV cannot be transmitted via saliva.

**Conclusions: **Accuracy of the oral fluid test was high in Latvia. Oral fluid tests were more accepted by MSM than PWID. Educational interventions should clarify the existing target‐group specific misconceptions.

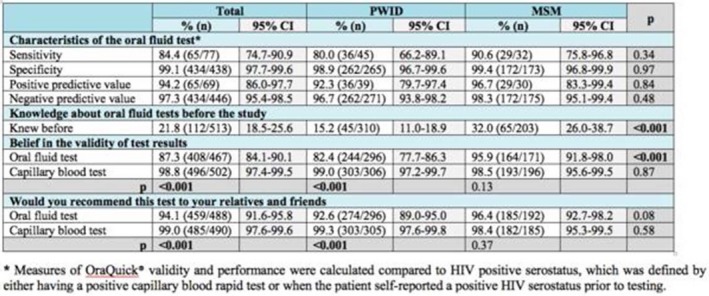

**Abstract THPDC0107LB‐Table 1. Diagnostic accuracy and acceptability of oral fluid test vs capillary blood test**

## THPDD0108LB

### Reducing health worker stigma and discrimination is critical to reaching 90‐90‐90 targets and is possible: Evaluation results of a whole‐facility approach in Ghana

**L. Nyblade^1^; N.A. Addo^2^; K. Atuahene^3^; E. Gyamera^2^; C. Stewart^1^; S. Jacinthe^4^; E. Essandoh^4^; N. Alsoufi^4^; R. Vormawor^2^ and J. Kraemer^5^**

^1^HP+/RTI International, Washington, United States, ^2^Educational Assessment and Research Center, Accra, Ghana, ^3^Ghana Aids Commission, Accra, Ghana, ^4^United States Agency for International Development, Accra, Ghana, ^5^Georgetown University, Washington, United States

**Background:** Achieving 90‐90‐90 targets requires addressing structural factors to reach people who are hidden due to stigma and discrimination(S&D) from HIV, key population or other marked status. S&D in health facilities are particularly detrimental, yet their reduction is not a standard or scaled practice in most HIV interventions or health systems. Lack of evaluated S&D‐reduction interventions is a contributing factor. The USAID‐and‐PEPFAR‐funded Health‐Policy‐Plus project has partnered with the Ghana AIDS Commission and the Educational Assessment Research Center, with support from The Global Fund, to test a whole‐facility approach to S&D‐reduction.

**Methods:** Representative baseline (n = 717 females/432 males; August 2017) and endline (n = 778 females/371 males; April 2018) surveys capturing stigma‐drivers and manifestations from health facility staff (HFS) in 10 high‐HIV caseload facilities (5 intervention matched with 5 comparison), in five regions of Ghana. Endline data was collected six months after starting the still‐ongoing intervention. We estimated before‐after trends and differences‐in‐differences by fitting generalized linear models with identity link functions and binomial error distributions. Standard errors were clustered by facility. Interventions included: capacity building for facility‐based S&D‐reduction training teams (health workers+clients); 2‐day participatory training for all staff levels (clinical+non‐clinical), targeting 70% of all facility staff; facility‐based S&D‐reduction champion teams that led onsite activities. All 10 facilities received baseline data collection and participatory data validation that included action planning.

**Results:** There were statistically significant before‐after improvements in most outcome domains in intervention facilities (Table 1/Figure 1), and smaller improvements in comparison facilities. We observed statistically significant difference‐in‐differences in reductions in unnecessary fear (23.9%; *p* < 0.001), stigmatizing avoidance behaviors (15.7%; *p* = 0.011), preferences not to treat MSM (14.2%; *p* = 0.001), as well as several facility policies. Intervention facilities’ staff were 21.5% (*p* = 0.019) more likely to report that behavior toward patients was much better at follow‐up than comparison staff. Key intervention ingredients include: data to define the problem and catalyze action; building facility ownership and facility‐led response; participatory training; empowered HFS champions.

**Conclusions: **S&D‐reduction interventions targeting the whole facility are feasible, welcomed, and stimuli for change in a short time frame (6‐months). Reducing S&D in health facilities is critical to improving quality of care to ensure equitable access to services for all.

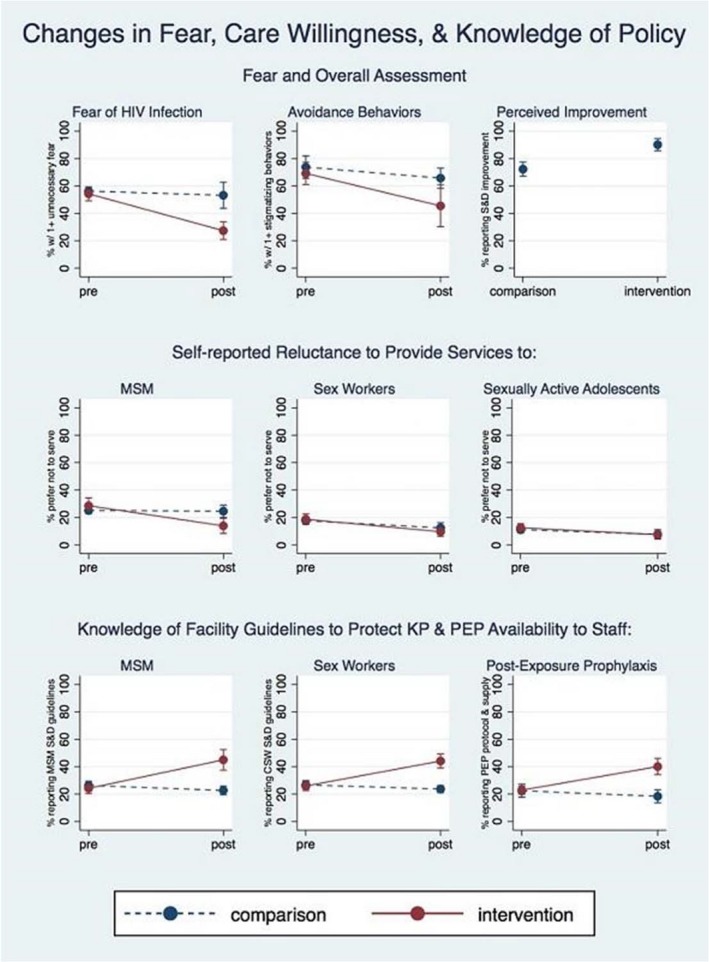

**Abstract THPDD0108LB‐Figure 1. Changes in Fear, Care Willingness, and Knowledge of Policy.**

**Abstract THPDD0108LB‐Table 1. Selected difference‐in‐differences (change) in pre‐post data collection, comparing intervention (n = 1198) to comparison (n = 1110) health facilities**

**Table:** 

S&D indicator	% difference in change between intervention and comparison facilities in non‐stigmatizing direction	Significance (*p*‐value)	95% Confidence Interval	S&D indicator	% difference in change between intervention and comparison facilities in non‐stigmatizing direction	Significance (*p*‐value)	95% Confidence Interval
Fear of HIV transmission during routine care for people living with HIV				Routinely engaging in unnecessary and stigmatizing avoidance behaviors with clients living with HIV			
Touching clothing of a client living with HIV (n = 1848)	11.25%	0.037	7.0, 21.8	Avoid physical contact (n = 1827)	1.7%	0.367	−2.0, 5.42
Dressing wounds of a client living with HIV (n = 1513)	18.92%	0.001	7.46, 30.39	Wear double gloves (n = 1673)	16.73%	0.001	6.90, 26.56
Drawing blood from a client living with HIV (n = 1588)	23.29%	<0.001	14.53, 32.04	Wear gloves during all aspects of the patient´s care (n = 1698)	4.2%	0.320	−3.92, 11.97
Taking the temperature of a client living with HIV (n = 1613)	6.7%	0.184	−3.21, 16.67	Use extra precautionary measures only with clients living with HIV (n = 1678)	24.67%	<0.001	17.46, 31.88
Composite: at least one of the above (n = 1958)	23.86%	<0.001	10.69, 37.04	Composite: usually engage in at least one of the avoidance behaviors (n = 1940)	15.65%	0.011	3.61, 27.70
Own preference to not treat men who have sex with men				Own preference to not treat sex workers			
Men who have sex with men (n = 2275)	14.2%	0.001	5.71, 22.59	Sex workers (n = 2274)	3.8%	0.238	−2.51, 10.08

